# RSEQREP: RNA-Seq Reports, an open-source cloud-enabled framework for reproducible RNA-Seq data processing, analysis, and result reporting

**DOI:** 10.12688/f1000research.13049.2

**Published:** 2018-04-13

**Authors:** Travis L. Jensen, Michael Frasketi, Kevin Conway, Leigh Villarroel, Heather Hill, Konstantinos Krampis, Johannes B. Goll

**Affiliations:** 1 Vaccine and Infectious Disease Department , The Emmes Corporation, Rockville, MD, USA; 2 IT Operations, The Emmes Corporation, Rockville, MD, USA; 3 Department of Biological Sciences, Hunter College, City University of New York, New York, NY, USA

**Keywords:** RSEQREP, RNA-Seq, transcriptomics, differential gene expression, pathway enrichment, reproducible research, cloud computing, trivalent influenza vaccine

## Abstract

RNA-Seq is increasingly being used to measure human RNA expression on a genome-wide scale. Expression profiles can be interrogated to identify and functionally characterize treatment-responsive genes. Ultimately, such controlled studies promise to reveal insights into molecular mechanisms of treatment effects, identify biomarkers, and realize personalized medicine. RNA-Seq Reports (RSEQREP) is a new open-source cloud-enabled framework that allows users to execute start-to-end gene-level RNA-Seq analysis on a preconfigured RSEQREP Amazon Virtual Machine Image (AMI) hosted by AWS or on their own Ubuntu Linux machine via a Docker container or installation script. The framework works with unstranded, stranded, and paired-end sequence FASTQ files stored locally, on Amazon Simple Storage Service (S3), or at the Sequence Read Archive (SRA). RSEQREP automatically executes a series of customizable steps including reference alignment, CRAM compression, reference alignment QC, data normalization, multivariate data visualization, identification of differentially expressed genes, heatmaps, co-expressed gene clusters, enriched pathways, and a series of custom visualizations. The framework outputs a file collection that includes a dynamically generated PDF report using R, knitr, and LaTeX, as well as publication-ready table and figure files. A user-friendly configuration file handles sample metadata entry, processing, analysis, and reporting options. The configuration supports time series RNA-Seq experimental designs with at least one pre- and one post-treatment sample for each subject, as well as multiple treatment groups and specimen types. All RSEQREP analyses components are built using open-source R code and R/Bioconductor packages allowing for further customization. As a use case, we provide RSEQREP results for a trivalent influenza vaccine (TIV) RNA-Seq study that collected 1 pre-TIV and 10 post-TIV vaccination samples (days 1-10) for 5 subjects and two specimen types (peripheral blood mononuclear cells and B-cells).

## Introduction

The advent of next-generation sequencing (NGS) technologies has dramatically reduced costs and thus democratized sequencing ^1^. Consequently, both big research consortia and small laboratories now have the ability to utilize large-scale genomic applications such as RNA sequencing (RNA-Seq) for transcriptome profiling. However, while sequencing cost is on the decline, the cost of data storage, analysis and interpretation is increasing ^1^. Major challenges for analyses of RNA-Seq data include the need for a substantial informatics hardware and software infrastructure as well as a wide range of computational skills to effectively manage and process the data. With the plethora of published bioinformatics software, data formats, and human genome information, careful bioinformatics workflow development, parameterization, reference dataset management, and execution are required to generate consistent, reproducible and high-quality analysis datasets ^2^. Interpretation of RNA-Seq data requires special statistical and visualization techniques ^3, 4^. In addition, most of the NGS bioinformatics software only runs on the Linux operating system (OS) or is dependent on Linux tools/utilities. These requirements limit the ability of small labs and individual principal investigators to analyze such data, in particular, those that use desktop computers running non-Linux based OS with limited IT support. Emerging information technologies, bioinformatics workflow engines, and open-source analytical modules are presenting opportunities to reduce this burden ^5^. Virtualization technologies, for example, now allow entire OS replete with all the necessary software packages to be archived and then instantiated just about anywhere at a moment’s notice, independent of the hardware architecture available. For instance, all software components and dependencies can be encapsulated within Virtual Machines (VMs). A more lightweight approach to bundle software are Docker containers. Compared to VMs, Docker containers execute processes directly on top of the kernel of a host OS, and thus, unlike VMs, they do not require an OS to be encapsulated. Furthermore, they require minimal installation effort, while also providing a mechanism for software version tracking, update, and configuration. Using virtual appliances allows users to choose the number and size of VMs to be provisioned and thus provide on-demand computational scalability. Commercial cloud service providers such as Amazon Web Services, Google Cloud Platform, and Windows Azure provide user-friendly web-based tools to manage VMs and associated computational resources, including cloud storage, networking, security, identity management, and backup and disaster recovery. This pay-as-you go model eliminates upfront capital expenses by converting the budgeting representation of bioinformatics processing tasks and storage into well-defined operational costs. The open-source R statistical programming language in combination with the Bioconductor package resource provides researchers with a consistent way to share and use specialized statistical methods for RNA-Seq analysis ^6, 7^. In combination with the R knitr package, analysis data sets can be processed automatically using R and summarized in reports by integrating formatting instructions with analytical components ^8^. Together, these technologies can reduce analysis time and programming effort, allow more accurate estimation of hardware costs, improve quality of results, and facilitate reproducible research by transparently documenting all steps including software and OS.

RNA-Seq allows snapshot measurements of the human transcriptome by partially sequencing reverse-transcribed RNA transcripts (cDNA) expressed in cell populations or single cells of interest. In the context of clinical trials, the goal of transcriptomics studies is to identify and better understand changes in cell states on the gene expression level that can be attributed to a certain treatment (e.g., a vaccine or drug) ^9, 10^, or changes that can predict individual treatment responses (e.g. the likelihood of developing protective levels of antibody) ^11, 12^. The number of RNA-Seq reads (short DNA sequence) corresponding to a transcript has been shown to be linearly associated with true transcript abundance spanning a large quantitative range ^13^. Prior to gene expression quantification, processing of human RNA-Seq data requires a computationally intensive alignment step that maps sequence reads against the human reference transcriptome and/or genome sequence ^14– 16^. Resulting alignment metrics including genomic mapping locations (chromosome and position), alignment information (insertions, deletions, and matching bases), alignment quality scores, among other information, are recorded in the form of Binary Alignment Mapping (BAM) files ^17^. Various algorithms have been developed that use this mapping information for determining/counting which sequence read originated from a certain gene, gene isoform, or gene exon ^18– 22^. Following gene expression quantification, key analysis steps include the detection of treatment-responsive genes (e.g. 4) and subsequent characterization of these genes using pathway enrichment analysis (e.g. 23). Challenges prior to RNA-Seq data interpretation include (1) estimation of expected cost for storage and data processing, (2) provisioning of computational resources for storage and data processing, (3) installation of Linux OS, required bioinformatics software, and reference data sets, (4) suitable analytical methods including advanced data visualizations to summarize key tends in the data, and (5) automation and documentation of all steps to facilitate reproducible research.

In this article, we summarize the RSEQREP framework we developed that allows researchers to address these challenges and to streamline the transition from a desktop environment to a server-based scalable cloud infrastructure using Amazon Web Services (AWS). Alternatively, the framework can be installed on a local Ubuntu machine via a RSEQREP Docker container or installation scripts that we provide. We exemplify the framework’s capabilities using RNA-Seq data generated for an influenza vaccine study that extracted RNA from peripheral blood mononuclear cells (PBMCs) and B-cells samples collected from 5 subjects prior to trivalent influenza vaccine (TIV) vaccination and at 10 time points post TIV vaccination (days 1-10) (GEO accession: GSE45764, Dataset 1, ^10^).

## Methods

### Implementation

**Figure 1.**
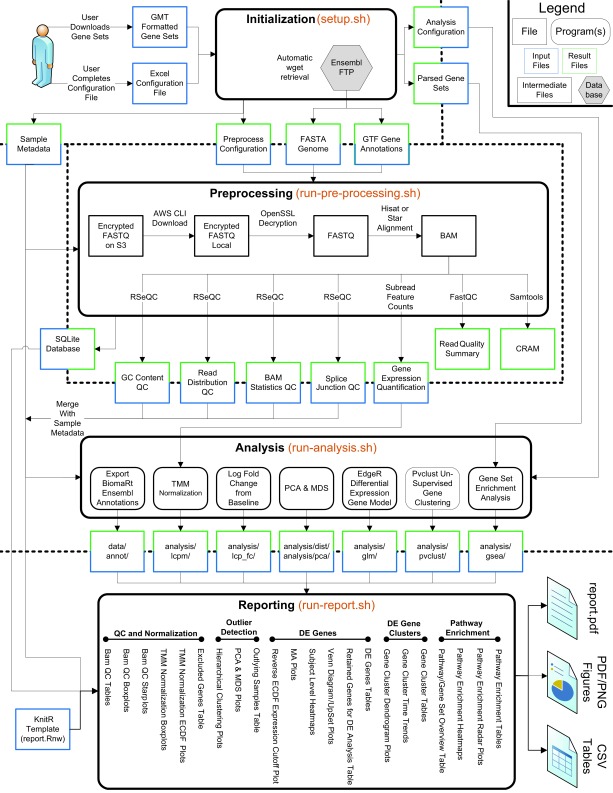
RNA-Seq Reports (RSEQREP) implementation overview. RSEQREP provides a reproducible start-to-end analysis solution for RNA-Seq data by automating (1) reference dataset initialization/download, (2) RNA-Seq data processing (3) RNA-Seq analysis, and (4) reporting including a summary PDF report and publication-ready table and figure files. Steps can be run in a modular fashion and key computational metrics are tracked in a SQLite database. The software runs on a pre-configured RSEQREP AMI or on a local Ubuntu Linux machine. Users can customize individual steps and enter their experimental design information via an Excel configuration file.

Figure 1 provides an overview of RSEQREP software components. The framework is organized into four main components: (1) reference data setup, (2) pre-processing, (3) analysis, and (4) reporting. The pre-processing component uses a combination of open-source software, shell, R, and Perl scripts and a SQLite relational database to process raw sequence data, quantify gene expression, and track storage, file check-sums, CPU, memory, and other runtime metrics. The analysis component is based on R using both custom R programs, as well as existing R/Bioconductor packages. The reporting component is based on R, the knitr R package, and LaTeX for reproducible and automatic PDF report and figure/table generation. All components read user-defined arguments from the respective tab in the RSEQREP configuration spreadsheet ( *RSEQREP/config/config.xls*).

### Operation

All four workflow components can be run in sequence via the *RSEQREP/run-all.sh* script or run individually to update results of the respective component. When running each individual step, the most recent version of the configuration file will be reloaded to ensure that any modifications to the configuration will be reflected. This is particularly useful for optimizing results and customizing result presentation, for example, by removing outliers, optimizing the low-expression cut-off, or adjusting the color-coding range for heatmaps. In the following, we provide an overview of each of these steps. Additional information can be found in the method section of the RSEQREP summary report ( Supplementary File S1).

Step 1) Reference Data Set-up. The *RSEQREP/setup.sh* script reads all user-specified arguments provided in the config.xls file, downloads all required reference data including user-specified versions of the human reference genome sequence and associated gene model information from the Ensembl database ^24^. Input for pathway enrichment analysis is handled via Gene Matrix Transposed (GMT) files. For GMT files, Entrez Gene IDs, Ensembl Gene IDs, or gene symbols are supported and will be automatically mapped to the human Ensembl reference annotations. We recommend that users obtain reference pathway GMT files from the Molecular Signatures Database (MSigDB) ^25^. The MSigDB import is not automated as download requires registration but the location of downloaded GMT file can be specified in the configuration file. We do provide a script ( *RSEQREP/source/shell/download-gene-sets.sh*) to automatically download Reactome, Blood Transcription Module ^26^, and KEGG ^27^ pathway information and convert this information to GMT files (note, a license may be required prior to downloading KEGG pathway information). Following the reference dataset download, an index of the human reference genome sequence will be created to optimize reference alignment searches ^15, 16^. Result files generated as part of this step are saved under the data output directory.

Step 2) Data Pre-processing. Based on FASTQ file input specifications in the config.xls, the *RSEQREP/run-pre-processing.sh* script downloads and decrypts (optional) FASTQ files hosted on AWS Simple Storage Service (S3) storage ( https://aws.amazon.com/s3), a local file location (Linux file path), or directly from Sequence Read Archive (SRA) ^28^ via the fastq-dump utility that is included in the SRA toolkit. Following the download, the script executes sequence data QC (FastQC), reference genome alignments (STAR ^16^ or HISAT2 ^15^ splice-aware aligner on stranded, unstranded, or paired-end read data as specified in the config.xls), reference based compression to generate storage-optimized CRAM files (SAMtools ^17^), gene expression quantification (featureCounts as implemented in subread ^18^), and reference genome alignment QC (RSeQC ^29^). Additionally, the script tracks program arguments, program return codes, input and output file names, file sizes, MDS checksums, wall clock times, CPU times and memory consumption in a SQLite relational database. Interim result files generated as part of this step are saved under the specified pre-processing output directory.

Step 3) Data Analysis. The *RSEQREP/run-analysis.sh* script initializes analysis datasets for the final reporting step including (1) TMM-normalization ^30^ and exclusion of low-expressed genes, (2) principal component analysis (PCA), distance matrix calculations for non-metric multidimensional scaling (MDS), and hierarchical clustering for global multivariate analyses, (3) log2 fold change calculations used as input for heatmap and co-expressed gene-cluster analyses, (4) identification of differentially expressed (DE) genes (edgeR ^31^), co-expressed gene clusters (pvclust ^32^), and enriched pathways (GoSeq ^23^). Interim result files generated as part of this step are saved under the specified report output directory.

Step 4) Automatic Report Generation. The *RSEQREP/run-report.sh* script produces the final results. It runs R analyses on the intermediate analysis files generated in Step 3, generates a summary PDF report using the knitr R package in combination with LaTeX, and result tables in gzipped .csv format as well as individual figure files in .pdf, and .png format. This script also summarizes key run time statistics that were collected as part of Step 2. Result files generated as part of this step are saved under the specified report output directory.

### Minimal system requirements

A 35 GiB Elastic Block Store (EBS) volume, i.e. storage immediately accessible to the OS ( http://docs.aws.amazon.com/AWSEC2/latest/UserGuide/EBSVolumes.html), sufficiently covers space for the OS, user accounts, reference data, and to process and analyze dataset sizes similar to that of the influenza vaccine case study when CRAM compression is deactivated. To accommodate storage for CRAM-compressed files and studies with larger sample sizes and/or sequence coverage, additional EBS volumes are required (see information on AWS set-up under https://aws.amazon.com/ebs/getting-started).

We found that a c3.xlarge computational Elastic Compute Cloud (EC2) instance type (4 vCPUs, 7.5 GiB, https://aws.amazon.com/ec2/instance-types) is sufficient for data processing and analysis, but a higher memory machine (c3.4xlarge: 16 Gib for HISAT2 and c3.8xlarge: 37 Gib for STAR) is required to successfully complete the indexing of the reference genome sequence as part of Step 1.

### Installation

We provide a pre-configured RSEQREP Amazon Virtual Machine Image available on AWS at ( https://aws.amazon.com, AMI ID: RSEQREP (RNA-Seq Reports) v1.0) that combines the Ubuntu operating system Version 16.04.2 (long-term support) with all additional software that is required for RSEQREP operation ( *RSEQREP/SOFTWARE.xlsx*). We prepared a manual that provides instructions on how to set-up an AWS instance including mounting of EBS volumes for local storage and an optional Elastic IP address for machine access ( *RSEQREP/aws/aws_instructions.docx*). Alternatively, we provide a RSEQREP Docker container ( https://hub.docker.com/r/emmesdock/rseqrep) and installation scripts that can be executed on a local Ubuntu machine (Version 16.04.2) to install necessary dependencies ( *RSEQREP/ubuntu/install-software.sh*). In both cases, AWS and local set-up, prior to workflow execution, users would need to pull the latest RSEQREP source code from GitHub (git clone https://github.com/emmesgit/RSEQREP).

### Configuration

RSEQREP configuration is handled via the *RSEQREP/config/config.xlsx* file. The first tab allows users to specify sample metadata. Fields include subject ID, sample ID, sampling time point, a flag (is_baseline) that indicates if a sample was collected prior to treatment, the treatment group, specimen type (e.g. B-cells, PBMCs, etc.), and FASTQ sequence file location (AWS S3, local, SRA ID via the fastq-dump utility that is part of the SRA toolkit). In addition, color-coding for time points, treatment groups, and specimen types can be defined. The second tab specifies options related to the pre-processing step. This tab uses a two-column key value pair format to define options. For example, to specify the Ensembl database version 87, users can set the version value via the ensembl_version key value pair to 74. Other options include the type of RNA-Seq data (stranded: yes/no) and reference alignment software (Star or Hisat2). Paired-end experiments can be accommodated for each sample by specifying two input FASTQ files. The third tab allows users to customize analysis and reporting components. Options include specification of cut-offs to define lowly-expressed genes, DE genes, and enriched pathways, as well as the distance metric and hierarchical clustering algorithm used for heatmap and gene clustering analysis. For further information, see descriptions and examples for each of these options in the influenza vaccine case study configuration file ( Supplementary File S2). We implemented the framework to dynamically adjust the report presentation depending on the number of subjects, time points, specimen types, and treatment group combinations. For example, Venn diagrams are shown for comparisons between up to five sets (e.g. five time points). Larger sets are accommodated via UpSet plots ^33^. The configuration file allows users to carry out subgroup analysis by limiting the metadata file to samples, treatment groups, and time points of interest.

## Use case

To illustrate the capabilities of RSEQREP, we analyzed a publicly available RNA-Seq dataset comprising 110 RNA-Seq samples: five subjects, 11 time points (pre-vaccination and days 1-10 post-vaccination), two specimen types (PBMCs and B-cells), and one treatment group (Trivalent Influenza Vaccination (TIV)) (GEO accession: GSE45764, Dataset 1, ^10^). The unstranded single-end RNA-Seq experiment was carried out with a read length of 65 nt (nucleotides) and an average sequence coverage of 12 million total mapped reads. The study was designed to obtain detailed information on the early temporal gene expression response following TIV vaccination in both PBMC and B-cells. The configuration file that specifies the case study experimental design, SRA identifiers, data processing and analysis parameters is provided in Supplementary File S2. The configuration file allows users to reproduce RSEQREP results for this case study on their own RSEQREP AWS instance or Ubuntu Linux machine. Supplementary File S1 represents the corresponding RSEQREP Summary PDF report, including 134 figures and 135 tables. In the following, we describe a subset of key findings (referenced supplemental tables and figures refer to the corresponding results in the supplemental PDF report). See Supplementary File S1 methods for additional information on pre-processing and analysis steps.

### Global gene expression patterns and DE gene time trends

**Figure 2.**
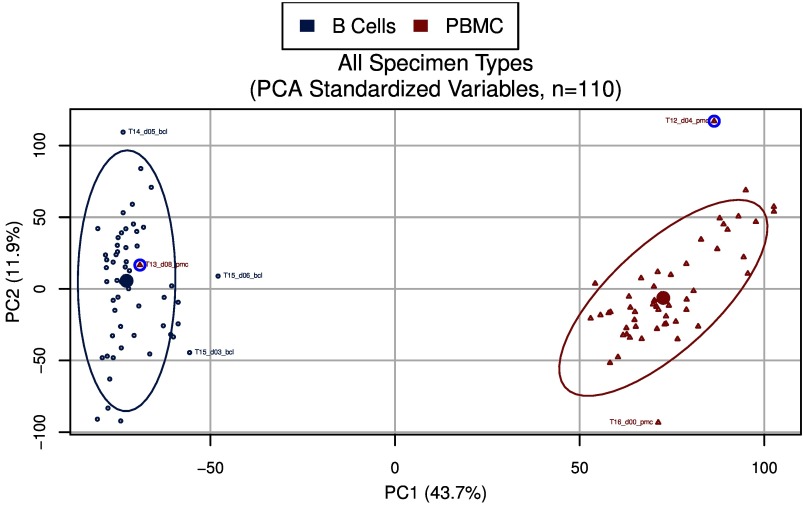
Global gene expression pattern analysis to identify outliers and batch effects (influenza vaccine case study). RSEQREP supports multivariate visualizations, including principal component analysis (PCA) to visualize key trends in the data. The analysis uses standardized log _2_ counts per million (mapped reads) for genes that met the low expression cut off as input. As shown for the influenza case study, the PCA analysis indicated that PBMC (highlighted in red) and B-cell (highlighted in blue) samples differ substantially in their transcriptional profiles. In addition, two outliers were identified in relation to the other samples (highlighted in blue circles). Ellipses represent the 95% confidence interval for the bivariate mean based on the first two principal components by specimen type.

**Figure 3.**
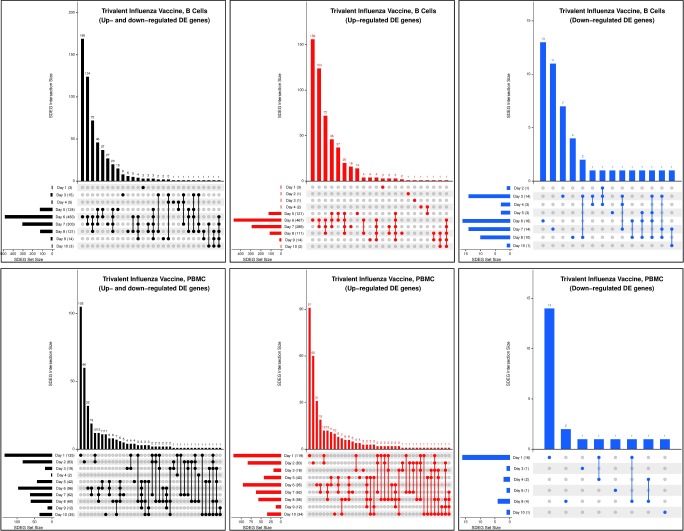
UpSet plots to summarize key differentially expressed (DE) gene time trends (influenza vaccine case study). These panels summarize the DE gene overlap between post-treatment days for up- or down-regulated DE genes (shown to the right in black), for up-regulated DE genes (shown in the middle in red), and down-regulated DE genes (shown to the right in blue), respectively within specimen type (B-cells are shown in the top row, PBMCS in the bottom row). In each panel, the bottom left horizontal bar graph labeled SDEG Set Size shows the total number of DE genes per post-treatment time point. The circles in each panel’s matrix represent what would be the different Venn diagram sections (unique and overlapping DE genes). Connected circles indicate a certain intersection of DE genes between post-treatment days. The top bar graph in each panel summarizes the number of DE genes for each unique or overlapping combination. In the top left panel, for example, the first vertical bar/column shows those DE genes that are unique to day 6 (169 DE genes). The second shows those DE genes that are shared only between days 6 and 7 (124 DE genes). The third are those DE genes that are shared between days 6, 7, and 8 (72 DE genes), and so forth. As shown for the influenza case study, most of the DE genes for B-cells were detected and overlapped between days 5, 6, 7, or 8 while most of the DE genes for PBMCs were uniquely identified at day 1.

PCA results revealed that most variation in gene expression based on standardized log _2_ counts per million across all 110 samples was attributable to cell type (B-cells vs. PBMCs, Figure 2). In addition, two extreme outliers, including one B-cell sample that was likely mislabel as a PBMC sample, were identified. These samples were added to the configuration file as outliers to be excluded from downstream analysis. Negative binomial models as implemented in the edgeR package ^31^ were fit to identify genes that were DE compared to pre-vaccination at each of the post-vaccination days. UpSet plots visualizing the number and overlap of DE genes over time are presented in Figure 3. PBMCs showed overall peak DE responses at day 1 (24 hours after TIV vaccination) with 135 genes being DE compared to pre-treatment gene expression levels. Between days 1–4, PBMC DE signals declined followed by a broader second peak response for days 5–8 reaching the second highest response of 96 DE genes at day 6. While most DE genes in PBMCs at day 1 were unique (105 of 135 genes (78%)), most DE genes at day 6 (64 of 96 (67%)) were overlapping with other DE gene responses, in particular, with days 5, 7, and 8. In contrast to PBMCs, B-cells did not exhibit a noticeable DE gene signal at day 1, but showed responses between days 5–8 (121–483 genes) reaching highest responses at day 6 (483 genes). While some DE genes were unique to day 6 (169 of 483 (35%)), many were shared with day 7 (124 genes), as well as day 7 and day 8 (72 genes). For both cell types, most DE genes were up-regulated from pre-vaccination ( Figure 3, middle panel vs. right panel). Most of the overlap between PBMC and B-cell DE genes was observed at day 6, at which 62 of 96 DE PBMC genes (65%) were also reported as DE in B-cells ( Figure S38). Tables S7–S26 list individual DE gene results. In the following, pathway enrichment analysis results for peak DE responses and a selection of identified co-expressed gene clusters are summarized.

### Pathway enrichment analysis results

**Figure 4.**
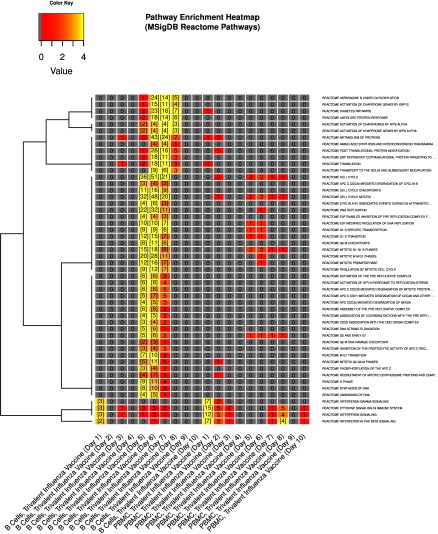
Heatmaps for visualizing pathway enrichment over time (influenza vaccine case study). Reactome pathways that were enriched in at least two conditions are shown. Cells are color-coded by enrichment score: -1 × log _10_(FDR-adjusted p-value). Cell values represent the number of DE genes that overlap with a certain pathway. Numbers in brackets indicate enriched pathways, i.e. pathways that met the specified FDR-adjusted p-value cut off. Pathways were clustered based on enrichment score. As shown for the influenza case study, pathways related to cell-cycle as well as protein metabolism were enriched in B-cells at day 6. Both, B-cell and PBMCs showed an enrichment of interferon signaling-related pathways at day 1.

To functionally characterize DE gene responses, pathway enrichment analysis as implemented in the GoSeq R package ^23^ was carried out using MSigDB (Version 5.2, Dataset 2) and Blood Transcription Modules ( Dataset 3) reference gene sets/pathways. Pathway enrichment analysis of the day 1 peak DE gene signal in PBMCs identified innate immune response signaling pathways including Reactome-based *interferon signaling*, in particular, *interferon gamma signaling* and *interferon alpha/beta signaling* ( Figure 4, Table S97). Top enriched GO Biological processes included *innate immune response*, *defense response to virus* and *response to type I interferon (* Table S92). The top Blood Transcription Modules indicated that day 1 PBMC DE genes were most preferentially *enriched in monocytes (II) (M11.0)* but also *enriched in activated dendritic cells (II) (M165)*, and *enriched in neutrophils (I) (M37.1)* ( Table S91). The day 6 PBMC DE gene signal was related to plasmablast and B-cell Blood Transcription Module signatures including *plasma cells, immunoglobulins (M156.1)*, *plasma cells and B cells, immunoglobulins (M156.0)*, and *enriched in B-cells (II) (M47.1)* ( Table S115). The day 6 peak DE gene response in B-cells was enriched in several cell cycle-related pathways including Reactome *cell cycle mitotic*, *cell cycle* and *DNA replication* ( Figure 4, Table S73). In addition, processes involved in protein processing such as GO Cellular Component *endoplasmic reticulum part* and *endoplasmic reticulum (* Table S69) and GO Biological Process *protein complex assembly* and *intracellular protein transport* ( Table S68), as well as Reactome *metabolism of proteins*, *post-translational protein modification*, and *asparagine N-linked glycosylation* were identified ( Figure 4, Table S73). Enrichment results based on Blood Transcription Modules confirmed enrichment of cell cycle-related modules but also identified several plasma cell-related signatures such as *plasma cells surface signature (S3)*, *plasma cells and B cells, immunoglobulins (M156.0)*, and *plasma cells, immunoglobulins (M156.1)* ( Table S67). The top most enriched MSigDB Immunological Signature was related to genes that were up-regulated at day 7 following TIV vaccination compared to pre-vaccination in a previous influenza vaccine study by Nakaya *et al.* (GEO accession: GSE29614, 34) ( Table S70). Tables S50–S133 list all pathway enrichment analysis results.

### Co-expressed gene cluster results

**Figure 5.**
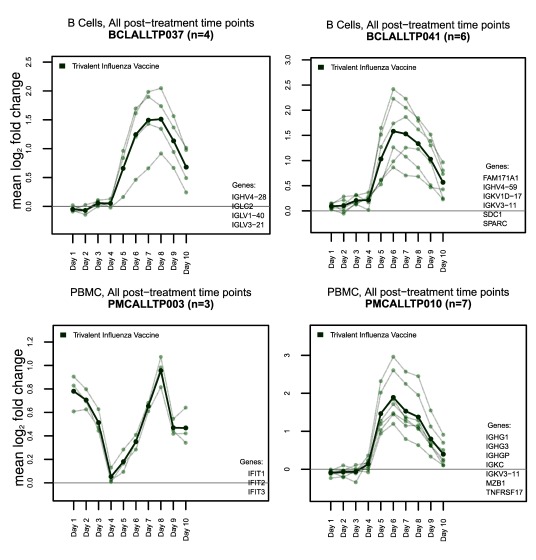
Co-expressed gene cluster time trends (influenza vaccine case study). RSEQREP supports unsupervised multiscale bootstrap resampling to identify co-expressed gene clusters based on their log _2_ fold change pattern over time. A subset of trends is shown for the influenza case study. Several co-expressed immunoglobulin genes reached peak *log*_2_ fold changes compared to pre-treatment between day 6 and 8 while a cluster of interferon-induced antiviral ( *IFIT*) genes showed an earlier peak in log _2_ fold change at day 1 in addition to a peak at day 8 in PBMCs.

To identify robust clusters of co-expressed DE genes based on correlation between log _2_ fold change responses, unsupervised multi-scale bootstrap resampling as implemented in the pvclust R package ^32^ was executed. Several known immuno-globulin genes had robustly correlated log _2_ fold changes across all post-vaccination days (day 1–10) in B-cells and PBMCs reaching peak mean log _2_ fold change responses between days 6 and 8 ( Figure 5). The immunoglobulin gene cluster highlighted for PBMCs comprised 7 genes (5 immunoglobulin genes: *IGHG1*, *IGHG3*, *IGHGP*, *IGKC*, *IGKV3-11* and 2 genes not encoding for immunoglobulins: *MZB1*, and *TNFRSF17*) ( Figure 5 bottom right). *MZB1* is known to play a role in IgM assembly and secretion while *TNFRSF17* is known to regulate humoral immunity including plasma cells. Several known interferon-inducible genes co-expressed in PBMCs ( *IFIT1*, *IFIT2*, and *IFIT3*) showed an initial peak in log _2_ fold change response at day 1, which declined to pre-vaccination levels by day 4, followed by a second higher peak response at day 8 ( Figure 5 bottom left). Time trends for all identified gene clusters are shown in Figures S82–S89.

## Discussion

There is an increasing trend towards more open and transparent research including increasing demands for sharing of source code, software snapshots as well as enhanced scalability to facilitate processing of increasingly larger datasets. A plethora of open-source software for RNA-Seq data processing and analysis has been developed ^4, 35, 36^. The strength of the RSEQREP framework is its start-to-end open-source solution that combines operating system, bioinformatics software, reference data set-up, data processing, analysis, advanced data visualizations, and automatic reporting. The resulting RNA-Seq PDF reports can easily be customized, extended, and shared.

**Figure 6.**
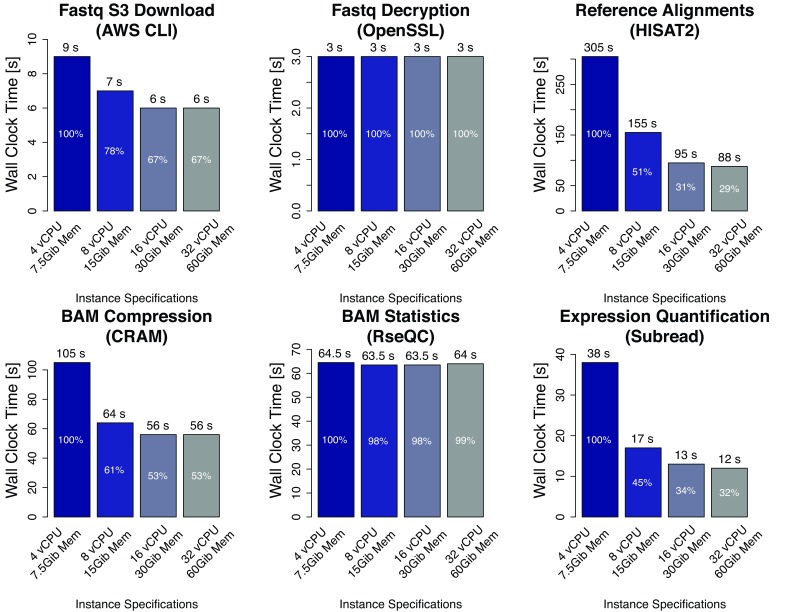
Wall clock time benchmarks for RNA-Seq pre-processing steps by AWS EC2 instance type. Metrics are based on 110 influenza case study RNA-Seq samples. The following instance types were used: c3.xlarge (4 vCPUs, 7.5 GiB Mem), c3.2xlarge (8 vCPUs, 15 GiB Mem), c3.4xlarge (16 vCPUs, 30 GiB Mem), c3.8xlarge (32 vCPUs, 60 GiB Mem). Median wall clock time is summarized as tracked in the RSEQREP SQLite database. The biggest relative reduction in wall clock time across processes was observed when switching from the 4 vCPU to the 8 vCPU instance type (c3.xlarge vs. c3.2xlarge). Higher core machines (16 and 32 vCPUs) did result in further reduced wall clock time for completing reference alignments (HISAT2) and gene expression quantification (Subread) but the change was not as substantial.

RSEQREP supports the reproducible research paradigm via its pre-configured AMI and Docker container, open-source components, user-friendly configuration file, and functionality to rerun analyses from start-to-end or in parts. Using the RSEQREP AMI, in addition to on-demand scalable computational resources, has the benefit of integrating the operating system and all software installations as part of analysis snapshots referenced in the report, providing for complete transparency and full reproducibility of all components involved. In addition, the software tracks computational runtime metrics (CPU and memory consumption), which can be used to track and estimate computational cost. Towards that end, we benchmarked the preprocessing step for the influenza vaccine case study data (110 samples) using increasingly powerful but also more expensive AWS EC2 instance types: c3.xlarge (4 vCPUs; 7.5 Gib RAM), c3.2xlarge (8 vCPUs; 15 Gib RAM), c3.4xlarge (16 vCPUs; 30 Gib RAM), and c3.8xlarge (32 vCPUs; 60 Gib RAM). We found that the c3.2xlarge (8 vCPUs; 15 Gib RAM) machine marks the ideal convergence of processing time and cost ( Figure 6).

RSEQREP includes a collection of best practice analytical tools that we identified through extensive review of the peer-reviewed literature. This includes TMM-normalization to remove systematic differences between samples ^30^, filtering of lowly expressed genes to improve accuracy of fold change estimates and power of DE detection, application of statistical methods that model read count variability using a discrete negative binomial distribution and share information across genes ^31^, the use of moderated log _2_ counts per million for multivariate analyses, and adjustment for gene length bias ^37, 38^ as part of pathway enrichment analysis ^23^. In addition, the software provides several unique visualizations, including multivariate starplots for reference alignment QC ( Figure S2), co-expressed gene cluster time trends ( Figure 5), as well as pathway enrichment heatmaps ( Figure 4) and radar plots ( Figure S120).

RNA-Seq data processing and analysis is a constantly evolving field and there is no consensus on how to best analyze the data. For example, RSEQREP summarizes gene expression on the gene level - a widely used robust gene expression quantification approach ^18, 19^. However, methods that support expression quantification on the gene-isoform level have been developed ^20– 22^. Depending on the research question, RNA-Seq analysis may include novel transcript/splice junction discovery, determination of single nucleotide polymorphism (SNPs), detection of RNA-editing events, and fusion genes ^39^. In addition, several other popular DE gene detection algorithms such as DESeq2 exist ^40^. While such additional analysis choices are currently not implemented in RSEQREP, the key advantages of this framework are that users have complete access to the source code to make custom updates to all workflow, analysis, and reporting components. In combination with scalable cloud resources this allows for rapid prototyping of analysis reports.

Using RSEQREP on published RNA-Seq data of an influenza vaccine study, we confirmed key transcriptional events in PBMCs and B-cells following TIV vaccination ^10^. Three of five subjects in this study had reported previous influenza vaccinations. A memory response was confirmed by the RSEQREP analysis, which identified an early plasma cell and cell proliferation signature in B-cells with a peak 6 days following vaccination. This signal included cluster responses for several co-expressed immunoglobulin genes as well as an up-regulation of genes preferentially involved in protein assembly, protein transport, ER-related pathways – all of which are at the core of antibody-generating cellular machinery. While not as strong as for B-cells, a peak day 6 plasma cell signature and co-expressed immunoglobulin gene response was also identified in PBMCs. This makes sense as B-cells are included in bulk PBMCs. PBMCs showed a strong up-regulation of an innate immune signaling responses 24 hours post-vaccination, in particular, responses related to interferon signaling. This signaling response was enriched in monocyte, dendritic cell, and neutrophil-specific gene expression signatures indicating that it was driven by the innate immune cell subset within PBMCs. Several co-expressed genes in the *IFIT* gene family were significantly up-regulated at day 1. These genes are known to be activated following interferon signaling and to exhibit antiviral activity by recognizing and inhibiting viral RNA ^41, 42^. This is in agreement with other studies that have shown that *IFIT genes* are up-regulated 24 hours post-influenza vaccination ^12, 43^.

## Data and software availability

RSEQREP source code available from: https://github.com/emmesgit/RSEQREP

Archived source code as at time of publication: DOI is https://doi.org/10.5281/zenodo.1211171 ^44^

RSEQREP Amazon Virtual Machine Image available from: https://aws.amazon.com, AMI ID: RSEQREP (RNA- Seq Reports) v1.0

RSEQREP Docker container available from: https://hub.docker.com/r/emmesdock/rseqrep

License: Subject to various licenses, namely, the GNU General Public License version 3 (or later), the GNU Affero General Public License version 3 (or later), and the LaTeX Project Public License v.1.3(c).

A list of the software contained in this program, including the applicable licenses, can be accessed at https://github.com/emmesgit/RSEQREP/blob/master/SOFTWARE.xlsx

**Dataset 1.** RNA-Seq of PBMC and B cell gene expression profiles in healthy humans following influenza vaccination available from NCBI GEO with accession number GSE45764.

**Dataset 2.** MSigDB Version 5.2 GMT gene set files used for the influenza vaccine case study available from:

http://software.broadinstitute.org/gsea/msigdb/download_file.jsp?filePath=/resources/msigdb/5.2/msigdb_v5.2_files_to_download_locally.zip

For MSigDB license terms, please refer to http://software.broadinstitute.org/gsea/license_terms_list.jsp. Users are requested to create a login prior to data access:

http://software.broadinstitute.org/gsea/register.jsp?next=index.jsp

**Dataset 3.** Blood Transcription Modules GMT file used for the influenza vaccine case study available from:

https://www.nature.com/articles/ni.2789#supplementary-information

(Zip file 1).

## Supplementary material

**S1:** RSEQREP Summary PDF report for influenza vaccine case study.

**S2:** RSEQREP configuration file for influenza vaccine case study.
