# Supplementary material for: RSEQREP: RNA-Seq Reports, an open-source cloud-enabled framework for reproducible RNA-Seq data processing, analysis, and result reporting
Source: F1000Res. 2018 Apr 13;6:2162. Originally published 2017 Dec 21. [Version 2] doi: 10.12688/f1000research.13049.2 (PMC6039931; doi:10.12688/f1000research.13049.2)
Supplement: Supplementary file 1 [file f1000research-6-15754-s0002.tgz › d738469f-e56d-4e5e-b0c3-a677885fb425.pdf]

RNA-Seq Report (Version 1.1.1)

RSEQREP analysis of PBMC and B cell gene expression profiles in healthy humans in response to influenza vaccination

March 25, 2018

Table of Contents

1 Synopsis **10**

2 Methods **10**

2.1 RNA-Seq experiment 10

2.2 RNA-Seq data preprocessing and data qc 10

2.2.1 Identification of differentially expressed genes 11

2.2.2 Determination of co-expressed gene clusters 11

2.2.3 Pathway enrichment analysis 11

2.3 Software 12

3 Results **12**

3.1 Figures 12

3.2 Tables 118

List of Figures

Figure 1 Boxplots of human reference genome alignment genome statistics (All specimen types) 13
Figure 2 Starplots of human reference genome alignment statistics (B Cells) 14
Figure 3 Starplots of human reference genome alignment statistics (PBMC) 15
Figure 4 Boxplots of log_2 counts per million before TMM normalization 1 of 3 (All specimen types) 16
Figure 5 Boxplots of log_2 counts per million before TMM normalization 2 of 3 (All specimen types) 17
Figure 6 Boxplots of log_2 counts per million before TMM normalization 3 of 3 (All specimen types) 18
Figure 7 Boxplots of log_2 counts per million after TMM normalization 1 of 3 (All specimen types) 19
Figure 8 Boxplots of log_2 counts per million after TMM normalization 2 of 3 (All specimen types) 20
Figure 9 Boxplots of log_2 counts per million after TMM normalization 3 of 3 (All specimen types) 21
Figure 10 Empirical cumulative distribution function plots of log_2 counts per million before and after TMM normalization 1 of 3 (All specimen types) 22
Figure 11 Empirical cumulative distribution function plots of log_2 counts per million before and after TMM normalization 2 of 3 (All specimen types) 23
Figure 12 Empirical cumulative distribution function plots of log_2 counts per million before and after TMM normalization 3 of 3 (All specimen types) 24
Figure 13 Reverse empirical cumulative distribution function plots of maximum gene expression levels across study samples (All specimen types) 24
Figure 14 PCA and non-metric multidimensional scaling biplots (All Specimen Types) 25
Figure 15 PCA and non-metric multidimensional scaling biplots (B Cells) 26
Figure 16 PCA and non-metric multidimensional scaling biplots (PBMC) 27
Figure 17 Hierarchical clustering plots (All Specimen Types) 28
Figure 18 Hierarchical clustering plots (B Cells) 29
Figure 19 Hierarchical clustering plots (PBMC) 30
Figure 20 Volcano plots (Trivalent Influenza Vaccine, B Cells) 31
Figure 21 Volcano plots (Trivalent Influenza Vaccine, PBMC) 32
Figure 22 MA plot (B Cells) 33
Figure 23 MA plot (PBMC) 34
Figure 24 UpSet plots summarizing overlap in DE genes between post-treatment time points (B Cells, Trivalent Influenza Vaccine) 35
Figure 25 UpSet plots summarizing overlap in DE genes between post-treatment time points (PBMC, Trivalent Influenza Vaccine) 35
Figure 26 UpSet plots summarizing overlap in DE genes between specimen types (Trivalent Influenza Vaccine, Day 1) 36
Figure 27 UpSet plots summarizing overlap in DE genes between specimen types (Trivalent Influenza Vaccine, Day 2) 36
Figure 28 UpSet plots summarizing overlap in DE genes between specimen types (Trivalent Influenza Vaccine, Day 3) 37
Figure 29 UpSet plots summarizing overlap in DE genes between specimen types (Trivalent Influenza Vaccine, Day 4) 37
Figure 30 UpSet plots summarizing overlap in DE genes between specimen types (Trivalent Influenza Vaccine, Day 5) 38
Figure 31 UpSet plots summarizing overlap in DE genes between specimen types (Trivalent Influenza Vaccine, Day 6) 38
Figure 32 UpSet plots summarizing overlap in DE genes between specimen types (Trivalent Influenza Vaccine, Day 7) 39
Figure 33 UpSet plots summarizing overlap in DE genes between specimen types (Trivalent Influenza Vaccine, Day 8) 39
Figure 34 UpSet plots summarizing overlap in DE genes between specimen types (Trivalent Influenza Vaccine, Day 9) 40
Figure 35 UpSet plots summarizing overlap in DE genes between specimen types (Trivalent Influenza Vaccine, Day 10) 40
Figure 36 UpSet plots summarizing overlap in DE genes between specimen types (Trivalent Influenza Vaccine, All post-treatment time points) 41

Figure 37 Venn diagrams summarizing overlap in DE genes between specimen types 1 of 3 (Trivalent Influenza Vaccine) . . . 42
Figure 38 Venn diagrams summarizing overlap in DE genes between specimen types 2 of 3 (Trivalent Influenza Vaccine) . . . 43
Figure 39 Venn diagrams summarizing overlap in DE genes between specimen types 3 of 3 (Trivalent Influenza Vaccine) . . . 44
Figure 40 Heatmap of log_2 fold change from pre-treatment (B Cells, Day 1) . . . 45
Figure 41 Heatmap of log_2 fold change from pre-treatment (B Cells, Day 2) . . . 46
Figure 42 Heatmap of log_2 fold change from pre-treatment (B Cells, Day 3) . . . 47
Figure 43 Heatmap of log_2 fold change from pre-treatment (B Cells, Day 4) . . . 48
Figure 44 Heatmap of log_2 fold change from pre-treatment (B Cells, Day 5) . . . 49
Figure 45 Heatmap of log_2 fold change from pre-treatment (B Cells, Day 6) . . . 50
Figure 46 Heatmap of log_2 fold change from pre-treatment (B Cells, Day 7) . . . 51
Figure 47 Heatmap of log_2 fold change from pre-treatment (B Cells, Day 8) . . . 52
Figure 48 Heatmap of log_2 fold change from pre-treatment (B Cells, Day 9) . . . 53
Figure 49 Heatmap of log_2 fold change from pre-treatment (B Cells, Day 10) . . . 54
Figure 50 Heatmap of log_2 fold change from pre-treatment (PBMC, Day 1) . . . 55
Figure 51 Heatmap of log_2 fold change from pre-treatment (PBMC, Day 2) . . . 56
Figure 52 Heatmap of log_2 fold change from pre-treatment (PBMC, Day 3) . . . 57
Figure 53 Heatmap of log_2 fold change from pre-treatment (PBMC, Day 4) . . . 58
Figure 54 Heatmap of log_2 fold change from pre-treatment (PBMC, Day 5) . . . 59
Figure 55 Heatmap of log_2 fold change from pre-treatment (PBMC, Day 6) . . . 60
Figure 56 Heatmap of log_2 fold change from pre-treatment (PBMC, Day 7) . . . 61
Figure 57 Heatmap of log_2 fold change from pre-treatment (PBMC, Day 8) . . . 62
Figure 58 Heatmap of log_2 fold change from pre-treatment (PBMC, Day 9) . . . 63
Figure 59 Heatmap of log_2 fold change from pre-treatment (PBMC, Day 10) . . . 64
Figure 60 Co-expressed gene cluster dendrogram with bootstrap probabilities (B Cells, Day 1) . . . 65
Figure 61 Co-expressed gene cluster dendrogram with bootstrap probabilities (B Cells, Day 2) . . . 65
Figure 62 Co-expressed gene cluster dendrogram with bootstrap probabilities (B Cells, Day 3) . . . 65
Figure 63 Co-expressed gene cluster dendrogram with bootstrap probabilities (B Cells, Day 4) . . . 66
Figure 64 Co-expressed gene cluster dendrogram with bootstrap probabilities (B Cells, Day 5) . . . 66
Figure 65 Co-expressed gene cluster dendrogram with bootstrap probabilities (B Cells, Day 6) . . . 66
Figure 66 Co-expressed gene cluster dendrogram with bootstrap probabilities (B Cells, Day 7) . . . 67
Figure 67 Co-expressed gene cluster dendrogram with bootstrap probabilities (B Cells, Day 8) . . . 67
Figure 68 Co-expressed gene cluster dendrogram with bootstrap probabilities (B Cells, Day 9) . . . 67
Figure 69 Co-expressed gene cluster dendrogram with bootstrap probabilities (B Cells, Day 10) . . . 68
Figure 70 Co-expressed gene cluster dendrogram with bootstrap probabilities (B Cells, All post-treatment time points) 68
Figure 71 Co-expressed gene cluster dendrogram with bootstrap probabilities (PBMC, Day 1) . . . 68
Figure 72 Co-expressed gene cluster dendrogram with bootstrap probabilities (PBMC, Day 2) . . . 69
Figure 73 Co-expressed gene cluster dendrogram with bootstrap probabilities (PBMC, Day 3) . . . 69
Figure 74 Co-expressed gene cluster dendrogram with bootstrap probabilities (PBMC, Day 4) . . . 69
Figure 75 Co-expressed gene cluster dendrogram with bootstrap probabilities (PBMC, Day 5) . . . 70
Figure 76 Co-expressed gene cluster dendrogram with bootstrap probabilities (PBMC, Day 6) . . . 70
Figure 77 Co-expressed gene cluster dendrogram with bootstrap probabilities (PBMC, Day 7) . . . 70
Figure 78 Co-expressed gene cluster dendrogram with bootstrap probabilities (PBMC, Day 8) . . . 71
Figure 79 Co-expressed gene cluster dendrogram with bootstrap probabilities (PBMC, Day 9) . . . 71
Figure 80 Co-expressed gene cluster dendrogram with bootstrap probabilities (PBMC, Day 10) . . . 71

Figure 81 Co-expressed gene cluster dendrogram with bootstrap probabilities (PBMC, All post-treatment time points) 72
Figure 82 Co-expressed gene cluster time trends of log_2 fold change from pre-treatment levels by treatment 1 of 5 (B Cells) . . . 73
Figure 83 Co-expressed gene cluster time trends of log_2 fold change from pre-treatment levels by treatment 2 of 5 (B Cells) . . . 74
Figure 84 Co-expressed gene cluster time trends of log_2 fold change from pre-treatment levels by treatment 3 of 5 (B Cells) . . . 75
Figure 85 Co-expressed gene cluster time trends of log_2 fold change from pre-treatment levels by treatment 4 of 5 (B Cells) . . . 76
Figure 86 Co-expressed gene cluster time trends of log_2 fold change from pre-treatment levels by treatment 5 of 5 (B Cells) . . . 77
Figure 87 Co-expressed gene cluster time trends of log_2 fold change from pre-treatment levels by treatment 1 of 3 (PBMC) . . . 78
Figure 88 Co-expressed gene cluster time trends of log_2 fold change from pre-treatment levels by treatment 2 of 3 (PBMC) . . . 79
Figure 89 Co-expressed gene cluster time trends of log_2 fold change from pre-treatment levels by treatment 3 of 3 (PBMC) . . . 80
Figure 90 UpSet plots of enriched gene sets between post-treatment time points (B Cells, Trivalent Influenza Vaccine) 81
Figure 91 UpSet plots of enriched gene sets between post-treatment time points (PBMC, Trivalent Influenza Vaccine) 82
Figure 92 UpSet plots of enriched gene sets between specimen types (Trivalent Influenza Vaccine, Blood Transcription Modules) . . . 83
Figure 93 UpSet plots of enriched gene sets between specimen types (Trivalent Influenza Vaccine, MSigDB Biological Processes) . . . 84
Figure 94 UpSet plots of enriched gene sets between specimen types (Trivalent Influenza Vaccine, MSigDB Cellular Components) . . . 85
Figure 95 UpSet plots of enriched gene sets between specimen types (Trivalent Influenza Vaccine, MSigDB Immunological Signatures) . . . 86
Figure 96 UpSet plots of enriched gene sets between specimen types (Trivalent Influenza Vaccine, MSigDB KEGG Pathways) . . . 87
Figure 97 UpSet plots of enriched gene sets between specimen types (Trivalent Influenza Vaccine, MSigDB Molecular Functions) . . . 87
Figure 98 UpSet plots of enriched gene sets between specimen types (Trivalent Influenza Vaccine, MSigDB Reactome Pathways) . . . 88
Figure 99 Venn diagrams of enriched Blood Transcription Modules between specimen types 1 of 2 (Trivalent Influenza Vaccine) . . . 89
Figure 100 Venn diagrams of enriched Blood Transcription Modules between specimen types 2 of 2 (Trivalent Influenza Vaccine) . . . 90
Figure 101 Venn diagrams of enriched MSigDB Biological Processes between specimen types 1 of 2 (Trivalent Influenza Vaccine) . . . 91
Figure 102 Venn diagrams of enriched MSigDB Biological Processes between specimen types 2 of 2 (Trivalent Influenza Vaccine) . . . 92
Figure 103 Venn diagrams of enriched MSigDB Cellular Components between specimen types 1 of 2 (Trivalent Influenza Vaccine) . . . 93
Figure 104 Venn diagrams of enriched MSigDB Cellular Components between specimen types 2 of 2 (Trivalent Influenza Vaccine) . . . 94
Figure 105 Venn diagrams of enriched MSigDB Immunological Signatures between specimen types 1 of 2 (Trivalent Influenza Vaccine) . . . 95

Figure 106 Venn diagrams of enriched MSigDB Immunological Signatures between specimen types 2 of 2 (Trivalent Influenza Vaccine) 96
Figure 107 Venn diagrams of enriched MSigDB KEGG Pathways between specimen types 1 of 2 (Trivalent Influenza Vaccine) 97
Figure 108 Venn diagrams of enriched MSigDB KEGG Pathways between specimen types 2 of 2 (Trivalent Influenza Vaccine) 98
Figure 109 Venn diagrams of enriched MSigDB Molecular Functions between specimen types 1 of 2 (Trivalent Influenza Vaccine) 99
Figure 110 Venn diagrams of enriched MSigDB Molecular Functions between specimen types 2 of 2 (Trivalent Influenza Vaccine) 100
Figure 111 Venn diagrams of enriched MSigDB Reactome Pathways between specimen types 1 of 2 (Trivalent Influenza Vaccine) 101
Figure 112 Venn diagrams of enriched MSigDB Reactome Pathways between specimen types 2 of 2 (Trivalent Influenza Vaccine) 102
Figure 113 Heatmap of enriched Blood Transcription Modules (RNA-Seq) 103
Figure 114 Heatmap of enriched MSigDB Biological Processes (RNA-Seq) 104
Figure 115 Heatmap of enriched MSigDB Cellular Components (RNA-Seq) 105
Figure 116 Heatmap of enriched MSigDB Immunological Signatures (RNA-Seq) 106
Figure 117 Heatmap of enriched MSigDB KEGG Pathways (RNA-Seq) 107
Figure 118 Heatmap of enriched MSigDB Molecular Functions (RNA-Seq) 108
Figure 119 Heatmap of enriched MSigDB Reactome Pathways (RNA-Seq) 109
Figure 120 Radar Plot of enriched Blood Transcription Modules (B Cells, Trivalent Influenza Vaccine) 110
Figure 121 Radar Plot of enriched Blood Transcription Modules (PBMC, Trivalent Influenza Vaccine) 110
Figure 122 Radar Plot of enriched MSigDB Biological Processes (B Cells, Trivalent Influenza Vaccine) 111
Figure 123 Radar Plot of enriched MSigDB Biological Processes (PBMC, Trivalent Influenza Vaccine) 111
Figure 124 Radar Plot of enriched MSigDB Cellular Components (B Cells, Trivalent Influenza Vaccine) 112
Figure 125 Radar Plot of enriched MSigDB Cellular Components (PBMC, Trivalent Influenza Vaccine) 112
Figure 126 Radar Plot of enriched MSigDB Immunological Signatures (B Cells, Trivalent Influenza Vaccine) 113
Figure 127 Radar Plot of enriched MSigDB Immunological Signatures (PBMC, Trivalent Influenza Vaccine) 113
Figure 128 Radar Plot of enriched MSigDB KEGG Pathways (B Cells, Trivalent Influenza Vaccine) 114
Figure 129 Radar Plot of enriched MSigDB KEGG Pathways (PBMC, Trivalent Influenza Vaccine) 114
Figure 130 Radar Plot of enriched MSigDB Molecular Functions (B Cells, Trivalent Influenza Vaccine) 115
Figure 131 Radar Plot of enriched MSigDB Molecular Functions (PBMC, Trivalent Influenza Vaccine) 115
Figure 132 Radar Plot of enriched MSigDB Reactome Pathways (B Cells, Trivalent Influenza Vaccine) 116
Figure 133 Radar Plot of enriched MSigDB Reactome Pathways (PBMC, Trivalent Influenza Vaccine) 116
Figure 134 Summary of preprocessing benchmarks. 117

List of Tables

Table 1 Number of excluded genes by gene type (RNA-Seq). . . . 118
Table 2 Summary human reference genome alignment statistics for study samples (All Specimen Types) . . . 118
Table 3 Summary human reference genome alignment statistics for study samples (B Cells) . . . 118
Table 4 Summary human reference genome alignment statistics for study samples (PBMC) . . . 118
Table 5 Outlying observations (RNA-Seq). . . . 119
Table 6 Number of genes that passed the low expression cut off (RNA-Seq). . . . 119
Table 7 Genes differentially expressed at Day 1 compared to pre-treatment (B Cells, Trivalent Influenza Vaccine). . 120
Table 8 Genes differentially expressed at Day 2 compared to pre-treatment (B Cells, Trivalent Influenza Vaccine). . 120
Table 9 Genes differentially expressed at Day 3 compared to pre-treatment (B Cells, Trivalent Influenza Vaccine). . 121
Table 10 Genes differentially expressed at Day 4 compared to pre-treatment (B Cells, Trivalent Influenza Vaccine). . 121
Table 11 Genes differentially expressed at Day 5 compared to pre-treatment (B Cells, Trivalent Influenza Vaccine). . 127
Table 12 Genes differentially expressed at Day 6 compared to pre-treatment (B Cells, Trivalent Influenza Vaccine). . 149
Table 13 Genes differentially expressed at Day 7 compared to pre-treatment (B Cells, Trivalent Influenza Vaccine). . 163
Table 14 Genes differentially expressed at Day 8 compared to pre-treatment (B Cells, Trivalent Influenza Vaccine). . 168
Table 15 Genes differentially expressed at Day 9 compared to pre-treatment (B Cells, Trivalent Influenza Vaccine). . 169
Table 16 Genes differentially expressed at Day 10 compared to pre-treatment (B Cells, Trivalent Influenza Vaccine). 169
Table 17 Genes differentially expressed at Day 1 compared to pre-treatment (PBMC, Trivalent Influenza Vaccine). . 176
Table 18 Genes differentially expressed at Day 2 compared to pre-treatment (PBMC, Trivalent Influenza Vaccine). . 180
Table 19 Genes differentially expressed at Day 3 compared to pre-treatment (PBMC, Trivalent Influenza Vaccine). . 181
Table 20 Genes differentially expressed at Day 4 compared to pre-treatment (PBMC, Trivalent Influenza Vaccine). . 181
Table 21 Genes differentially expressed at Day 5 compared to pre-treatment (PBMC, Trivalent Influenza Vaccine). . 183
Table 22 Genes differentially expressed at Day 6 compared to pre-treatment (PBMC, Trivalent Influenza Vaccine). . 187
Table 23 Genes differentially expressed at Day 7 compared to pre-treatment (PBMC, Trivalent Influenza Vaccine). . 190
Table 24 Genes differentially expressed at Day 8 compared to pre-treatment (PBMC, Trivalent Influenza Vaccine). . 193
Table 25 Genes differentially expressed at Day 9 compared to pre-treatment (PBMC, Trivalent Influenza Vaccine). . 194
Table 26 Genes differentially expressed at Day 10 compared to pre-treatment (PBMC, Trivalent Influenza Vaccine). 196
Table 27 Co-expressed gene clusters (B Cells, Day 1) . . . 197
Table 28 Co-expressed gene clusters (B Cells, Day 2) . . . 197
Table 29 Co-expressed gene clusters (B Cells, Day 3) . . . 198
Table 30 Co-expressed gene clusters (B Cells, Day 4) . . . 199
Table 31 Co-expressed gene clusters (B Cells, Day 5) . . . 204
Table 32 Co-expressed gene clusters (B Cells, Day 6) . . . 218
Table 33 Co-expressed gene clusters (B Cells, Day 7) . . . 231
Table 34 Co-expressed gene clusters (B Cells, Day 8) . . . 237
Table 35 Co-expressed gene clusters (B Cells, Day 9) . . . 238
Table 36 Co-expressed gene clusters (B Cells, Day 10) . . . 239
Table 37 Co-expressed gene clusters (B Cells, All post-treatment time points) . . . 244
Table 38 Co-expressed gene clusters (PBMC, Day 1) . . . 249
Table 39 Co-expressed gene clusters (PBMC, Day 2) . . . 252
Table 40 Co-expressed gene clusters (PBMC, Day 3) . . . 254
Table 41 Co-expressed gene clusters (PBMC, Day 4) . . . 254
Table 42 Co-expressed gene clusters (PBMC, Day 5) . . . 256
Table 43 Co-expressed gene clusters (PBMC, Day 6) . . . 260
Table 44 Co-expressed gene clusters (PBMC, Day 7) . . . 262
Table 45 Co-expressed gene clusters (PBMC, Day 8) . . . 266

Table 46 Co-expressed gene clusters (PBMC, Day 9) 266
Table 47 Co-expressed gene clusters (PBMC, Day 10) 268
Table 48 Co-expressed gene clusters (PBMC, All post-treatment time points) 272
Table 49 Overview of gene sets used for the enrichment analysis (RNA-Seq). 272
Table 50 Enriched MSigDB Biological Processes (B Cells, Trivalent Influenza Vaccine, Day 1) 273
Table 51 Enriched MSigDB Immunological Signatures (B Cells, Trivalent Influenza Vaccine, Day 1) 274
Table 52 Enriched MSigDB Reactome Pathways (B Cells, Trivalent Influenza Vaccine, Day 1) 274
Table 53 Enriched MSigDB Biological Processes (B Cells, Trivalent Influenza Vaccine, Day 3) 274
Table 54 Enriched MSigDB Cellular Components (B Cells, Trivalent Influenza Vaccine, Day 3) 275
Table 55 Enriched MSigDB Immunological Signatures (B Cells, Trivalent Influenza Vaccine, Day 3) 275
Table 56 Enriched MSigDB Molecular Functions (B Cells, Trivalent Influenza Vaccine, Day 3) 275
Table 57 Enriched MSigDB Biological Processes (B Cells, Trivalent Influenza Vaccine, Day 4) 276
Table 58 Enriched MSigDB Cellular Components (B Cells, Trivalent Influenza Vaccine, Day 4) 276
Table 59 Enriched MSigDB Molecular Functions (B Cells, Trivalent Influenza Vaccine, Day 4) 276
Table 60 Enriched Blood Transcription Modules (B Cells, Trivalent Influenza Vaccine, Day 5) 277
Table 61 Enriched MSigDB Biological Processes (B Cells, Trivalent Influenza Vaccine, Day 5) 278
Table 62 Enriched MSigDB Cellular Components (B Cells, Trivalent Influenza Vaccine, Day 5) 279
Table 63 Enriched MSigDB Immunological Signatures (B Cells, Trivalent Influenza Vaccine, Day 5) 280
Table 64 Enriched MSigDB KEGG Pathways (B Cells, Trivalent Influenza Vaccine, Day 5) 280
Table 65 Enriched MSigDB Molecular Functions (B Cells, Trivalent Influenza Vaccine, Day 5) 281
Table 66 Enriched MSigDB Reactome Pathways (B Cells, Trivalent Influenza Vaccine, Day 5) 282
Table 67 Enriched Blood Transcription Modules (B Cells, Trivalent Influenza Vaccine, Day 6) 282
Table 68 Enriched MSigDB Biological Processes (B Cells, Trivalent Influenza Vaccine, Day 6) 284
Table 69 Enriched MSigDB Cellular Components (B Cells, Trivalent Influenza Vaccine, Day 6) 285
Table 70 Enriched MSigDB Immunological Signatures (B Cells, Trivalent Influenza Vaccine, Day 6) 286
Table 71 Enriched MSigDB KEGG Pathways (B Cells, Trivalent Influenza Vaccine, Day 6) 286
Table 72 Enriched MSigDB Molecular Functions (B Cells, Trivalent Influenza Vaccine, Day 6) 287
Table 73 Enriched MSigDB Reactome Pathways (B Cells, Trivalent Influenza Vaccine, Day 6) 288
Table 74 Enriched Blood Transcription Modules (B Cells, Trivalent Influenza Vaccine, Day 7) 289
Table 75 Enriched MSigDB Biological Processes (B Cells, Trivalent Influenza Vaccine, Day 7) 290
Table 76 Enriched MSigDB Cellular Components (B Cells, Trivalent Influenza Vaccine, Day 7) 291
Table 77 Enriched MSigDB Immunological Signatures (B Cells, Trivalent Influenza Vaccine, Day 7) 292
Table 78 Enriched MSigDB KEGG Pathways (B Cells, Trivalent Influenza Vaccine, Day 7) 292
Table 79 Enriched MSigDB Molecular Functions (B Cells, Trivalent Influenza Vaccine, Day 7) 293
Table 80 Enriched MSigDB Reactome Pathways (B Cells, Trivalent Influenza Vaccine, Day 7) 293
Table 81 Enriched Blood Transcription Modules (B Cells, Trivalent Influenza Vaccine, Day 8) 293
Table 82 Enriched MSigDB Biological Processes (B Cells, Trivalent Influenza Vaccine, Day 8) 294
Table 83 Enriched MSigDB Cellular Components (B Cells, Trivalent Influenza Vaccine, Day 8) 294
Table 84 Enriched MSigDB Immunological Signatures (B Cells, Trivalent Influenza Vaccine, Day 8) 295
Table 85 Enriched MSigDB KEGG Pathways (B Cells, Trivalent Influenza Vaccine, Day 8) 296
Table 86 Enriched MSigDB Molecular Functions (B Cells, Trivalent Influenza Vaccine, Day 8) 296
Table 87 Enriched MSigDB Reactome Pathways (B Cells, Trivalent Influenza Vaccine, Day 8) 296
Table 88 Enriched MSigDB Biological Processes (B Cells, Trivalent Influenza Vaccine, Day 9) 297
Table 89 Enriched MSigDB Cellular Components (B Cells, Trivalent Influenza Vaccine, Day 9) 297
Table 90 Enriched MSigDB Molecular Functions (B Cells, Trivalent Influenza Vaccine, Day 9) 297
Table 91 Enriched Blood Transcription Modules (PBMC, Trivalent Influenza Vaccine, Day 1) 298
Table 92 Enriched MSigDB Biological Processes (PBMC, Trivalent Influenza Vaccine, Day 1) 299

Table 93	Enriched MSigDB Cellular Components (PBMC, Trivalent Influenza Vaccine, Day 1)	299
Table 94	Enriched MSigDB Immunological Signatures (PBMC, Trivalent Influenza Vaccine, Day 1)	301
Table 95	Enriched MSigDB KEGG Pathways (PBMC, Trivalent Influenza Vaccine, Day 1)	301
Table 96	Enriched MSigDB Molecular Functions (PBMC, Trivalent Influenza Vaccine, Day 1)	301
Table 97	Enriched MSigDB Reactome Pathways (PBMC, Trivalent Influenza Vaccine, Day 1)	301
Table 98	Enriched Blood Transcription Modules (PBMC, Trivalent Influenza Vaccine, Day 2)	302
Table 99	Enriched MSigDB Biological Processes (PBMC, Trivalent Influenza Vaccine, Day 2)	303
Table 100	Enriched MSigDB Cellular Components (PBMC, Trivalent Influenza Vaccine, Day 2)	303
Table 101	Enriched MSigDB Immunological Signatures (PBMC, Trivalent Influenza Vaccine, Day 2)	304
Table 102	Enriched MSigDB KEGG Pathways (PBMC, Trivalent Influenza Vaccine, Day 2)	305
Table 103	Enriched MSigDB Molecular Functions (PBMC, Trivalent Influenza Vaccine, Day 2)	305
Table 104	Enriched MSigDB Reactome Pathways (PBMC, Trivalent Influenza Vaccine, Day 2)	305
Table 105	Enriched Blood Transcription Modules (PBMC, Trivalent Influenza Vaccine, Day 3)	305
Table 106	Enriched MSigDB Biological Processes (PBMC, Trivalent Influenza Vaccine, Day 3)	307
Table 107	Enriched MSigDB Cellular Components (PBMC, Trivalent Influenza Vaccine, Day 3)	307
Table 108	Enriched MSigDB Immunological Signatures (PBMC, Trivalent Influenza Vaccine, Day 3)	308
Table 109	Enriched MSigDB KEGG Pathways (PBMC, Trivalent Influenza Vaccine, Day 3)	308
Table 110	Enriched Blood Transcription Modules (PBMC, Trivalent Influenza Vaccine, Day 5)	308
Table 111	Enriched MSigDB Biological Processes (PBMC, Trivalent Influenza Vaccine, Day 5)	309
Table 112	Enriched MSigDB Cellular Components (PBMC, Trivalent Influenza Vaccine, Day 5)	309
Table 113	Enriched MSigDB Immunological Signatures (PBMC, Trivalent Influenza Vaccine, Day 5)	310
Table 114	Enriched MSigDB Molecular Functions (PBMC, Trivalent Influenza Vaccine, Day 5)	311
Table 115	Enriched Blood Transcription Modules (PBMC, Trivalent Influenza Vaccine, Day 6)	311
Table 116	Enriched MSigDB Cellular Components (PBMC, Trivalent Influenza Vaccine, Day 6)	311
Table 117	Enriched MSigDB Immunological Signatures (PBMC, Trivalent Influenza Vaccine, Day 6)	312
Table 118	Enriched MSigDB Molecular Functions (PBMC, Trivalent Influenza Vaccine, Day 6)	312
Table 119	Enriched Blood Transcription Modules (PBMC, Trivalent Influenza Vaccine, Day 7)	313
Table 120	Enriched MSigDB Immunological Signatures (PBMC, Trivalent Influenza Vaccine, Day 7)	313
Table 121	Enriched Blood Transcription Modules (PBMC, Trivalent Influenza Vaccine, Day 8)	314
Table 122	Enriched MSigDB Biological Processes (PBMC, Trivalent Influenza Vaccine, Day 8)	315
Table 123	Enriched MSigDB Immunological Signatures (PBMC, Trivalent Influenza Vaccine, Day 8)	316
Table 124	Enriched MSigDB KEGG Pathways (PBMC, Trivalent Influenza Vaccine, Day 8)	316
Table 125	Enriched MSigDB Reactome Pathways (PBMC, Trivalent Influenza Vaccine, Day 8)	316
Table 126	Enriched MSigDB Biological Processes (PBMC, Trivalent Influenza Vaccine, Day 9)	317
Table 127	Enriched MSigDB Cellular Components (PBMC, Trivalent Influenza Vaccine, Day 9)	317
Table 128	Enriched MSigDB Molecular Functions (PBMC, Trivalent Influenza Vaccine, Day 9)	317
Table 129	Enriched Blood Transcription Modules (PBMC, Trivalent Influenza Vaccine, Day 10)	317
Table 130	Enriched MSigDB Biological Processes (PBMC, Trivalent Influenza Vaccine, Day 10)	318
Table 131	Enriched MSigDB Cellular Components (PBMC, Trivalent Influenza Vaccine, Day 10)	318
Table 132	Enriched MSigDB Immunological Signatures (PBMC, Trivalent Influenza Vaccine, Day 10)	319
Table 133	Enriched MSigDB Molecular Functions (PBMC, Trivalent Influenza Vaccine, Day 10)	319
Table 134	List of R packages and versions used for the analyses presented in this report.	320

1 Synopsis

The RNA experiment for this study comprises peripheral blood mononuclear cells (PBMCs) and B-cell samples from 5 subjects collected prior to Trivalent Influenza Vaccine (TIV) vaccination (Day 0) and at 10 time points post TIV vaccination (Days 1-10). Overall, the RNA-Seq dataset comprised 110 samples (https://www.nature.com/articles/srep02327, GEO: GSE45764).

2 Methods

2.1 RNA-Seq experiment

RNA was extracted with the Qiagen RNeasy micro kit. Concentrations were determined by UV spectrophotometry (Nanodrop) and integrity of ribosomal RNA was confirmed with the Agilent Bioanalyzer. Barcoded sequencing libraries were prepared with Illumina TruSeq RNA kits as recommended by Illumina, using 100 ng total RNA as input. All samples from an individual subject were sequenced in the same run using an Illumina Genome Analyzer IIx (22 samples per 8 lanes). CASAVA programs (Illumina, version 1.7) were used to demultiplex samples according to barcode.

2.2 RNA-Seq data preprocessing and data qc

The human reference genome assembly, gene models, and associated gene annotation information in the form of a Gene Transfer Format (GTF) were obtained from the ENSEMBL database (Version 87). The genomic reference was built by merging all human chromosomes. Sequence reads were aligned to the reference transcriptome/genome using the *STAR* splice-aware read aligner (Version 2.5.2a). Ensembl gene models were used to guide the alignment process. For each sample, the quality of reference alignments was evaluated using the *RSeQC* software (Version 2.6.4). Quality measures were summarized in tabular form, univariate boxplots, and multivariate starplots.

Gene expression quantification was carried out on the gene level using the featureCounts function as implemented in the *Subread* software (Version 1.5.3). Reads that overlapped with multiple genes or mapped to multiple genomic locations on the reference genome were excluded. Systematic sample differences in sequencing coverage were corrected for by calculating scaling factors for each sample using the trimmed mean of M-values (TMM) method as implemented in the *edgeR* R package (Version 3.18.1). Post- normalization, Mt-rRNA, Mt-tRNA, Mt-tRNA-pseudogene, tRNA, rRNA, rRNA-pseudogene, tRNA-pseudogene genes based on ENSEMBL GTF annotations were excluded from the final read count results (**Table 1**). TMM normalization was executed across all samples to evaluate global gene expression patterns, systematic effects, and outliers. Following outlier removal, TMM-normalization was rerun separately for each specimen type. The resulting normalized data was used for all downstream specimen type-specific analyses.

For data visualizations and multivariate analyses, TMM-normalized moderated log_2 counts per million (LCPM) were computed using the *edgeR* R package. To avoid taking the log_2 of zero values, a TMM-scaled count of 0.5 was added to each gene. Subject-specific log_2 fold changes from pre-treatment were calculated for each subject and post-treatment time point by subtracting the pre-treatment LCPM value from the respective post-treatment LCPM value.

Genes with maximum expression levels across all samples per specimen type that did not exceed the specified cut off of 3 LCPM were considered to be lowly expressed and were excluded from downstream analysis. To guide the cut-off selection, reverse cumulative distribution functions summarizing the percentage of genes whose maximum LCPM

exceeded a certain LCPM cut off were plotted for each specimen type (**Figure 13**). Filtered TMM-normalized LCPM were standardized (z-score: mean=0, variance=1) and LCPM distributions across samples were inspected for outliers and systematic effects using principal component analysis, multidimensional scaling, and hierarchical clustering analysis. Identified outliers were were excluded from downstream analysis.

2.2.1 Identification of differentially expressed genes

Negative binomial generalized linear models as implemented in the edgeR software were applied to identify differentially expressed (DE) genes after exclusion of outlying samples and lowly expressed genes. TMM-adjusted total read counts per sample were included in the models as an offset to account for systematic sample differences. For each specimen type, DE gene analysis was carried per post-treatment time point (Day 1, Day 2, Day 3, Day 4, Day 5, Day 6, Day 7, Day 8, Day 9, Day 10) in relation to pre-treatment. Each model included coefficients to estimate subject and pre- vs. post-treatment effects, i.e. a subject and a time factor. The subject effect for estimating subject-specific pre-treatment levels was added to account for paired samples from the same subject. The statistical significance of the post- vs. pre-treatment effect was evaluated using a likelihood ratio test. To control for testing multiple genes, the false-discovery rate (FDR) based on the Benjamini-Hochberg procedure as implemented in the *p.adjust* R function was applied for each model. Genes with a pre-treatment fold change of $\geq$ 1.5 and FDR-adjusted p-value $<$ 0.05 were considered to be DE genes. DE gene results were tabulated and treatment effects in terms of overall log_2 fold changes and FDR-adjusted p-values were summarized for each specimen type and post-treatment time point using MA plots and Volcano plots. Overlap in DE genes between post-treatment time points and/or specimen types was assessed using Venn diagrams and/or UpSet plots.

2.2.2 Determination of co-expressed gene clusters

Unsupervised multiscale bootstrap resampling as implemented in the *pvclust* R package (Version 2.0-0) was carried out for each specimen type and post-treatment time point to identify robust clusters of co-expressed genes with correlated log_2 fold change responses. Genes identified as DE for any post-treatment time point were included as part of this analysis. Bootstrap resampling was based on uncentered pearson correlation distances between log_2 fold change responses in combination with the complete linkage clustering algorithm using varying dataset sizes. For each dataset size bin, 1000 bootstrap samples were obtained, and *pvclust* bootstrap probabilities and unbiased p-values were calculated. An unbiased p-value cut-off of $\geq$ 0.95 was applied to determine significant clusters. The maximum distance to form a significant cluster was set to 0.5. Clusters that were formed at a larger distance were excluded. Gene cluster dendrograms were visualized and cluster information was tabulated. For gene clusters with correlated responses across all post-treatment time points (Day 1, Day 2, Day 3, Day 4, Day 5, Day 6, Day 7, Day 8, Day 9, Day 10), pre-treatment fold change time trends across all time points were provided.

2.2.3 Pathway enrichment analysis

Pathway enrichment analysis was carried out separately for specimen type and post-treatment time point using gene sets listed in **Table 49**. The enrichment analysis was conducted using the *GOseq* software (Version 1.28.0) which adjusts for RNA-Seq gene length bias. Gene length information was obtained from the Ensembl database using the *biomaRt* R package (Version 2.32.1, Ensembl Version 87). Gene length was defined as the length of the longest transcript per gene including untranslated regions and coding sequence. *GOseq* probability weighting functions were estimated for each specimen type and post-treatment time point based on the respective DE gene results. Null distributions were estimated using *GOseq's* random sampling option (10^4 randomizations were applied). To account for testing multiple gene sets,

gene sets with a FDR-adjusted p-value < 0.1 were considered to be significantly enriched. In addition, the Jaccard index (to assess agreement between DE genes and gene sets) and enrichment score (-1 x log_{10}(FDR-adjusted p-value)) for each gene set was calculated. Pathway enrichment trends over time were visualized using heatmap and radar plots. Overlap in enriched pathways between post-treatment time points and/or specimen types was assessed using using Venn diagrams and/or UpSet plots.

2.3 Software

Data was analyzed using the *R statistical programming language* (version 3.4.1 (2017-06-30)) and *R Bioconductor* packages. This report was generated using the *knitr* R package (Version 1.17) and *LaTeX* typesetting software (Version TeX Live 2012/Debian). The operating used was *Ubuntu* (Version 16.04.2 LTS). Additional software along with version information is listed in the respective method sections and in **Table 134**.

3 Results

3.1 Figures

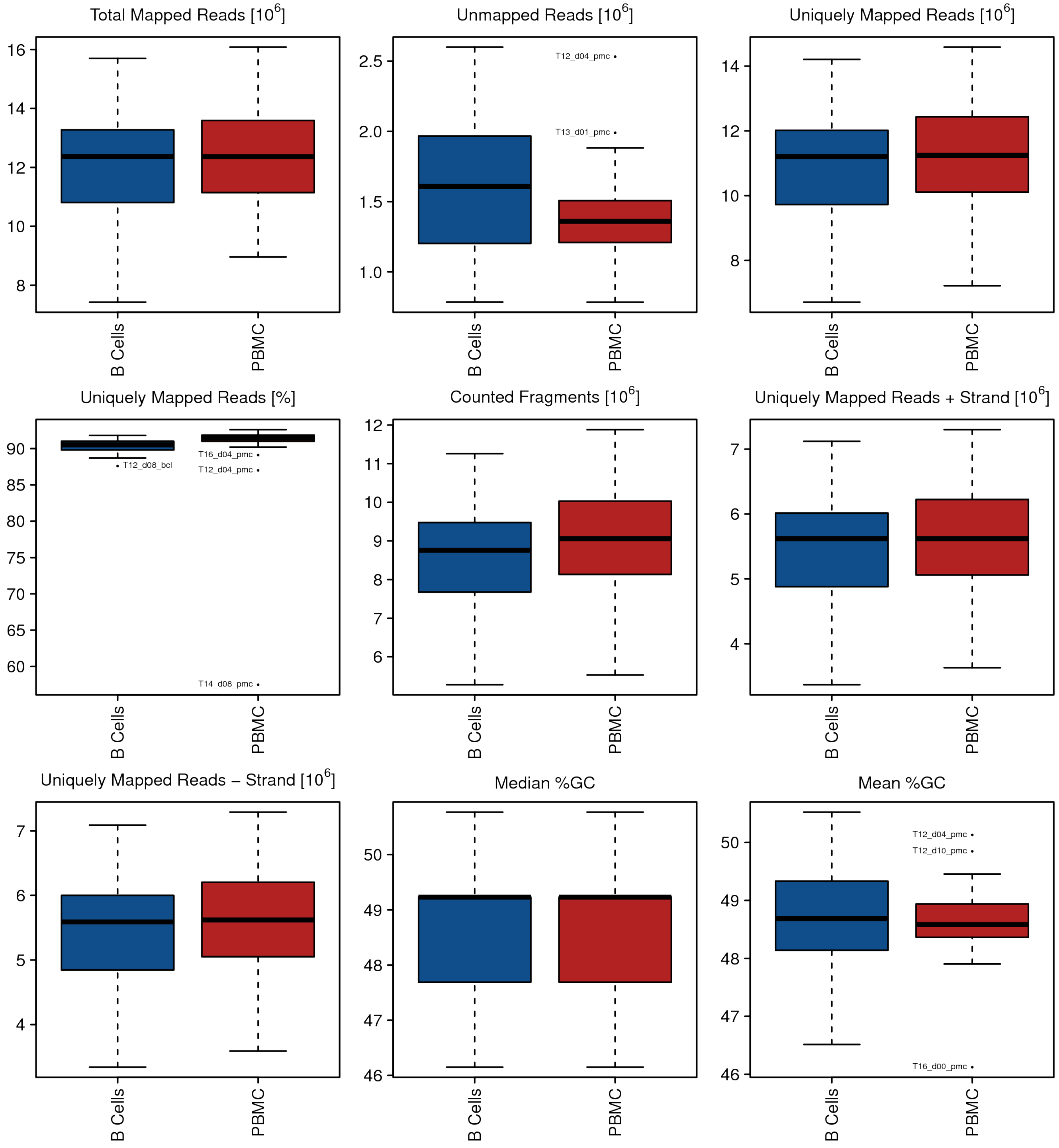

Figure 1: Boxplots of human reference genome alignment genome statistics (All specimen types).

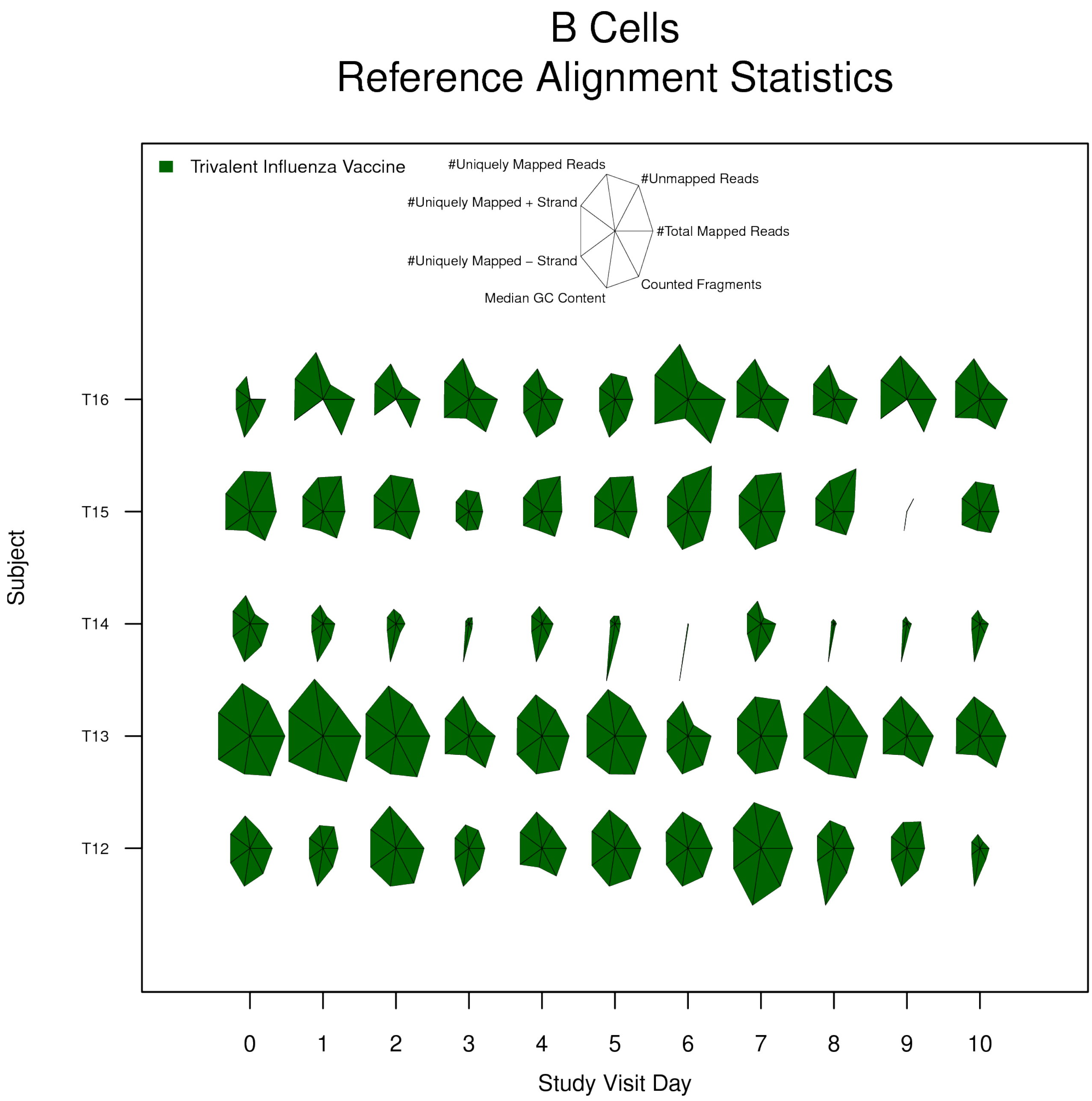

Figure 2: Starplots of human reference genome alignment statistics (B Cells).

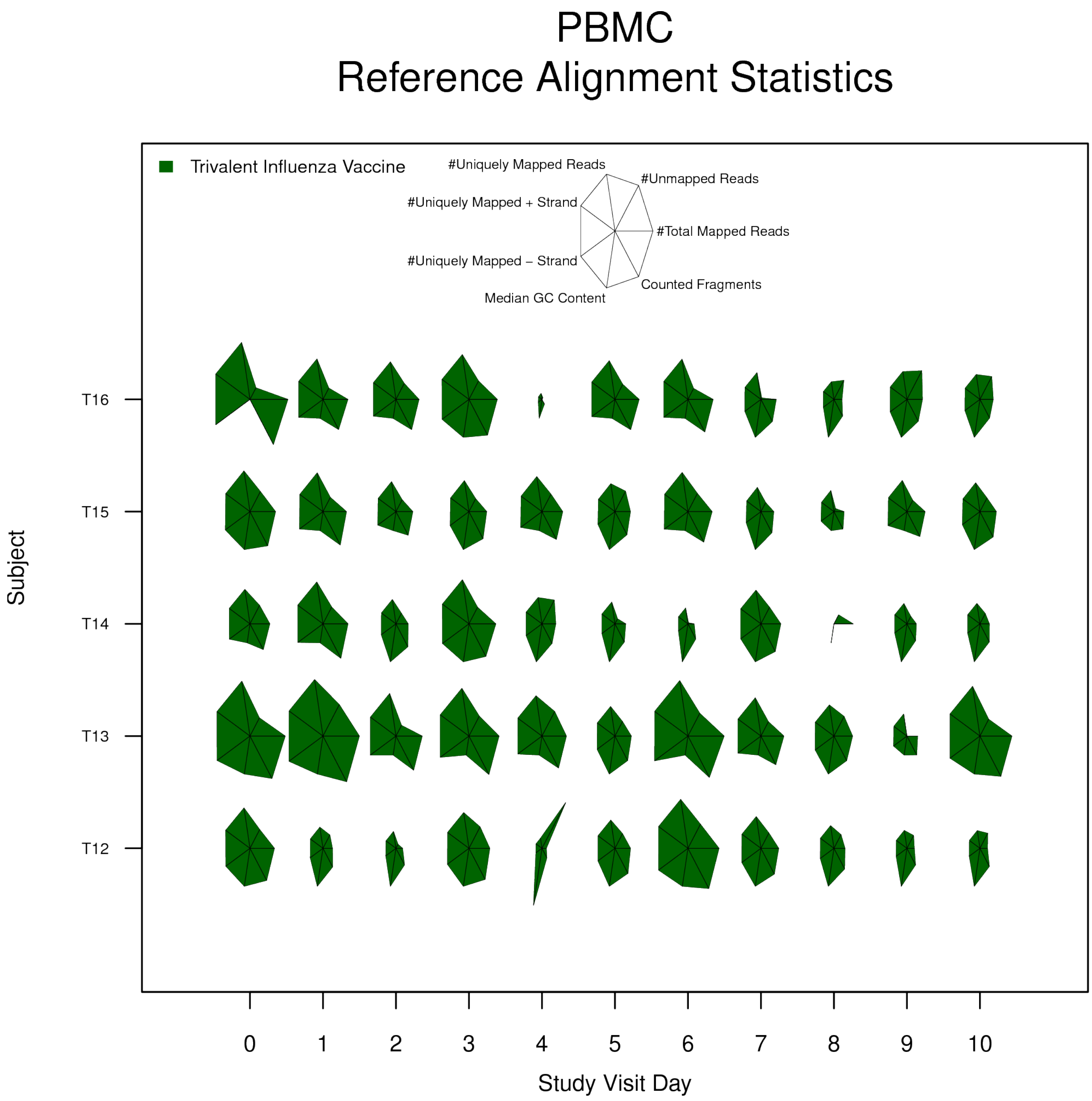

Figure 3: Starplots of human reference genome alignment statistics (PBMC).

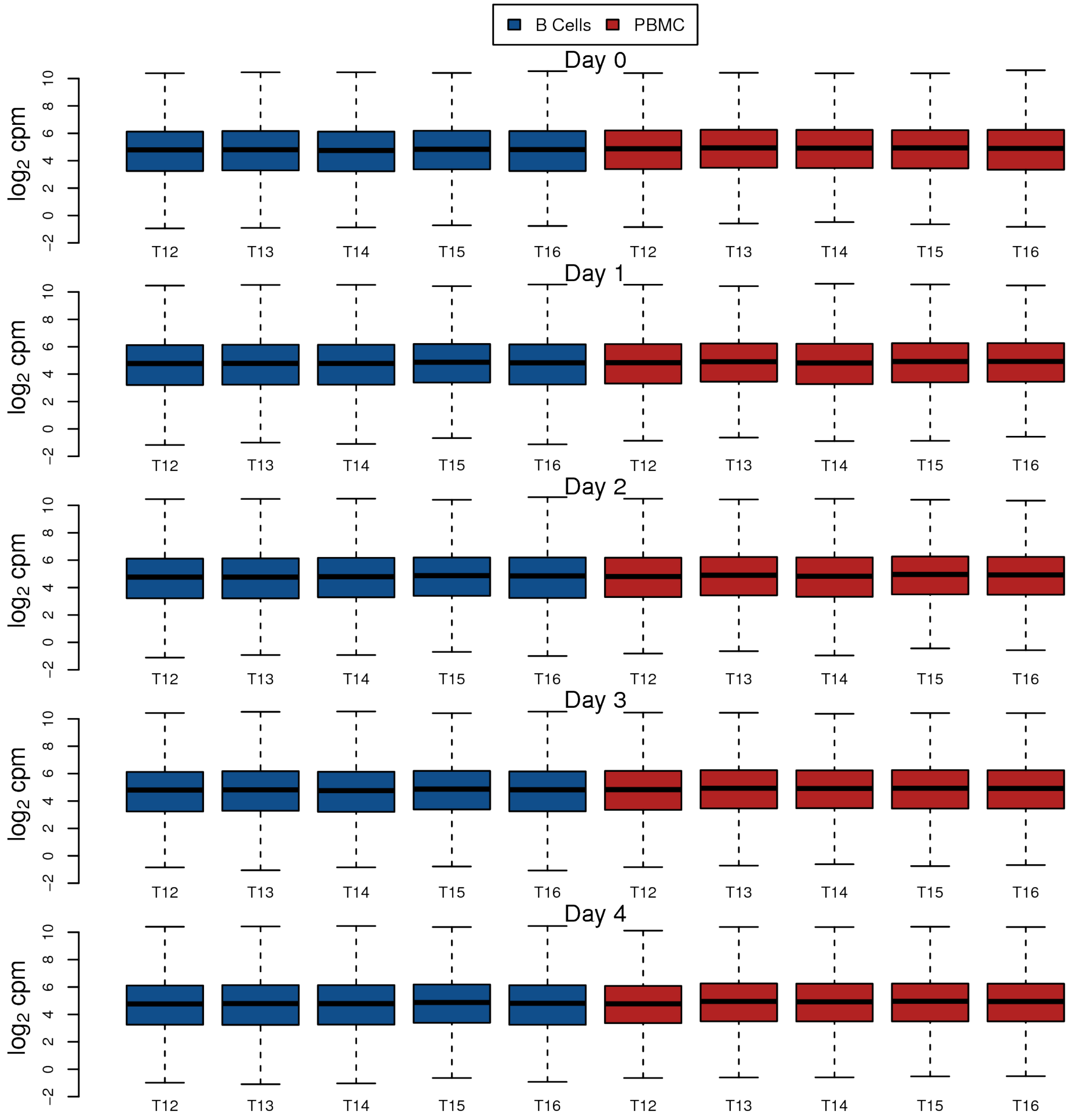

Figure 4: Boxplots of log_2 counts per million before TMM normalization 1 of 3 (All specimen types).

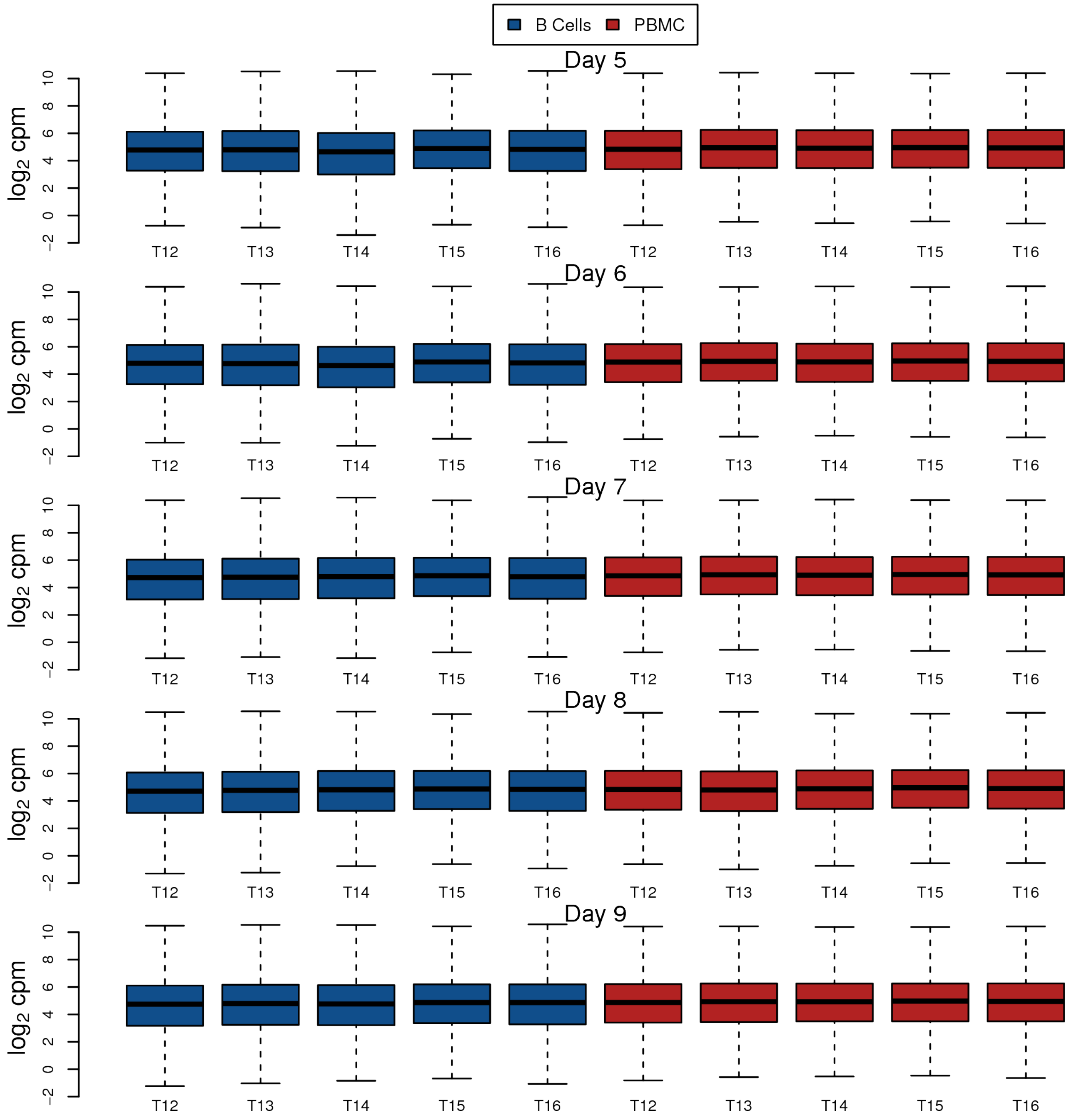

Figure 5: Boxplots of log_2 counts per million before TMM normalization 2 of 3 (All specimen types).

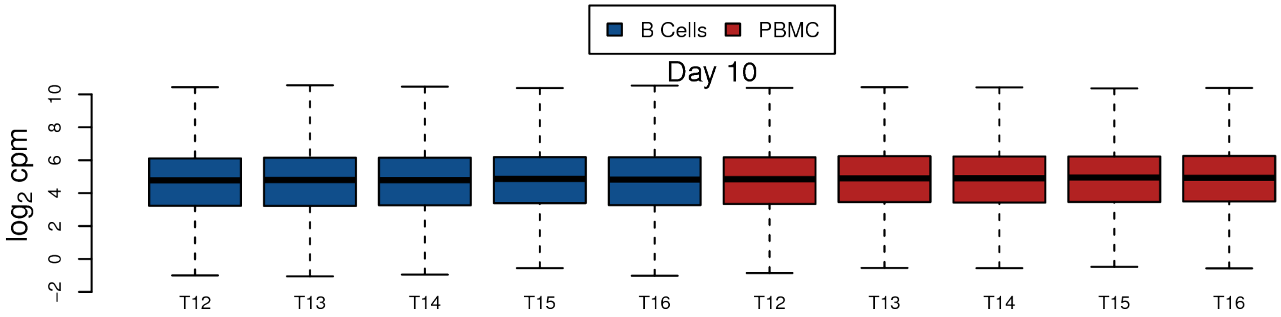

Figure 6: Boxplots of log_2 counts per million before TMM normalization 3 of 3 (All specimen types).

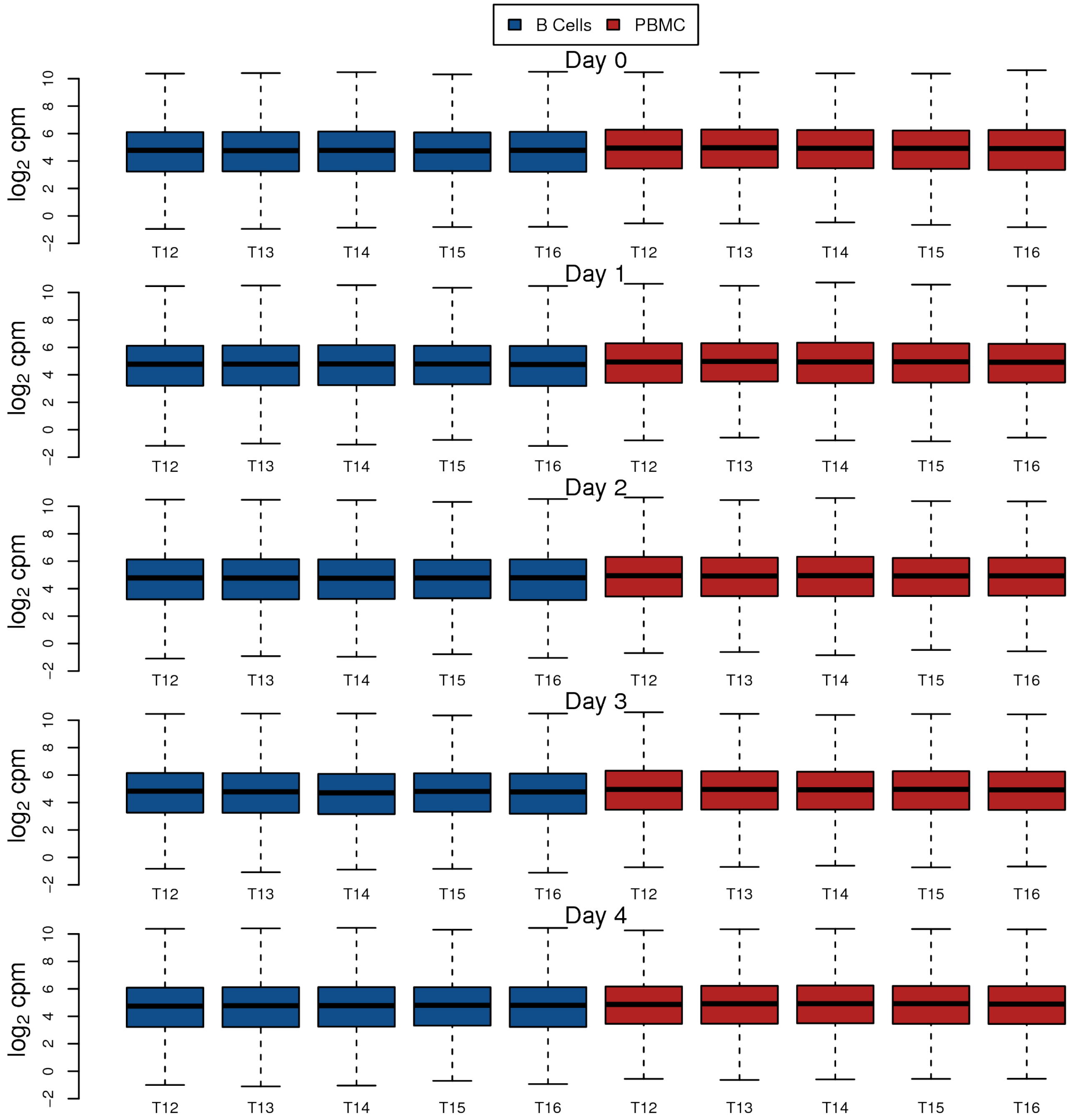

Figure 7: Boxplots of log_2 counts per million after TMM normalization 1 of 3 (All specimen types).

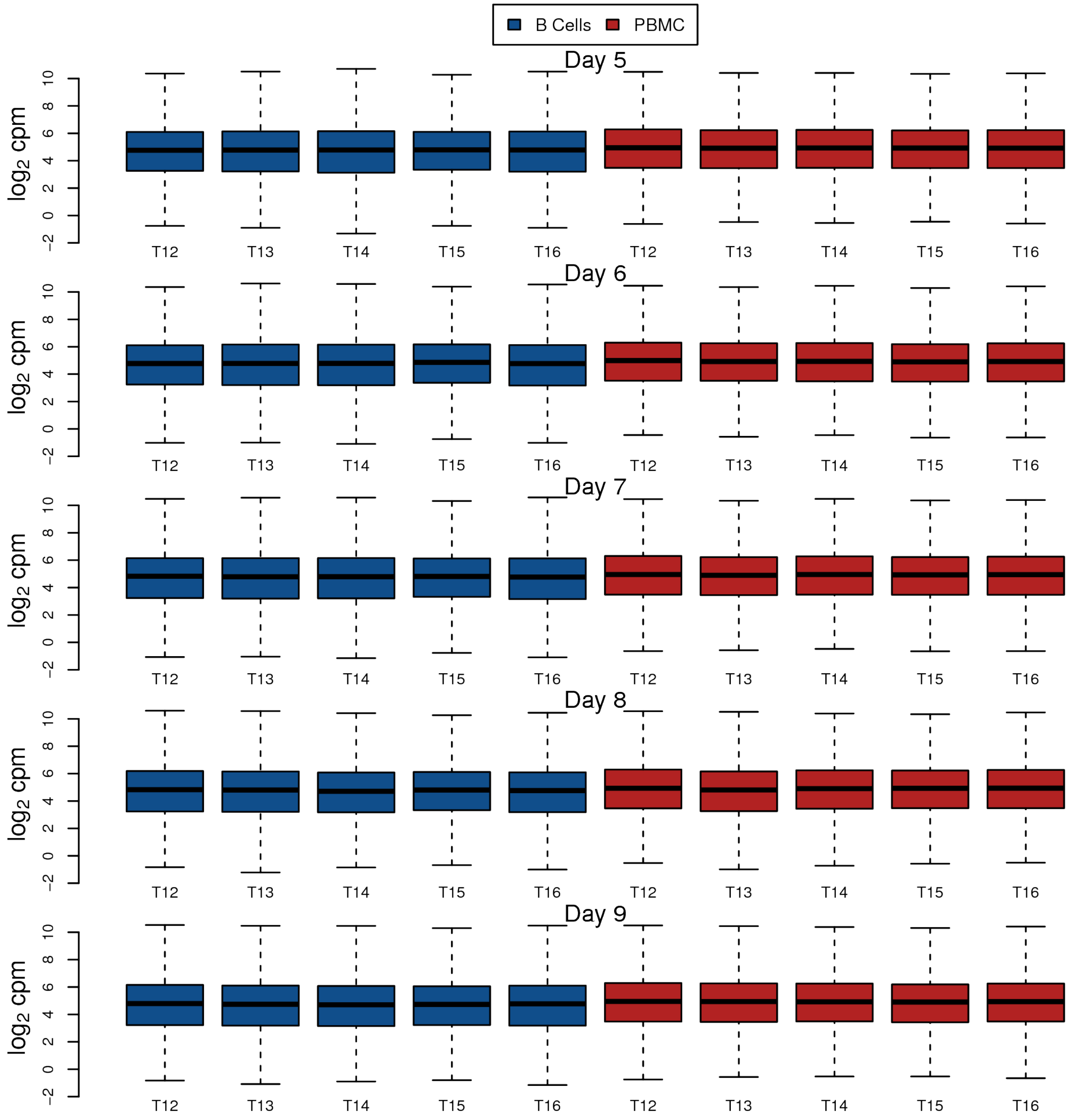

Figure 8: Boxplots of log_2 counts per million after TMM normalization 2 of 3 (All specimen types).

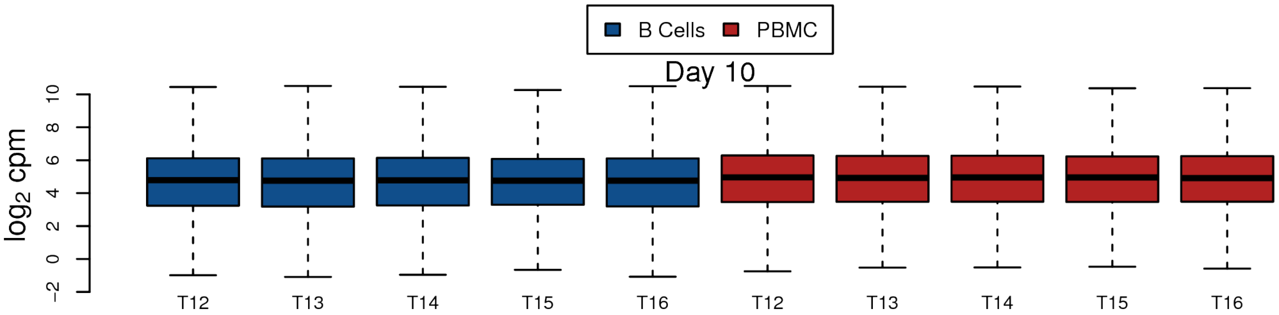

Figure 9: Boxplots of log_2 counts per million after TMM normalization 3 of 3 (All specimen types).

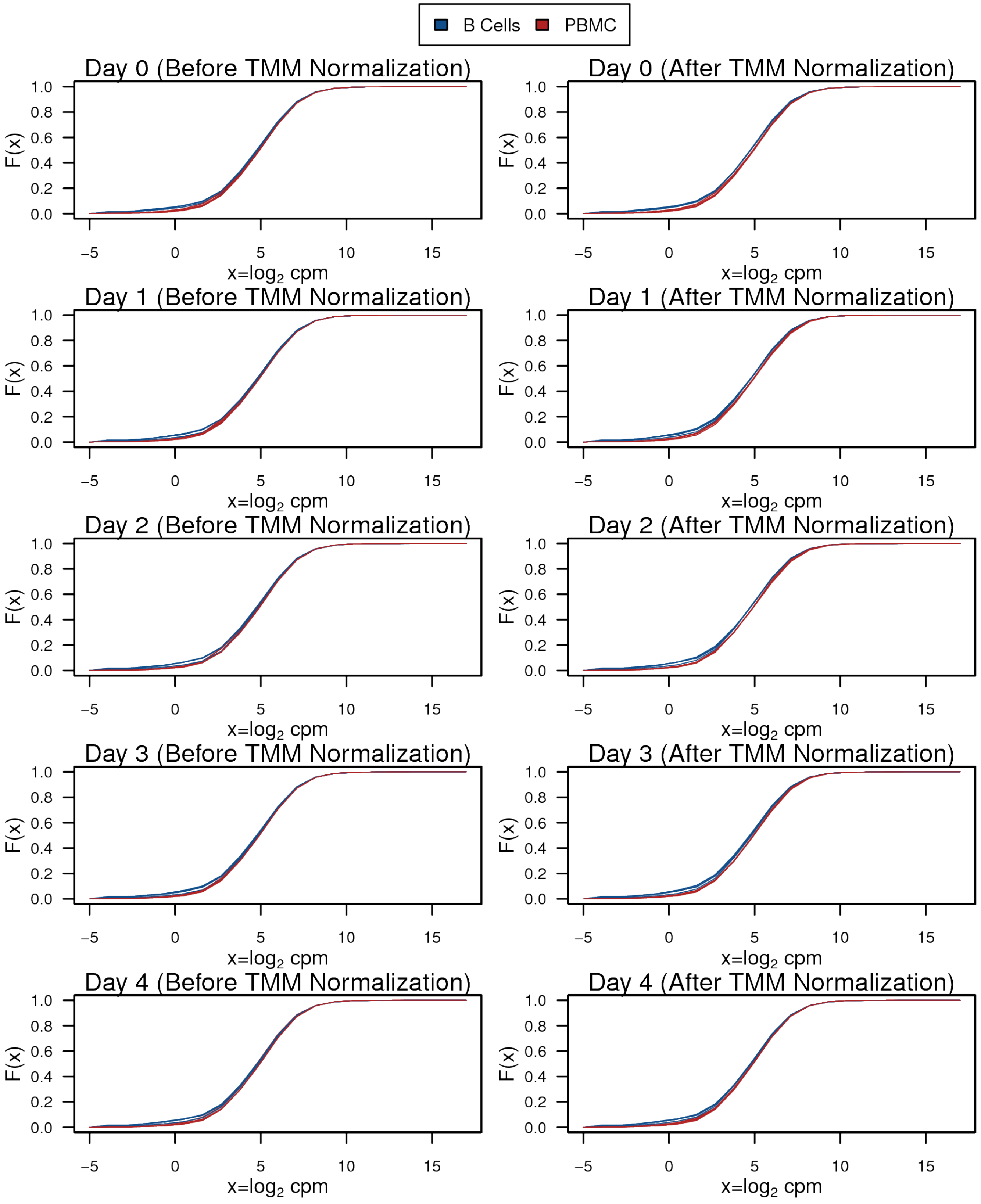

Figure 10: Empirical cumulative distribution function plots of log_2 counts per million before and after TMM normalization 1 of 3 (All specimen types).

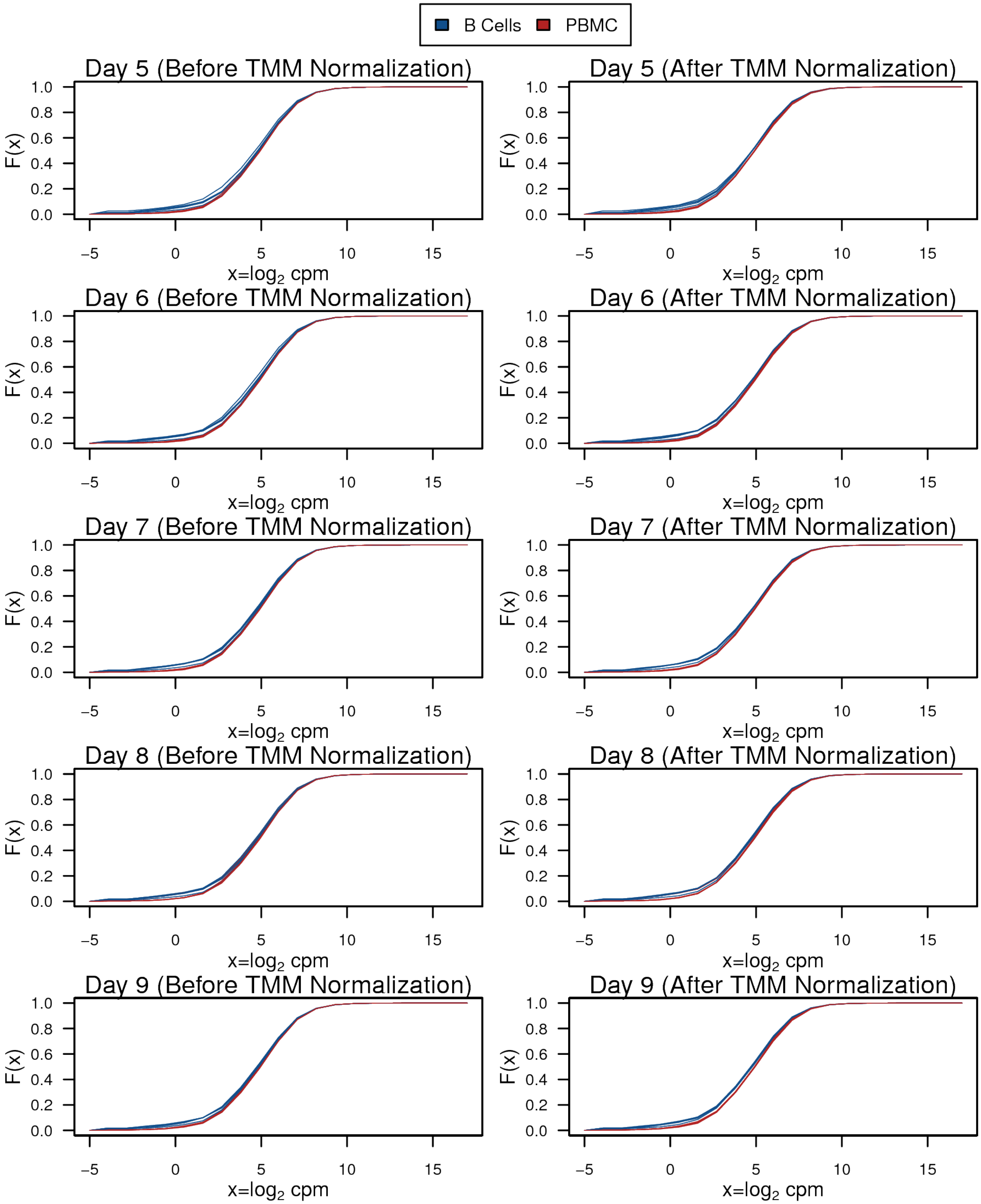

Figure 11: Empirical cumulative distribution function plots of log_2 counts per million before and after TMM normalization 2 of 3 (All specimen types).

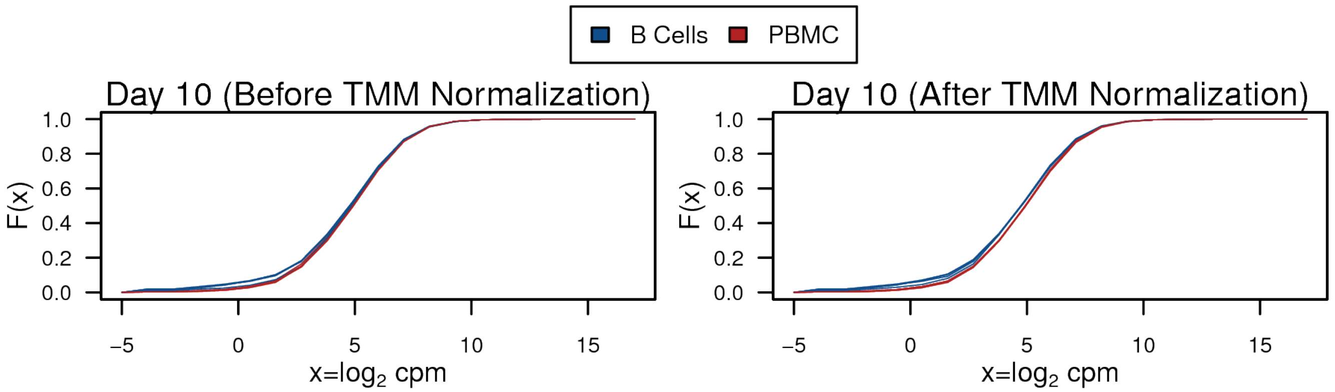

Figure 12: Empirical cumulative distribution function plots of log_2 counts per million before and after TMM normalization 3 of 3 (All specimen types).

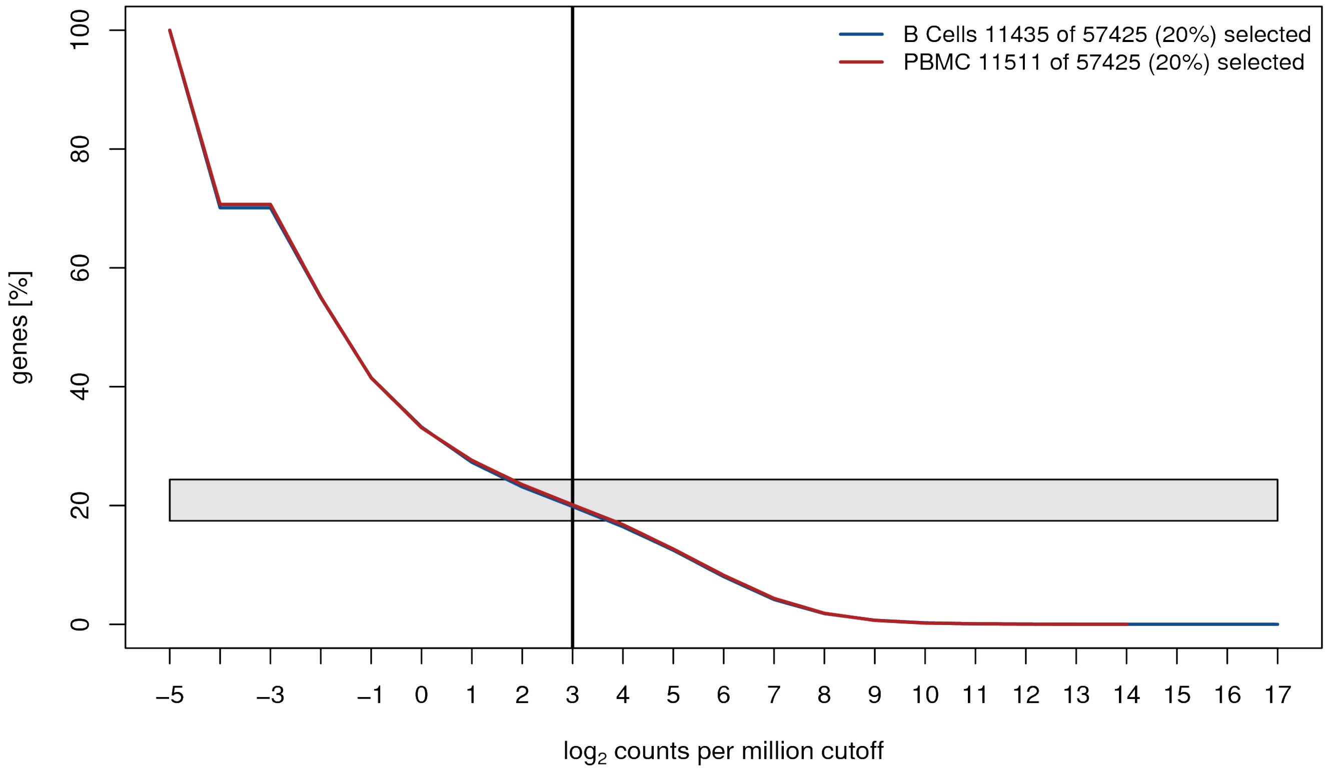

Figure 13: Reverse empirical cumulative distribution function plots of maximum gene expression levels across study samples (All specimen types). The x-axis represents the log_2 count per million cut off for identifying lowly expressed genes. The y-axis shows the percentage of all genes whose maximum gene expression level across all study samples exceeds the respective cut off. The grey box indicates the target range of genes to be selected (between 10000 and 14000 genes). The black vertical lines represent the specified cut off (3 log_2 counts per million).

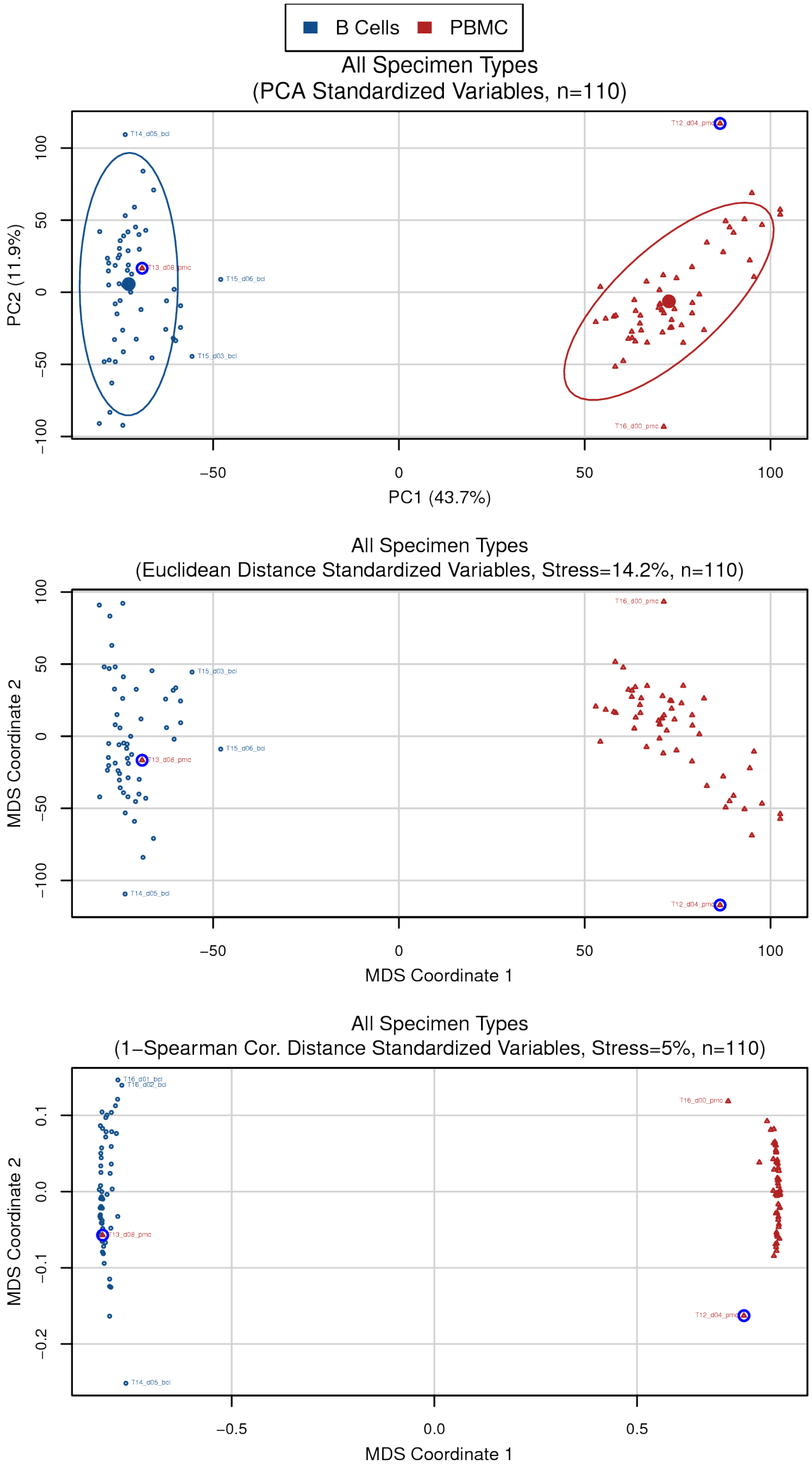

Figure 14: PCA and non-metric multidimensional scaling biplots (All Specimen Types). PCA biplots with bivariate 95% confidence ellipses for standardized variables are shown at the top. Non-metric MDS results for standardized variables and pairwise differences based on Euclidean distance are shown in the middle. Non-metric MDS results for original variables based on 1-Spearman correlation distance are shown at the bottom. Labels for the four most outlying samples per laboratory based on maximum Mahalanobis distance are shown. Strong outliers are highlighted in blue.

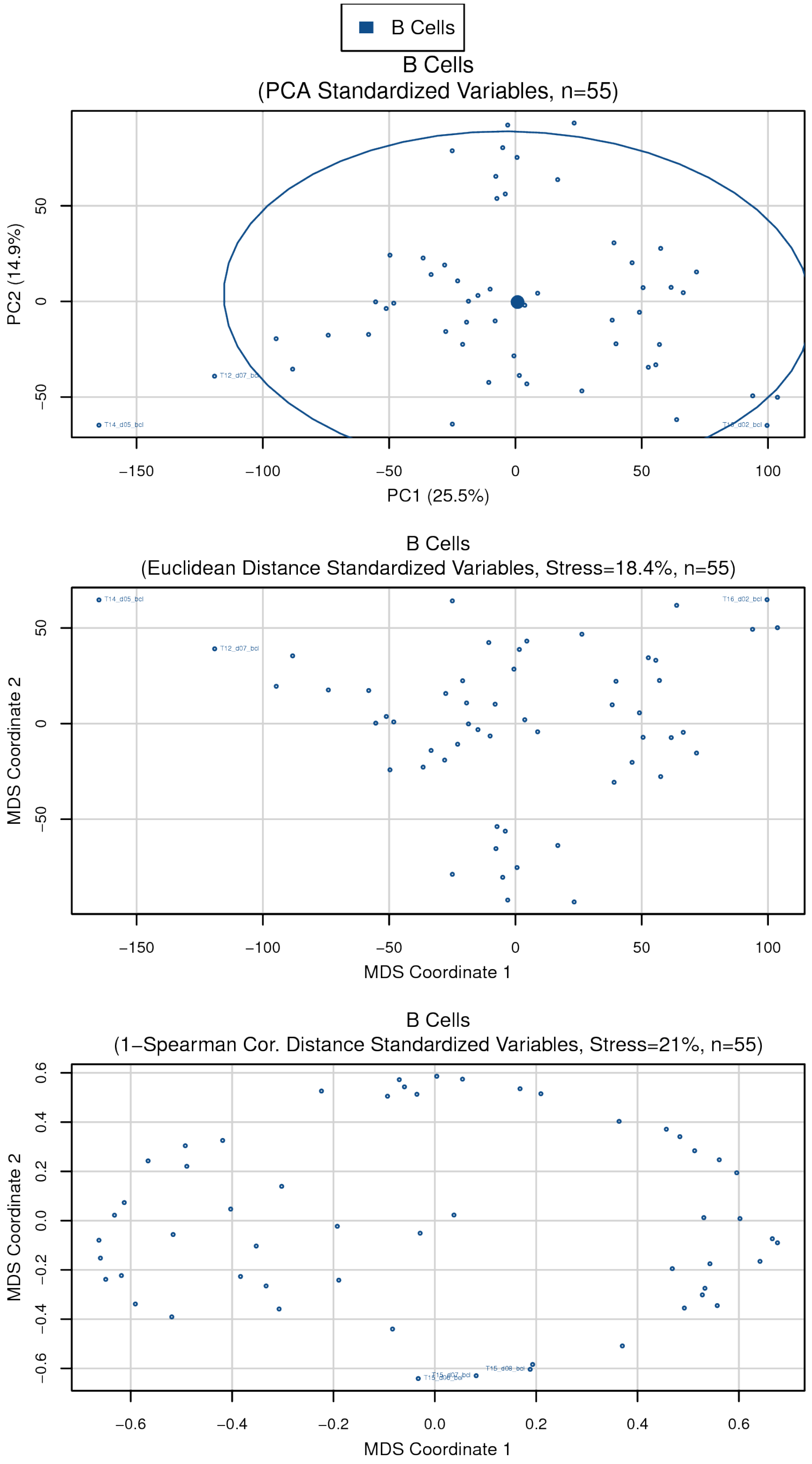

Figure 15: PCA and non-metric multidimensional scaling biplots (B Cells). PCA biplots with bivariate 95% confidence ellipses for standardized variables are shown at the top. Non-metric MDS results for standardized variables and pairwise differences based on Euclidean distance are shown in the middle. Non-metric MDS results for original variables based on 1-Spearman correlation distance are shown at the bottom. Labels for the four most outlying samples per laboratory based on maximum Mahalanobis distance are shown. Strong outliers are highlighted in blue.

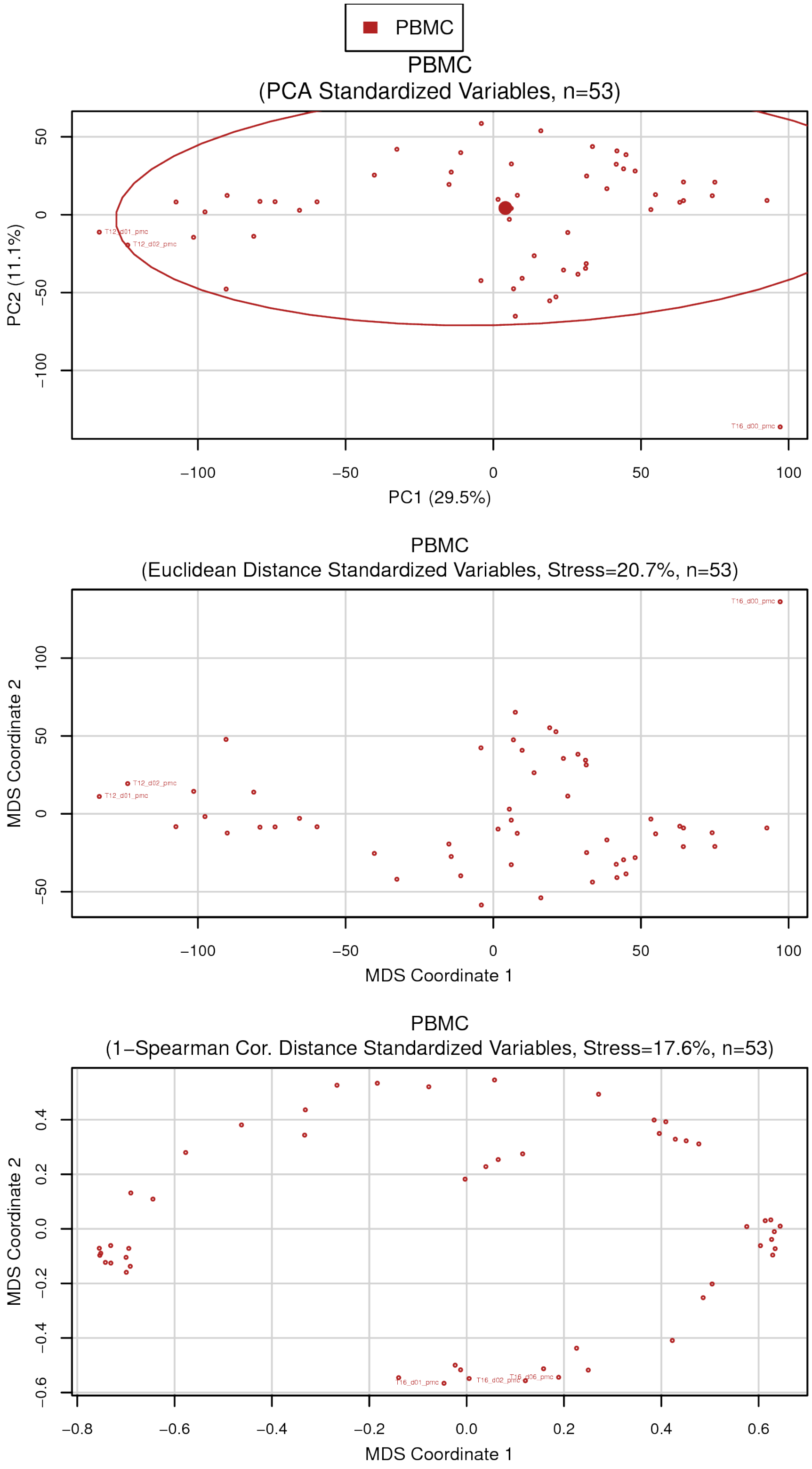

Figure 16: PCA and non-metric multidimensional scaling biplots (PBMC). PCA biplots with bivariate 95% confidence ellipses for standardized variables are shown at the top. Non-metric MDS results for standardized variables and pairwise differences based on Euclidean distance are shown in the middle. Non-metric MDS results for original variables based on 1-Spearman correlation distance are shown at the bottom. Labels for the four most outlying samples per laboratory based on maximum Mahalanobis distance are shown. Strong outliers are highlighted in blue.

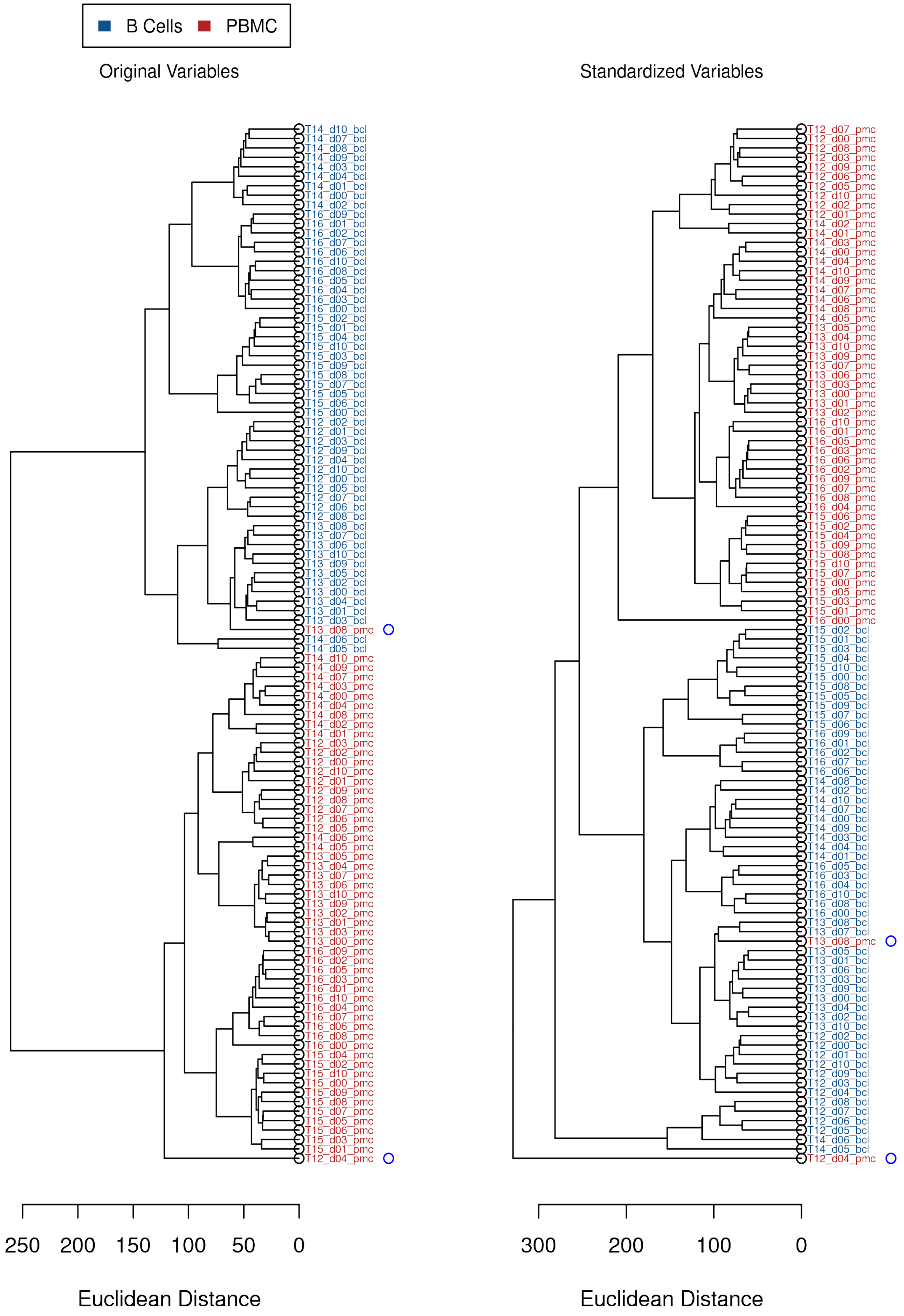

Figure 17: Hierarchical clustering plots (All Specimen Types). Euclidean distances hierarchically clustered using the complete linkage clustering algorithm. Outliers are marked by a blue circle next to the sample ID.

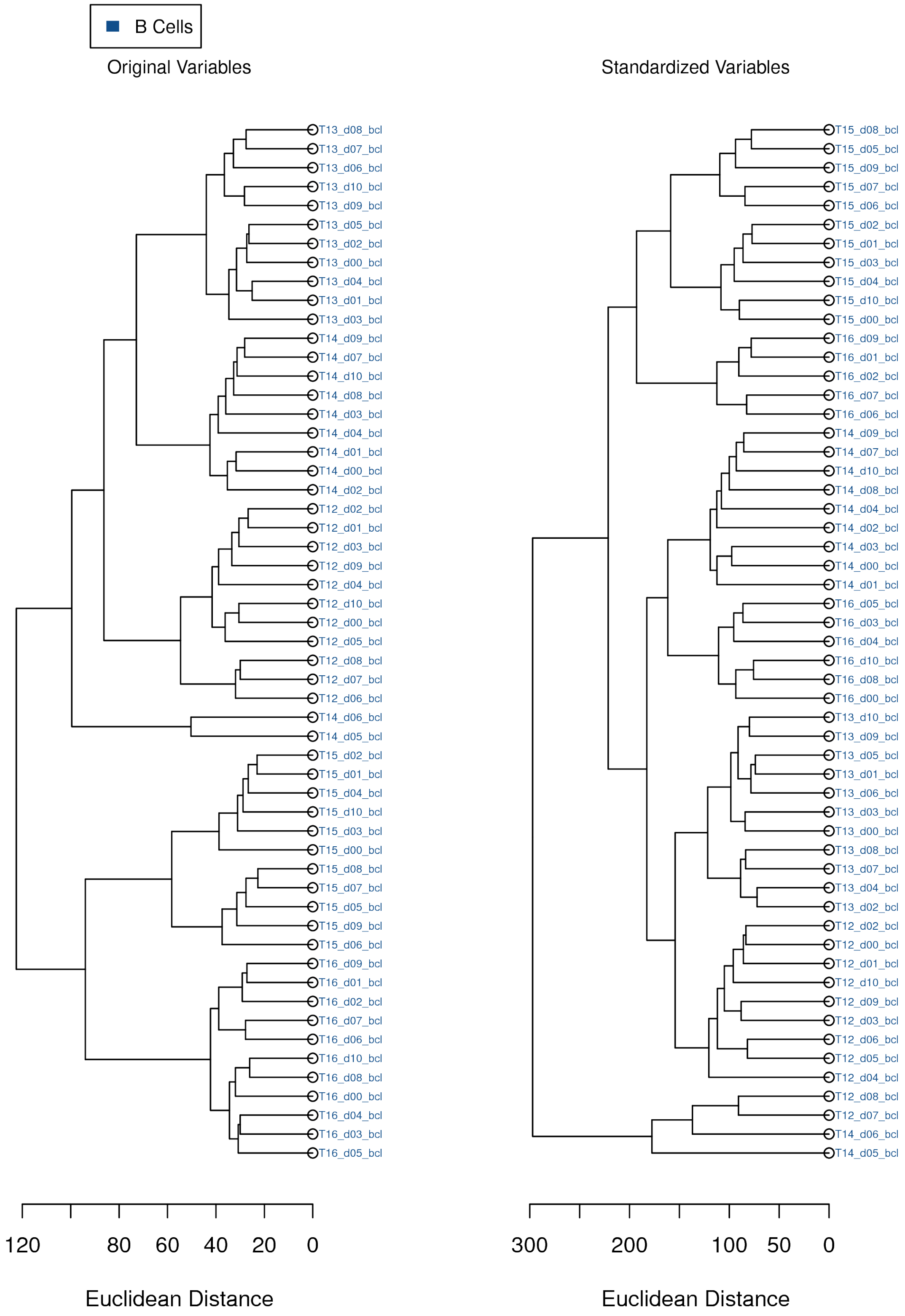

Figure 18: Hierarchical clustering plots (B Cells). Euclidean distances hierarchically clustered using the complete linkage clustering algorithm. Outliers are marked by a blue circle next to the sample ID.

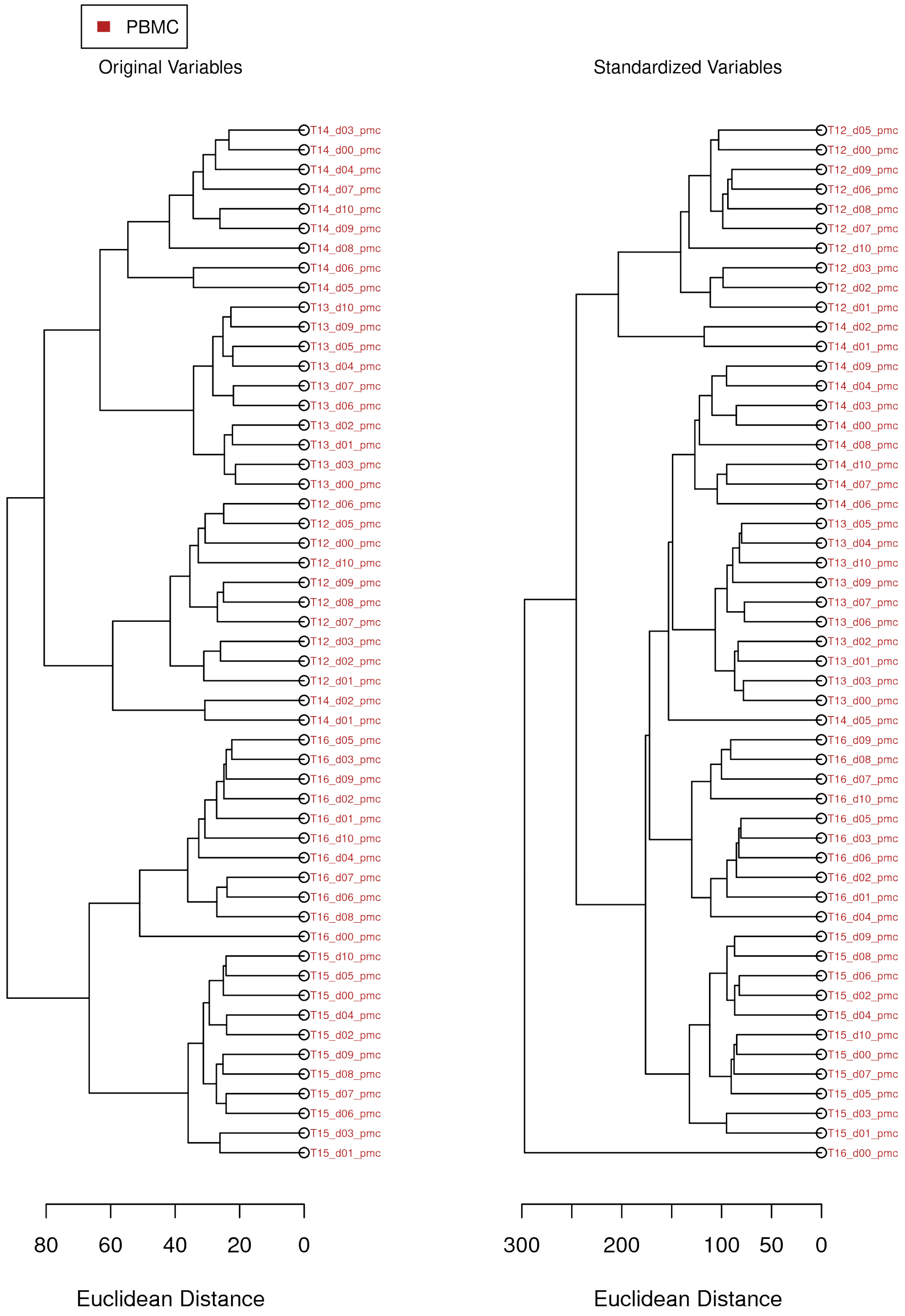

Figure 19: Hierarchical clustering plots (PBMC). Euclidean distances hierarchically clustered using the complete linkage clustering algorithm. Outliers are marked by a blue circle next to the sample ID.

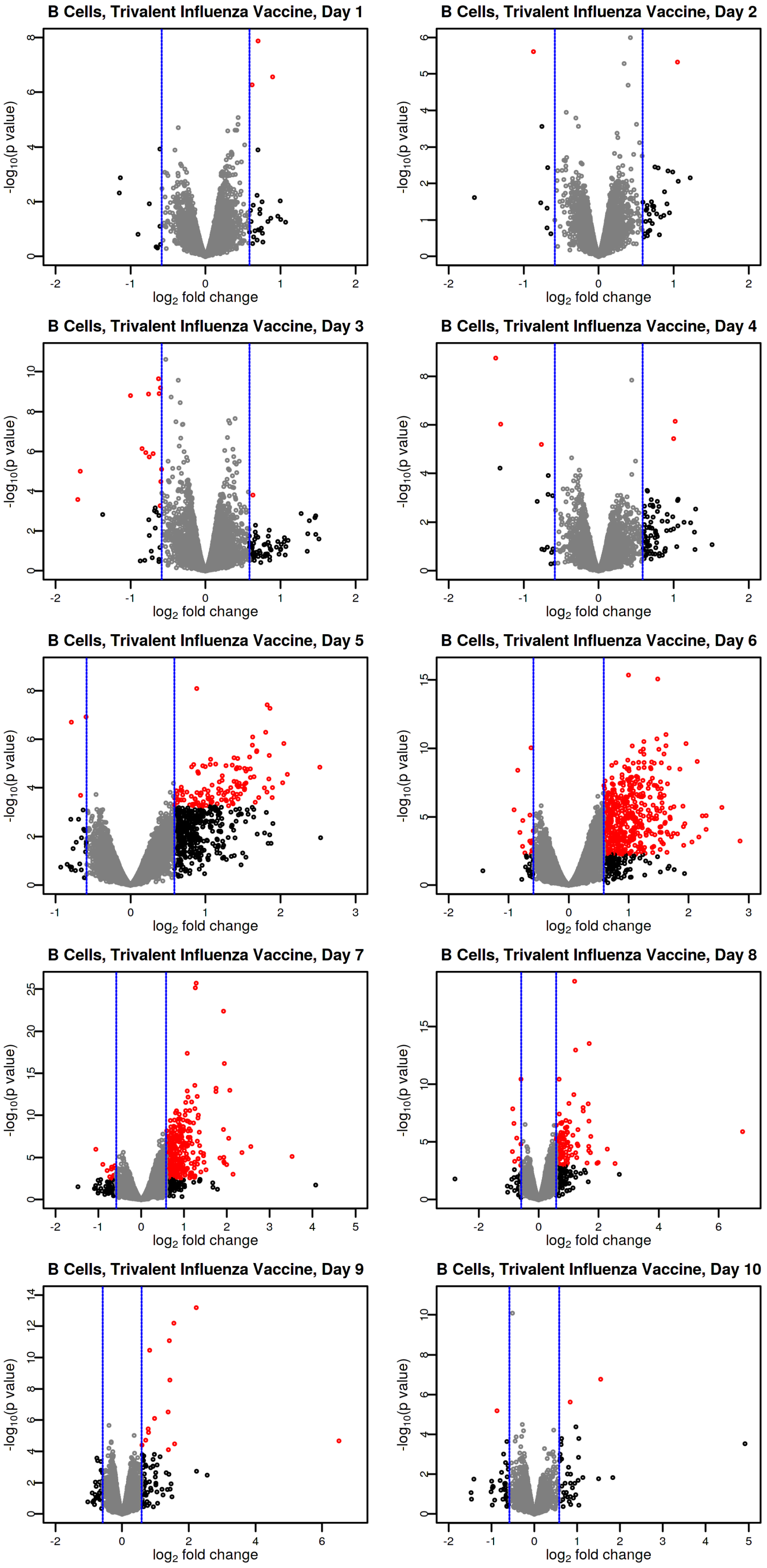

Figure 20: Volcano plots (Trivalent Influenza Vaccine, B Cells). In red: DE genes; in grey: genes that did not pass the fold change cut offs; in black: genes that passed the fold change cut off but were not DE.

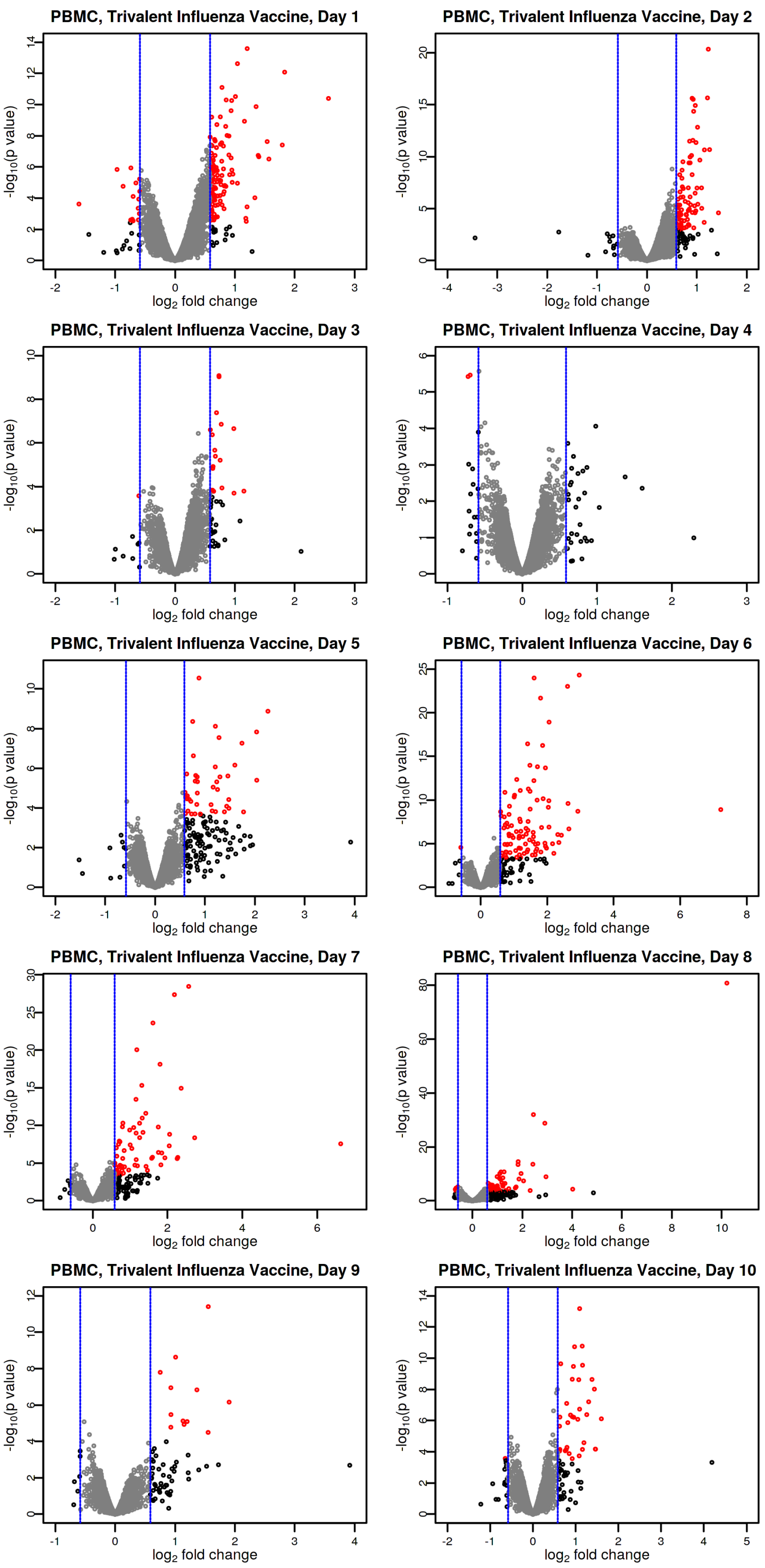

Figure 21: Volcano plots (Trivalent Influenza Vaccine, PBMC). In red: DE genes; in grey: genes that did not pass the fold change cut offs; in black: genes that passed the fold change cut off but were not DE.

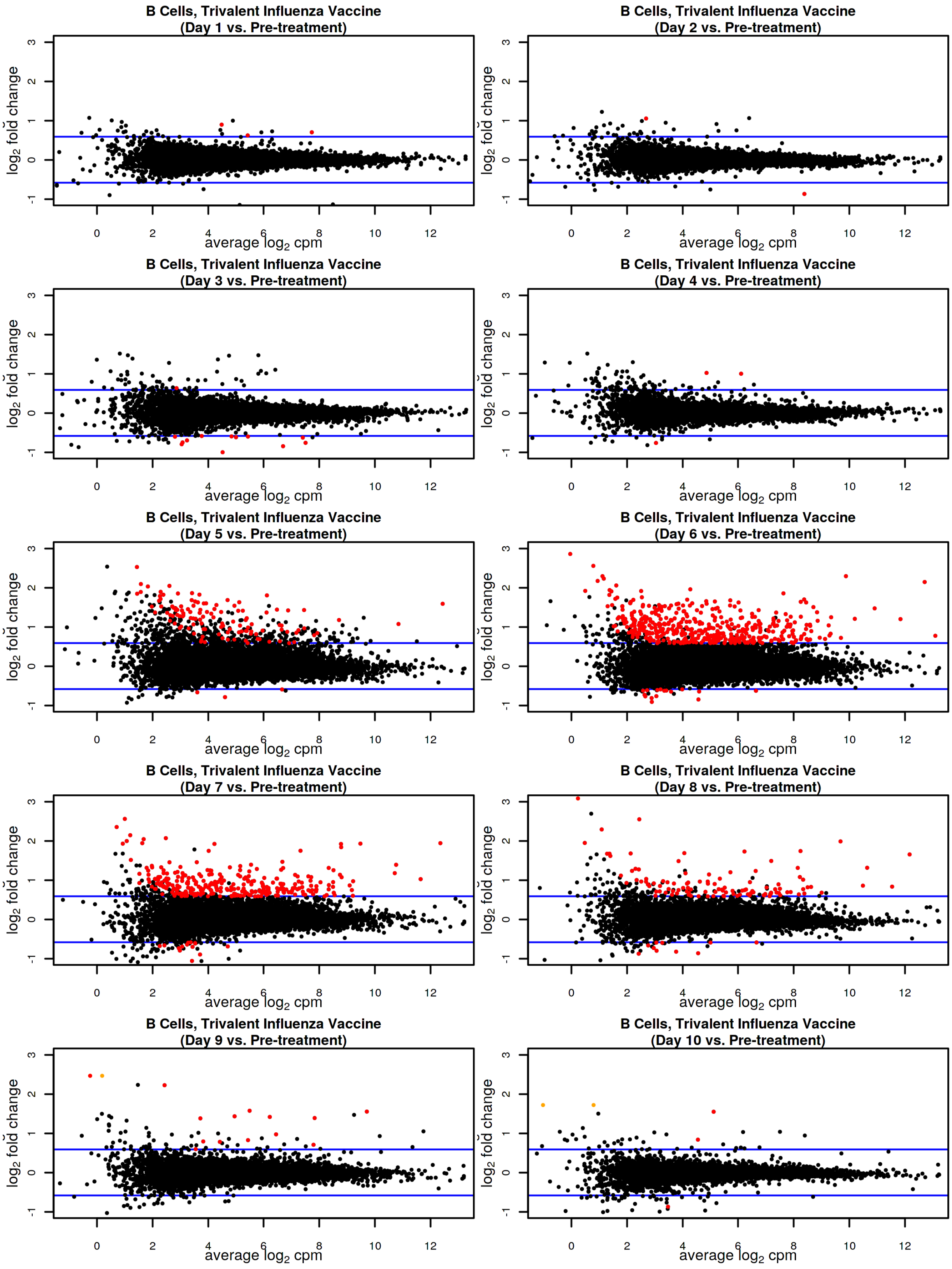

Figure 22: MA plot (B Cells). Average log_2 counts per million is displayed on the x-axis, the y-axis shows average log_2 fold change from pre-treatment. Blue lines indicate the pre-specified minimum fold change cut off. DE genes are colored in red. Top optimize the visualization, the largest and smallest 0.1% of logFC observations are not included (0.1% < x < 99.9%).

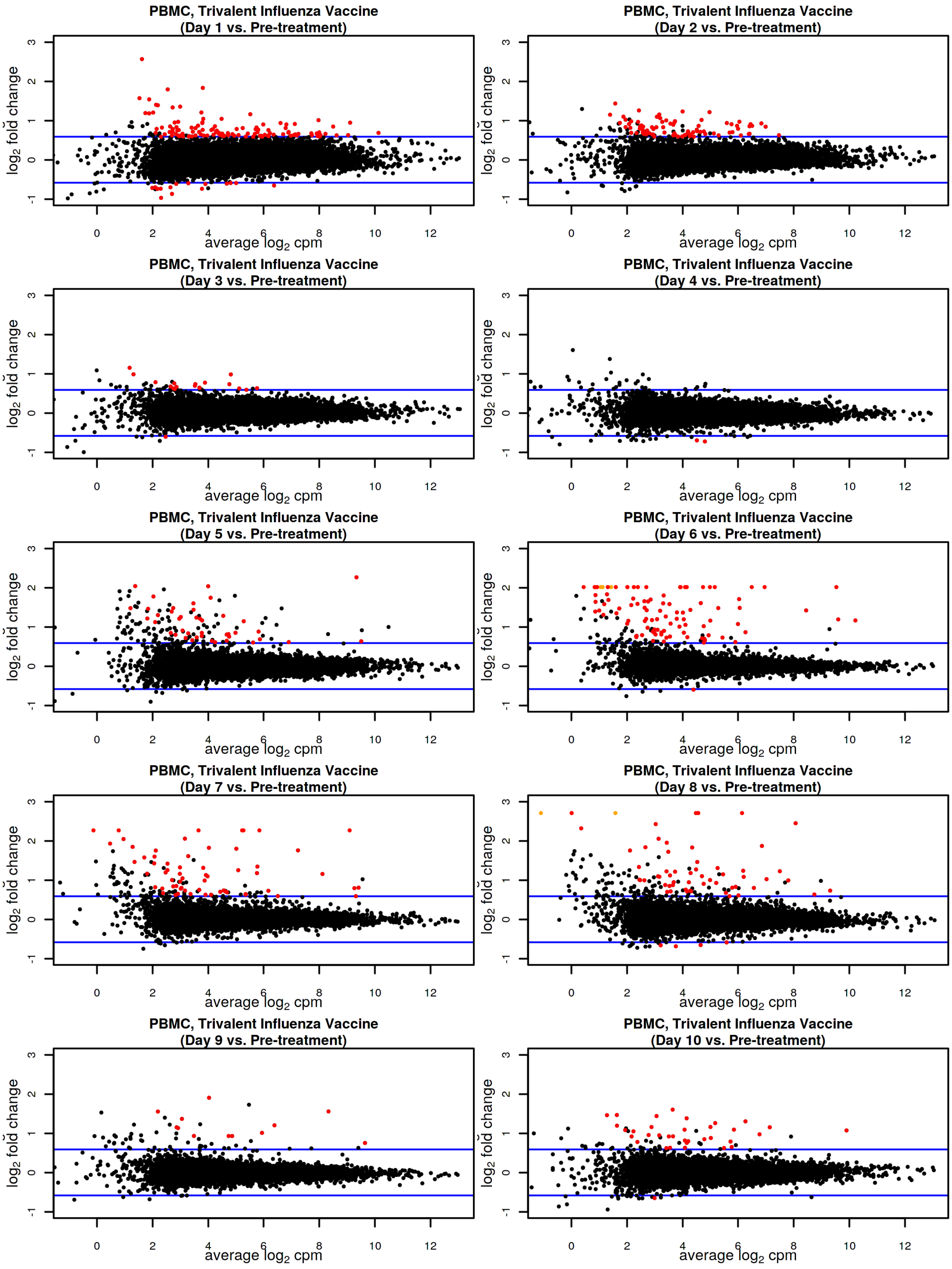

Figure 23: MA plot (PBMC). Average log_2 counts per million is displayed on the x-axis, the y-axis shows average log_2 fold change from pre-treatment. Blue lines indicate the pre-specified minimum fold change cut off. DE genes are colored in red. Top optimize the visualization, the largest and smallest 0.1% of logFC observations are not included (0.1% $<$ x $<$ 99.9%).

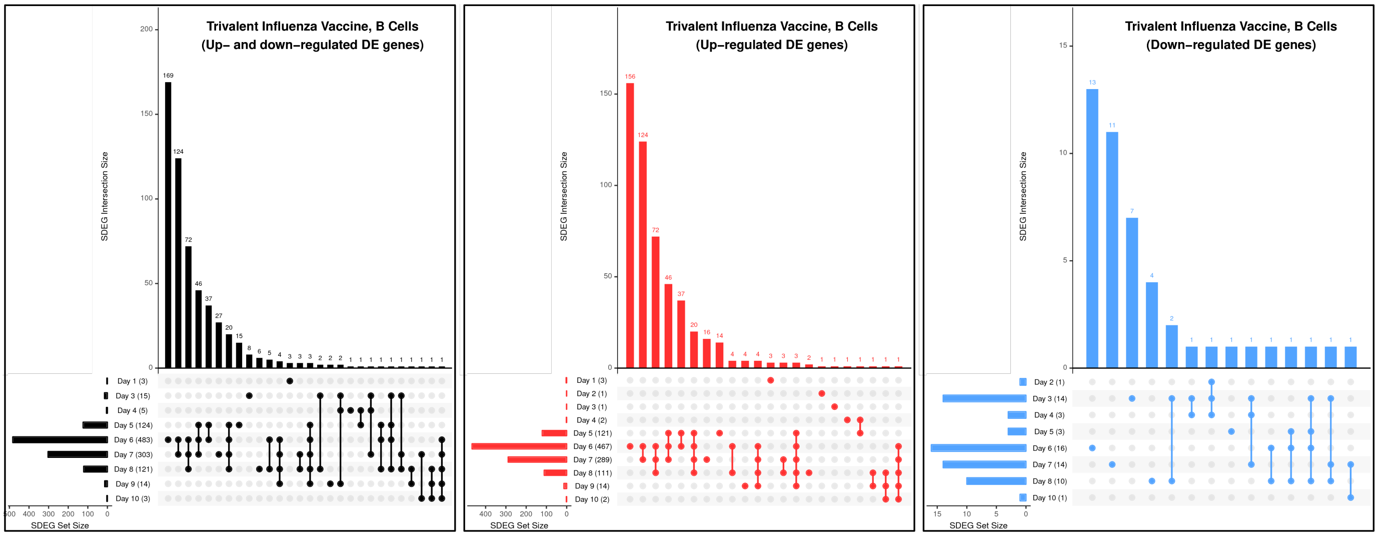

Figure 24: UpSet plots summarizing overlap in DE genes between post-treatment time points (B Cells, Trivalent Influenza Vaccine). In red: up-regulated compared to pre-treatment, in blue: down-regulated compared to pre-treatment.

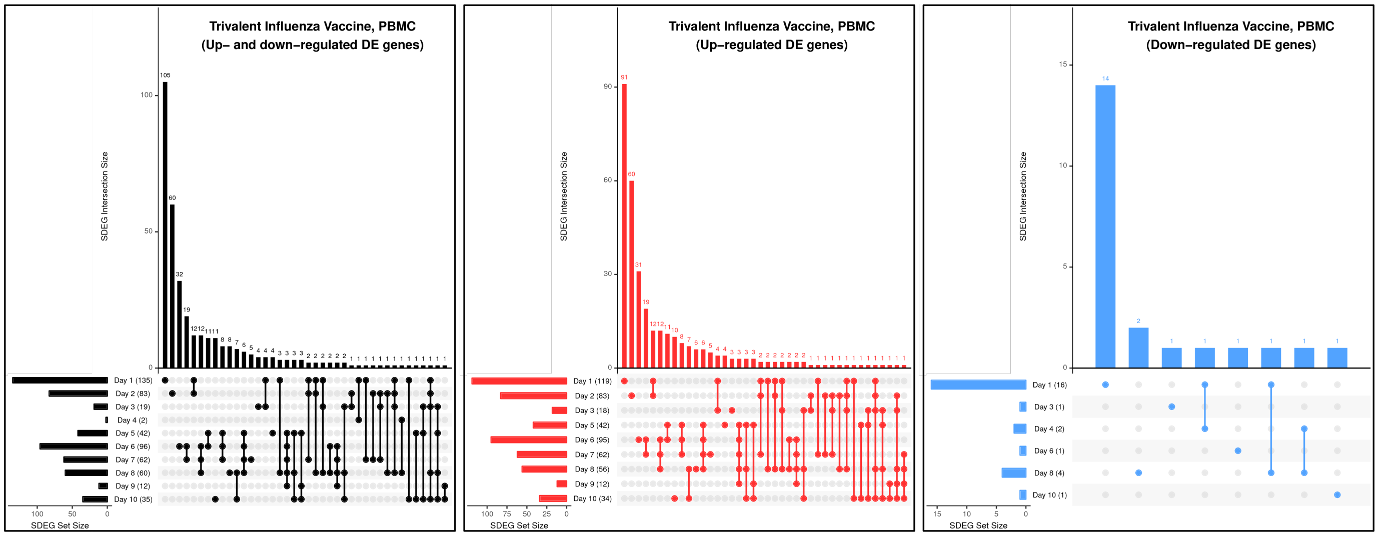

Figure 25: UpSet plots summarizing overlap in DE genes between post-treatment time points (PBMC, Trivalent Influenza Vaccine). In red: up-regulated compared to pre-treatment, in blue: down-regulated compared to pre-treatment.

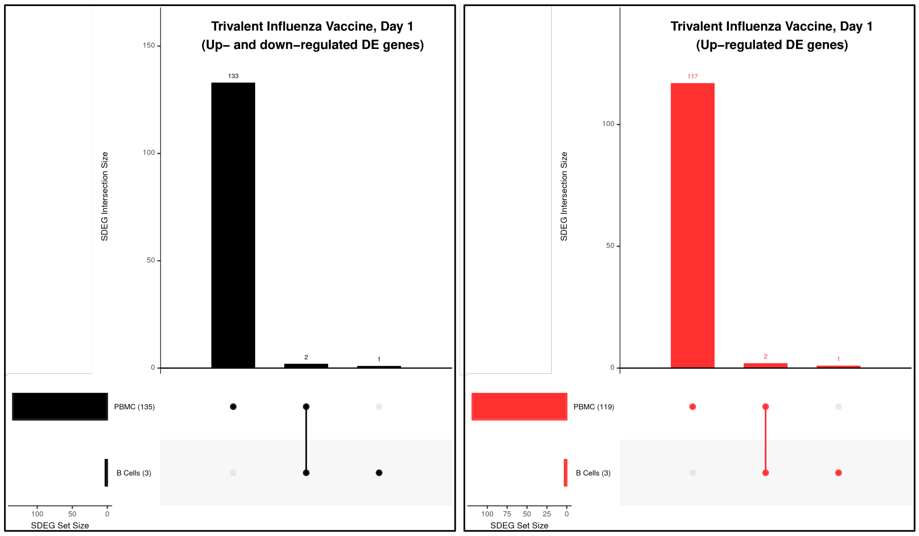

Figure 26: UpSet plots summarizing overlap in DE genes between specimen types (Trivalent Influenza Vaccine, Day 1). In red: up-regulated from pre-treatment, in blue: down-regulated from pre-treatment.

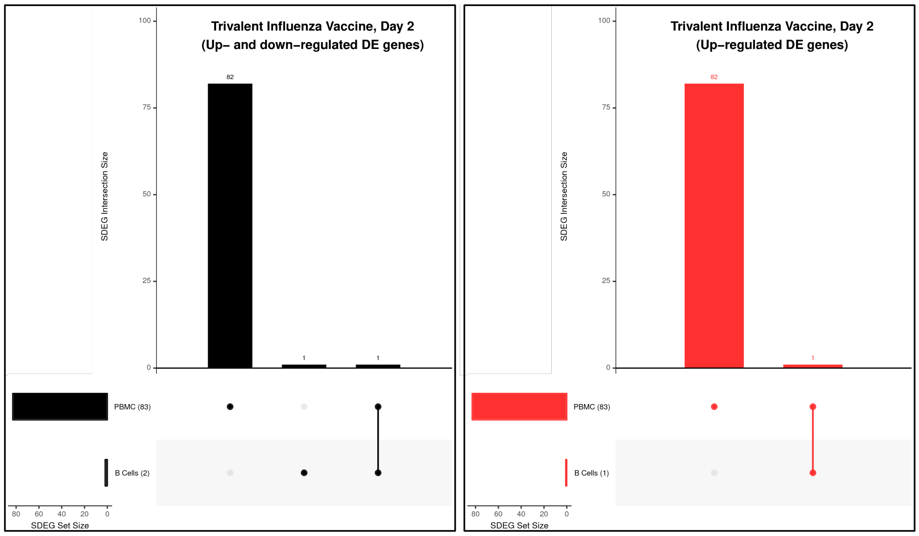

Figure 27: UpSet plots summarizing overlap in DE genes between specimen types (Trivalent Influenza Vaccine, Day 2). In red: up-regulated from pre-treatment, in blue: down-regulated from pre-treatment.

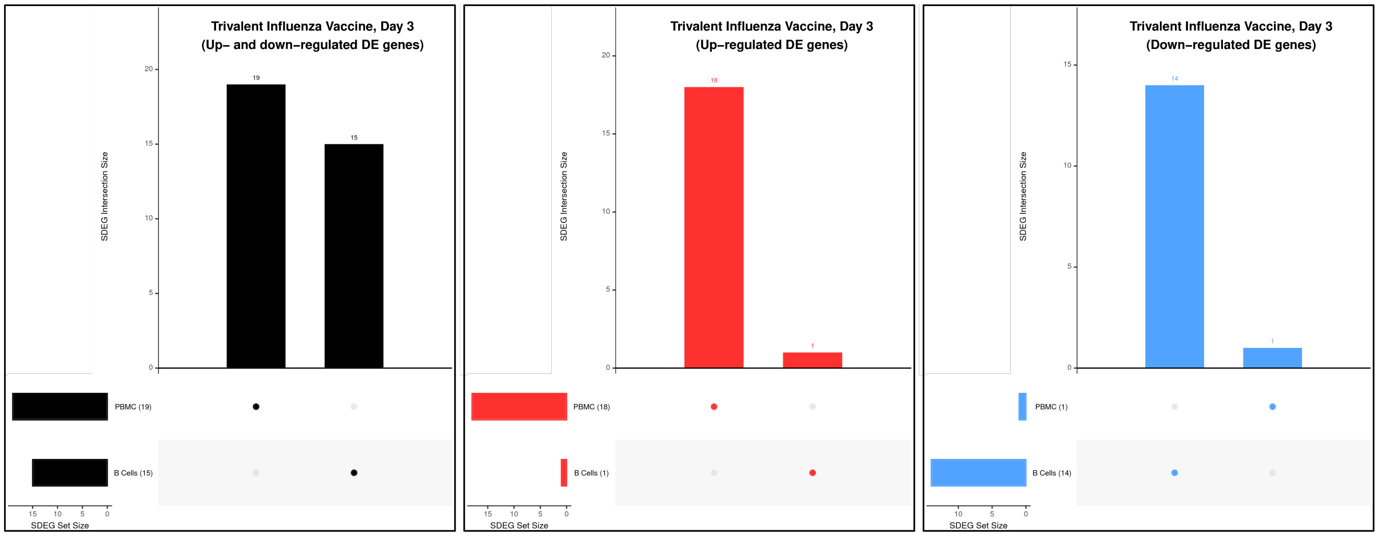

Figure 28: UpSet plots summarizing overlap in DE genes between specimen types (Trivalent Influenza Vaccine, Day 3). In red: up-regulated from pre-treatment, in blue: down-regulated from pre-treatment.

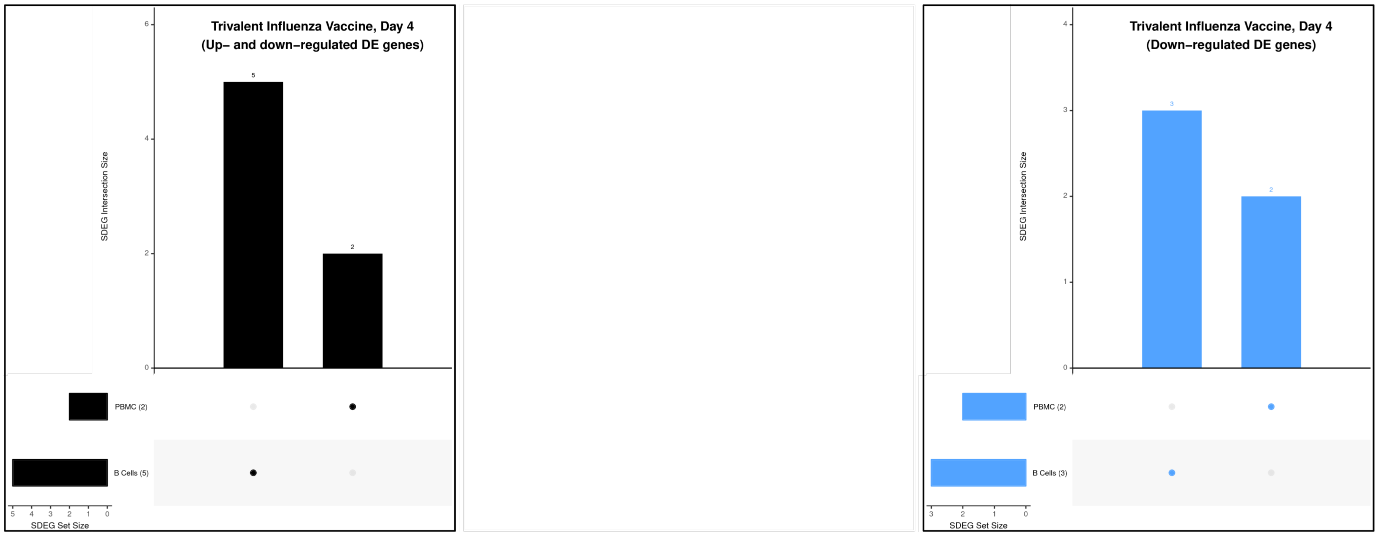

Figure 29: UpSet plots summarizing overlap in DE genes between specimen types (Trivalent Influenza Vaccine, Day 4). In red: up-regulated from pre-treatment, in blue: down-regulated from pre-treatment.

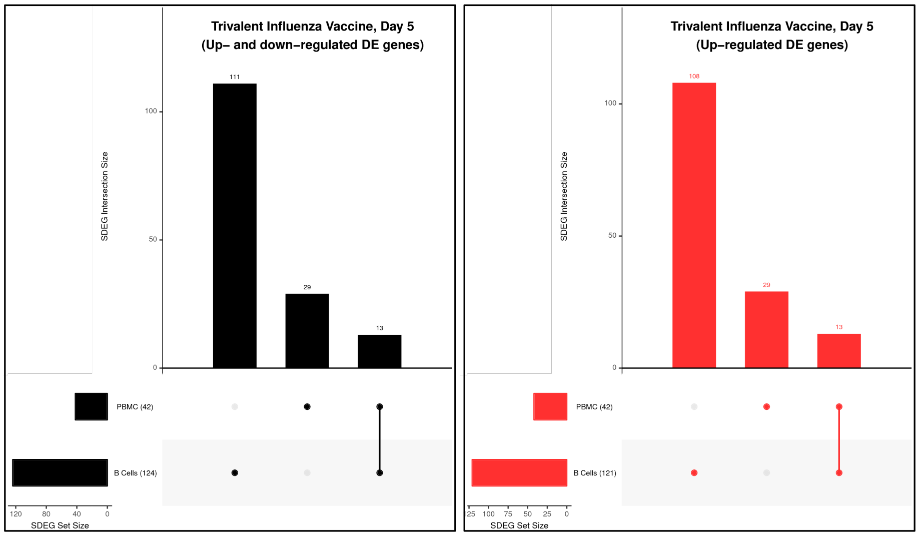

Figure 30: UpSet plots summarizing overlap in DE genes between specimen types (Trivalent Influenza Vaccine, Day 5). In red: up-regulated from pre-treatment, in blue: down-regulated from pre-treatment.

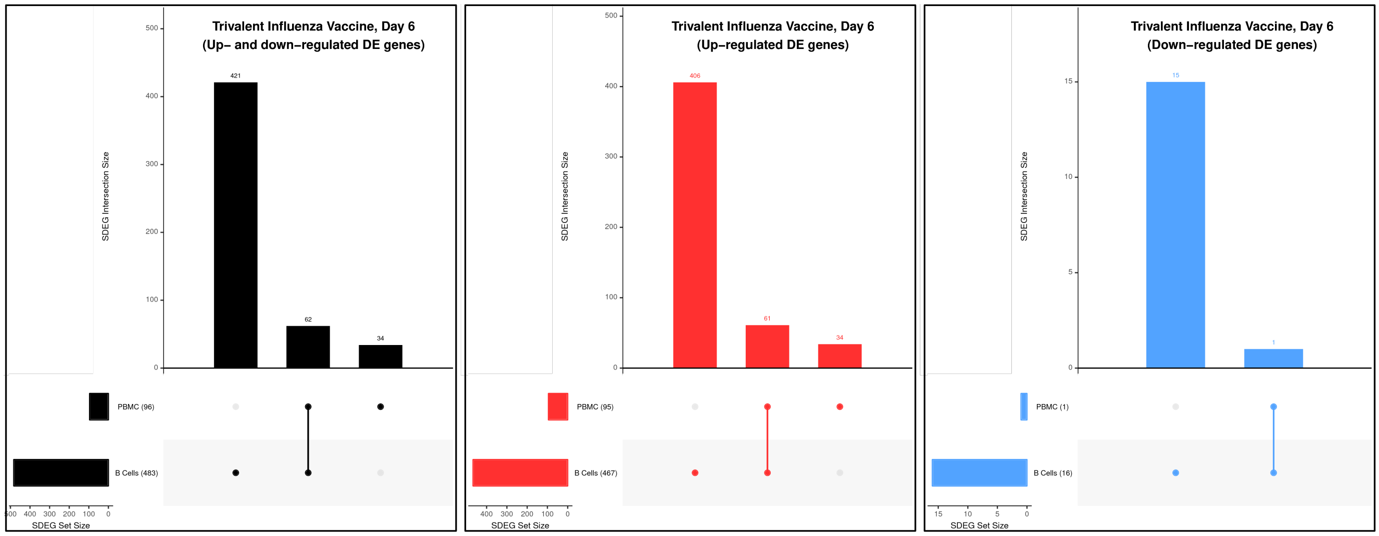

Figure 31: UpSet plots summarizing overlap in DE genes between specimen types (Trivalent Influenza Vaccine, Day 6). In red: up-regulated from pre-treatment, in blue: down-regulated from pre-treatment.

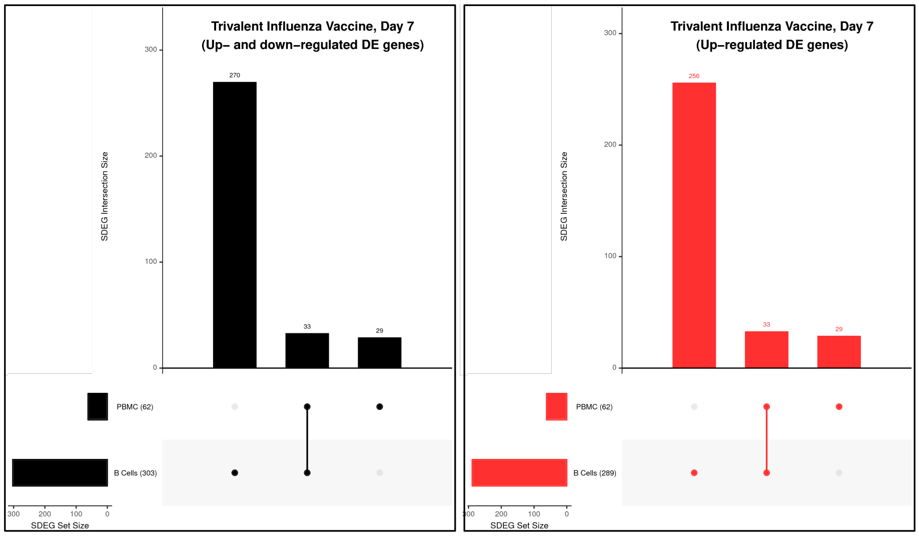

Figure 32: UpSet plots summarizing overlap in DE genes between specimen types (Trivalent Influenza Vaccine, Day 7). In red: up-regulated from pre-treatment, in blue: down-regulated from pre-treatment.

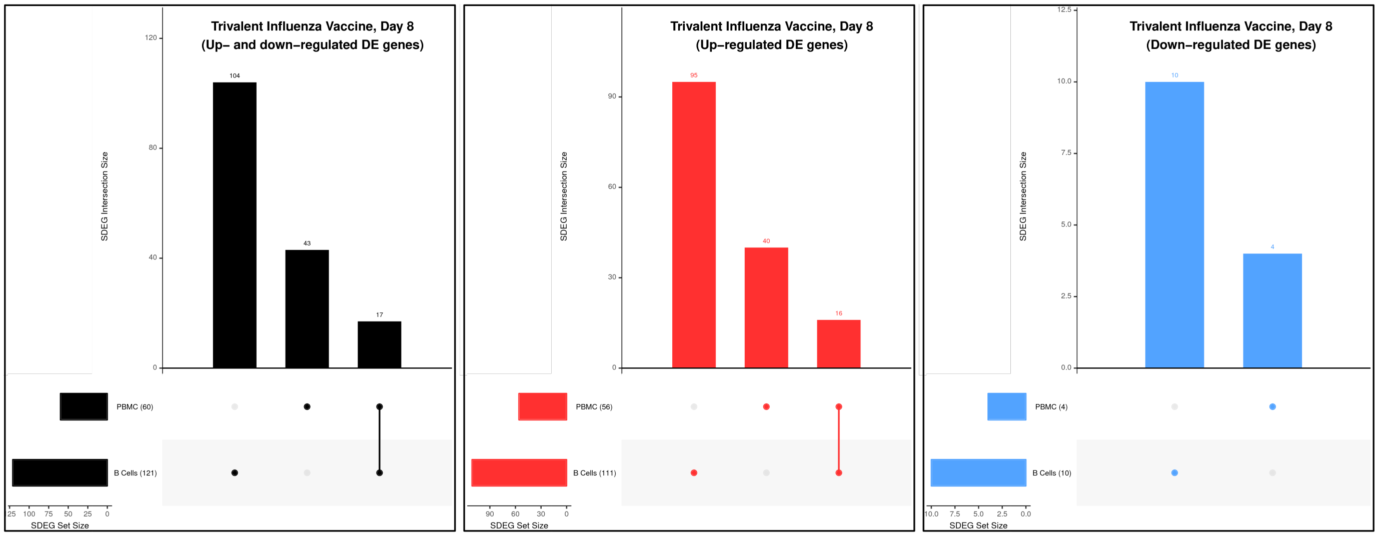

Figure 33: UpSet plots summarizing overlap in DE genes between specimen types (Trivalent Influenza Vaccine, Day 8). In red: up-regulated from pre-treatment, in blue: down-regulated from pre-treatment.

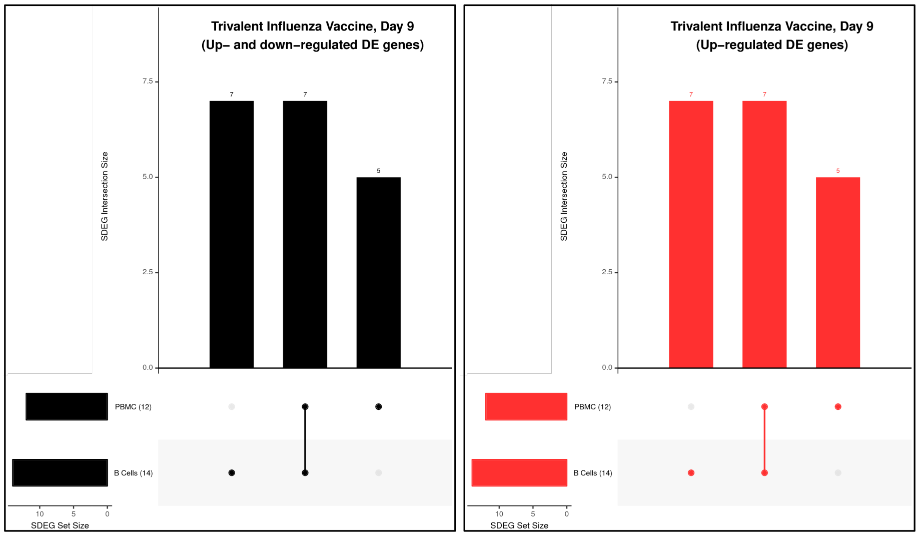

Figure 34: UpSet plots summarizing overlap in DE genes between specimen types (Trivalent Influenza Vaccine, Day 9). In red: up-regulated from pre-treatment, in blue: down-regulated from pre-treatment.

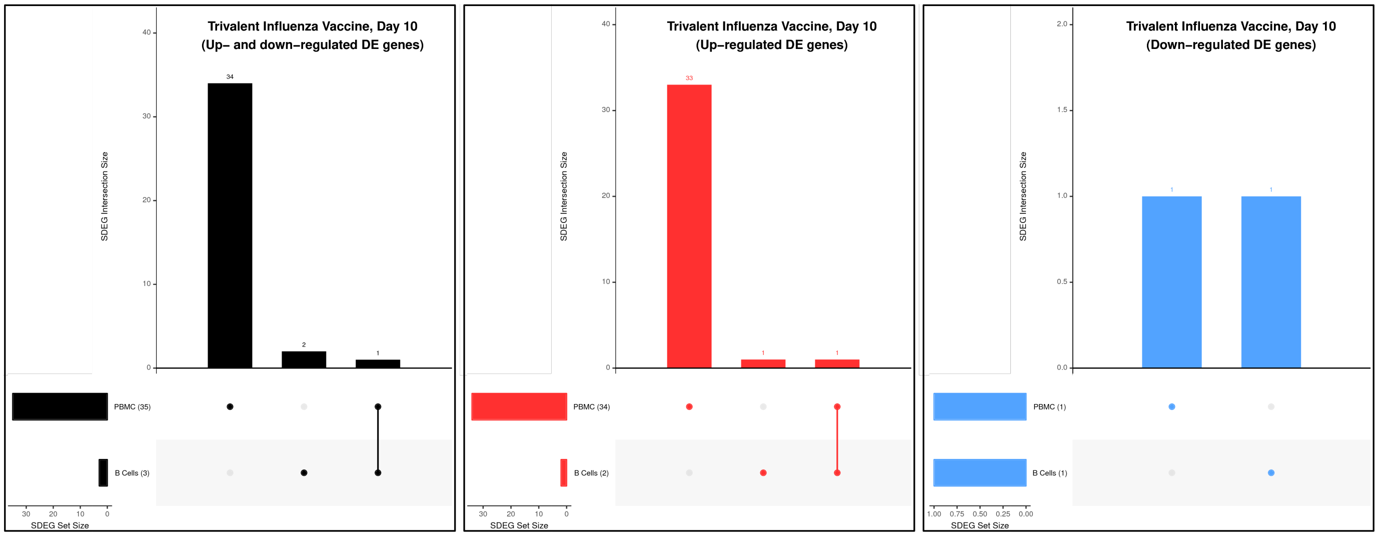

Figure 35: UpSet plots summarizing overlap in DE genes between specimen types (Trivalent Influenza Vaccine, Day 10). In red: up-regulated from pre-treatment, in blue: down-regulated from pre-treatment.

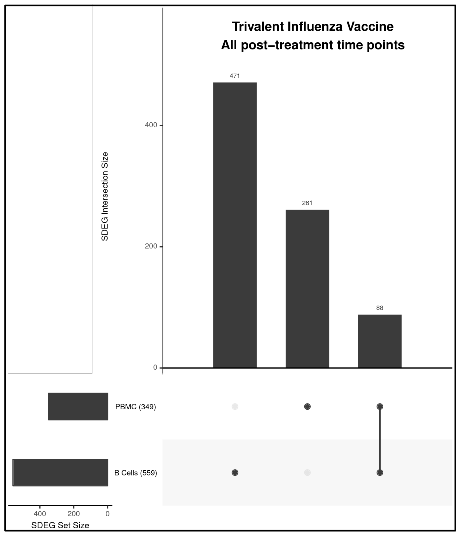

Figure 36: UpSet plots summarizing overlap in DE genes between specimen types (Trivalent Influenza Vaccine, All post-treatment time points). In red: up-regulated from pre-treatment, in blue: down-regulated from pre-treatment.

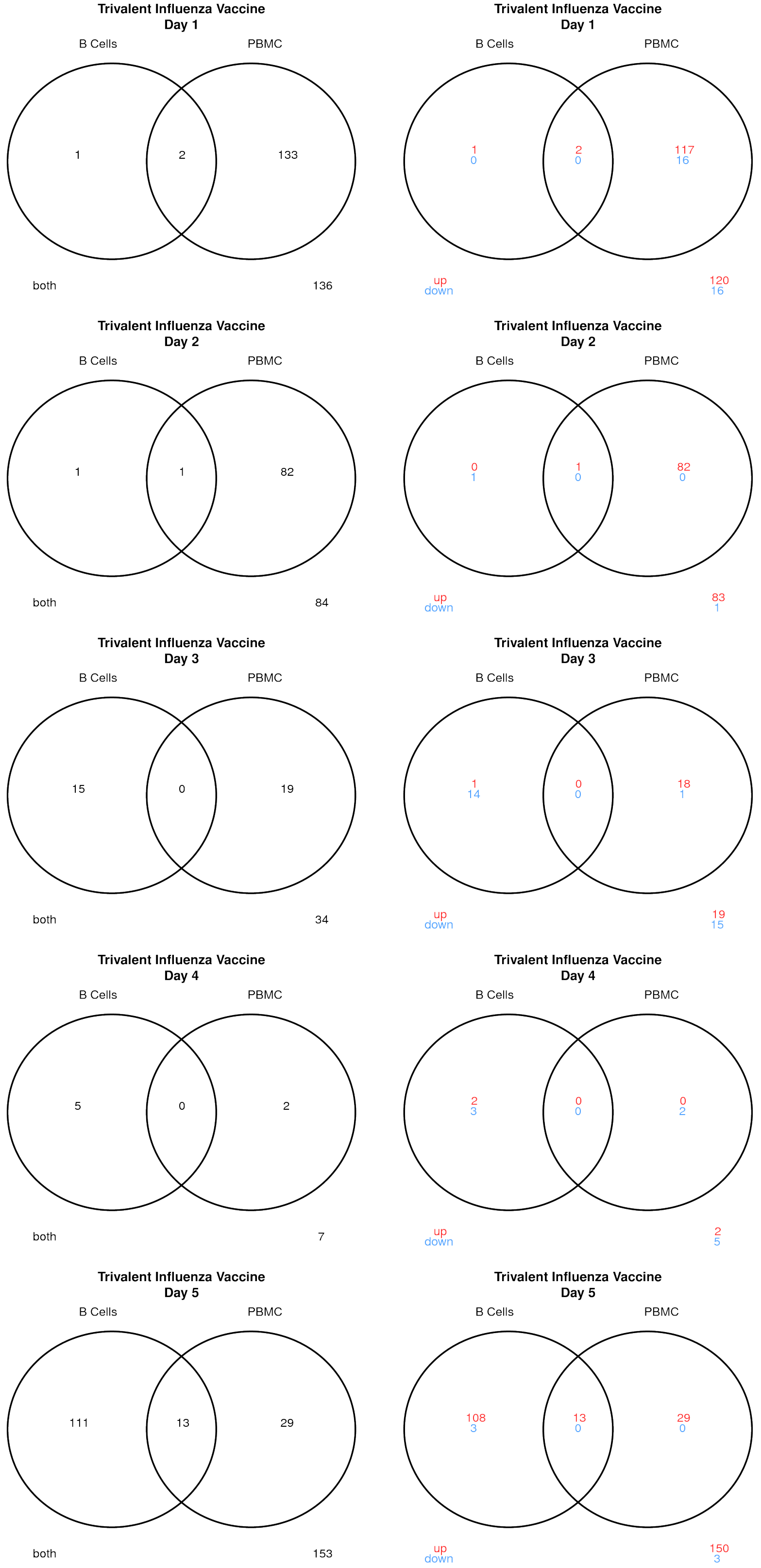

Figure 37: Venn diagrams summarizing overlap in DE genes between specimen types 1 of 3 (Trivalent Influenza Vaccine). In red: up-regulated from pre-treatment, in blue: down-regulated from pre-treatment.

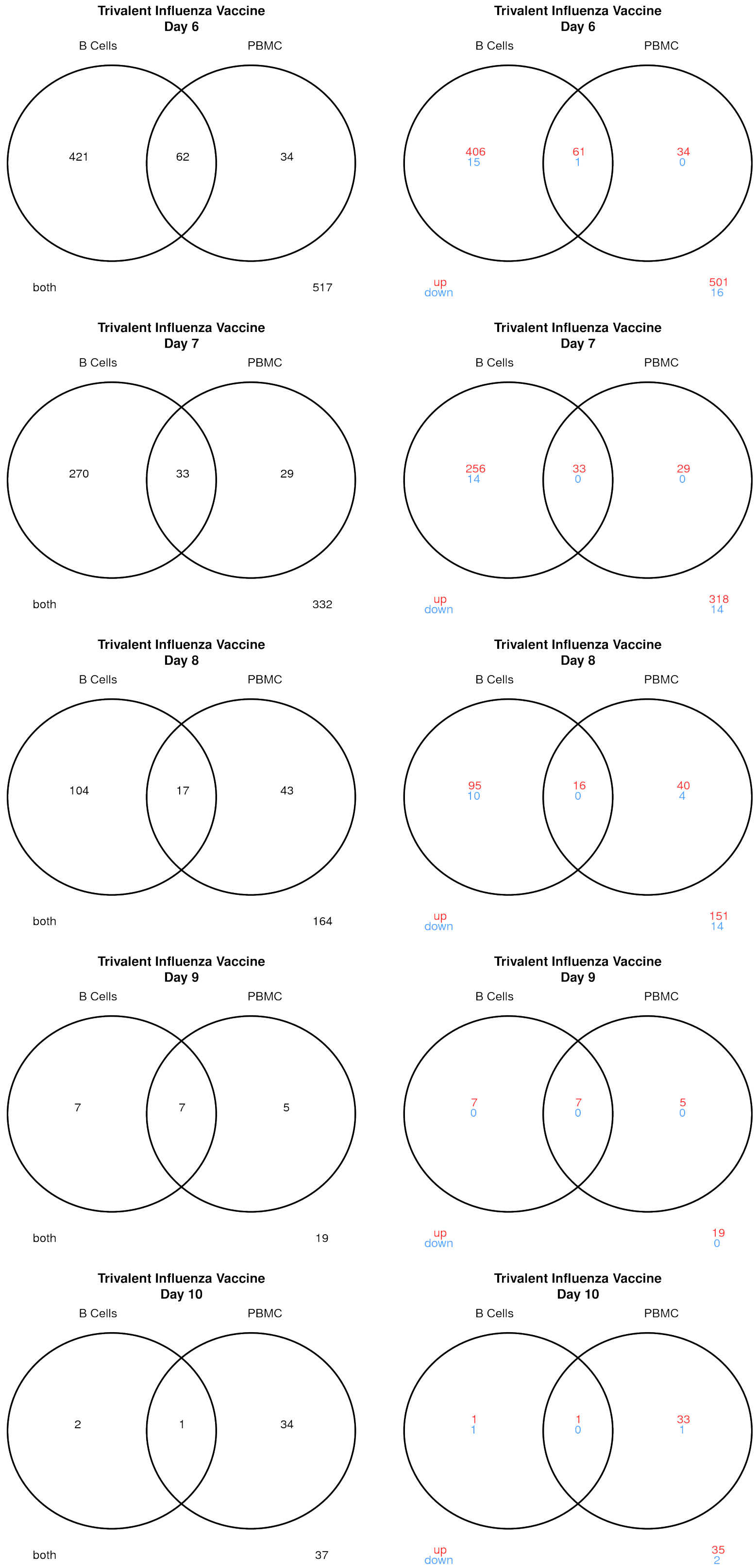

Figure 38: Venn diagrams summarizing overlap in DE genes between specimen types 2 of 3 (Trivalent Influenza Vaccine). In red: up-regulated from pre-treatment, in blue: down-regulated from pre-treatment.

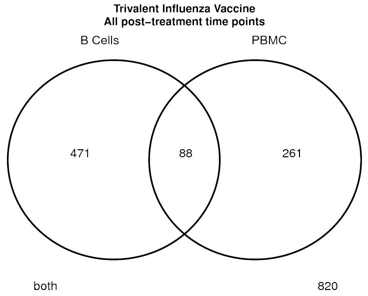

Figure 39: Venn diagrams summarizing overlap in DE genes between specimen types 3 of 3 (Trivalent Influenza Vaccine). In red: up-regulated from pre-treatment, in blue: down-regulated from pre-treatment.

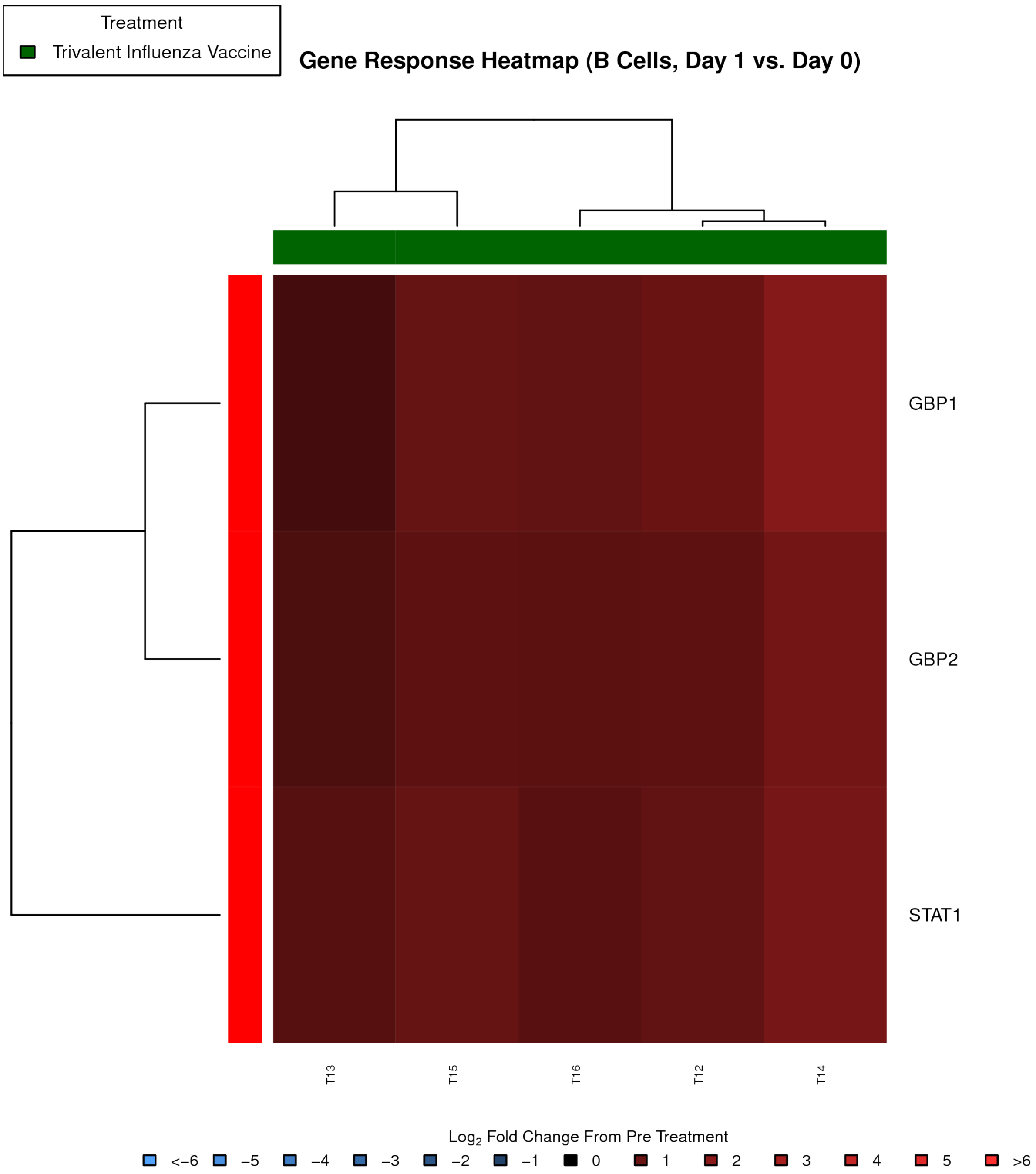

Figure 40: Heatmap of log_2 fold change from pre-treatment (B Cells, Day 1). Rows represent DE genes across treatment types, columns represent samples. In red: up regulated compared to pre-treatment; in green: down-regulated compared to pre-treatment. Dendrograms were obtained using complete linkage clustering of uncentered pairwise Pearson correlation distances for log_2 fold changes. Samples are color-coded by treatment group (see row below sample dendrogram).

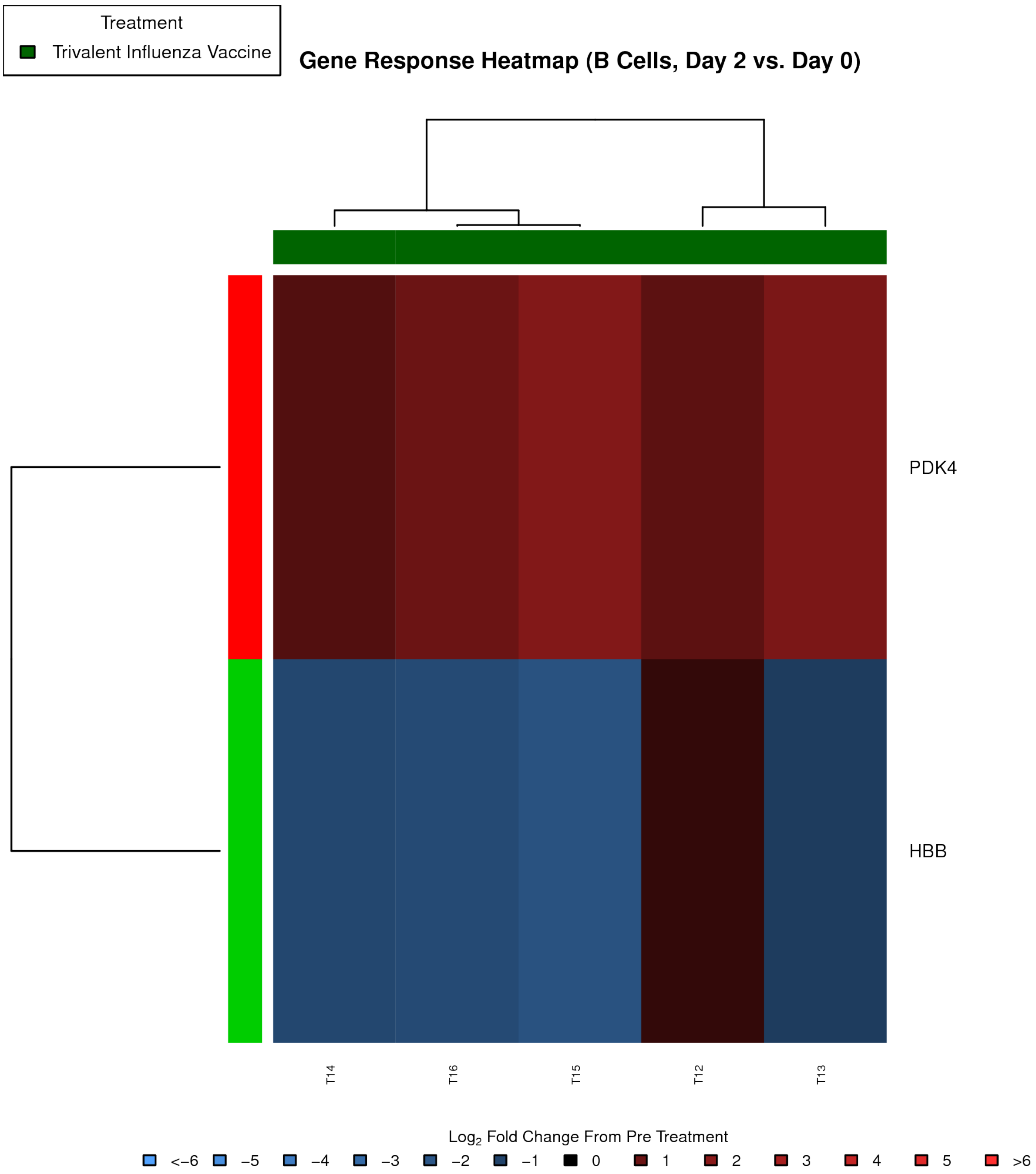

Figure 41: Heatmap of log_2 fold change from pre-treatment (B Cells, Day 2). Rows represent DE genes across treatment types, columns represent samples. In red: up regulated compared to pre-treatment; in green: down-regulated compared to pre-treatment. Dendrograms were obtained using complete linkage clustering of uncentered pairwise Pearson correlation distances for log_2 fold changes. Samples are color-coded by treatment group (see row below sample dendrogram).

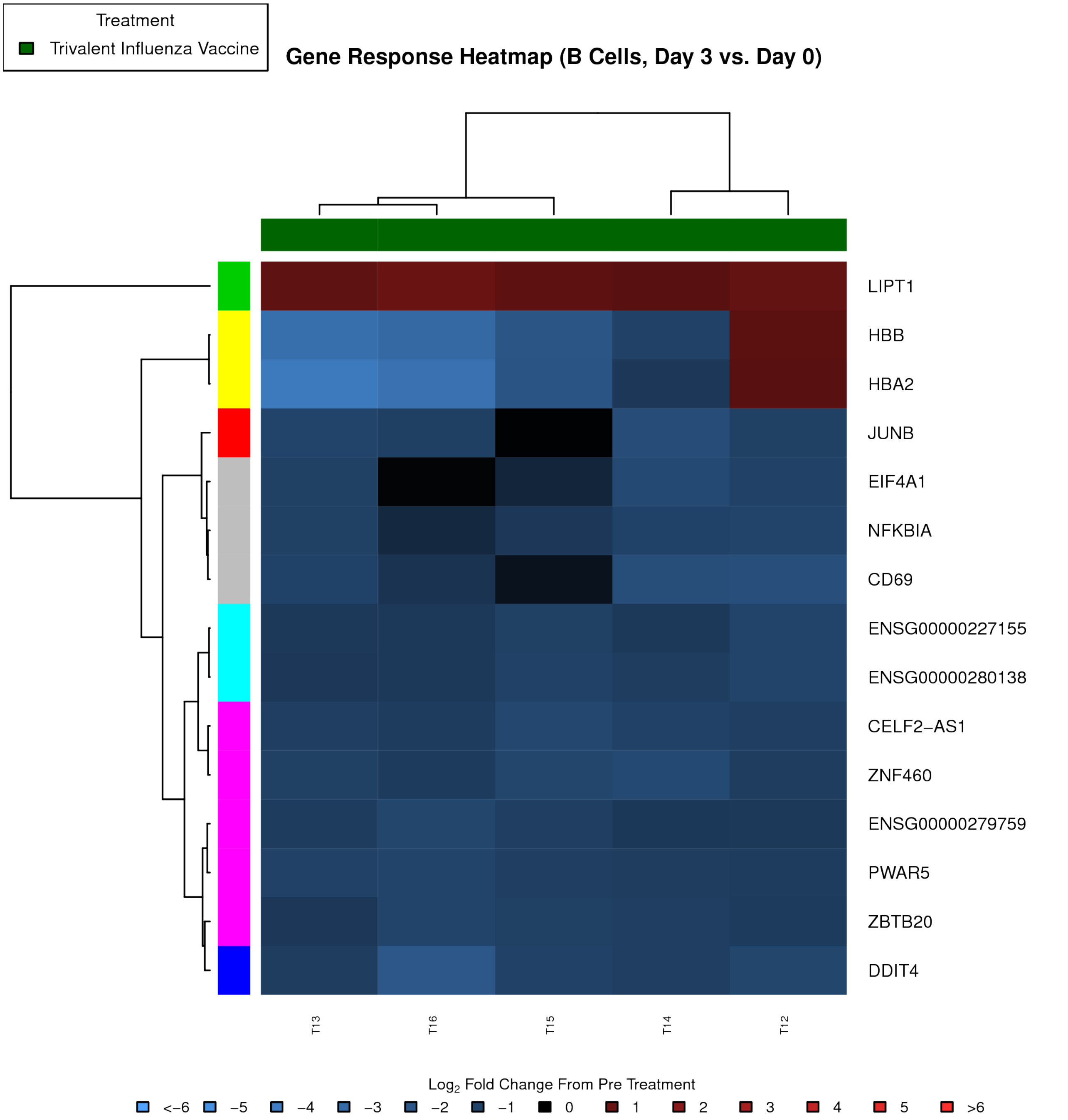

Figure 42: Heatmap of log_2 fold change from pre-treatment (B Cells, Day 3). Rows represent DE genes across treatment types, columns represent samples. In red: up regulated compared to pre-treatment; in green: down-regulated compared to pre-treatment. Dendrograms were obtained using complete linkage clustering of uncentered pairwise Pearson correlation distances for log_2 fold changes. Samples are color-coded by treatment group (see row below sample dendrogram).

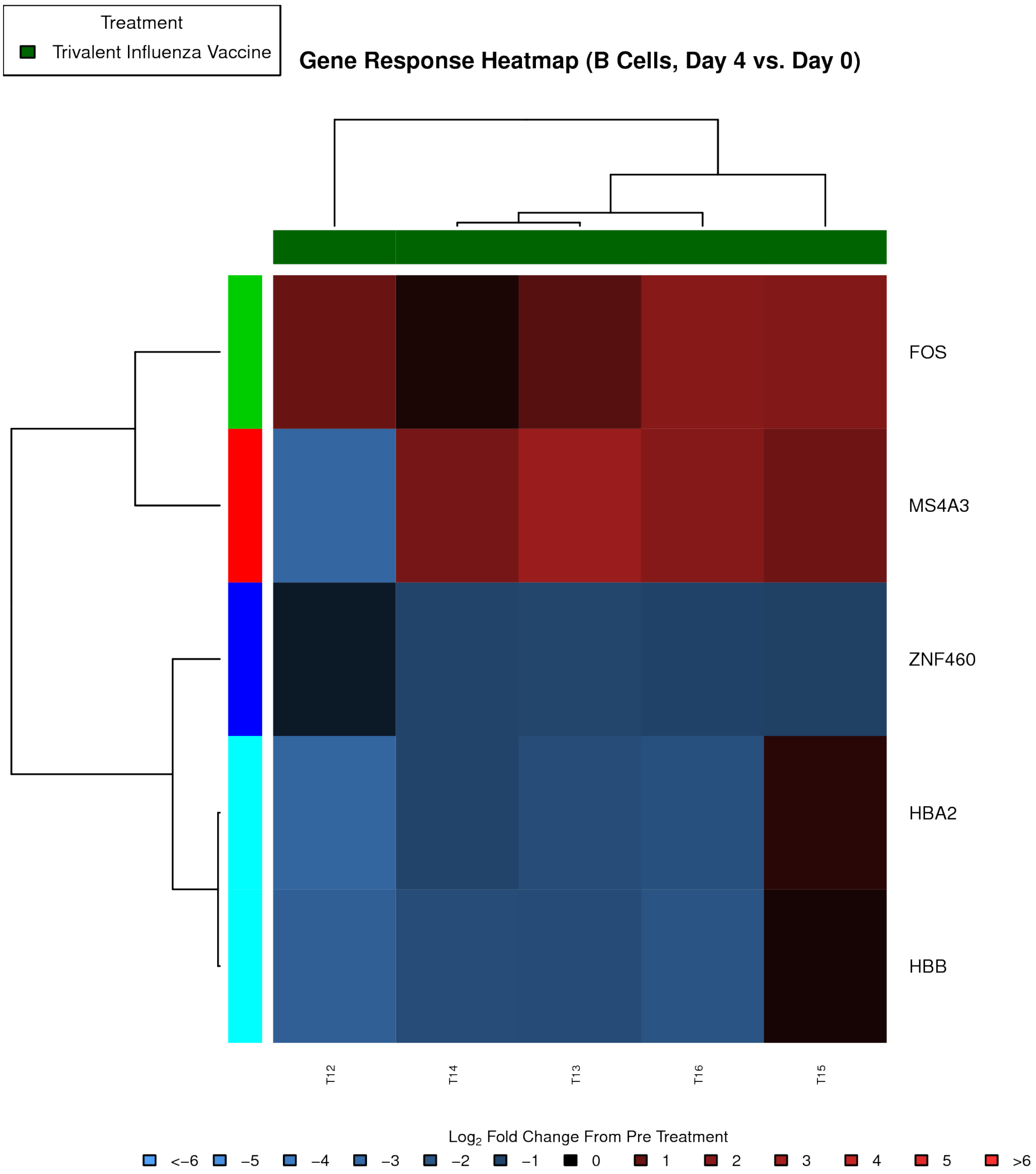

Figure 43: Heatmap of log_2 fold change from pre-treatment (B Cells, Day 4). Rows represent DE genes across treatment types, columns represent samples. In red: up regulated compared to pre-treatment; in green: down-regulated compared to pre-treatment. Dendrograms were obtained using complete linkage clustering of uncentered pairwise Pearson correlation distances for log_2 fold changes. Samples are color-coded by treatment group (see row below sample dendrogram).

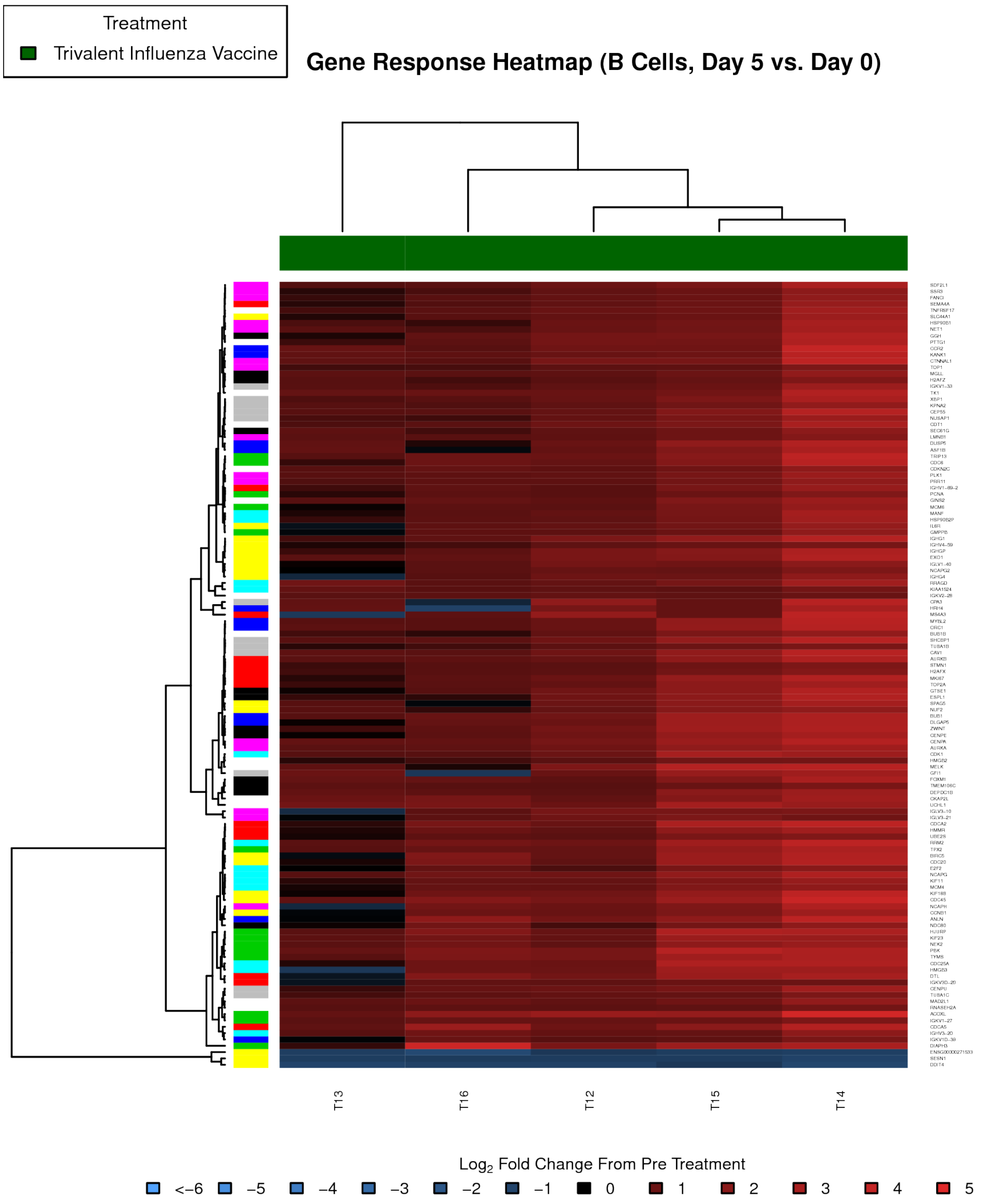

Figure 44: Heatmap of log_2 fold change from pre-treatment (B Cells, Day 5). Rows represent DE genes across treatment types, columns represent samples. In red: up regulated compared to pre-treatment; in green: down-regulated compared to pre-treatment. Dendrograms were obtained using complete linkage clustering of uncentered pairwise Pearson correlation distances for log_2 fold changes. Samples are color-coded by treatment group (see row below sample dendrogram).

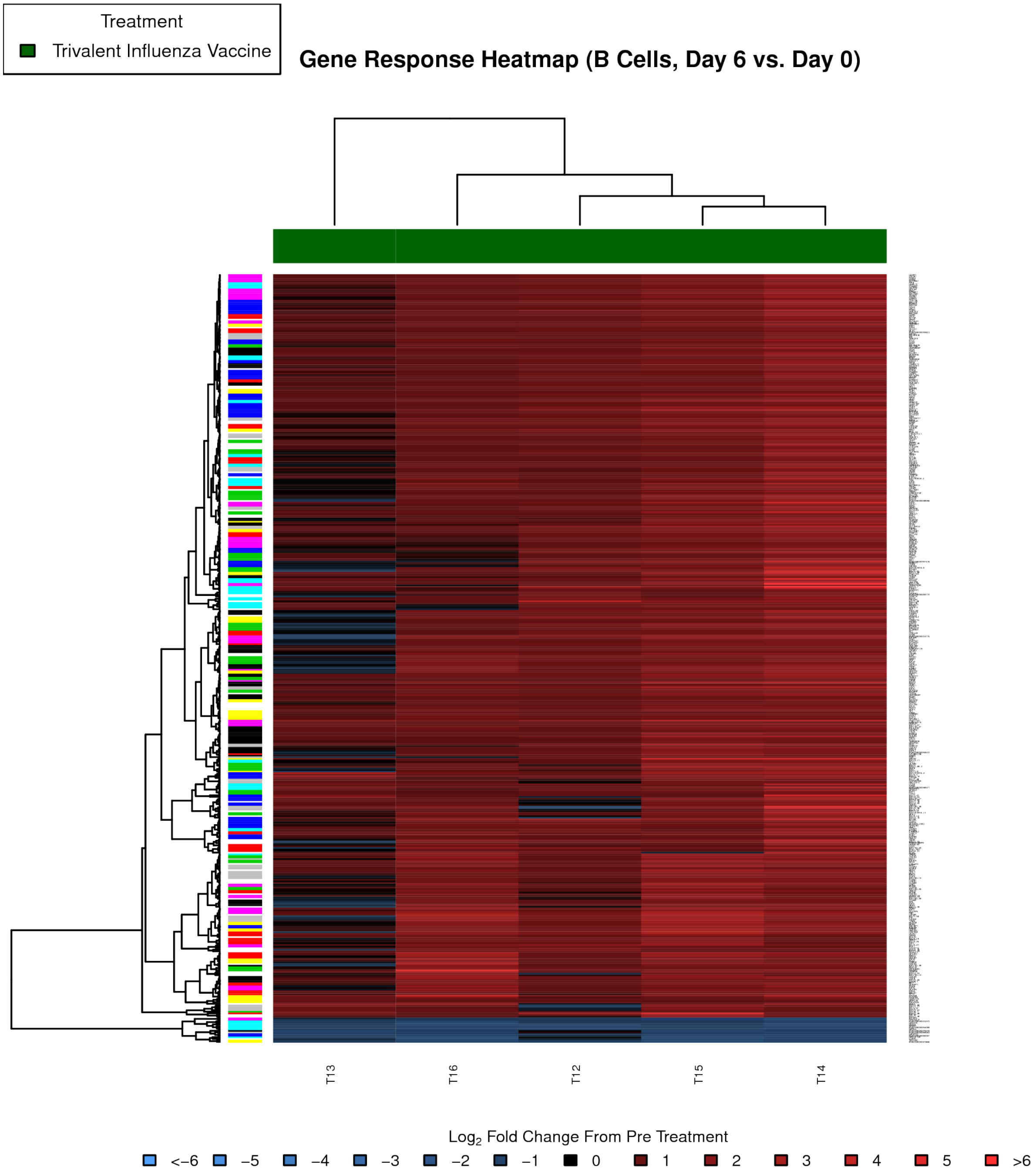

Figure 45: Heatmap of log_2 fold change from pre-treatment (B Cells, Day 6). Rows represent DE genes across treatment types, columns represent samples. In red: up regulated compared to pre-treatment; in green: down-regulated compared to pre-treatment. Dendrograms were obtained using complete linkage clustering of uncentered pairwise Pearson correlation distances for log_2 fold changes. Samples are color-coded by treatment group (see row below sample dendrogram).

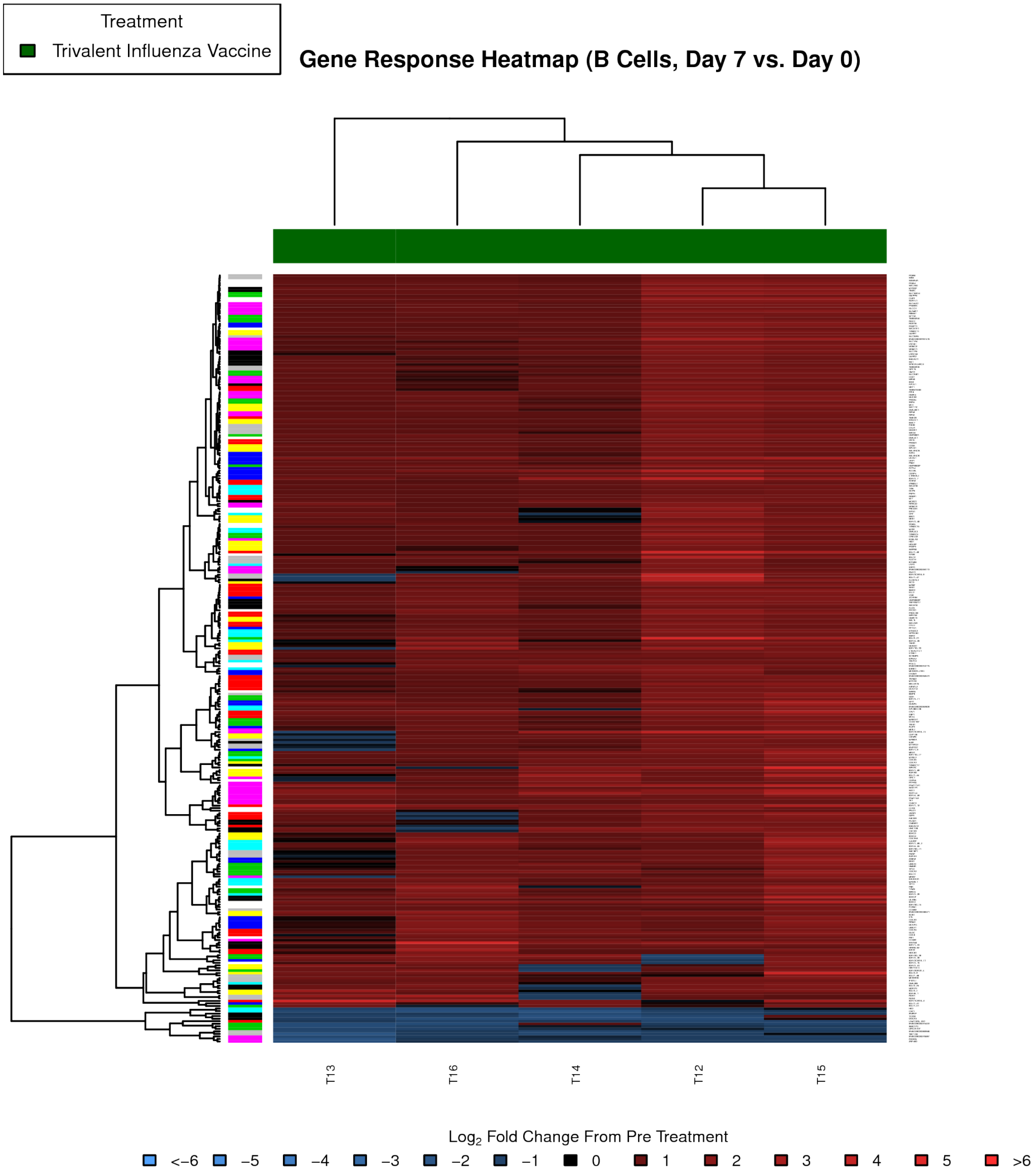

Figure 46: Heatmap of log_2 fold change from pre-treatment (B Cells, Day 7). Rows represent DE genes across treatment types, columns represent samples. In red: up regulated compared to pre-treatment; in green: down-regulated compared to pre-treatment. Dendrograms were obtained using complete linkage clustering of uncentered pairwise Pearson correlation distances for log_2 fold changes. Samples are color-coded by treatment group (see row below sample dendrogram).

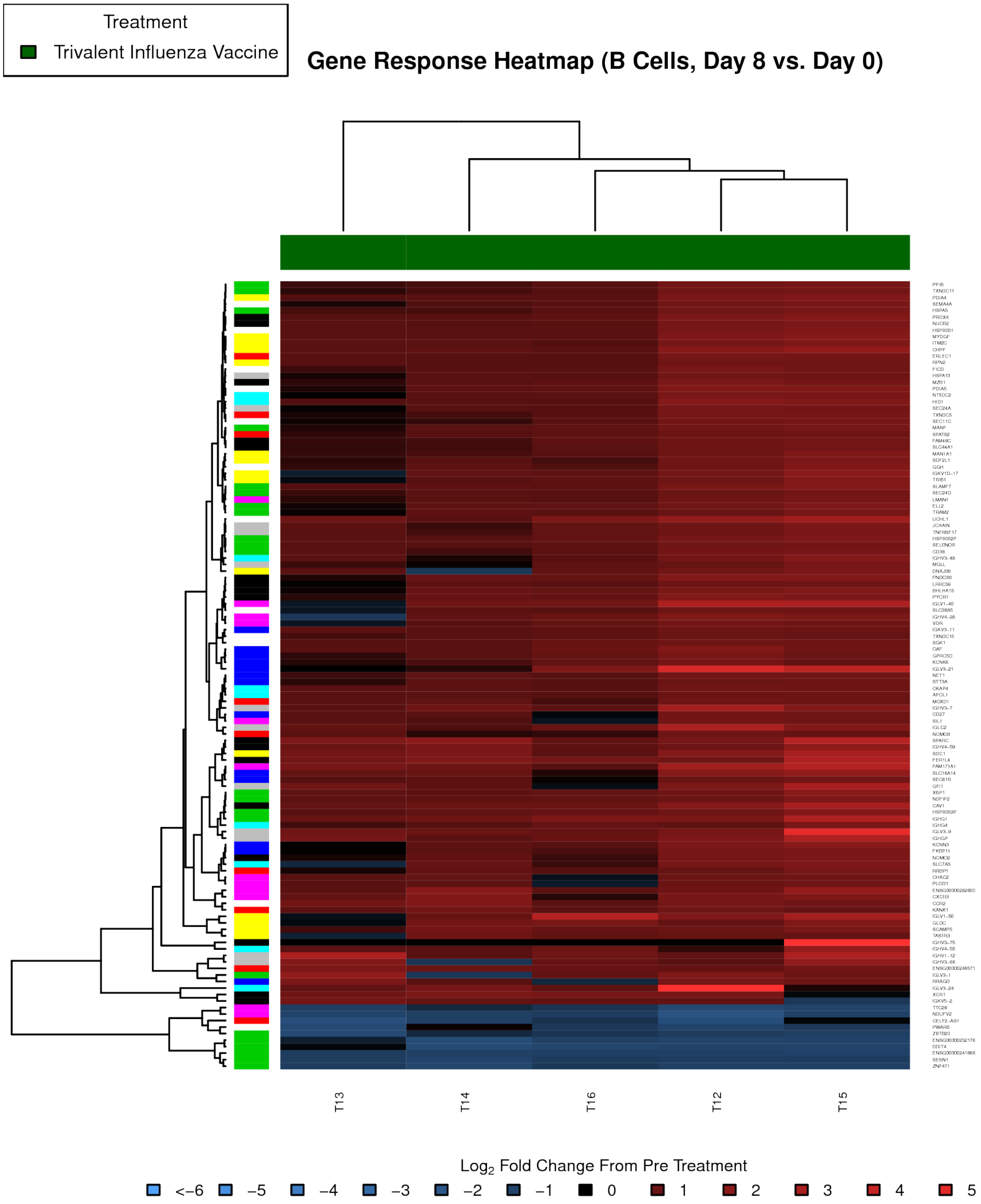

Figure 47: Heatmap of log_2 fold change from pre-treatment (B Cells, Day 8). Rows represent DE genes across treatment types, columns represent samples. In red: up regulated compared to pre-treatment; in green: down-regulated compared to pre-treatment. Dendrograms were obtained using complete linkage clustering of uncentered pairwise Pearson correlation distances for log_2 fold changes. Samples are color-coded by treatment group (see row below sample dendrogram).

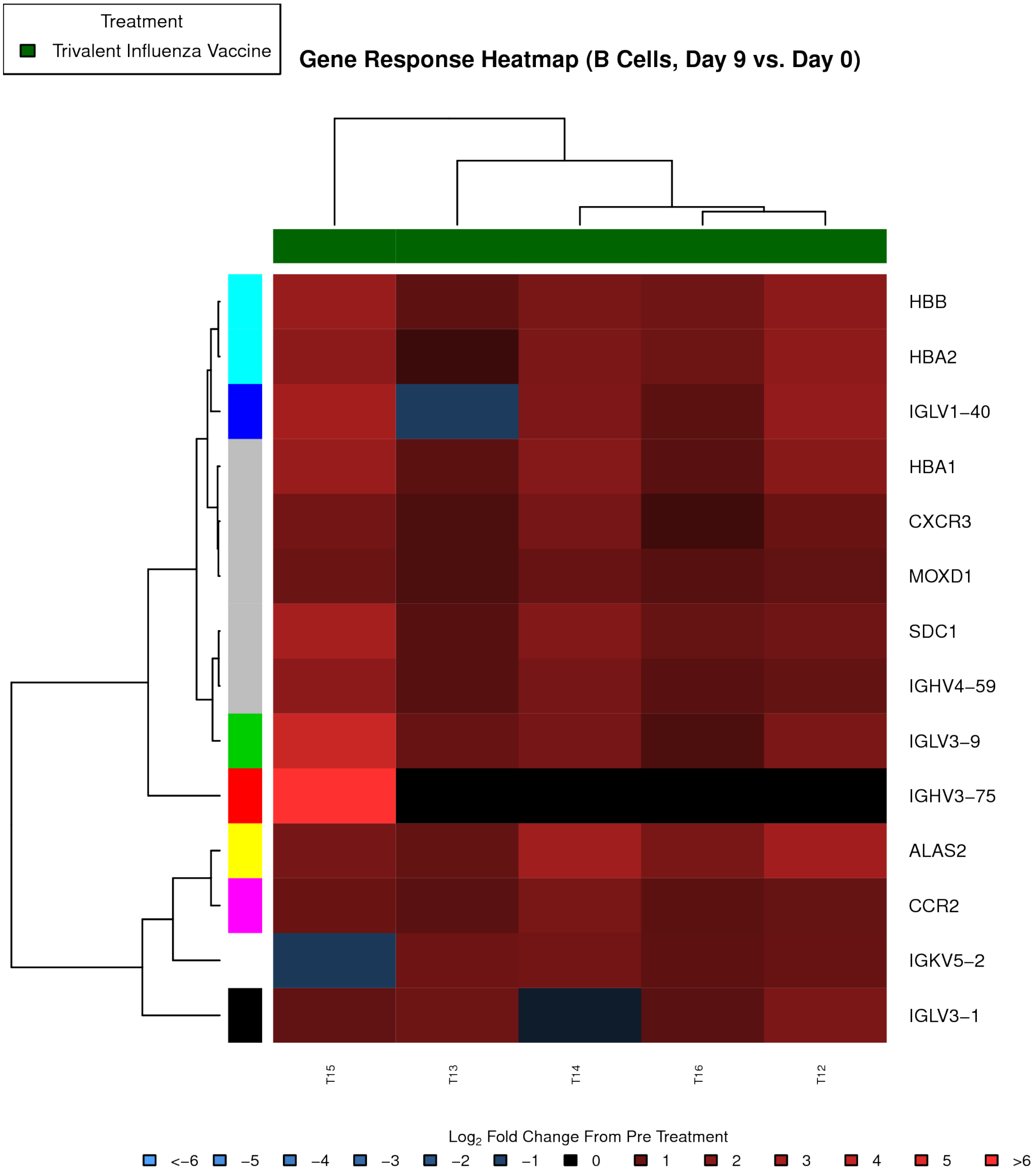

Figure 48: Heatmap of log_2 fold change from pre-treatment (B Cells, Day 9). Rows represent DE genes across treatment types, columns represent samples. In red: up regulated compared to pre-treatment; in green: down-regulated compared to pre-treatment. Dendrograms were obtained using complete linkage clustering of uncentered pairwise Pearson correlation distances for log_2 fold changes. Samples are color-coded by treatment group (see row below sample dendrogram).

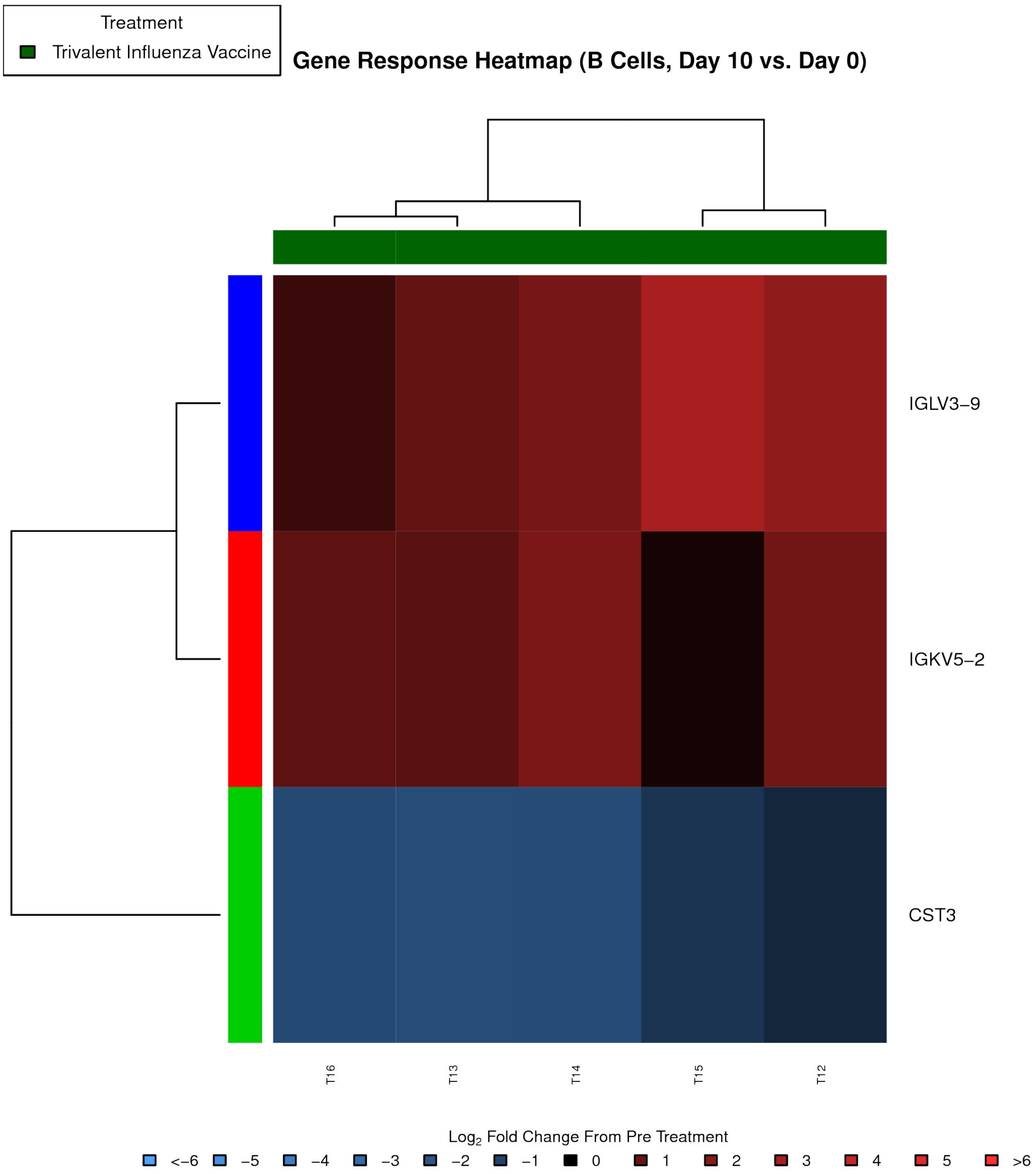

Figure 49: Heatmap of log_2 fold change from pre-treatment (B Cells, Day 10). Rows represent DE genes across treatment types, columns represent samples. In red: up regulated compared to pre-treatment; in green: down-regulated compared to pre-treatment. Dendrograms were obtained using complete linkage clustering of uncentered pairwise Pearson correlation distances for log_2 fold changes. Samples are color-coded by treatment group (see row below sample dendrogram).

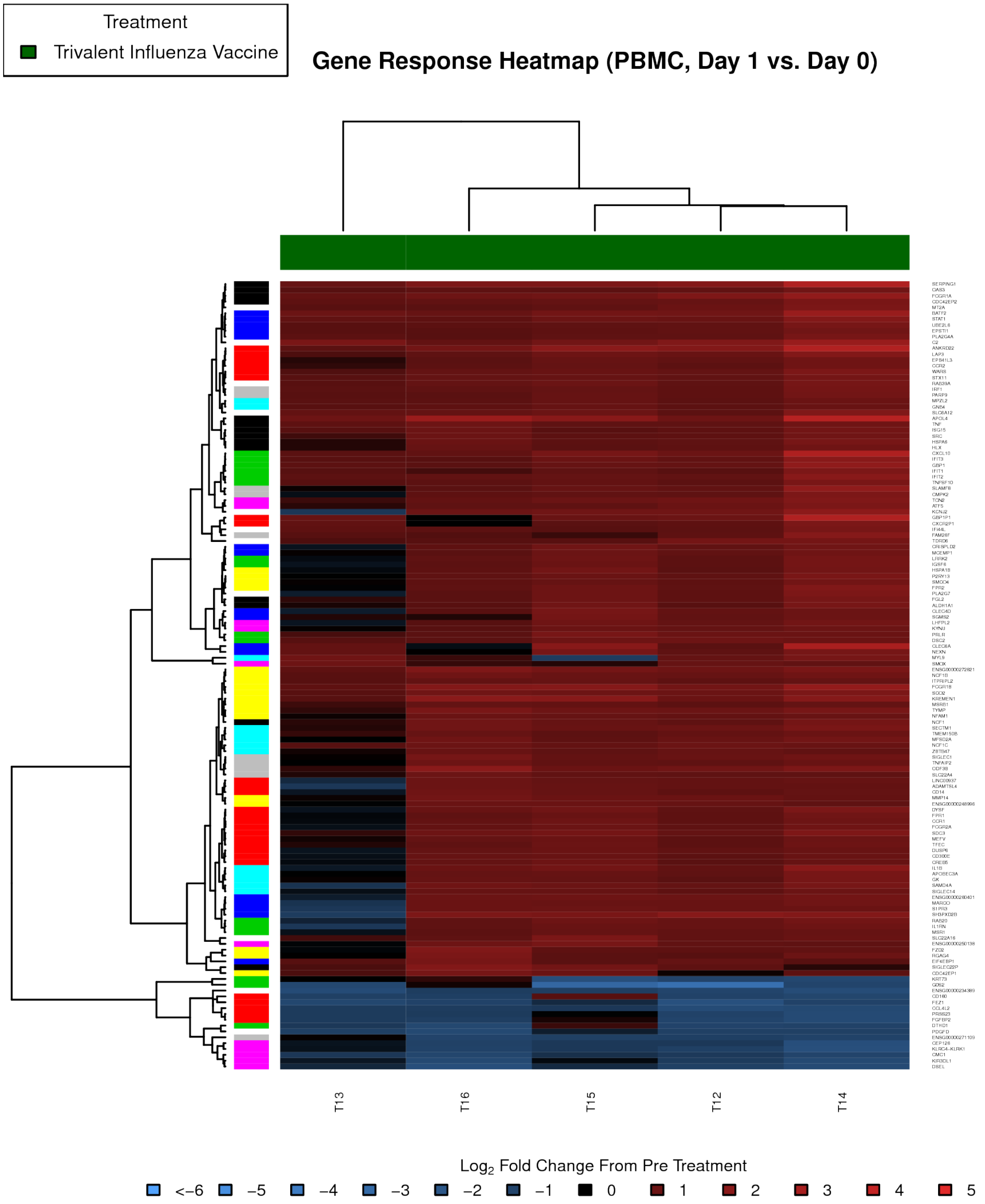

Figure 50: Heatmap of log_2 fold change from pre-treatment (PBMC, Day 1). Rows represent DE genes across treatment types, columns represent samples. In red: up regulated compared to pre-treatment; in green: down-regulated compared to pre-treatment. Dendrograms were obtained using complete linkage clustering of uncentered pairwise Pearson correlation distances for log_2 fold changes. Samples are color-coded by treatment group (see row below sample dendrogram).

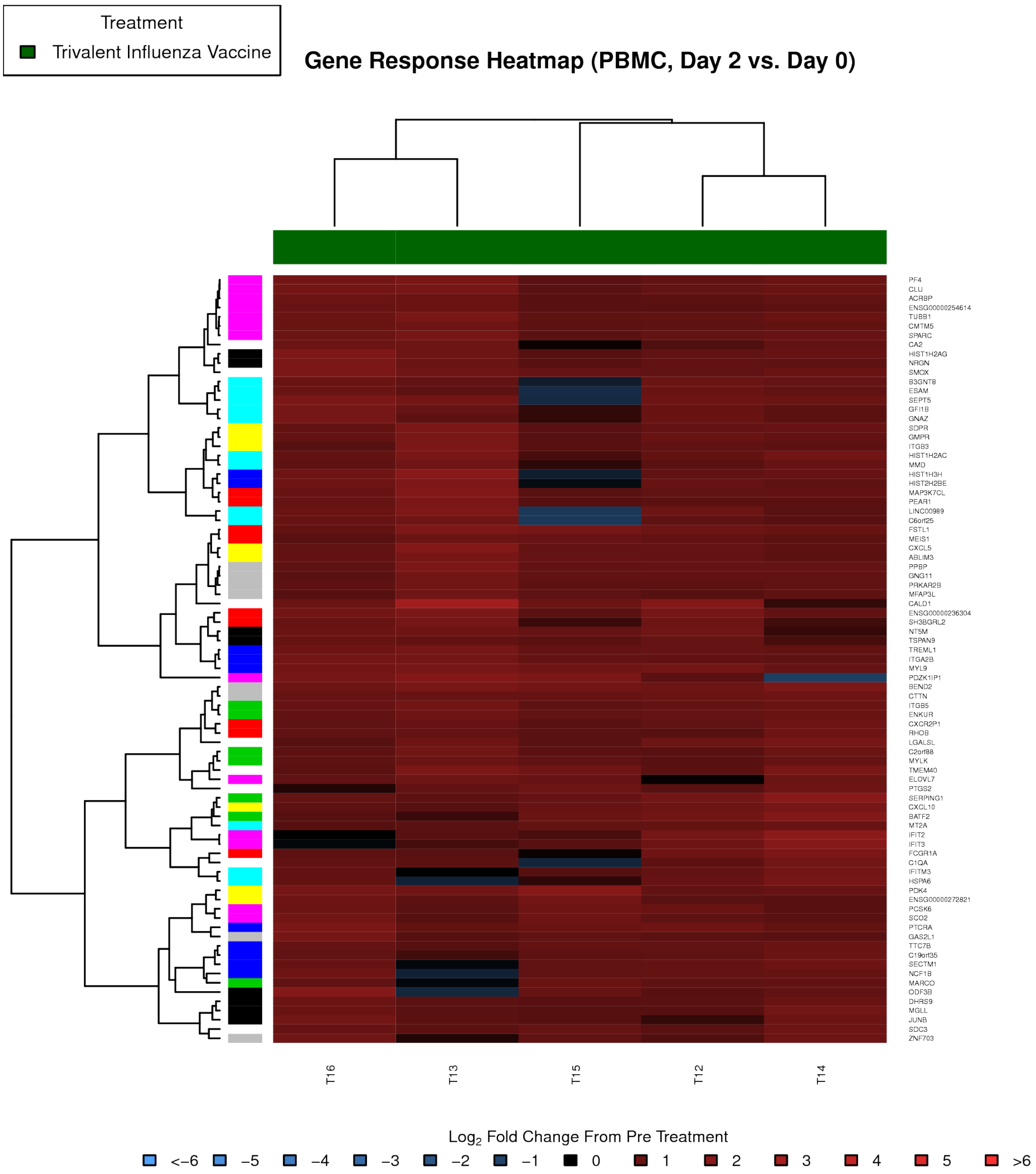

Figure 51: Heatmap of log_2 fold change from pre-treatment (PBMC, Day 2). Rows represent DE genes across treatment types, columns represent samples. In red: up regulated compared to pre-treatment; in green: down-regulated compared to pre-treatment. Dendrograms were obtained using complete linkage clustering of uncentered pairwise Pearson correlation distances for log_2 fold changes. Samples are color-coded by treatment group (see row below sample dendrogram).

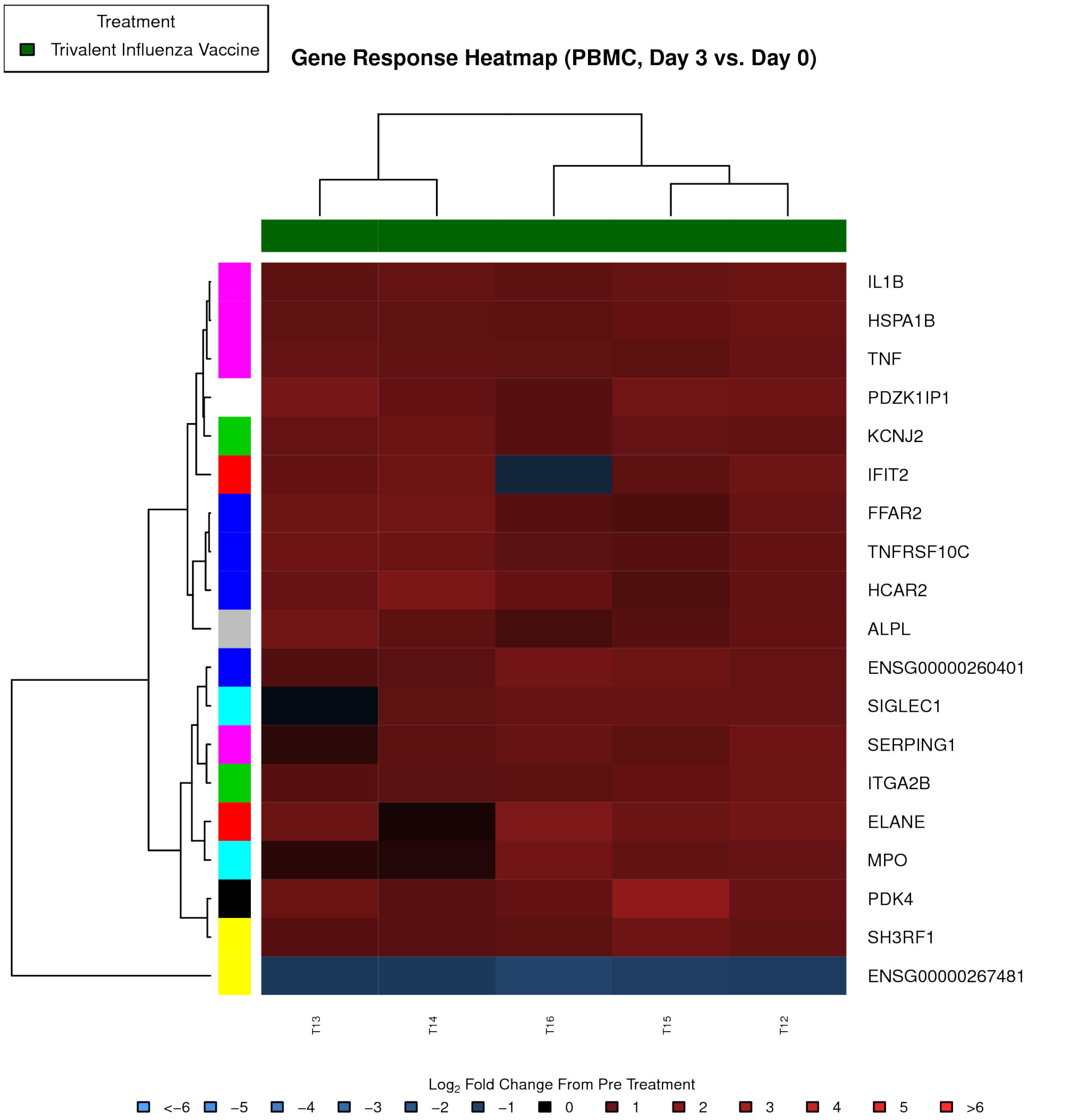

Figure 52: Heatmap of log_2 fold change from pre-treatment (PBMC, Day 3). Rows represent DE genes across treatment types, columns represent samples. In red: up regulated compared to pre-treatment; in green: down-regulated compared to pre-treatment. Dendrograms were obtained using complete linkage clustering of uncentered pairwise Pearson correlation distances for log_2 fold changes. Samples are color-coded by treatment group (see row below sample dendrogram).

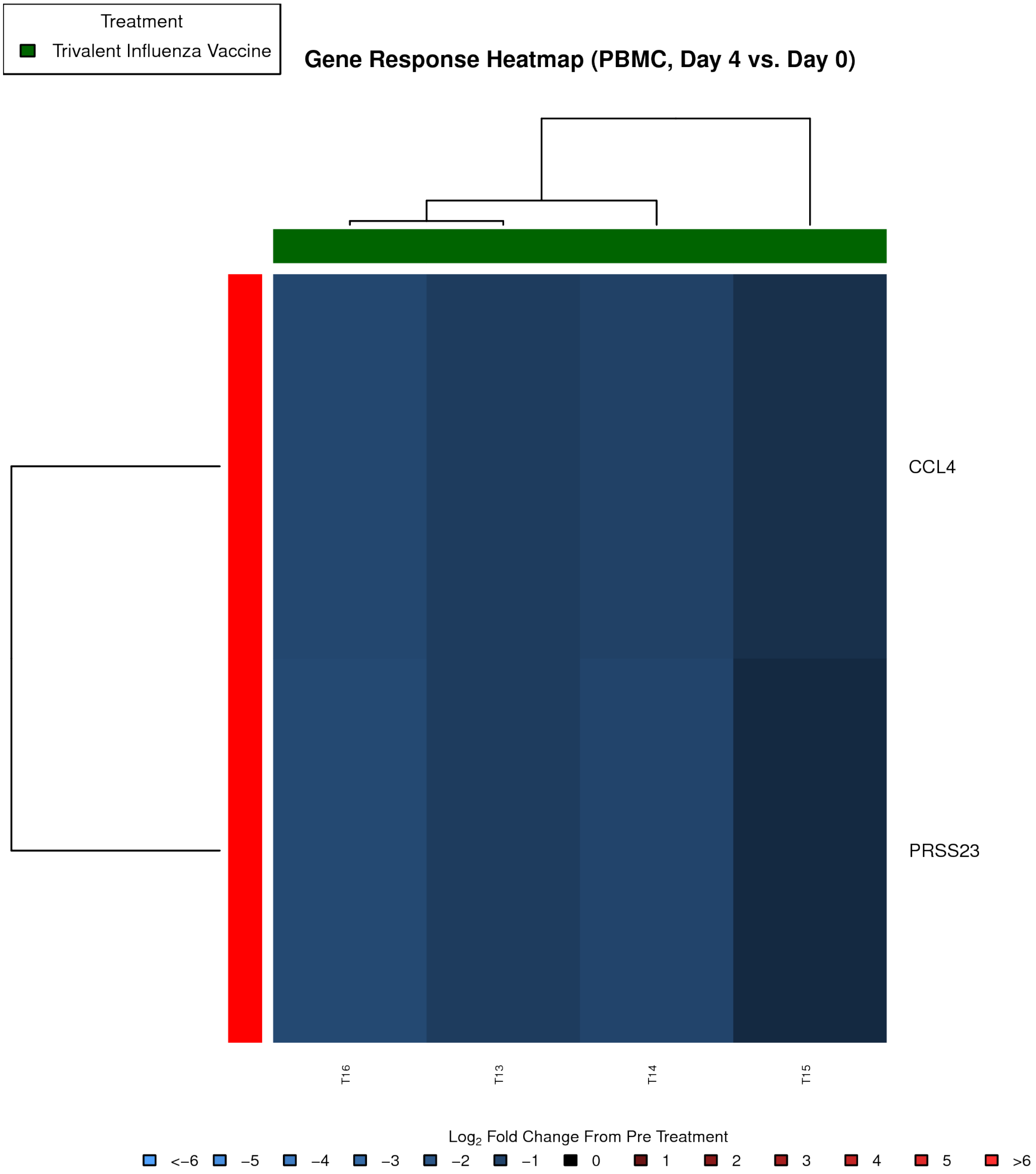

Figure 53: Heatmap of log_2 fold change from pre-treatment (PBMC, Day 4). Rows represent DE genes across treatment types, columns represent samples. In red: up regulated compared to pre-treatment; in green: down-regulated compared to pre-treatment. Dendrograms were obtained using complete linkage clustering of uncentered pairwise Pearson correlation distances for log_2 fold changes. Samples are color-coded by treatment group (see row below sample dendrogram).

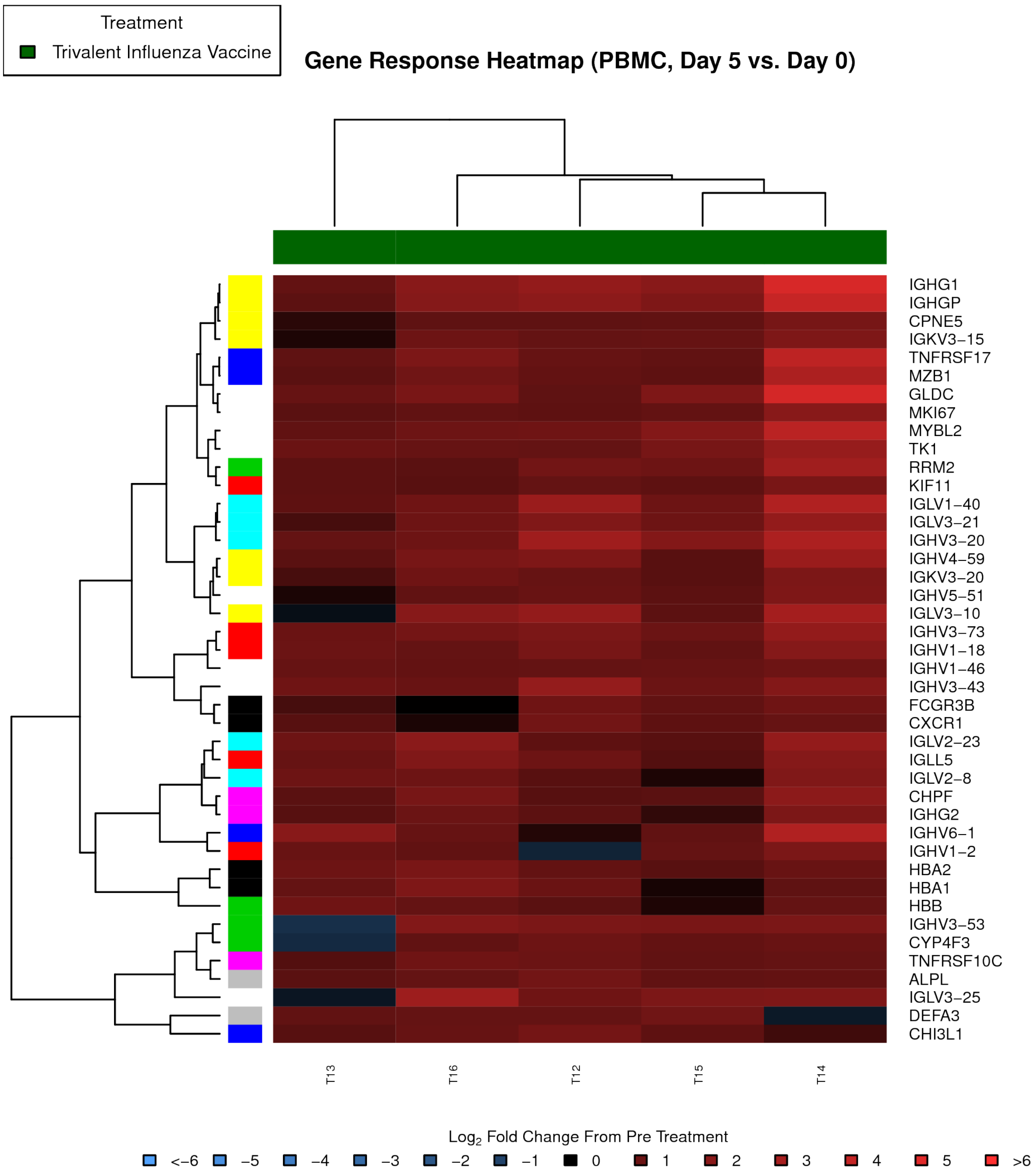

Figure 54: Heatmap of log_2 fold change from pre-treatment (PBMC, Day 5). Rows represent DE genes across treatment types, columns represent samples. In red: up regulated compared to pre-treatment; in green: down-regulated compared to pre-treatment. Dendrograms were obtained using complete linkage clustering of uncentered pairwise Pearson correlation distances for log_2 fold changes. Samples are color-coded by treatment group (see row below sample dendrogram).

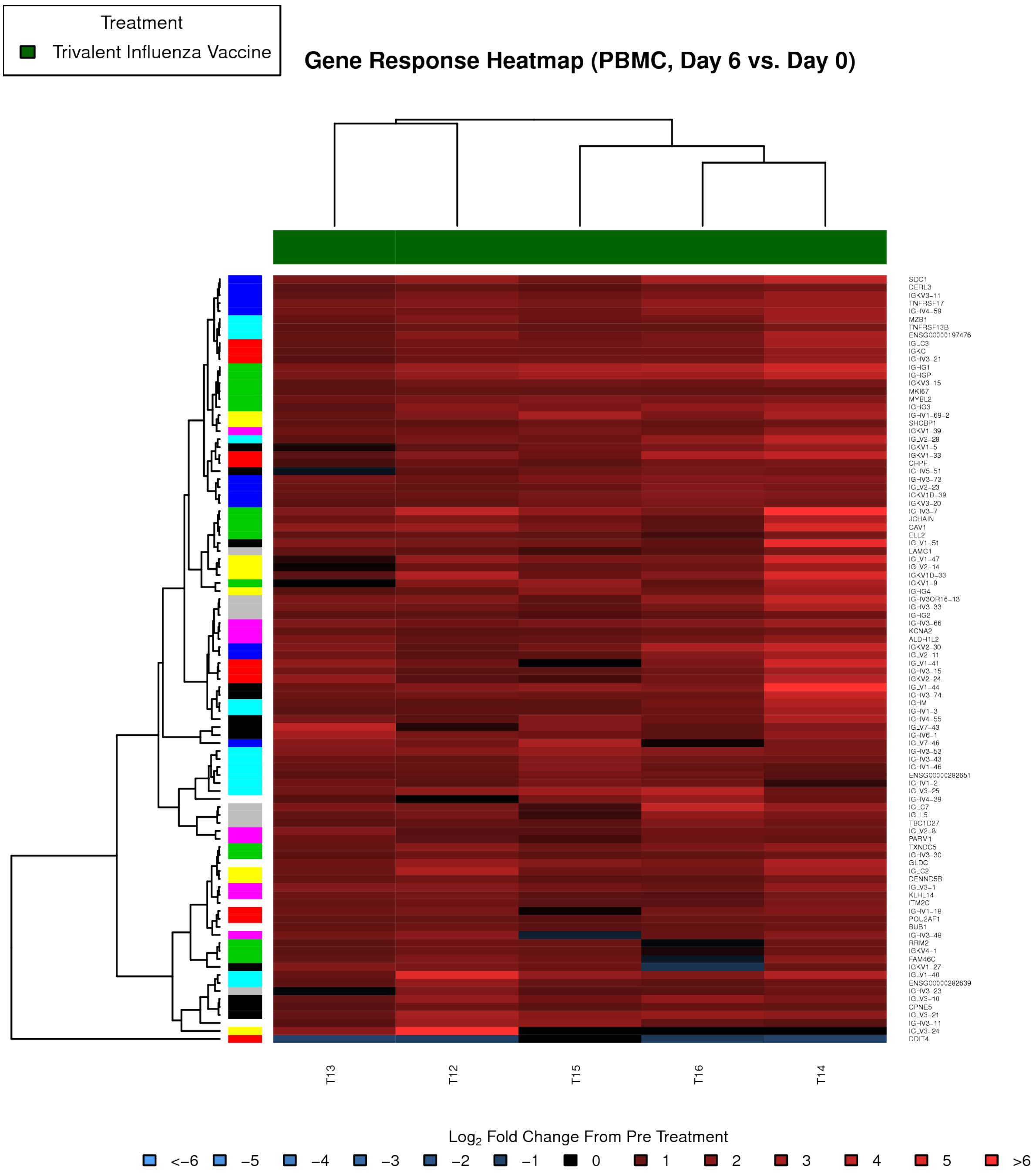

Figure 55: Heatmap of log_2 fold change from pre-treatment (PBMC, Day 6). Rows represent DE genes across treatment types, columns represent samples. In red: up regulated compared to pre-treatment; in green: down-regulated compared to pre-treatment. Dendrograms were obtained using complete linkage clustering of uncentered pairwise Pearson correlation distances for log_2 fold changes. Samples are color-coded by treatment group (see row below sample dendrogram).

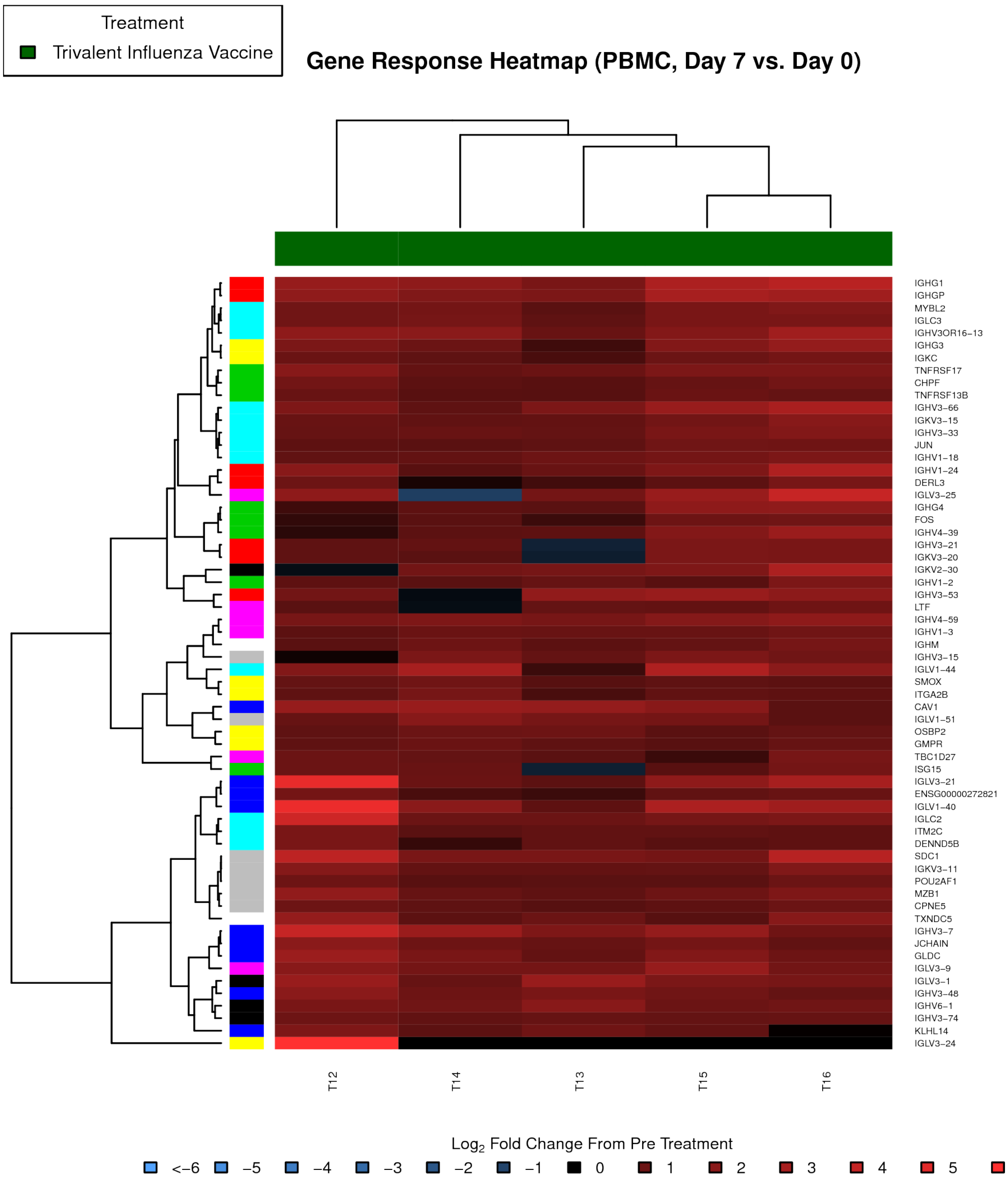

Figure 56: Heatmap of log_2 fold change from pre-treatment (PBMC, Day 7). Rows represent DE genes across treatment types, columns represent samples. In red: up regulated compared to pre-treatment; in green: down-regulated compared to pre-treatment. Dendrograms were obtained using complete linkage clustering of uncentered pairwise Pearson correlation distances for log_2 fold changes. Samples are color-coded by treatment group (see row below sample dendrogram).

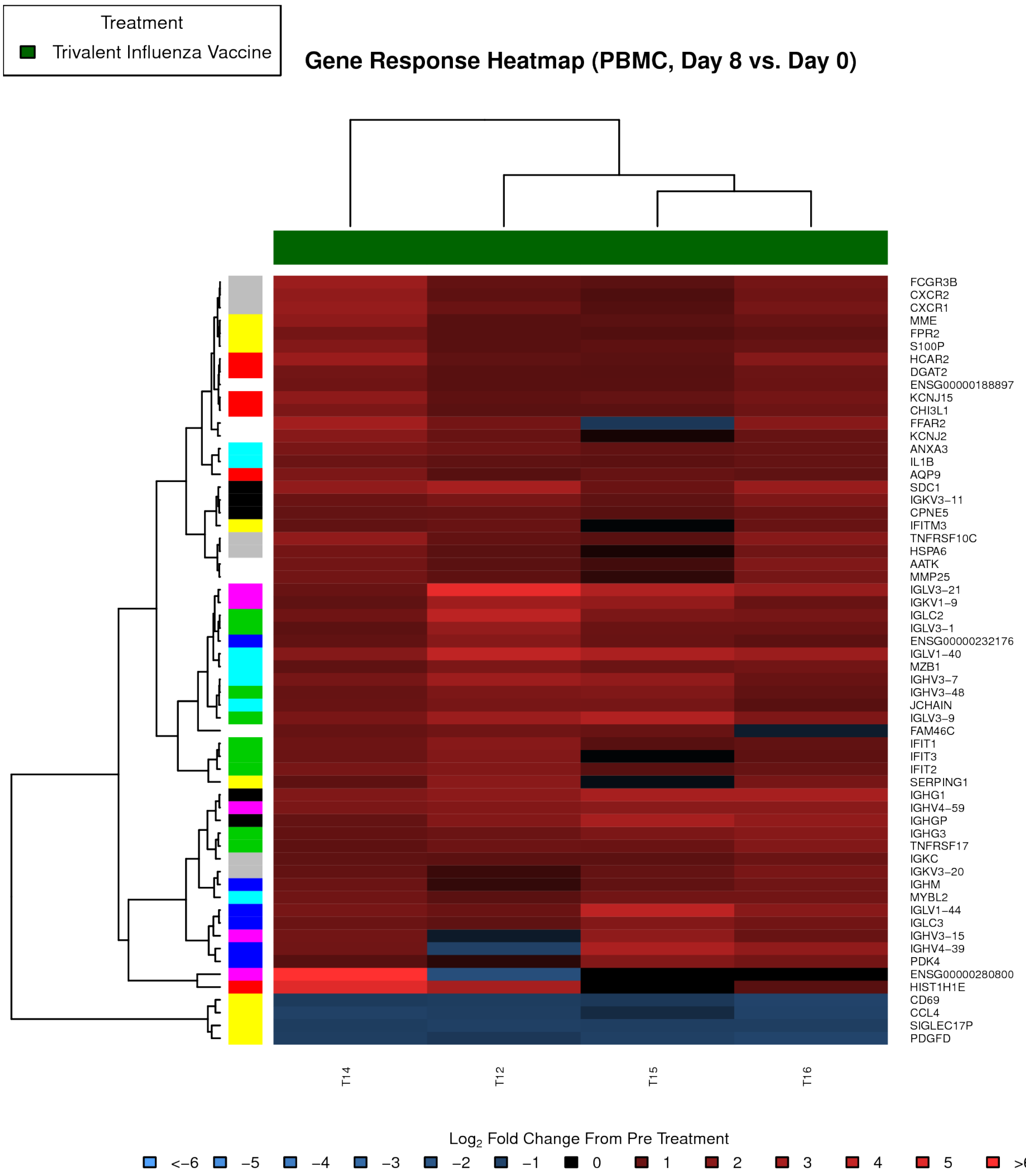

Figure 57: Heatmap of log_2 fold change from pre-treatment (PBMC, Day 8). Rows represent DE genes across treatment types, columns represent samples. In red: up regulated compared to pre-treatment; in green: down-regulated compared to pre-treatment. Dendrograms were obtained using complete linkage clustering of uncentered pairwise Pearson correlation distances for log_2 fold changes. Samples are color-coded by treatment group (see row below sample dendrogram).

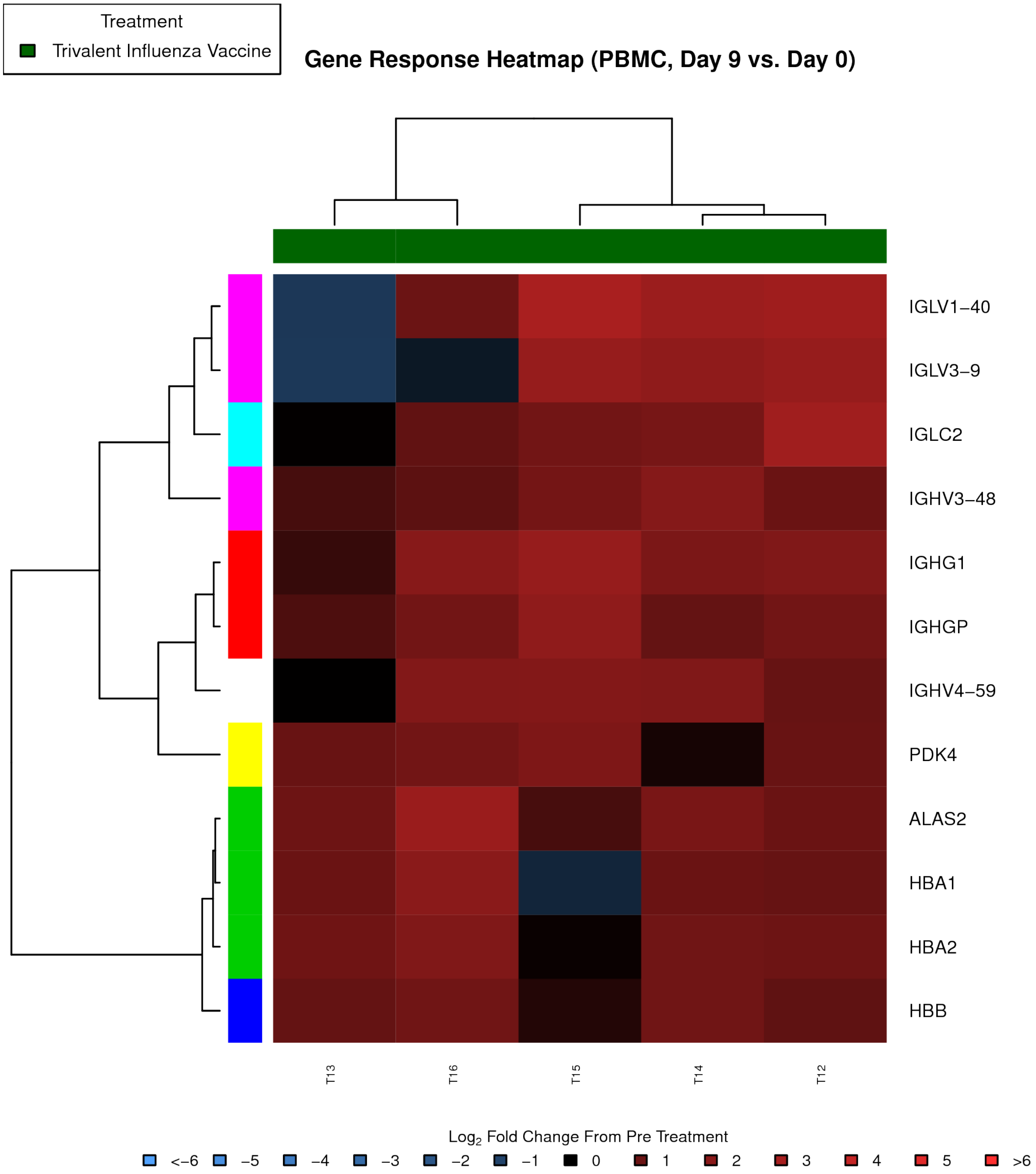

Figure 58: Heatmap of log_2 fold change from pre-treatment (PBMC, Day 9). Rows represent DE genes across treatment types, columns represent samples. In red: up regulated compared to pre-treatment; in green: down-regulated compared to pre-treatment. Dendrograms were obtained using complete linkage clustering of uncentered pairwise Pearson correlation distances for log_2 fold changes. Samples are color-coded by treatment group (see row below sample dendrogram).

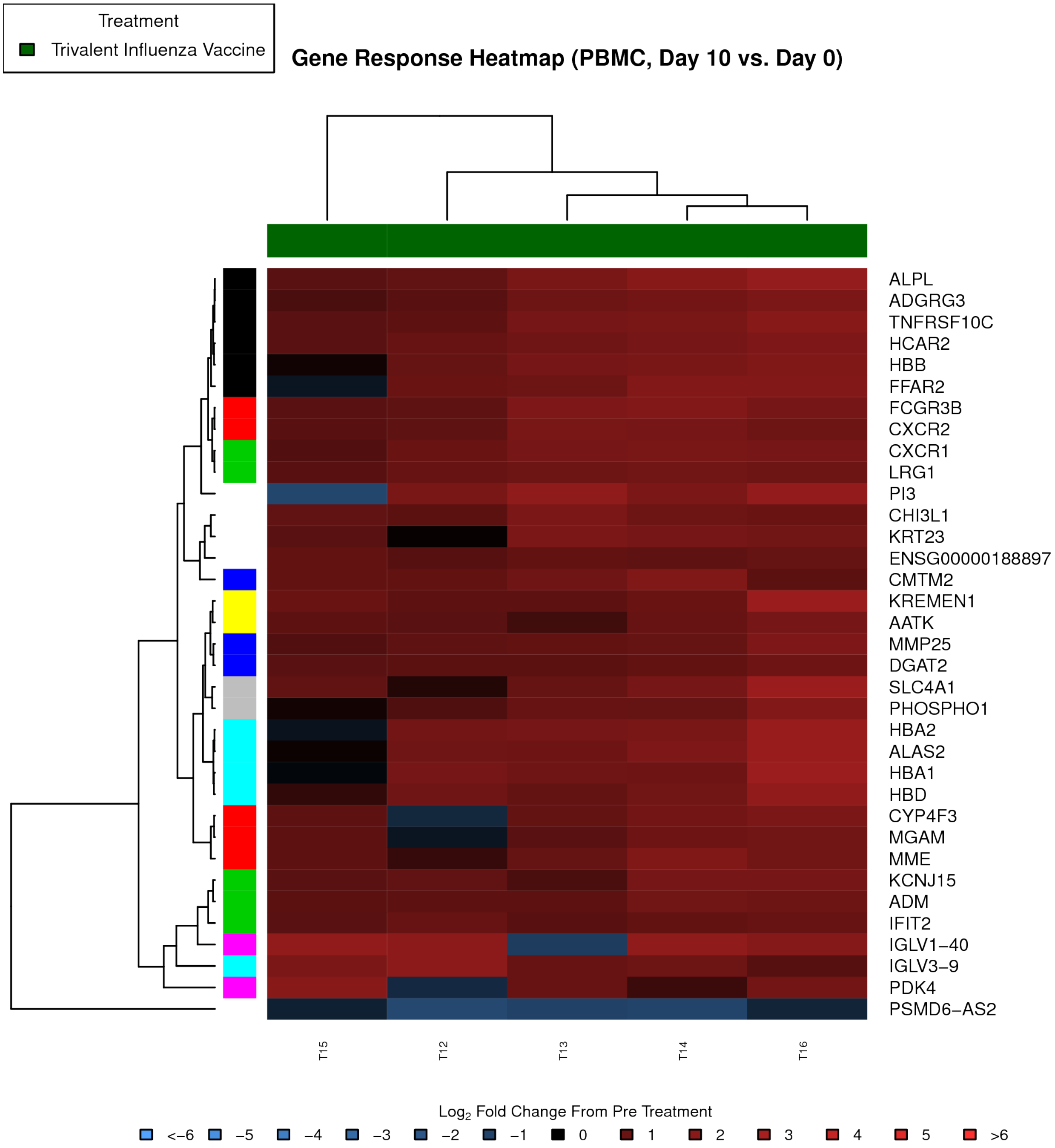

Figure 59: Heatmap of log_2 fold change from pre-treatment (PBMC, Day 10). Rows represent DE genes across treatment types, columns represent samples. In red: up regulated compared to pre-treatment; in green: down-regulated compared to pre-treatment. Dendrograms were obtained using complete linkage clustering of uncentered pairwise Pearson correlation distances for log_2 fold changes. Samples are color-coded by treatment group (see row below sample dendrogram).

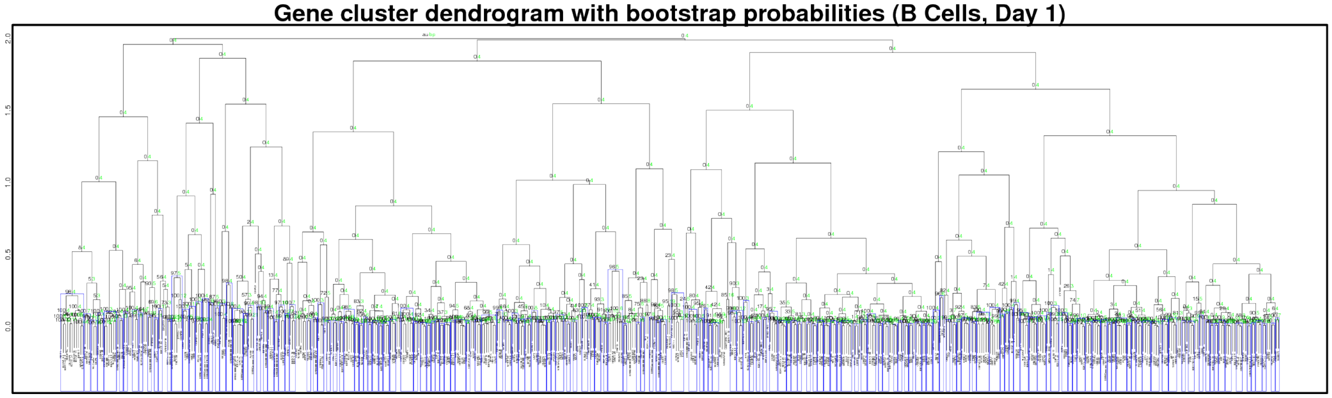

Figure 60: Co-expressed gene cluster dendrogram with bootstrap probabilities (B Cells, Day 1). The y axis shows the distance at which clusters were formed by the complete linkage clustering algorithm based on uncentered Pearson correlation distance between log_2 fold changes. Multiscale bootstrap probabilities are shown at each branch intersection. Significant clusters are highlighted in blue. Asterisks indicate genes that were significantly expressed at a certain day.

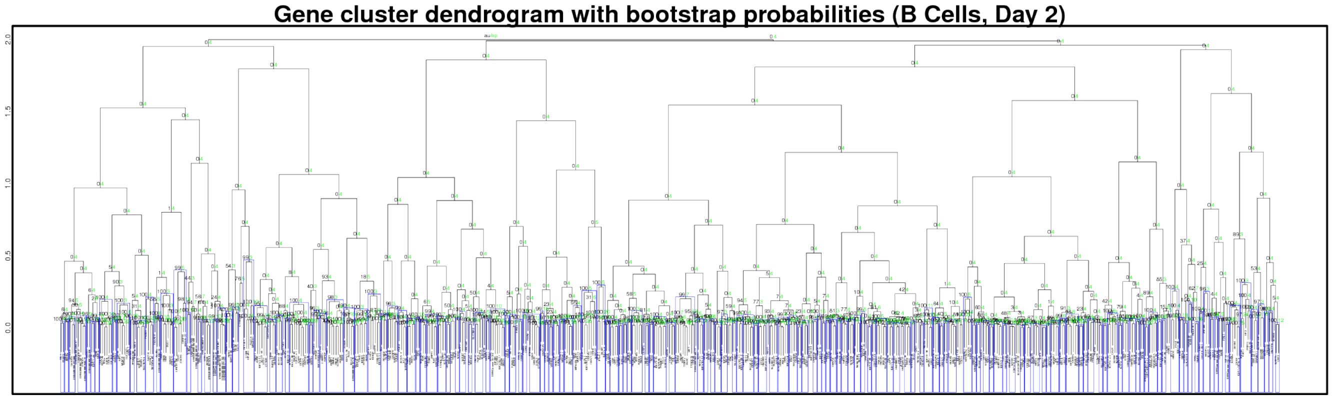

Figure 61: Co-expressed gene cluster dendrogram with bootstrap probabilities (B Cells, Day 2). The y axis shows the distance at which clusters were formed by the complete linkage clustering algorithm based on uncentered Pearson correlation distance between log_2 fold changes. Multiscale bootstrap probabilities are shown at each branch intersection. Significant clusters are highlighted in blue. Asterisks indicate genes that were significantly expressed at a certain day.

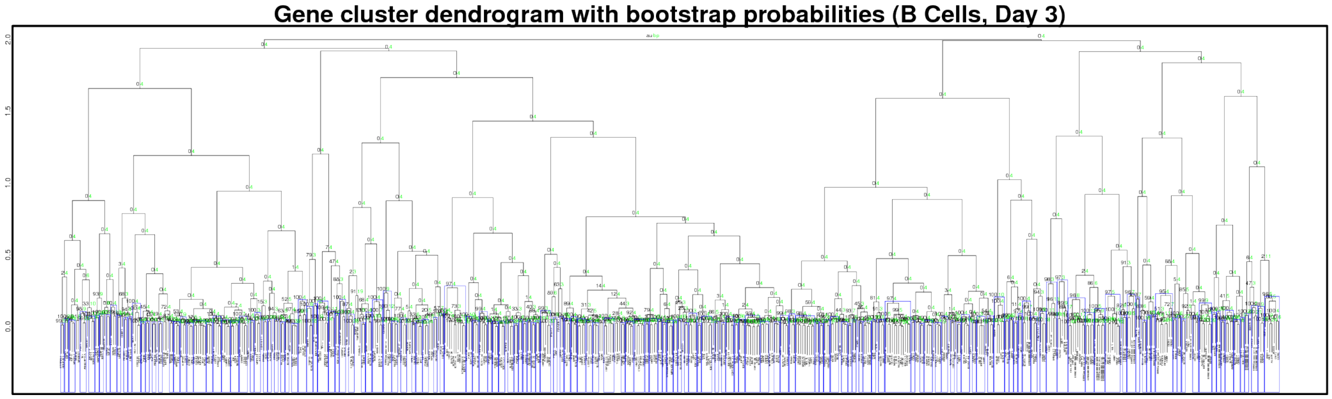

Figure 62: Co-expressed gene cluster dendrogram with bootstrap probabilities (B Cells, Day 3). The y axis shows the distance at which clusters were formed by the complete linkage clustering algorithm based on uncentered Pearson correlation distance between log_2 fold changes. Multiscale bootstrap probabilities are shown at each branch intersection. Significant clusters are highlighted in blue. Asterisks indicate genes that were significantly expressed at a certain day.

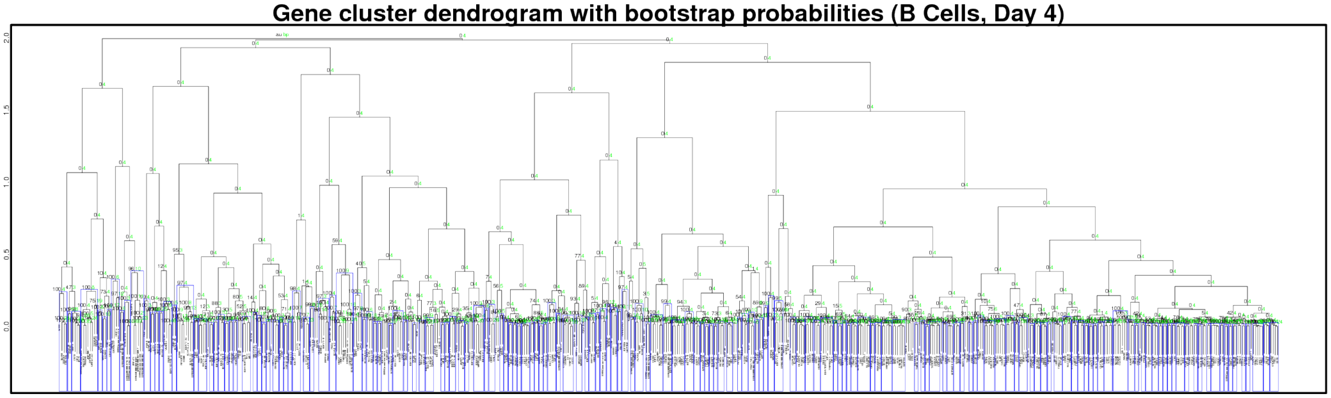

Figure 63: Co-expressed gene cluster dendrogram with bootstrap probabilities (B Cells, Day 4). The y axis shows the distance at which clusters were formed by the complete linkage clustering algorithm based on uncentered Pearson correlation distance between log_2 fold changes. Multiscale bootstrap probabilities are shown at each branch intersection. Significant clusters are highlighted in blue. Asterisks indicate genes that were significantly expressed at a certain day.

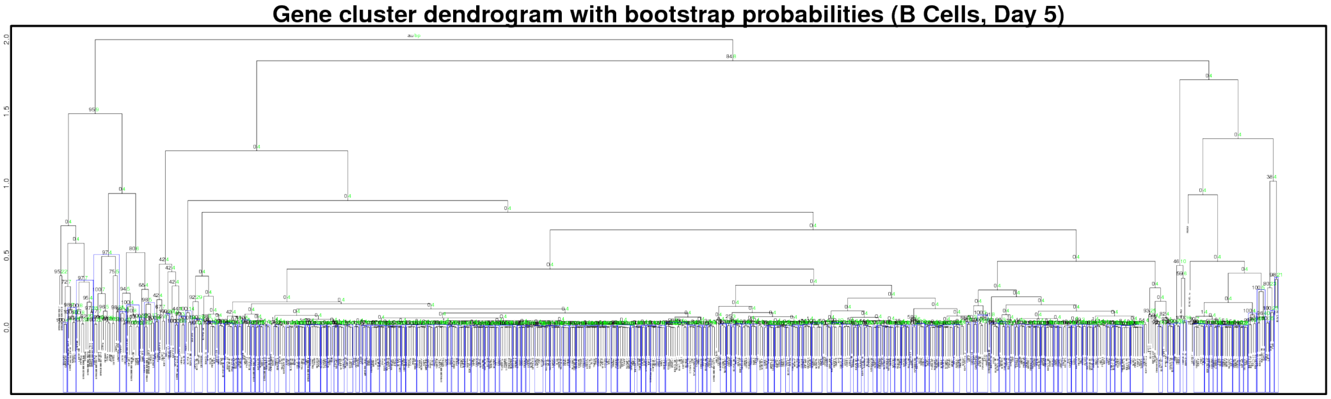

Figure 64: Co-expressed gene cluster dendrogram with bootstrap probabilities (B Cells, Day 5). The y axis shows the distance at which clusters were formed by the complete linkage clustering algorithm based on uncentered Pearson correlation distance between log_2 fold changes. Multiscale bootstrap probabilities are shown at each branch intersection. Significant clusters are highlighted in blue. Asterisks indicate genes that were significantly expressed at a certain day.

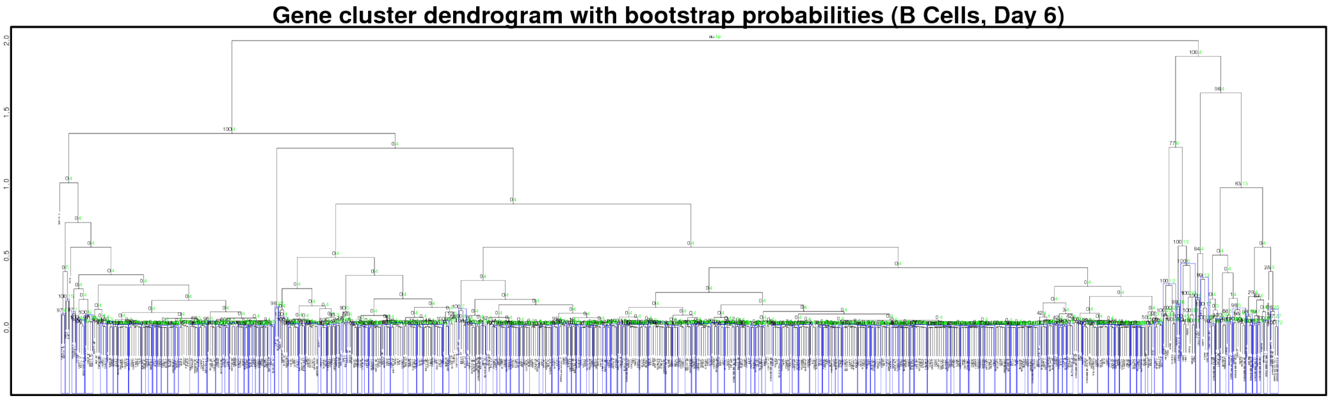

Figure 65: Co-expressed gene cluster dendrogram with bootstrap probabilities (B Cells, Day 6). The y axis shows the distance at which clusters were formed by the complete linkage clustering algorithm based on uncentered Pearson correlation distance between log_2 fold changes. Multiscale bootstrap probabilities are shown at each branch intersection. Significant clusters are highlighted in blue. Asterisks indicate genes that were significantly expressed at a certain day.

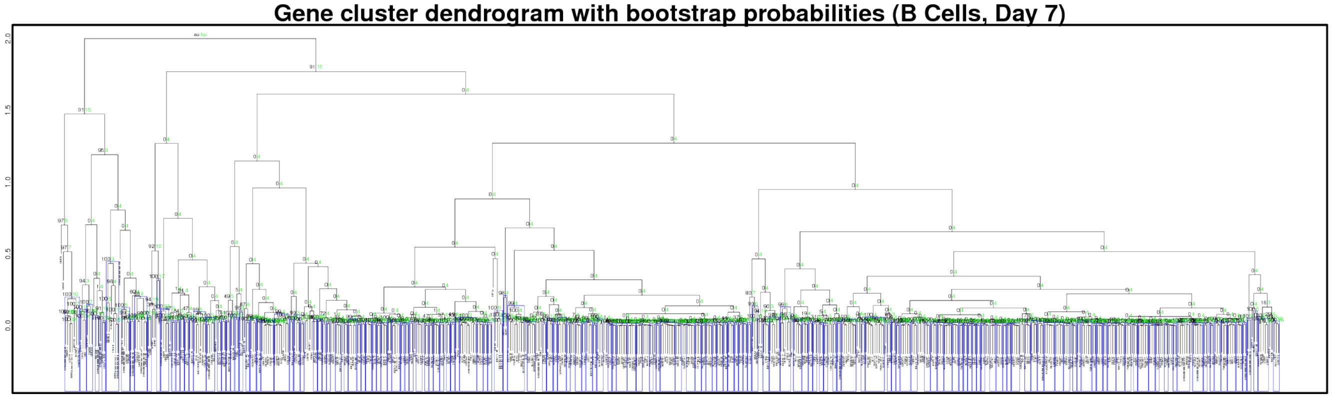

Figure 66: Co-expressed gene cluster dendrogram with bootstrap probabilities (B Cells, Day 7). The y axis shows the distance at which clusters were formed by the complete linkage clustering algorithm based on uncentered Pearson correlation distance between log_2 fold changes. Multiscale bootstrap probabilities are shown at each branch intersection. Significant clusters are highlighted in blue. Asterisks indicate genes that were significantly expressed at a certain day.

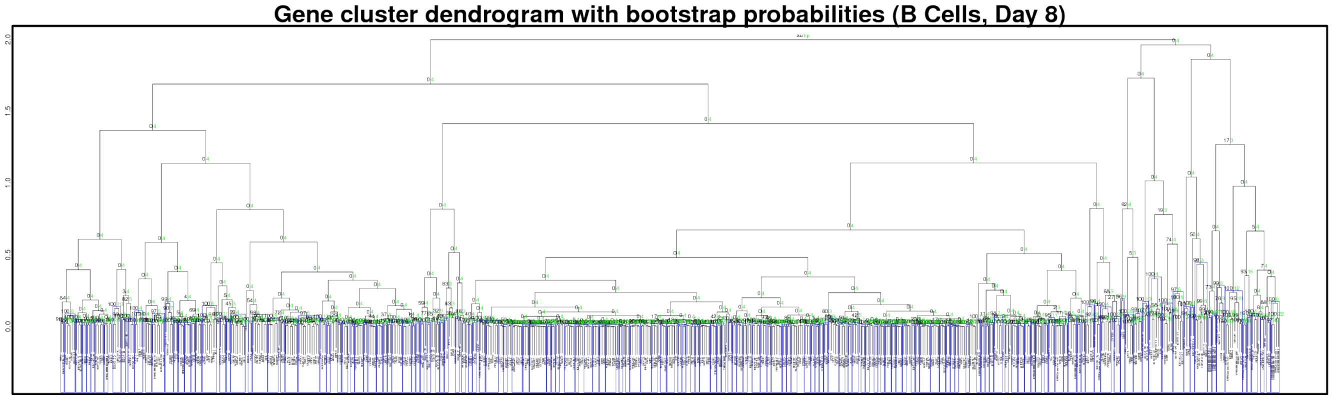

Figure 67: Co-expressed gene cluster dendrogram with bootstrap probabilities (B Cells, Day 8). The y axis shows the distance at which clusters were formed by the complete linkage clustering algorithm based on uncentered Pearson correlation distance between log_2 fold changes. Multiscale bootstrap probabilities are shown at each branch intersection. Significant clusters are highlighted in blue. Asterisks indicate genes that were significantly expressed at a certain day.

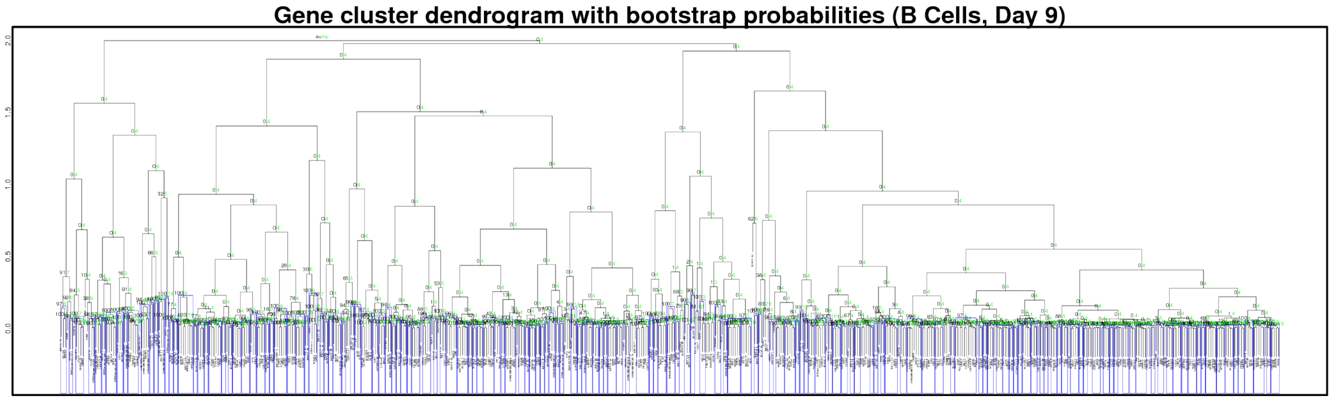

Figure 68: Co-expressed gene cluster dendrogram with bootstrap probabilities (B Cells, Day 9). The y axis shows the distance at which clusters were formed by the complete linkage clustering algorithm based on uncentered Pearson correlation distance between log_2 fold changes. Multiscale bootstrap probabilities are shown at each branch intersection. Significant clusters are highlighted in blue. Asterisks indicate genes that were significantly expressed at a certain day.

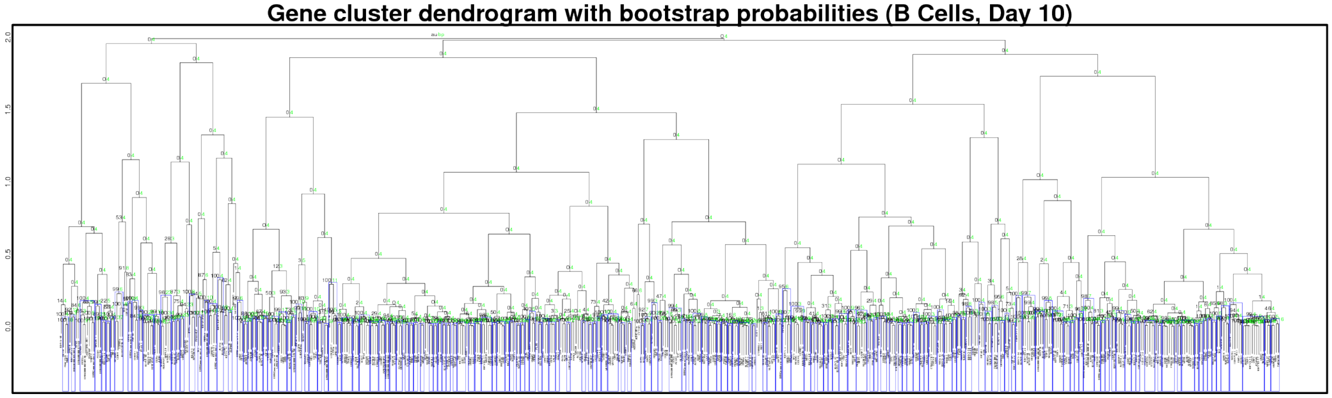

Figure 69: Co-expressed gene cluster dendrogram with bootstrap probabilities (B Cells, Day 10). The y axis shows the distance at which clusters were formed by the complete linkage clustering algorithm based on uncentered Pearson correlation distance between log_2 fold changes. Multiscale bootstrap probabilities are shown at each branch intersection. Significant clusters are highlighted in blue. Asterisks indicate genes that were significantly expressed at a certain day.

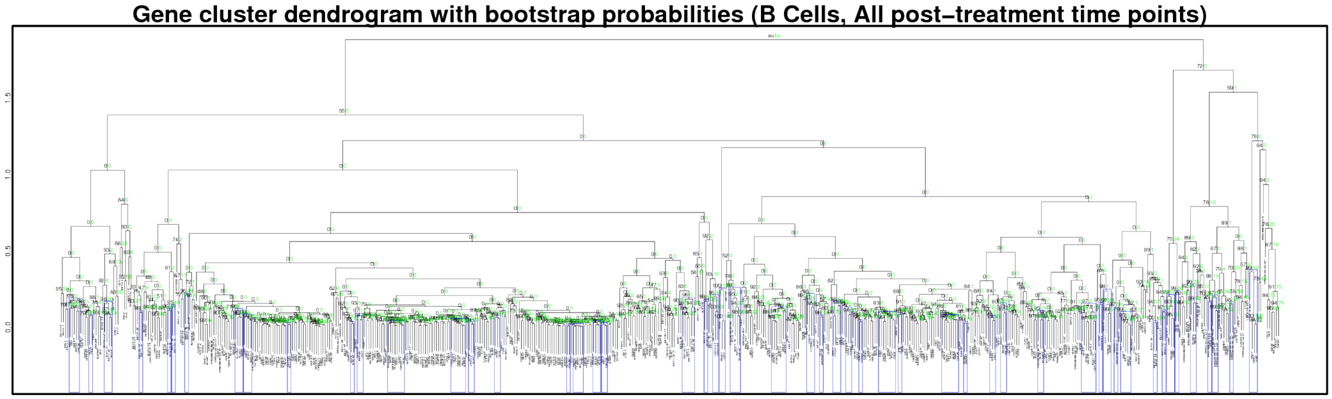

Figure 70: Co-expressed gene cluster dendrogram with bootstrap probabilities (B Cells, All post-treatment time points). The y axis shows the distance at which clusters were formed by the complete linkage clustering algorithm based on uncentered Pearson correlation distance between log_2 fold changes. Multiscale bootstrap probabilities are shown at each branch intersection. Significant clusters are highlighted in blue. Asterisks indicate genes that were significantly expressed at a certain day.

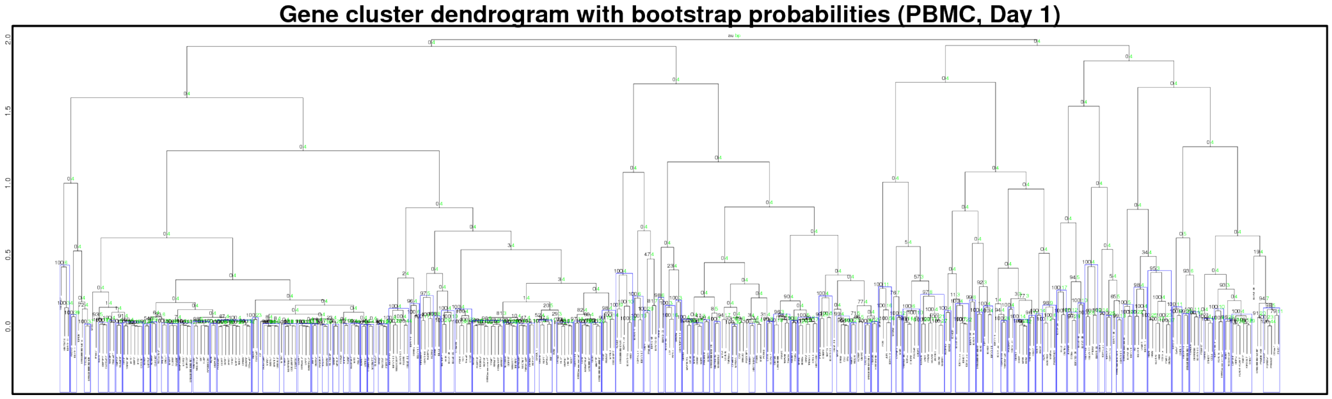

Figure 71: Co-expressed gene cluster dendrogram with bootstrap probabilities (PBMC, Day 1). The y axis shows the distance at which clusters were formed by the complete linkage clustering algorithm based on uncentered Pearson correlation distance between log_2 fold changes. Multiscale bootstrap probabilities are shown at each branch intersection. Significant clusters are highlighted in blue. Asterisks indicate genes that were significantly expressed at a certain day.

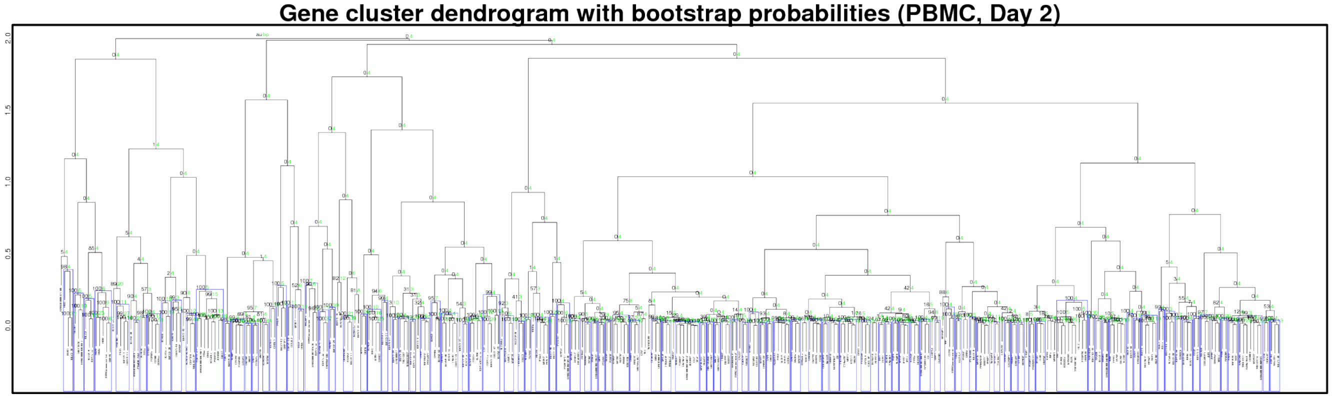

Figure 72: Co-expressed gene cluster dendrogram with bootstrap probabilities (PBMC, Day 2). The y axis shows the distance at which clusters were formed by the complete linkage clustering algorithm based on uncentered Pearson correlation distance between log_2 fold changes. Multiscale bootstrap probabilities are shown at each branch intersection. Significant clusters are highlighted in blue. Asterisks indicate genes that were significantly expressed at a certain day.

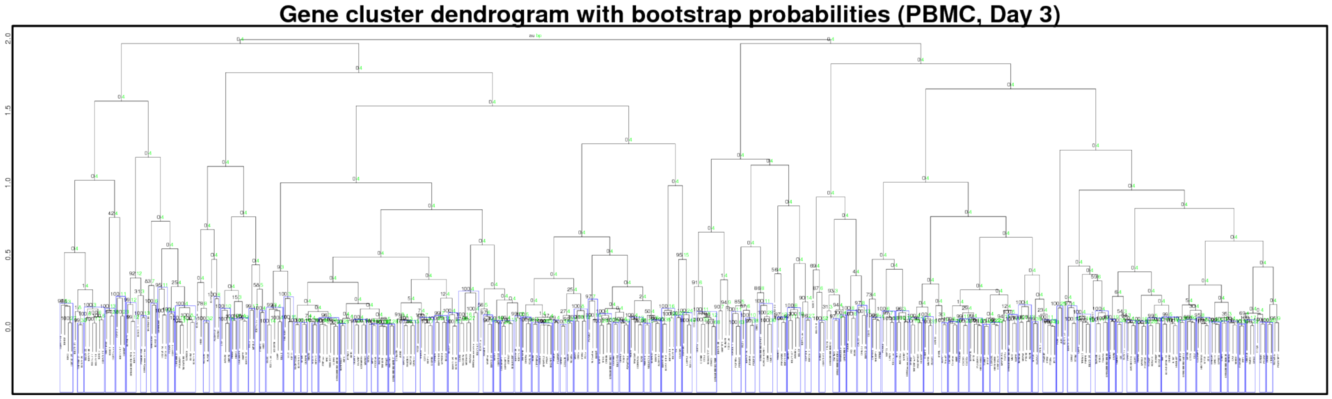

Figure 73: Co-expressed gene cluster dendrogram with bootstrap probabilities (PBMC, Day 3). The y axis shows the distance at which clusters were formed by the complete linkage clustering algorithm based on uncentered Pearson correlation distance between log_2 fold changes. Multiscale bootstrap probabilities are shown at each branch intersection. Significant clusters are highlighted in blue. Asterisks indicate genes that were significantly expressed at a certain day.

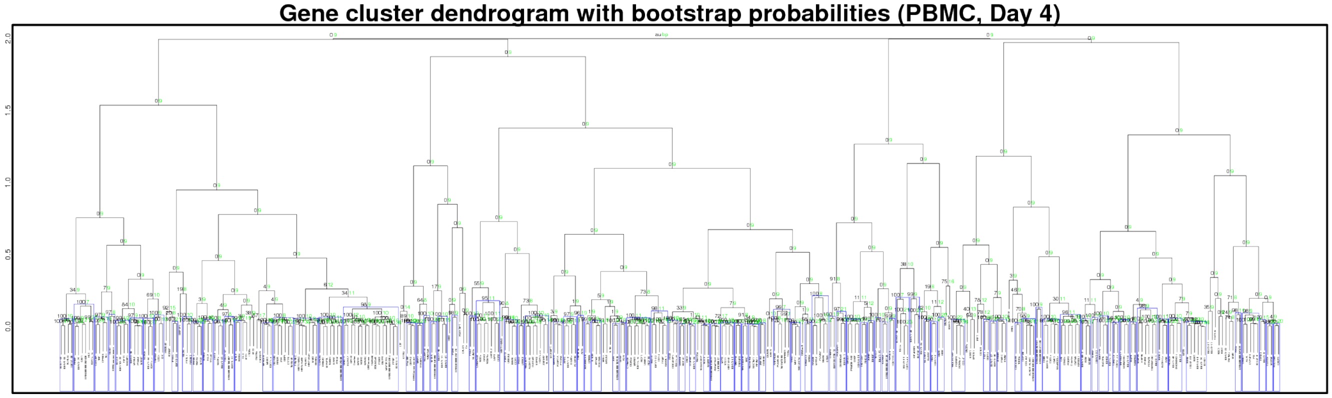

Figure 74: Co-expressed gene cluster dendrogram with bootstrap probabilities (PBMC, Day 4). The y axis shows the distance at which clusters were formed by the complete linkage clustering algorithm based on uncentered Pearson correlation distance between log_2 fold changes. Multiscale bootstrap probabilities are shown at each branch intersection. Significant clusters are highlighted in blue. Asterisks indicate genes that were significantly expressed at a certain day.

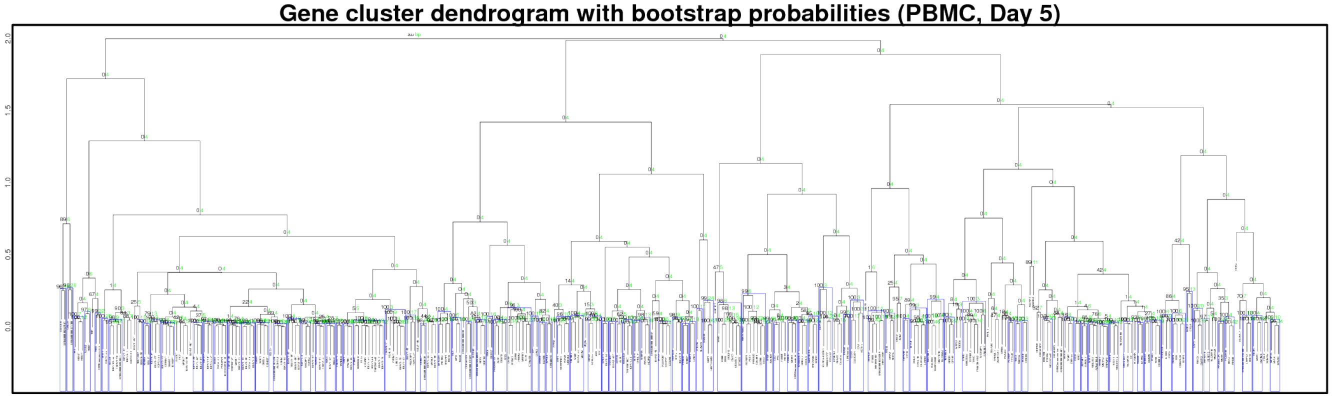

Figure 75: Co-expressed gene cluster dendrogram with bootstrap probabilities (PBMC, Day 5). The y axis shows the distance at which clusters were formed by the complete linkage clustering algorithm based on uncentered Pearson correlation distance between log_2 fold changes. Multiscale bootstrap probabilities are shown at each branch intersection. Significant clusters are highlighted in blue. Asterisks indicate genes that were significantly expressed at a certain day.

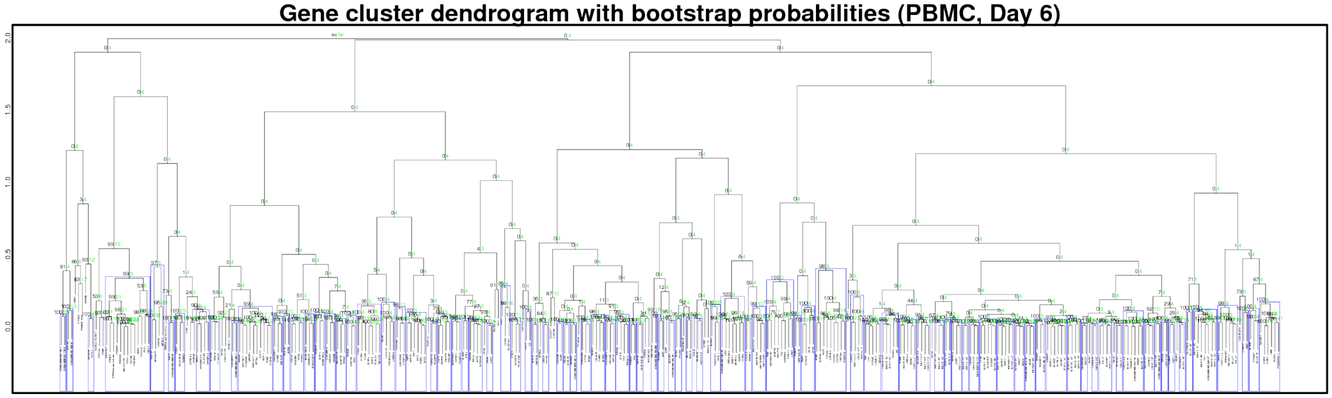

Figure 76: Co-expressed gene cluster dendrogram with bootstrap probabilities (PBMC, Day 6). The y axis shows the distance at which clusters were formed by the complete linkage clustering algorithm based on uncentered Pearson correlation distance between log_2 fold changes. Multiscale bootstrap probabilities are shown at each branch intersection. Significant clusters are highlighted in blue. Asterisks indicate genes that were significantly expressed at a certain day.

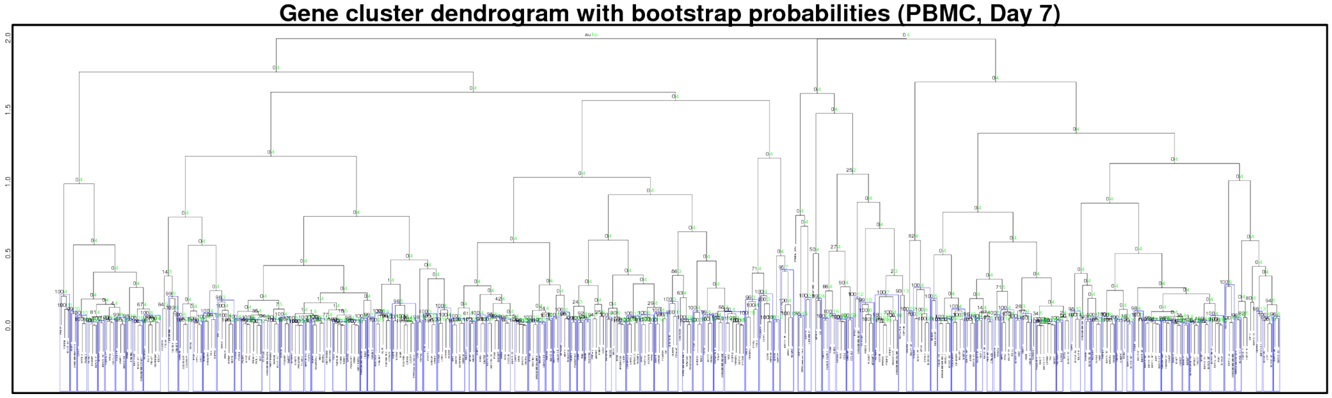

Figure 77: Co-expressed gene cluster dendrogram with bootstrap probabilities (PBMC, Day 7). The y axis shows the distance at which clusters were formed by the complete linkage clustering algorithm based on uncentered Pearson correlation distance between log_2 fold changes. Multiscale bootstrap probabilities are shown at each branch intersection. Significant clusters are highlighted in blue. Asterisks indicate genes that were significantly expressed at a certain day.

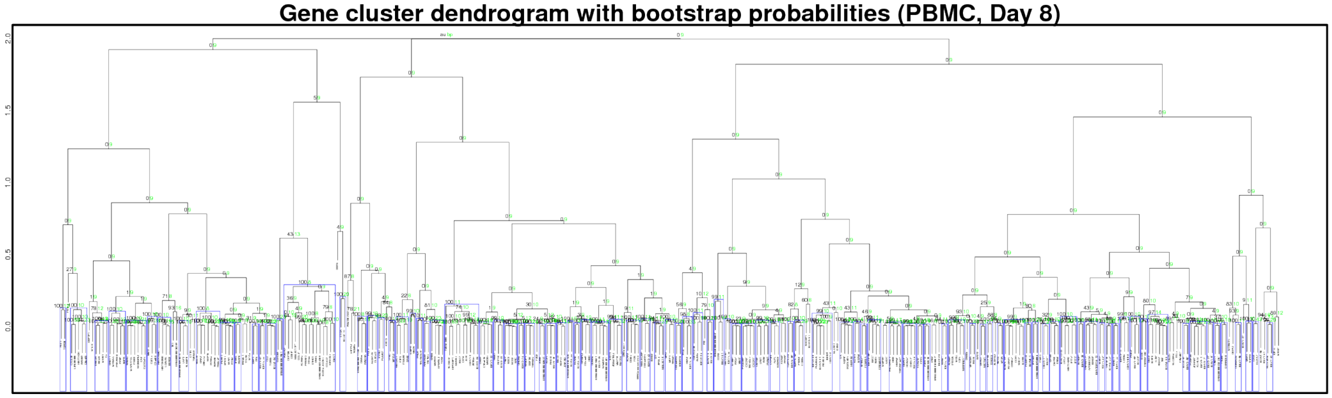

Figure 78: Co-expressed gene cluster dendrogram with bootstrap probabilities (PBMC, Day 8). The y axis shows the distance at which clusters were formed by the complete linkage clustering algorithm based on uncentered Pearson correlation distance between log_2 fold changes. Multiscale bootstrap probabilities are shown at each branch intersection. Significant clusters are highlighted in blue. Asterisks indicate genes that were significantly expressed at a certain day.

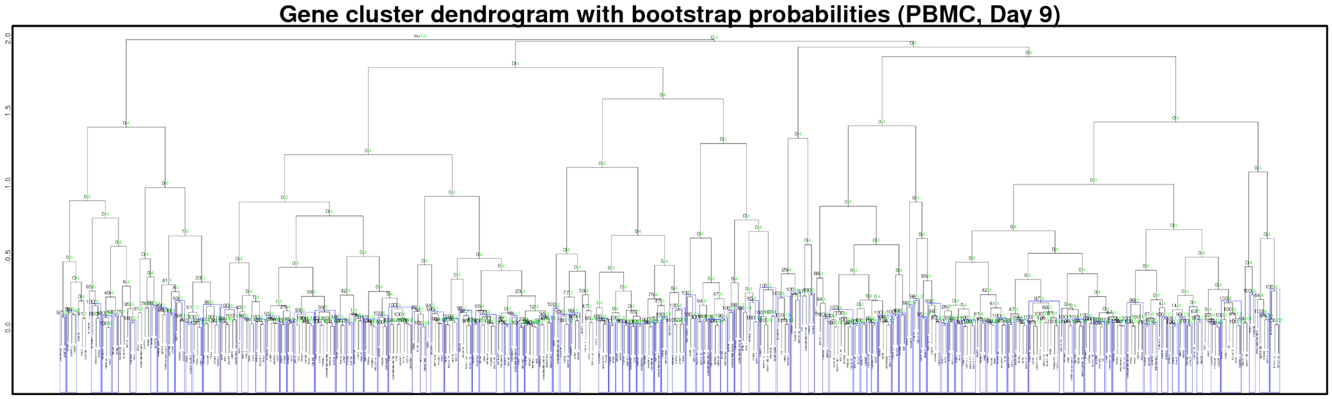

Figure 79: Co-expressed gene cluster dendrogram with bootstrap probabilities (PBMC, Day 9). The y axis shows the distance at which clusters were formed by the complete linkage clustering algorithm based on uncentered Pearson correlation distance between log_2 fold changes. Multiscale bootstrap probabilities are shown at each branch intersection. Significant clusters are highlighted in blue. Asterisks indicate genes that were significantly expressed at a certain day.

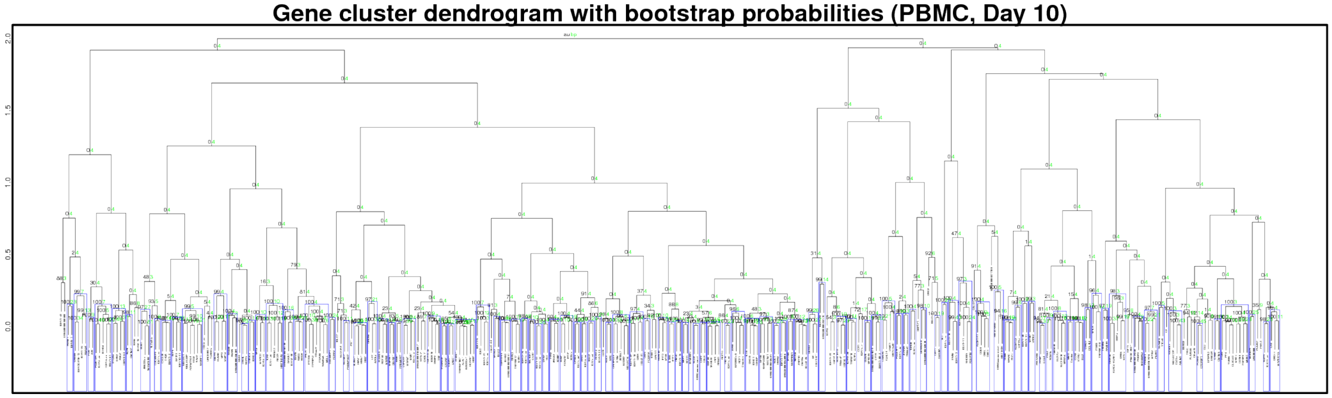

Figure 80: Co-expressed gene cluster dendrogram with bootstrap probabilities (PBMC, Day 10). The y axis shows the distance at which clusters were formed by the complete linkage clustering algorithm based on uncentered Pearson correlation distance between log_2 fold changes. Multiscale bootstrap probabilities are shown at each branch intersection. Significant clusters are highlighted in blue. Asterisks indicate genes that were significantly expressed at a certain day.

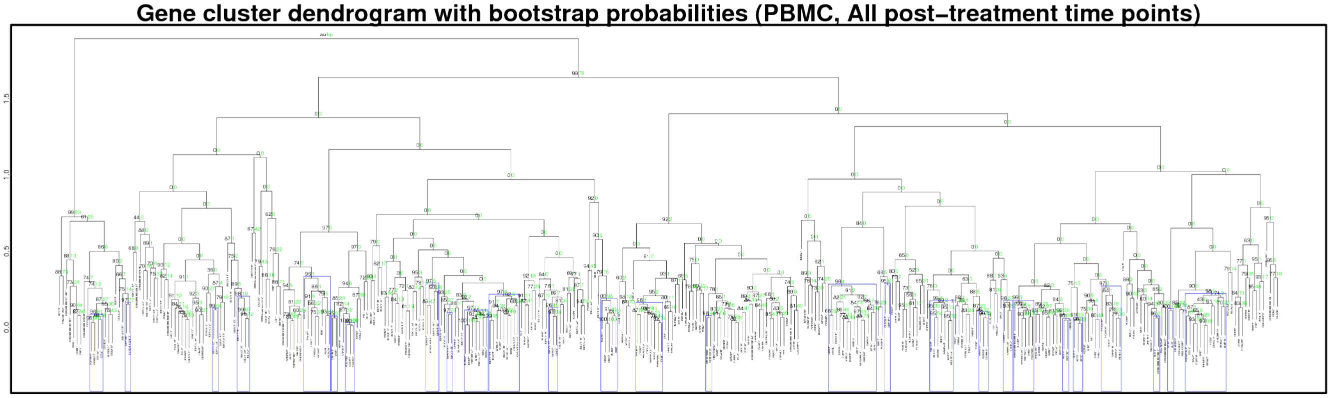

Figure 81: Co-expressed gene cluster dendrogram with bootstrap probabilities (PBMC, All post-treatment time points). The y axis shows the distance at which clusters were formed by the complete linkage clustering algorithm based on uncentered Pearson correlation distance between log_2 fold changes. Multiscale bootstrap probabilities are shown at each branch intersection. Significant clusters are highlighted in blue. Asterisks indicate genes that were significantly expressed at a certain day.

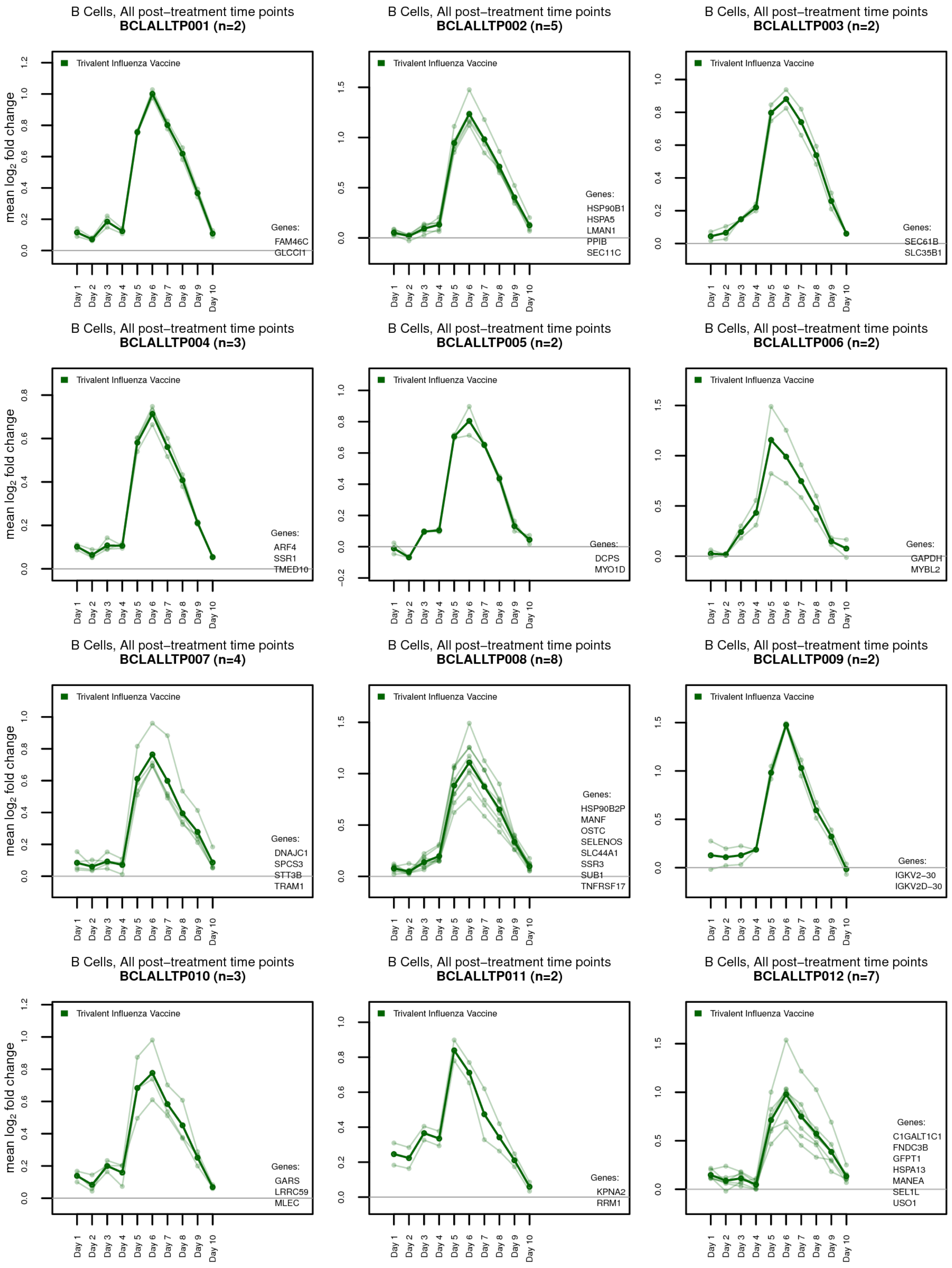

Figure 82: Co-expressed gene cluster time trends of log_2 fold change from pre-treatment levels by treatment 1 of 5 (B Cells). Header indicates cluster ID. Mean log_2 fold change across cluster genes is drawn in bold. Individual mean gene log_2 fold changes are plotted in lighter colors.

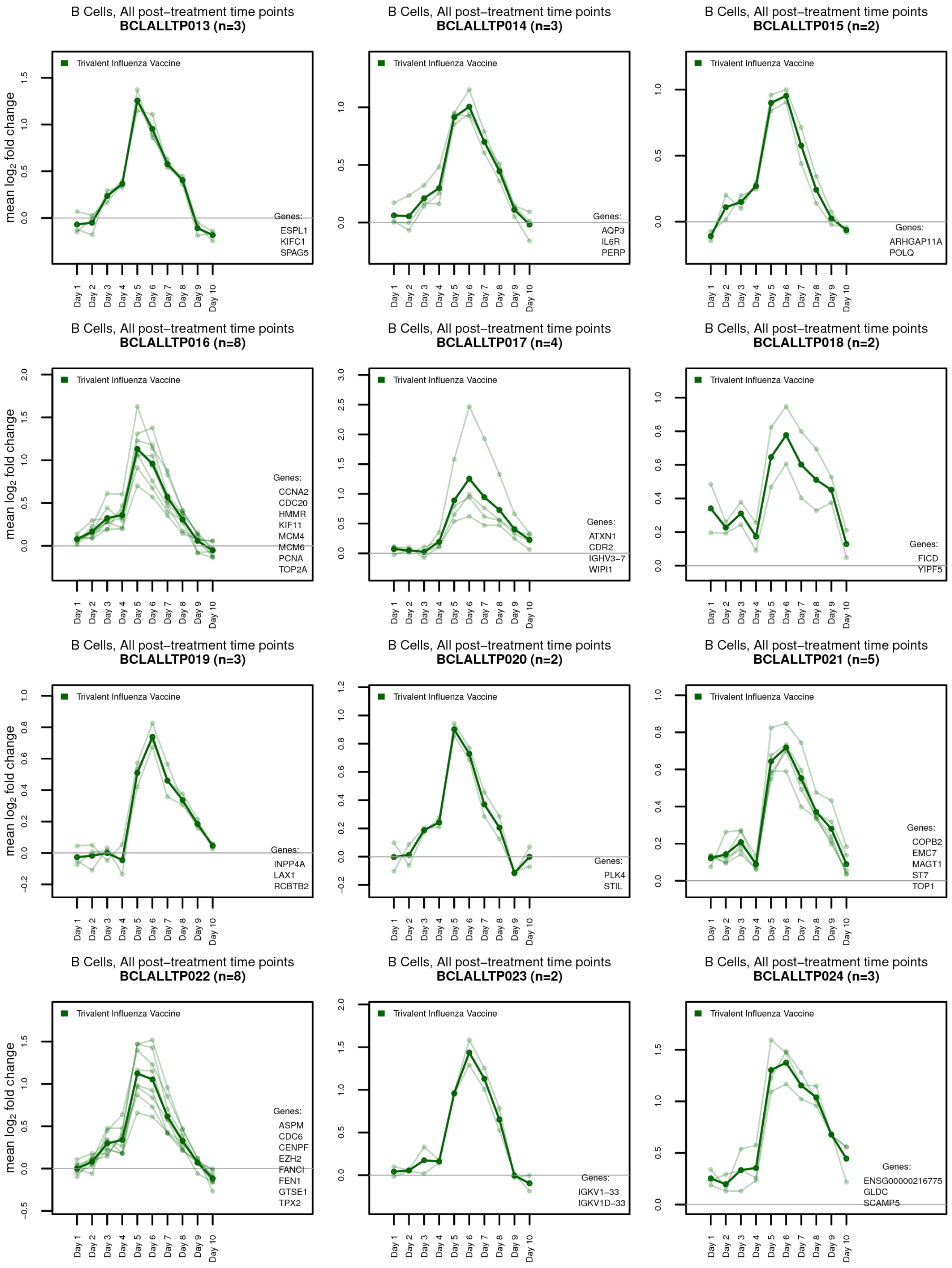

Figure 83: Co-expressed gene cluster time trends of log_2 fold change from pre-treatment levels by treatment 2 of 5 (B Cells). Header indicates cluster ID. Mean log_2 fold change across cluster genes is drawn in bold. Individual mean gene log_2 fold changes are plotted in lighter colors.

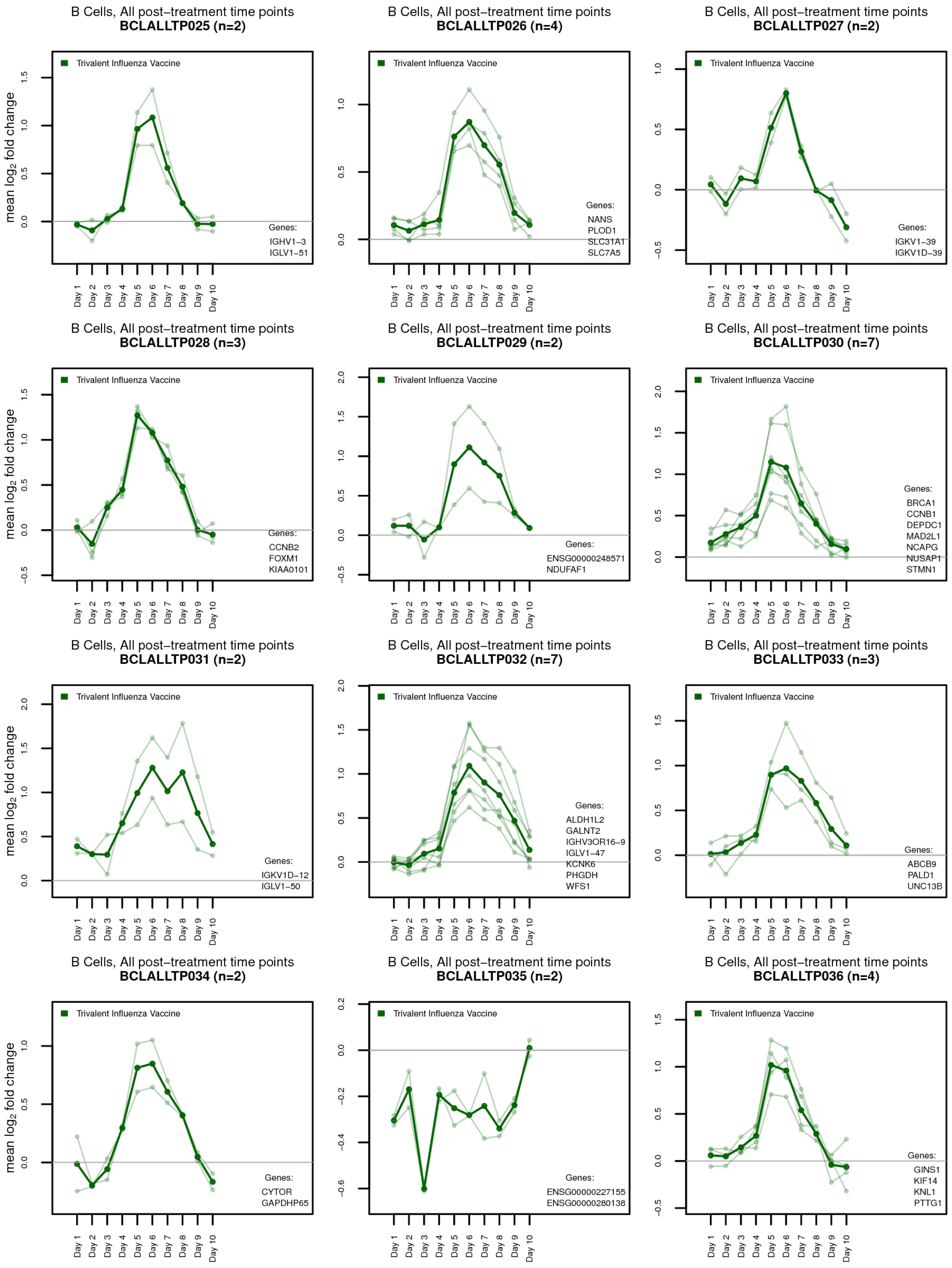

Figure 84: Co-expressed gene cluster time trends of log_2 fold change from pre-treatment levels by treatment 3 of 5 (B Cells). Header indicates cluster ID. Mean log_2 fold change across cluster genes is drawn in bold. Individual mean gene log_2 fold changes are plotted in lighter colors.

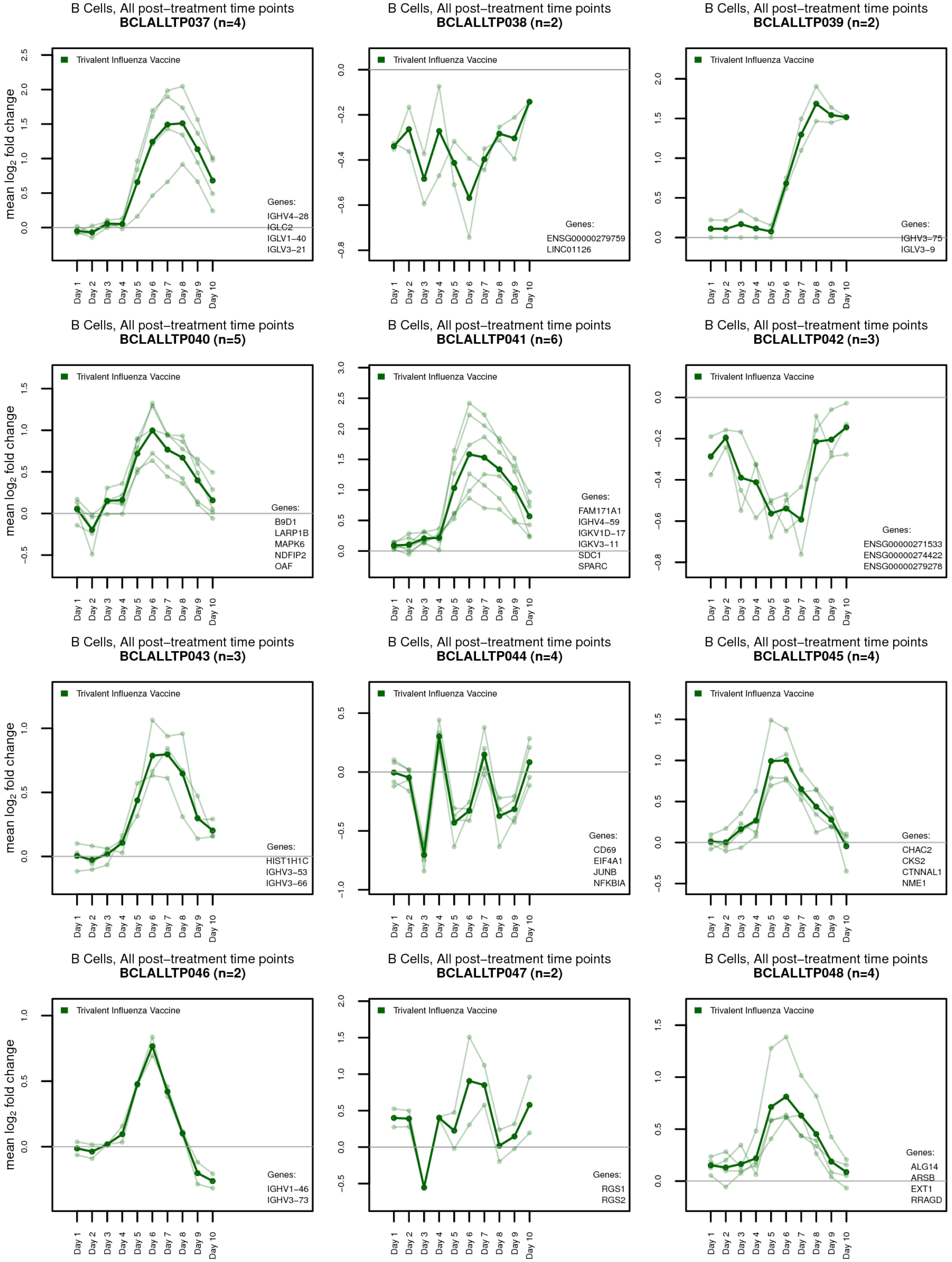

Figure 85: Co-expressed gene cluster time trends of log_2 fold change from pre-treatment levels by treatment 4 of 5 (B Cells). Header indicates cluster ID. Mean log_2 fold change across cluster genes is drawn in bold. Individual mean gene log_2 fold changes are plotted in lighter colors.

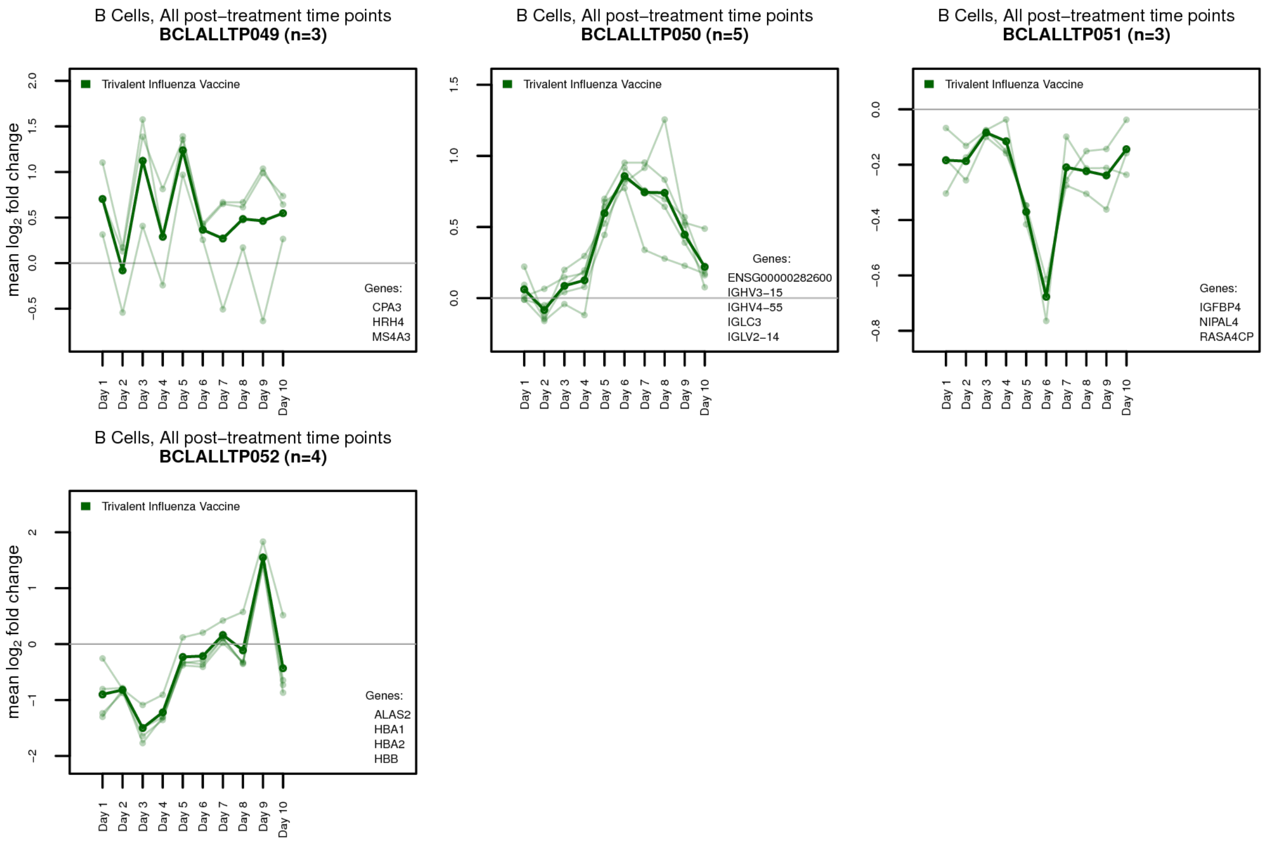

Figure 86: Co-expressed gene cluster time trends of log_2 fold change from pre-treatment levels by treatment 5 of 5 (B Cells). Header indicates cluster ID. Mean log_2 fold change across cluster genes is drawn in bold. Individual mean gene log_2 fold changes are plotted in lighter colors.

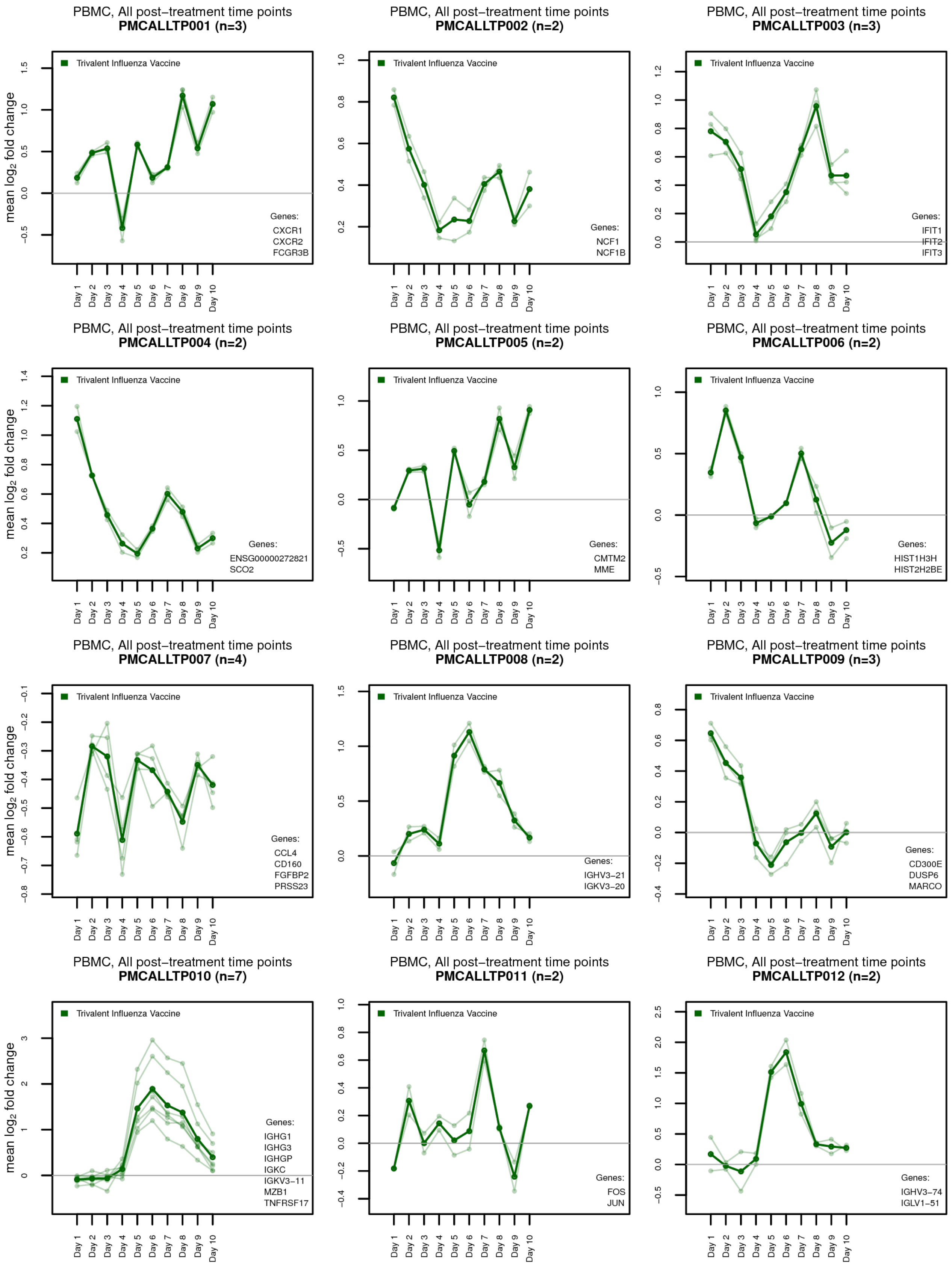

Figure 87: Co-expressed gene cluster time trends of log_2 fold change from pre-treatment levels by treatment 1 of 3 (PBMC). Header indicates cluster ID. Mean log_2 fold change across cluster genes is drawn in bold. Individual mean gene log_2 fold changes are plotted in lighter colors.

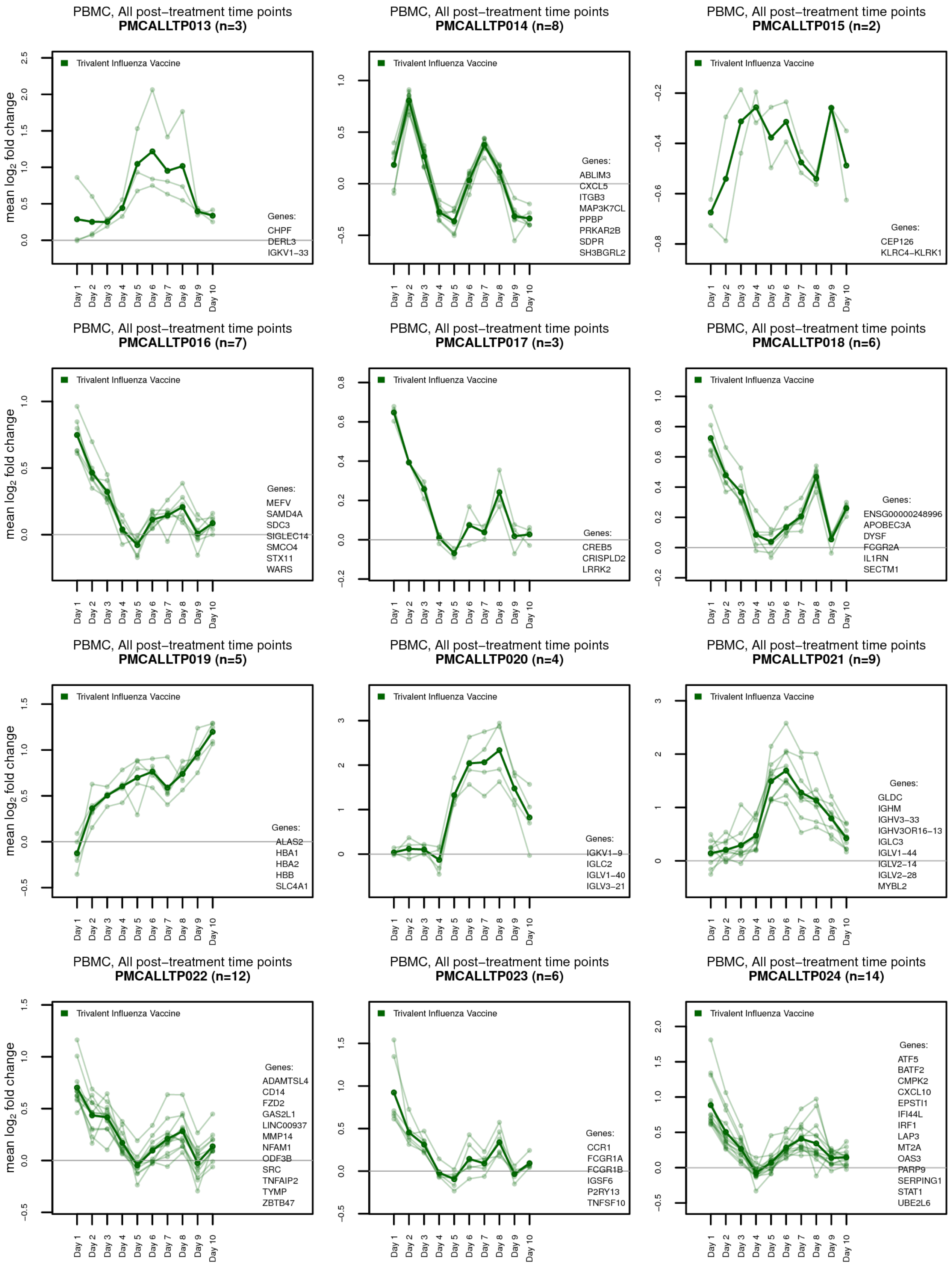

Figure 88: Co-expressed gene cluster time trends of log_2 fold change from pre-treatment levels by treatment 2 of 3 (PBMC). Header indicates cluster ID. Mean log_2 fold change across cluster genes is drawn in bold. Individual mean gene log_2 fold changes are plotted in lighter colors.

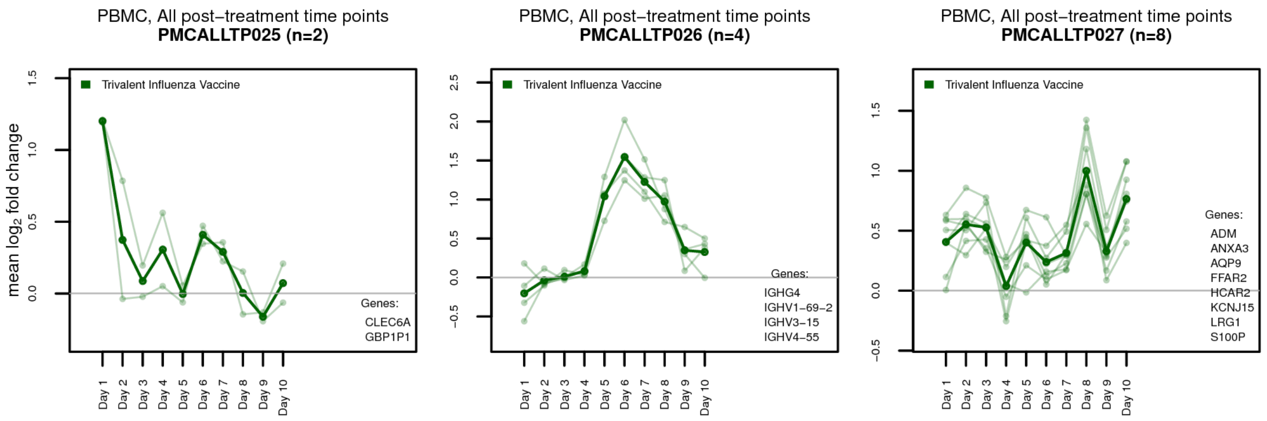

Figure 89: Co-expressed gene cluster time trends of log_2 fold change from pre-treatment levels by treatment 3 of 3 (PBMC). Header indicates cluster ID. Mean log_2 fold change across cluster genes is drawn in bold. Individual mean gene log_2 fold changes are plotted in lighter colors.

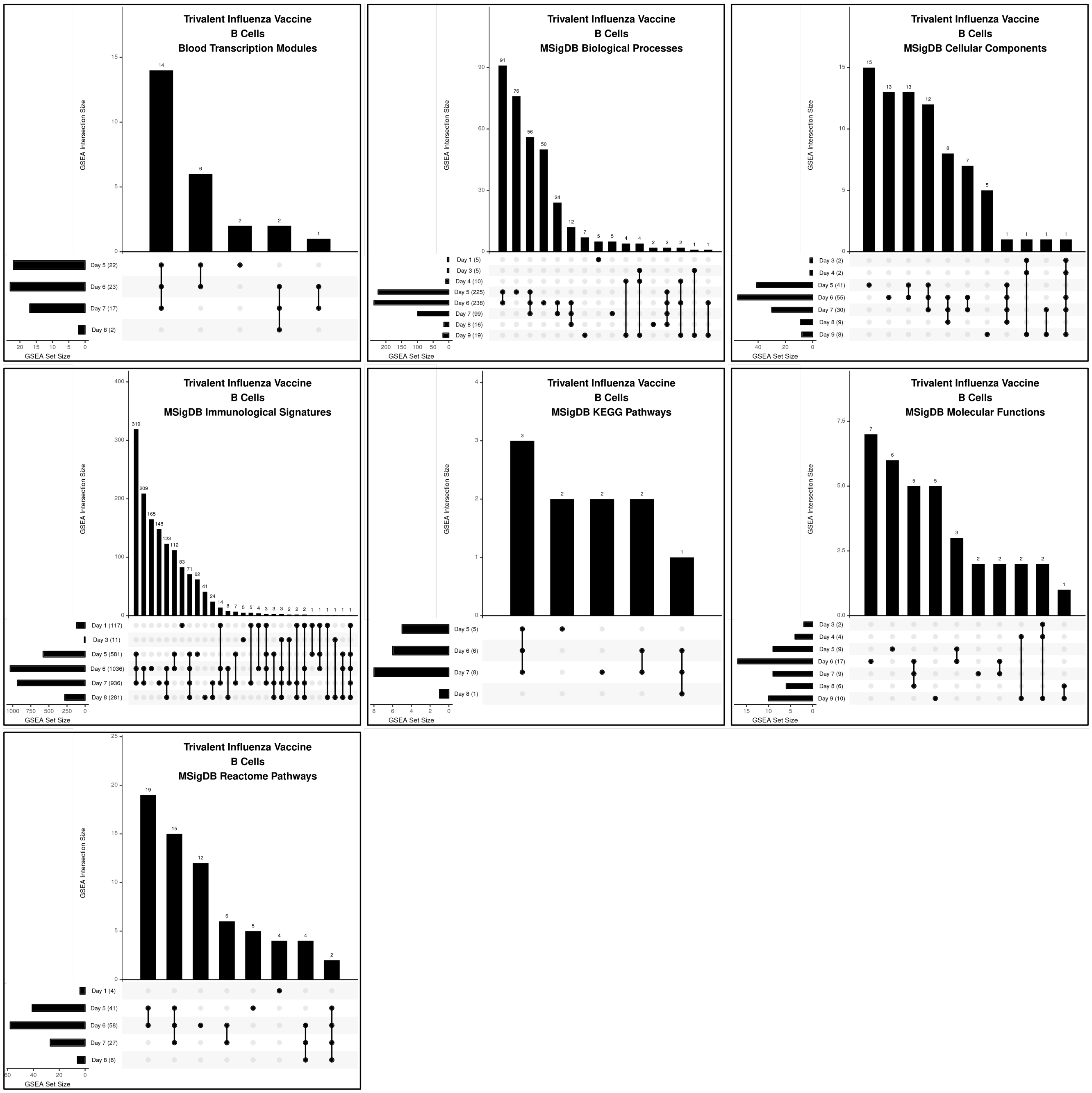

Figure 90: UpSet plots of enriched gene sets between post-treatment time points (B Cells, Trivalent Influenza Vaccine).

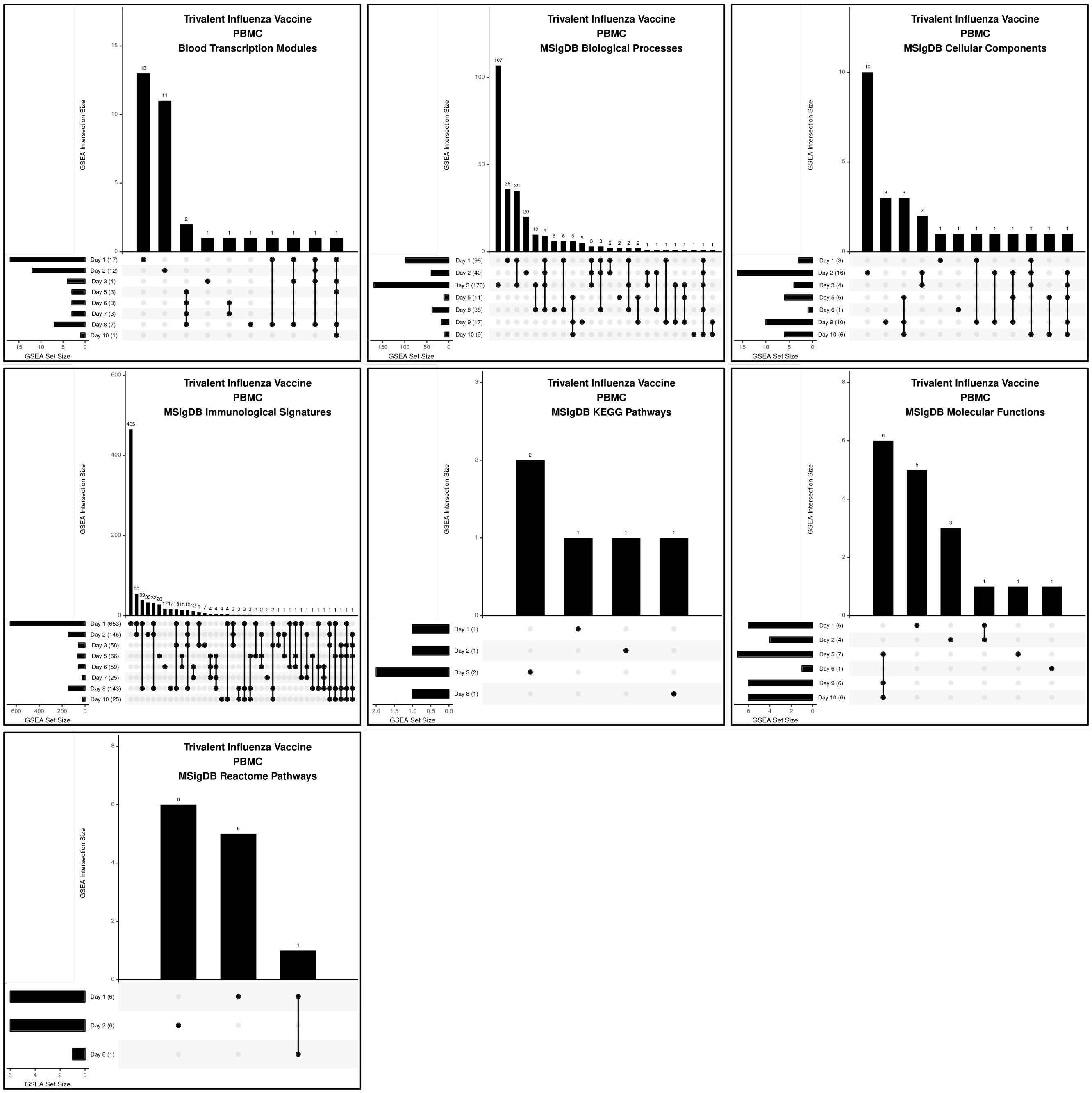

Figure 91: UpSet plots of enriched gene sets between post-treatment time points (PBMC, Trivalent Influenza Vaccine).

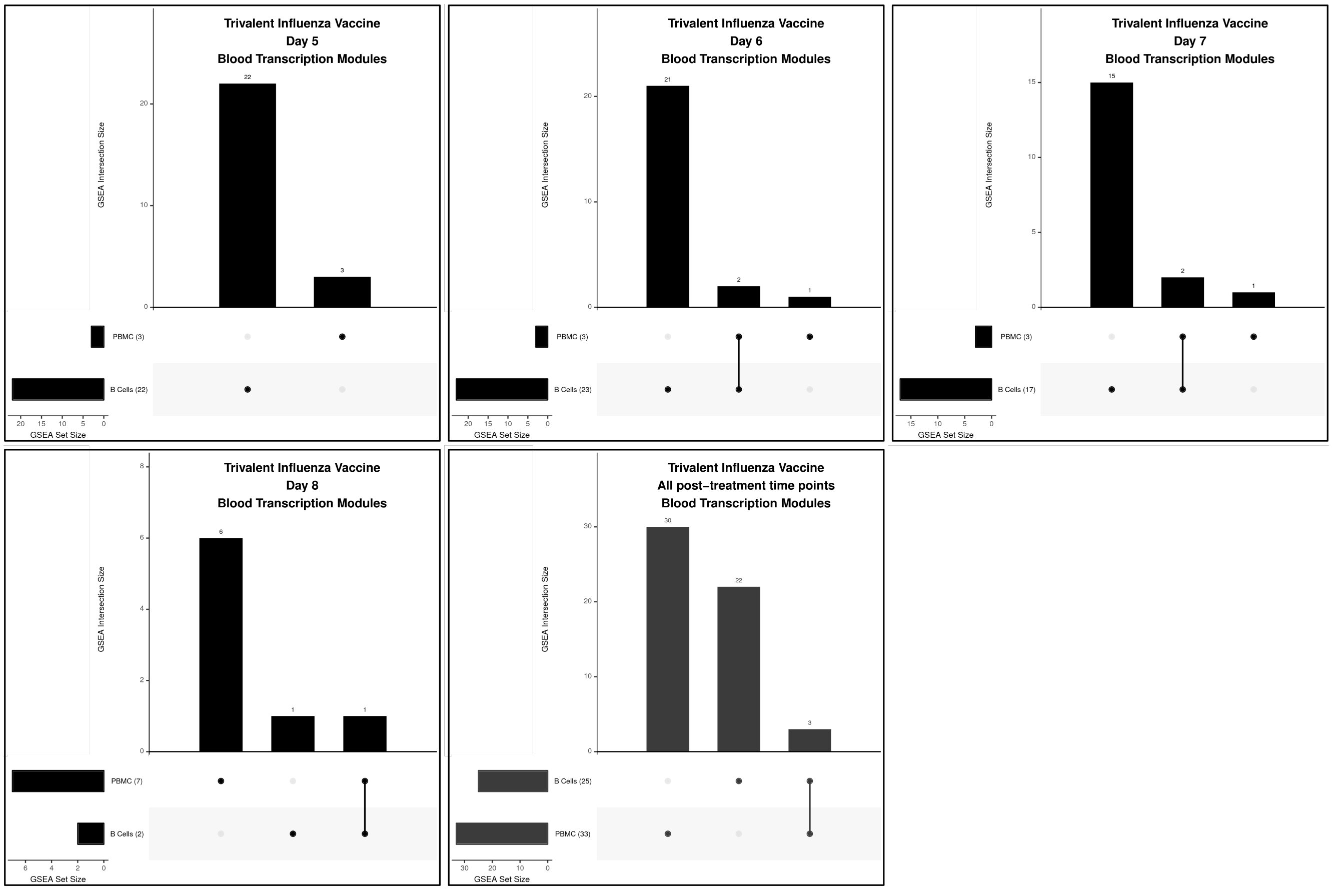

Figure 92: UpSet plots of enriched gene sets between specimen types (Trivalent Influenza Vaccine, Blood Transcription Modules).

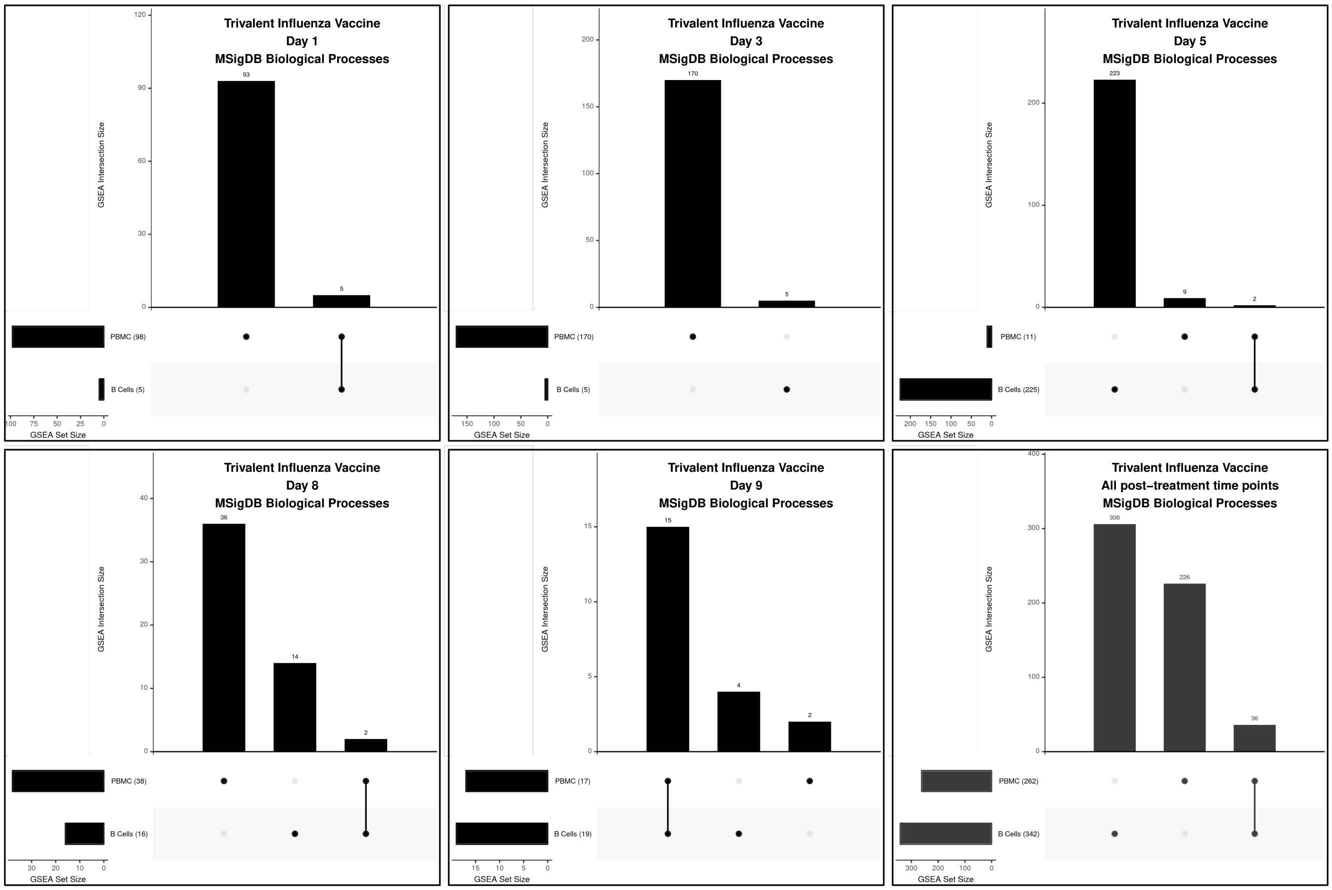

Figure 93: UpSet plots of enriched gene sets between specimen types (Trivalent Influenza Vaccine, MSigDB Biological Processes).

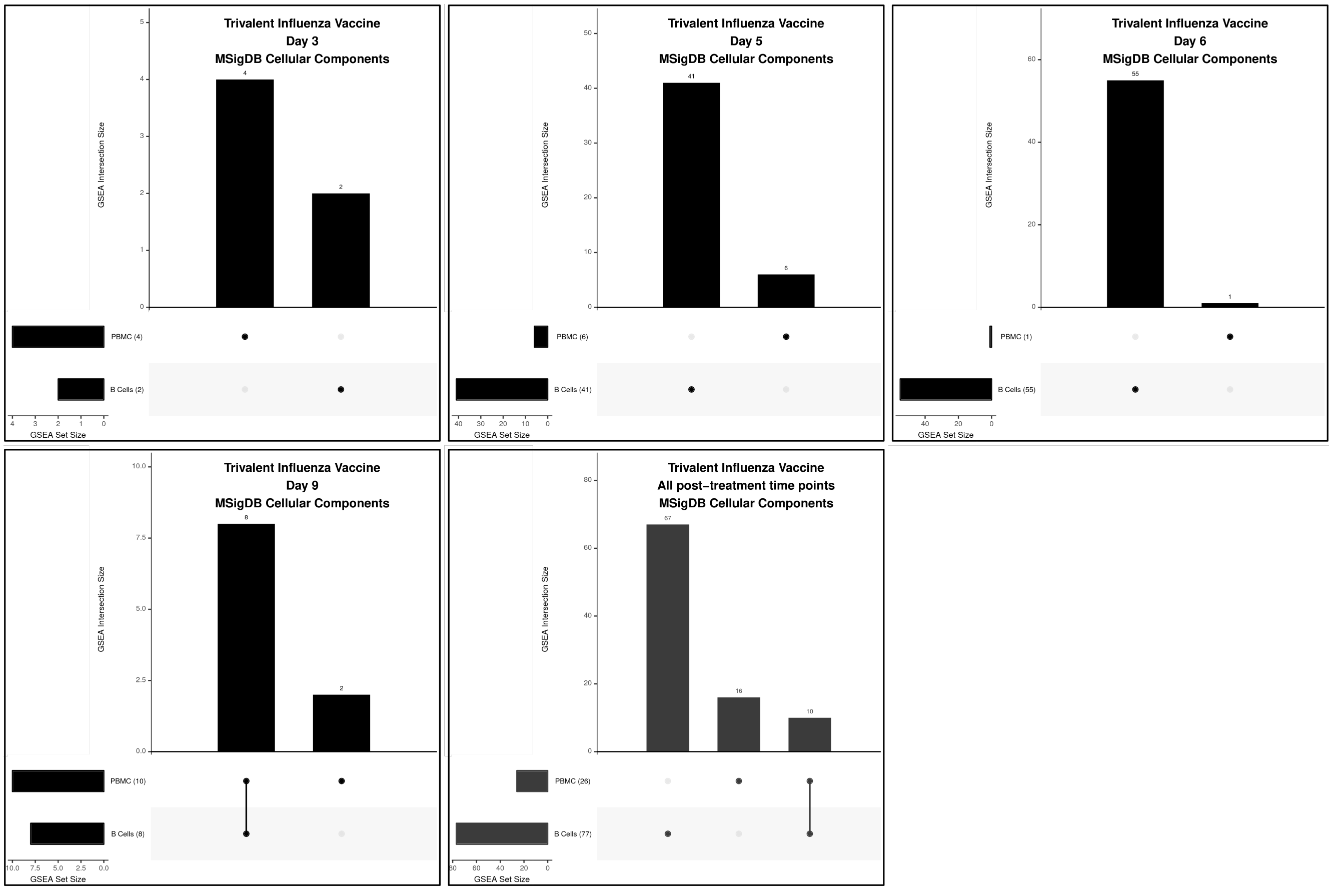

Figure 94: UpSet plots of enriched gene sets between specimen types (Trivalent Influenza Vaccine, MSigDB Cellular Components).

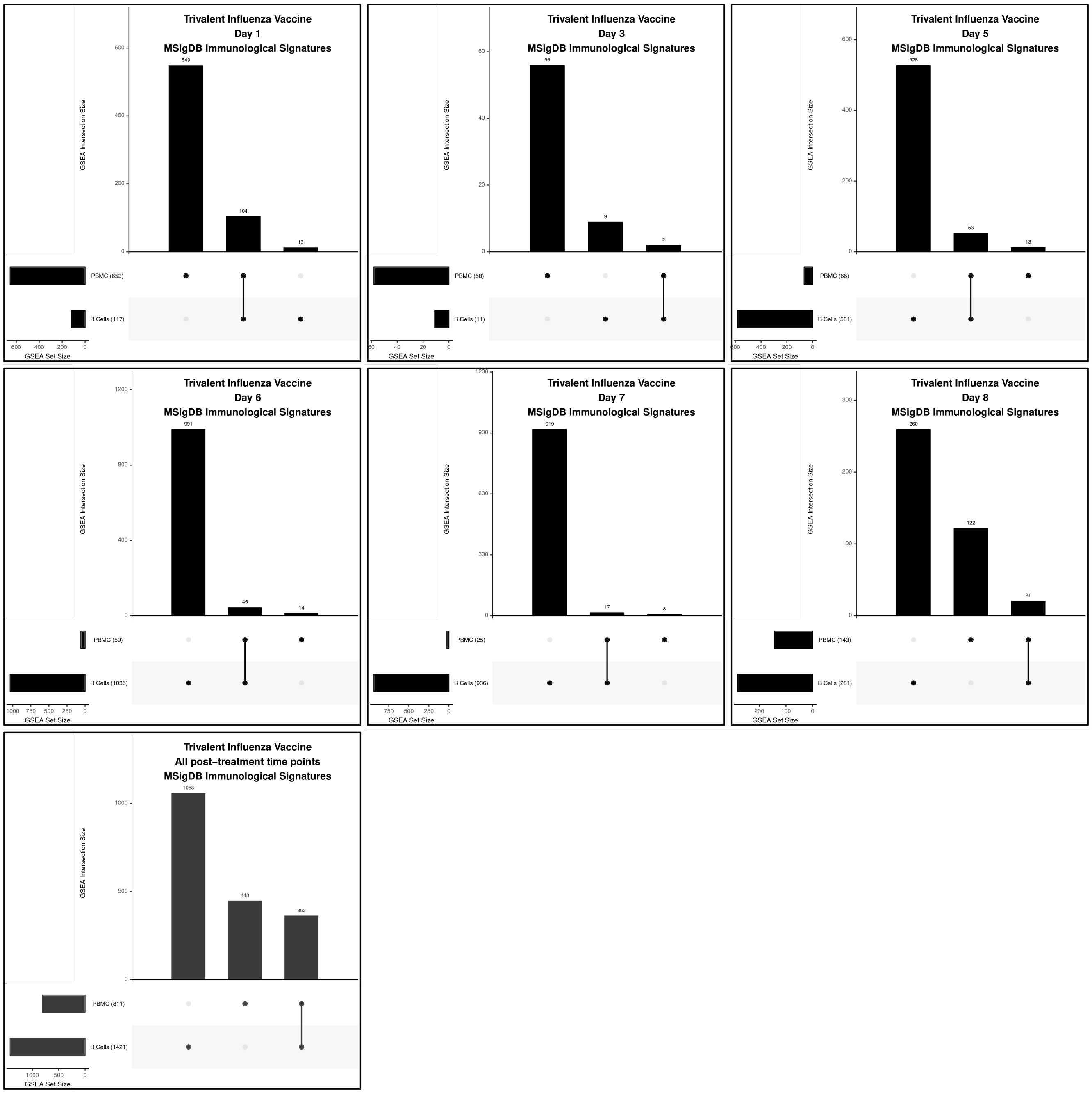

Figure 95: UpSet plots of enriched gene sets between specimen types (Trivalent Influenza Vaccine, MSigDB Immunological Signatures).

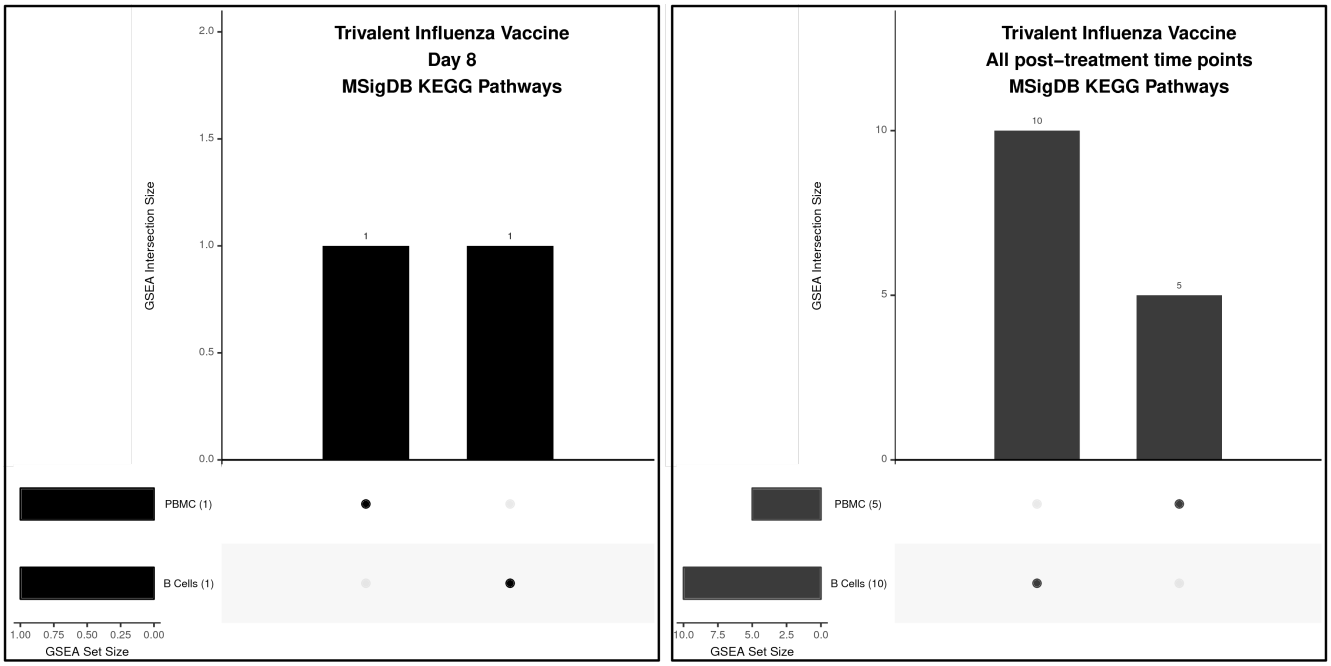

Figure 96: UpSet plots of enriched gene sets between specimen types (Trivalent Influenza Vaccine, MSigDB KEGG Pathways).

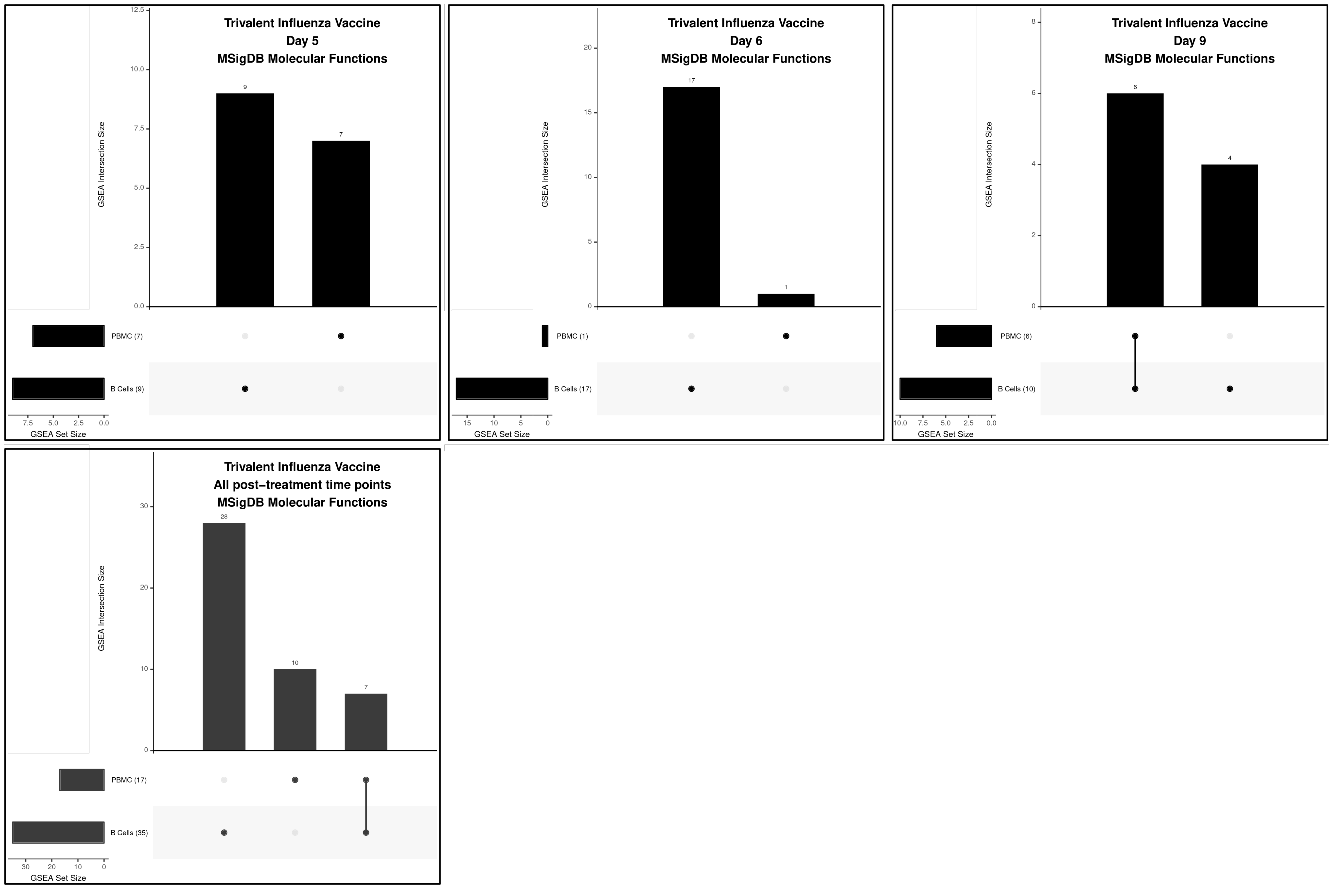

Figure 97: UpSet plots of enriched gene sets between specimen types (Trivalent Influenza Vaccine, MSigDB Molecular Functions).

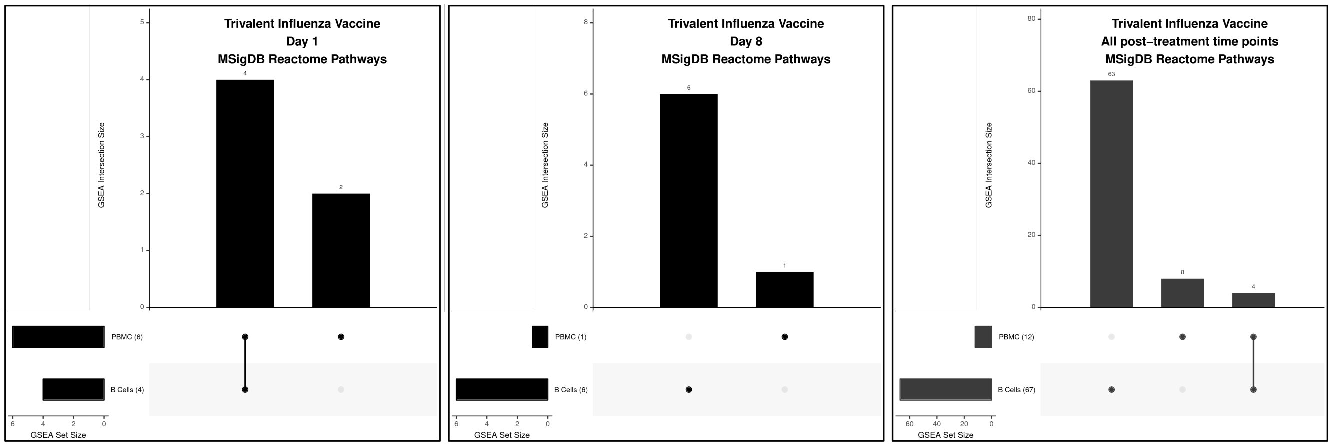

Figure 98: UpSet plots of enriched gene sets between specimen types (Trivalent Influenza Vaccine, MSigDB Reactome Pathways).

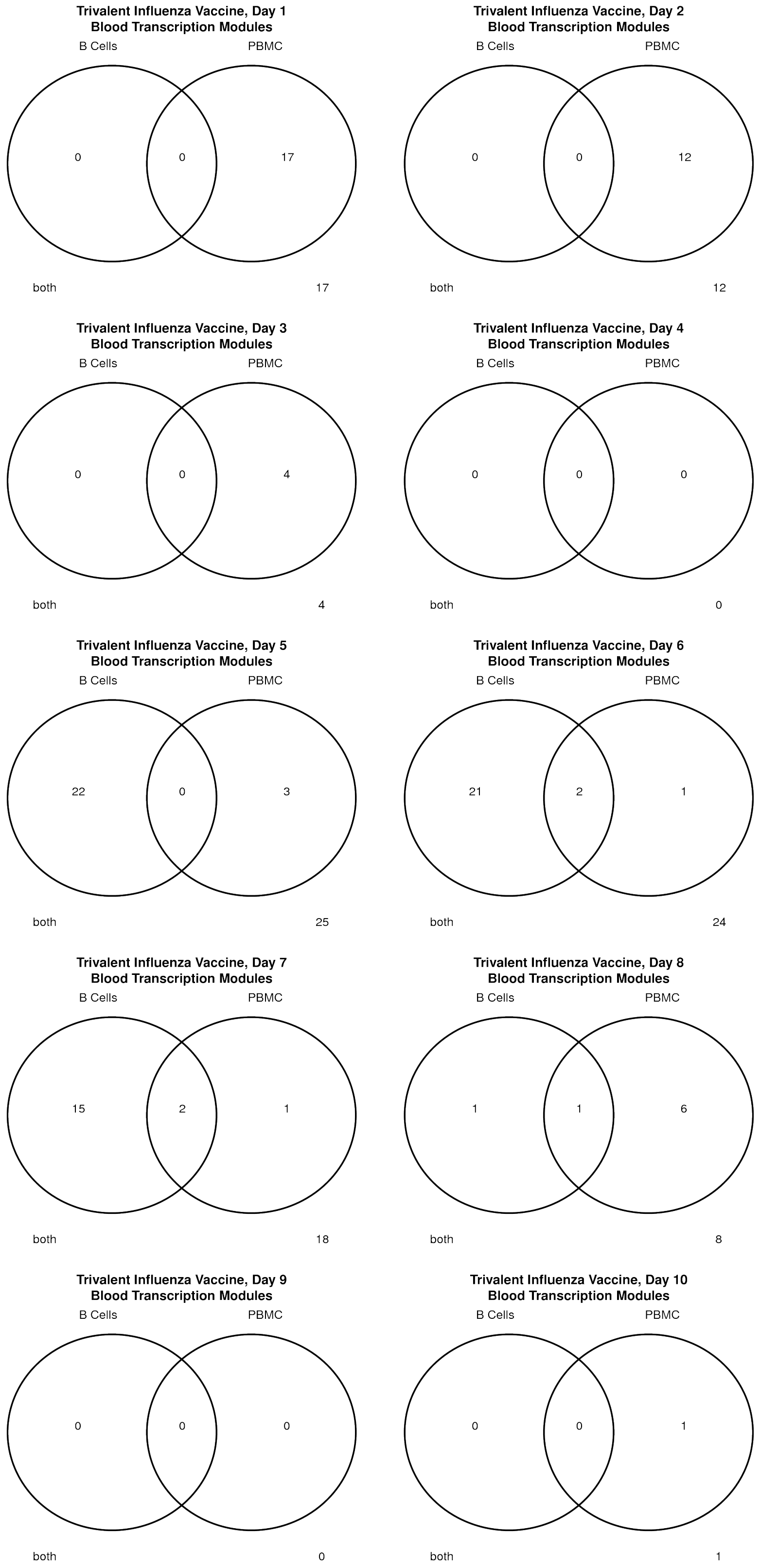

Figure 99: Venn diagrams of enriched Blood Transcription Modules between specimen types 1 of 2 (Trivalent Influenza Vaccine).

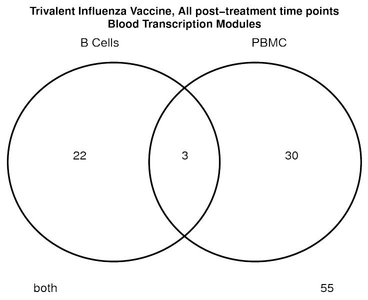

Figure 100: Venn diagrams of enriched Blood Transcription Modules between specimen types 2 of 2 (Trivalent Influenza Vaccine).

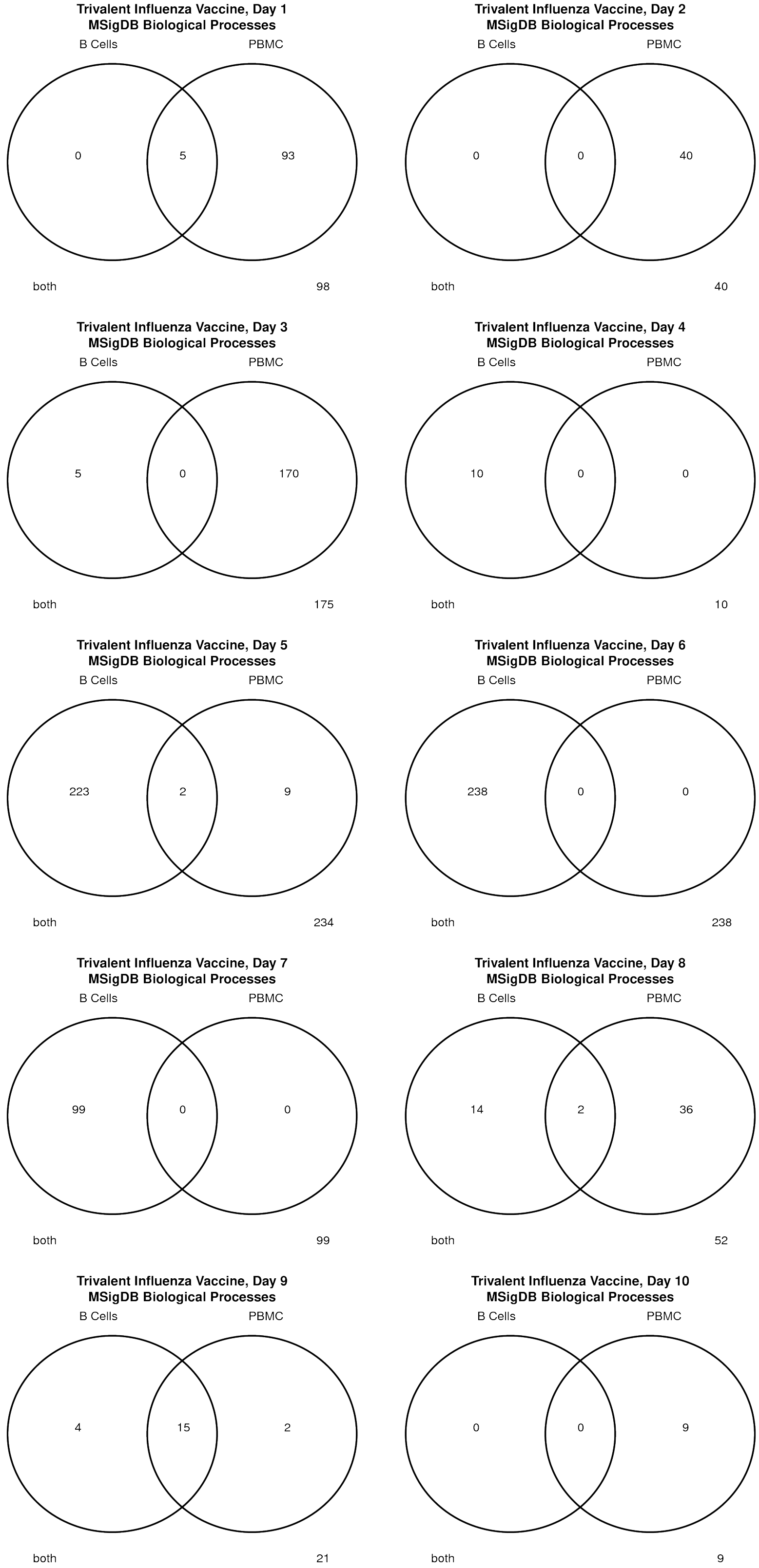

Figure 101: Venn diagrams of enriched MSigDB Biological Processes between specimen types 1 of 2 (Trivalent Influenza Vaccine).

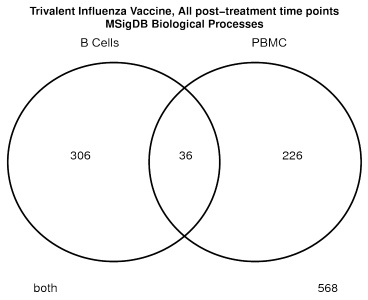

Figure 102: Venn diagrams of enriched MSigDB Biological Processes between specimen types 2 of 2 (Trivalent Influenza Vaccine).

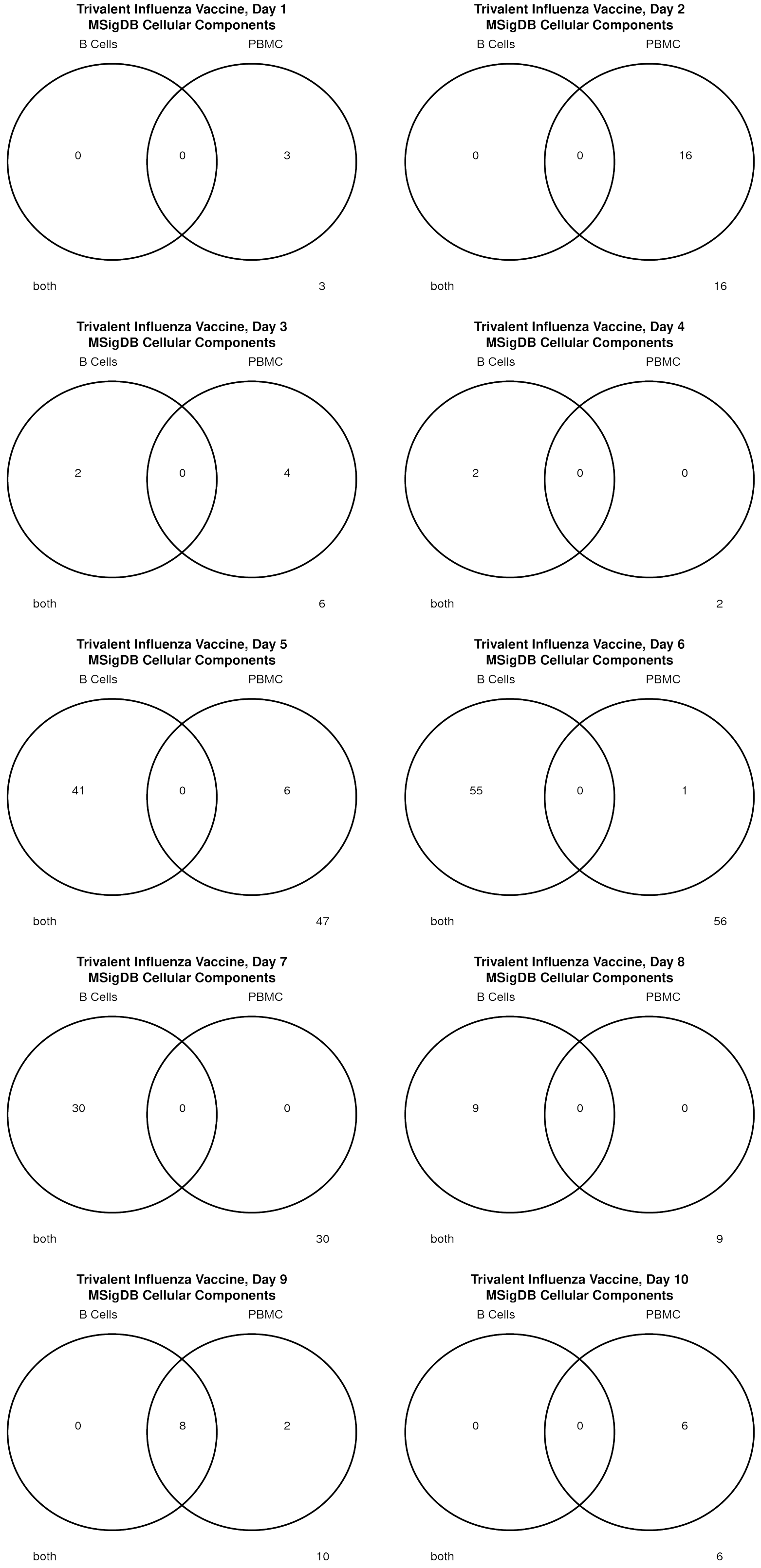

Figure 103: Venn diagrams of enriched MSigDB Cellular Components between specimen types 1 of 2 (Trivalent Influenza Vaccine).

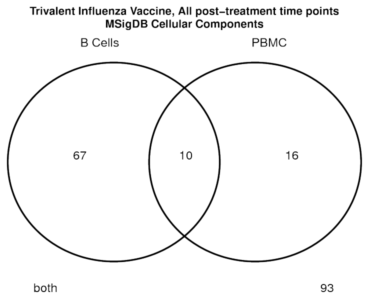

Figure 104: Venn diagrams of enriched MSigDB Cellular Components between specimen types 2 of 2 (Trivalent Influenza Vaccine).

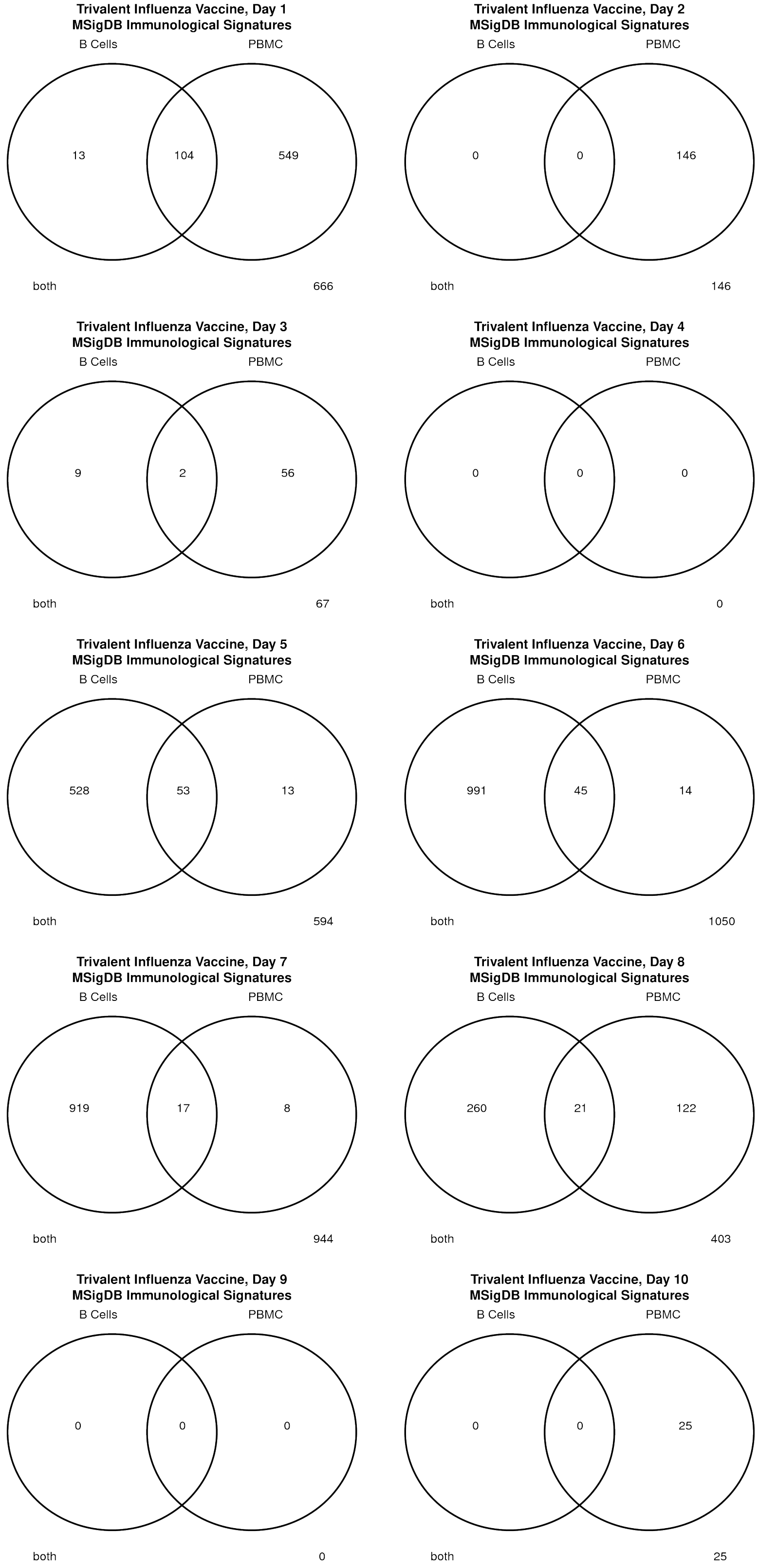

Figure 105: Venn diagrams of enriched MSigDB Immunological Signatures between specimen types 1 of 2 (Trivalent Influenza Vaccine).

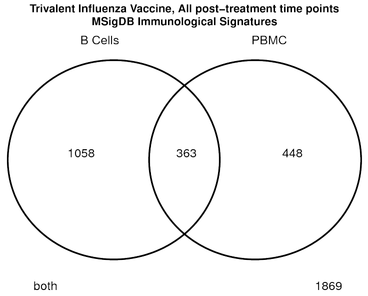

Figure 106: Venn diagrams of enriched MSigDB Immunological Signatures between specimen types 2 of 2 (Trivalent Influenza Vaccine).

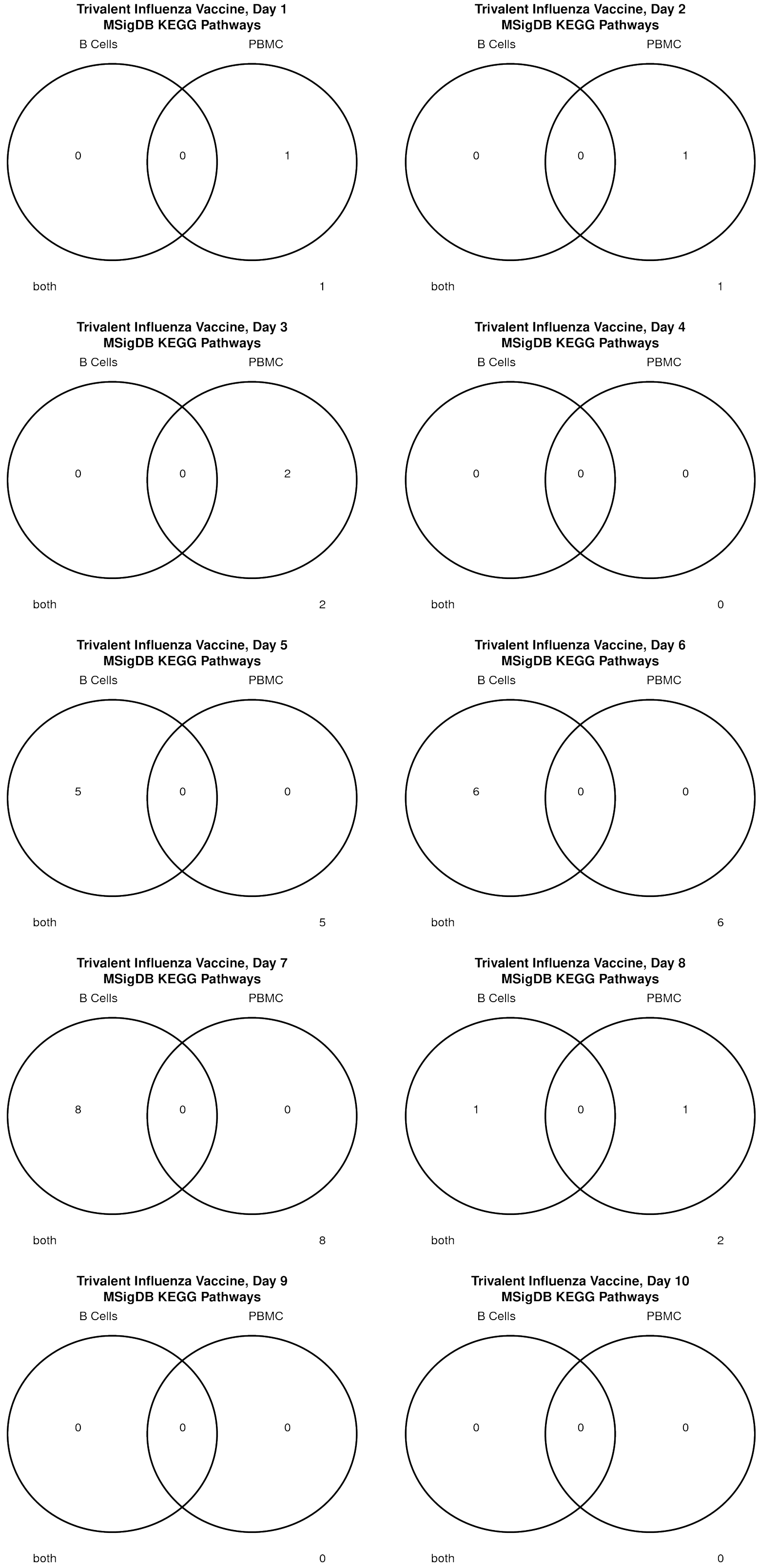

Figure 107: Venn diagrams of enriched MSigDB KEGG Pathways between specimen types 1 of 2 (Trivalent Influenza Vaccine).

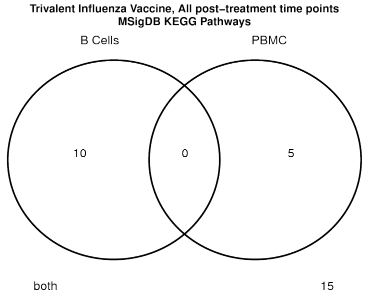

Figure 108: Venn diagrams of enriched MSigDB KEGG Pathways between specimen types 2 of 2 (Trivalent Influenza Vaccine).

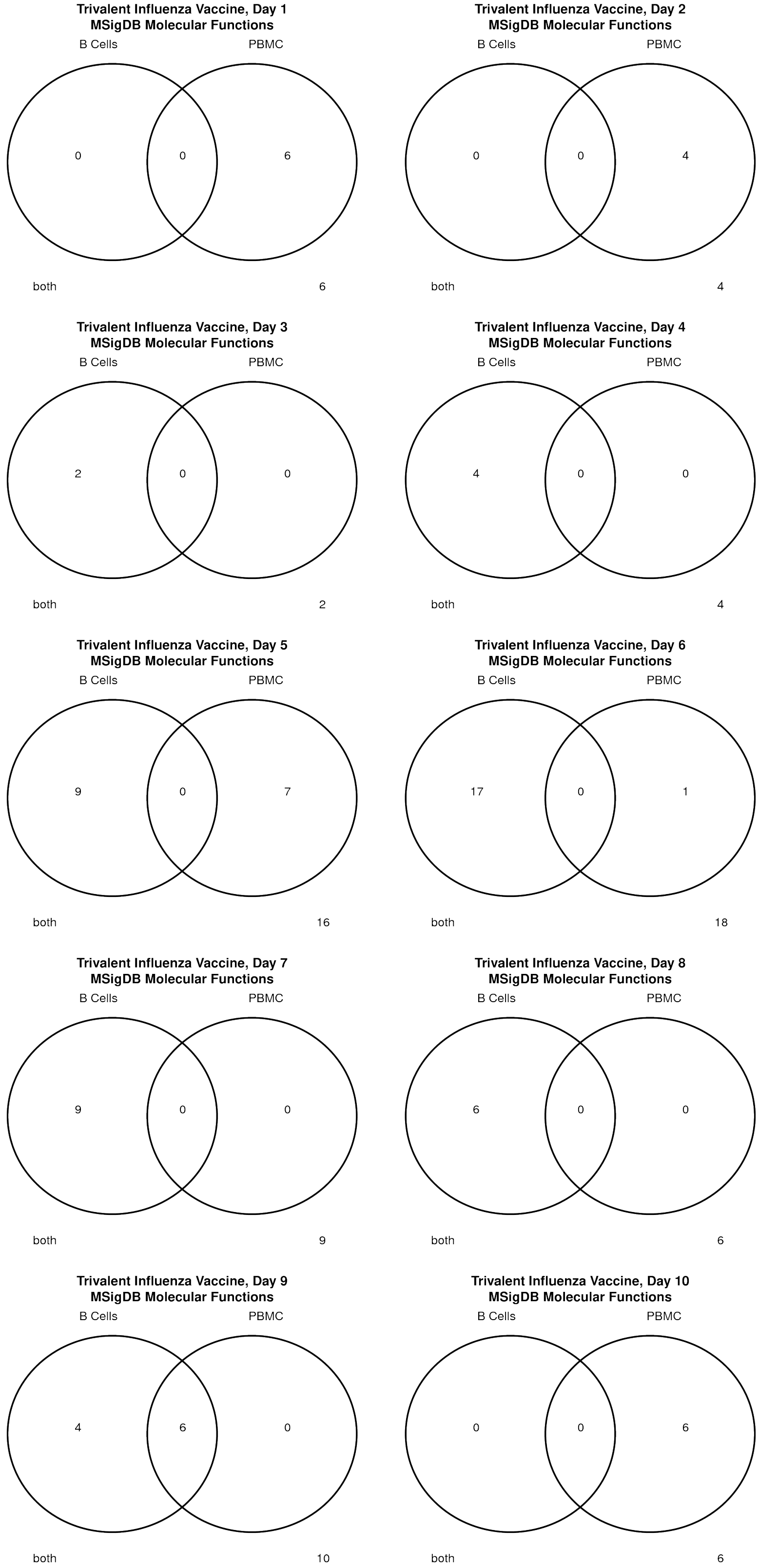

Figure 109: Venn diagrams of enriched MSigDB Molecular Functions between specimen types 1 of 2 (Trivalent Influenza Vaccine).

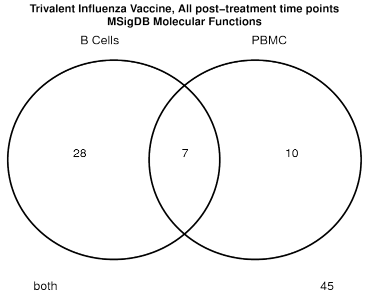

Figure 110: Venn diagrams of enriched MSigDB Molecular Functions between specimen types 2 of 2 (Trivalent Influenza Vaccine).

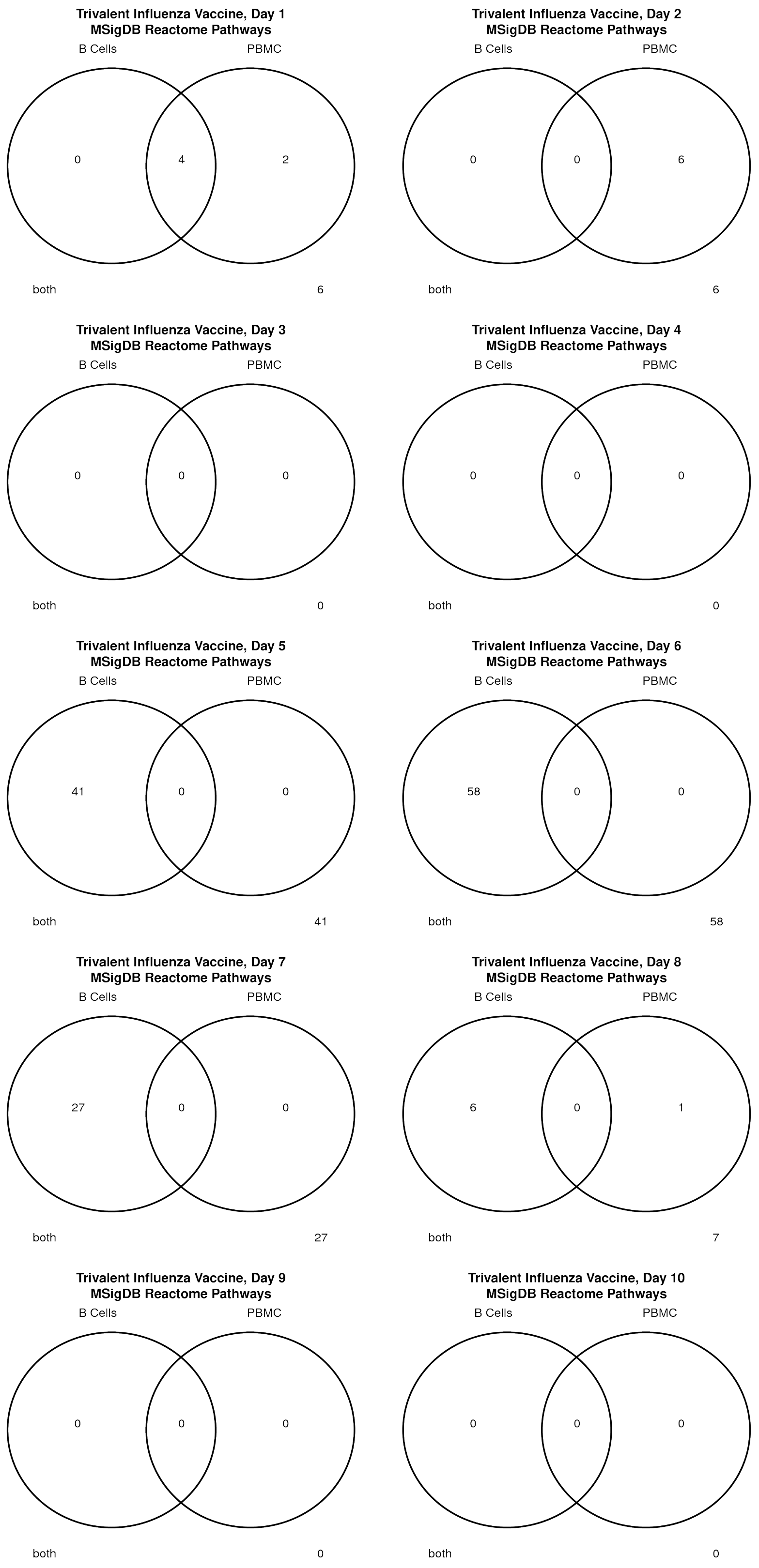

Figure 111: Venn diagrams of enriched MSigDB Reactome Pathways between specimen types 1 of 2 (Trivalent Influenza Vaccine).

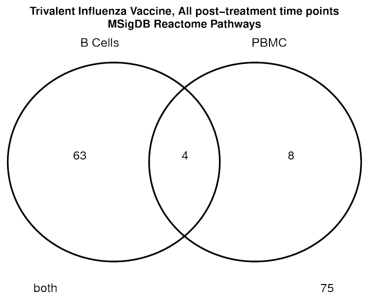

Figure 112: Venn diagrams of enriched MSigDB Reactome Pathways between specimen types 2 of 2 (Trivalent Influenza Vaccine).

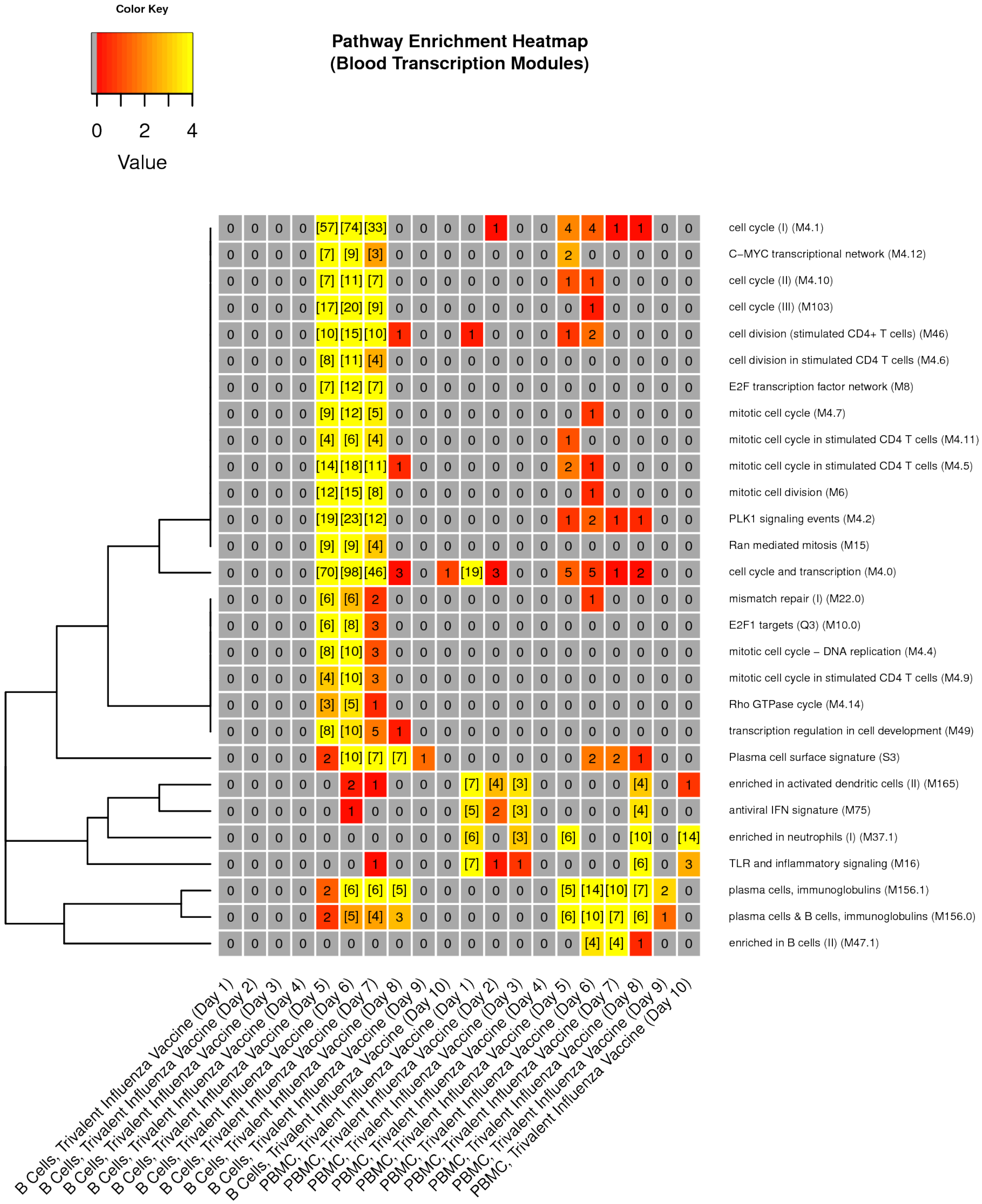

Figure 113: Heatmap of enriched Blood Transcription Modules (RNA-Seq). Gene sets significantly enriched in at least two conditions are shown. Cells are color-coded by the enrichment score (-1 x log_{10}(FDR-adjusted p-value))). Cells contain the number of significant genes in the gene set with gene numbers in brackets indicating significantly enriched sets. Sets were clustered based on the euclidean distance between their enrichment score pattern. If there were more than 50 gene sets in the results, gene sets were further filtered to only include sets whose sum of enrichment score across conditions was the top 50 of all sets. Cells colored in grey have an enrichment score equal to zero.

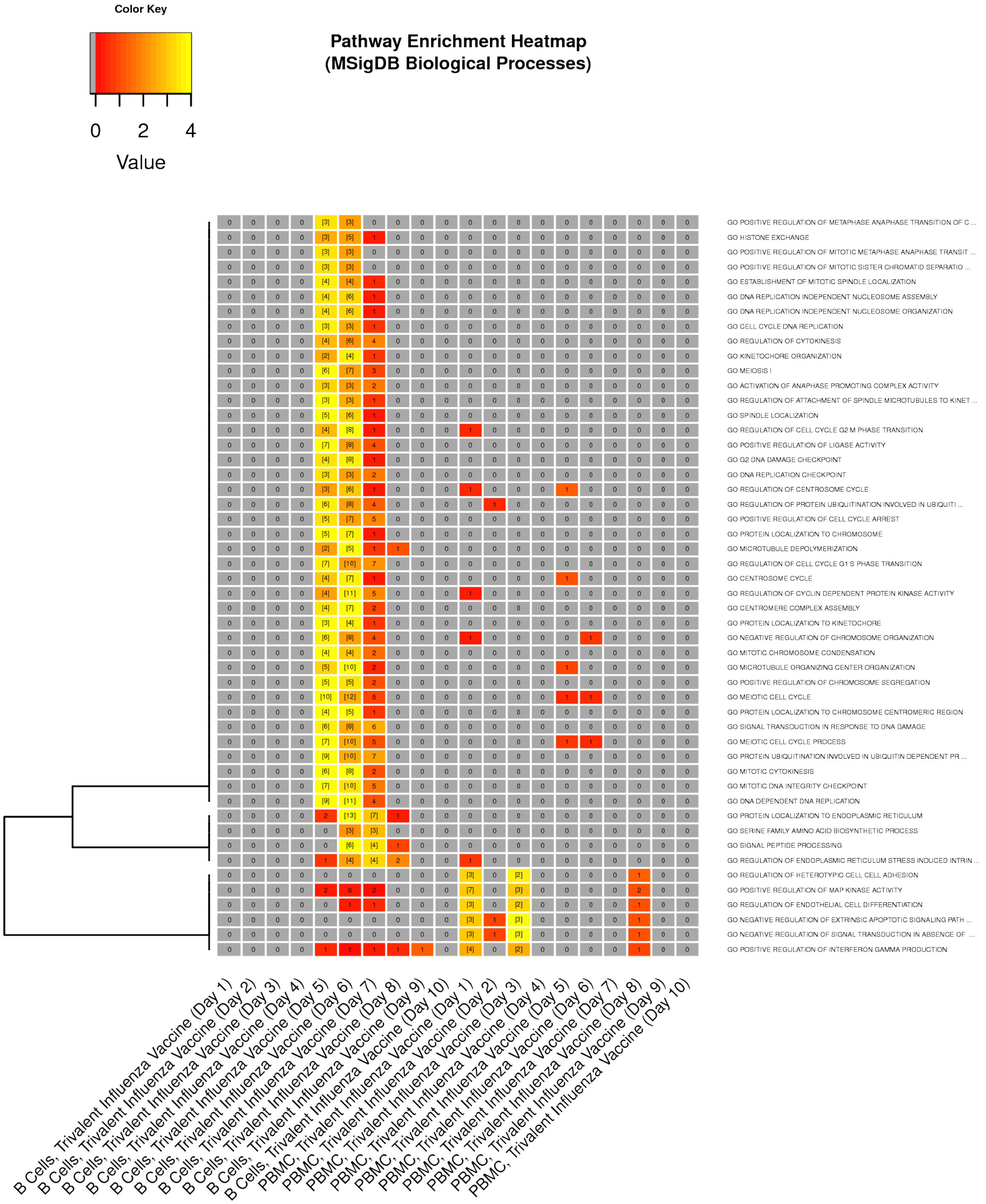

Figure 114: Heatmap of enriched MSigDB Biological Processes (RNA-Seq). Gene sets significantly enriched in at least two conditions are shown. Cells are color-coded by the enrichment score (-1 x log_{10}(FDR-adjusted p-value))). Cells contain the number of significant genes in the gene set with gene numbers in brackets indicating significantly enriched sets. Sets were clustered based on the euclidean distance between their enrichment score pattern. If there were more than 50 gene sets in the results, gene sets were further filtered to only include sets whose sum of enrichment score across conditions was the top 50 of all sets. Cells colored in grey have an enrichment score equal to zero.

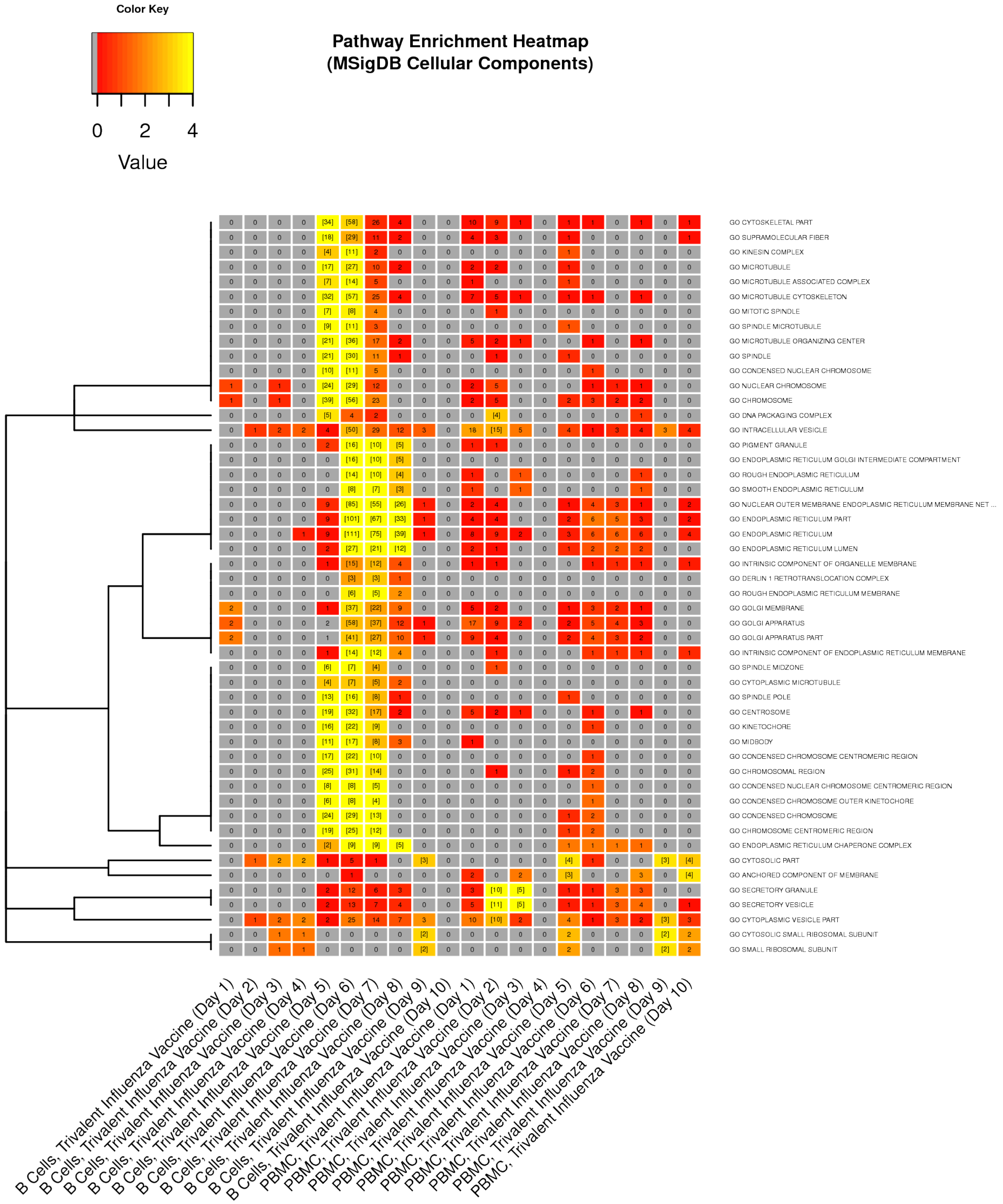

Figure 115: Heatmap of enriched MSigDB Cellular Components (RNA-Seq). Gene sets significantly enriched in at least two conditions are shown. Cells are color-coded by the enrichment score (-1 x log_{10}(FDR-adjusted p-value))). Cells contain the number of significant genes in the gene set with gene numbers in brackets indicating significantly enriched sets. Sets were clustered based on the euclidean distance between their enrichment score pattern. If there were more than 50 gene sets in the results, gene sets were further filtered to only include sets whose sum of enrichment score across conditions was the top 50 of all sets. Cells colored in grey have an enrichment score equal to zero.

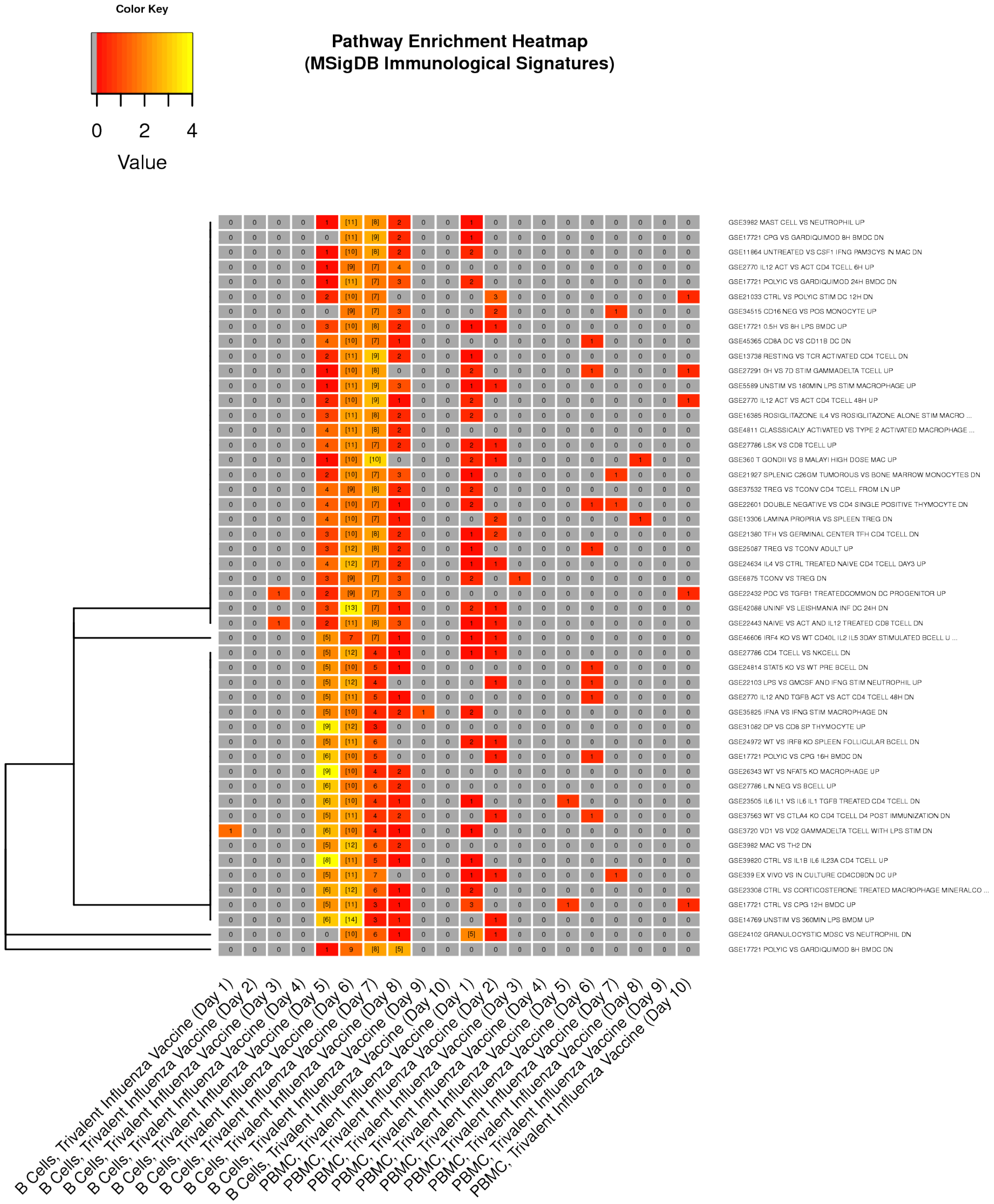

Figure 116: Heatmap of enriched MSigDB Immunological Signatures (RNA-Seq). Gene sets significantly enriched in at least two conditions are shown. Cells are color-coded by the enrichment score (-1 x log_{10}(FDR-adjusted p-value))). Cells contain the number of significant genes in the gene set with gene numbers in brackets indicating significantly enriched sets. Sets were clustered based on the euclidean distance between their enrichment score pattern. If there were more than 50 gene sets in the results, gene sets were further filtered to only include sets whose sum of enrichment score across conditions was the top 50 of all sets. Cells colored in grey have an enrichment score equal to zero.

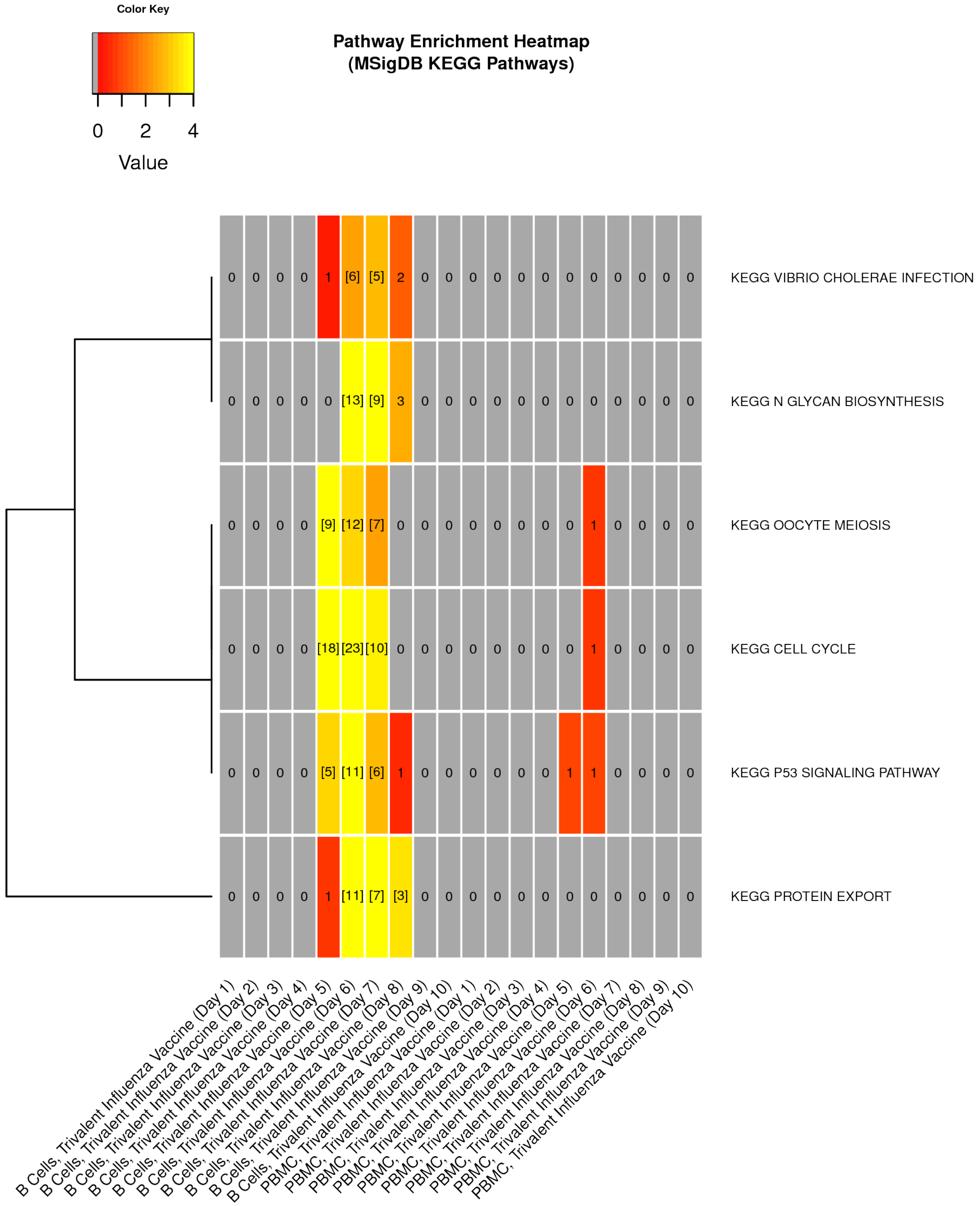

Figure 117: Heatmap of enriched MSigDB KEGG Pathways (RNA-Seq). Gene sets significantly enriched in at least two conditions are shown. Cells are color-coded by the enrichment score (-1 x log_{10}(FDR-adjusted p-value))). Cells contain the number of significant genes in the gene set with gene numbers in brackets indicating significantly enriched sets. Sets were clustered based on the euclidean distance between their enrichment score pattern. If there were more than 50 gene sets in the results, gene sets were further filtered to only include sets whose sum of enrichment score across conditions was the top 50 of all sets. Cells colored in grey have an enrichment score equal to zero.

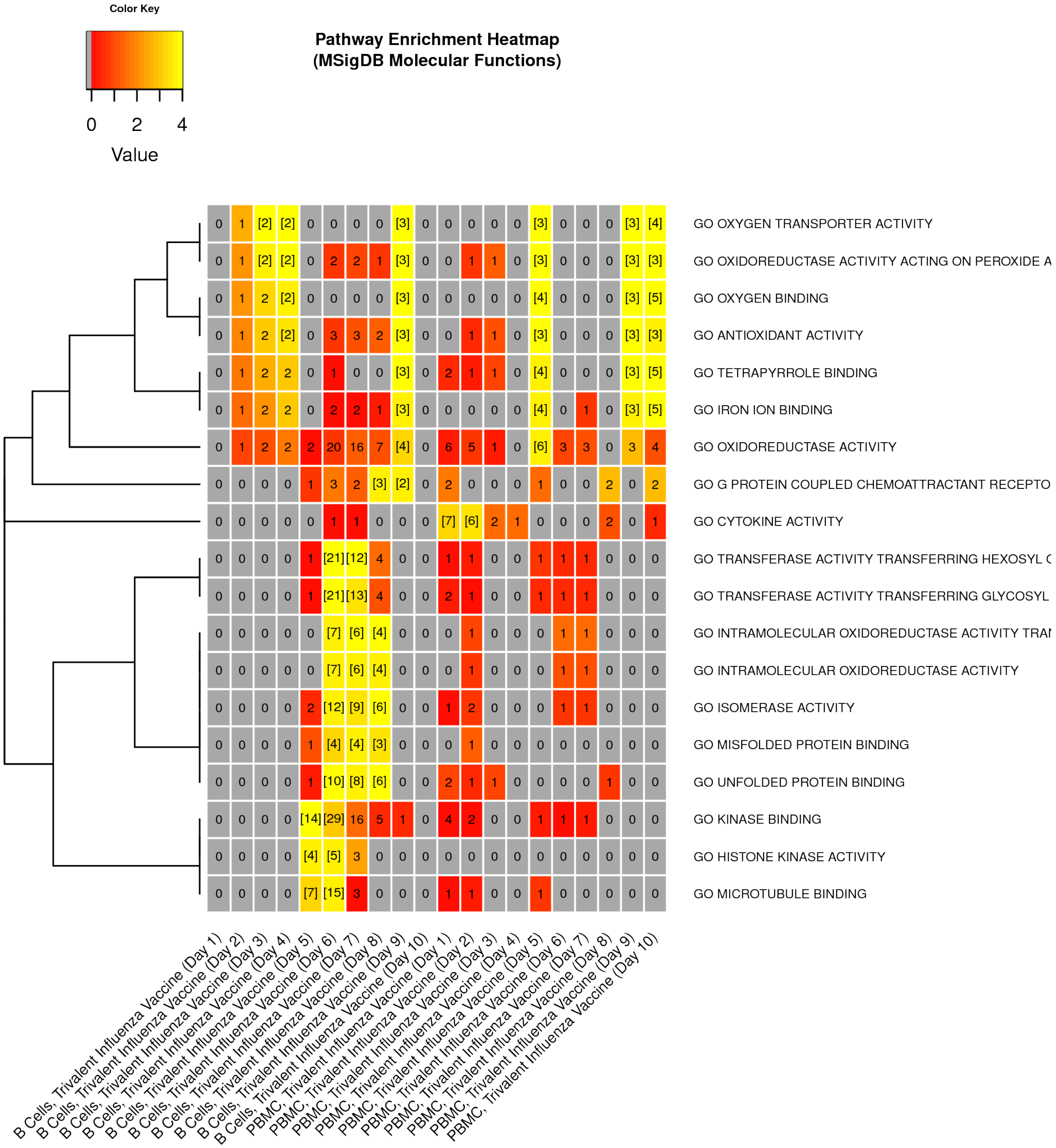

Figure 118: Heatmap of enriched MSigDB Molecular Functions (RNA-Seq). Gene sets significantly enriched in at least two conditions are shown. Cells are color-coded by the enrichment score (-1 x log_{10}(FDR-adjusted p-value))). Cells contain the number of significant genes in the gene set with gene numbers in brackets indicating significantly enriched sets. Sets were clustered based on the euclidean distance between their enrichment score pattern. If there were more than 50 gene sets in the results, gene sets were further filtered to only include sets whose sum of enrichment score across conditions was the top 50 of all sets. Cells colored in grey have an enrichment score equal to zero.

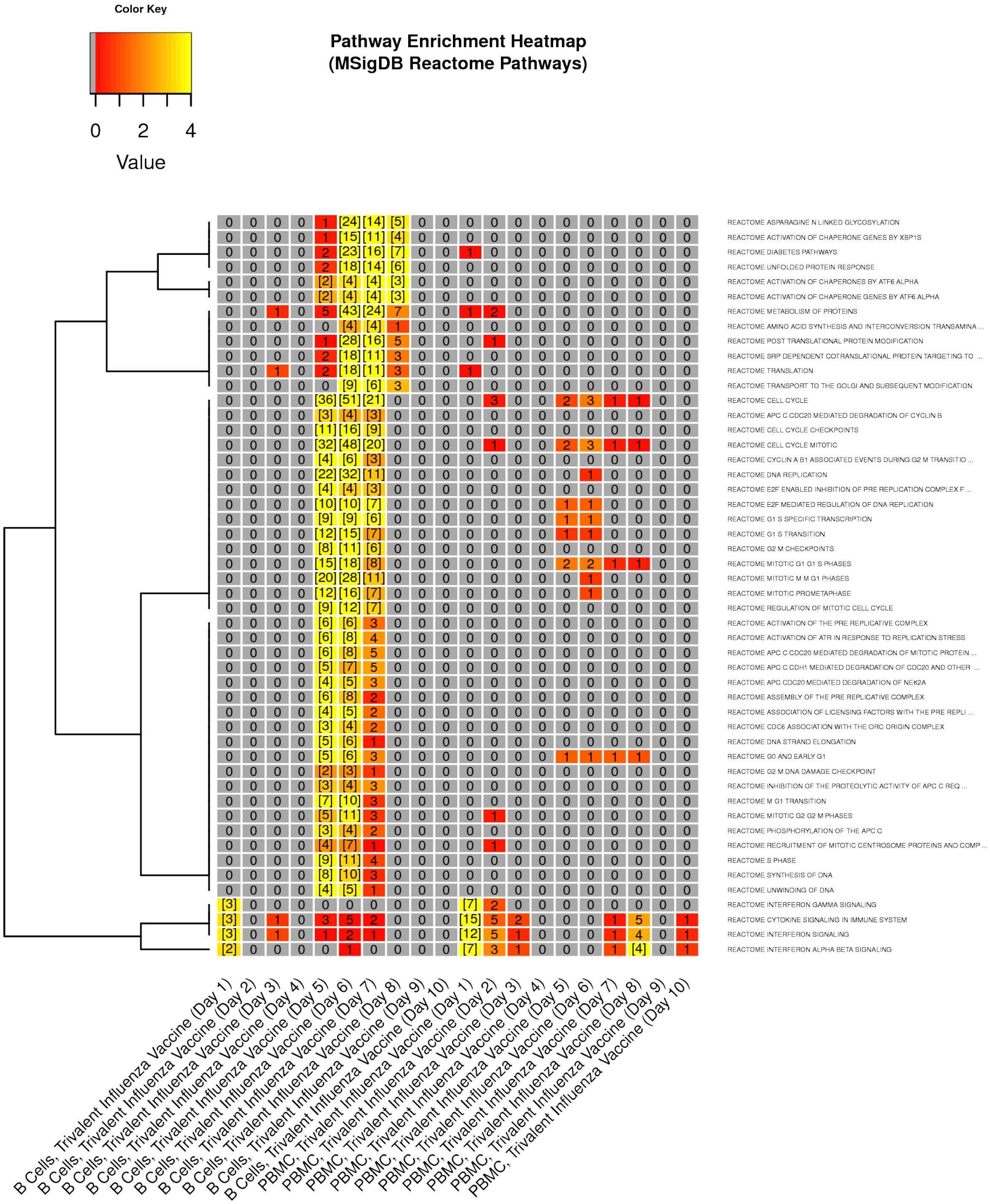

Figure 119: Heatmap of enriched MSigDB Reactome Pathways (RNA-Seq). Gene sets significantly enriched in at least two conditions are shown. Cells are color-coded by the enrichment score (-1 x log_{10}(FDR-adjusted p-value))). Cells contain the number of significant genes in the gene set with gene numbers in brackets indicating significantly enriched sets. Sets were clustered based on the euclidean distance between their enrichment score pattern. If there were more than 50 gene sets in the results, gene sets were further filtered to only include sets whose sum of enrichment score across conditions was the top 50 of all sets. Cells colored in grey have an enrichment score equal to zero.

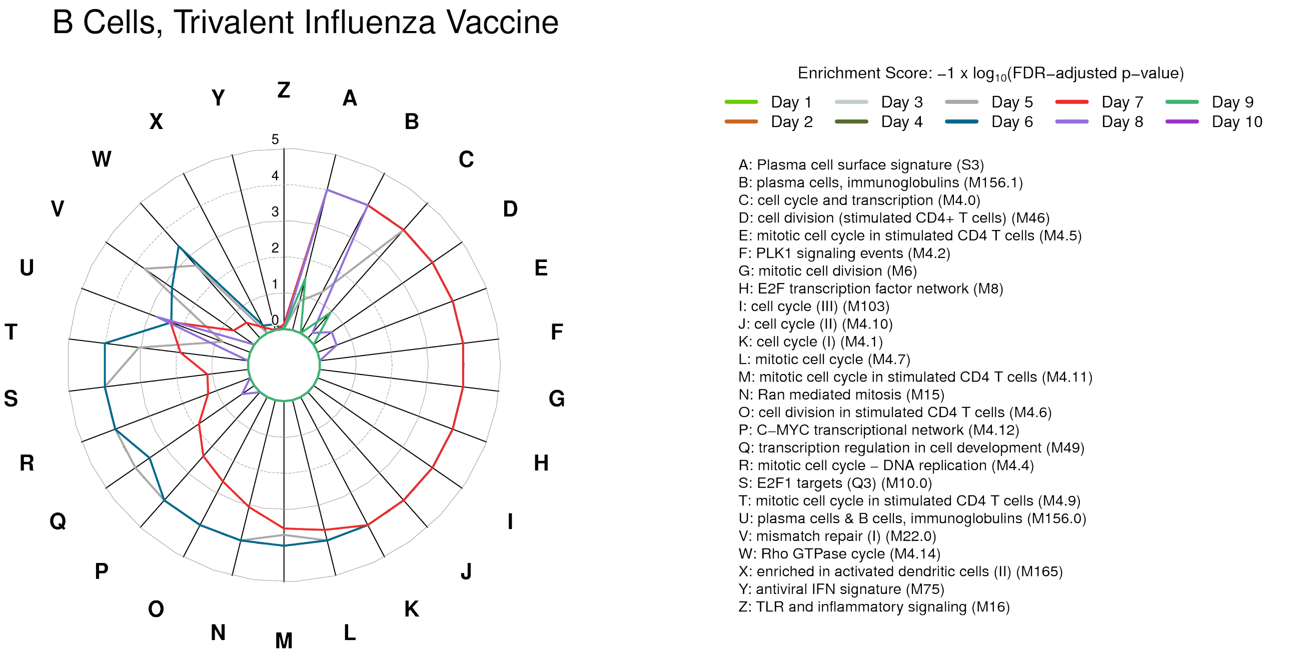

Figure 120: Radar Plot of enriched Blood Transcription Modules (B Cells, Trivalent Influenza Vaccine). Gene sets significantly enriched in at least two conditions are shown. If there were more than 26 gene sets in the results, gene sets were further filtered to only include sets whose enrichment score was the top 26 of all sets.

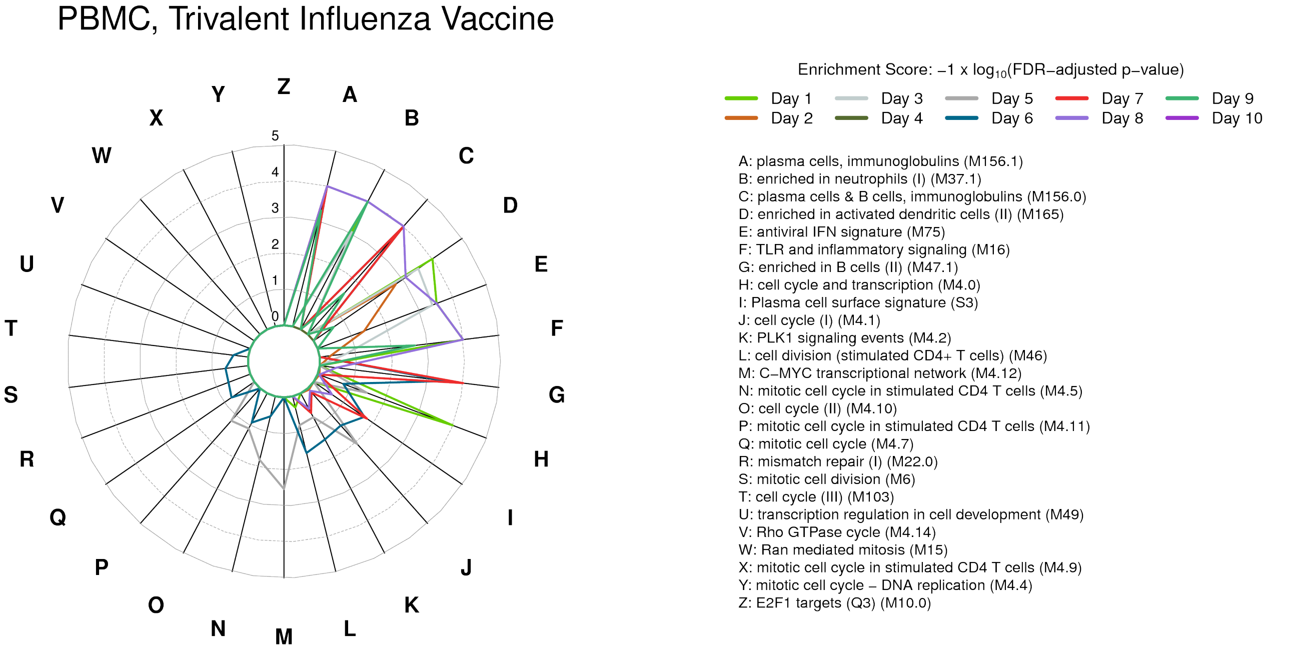

Figure 121: Radar Plot of enriched Blood Transcription Modules (PBMC, Trivalent Influenza Vaccine). Gene sets significantly enriched in at least two conditions are shown. If there were more than 26 gene sets in the results, gene sets were further filtered to only include sets whose enrichment score was the top 26 of all sets.

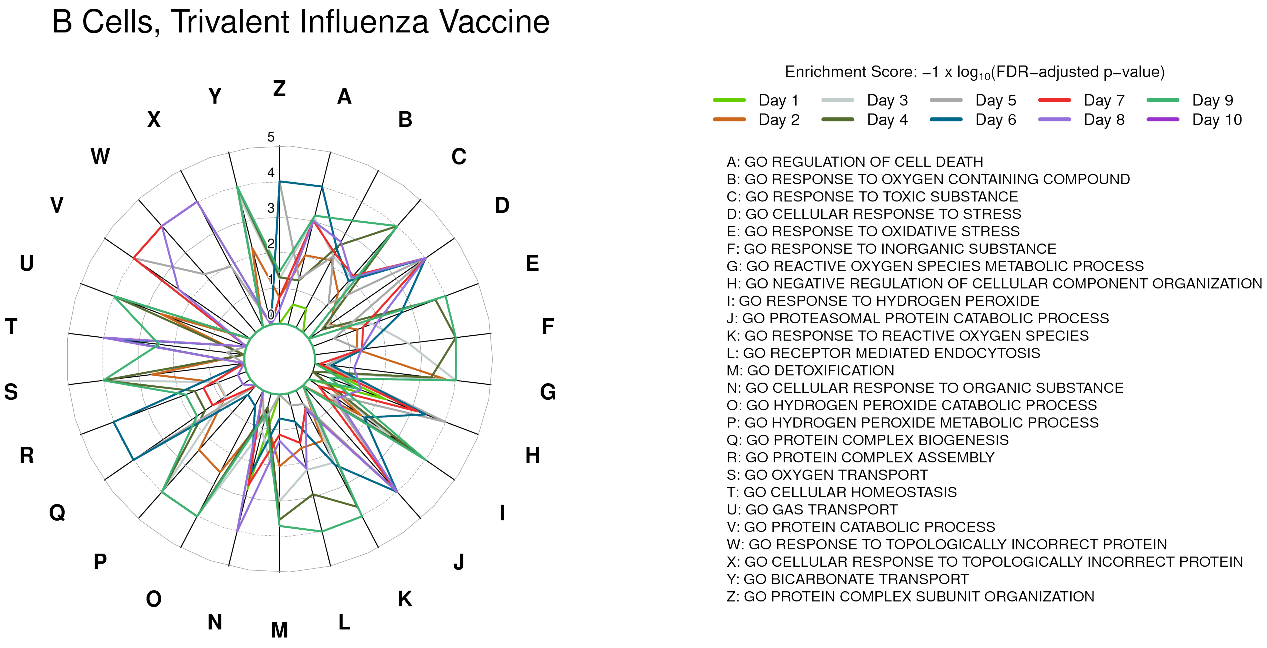

Figure 122: Radar Plot of enriched MSigDB Biological Processes (B Cells, Trivalent Influenza Vaccine). Gene sets significantly enriched in at least two conditions are shown. If there were more than 26 gene sets in the results, gene sets were further filtered to only include sets whose enrichment score was the top 26 of all sets.

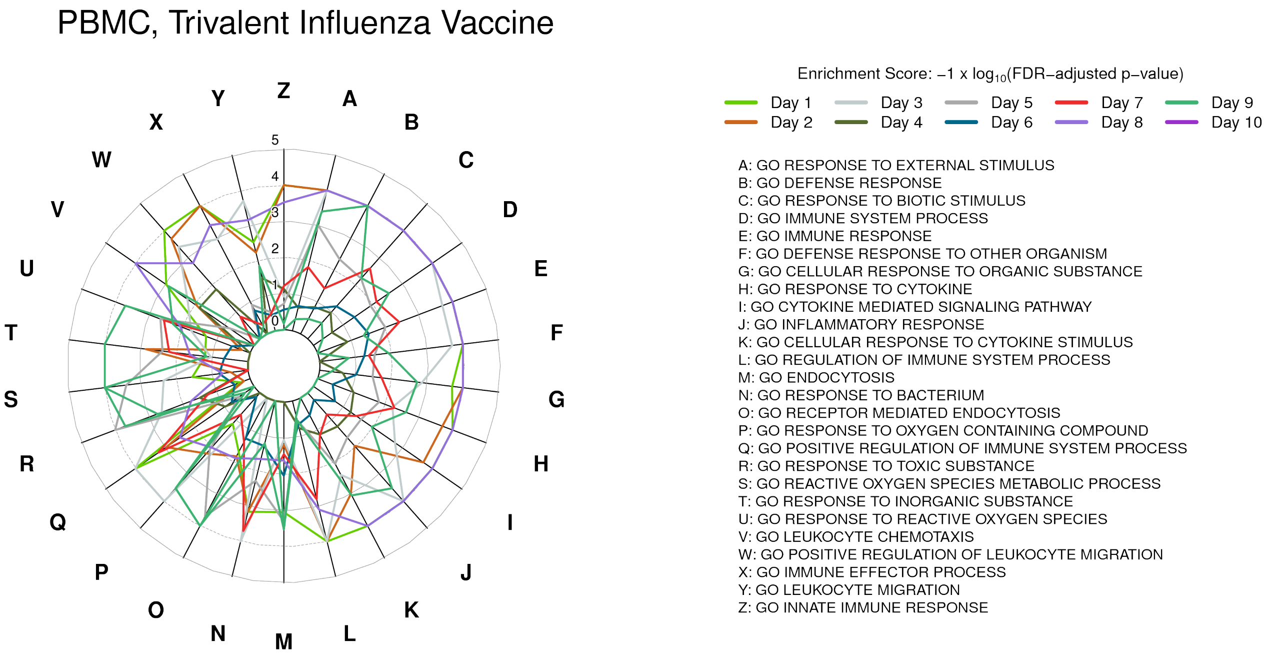

Figure 123: Radar Plot of enriched MSigDB Biological Processes (PBMC, Trivalent Influenza Vaccine). Gene sets significantly enriched in at least two conditions are shown. If there were more than 26 gene sets in the results, gene sets were further filtered to only include sets whose enrichment score was the top 26 of all sets.

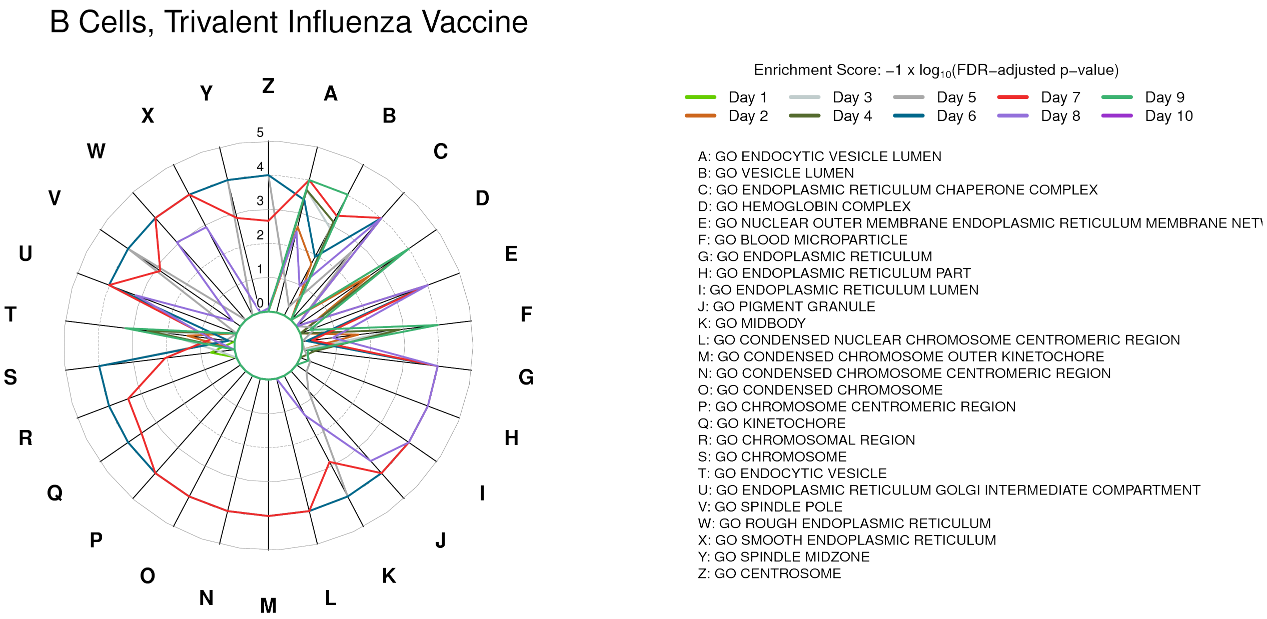

Figure 124: Radar Plot of enriched MSigDB Cellular Components (B Cells, Trivalent Influenza Vaccine). Gene sets significantly enriched in at least two conditions are shown. If there were more than 26 gene sets in the results, gene sets were further filtered to only include sets whose enrichment score was the top 26 of all sets.

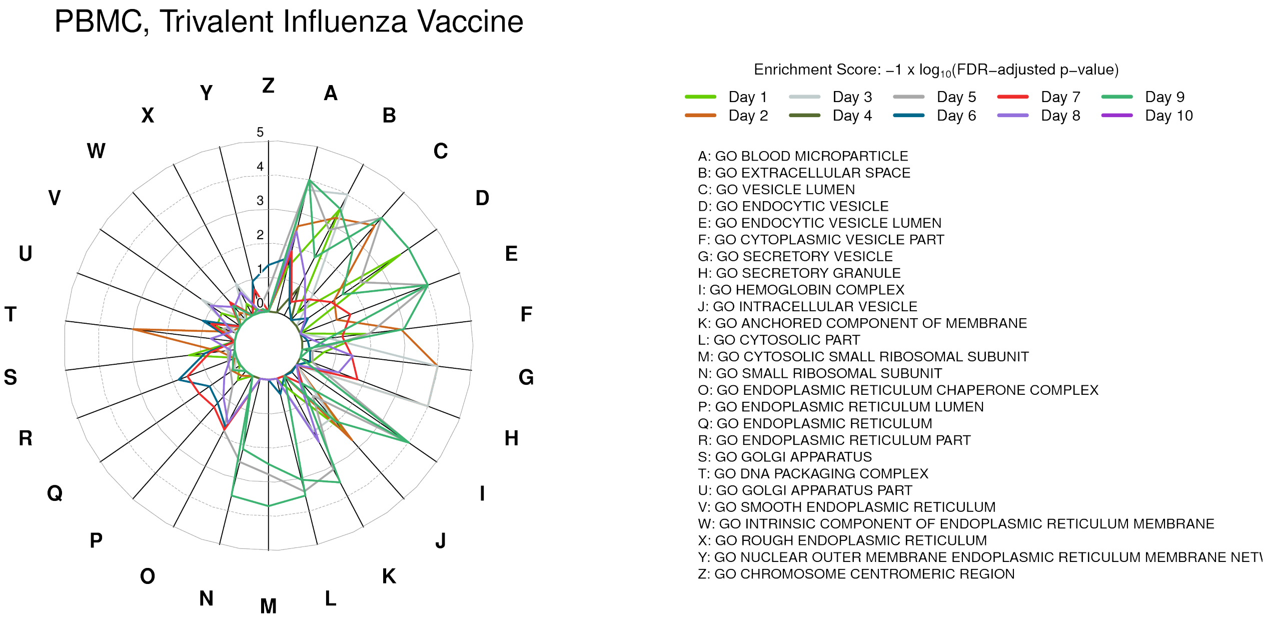

Figure 125: Radar Plot of enriched MSigDB Cellular Components (PBMC, Trivalent Influenza Vaccine). Gene sets significantly enriched in at least two conditions are shown. If there were more than 26 gene sets in the results, gene sets were further filtered to only include sets whose enrichment score was the top 26 of all sets.

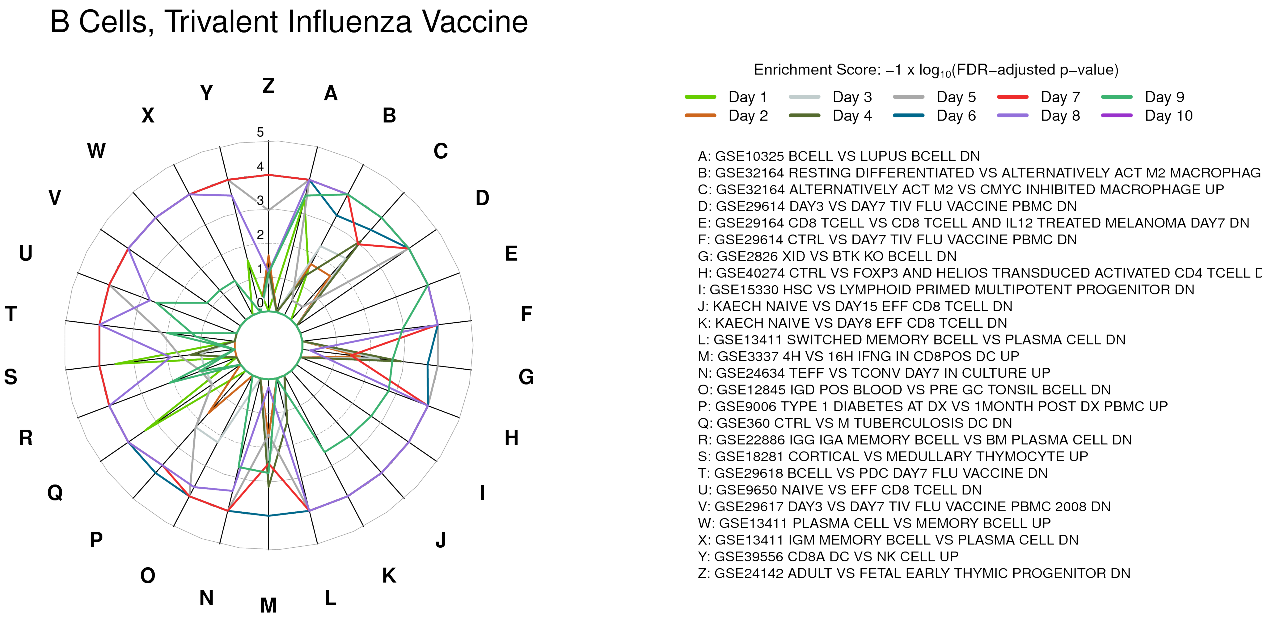

Figure 126: Radar Plot of enriched MSigDB Immunological Signatures (B Cells, Trivalent Influenza Vaccine). Gene sets significantly enriched in at least two conditions are shown. If there were more than 26 gene sets in the results, gene sets were further filtered to only include sets whose enrichment score was the top 26 of all sets.

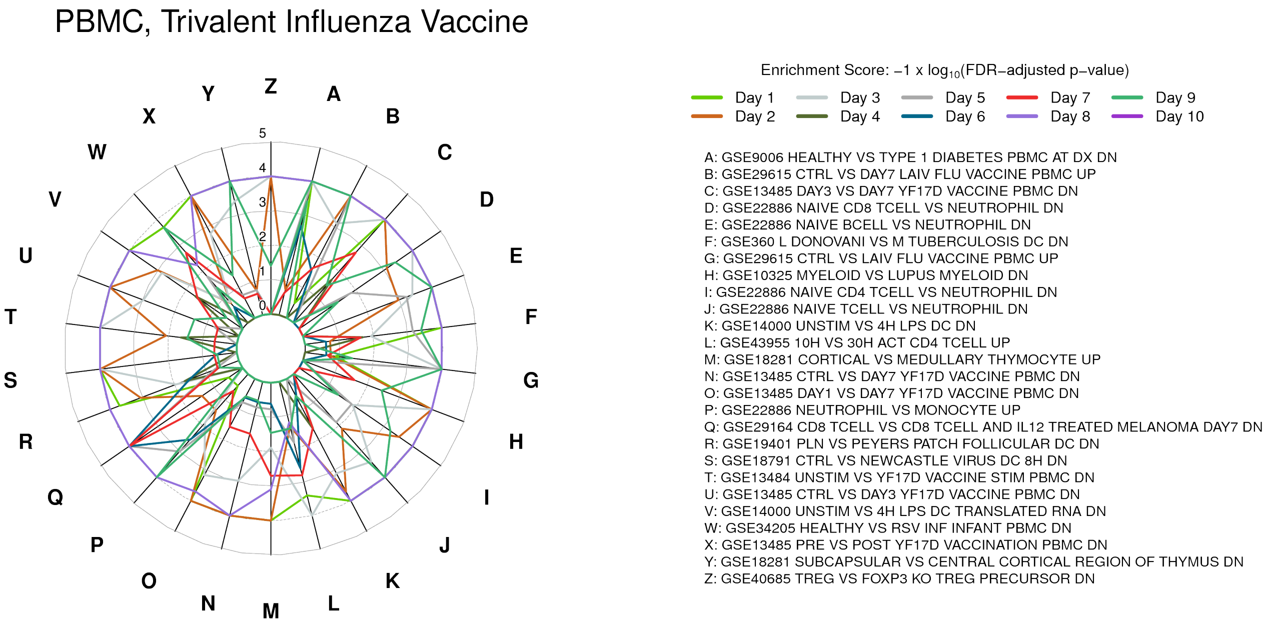

Figure 127: Radar Plot of enriched MSigDB Immunological Signatures (PBMC, Trivalent Influenza Vaccine). Gene sets significantly enriched in at least two conditions are shown. If there were more than 26 gene sets in the results, gene sets were further filtered to only include sets whose enrichment score was the top 26 of all sets.

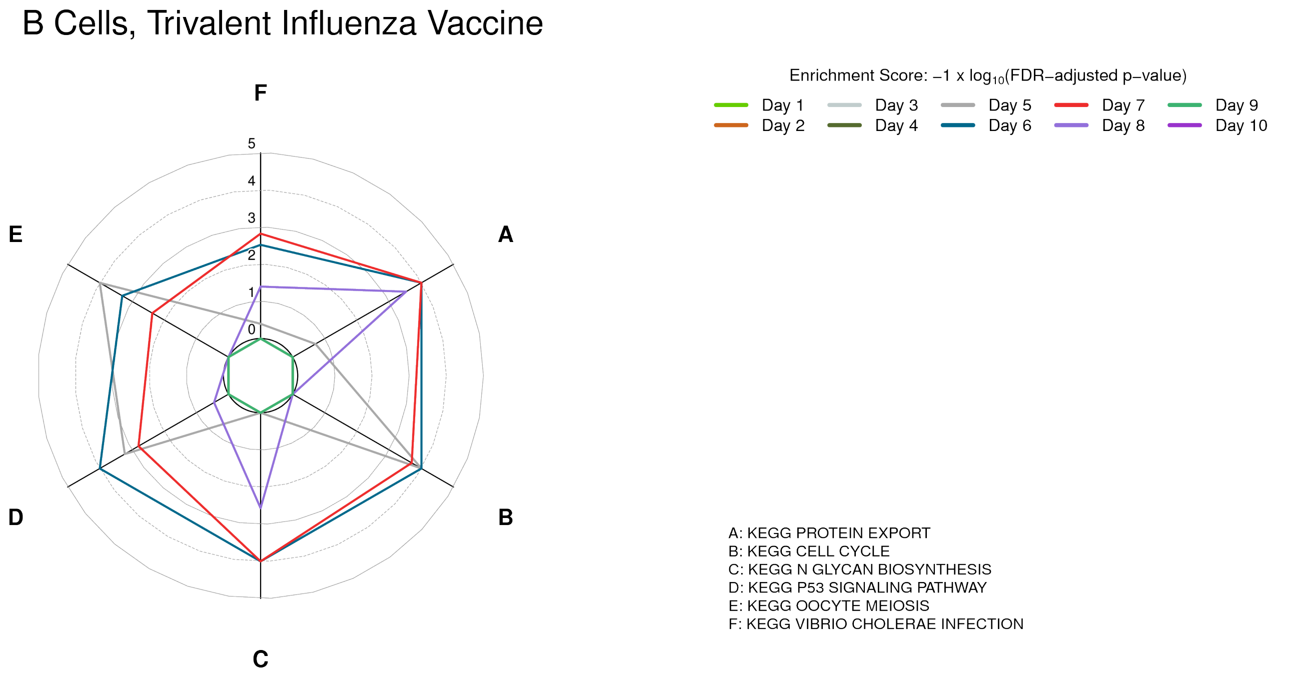

Figure 128: Radar Plot of enriched MSigDB KEGG Pathways (B Cells, Trivalent Influenza Vaccine). Gene sets significantly enriched in at least two conditions are shown. If there were more than 26 gene sets in the results, gene sets were further filtered to only include sets whose enrichment score was the top 26 of all sets.

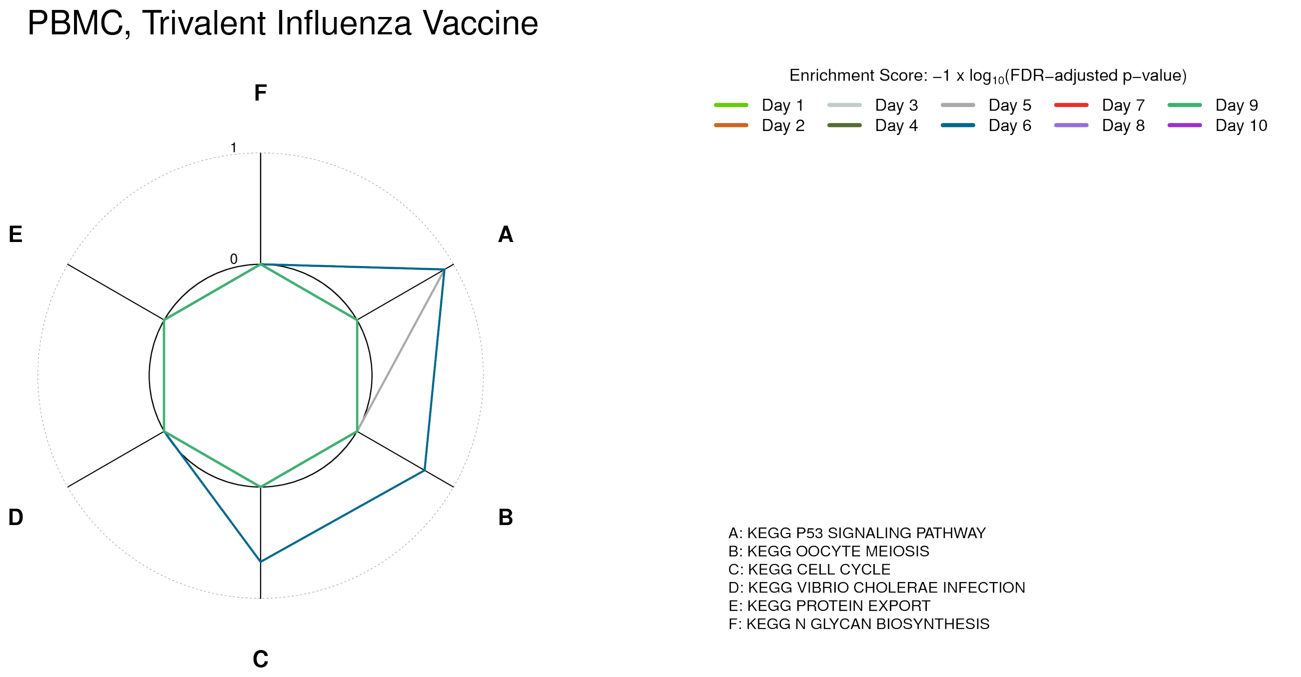

Figure 129: Radar Plot of enriched MSigDB KEGG Pathways (PBMC, Trivalent Influenza Vaccine). Gene sets significantly enriched in at least two conditions are shown. If there were more than 26 gene sets in the results, gene sets were further filtered to only include sets whose enrichment score was the top 26 of all sets.

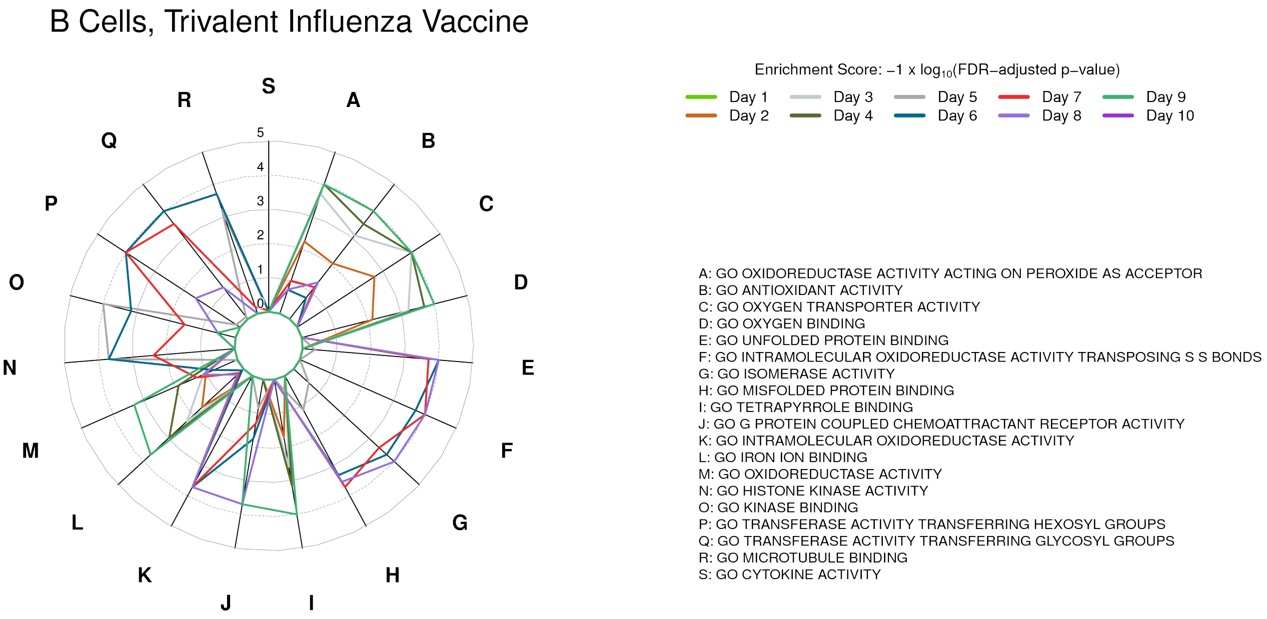

Figure 130: Radar Plot of enriched MSigDB Molecular Functions (B Cells, Trivalent Influenza Vaccine). Gene sets significantly enriched in at least two conditions are shown. If there were more than 26 gene sets in the results, gene sets were further filtered to only include sets whose enrichment score was the top 26 of all sets.

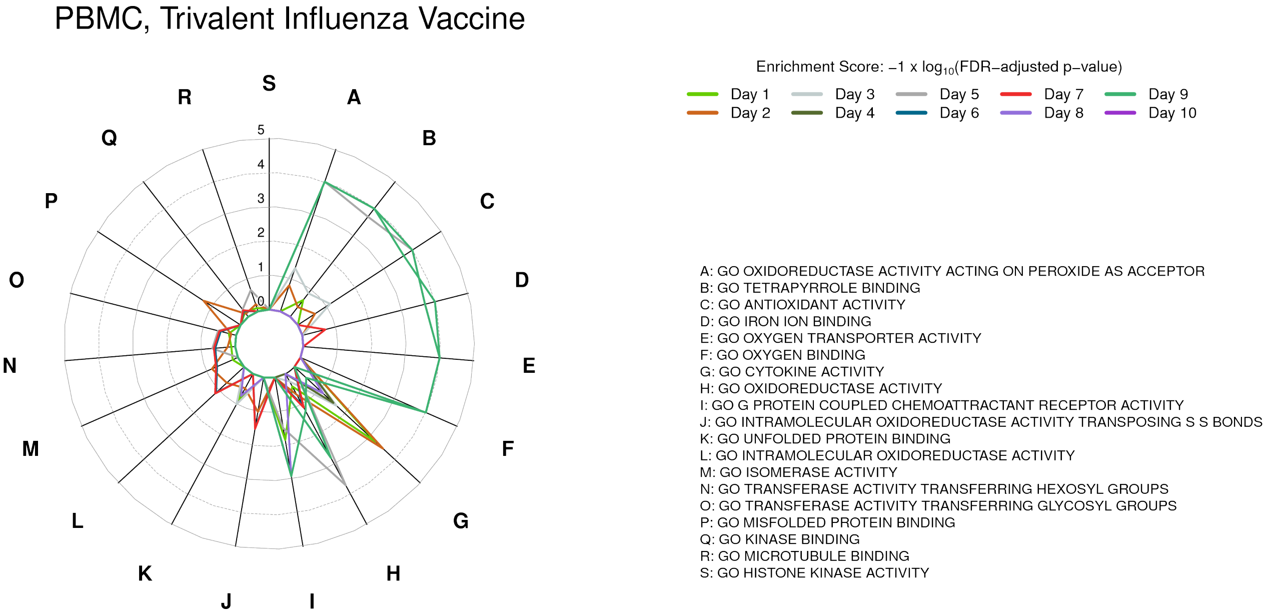

Figure 131: Radar Plot of enriched MSigDB Molecular Functions (PBMC, Trivalent Influenza Vaccine). Gene sets significantly enriched in at least two conditions are shown. If there were more than 26 gene sets in the results, gene sets were further filtered to only include sets whose enrichment score was the top 26 of all sets.

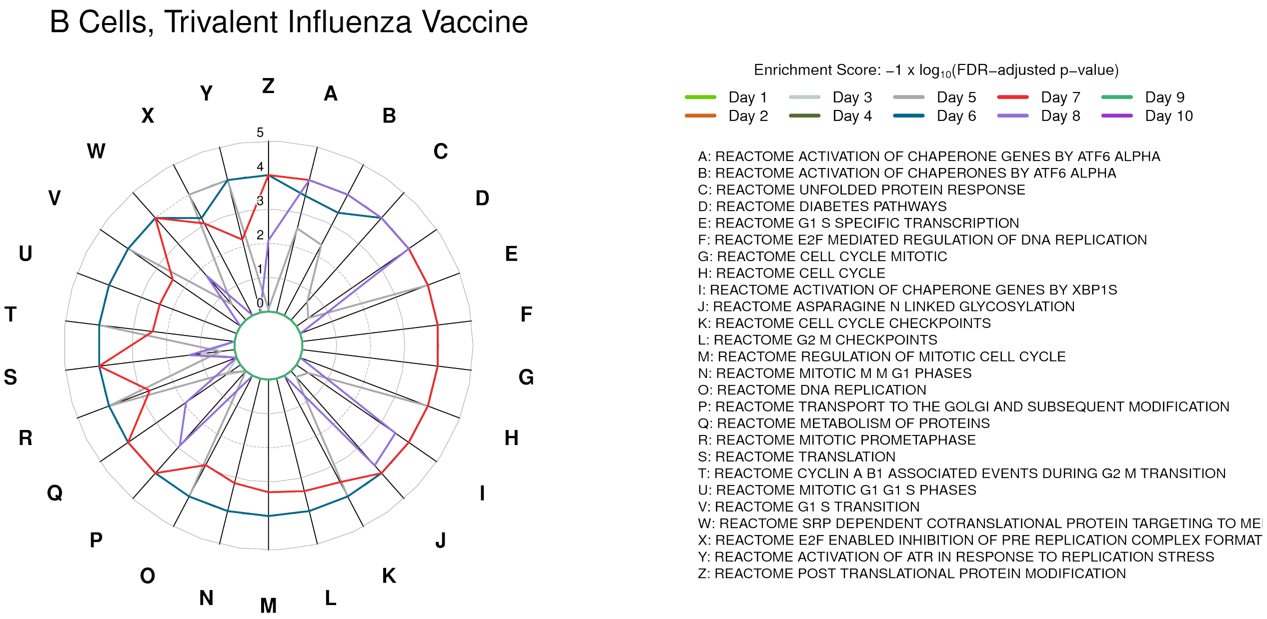

Figure 132: Radar Plot of enriched MSigDB Reactome Pathways (B Cells, Trivalent Influenza Vaccine). Gene sets significantly enriched in at least two conditions are shown. If there were more than 26 gene sets in the results, gene sets were further filtered to only include sets whose enrichment score was the top 26 of all sets.

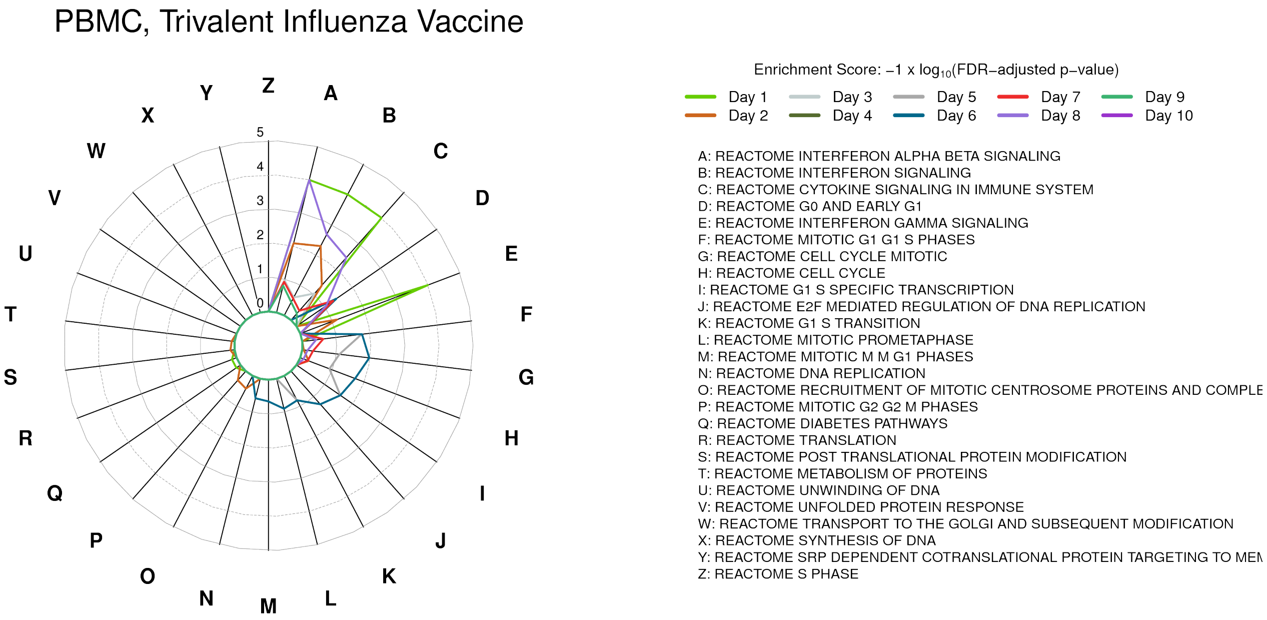

Figure 133: Radar Plot of enriched MSigDB Reactome Pathways (PBMC, Trivalent Influenza Vaccine). Gene sets significantly enriched in at least two conditions are shown. If there were more than 26 gene sets in the results, gene sets were further filtered to only include sets whose enrichment score was the top 26 of all sets.

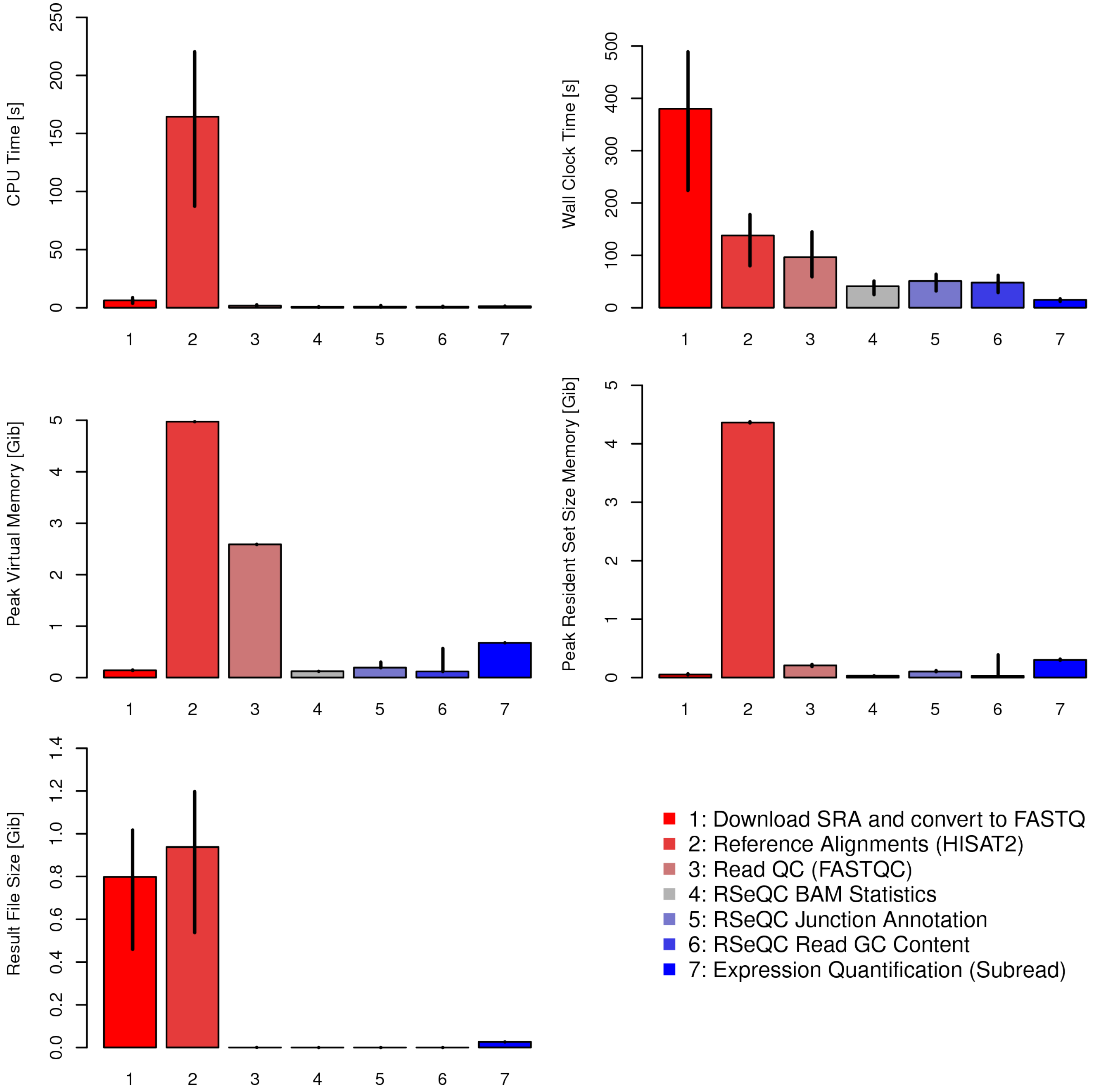

Figure 134: Summary of preprocessing benchmarks. Barplot height represents the median metric across samples. The vertical lines represent the range of the metric (minimum to maximum) across samples.

3.2 Tables

Category/Chromosome	#Genes
mitochondrial ribosomal RNA (Mt_rRNA)	2
mitochondrial transfer RNA (Mt_tRNA)	22
ribosomal RNA (rRNA)	543
Total	567

Table 1: Number of excluded genes by gene type (RNA-Seq).

	Min	Q1	Median	Mean	Q3	Max	SD	MAD	N
Total Mapped Reads [10^6]	7.43	10.97	12.37	12.25	13.50	16.08	1.88	1.75	110
Unmapped Reads [10^6]	0.78	1.20	1.42	1.48	1.69	2.60	0.41	0.35	110
Uniquely Mapped Reads [10^6]	6.71	9.97	11.23	11.09	12.23	14.58	1.75	1.67	110
Uniquely Mapped Reads [%]	57.50	90.20	91.00	90.54	91.50	92.60	3.33	0.89	110
Counted Fragments [10^6]	5.28	8.05	8.96	8.84	9.74	11.88	1.42	1.31	110
Uniquely Mapped Reads + Strand [10^6]	3.37	4.99	5.62	5.55	6.12	7.30	0.87	0.84	110
Uniquely Mapped Reads - Strand [10^6]	3.34	4.99	5.61	5.53	6.12	7.29	0.88	0.85	110
Median GC [%]	46.15	47.69	49.23	48.66	49.23	50.77	0.98	0.00	110
Mean GC [%]	46.12	48.26	48.62	48.66	49.12	50.52	0.73	0.66	110

Table 2: Summary human reference genome alignment statistics for study samples (All Specimen Types)

	Min	Q1	Median	Mean	Q3	Max	SD	MAD	N
Total Mapped Reads [10^6]	7.43	10.81	12.37	12.00	13.27	15.70	2.06	1.67	55
Unmapped Reads [10^6]	0.79	1.20	1.61	1.59	1.97	2.60	0.47	0.58	55
Uniquely Mapped Reads [10^6]	6.71	9.73	11.21	10.83	12.02	14.21	1.83	1.56	55
Uniquely Mapped Reads [%]	87.60	89.80	90.50	90.35	91.00	91.80	0.89	0.89	55
Counted Fragments [10^6]	5.28	7.67	8.76	8.53	9.48	11.26	1.45	1.17	55
Uniquely Mapped Reads + Strand [10^6]	3.37	4.88	5.62	5.43	6.01	7.12	0.91	0.79	55
Uniquely Mapped Reads - Strand [10^6]	3.34	4.84	5.59	5.40	6.00	7.09	0.92	0.79	55
Median GC [%]	46.15	47.69	49.23	48.67	49.23	50.77	1.08	0.00	55
Mean GC [%]	46.51	48.14	48.69	48.67	49.33	50.52	0.85	0.88	55

Table 3: Summary human reference genome alignment statistics for study samples (B Cells)

	Min	Q1	Median	Mean	Q3	Max	SD	MAD	N
Total Mapped Reads [10^6]	8.96	11.14	12.37	12.49	13.59	16.08	1.66	1.89	55
Unmapped Reads [10^6]	0.78	1.21	1.36	1.37	1.51	2.53	0.30	0.23	55
Uniquely Mapped Reads [10^6]	7.22	10.11	11.24	11.34	12.43	14.58	1.64	1.76	55
Uniquely Mapped Reads [%]	57.50	91.00	91.50	90.73	91.85	92.60	4.64	0.59	55
Counted Fragments [10^6]	5.53	8.13	9.06	9.15	10.03	11.88	1.33	1.40	55
Uniquely Mapped Reads + Strand [10^6]	3.63	5.06	5.62	5.67	6.22	7.30	0.82	0.89	55
Uniquely Mapped Reads - Strand [10^6]	3.59	5.05	5.62	5.66	6.21	7.29	0.82	0.87	55
Median GC [%]	46.15	47.69	49.23	48.64	49.23	50.77	0.86	0.00	55
Mean GC [%]	46.12	48.36	48.58	48.65	48.94	50.13	0.59	0.42	55

Table 4: Summary human reference genome alignment statistics for study samples (PBMC)

Subject ID	Sequence Library ID	Specimen Type	Timepoint
T13	T13_d08_pmc	PBMC	Day 8
T12	T12_d04_pmc	PBMC	Day 4

Table 5: Outlying observations (RNA-Seq).

	#Genes
B Cells	11435
PBMC	11511

Table 6: Number of genes that passed the low expression cut off (RNA-Seq).

Ensembl Gene ID	Ensembl Gene Name	Ensembl Gene Description	Gene Type	Log_2 Fold Change (Day 1 vs. pre-treatment)	Average Log_2 CPM	Likelihood Ratio Test Statistic	P-Value	FDR Adjusted P-Value
ENSG00000117228	GBP1	guanylate binding protein 1 [Source:HGNC Symbol;Acc:HGNC:4182]	protein coding	0.90	4.49	26.41	0.00	0.00
ENSG00000115415	STAT1	signal transducer and activator of transcription 1 [Source:HGNC Symbol;Acc:HGNC:11362]	protein coding	0.70	7.73	32.26	0.00	0.00
ENSG00000162645	GBP2	guanylate binding protein 2 [Source:HGNC Symbol;Acc:HGNC:4183]	protein coding	0.62	5.42	25.11	0.00	0.00

Table 7: Genes differentially expressed at Day 1 compared to pre-treatment (B Cells, Trivalent Influenza Vaccine). Sorted by descending absolute log_2 fold change (Day 1 vs. pre-treatment). Gene model summaries and annotations are based on Ensembl Version 87.

Ensembl Gene ID	Ensembl Gene Name	Ensembl Gene Description	Gene Type	Log_2 Fold Change (Day 2 vs. pre-treatment)	Average Log_2 CPM	Likelihood Ratio Test Statistic	P-Value	FDR Adjusted P-Value
ENSG00000004799	PDK4	pyruvate dehydrogenase kinase 4 [Source:HGNC Symbol;Acc:HGNC:8812]	protein coding	1.05	2.69	20.94	0.00	0.01
ENSG00000244734	HBB	hemoglobin subunit beta [Source:HGNC Symbol;Acc:HGNC:4827]	protein coding	-0.87	8.38	22.20	0.00	0.01

Table 8: Genes differentially expressed at Day 2 compared to pre-treatment (B Cells, Trivalent Influenza Vaccine). Sorted by descending absolute log_2 fold change (Day 2 vs. pre-treatment). Gene model summaries and annotations are based on Ensembl Version 87.

Ensembl Gene ID	Ensembl Gene Name	Ensembl Gene Description	Gene Type	Log_2 Fold Change (Day 3 vs. pre-treatment)	Average Log_2 CPM	Likelihood Ratio Test Statistic	P-Value	FDR Adjusted P-Value
ENSG00000188536	HBA2	hemoglobin subunit alpha 2 [Source:HGNC Symbol;Acc:HGNC:4824]	protein coding	-1.70	4.95	13.32	0.00	0.03
ENSG00000244734	HBB	hemoglobin subunit beta [Source:HGNC Symbol;Acc:HGNC:4827]	protein coding	-1.67	8.41	19.52	0.00	0.00
ENSG00000168209	DDIT4	DNA damage inducible transcript 4 [Source:HGNC Symbol;Acc:HGNC:24944]	protein coding	-1.00	4.52	36.43	0.00	0.00
ENSG00000110848	CD69	CD69 molecule [Source:HGNC Symbol;Acc:HGNC:1694]	protein coding	-0.85	6.70	24.54	0.00	0.00
ENSG00000197714	ZNF460	zinc finger protein 460 [Source:HGNC Symbol;Acc:HGNC:21628]	protein coding	-0.80	3.05	23.65	0.00	0.00
ENSG00000171223	JUNB	JunB proto-oncogene, AP-1 transcription factor subunit [Source:HGNC Symbol;Acc:HGNC:6205]	protein coding	-0.76	7.51	36.78	0.00	0.00
ENSG00000181800	CELF2-AS1	CELF2 antisense RNA 1 [Source:HGNC Symbol;Acc:HGNC:23515]	antisense	-0.75	3.08	22.69	0.00	0.00
ENSG00000279192	PWAR5	Prader Willi/Angelman region RNA 5 [Source:HGNC Symbol;Acc:HGNC:30090]	TEC	-0.70	3.24	23.42	0.00	0.00
ENSG00000144182	LIPT1	lipoyltransferase 1 [Source:HGNC Symbol;Acc:HGNC:29569]	protein coding	0.63	2.86	14.29	0.00	0.02

Ensembl Gene ID	Ensembl Gene Name	Ensembl Gene Description	Gene Type	Log_2 Fold Change (Day 3 vs. pre-treatment)	Average Log_2 CPM	Likelihood Ratio Test Statistic	P-Value	FDR Adjusted P-Value
ENSG00000100906	NFKBIA	NFKB inhibitor alpha [Source:HGNC Symbol;Acc:HGNC:7797]	protein coding	-0.63	7.41	40.23	0.00	0.00
ENSG00000181722	ZBTB20	zinc finger and BTB domain containing 20 [Source:HGNC Symbol;Acc:HGNC:13503]	protein coding	-0.62	5.00	36.87	0.00	0.00
ENSG00000279759			TEC	-0.60	2.81	11.98	0.00	0.05
ENSG00000280138			TEC	-0.60	5.43	38.18	0.00	0.00
ENSG00000161960	EIF4A1	eukaryotic translation initiation factor 4A1 [Source:HGNC Symbol;Acc:HGNC:3282]	protein coding	-0.60	4.84	17.25	0.00	0.01
ENSG00000227155			antisense	-0.59	3.77	19.96	0.00	0.00

Table 9: Genes differentially expressed at Day 3 compared to pre-treatment (B Cells, Trivalent Influenza Vaccine). Sorted by descending absolute log_2 fold change (Day 3 vs. pre-treatment). Gene model summaries and annotations are based on Ensembl Version 87.

Ensembl Gene ID	Ensembl Gene Name	Ensembl Gene Description	Gene Type	Log_2 Fold Change (Day 4 vs. pre-treatment)	Average Log_2 CPM	Likelihood Ratio Test Statistic	P-Value	FDR Adjusted P-Value
ENSG00000244734	HBB	hemoglobin subunit beta [Source:HGNC Symbol;Acc:HGNC:4827]	protein coding	-1.37	8.12	36.17	0.00	0.00
ENSG00000188536	HBA2	hemoglobin subunit alpha 2 [Source:HGNC Symbol;Acc:HGNC:4824]	protein coding	-1.31	4.69	24.05	0.00	0.00
ENSG00000149516	MS4A3	membrane spanning 4-domains A3 [Source:HGNC Symbol;Acc:HGNC:7317]	protein coding	1.02	4.86	24.57	0.00	0.00
ENSG00000170345	FOS	Fos proto-oncogene, AP-1 transcription factor subunit [Source:HGNC Symbol;Acc:HGNC:3796]	protein coding	1.00	6.11	21.42	0.00	0.01
ENSG00000197714	ZNF460	zinc finger protein 460 [Source:HGNC Symbol;Acc:HGNC:21628]	protein coding	-0.76	3.04	20.37	0.00	0.01

Table 10: Genes differentially expressed at Day 4 compared to pre-treatment (B Cells, Trivalent Influenza Vaccine). Sorted by descending absolute log_2 fold change (Day 4 vs. pre-treatment). Gene model summaries and annotations are based on Ensembl Version 87.

Ensembl Gene ID	Ensembl Gene Name	Ensembl Gene Description	Gene Type	Log_2 Fold Change (Day 5 vs. pre-treatment)	Average Log_2 CPM	Likelihood Ratio Test Statistic	P-Value	FDR Adjusted P-Value
ENSG00000153093	ACOXL	acyl-CoA oxidase-like [Source:HGNC Symbol;Acc:HGNC:25621]	protein coding	2.53	1.44	18.85	0.00	0.01
ENSG00000168078	PBK	PDZ binding kinase [Source:HGNC Symbol;Acc:HGNC:18282]	protein coding	2.09	1.58	17.56	0.00	0.01
ENSG00000093009	CDC45	cell division cycle 45 [Source:HGNC Symbol;Acc:HGNC:1739]	protein coding	2.05	2.61	23.15	0.00	0.00
ENSG00000139734	DIAPH3	diaphanous related formin 3 [Source:HGNC Symbol;Acc:HGNC:15480]	protein coding	2.03	1.83	16.05	0.00	0.02

Ensembl Gene ID	Ensembl Gene Name	Ensembl Gene Description	Gene Type	Log_2 Fold Change (Day 5 vs. pre-treatment)	Average Log_2 CPM	Likelihood Ratio Test Statistic	P-Value	FDR Adjusted P-Value
ENSG00000165304	MELK	maternal embryonic leucine zipper kinase [Source:HGNC Symbol;Acc:HGNC:16870]	protein coding	1.89	2.29	15.19	0.00	0.02
ENSG00000115163	CENPA	centromere protein A [Source:HGNC Symbol;Acc:HGNC:1851]	protein coding	1.89	1.57	13.41	0.00	0.03
ENSG00000146670	CDCA5	cell division cycle associated 5 [Source:HGNC Symbol;Acc:HGNC:14626]	protein coding	1.86	3.42	29.57	0.00	0.00
ENSG00000123485	HJURP	Holliday junction recognition protein [Source:HGNC Symbol;Acc:HGNC:25444]	protein coding	1.85	2.91	20.98	0.00	0.00
ENSG00000154277	UCHL1	ubiquitin C-terminal hydrolase L1 [Source:HGNC Symbol;Acc:HGNC:12513]	protein coding	1.85	1.44	16.74	0.00	0.01
ENSG00000176890	TYMS	thymidylate synthetase [Source:HGNC Symbol;Acc:HGNC:12441]	protein coding	1.82	3.69	30.21	0.00	0.00
ENSG00000184661	CDCA2	cell division cycle associated 2 [Source:HGNC Symbol;Acc:HGNC:14623]	protein coding	1.82	2.39	14.38	0.00	0.02
ENSG00000171848	RRM2	ribonucleotide reductase regulatory subunit M2 [Source:HGNC Symbol;Acc:HGNC:10452]	protein coding	1.80	6.11	25.19	0.00	0.00
ENSG00000011426	ANLN	anillin actin binding protein [Source:HGNC Symbol;Acc:HGNC:14082]	protein coding	1.79	2.27	15.13	0.00	0.02
ENSG00000164045	CDC25A	cell division cycle 25A [Source:HGNC Symbol;Acc:HGNC:1725]	protein coding	1.71	2.29	13.41	0.00	0.03
ENSG00000149516	MS4A3	membrane spanning 4-domains A3 [Source:HGNC Symbol;Acc:HGNC:7317]	protein coding	1.68	4.70	21.82	0.00	0.00
ENSG00000143476	DTL	denticleless E3 ubiquitin protein ligase homolog [Source:HGNC Symbol;Acc:HGNC:30288]	protein coding	1.68	3.41	21.67	0.00	0.00
ENSG00000186185	KIF18B	kinesin family member 18B [Source:HGNC Symbol;Acc:HGNC:27102]	protein coding	1.66	2.23	12.58	0.00	0.04
ENSG00000089685	BIRC5	baculoviral IAP repeat containing 5 [Source:HGNC Symbol;Acc:HGNC:593]	protein coding	1.65	3.53	17.13	0.00	0.01
ENSG00000169679	BUB1	BUB1 mitotic checkpoint serine/threonine kinase [Source:HGNC Symbol;Acc:HGNC:1148]	protein coding	1.63	5.12	24.34	0.00	0.00
ENSG00000109805	NCAPG	non-SMC condensin I complex subunit G [Source:HGNC Symbol;Acc:HGNC:24304]	protein coding	1.63	3.63	22.85	0.00	0.00
ENSG00000117399	CDC20	cell division cycle 20 [Source:HGNC Symbol;Acc:HGNC:1723]	protein coding	1.60	3.92	17.00	0.00	0.01
ENSG00000122952	ZWINT	ZW10 interacting kinetochore protein [Source:HGNC Symbol;Acc:HGNC:13195]	protein coding	1.60	3.84	18.55	0.00	0.01
ENSG00000211896	IGHG1	immunoglobulin heavy constant gamma 1 (G1m marker) [Source:HGNC Symbol;Acc:HGNC:5525]	IG C gene	1.59	12.44	20.69	0.00	0.00
ENSG00000105974	CAV1	caveolin 1 [Source:HGNC Symbol;Acc:HGNC:1527]	protein coding	1.56	4.30	18.47	0.00	0.01
ENSG00000126787	DLGAP5	DLG associated protein 5 [Source:HGNC Symbol;Acc:HGNC:16864]	protein coding	1.54	3.47	16.02	0.00	0.02
ENSG00000171241	SHCBP1	SHC binding and spindle associated 1 [Source:HGNC Symbol;Acc:HGNC:29547]	protein coding	1.54	4.95	18.15	0.00	0.01
ENSG00000071539	TRIP13	thyroid hormone receptor interactor 13 [Source:HGNC Symbol;Acc:HGNC:12307]	protein coding	1.52	3.03	15.85	0.00	0.02

Ensembl Gene ID	Ensembl Gene Name	Ensembl Gene Description	Gene Type	Log_2 Fold Change (Day 5 vs. pre-treatment)	Average Log_2 CPM	Likelihood Ratio Test Statistic	P-Value	FDR Adjusted P-Value
ENSG00000174371	EXO1	exonuclease 1 [Source:HGNC Symbol;Acc:HGNC:3511]	protein coding	1.52	2.31	15.01	0.00	0.02
ENSG00000137807	KIF23	kinesin family member 23 [Source:HGNC Symbol;Acc:HGNC:6392]	protein coding	1.52	2.71	18.63	0.00	0.01
ENSG00000169607	CKAP2L	cytoskeleton associated protein 2 like [Source:HGNC Symbol;Acc:HGNC:26877]	protein coding	1.52	1.97	14.81	0.00	0.02
ENSG00000119326	CTNNAL1	catenin alpha like 1 [Source:HGNC Symbol;Acc:HGNC:2512]	protein coding	1.49	2.76	15.39	0.00	0.02
ENSG00000085840	ORC1	origin recognition complex subunit 1 [Source:HGNC Symbol;Acc:HGNC:8487]	protein coding	1.47	2.84	14.07	0.00	0.03
ENSG00000088325	TPX2	TPX2, microtubule nucleation factor [Source:HGNC Symbol;Acc:HGNC:1249]	protein coding	1.46	4.23	18.74	0.00	0.01
ENSG00000170312	CDK1	cyclin dependent kinase 1 [Source:HGNC Symbol;Acc:HGNC:1722]	protein coding	1.45	3.27	15.96	0.00	0.02
ENSG00000178999	AURKB	aurora kinase B [Source:HGNC Symbol;Acc:HGNC:11390]	protein coding	1.45	2.84	15.37	0.00	0.02
ENSG00000101057	MYBL2	MYB proto-oncogene like 2 [Source:HGNC Symbol;Acc:HGNC:7548]	protein coding	1.44	6.48	15.57	0.00	0.02
ENSG00000094804	CDC6	cell division cycle 6 [Source:HGNC Symbol;Acc:HGNC:1744]	protein coding	1.44	3.72	15.34	0.00	0.02
ENSG00000075218	GTSE1	G2 and S-phase expressed 1 [Source:HGNC Symbol;Acc:HGNC:13698]	protein coding	1.43	2.89	13.17	0.00	0.03
ENSG00000253755	IGHGP	immunoglobulin heavy constant gamma P (non-functional) [Source:HGNC Symbol;Acc:HGNC:5529]	IG C pseudogene	1.43	7.45	20.43	0.00	0.00
ENSG00000087586	AURKA	aurora kinase A [Source:HGNC Symbol;Acc:HGNC:11393]	protein coding	1.42	2.84	18.78	0.00	0.01
ENSG00000148773	MKI67	marker of proliferation Ki-67 [Source:HGNC Symbol;Acc:HGNC:7107]	protein coding	1.42	6.88	15.63	0.00	0.02
ENSG00000029993	HMGB3	high mobility group box 3 [Source:HGNC Symbol;Acc:HGNC:5004]	protein coding	1.42	3.06	13.99	0.00	0.03
ENSG00000138166	DUSP5	dual specificity phosphatase 5 [Source:HGNC Symbol;Acc:HGNC:3071]	protein coding	1.41	5.25	16.23	0.00	0.02
ENSG00000163751	CPA3	carboxypeptidase A3 [Source:HGNC Symbol;Acc:HGNC:2298]	protein coding	1.41	5.08	18.55	0.00	0.01
ENSG00000138778	CENPE	centromere protein E [Source:HGNC Symbol;Acc:HGNC:1856]	protein coding	1.38	3.96	16.30	0.00	0.02
ENSG00000135476	ESPL1	extra spindle pole bodies like 1, separase [Source:HGNC Symbol;Acc:HGNC:16856]	protein coding	1.38	3.36	13.50	0.00	0.03
ENSG00000167900	TK1	thymidine kinase 1 [Source:HGNC Symbol;Acc:HGNC:11830]	protein coding	1.38	4.47	20.55	0.00	0.00
ENSG00000117650	NEK2	NIMA related kinase 2 [Source:HGNC Symbol;Acc:HGNC:7745]	protein coding	1.37	2.09	12.30	0.00	0.04
ENSG00000121807	CCR2	C-C motif chemokine receptor 2 [Source:HGNC Symbol;Acc:HGNC:1603]	protein coding	1.37	6.10	14.24	0.00	0.02
ENSG00000162676	GFI1	growth factor independent 1 transcriptional repressor [Source:HGNC Symbol;Acc:HGNC:4237]	protein coding	1.36	2.53	12.48	0.00	0.04

Ensembl Gene ID	Ensembl Gene Name	Ensembl Gene Description	Gene Type	Log_2 Fold Change (Day 5 vs. pre-treatment)	Average Log_2 CPM	Likelihood Ratio Test Statistic	P-Value	FDR Adjusted P-Value
ENSG00000111206	FOXM1	forkhead box M1 [Source:HGNC Symbol;Acc:HGNC:3818]	protein coding	1.36	3.70	19.23	0.00	0.01
ENSG00000121152	NCAPH	non-SMC condensin I complex subunit H [Source:HGNC Symbol;Acc:HGNC:1112]	protein coding	1.34	3.11	13.05	0.00	0.03
ENSG00000137563	GGH	gamma-glutamyl hydrolase [Source:HGNC Symbol;Acc:HGNC:4248]	protein coding	1.34	4.11	15.18	0.00	0.02
ENSG00000138180	CEP55	centrosomal protein 55 [Source:HGNC Symbol;Acc:HGNC:1161]	protein coding	1.31	2.88	11.97	0.00	0.05
ENSG00000035499	DEPDC1B	DEP domain containing 1B [Source:HGNC Symbol;Acc:HGNC:24902]	protein coding	1.30	2.64	14.52	0.00	0.02
ENSG00000072571	HMMR	hyaluronan mediated motility receptor [Source:HGNC Symbol;Acc:HGNC:5012]	protein coding	1.29	3.11	14.49	0.00	0.02
ENSG00000167513	CDT1	chromatin licensing and DNA replication factor 1 [Source:HGNC Symbol;Acc:HGNC:24576]	protein coding	1.28	2.73	12.83	0.00	0.04
ENSG00000025039	RRAGD	Ras related GTP binding D [Source:HGNC Symbol;Acc:HGNC:19903]	protein coding	1.27	2.97	19.13	0.00	0.01
ENSG00000107104	KANK1	KN motif and ankyrin repeat domains 1 [Source:HGNC Symbol;Acc:HGNC:19309]	protein coding	1.27	3.08	13.04	0.00	0.03
ENSG00000164611	PTTG1	pituitary tumor-transforming 1 [Source:HGNC Symbol;Acc:HGNC:9690]	protein coding	1.26	3.82	14.25	0.00	0.02
ENSG00000076382	SPAG5	sperm associated antigen 5 [Source:HGNC Symbol;Acc:HGNC:13452]	protein coding	1.24	4.19	13.88	0.00	0.03
ENSG00000151725	CENPU	centromere protein U [Source:HGNC Symbol;Acc:HGNC:21348]	protein coding	1.22	3.37	17.31	0.00	0.01
ENSG00000131747	TOP2A	topoisomerase (DNA) II alpha [Source:HGNC Symbol;Acc:HGNC:11989]	protein coding	1.22	5.39	16.49	0.00	0.01
ENSG00000105011	ASF1B	anti-silencing function 1B histone chaperone [Source:HGNC Symbol;Acc:HGNC:20996]	protein coding	1.21	3.46	13.01	0.00	0.03
ENSG00000134489	HRH4	histamine receptor H4 [Source:HGNC Symbol;Acc:HGNC:17383]	protein coding	1.21	3.55	14.74	0.00	0.02
ENSG00000134057	CCNB1	cyclin B1 [Source:HGNC Symbol;Acc:HGNC:1579]	protein coding	1.19	3.71	16.21	0.00	0.02
ENSG00000100219	XBP1	X-box binding protein 1 [Source:HGNC Symbol;Acc:HGNC:12801]	protein coding	1.18	8.71	14.15	0.00	0.02
ENSG00000128228	SDF2L1	stromal cell derived factor 2 like 1 [Source:HGNC Symbol;Acc:HGNC:10676]	protein coding	1.18	5.35	14.98	0.00	0.02
ENSG00000131153	GINS2	GINS complex subunit 2 [Source:HGNC Symbol;Acc:HGNC:24575]	protein coding	1.16	2.63	12.32	0.00	0.04
ENSG00000138160	KIF11	kinesin family member 11 [Source:HGNC Symbol;Acc:HGNC:6388]	protein coding	1.14	4.90	19.42	0.00	0.01
ENSG00000166851	PLK1	polo like kinase 1 [Source:HGNC Symbol;Acc:HGNC:9077]	protein coding	1.09	3.76	15.64	0.00	0.02
ENSG00000143228	NUF2	NUF2, NDC80 kinetochore complex component [Source:HGNC Symbol;Acc:HGNC:14621]	protein coding	1.09	2.80	12.68	0.00	0.04
ENSG00000173848	NET1	neuroepithelial cell transforming 1 [Source:HGNC Symbol;Acc:HGNC:14592]	protein coding	1.09	4.51	13.46	0.00	0.03

Ensembl Gene ID	Ensembl Gene Name	Ensembl Gene Description	Gene Type	Log_2 Fold Change (Day 5 vs. pre-treatment)	Average Log_2 CPM	Likelihood Ratio Test Statistic	P-Value	FDR Adjusted P-Value
ENSG00000166598	HSP90B1	heat shock protein 90 beta family member 1 [Source:HGNC Symbol;Acc:HGNC:12028]	protein coding	1.07	10.85	12.45	0.00	0.04
ENSG00000104738	MCM4	minichromosome maintenance complex component 4 [Source:HGNC Symbol;Acc:HGNC:6947]	protein coding	1.07	5.57	20.28	0.00	0.00
ENSG00000164109	MAD2L1	MAD2 mitotic arrest deficient-like 1 (yeast) [Source:HGNC Symbol;Acc:HGNC:6763]	protein coding	1.06	3.47	19.34	0.00	0.01
ENSG00000145050	MANF	mesencephalic astrocyte derived neurotrophic factor [Source:HGNC Symbol;Acc:HGNC:15461]	protein coding	1.05	6.57	13.11	0.00	0.03
ENSG00000048462	TNFRSF17	TNF receptor superfamily member 17 [Source:HGNC Symbol;Acc:HGNC:11913]	protein coding	1.03	6.64	14.59	0.00	0.02
ENSG00000259706	HSP90B2P	heat shock protein 90 beta family member 2, pseudogene [Source:HGNC Symbol;Acc:HGNC:12099]	processed pseudogene	1.03	5.15	13.22	0.00	0.03
ENSG00000137804	NUSAP1	nucleolar and spindle associated protein 1 [Source:HGNC Symbol;Acc:HGNC:18538]	protein coding	1.03	4.54	13.84	0.00	0.03
ENSG00000156970	BUB1B	BUB1 mitotic checkpoint serine/threonine kinase B [Source:HGNC Symbol;Acc:HGNC:1149]	protein coding	1.01	3.03	12.02	0.00	0.05
ENSG00000211946	IGHV3-20	immunoglobulin heavy variable 3-20 [Source:HGNC Symbol;Acc:HGNC:5585]	IG V gene	1.00	4.47	18.95	0.00	0.01
ENSG00000280411	IGHV1-69-2	immunoglobulin heavy variable 1-69-2 [Source:HGNC Symbol;Acc:HGNC:5562]	IG V gene	0.98	5.43	14.90	0.00	0.02
ENSG00000123080	CDKN2C	cyclin dependent kinase inhibitor 2C [Source:HGNC Symbol;Acc:HGNC:1789]	protein coding	0.97	2.98	12.68	0.00	0.04
ENSG00000211653	IGLV1-40	immunoglobulin lambda variable 1-40 [Source:HGNC Symbol;Acc:HGNC:5877]	IG V gene	0.96	7.46	19.11	0.00	0.01
ENSG00000080986	NDC80	NDC80, kinetochore complex component [Source:HGNC Symbol;Acc:HGNC:16909]	protein coding	0.96	3.30	11.88	0.00	0.05
ENSG00000068489	PRR11	proline rich 11 [Source:HGNC Symbol;Acc:HGNC:25619]	protein coding	0.94	3.84	14.17	0.00	0.02
ENSG00000242076	IGKV1-33	immunoglobulin kappa variable 1-33 [Source:HGNC Symbol;Acc:HGNC:5737]	IG V gene	0.94	4.78	12.05	0.00	0.05
ENSG00000074416	MGLL	monoglyceride lipase [Source:HGNC Symbol;Acc:HGNC:17038]	protein coding	0.94	3.89	14.26	0.00	0.02
ENSG00000160712	IL6R	interleukin 6 receptor [Source:HGNC Symbol;Acc:HGNC:6019]	protein coding	0.93	6.64	13.83	0.00	0.03
ENSG00000070214	SLC44A1	solute carrier family 44 member 1 [Source:HGNC Symbol;Acc:HGNC:18798]	protein coding	0.92	7.25	11.96	0.00	0.05
ENSG00000211669	IGLV3-10	immunoglobulin lambda variable 3-10 [Source:HGNC Symbol;Acc:HGNC:5897]	IG V gene	0.91	5.96	17.76	0.00	0.01
ENSG00000076003	MCM6	minichromosome maintenance complex component 6 [Source:HGNC Symbol;Acc:HGNC:6949]	protein coding	0.90	5.36	12.50	0.00	0.04
ENSG00000132432	SEC61G	Sec61 translocon gamma subunit [Source:HGNC Symbol;Acc:HGNC:18277]	protein coding	0.90	5.39	18.04	0.00	0.01
ENSG00000182481	KPNA2	karyopherin subunit alpha 2 [Source:HGNC Symbol;Acc:HGNC:6395]	protein coding	0.89	5.21	14.30	0.00	0.02

Ensembl Gene ID	Ensembl Gene Name	Ensembl Gene Description	Gene Type	Log_2 Fold Change (Day 5 vs. pre-treatment)	Average Log_2 CPM	Likelihood Ratio Test Statistic	P-Value	FDR Adjusted P-Value
ENSG00000244575	IGKV1-27	immunoglobulin kappa variable 1-27 [Source:HGNC Symbol;Acc:HGNC:5735]	IG V gene	0.88	5.55	33.21	0.00	0.00
ENSG00000196189	SEMA4A	semaphorin 4A [Source:HGNC Symbol;Acc:HGNC:10729]	protein coding	0.87	6.85	12.34	0.00	0.04
ENSG00000007968	E2F2	E2F transcription factor 2 [Source:HGNC Symbol;Acc:HGNC:3114]	protein coding	0.86	5.11	13.12	0.00	0.03
ENSG00000140525	FANCI	Fanconi anemia complementation group I [Source:HGNC Symbol;Acc:HGNC:25568]	protein coding	0.86	4.70	13.21	0.00	0.03
ENSG00000211892	IGHG4	immunoglobulin heavy constant gamma 4 (G4m marker) [Source:HGNC Symbol;Acc:HGNC:5528]	IG C gene	0.85	7.17	12.26	0.00	0.04
ENSG00000211662	IGLV3-21	immunoglobulin lambda variable 3-21 [Source:HGNC Symbol;Acc:HGNC:5905]	IG V gene	0.84	7.93	19.33	0.00	0.01
ENSG00000108106	UBE2S	ubiquitin conjugating enzyme E2 S [Source:HGNC Symbol;Acc:HGNC:17895]	protein coding	0.83	3.49	11.99	0.00	0.05
ENSG00000173540	GMPPB	GDP-mannose pyrophosphorylase B [Source:HGNC Symbol;Acc:HGNC:22932]	protein coding	0.83	5.54	12.22	0.00	0.04
ENSG00000113368	LMNB1	lamin B1 [Source:HGNC Symbol;Acc:HGNC:6637]	protein coding	0.82	5.73	18.91	0.00	0.01
ENSG00000188486	H2AFX	H2A histone family member X [Source:HGNC Symbol;Acc:HGNC:4739]	protein coding	0.81	4.39	13.64	0.00	0.03
ENSG00000114850	SSR3	signal sequence receptor subunit 3 [Source:HGNC Symbol;Acc:HGNC:11325]	protein coding	0.79	7.82	12.12	0.00	0.05
ENSG00000168209	DDIT4	DNA damage inducible transcript 4 [Source:HGNC Symbol;Acc:HGNC:24944]	protein coding	-0.79	4.61	27.04	0.00	0.00
ENSG00000167553	TUBA1C	tubulin alpha 1c [Source:HGNC Symbol;Acc:HGNC:20768]	protein coding	0.75	3.80	14.39	0.00	0.02
ENSG00000146918	NCAPG2	non-SMC condensin II complex subunit G2 [Source:HGNC Symbol;Acc:HGNC:21904]	protein coding	0.71	4.52	12.98	0.00	0.03
ENSG00000132646	PCNA	proliferating cell nuclear antigen [Source:HGNC Symbol;Acc:HGNC:8729]	protein coding	0.70	5.78	13.89	0.00	0.03
ENSG00000117632	STMN1	stathmin 1 [Source:HGNC Symbol;Acc:HGNC:6510]	protein coding	0.69	5.85	14.63	0.00	0.02
ENSG00000134291	TMEM106C	transmembrane protein 106C [Source:HGNC Symbol;Acc:HGNC:28775]	protein coding	0.68	4.94	15.26	0.00	0.02
ENSG00000251546	IGKV1D-39	immunoglobulin kappa variable 1D-39 [Source:HGNC Symbol;Acc:HGNC:5756]	IG V gene	0.68	4.71	12.99	0.00	0.03
ENSG00000163507	KIAA1524	KIAA1524 [Source:HGNC Symbol;Acc:HGNC:29302]	protein coding	0.67	3.76	14.69	0.00	0.02
ENSG00000271533			sense intronic	-0.67	3.61	13.80	0.00	0.03
ENSG00000123416	TUBA1B	tubulin alpha 1b [Source:HGNC Symbol;Acc:HGNC:18809]	protein coding	0.65	5.35	12.99	0.00	0.03
ENSG00000164032	H2AFZ	H2A histone family member Z [Source:HGNC Symbol;Acc:HGNC:4741]	protein coding	0.63	6.58	13.02	0.00	0.03
ENSG00000104889	RNASEH2A	ribonuclease H2 subunit A [Source:HGNC Symbol;Acc:HGNC:18518]	protein coding	0.62	3.74	12.55	0.00	0.04

Ensembl Gene ID	Ensembl Gene Name	Ensembl Gene Description	Gene Type	Log_2 Fold Change (Day 5 vs. pre-treatment)	Average Log_2 CPM	Likelihood Ratio Test Statistic	P-Value	FDR Adjusted P-Value
ENSG00000224373	IGHV4-59	immunoglobulin heavy variable 4-59 [Source:HGNC Symbol;Acc:HGNC:5654]	IG V gene	0.62	6.20	14.02	0.00	0.03
ENSG00000244116	IGKV2-28	immunoglobulin kappa variable 2-28 [Source:HGNC Symbol;Acc:HGNC:5783]	IG V gene	0.61	3.88	14.63	0.00	0.02
ENSG00000164104	HMGB2	high mobility group box 2 [Source:HGNC Symbol;Acc:HGNC:5000]	protein coding	0.61	7.10	13.54	0.00	0.03
ENSG00000080546	SESN1	sestrin 1 [Source:HGNC Symbol;Acc:HGNC:21595]	protein coding	-0.59	6.66	28.01	0.00	0.00
ENSG00000211625	IGKV3D-20	immunoglobulin kappa variable 3D-20 [Source:HGNC Symbol;Acc:HGNC:5825]	IG V gene	0.59	5.42	13.31	0.00	0.03
ENSG00000198900	TOP1	topoisomerase (DNA) I [Source:HGNC Symbol;Acc:HGNC:11986]	protein coding	0.59	6.91	12.13	0.00	0.05

Table 11: Genes differentially expressed at Day 5 compared to pre-treatment (B Cells, Trivalent Influenza Vaccine). Sorted by descending absolute log_2 fold change (Day 5 vs. pre-treatment). Gene model summaries and annotations are based on Ensembl Version 87.

Ensembl Gene ID	Ensembl Gene Name	Ensembl Gene Description	Gene Type	Log_2 Fold Change (Day 6 vs. pre-treatment)	Average Log_2 CPM	Likelihood Ratio Test Statistic	P-Value	FDR Adjusted P-Value
ENSG00000259997	IGHV1OR16-4	immunoglobulin heavy variable 1/OR16-4 (pseudogene) [Source:HGNC Symbol;Acc:HGNC:5573]	IG V pseudogene	2.86	-0.04	11.82	0.00	0.01
ENSG00000148468	FAM171A1	family with sequence similarity 171 member A1 [Source:HGNC Symbol;Acc:HGNC:23522]	protein coding	2.55	0.79	22.53	0.00	0.00
ENSG00000211938	IGHV3-7	immunoglobulin heavy variable 3-7 [Source:HGNC Symbol;Acc:HGNC:5620]	IG V gene	2.29	9.88	19.87	0.00	0.00
ENSG00000153093	ACOXL	acyl-CoA oxidase-like [Source:HGNC Symbol;Acc:HGNC:25621]	protein coding	2.29	1.12	15.48	0.00	0.00
ENSG00000111186	WNT5B	Wnt family member 5B [Source:HGNC Symbol;Acc:HGNC:16265]	protein coding	2.23	1.16	19.80	0.00	0.00
ENSG00000253131	IGHV7-56	immunoglobulin heavy variable 7-56 (pseudogene) [Source:HGNC Symbol;Acc:HGNC:5667]	IG V pseudogene	2.17	0.94	13.07	0.00	0.01
ENSG00000211896	IGHG1	immunoglobulin heavy constant gamma 1 (G1m marker) [Source:HGNC Symbol;Acc:HGNC:5525]	IG C gene	2.14	12.71	37.50	0.00	0.00
ENSG00000113140	SPARC	secreted protein acidic and cysteine rich [Source:HGNC Symbol;Acc:HGNC:11219]	protein coding	2.06	1.61	11.51	0.00	0.01
ENSG00000105974	CAV1	caveolin 1 [Source:HGNC Symbol;Acc:HGNC:1527]	protein coding	1.96	4.28	43.38	0.00	0.00
ENSG00000168078	PBK	PDZ binding kinase [Source:HGNC Symbol;Acc:HGNC:18282]	protein coding	1.94	1.37	17.30	0.00	0.00
ENSG00000254174	IGHV1-12	immunoglobulin heavy variable 1-12 (pseudogene) [Source:HGNC Symbol;Acc:HGNC:5546]	IG V pseudogene	1.92	0.50	10.47	0.00	0.02

Ensembl Gene ID	Ensembl Gene Name	Ensembl Gene Description	Gene Type	Log_2 Fold Change (Day 6 vs. pre-treatment)	Average Log_2 CPM	Likelihood Ratio Test Statistic	P-Value	FDR Adjusted P-Value
ENSG00000088340	FER1L4	fer-1 like family member 4, pseudogene [Source:HGNC Symbol;Acc:HGNC:15801]	transcribed unitary pseudogene	1.91	2.51	16.37	0.00	0.00
ENSG00000154277	UCHL1	ubiquitin C-terminal hydrolase L1 [Source:HGNC Symbol;Acc:HGNC:12513]	protein coding	1.91	1.44	22.93	0.00	0.00
ENSG00000253755	IGHGP	immunoglobulin heavy constant gamma P (non-functional) [Source:HGNC Symbol;Acc:HGNC:5529]	IG C pseudogene	1.85	7.63	35.00	0.00	0.00
ENSG00000115163	CENPA	centromere protein A [Source:HGNC Symbol;Acc:HGNC:1851]	protein coding	1.81	1.36	14.06	0.00	0.00
ENSG00000163053	SLC16A14	solute carrier family 16 member 14 [Source:HGNC Symbol;Acc:HGNC:26417]	protein coding	1.76	2.53	22.76	0.00	0.00
ENSG00000129173	E2F8	E2F transcription factor 8 [Source:HGNC Symbol;Acc:HGNC:24727]	protein coding	1.75	1.65	14.21	0.00	0.00
ENSG00000024526	DEPDC1	DEP domain containing 1 [Source:HGNC Symbol;Acc:HGNC:22949]	protein coding	1.71	1.58	13.15	0.00	0.01
ENSG00000211653	IGLV1-40	immunoglobulin lambda variable 1-40 [Source:HGNC Symbol;Acc:HGNC:5877]	IG V gene	1.70	8.37	22.32	0.00	0.00
ENSG00000115884	SDC1	syndecan 1 [Source:HGNC Symbol;Acc:HGNC:10658]	protein coding	1.69	4.16	18.89	0.00	0.00
ENSG00000248571			antisense	1.69	2.23	19.44	0.00	0.00
ENSG00000171848	RRM2	ribonucleotide reductase regulatory subunit M2 [Source:HGNC Symbol;Acc:HGNC:10452]	protein coding	1.68	5.72	35.36	0.00	0.00
ENSG00000211973	IGHV1-69	immunoglobulin heavy variable 1-69 [Source:HGNC Symbol;Acc:HGNC:5558]	IG V gene	1.66	4.85	13.96	0.00	0.00
ENSG00000138166	DUSP5	dual specificity phosphatase 5 [Source:HGNC Symbol;Acc:HGNC:3071]	protein coding	1.65	5.16	30.19	0.00	0.00
ENSG00000211651	IGLV1-44	immunoglobulin lambda variable 1-44 [Source:HGNC Symbol;Acc:HGNC:5879]	IG V gene	1.64	8.25	8.31	0.00	0.04
ENSG00000164045	CDC25A	cell division cycle 25A [Source:HGNC Symbol;Acc:HGNC:1725]	protein coding	1.64	2.14	14.62	0.00	0.00
ENSG00000211662	IGLV3-21	immunoglobulin lambda variable 3-21 [Source:HGNC Symbol;Acc:HGNC:5905]	IG V gene	1.62	8.45	46.36	0.00	0.00
ENSG00000121807	CCR2	C-C motif chemokine receptor 2 [Source:HGNC Symbol;Acc:HGNC:1603]	protein coding	1.62	6.01	42.67	0.00	0.00
ENSG00000184661	CDCA2	cell division cycle associated 2 [Source:HGNC Symbol;Acc:HGNC:14623]	protein coding	1.61	2.11	17.75	0.00	0.00
ENSG00000093009	CDC45	cell division cycle 45 [Source:HGNC Symbol;Acc:HGNC:1739]	protein coding	1.60	2.12	17.37	0.00	0.00
ENSG00000239975	IGKV1D-33	immunoglobulin kappa variable 1D-33 [Source:HGNC Symbol;Acc:HGNC:5753]	IG V gene	1.60	4.35	20.76	0.00	0.00
ENSG00000136010	ALDH1L2	aldehyde dehydrogenase 1 family member L2 [Source:HGNC Symbol;Acc:HGNC:26777]	protein coding	1.59	3.85	26.41	0.00	0.00
ENSG00000169679	BUB1	BUB1 mitotic checkpoint serine/threonine kinase [Source:HGNC Symbol;Acc:HGNC:1148]	protein coding	1.59	4.86	39.96	0.00	0.00

Ensembl Gene ID	Ensembl Gene Name	Ensembl Gene Description	Gene Type	Log_2 Fold Change (Day 6 vs. pre-treatment)	Average Log_2 CPM	Likelihood Ratio Test Statistic	P-Value	FDR Adjusted P-Value
ENSG00000197476			processed pseudo-gene	1.58	3.78	10.93	0.00	0.01
ENSG00000162676	GFI1	growth factor independent 1 transcriptional repressor [Source:HGNC Symbol;Acc:HGNC:4237]	protein coding	1.56	2.58	22.37	0.00	0.00
ENSG00000090104	RGS1	regulator of G-protein signaling 1 [Source:HGNC Symbol;Acc:HGNC:9991]	protein coding	1.56	3.15	24.02	0.00	0.00
ENSG00000139734	DIAPH3	diaphanous related formin 3 [Source:HGNC Symbol;Acc:HGNC:15480]	protein coding	1.56	1.46	11.41	0.00	0.01
ENSG00000137563	GGH	gamma-glutamyl hydrolase [Source:HGNC Symbol;Acc:HGNC:4248]	protein coding	1.55	4.01	37.07	0.00	0.00
ENSG00000075420	FNDC3B	fibronectin type III domain containing 3B [Source:HGNC Symbol;Acc:HGNC:24670]	protein coding	1.55	5.71	28.60	0.00	0.00
ENSG00000122952	ZWINT	ZW10 interacting kinetochore protein [Source:HGNC Symbol;Acc:HGNC:13195]	protein coding	1.55	3.58	25.71	0.00	0.00
ENSG00000270472	IGHV3OR16-9	immunoglobulin heavy variable 3/OR16-9 (non-functional) [Source:HGNC Symbol;Acc:HGNC:5644]	IG V gene	1.54	4.26	14.51	0.00	0.00
ENSG00000165409	TSHR	thyroid stimulating hormone receptor [Source:HGNC Symbol;Acc:HGNC:12373]	protein coding	1.54	2.79	16.37	0.00	0.00
ENSG00000171241	SHCBP1	SHC binding and spindle associated 1 [Source:HGNC Symbol;Acc:HGNC:29547]	protein coding	1.53	4.70	30.84	0.00	0.00
ENSG00000109805	NCAPG	non-SMC condensin I complex subunit G [Source:HGNC Symbol;Acc:HGNC:24304]	protein coding	1.53	3.36	26.95	0.00	0.00
ENSG00000216775			transcribed unprocessed pseudo-gene	1.53	1.83	12.21	0.00	0.01
ENSG00000211645	IGLV1-50	immunoglobulin lambda variable 1-50 (non-functional) [Source:HGNC Symbol;Acc:HGNC:5881]	IG V gene	1.53	1.30	9.18	0.00	0.03
ENSG00000123485	HJURP	Holliday junction recognition protein [Source:HGNC Symbol;Acc:HGNC:25444]	protein coding	1.52	2.56	16.25	0.00	0.00
ENSG00000165304	MELK	maternal embryonic leucine zipper kinase [Source:HGNC Symbol;Acc:HGNC:16870]	protein coding	1.52	1.83	14.57	0.00	0.00
ENSG00000280411	IGHV1-69-2	immunoglobulin heavy variable 1-69-2 [Source:HGNC Symbol;Acc:HGNC:5562]	IG V gene	1.52	5.87	21.35	0.00	0.00
ENSG00000126787	DLGAP5	DLG associated protein 5 [Source:HGNC Symbol;Acc:HGNC:16864]	protein coding	1.51	3.25	25.83	0.00	0.00
ENSG00000100219	XBP1	X-box binding protein 1 [Source:HGNC Symbol;Acc:HGNC:12801]	protein coding	1.50	8.71	41.49	0.00	0.00
ENSG00000203914	HSP90B3P	heat shock protein 90 beta family member 3, pseudogene [Source:HGNC Symbol;Acc:HGNC:12100]	processed pseudo-gene	1.49	2.99	28.60	0.00	0.00
ENSG00000259706	HSP90B2P	heat shock protein 90 beta family member 2, pseudogene [Source:HGNC Symbol;Acc:HGNC:12099]	processed pseudo-gene	1.49	5.26	64.73	0.00	0.00

Ensembl Gene ID	Ensembl Gene Name	Ensembl Gene Description	Gene Type	Log_2 Fold Change (Day 6 vs. pre-treatment)	Average Log_2 CPM	Likelihood Ratio Test Statistic	P-Value	FDR Adjusted P-Value
ENSG00000094804	CDC6	cell division cycle 6 [Source:HGNC Symbol;Acc:HGNC:1744]	protein coding	1.47	3.51	29.32	0.00	0.00
ENSG00000143476	DTL	denticleless E3 ubiquitin protein ligase homolog [Source:HGNC Symbol;Acc:HGNC:30288]	protein coding	1.47	3.16	21.56	0.00	0.00
ENSG00000166598	HSP90B1	heat shock protein 90 beta family member 1 [Source:HGNC Symbol;Acc:HGNC:12028]	protein coding	1.47	10.91	44.94	0.00	0.00
ENSG00000253691	IGKV2OR22-4	immunoglobulin kappa variable 2/OR22-4 (pseudogene) [Source:HGNC Symbol;Acc:HGNC:5813]	IG V pseudogene	1.47	3.86	18.92	0.00	0.00
ENSG00000178445	GLDC	glycine decarboxylase [Source:HGNC Symbol;Acc:HGNC:4313]	protein coding	1.47	5.19	22.25	0.00	0.00
ENSG00000089685	BIRC5	baculoviral IAP repeat containing 5 [Source:HGNC Symbol;Acc:HGNC:593]	protein coding	1.46	3.23	19.89	0.00	0.00
ENSG00000123989	CHPF	chondroitin polymerizing factor [Source:HGNC Symbol;Acc:HGNC:24291]	protein coding	1.45	5.08	29.66	0.00	0.00
ENSG00000112984	KIF20A	kinesin family member 20A [Source:HGNC Symbol;Acc:HGNC:9787]	protein coding	1.44	1.84	13.77	0.00	0.00
ENSG00000170312	CDK1	cyclin dependent kinase 1 [Source:HGNC Symbol;Acc:HGNC:1722]	protein coding	1.43	3.09	18.89	0.00	0.00
ENSG00000074842	MYDGF	myeloid derived growth factor [Source:HGNC Symbol;Acc:HGNC:16948]	protein coding	1.43	6.74	36.94	0.00	0.00
ENSG00000065485	PDIA5	protein disulfide isomerase family A member 5 [Source:HGNC Symbol;Acc:HGNC:24811]	protein coding	1.43	4.52	29.72	0.00	0.00
ENSG00000243238	IGKV2-30	immunoglobulin kappa variable 2-30 [Source:HGNC Symbol;Acc:HGNC:5785]	IG V gene	1.42	6.08	10.14	0.00	0.02
ENSG00000176890	TYMS	thymidylate synthetase [Source:HGNC Symbol;Acc:HGNC:12441]	protein coding	1.41	3.24	23.27	0.00	0.00
ENSG00000239571	IGKV2D-30	immunoglobulin kappa variable 2D-30 [Source:HGNC Symbol;Acc:HGNC:5801]	IG V gene	1.40	4.06	8.23	0.00	0.04
ENSG00000088325	TPX2	TPX2, microtubule nucleation factor [Source:HGNC Symbol;Acc:HGNC:1249]	protein coding	1.40	4.03	32.04	0.00	0.00
ENSG00000253818	IGLV1-41	immunoglobulin lambda variable 1-41 (pseudogene) [Source:HGNC Symbol;Acc:HGNC:5878]	IG V pseudogene	1.39	5.33	9.81	0.00	0.02
ENSG00000137807	KIF23	kinesin family member 23 [Source:HGNC Symbol;Acc:HGNC:6392]	protein coding	1.39	2.48	19.19	0.00	0.00
ENSG00000065328	MCM10	minichromosome maintenance 10 replication initiation factor [Source:HGNC Symbol;Acc:HGNC:18043]	protein coding	1.39	2.26	14.10	0.00	0.00
ENSG00000025039	RRAGD	Ras related GTP binding D [Source:HGNC Symbol;Acc:HGNC:19903]	protein coding	1.38	3.01	37.33	0.00	0.00
ENSG00000155660	PDIA4	protein disulfide isomerase family A member 4 [Source:HGNC Symbol;Acc:HGNC:30167]	protein coding	1.37	8.84	35.12	0.00	0.00
ENSG00000118985	ELL2	elongation factor for RNA polymerase II 2 [Source:HGNC Symbol;Acc:HGNC:17064]	protein coding	1.37	7.37	35.51	0.00	0.00
ENSG00000100629	CEP128	centrosomal protein 128 [Source:HGNC Symbol;Acc:HGNC:20359]	protein coding	1.36	4.66	30.82	0.00	0.00
ENSG00000029993	HMGB3	high mobility group box 3 [Source:HGNC Symbol;Acc:HGNC:5004]	protein coding	1.36	2.90	20.55	0.00	0.00

Ensembl Gene ID	Ensembl Gene Name	Ensembl Gene Description	Gene Type	Log_2 Fold Change (Day 6 vs. pre-treatment)	Average Log_2 CPM	Likelihood Ratio Test Statistic	P-Value	FDR Adjusted P-Value
ENSG00000138778	CENPE	centromere protein E [Source:HGNC Symbol;Acc:HGNC:1856]	protein coding	1.36	3.78	25.82	0.00	0.00
ENSG00000198722	UNC13B	unc-13 homolog B [Source:HGNC Symbol;Acc:HGNC:12566]	protein coding	1.35	2.18	14.91	0.00	0.00
ENSG00000108641	B9D1	B9 domain containing 1 [Source:HGNC Symbol;Acc:HGNC:24123]	protein coding	1.34	2.17	18.47	0.00	0.00
ENSG00000148773	MKI67	marker of proliferation Ki-67 [Source:HGNC Symbol;Acc:HGNC:7107]	protein coding	1.34	6.50	30.16	0.00	0.00
ENSG00000072571	HMMR	hyaluronan mediated motility receptor [Source:HGNC Symbol;Acc:HGNC:5012]	protein coding	1.33	3.04	19.69	0.00	0.00
ENSG00000119326	CTNNAL1	catenin alpha like 1 [Source:HGNC Symbol;Acc:HGNC:2512]	protein coding	1.33	2.51	21.66	0.00	0.00
ENSG00000026751	SLAMF7	SLAM family member 7 [Source:HGNC Symbol;Acc:HGNC:21394]	protein coding	1.33	7.71	28.70	0.00	0.00
ENSG00000271178	IGHV3OR16-13	immunoglobulin heavy variable 3/OR16-13 (non-functional) [Source:HGNC Symbol;Acc:HGNC:5637]	IG V gene	1.32	4.14	15.33	0.00	0.00
ENSG00000102471	NDFIP2	Nedd4 family interacting protein 2 [Source:HGNC Symbol;Acc:HGNC:18537]	protein coding	1.29	2.17	13.62	0.00	0.00
ENSG00000242076	IGKV1-33	immunoglobulin kappa variable 1-33 [Source:HGNC Symbol;Acc:HGNC:5737]	IG V gene	1.29	5.08	13.71	0.00	0.00
ENSG00000107104	KANK1	KN motif and ankyrin repeat domains 1 [Source:HGNC Symbol;Acc:HGNC:19309]	protein coding	1.29	3.14	14.41	0.00	0.00
ENSG00000186185	KIF18B	kinesin family member 18B [Source:HGNC Symbol;Acc:HGNC:27102]	protein coding	1.27	1.85	7.99	0.00	0.05
ENSG00000186810	CXCR3	C-X-C motif chemokine receptor 3 [Source:HGNC Symbol;Acc:HGNC:4540]	protein coding	1.27	4.16	23.33	0.00	0.00
ENSG00000173578	XCR1	X-C motif chemokine receptor 1 [Source:HGNC Symbol;Acc:HGNC:1625]	protein coding	1.27	2.64	9.81	0.00	0.02
ENSG00000111885	MAN1A1	mannosidase alpha class 1A member 1 [Source:HGNC Symbol;Acc:HGNC:6821]	protein coding	1.26	8.25	35.53	0.00	0.00
ENSG00000004468	CD38	CD38 molecule [Source:HGNC Symbol;Acc:HGNC:1667]	protein coding	1.26	7.89	37.15	0.00	0.00
ENSG00000048462	TNFRSF17	TNF receptor superfamily member 17 [Source:HGNC Symbol;Acc:HGNC:11913]	protein coding	1.26	6.60	39.89	0.00	0.00
ENSG00000145050	MANF	mesencephalic astrocyte derived neurotrophic factor [Source:HGNC Symbol;Acc:HGNC:15461]	protein coding	1.25	6.51	44.05	0.00	0.00
ENSG00000100526	CDKN3	cyclin dependent kinase inhibitor 3 [Source:HGNC Symbol;Acc:HGNC:1791]	protein coding	1.25	1.71	8.96	0.00	0.03
ENSG00000123131	PRDX4	peroxiredoxin 4 [Source:HGNC Symbol;Acc:HGNC:17169]	protein coding	1.25	5.39	26.27	0.00	0.00
ENSG00000172965	MIR4435-2HG	MIR4435-2 host gene [Source:HGNC Symbol;Acc:HGNC:35163]	lincRNA	1.25	2.70	19.74	0.00	0.00
ENSG00000044574	HSPA5	heat shock protein family A (Hsp70) member 5 [Source:HGNC Symbol;Acc:HGNC:5238]	protein coding	1.25	9.35	41.58	0.00	0.00
ENSG00000101057	MYBL2	MYB proto-oncogene like 2 [Source:HGNC Symbol;Acc:HGNC:7548]	protein coding	1.24	6.14	27.12	0.00	0.00

Ensembl Gene ID	Ensembl Gene Name	Ensembl Gene Description	Gene Type	Log_2 Fold Change (Day 6 vs. pre-treatment)	Average Log_2 CPM	Likelihood Ratio Test Statistic	P-Value	FDR Adjusted P-Value
ENSG00000128228	SDF2L1	stromal cell derived factor 2 like 1 [Source:HGNC Symbol;Acc:HGNC:10676]	protein coding	1.24	5.17	31.30	0.00	0.00
ENSG00000011426	ANLN	anillin actin binding protein [Source:HGNC Symbol;Acc:HGNC:14082]	protein coding	1.23	1.83	9.35	0.00	0.03
ENSG00000242766	IGKV1D-17	immunoglobulin kappa variable 1D-17 [Source:HGNC Symbol;Acc:HGNC:5749]	IG V gene	1.23	3.41	16.00	0.00	0.00
ENSG00000173334	TRIB1	tribbles pseudokinase 1 [Source:HGNC Symbol;Acc:HGNC:16891]	protein coding	1.23	6.09	25.98	0.00	0.00
ENSG00000085840	ORC1	origin recognition complex subunit 1 [Source:HGNC Symbol;Acc:HGNC:8487]	protein coding	1.22	2.56	17.00	0.00	0.00
ENSG00000111424	VDR	vitamin D (1,25- dihydroxyvitamin D3) receptor [Source:HGNC Symbol;Acc:HGNC:12679]	protein coding	1.22	3.71	23.89	0.00	0.00
ENSG00000211648	IGLV1-47	immunoglobulin lambda variable 1-47 [Source:HGNC Symbol;Acc:HGNC:5880]	IG V gene	1.21	7.27	8.51	0.00	0.04
ENSG00000227295	ELL2P1	elongation factor for RNA polymerase II 2 pseudogene 1 [Source:HGNC Symbol;Acc:HGNC:39343]	processed pseudogene	1.21	2.21	17.10	0.00	0.00
ENSG00000171444	MCC	mutated in colorectal cancers [Source:HGNC Symbol;Acc:HGNC:6935]	protein coding	1.21	2.19	16.52	0.00	0.00
ENSG00000169607	CKAP2L	cytoskeleton associated protein 2 like [Source:HGNC Symbol;Acc:HGNC:26877]	protein coding	1.21	1.67	9.91	0.00	0.02
ENSG00000211677	IGLC2	immunoglobulin lambda constant 2 [Source:HGNC Symbol;Acc:HGNC:5856]	IG C gene	1.21	10.20	29.40	0.00	0.00
ENSG00000185164	NOMO2	NODAL modulator 2 [Source:HGNC Symbol;Acc:HGNC:22652]	protein coding	1.21	4.42	21.84	0.00	0.00
ENSG00000105976	MET	MET proto-oncogene, receptor tyrosine kinase [Source:HGNC Symbol;Acc:HGNC:7029]	protein coding	1.20	1.94	11.08	0.00	0.01
ENSG00000132465	JCHAIN	joining chain of multimeric IgA and IgM [Source:HGNC Symbol;Acc:HGNC:5713]	protein coding	1.20	11.84	20.14	0.00	0.00
ENSG00000079931	MOXD1	monooxygenase DBH like 1 [Source:HGNC Symbol;Acc:HGNC:21063]	protein coding	1.20	4.02	26.77	0.00	0.00
ENSG00000186594	MIR22HG	MIR22 host gene [Source:HGNC Symbol;Acc:HGNC:28219]	lincRNA	1.20	3.21	26.00	0.00	0.00
ENSG00000173848	NET1	neuroepithelial cell transforming 1 [Source:HGNC Symbol;Acc:HGNC:14592]	protein coding	1.20	4.44	40.79	0.00	0.00
ENSG00000146670	CDCA5	cell division cycle associated 5 [Source:HGNC Symbol;Acc:HGNC:14626]	protein coding	1.20	2.83	11.28	0.00	0.01
ENSG00000138180	CEP55	centrosomal protein 55 [Source:HGNC Symbol;Acc:HGNC:1161]	protein coding	1.20	2.69	18.27	0.00	0.00
ENSG00000075218	GTSE1	G2 and S-phase expressed 1 [Source:HGNC Symbol;Acc:HGNC:13698]	protein coding	1.19	2.53	13.34	0.00	0.01
ENSG00000178999	AURKB	aurora kinase B [Source:HGNC Symbol;Acc:HGNC:11390]	protein coding	1.19	2.51	16.89	0.00	0.00
ENSG00000070081	NUCB2	nucleobindin 2 [Source:HGNC Symbol;Acc:HGNC:8044]	protein coding	1.19	5.73	29.98	0.00	0.00

Ensembl Gene ID	Ensembl Gene Name	Ensembl Gene Description	Gene Type	Log_2 Fold Change (Day 6 vs. pre-treatment)	Average Log_2 CPM	Likelihood Ratio Test Statistic	P-Value	FDR Adjusted P-Value
ENSG00000143333	RGS16	regulator of G-protein signaling 16 [Source:HGNC Symbol;Acc:HGNC:9997]	protein coding	1.18	2.26	9.59	0.00	0.02
ENSG00000071539	TRIP13	thyroid hormone receptor interactor 13 [Source:HGNC Symbol;Acc:HGNC:12307]	protein coding	1.18	2.68	17.04	0.00	0.00
ENSG00000164611	PTTG1	pituitary tumor-transforming 1 [Source:HGNC Symbol;Acc:HGNC:9690]	protein coding	1.18	3.58	28.87	0.00	0.00
ENSG00000166794	PPIB	peptidylprolyl isomerase B [Source:HGNC Symbol;Acc:HGNC:9255]	protein coding	1.17	7.76	35.31	0.00	0.00
ENSG00000070214	SLC44A1	solute carrier family 44 member 1 [Source:HGNC Symbol;Acc:HGNC:18798]	protein coding	1.17	7.27	37.23	0.00	0.00
ENSG00000131747	TOP2A	topoisomerase (DNA) II alpha [Source:HGNC Symbol;Acc:HGNC:11989]	protein coding	1.16	5.16	26.25	0.00	0.00
ENSG00000198794	SCAMP5	secretory carrier membrane protein 5 [Source:HGNC Symbol;Acc:HGNC:30386]	protein coding	1.16	3.61	16.23	0.00	0.00
ENSG00000224607	IGKV1D-27	immunoglobulin kappa variable 1D-27 (pseudogene) [Source:HGNC Symbol;Acc:HGNC:5751]	IG V pseudogene	1.16	2.24	16.38	0.00	0.00
ENSG00000175063	UBE2C	ubiquitin conjugating enzyme E2 C [Source:HGNC Symbol;Acc:HGNC:15937]	protein coding	1.16	2.29	9.99	0.00	0.02
ENSG00000117650	NEK2	NIMA related kinase 2 [Source:HGNC Symbol;Acc:HGNC:7745]	protein coding	1.16	1.86	8.55	0.00	0.04
ENSG00000135916	ITM2C	integral membrane protein 2C [Source:HGNC Symbol;Acc:HGNC:6175]	protein coding	1.16	8.86	21.17	0.00	0.00
ENSG00000166562	SEC11C	SEC11 homolog C, signal peptidase complex subunit [Source:HGNC Symbol;Acc:HGNC:23400]	protein coding	1.15	7.45	31.65	0.00	0.00
ENSG00000113615	SEC24A	SEC24 homolog A, COPII coat complex component [Source:HGNC Symbol;Acc:HGNC:10703]	protein coding	1.15	6.74	32.35	0.00	0.00
ENSG00000121152	NCAPH	non-SMC condensin I complex subunit H [Source:HGNC Symbol;Acc:HGNC:1112]	protein coding	1.15	2.85	14.35	0.00	0.00
ENSG00000167861	HID1	HID1 domain containing [Source:HGNC Symbol;Acc:HGNC:15736]	protein coding	1.13	4.25	16.86	0.00	0.00
ENSG00000168268	NT5DC2	5'-nucleotidase domain containing 2 [Source:HGNC Symbol;Acc:HGNC:25717]	protein coding	1.13	5.59	26.75	0.00	0.00
ENSG00000118515	SGK1	serum/glucocorticoid regulated kinase 1 [Source:HGNC Symbol;Acc:HGNC:10810]	protein coding	1.13	4.47	32.63	0.00	0.00
ENSG00000066279	ASPM	abnormal spindle microtubule assembly [Source:HGNC Symbol;Acc:HGNC:19048]	protein coding	1.13	4.14	18.24	0.00	0.00
ENSG00000183010	PYCR1	pyrroline-5-carboxylate reductase 1 [Source:HGNC Symbol;Acc:HGNC:9721]	protein coding	1.13	2.67	18.09	0.00	0.00
ENSG00000211946	IGHV3-20	immunoglobulin heavy variable 3-20 [Source:HGNC Symbol;Acc:HGNC:5585]	IG V gene	1.12	4.62	22.88	0.00	0.00
ENSG00000174132	FAM174A	family with sequence similarity 174 member A [Source:HGNC Symbol;Acc:HGNC:24943]	protein coding	1.12	1.98	7.85	0.01	0.05
ENSG00000112378	PERP	PERP, TP53 apoptosis effector [Source:HGNC Symbol;Acc:HGNC:17637]	protein coding	1.12	3.95	19.10	0.00	0.00
ENSG00000167634	NLRP7	NLR family pyrin domain containing 7 [Source:HGNC Symbol;Acc:HGNC:22947]	protein coding	1.12	1.97	7.72	0.01	0.05

Ensembl Gene ID	Ensembl Gene Name	Ensembl Gene Description	Gene Type	Log_2 Fold Change (Day 6 vs. pre-treatment)	Average Log_2 CPM	Likelihood Ratio Test Statistic	P-Value	FDR Adjusted P-Value
ENSG00000074695	LMAN1	lectin, mannose binding 1 [Source:HGNC Symbol;Acc:HGNC:6631]	protein coding	1.12	8.04	30.89	0.00	0.00
ENSG00000185155	MIXL1	Mix paired-like homeobox [Source:HGNC Symbol;Acc:HGNC:13363]	protein coding	1.11	2.93	13.92	0.00	0.00
ENSG00000103257	SLC7A5	solute carrier family 7 member 5 [Source:HGNC Symbol;Acc:HGNC:11063]	protein coding	1.11	5.44	22.43	0.00	0.00
ENSG00000182985	CADM1	cell adhesion molecule 1 [Source:HGNC Symbol;Acc:HGNC:5951]	protein coding	1.11	3.50	17.99	0.00	0.00
ENSG00000134285	FKBP11	FK506 binding protein 11 [Source:HGNC Symbol;Acc:HGNC:18624]	protein coding	1.10	6.38	24.28	0.00	0.00
ENSG00000111665	CDCA3	cell division cycle associated 3 [Source:HGNC Symbol;Acc:HGNC:14624]	protein coding	1.10	2.27	10.46	0.00	0.02
ENSG00000090889	KIF4A	kinesin family member 4A [Source:HGNC Symbol;Acc:HGNC:13339]	protein coding	1.10	2.45	15.66	0.00	0.00
ENSG00000068912	ERLEC1	endoplasmic reticulum lectin 1 [Source:HGNC Symbol;Acc:HGNC:25222]	protein coding	1.10	6.77	30.22	0.00	0.00
ENSG00000211892	IGHG4	immunoglobulin heavy constant gamma 4 (G4m marker) [Source:HGNC Symbol;Acc:HGNC:5528]	IG C gene	1.10	7.27	19.42	0.00	0.00
ENSG00000157456	CCNB2	cyclin B2 [Source:HGNC Symbol;Acc:HGNC:1580]	protein coding	1.09	3.16	16.06	0.00	0.00
ENSG00000174371	EXO1	exonuclease 1 [Source:HGNC Symbol;Acc:HGNC:3511]	protein coding	1.09	1.91	12.46	0.00	0.01
ENSG00000211972	IGHV3-66	immunoglobulin heavy variable 3-66 [Source:HGNC Symbol;Acc:HGNC:5619]	IG V gene	1.09	4.70	15.42	0.00	0.00
ENSG00000092853	CLSPN	claspin [Source:HGNC Symbol;Acc:HGNC:19715]	protein coding	1.09	2.57	12.97	0.00	0.01
ENSG00000237649	KIFC1	kinesin family member C1 [Source:HGNC Symbol;Acc:HGNC:6389]	protein coding	1.08	3.34	18.82	0.00	0.00
ENSG00000065308	TRAM2	translocation associated membrane protein 2 [Source:HGNC Symbol;Acc:HGNC:16855]	protein coding	1.08	7.25	25.43	0.00	0.00
ENSG00000198876	DCAF12	DDB1 and CUL4 associated factor 12 [Source:HGNC Symbol;Acc:HGNC:19911]	protein coding	1.08	4.62	27.92	0.00	0.00
ENSG00000196189	SEMA4A	semaphorin 4A [Source:HGNC Symbol;Acc:HGNC:10729]	protein coding	1.08	6.80	36.95	0.00	0.00
ENSG00000222041	CYTOR	cytoskeleton regulator RNA [Source:HGNC Symbol;Acc:HGNC:28717]	lincRNA	1.07	2.23	13.34	0.00	0.01
ENSG00000186818	LILRB4	leukocyte immunoglobulin like receptor B4 [Source:HGNC Symbol;Acc:HGNC:6608]	protein coding	1.07	3.86	14.82	0.00	0.00
ENSG00000117399	CDC20	cell division cycle 20 [Source:HGNC Symbol;Acc:HGNC:1723]	protein coding	1.07	3.49	13.98	0.00	0.00
ENSG00000239264	TXNDC5	thioredoxin domain containing 5 [Source:HGNC Symbol;Acc:HGNC:21073]	protein coding	1.07	5.77	20.40	0.00	0.00
ENSG00000111206	FOXM1	forkhead box M1 [Source:HGNC Symbol;Acc:HGNC:3818]	protein coding	1.07	3.37	18.95	0.00	0.00
ENSG00000180535	BHLHA15	basic helix-loop-helix family member a15 [Source:HGNC Symbol;Acc:HGNC:22265]	protein coding	1.06	3.35	15.31	0.00	0.00

Ensembl Gene ID	Ensembl Gene Name	Ensembl Gene Description	Gene Type	Log_2 Fold Change (Day 6 vs. pre-treatment)	Average Log_2 CPM	Likelihood Ratio Test Statistic	P-Value	FDR Adjusted P-Value
ENSG00000153066	TXNDC11	thioredoxin domain containing 11 [Source:HGNC Symbol;Acc:HGNC:28030]	protein coding	1.06	7.80	27.06	0.00	0.00
ENSG00000163694	RBM47	RNA binding motif protein 47 [Source:HGNC Symbol;Acc:HGNC:30358]	protein coding	1.06	4.95	20.77	0.00	0.00
ENSG00000177301	KCNA2	potassium voltage-gated channel subfamily A member 2 [Source:HGNC Symbol;Acc:HGNC:6220]	protein coding	1.06	3.14	12.14	0.00	0.01
ENSG00000211673	IGLV3-1	immunoglobulin lambda variable 3-1 [Source:HGNC Symbol;Acc:HGNC:5896]	IG V gene	1.06	7.88	42.61	0.00	0.00
ENSG00000170476	MZB1	marginal zone B and B1 cell specific protein [Source:HGNC Symbol;Acc:HGNC:30125]	protein coding	1.06	8.58	20.36	0.00	0.00
ENSG00000143603	KCNN3	potassium calcium-activated channel subfamily N member 3 [Source:HGNC Symbol;Acc:HGNC:6292]	protein coding	1.06	3.01	13.36	0.00	0.01
ENSG00000143942	CHAC2	ChaC cation transport regulator homolog 2 [Source:HGNC Symbol;Acc:HGNC:32363]	protein coding	1.06	3.21	22.88	0.00	0.00
ENSG00000136026	CKAP4	cytoskeleton associated protein 4 [Source:HGNC Symbol;Acc:HGNC:16991]	protein coding	1.05	6.75	30.57	0.00	0.00
ENSG00000118705	RPN2	ribophorin II [Source:HGNC Symbol;Acc:HGNC:10382]	protein coding	1.05	8.46	26.50	0.00	0.00
ENSG00000128595	CALU	calumenin [Source:HGNC Symbol;Acc:HGNC:1458]	protein coding	1.05	6.30	32.36	0.00	0.00
ENSG00000150961	SEC24D	SEC24 homolog D, COPII coat complex component [Source:HGNC Symbol;Acc:HGNC:10706]	protein coding	1.05	6.61	29.15	0.00	0.00
ENSG00000153162	BMP6	bone morphogenetic protein 6 [Source:HGNC Symbol;Acc:HGNC:1073]	protein coding	1.04	2.43	8.64	0.00	0.04
ENSG00000142945	KIF2C	kinesin family member 2C [Source:HGNC Symbol;Acc:HGNC:6393]	protein coding	1.04	2.80	19.60	0.00	0.00
ENSG00000241294	IGKV2-24	immunoglobulin kappa variable 2-24 [Source:HGNC Symbol;Acc:HGNC:5781]	IG V gene	1.04	5.25	12.16	0.00	0.01
ENSG00000211897	IGHG3	immunoglobulin heavy constant gamma 3 (G3m marker) [Source:HGNC Symbol;Acc:HGNC:5527]	IG C gene	1.04	9.26	14.98	0.00	0.00
ENSG00000134910	STT3A	STT3A, catalytic subunit of the oligosaccharyltransferase complex [Source:HGNC Symbol;Acc:HGNC:6172]	protein coding	1.04	7.40	32.09	0.00	0.00
ENSG00000155304	HSPA13	heat shock protein family A (Hsp70) member 13 [Source:HGNC Symbol;Acc:HGNC:11375]	protein coding	1.03	6.30	32.29	0.00	0.00
ENSG00000158089	GALNT14	polypeptide N-acetylgalactosaminyltransferase 14 [Source:HGNC Symbol;Acc:HGNC:22946]	protein coding	1.03	1.57	8.24	0.00	0.04
ENSG00000145386	CCNA2	cyclin A2 [Source:HGNC Symbol;Acc:HGNC:1578]	protein coding	1.03	3.67	19.98	0.00	0.00
ENSG00000131871	SELENOS	selenoprotein S [Source:HGNC Symbol;Acc:HGNC:30396]	protein coding	1.03	5.62	28.70	0.00	0.00
ENSG00000183508	FAM46C	family with sequence similarity 46 member C [Source:HGNC Symbol;Acc:HGNC:24712]	protein coding	1.03	8.59	29.80	0.00	0.00
ENSG00000172469	MANEA	mannosidase endo-alpha [Source:HGNC Symbol;Acc:HGNC:21072]	protein coding	1.03	6.01	26.63	0.00	0.00

Ensembl Gene ID	Ensembl Gene Name	Ensembl Gene Description	Gene Type	Log_2 Fold Change (Day 6 vs. pre-treatment)	Average Log_2 CPM	Likelihood Ratio Test Statistic	P-Value	FDR Adjusted P-Value
ENSG00000184232	OAF	out at first homolog [Source:HGNC Symbol;Acc:HGNC:28752]	protein coding	1.03	2.27	13.19	0.00	0.01
ENSG00000125844	RRBP1	ribosome binding protein 1 [Source:HGNC Symbol;Acc:HGNC:10448]	protein coding	1.02	7.32	20.42	0.00	0.00
ENSG00000186522	SEPT10	septin 10 [Source:HGNC Symbol;Acc:HGNC:14349]	protein coding	1.02	2.15	11.89	0.00	0.01
ENSG00000149428	HYOU1	hypoxia up-regulated 1 [Source:HGNC Symbol;Acc:HGNC:16931]	protein coding	1.02	7.84	29.75	0.00	0.00
ENSG00000092621	PHGDH	phosphoglycerate dehydrogenase [Source:HGNC Symbol;Acc:HGNC:8923]	protein coding	1.02	3.86	28.26	0.00	0.00
ENSG00000166803	KIAA0101	KIAA0101 [Source:HGNC Symbol;Acc:HGNC:28961]	protein coding	1.01	1.52	7.92	0.00	0.05
ENSG00000118193	KIF14	kinesin family member 14 [Source:HGNC Symbol;Acc:HGNC:19181]	protein coding	1.01	2.53	11.59	0.00	0.01
ENSG00000151725	CENPU	centromere protein U [Source:HGNC Symbol;Acc:HGNC:21348]	protein coding	1.01	3.18	21.51	0.00	0.00
ENSG00000211659	IGLV3-25	immunoglobulin lambda variable 3-25 [Source:HGNC Symbol;Acc:HGNC:5908]	IG V gene	1.01	7.14	30.89	0.00	0.00
ENSG00000114850	SSR3	signal sequence receptor subunit 3 [Source:HGNC Symbol;Acc:HGNC:11325]	protein coding	1.01	7.86	34.45	0.00	0.00
ENSG00000143870	PDIA6	protein disulfide isomerase family A member 6 [Source:HGNC Symbol;Acc:HGNC:30168]	protein coding	1.00	7.75	31.47	0.00	0.00
ENSG00000167900	TK1	thymidine kinase 1 [Source:HGNC Symbol;Acc:HGNC:11830]	protein coding	1.00	3.93	35.18	0.00	0.00
ENSG00000078900	TP73	tumor protein p73 [Source:HGNC Symbol;Acc:HGNC:12003]	protein coding	1.00	2.00	8.05	0.00	0.04
ENSG00000244575	IGKV1-27	immunoglobulin kappa variable 1-27 [Source:HGNC Symbol;Acc:HGNC:5735]	IG V gene	1.00	5.65	66.03	0.00	0.00
ENSG00000258572			lincRNA	1.00	2.63	10.36	0.00	0.02
ENSG00000111291	GPRC5D	G protein-coupled receptor class C group 5 member D [Source:HGNC Symbol;Acc:HGNC:13310]	protein coding	1.00	3.91	20.56	0.00	0.00
ENSG00000189233	NUGGC	nuclear GTPase, germinal center associated [Source:HGNC Symbol;Acc:HGNC:33550]	protein coding	0.99	4.95	19.04	0.00	0.00
ENSG00000171155	C1GALT1C1	C1GALT1 specific chaperone 1 [Source:HGNC Symbol;Acc:HGNC:24338]	protein coding	0.99	4.14	19.78	0.00	0.00
ENSG00000132432	SEC61G	Sec61 translocon gamma subunit [Source:HGNC Symbol;Acc:HGNC:18277]	protein coding	0.99	5.36	37.96	0.00	0.00
ENSG00000211669	IGLV3-10	immunoglobulin lambda variable 3-10 [Source:HGNC Symbol;Acc:HGNC:5897]	IG V gene	0.99	5.88	23.17	0.00	0.00
ENSG00000224373	IGHV4-59	immunoglobulin heavy variable 4-59 [Source:HGNC Symbol;Acc:HGNC:5654]	IG V gene	0.98	6.51	19.49	0.00	0.00
ENSG00000108829	LRRC59	leucine rich repeat containing 59 [Source:HGNC Symbol;Acc:HGNC:28817]	protein coding	0.98	6.63	31.60	0.00	0.00
ENSG00000051341	POLQ	DNA polymerase theta [Source:HGNC Symbol;Acc:HGNC:9186]	protein coding	0.98	3.02	24.35	0.00	0.00

Ensembl Gene ID	Ensembl Gene Name	Ensembl Gene Description	Gene Type	Log_2 Fold Change (Day 6 vs. pre-treatment)	Average Log_2 CPM	Likelihood Ratio Test Statistic	P-Value	FDR Adjusted P-Value
ENSG00000124788	ATXN1	ataxin 1 [Source:HGNC Symbol;Acc:HGNC:10548]	protein coding	0.98	5.03	25.84	0.00	0.00
ENSG00000156970	BUB1B	BUB1 mitotic checkpoint serine/threonine kinase B [Source:HGNC Symbol;Acc:HGNC:1149]	protein coding	0.98	2.88	16.22	0.00	0.00
ENSG00000106415	GLCCI1	glucocorticoid induced 1 [Source:HGNC Symbol;Acc:HGNC:18713]	protein coding	0.97	6.79	35.12	0.00	0.00
ENSG00000179750	APOBEC3B	apolipoprotein B mRNA editing enzyme catalytic subunit 3B [Source:HGNC Symbol;Acc:HGNC:17352]	protein coding	0.97	2.88	12.57	0.00	0.01
ENSG00000017483	SLC38A5	solute carrier family 38 member 5 [Source:HGNC Symbol;Acc:HGNC:18070]	protein coding	0.97	5.11	23.88	0.00	0.00
ENSG00000087586	AURKA	aurora kinase A [Source:HGNC Symbol;Acc:HGNC:11393]	protein coding	0.96	2.46	10.84	0.00	0.01
ENSG00000136770	DNAJC1	DnaJ heat shock protein family (Hsp40) member C1 [Source:HGNC Symbol;Acc:HGNC:20090]	protein coding	0.96	4.94	19.69	0.00	0.00
ENSG00000123352	SPATS2	spermatogenesis associated serine rich 2 [Source:HGNC Symbol;Acc:HGNC:18650]	protein coding	0.95	5.70	25.96	0.00	0.00
ENSG00000165948	IFI27L1	interferon alpha inducible protein 27 like 1 [Source:HGNC Symbol;Acc:HGNC:19754]	protein coding	0.95	2.59	11.66	0.00	0.01
ENSG00000198855	FICD	FIC domain containing [Source:HGNC Symbol;Acc:HGNC:18416]	protein coding	0.95	3.29	17.16	0.00	0.00
ENSG00000185624	P4HB	prolyl 4-hydroxylase subunit beta [Source:HGNC Symbol;Acc:HGNC:8548]	protein coding	0.95	8.44	24.79	0.00	0.00
ENSG00000135069	PSAT1	phosphoserine aminotransferase 1 [Source:HGNC Symbol;Acc:HGNC:19129]	protein coding	0.95	3.62	24.40	0.00	0.00
ENSG00000154839	SKA1	spindle and kinetochore associated complex subunit 1 [Source:HGNC Symbol;Acc:HGNC:28109]	protein coding	0.95	2.29	9.22	0.00	0.03
ENSG00000057657	PRDM1	PR/SET domain 1 [Source:HGNC Symbol;Acc:HGNC:9346]	protein coding	0.94	6.32	22.06	0.00	0.00
ENSG00000254395	IGHV4-55	immunoglobulin heavy variable 4-55 (pseudogene) [Source:HGNC Symbol;Acc:HGNC:5653]	IG V pseudogene	0.94	4.19	14.43	0.00	0.00
ENSG00000137804	NUSAP1	nucleolar and spindle associated protein 1 [Source:HGNC Symbol;Acc:HGNC:18538]	protein coding	0.94	4.33	24.73	0.00	0.00
ENSG00000074416	MGLL	monoglyceride lipase [Source:HGNC Symbol;Acc:HGNC:17038]	protein coding	0.94	3.79	20.74	0.00	0.00
ENSG00000117143	UAP1	UDP-N-acetylglucosamine pyrophosphorylase 1 [Source:HGNC Symbol;Acc:HGNC:12457]	protein coding	0.94	5.73	23.71	0.00	0.00
ENSG00000134057	CCNB1	cyclin B1 [Source:HGNC Symbol;Acc:HGNC:1579]	protein coding	0.94	3.49	14.57	0.00	0.00
ENSG00000165272	AQP3	aquaporin 3 (Gill blood group) [Source:HGNC Symbol;Acc:HGNC:636]	protein coding	0.94	6.16	19.11	0.00	0.00
ENSG00000131153	GINS2	GINS complex subunit 2 [Source:HGNC Symbol;Acc:HGNC:24575]	protein coding	0.94	2.45	13.18	0.00	0.01
ENSG00000090520	DNAJB11	DnaJ heat shock protein family (Hsp40) member B11 [Source:HGNC Symbol;Acc:HGNC:14889]	protein coding	0.93	6.17	35.76	0.00	0.00

Ensembl Gene ID	Ensembl Gene Name	Ensembl Gene Description	Gene Type	Log_2 Fold Change (Day 6 vs. pre-treatment)	Average Log_2 CPM	Likelihood Ratio Test Statistic	P-Value	FDR Adjusted P-Value
ENSG00000106803	SEC61B	Sec61 translocon beta subunit [Source:HGNC Symbol;Acc:HGNC:16993]	protein coding	0.93	6.16	22.82	0.00	0.00
ENSG00000100342	APOL1	apolipoprotein L1 [Source:HGNC Symbol;Acc:HGNC:618]	protein coding	0.93	4.82	18.24	0.00	0.00
ENSG00000241755	IGKV1-9	immunoglobulin kappa variable 1-9 [Source:HGNC Symbol;Acc:HGNC:5744]	IG V gene	0.93	6.19	8.66	0.00	0.04
ENSG00000070540	WIPI1	WD repeat domain, phosphoinositide interacting 1 [Source:HGNC Symbol;Acc:HGNC:25471]	protein coding	0.93	4.29	12.99	0.00	0.01
ENSG00000167476	JSRP1	junctional sarcoplasmic reticulum protein 1 [Source:HGNC Symbol;Acc:HGNC:24963]	protein coding	0.92	2.50	8.28	0.00	0.04
ENSG00000160712	IL6R	interleukin 6 receptor [Source:HGNC Symbol;Acc:HGNC:6019]	protein coding	0.92	6.53	24.81	0.00	0.00
ENSG00000211943	IGHV3-15	immunoglobulin heavy variable 3-15 [Source:HGNC Symbol;Acc:HGNC:5582]	IG V gene	0.92	7.06	11.83	0.00	0.01
ENSG00000163902	RPN1	ribophorin I [Source:HGNC Symbol;Acc:HGNC:10381]	protein coding	0.91	7.98	29.71	0.00	0.00
ENSG00000102580	DNAJC3	DnaJ heat shock protein family (Hsp40) member C3 [Source:HGNC Symbol;Acc:HGNC:9439]	protein coding	0.91	6.80	30.69	0.00	0.00
ENSG00000035499	DEPDC1B	DEP domain containing 1B [Source:HGNC Symbol;Acc:HGNC:24902]	protein coding	0.91	2.26	9.07	0.00	0.03
ENSG00000080986	NDC80	NDC80, kinetochore complex component [Source:HGNC Symbol;Acc:HGNC:16909]	protein coding	0.91	3.20	14.42	0.00	0.00
ENSG00000138160	KIF11	kinesin family member 11 [Source:HGNC Symbol;Acc:HGNC:6388]	protein coding	0.91	4.65	24.89	0.00	0.00
ENSG00000241666			antisense	-0.91	2.89	21.76	0.00	0.00
ENSG00000149554	CHEK1	checkpoint kinase 1 [Source:HGNC Symbol;Acc:HGNC:1925]	protein coding	0.91	3.04	13.17	0.00	0.01
ENSG00000064763	FAR2	fatty acyl-CoA reductase 2 [Source:HGNC Symbol;Acc:HGNC:25531]	protein coding	0.91	2.16	9.04	0.00	0.03
ENSG00000117724	CENPF	centromere protein F [Source:HGNC Symbol;Acc:HGNC:1857]	protein coding	0.91	4.59	18.72	0.00	0.00
ENSG00000143228	NUF2	NUF2, NDC80 kinetochore complex component [Source:HGNC Symbol;Acc:HGNC:14621]	protein coding	0.91	2.59	14.44	0.00	0.00
ENSG00000150967	ABCB9	ATP binding cassette subfamily B member 9 [Source:HGNC Symbol;Acc:HGNC:50]	protein coding	0.91	4.01	8.67	0.00	0.04
ENSG00000251546	IGKV1D-39	immunoglobulin kappa variable 1D-39 [Source:HGNC Symbol;Acc:HGNC:5756]	IG V gene	0.91	4.82	18.19	0.00	0.00
ENSG00000071537	SEL1L	SEL1L ERAD E3 ligase adaptor subunit [Source:HGNC Symbol;Acc:HGNC:10717]	protein coding	0.90	7.66	24.57	0.00	0.00
ENSG00000244116	IGKV2-28	immunoglobulin kappa variable 2-28 [Source:HGNC Symbol;Acc:HGNC:5783]	IG V gene	0.90	4.00	24.62	0.00	0.00
ENSG00000183087	GAS6	growth arrest specific 6 [Source:HGNC Symbol;Acc:HGNC:4168]	protein coding	0.90	3.67	12.04	0.00	0.01
ENSG00000244038	DDOST	dolichyl-diphosphooligosaccharide–protein glycosyltransferase non-catalytic subunit [Source:HGNC Symbol;Acc:HGNC:2728]	protein coding	0.90	7.95	28.08	0.00	0.00

Ensembl Gene ID	Ensembl Gene Name	Ensembl Gene Description	Gene Type	Log_2 Fold Change (Day 6 vs. pre-treatment)	Average Log_2 CPM	Likelihood Ratio Test Statistic	P-Value	FDR Adjusted P-Value
ENSG00000278857	IGKV1D-12	immunoglobulin kappa variable 1D-12 [Source:HGNC Symbol;Acc:HGNC:5746]	IG V gene	0.89	2.63	7.82	0.01	0.05
ENSG00000115677	HDLBP	high density lipoprotein binding protein [Source:HGNC Symbol;Acc:HGNC:4857]	protein coding	0.89	8.63	23.22	0.00	0.00
ENSG00000128590	DNAJB9	DnaJ heat shock protein family (Hsp40) member B9 [Source:HGNC Symbol;Acc:HGNC:6968]	protein coding	0.89	5.26	32.18	0.00	0.00
ENSG00000198856	OSTC	oligosaccharyltransferase complex non-catalytic subunit [Source:HGNC Symbol;Acc:HGNC:24448]	protein coding	0.89	6.21	33.17	0.00	0.00
ENSG00000176658	MYO1D	myosin ID [Source:HGNC Symbol;Acc:HGNC:7598]	protein coding	0.89	6.04	17.53	0.00	0.00
ENSG00000101294	HM13	histocompatibility minor 13 [Source:HGNC Symbol;Acc:HGNC:16435]	protein coding	0.89	7.10	21.48	0.00	0.00
ENSG00000115902	SLC1A4	solute carrier family 1 member 4 [Source:HGNC Symbol;Acc:HGNC:10942]	protein coding	0.89	6.16	21.52	0.00	0.00
ENSG00000139193	CD27	CD27 molecule [Source:HGNC Symbol;Acc:HGNC:11922]	protein coding	0.89	6.01	17.00	0.00	0.00
ENSG00000203760	CENPW	centromere protein W [Source:HGNC Symbol;Acc:HGNC:21488]	protein coding	0.89	1.66	7.78	0.01	0.05
ENSG00000185480	PARPBP	PARP1 binding protein [Source:HGNC Symbol;Acc:HGNC:26074]	protein coding	0.89	2.30	10.37	0.00	0.02
ENSG00000198826	ARHGAP11A	Rho GTPase activating protein 11A [Source:HGNC Symbol;Acc:HGNC:15783]	protein coding	0.89	4.01	25.37	0.00	0.00
ENSG00000076382	SPAG5	sperm associated antigen 5 [Source:HGNC Symbol;Acc:HGNC:13452]	protein coding	0.88	3.81	14.89	0.00	0.00
ENSG00000122862	SRGN	serglycin [Source:HGNC Symbol;Acc:HGNC:9361]	protein coding	0.88	7.26	30.49	0.00	0.00
ENSG00000133328	HRASLS2	HRAS like suppressor 2 [Source:HGNC Symbol;Acc:HGNC:17824]	protein coding	0.88	3.14	9.55	0.00	0.03
ENSG00000235162	C12orf75	chromosome 12 open reading frame 75 [Source:HGNC Symbol;Acc:HGNC:35164]	protein coding	0.87	4.74	26.30	0.00	0.00
ENSG00000249096			lincRNA	0.87	2.71	8.14	0.00	0.04
ENSG00000105011	ASF1B	anti-silencing function 1B histone chaperone [Source:HGNC Symbol;Acc:HGNC:20996]	protein coding	0.87	3.11	12.56	0.00	0.01
ENSG00000182054	IDH2	isocitrate dehydrogenase (NADP(+)) 2, mitochondrial [Source:HGNC Symbol;Acc:HGNC:5383]	protein coding	0.87	6.79	19.19	0.00	0.00
ENSG00000117411	B4GALT2	beta-1,4-galactosyltransferase 2 [Source:HGNC Symbol;Acc:HGNC:925]	protein coding	0.87	3.44	13.63	0.00	0.00
ENSG00000164109	MAD2L1	MAD2 mitotic arrest deficient-like 1 (yeast) [Source:HGNC Symbol;Acc:HGNC:6763]	protein coding	0.87	3.28	18.20	0.00	0.00
ENSG00000077152	UBE2T	ubiquitin conjugating enzyme E2 T [Source:HGNC Symbol;Acc:HGNC:25009]	protein coding	0.87	2.14	8.25	0.00	0.04
ENSG00000083444	PLOD1	procollagen-lysine,2-oxoglutarate 5-dioxygenase 1 [Source:HGNC Symbol;Acc:HGNC:9081]	protein coding	0.86	4.47	15.74	0.00	0.00
ENSG00000241351	IGKV3-11	immunoglobulin kappa variable 3-11 [Source:HGNC Symbol;Acc:HGNC:5815]	IG V gene	0.86	7.59	15.37	0.00	0.00

Ensembl Gene ID	Ensembl Gene Name	Ensembl Gene Description	Gene Type	Log_2 Fold Change (Day 6 vs. pre-treatment)	Average Log_2 CPM	Likelihood Ratio Test Statistic	P-Value	FDR Adjusted P-Value
ENSG00000101003	GINS1	GINS complex subunit 1 [Source:HGNC Symbol;Acc:HGNC:28980]	protein coding	0.86	2.43	10.38	0.00	0.02
ENSG00000136240	KDELR2	KDEL endoplasmic reticulum protein retention receptor 2 [Source:HGNC Symbol;Acc:HGNC:6305]	protein coding	0.86	6.27	30.35	0.00	0.00
ENSG00000123080	CDKN2C	cyclin dependent kinase inhibitor 2C [Source:HGNC Symbol;Acc:HGNC:1789]	protein coding	0.86	2.81	15.13	0.00	0.00
ENSG00000175984	DENND2C	DENN domain containing 2C [Source:HGNC Symbol;Acc:HGNC:24748]	protein coding	0.85	2.06	7.86	0.01	0.05
ENSG00000242371	IGKV1-39	immunoglobulin kappa variable 1-39 (gene/pseudogene) [Source:HGNC Symbol;Acc:HGNC:5740]	IG V gene	0.85	4.34	12.02	0.00	0.01
ENSG00000168209	DDIT4	DNA damage inducible transcript 4 [Source:HGNC Symbol;Acc:HGNC:24944]	protein coding	-0.85	4.57	34.62	0.00	0.00
ENSG00000228589	SPCS2P4	signal peptidase complex subunit 2 homolog (S. cerevisiae) pseudogene 4 [Source:HGNC Symbol;Acc:HGNC:45237]	processed pseudogene	0.84	2.23	8.69	0.00	0.04
ENSG00000211976	IGHV3-73	immunoglobulin heavy variable 3-73 [Source:HGNC Symbol;Acc:HGNC:5623]	IG V gene	0.84	4.70	11.89	0.00	0.01
ENSG00000198937	CCDC167	coiled-coil domain containing 167 [Source:HGNC Symbol;Acc:HGNC:21239]	protein coding	0.84	3.78	22.96	0.00	0.00
ENSG00000004866	ST7	suppression of tumorigenicity 7 [Source:HGNC Symbol;Acc:HGNC:11351]	protein coding	0.84	2.91	12.46	0.00	0.01
ENSG00000173540	GMPPB	GDP-mannose pyrophosphorylase B [Source:HGNC Symbol;Acc:HGNC:22932]	protein coding	0.84	5.46	25.55	0.00	0.00
ENSG00000135476	ESPL1	extra spindle pole bodies like 1, separase [Source:HGNC Symbol;Acc:HGNC:16856]	protein coding	0.84	2.83	9.83	0.00	0.02
ENSG00000102595	UGGT2	UDP-glucose glycoprotein glucosyltransferase 2 [Source:HGNC Symbol;Acc:HGNC:15664]	protein coding	0.83	2.74	10.32	0.00	0.02
ENSG00000112312	GMNN	geminin, DNA replication inhibitor [Source:HGNC Symbol;Acc:HGNC:17493]	protein coding	0.83	3.06	12.57	0.00	0.01
ENSG00000065911	MTHFD2	methylenetetrahydrofolate dehydrogenase (NADP+ dependent) 2, methenyltetrahydrofolate cyclohydrolase [Source:HGNC Symbol;Acc:HGNC:7434]	protein coding	0.83	5.34	20.74	0.00	0.00
ENSG00000168496	FEN1	flap structure-specific endonuclease 1 [Source:HGNC Symbol;Acc:HGNC:3650]	protein coding	0.83	4.36	16.89	0.00	0.00
ENSG00000211935	IGHV1-3	immunoglobulin heavy variable 1-3 [Source:HGNC Symbol;Acc:HGNC:5552]	IG V gene	0.83	6.30	10.35	0.00	0.02
ENSG00000163170	BOLA3	bolA family member 3 [Source:HGNC Symbol;Acc:HGNC:24415]	protein coding	0.83	2.23	10.00	0.00	0.02
ENSG00000103512	NOMO1	NODAL modulator 1 [Source:HGNC Symbol;Acc:HGNC:30060]	protein coding	0.83	5.39	13.57	0.00	0.00
ENSG00000163808	KIF15	kinesin family member 15 [Source:HGNC Symbol;Acc:HGNC:17273]	protein coding	0.83	2.75	10.53	0.00	0.02
ENSG00000211679	IGLC3	immunoglobulin lambda constant 3 (Kern-Oz+ marker) [Source:HGNC Symbol;Acc:HGNC:5857]	IG C gene	0.83	9.33	10.91	0.00	0.01
ENSG00000136868	SLC31A1	solute carrier family 31 member 1 [Source:HGNC Symbol;Acc:HGNC:11016]	protein coding	0.83	3.96	17.84	0.00	0.00

Ensembl Gene ID	Ensembl Gene Name	Ensembl Gene Description	Gene Type	Log_2 Fold Change (Day 6 vs. pre-treatment)	Average Log_2 CPM	Likelihood Ratio Test Statistic	P-Value	FDR Adjusted P-Value
ENSG00000109501	WFS1	wolframin ER transmembrane glycoprotein [Source:HGNC Symbol;Acc:HGNC:12762]	protein coding	0.82	4.09	17.93	0.00	0.00
ENSG00000121073	SLC35B1	solute carrier family 35 member B1 [Source:HGNC Symbol;Acc:HGNC:20798]	protein coding	0.82	5.38	22.89	0.00	0.00
ENSG00000198018	ENTPD7	ectonucleoside triphosphate diphosphohydrolase 7 [Source:HGNC Symbol;Acc:HGNC:19745]	protein coding	0.82	3.33	14.85	0.00	0.00
ENSG00000120725	SIL1	SIL1 nucleotide exchange factor [Source:HGNC Symbol;Acc:HGNC:24624]	protein coding	0.82	4.33	13.15	0.00	0.01
ENSG00000100228	RAB36	RAB36, member RAS oncogene family [Source:HGNC Symbol;Acc:HGNC:9775]	protein coding	0.82	2.44	8.02	0.00	0.05
ENSG00000102096	PIM2	Pim-2 proto-oncogene, serine/threonine kinase [Source:HGNC Symbol;Acc:HGNC:8987]	protein coding	0.82	7.96	23.94	0.00	0.00
ENSG00000068489	PRR11	proline rich 11 [Source:HGNC Symbol;Acc:HGNC:25619]	protein coding	0.82	3.68	18.68	0.00	0.00
ENSG00000058262	SEC61A1	Sec61 translocon alpha 1 subunit [Source:HGNC Symbol;Acc:HGNC:18276]	protein coding	0.82	8.38	21.73	0.00	0.00
ENSG00000168701	TMEM208	transmembrane protein 208 [Source:HGNC Symbol;Acc:HGNC:25015]	protein coding	0.82	4.09	18.12	0.00	0.00
ENSG00000101412	E2F1	E2F transcription factor 1 [Source:HGNC Symbol;Acc:HGNC:3113]	protein coding	0.82	2.70	8.92	0.00	0.03
ENSG00000134825	TMEM258	transmembrane protein 258 [Source:HGNC Symbol;Acc:HGNC:1164]	protein coding	0.81	5.75	25.73	0.00	0.00
ENSG00000211685	IGLC7	immunoglobulin lambda constant 7 [Source:HGNC Symbol;Acc:HGNC:5861]	IG C gene	0.81	4.89	13.95	0.00	0.00
ENSG00000006638	TBXA2R	thromboxane A2 receptor [Source:HGNC Symbol;Acc:HGNC:11608]	protein coding	-0.81	2.88	14.51	0.00	0.00
ENSG00000136161	RCBTB2	RCC1 and BTB domain containing protein 2 [Source:HGNC Symbol;Acc:HGNC:1914]	protein coding	0.81	4.34	10.75	0.00	0.02
ENSG00000155380	SLC16A1	solute carrier family 16 member 1 [Source:HGNC Symbol;Acc:HGNC:10922]	protein coding	0.81	4.29	19.65	0.00	0.00
ENSG00000099337	KCNK6	potassium two pore domain channel subfamily K member 6 [Source:HGNC Symbol;Acc:HGNC:6281]	protein coding	0.81	5.23	23.33	0.00	0.00
ENSG00000184164	CRELD2	cysteine rich with EGF like domains 2 [Source:HGNC Symbol;Acc:HGNC:28150]	protein coding	0.79	5.95	23.19	0.00	0.00
ENSG00000274576	IGHV2-70	immunoglobulin heavy variable 2-70 [Source:HGNC Symbol;Acc:HGNC:5577]	IG V gene	0.79	3.98	12.88	0.00	0.01
ENSG00000136810	TXN	thioredoxin [Source:HGNC Symbol;Acc:HGNC:12435]	protein coding	0.79	5.12	16.59	0.00	0.00
ENSG00000211933	IGHV6-1	immunoglobulin heavy variable 6-1 [Source:HGNC Symbol;Acc:HGNC:5662]	IG V gene	0.79	6.14	37.11	0.00	0.00
ENSG00000136052	SLC41A2	solute carrier family 41 member 2 [Source:HGNC Symbol;Acc:HGNC:31045]	protein coding	0.79	2.36	8.68	0.00	0.04
ENSG00000011478	QPCTL	glutaminyl-peptide cyclotransferase like [Source:HGNC Symbol;Acc:HGNC:25952]	protein coding	0.79	4.35	13.30	0.00	0.01

Ensembl Gene ID	Ensembl Gene Name	Ensembl Gene Description	Gene Type	Log_2 Fold Change (Day 6 vs. pre-treatment)	Average Log_2 CPM	Likelihood Ratio Test Statistic	P-Value	FDR Adjusted P-Value
ENSG00000213430	HSPD1P1	heat shock protein family D (Hsp60) member 1 pseudogene 1 [Source:HGNC Symbol;Acc:HGNC:35133]	processed pseudogene	0.79	2.21	10.19	0.00	0.02
ENSG00000224041	IGKV3D-15	immunoglobulin kappa variable 3D-15 (gene/pseudogene) [Source:HGNC Symbol;Acc:HGNC:5824]	IG V gene	0.78	4.58	14.20	0.00	0.00
ENSG00000179218	CALR	calreticulin [Source:HGNC Symbol;Acc:HGNC:1455]	protein coding	0.78	9.29	24.44	0.00	0.00
ENSG00000204386	NEU1	neuraminidase 1 [Source:HGNC Symbol;Acc:HGNC:7758]	protein coding	0.78	5.02	26.67	0.00	0.00
ENSG00000211592	IGKC	immunoglobulin kappa constant [Source:HGNC Symbol;Acc:HGNC:5716]	IG C gene	0.77	13.09	11.80	0.00	0.01
ENSG00000279873	LINC01126	long intergenic non-protein coding RNA 1126 [Source:HGNC Symbol;Acc:HGNC:49275]	lincRNA	-0.77	2.66	10.15	0.00	0.02
ENSG00000123975	CKS2	CDC28 protein kinase regulatory subunit 2 [Source:HGNC Symbol;Acc:HGNC:2000]	protein coding	0.77	3.23	14.97	0.00	0.00
ENSG00000211625	IGKV3D-20	immunoglobulin kappa variable 3D-20 [Source:HGNC Symbol;Acc:HGNC:5825]	IG V gene	0.77	5.49	12.45	0.00	0.01
ENSG00000103226	NOMO3	NODAL modulator 3 [Source:HGNC Symbol;Acc:HGNC:25242]	protein coding	0.77	3.09	14.85	0.00	0.00
ENSG00000232216	IGHV3-43	immunoglobulin heavy variable 3-43 [Source:HGNC Symbol;Acc:HGNC:5604]	IG V gene	0.76	5.19	13.08	0.00	0.01
ENSG00000172548	NIPAL4	NIPA like domain containing 4 [Source:HGNC Symbol;Acc:HGNC:28018]	protein coding	-0.76	3.06	18.32	0.00	0.00
ENSG00000211666	IGLV2-14	immunoglobulin lambda variable 2-14 [Source:HGNC Symbol;Acc:HGNC:5888]	IG V gene	0.76	7.56	12.17	0.00	0.01
ENSG00000140105	WARS	tryptophanyl-tRNA synthetase [Source:HGNC Symbol;Acc:HGNC:12729]	protein coding	0.76	7.83	20.19	0.00	0.00
ENSG00000184840	TMED9	transmembrane p24 trafficking protein 9 [Source:HGNC Symbol;Acc:HGNC:24878]	protein coding	0.76	6.94	22.68	0.00	0.00
ENSG00000182481	KPNA2	karyopherin subunit alpha 2 [Source:HGNC Symbol;Acc:HGNC:6395]	protein coding	0.76	5.06	27.50	0.00	0.00
ENSG00000211670	IGLV3-9	immunoglobulin lambda variable 3-9 (gene/pseudogene) [Source:HGNC Symbol;Acc:HGNC:5918]	IG V gene	0.76	4.34	11.80	0.00	0.01
ENSG00000112473	SLC39A7	solute carrier family 39 member 7 [Source:HGNC Symbol;Acc:HGNC:4927]	protein coding	0.76	6.40	28.32	0.00	0.00
ENSG00000113387	SUB1	SUB1 homolog, transcriptional regulator [Source:HGNC Symbol;Acc:HGNC:19985]	protein coding	0.76	8.28	23.81	0.00	0.00
ENSG00000120697	ALG5	ALG5, dolichyl-phosphate beta-glucosyltransferase [Source:HGNC Symbol;Acc:HGNC:20266]	protein coding	0.76	4.92	23.37	0.00	0.00
ENSG00000104738	MCM4	minichromosome maintenance complex component 4 [Source:HGNC Symbol;Acc:HGNC:6947]	protein coding	0.75	5.30	20.58	0.00	0.00
ENSG00000169223	LMAN2	lectin, mannose binding 2 [Source:HGNC Symbol;Acc:HGNC:16986]	protein coding	0.75	7.02	21.88	0.00	0.00
ENSG00000124783	SSR1	signal sequence receptor subunit 1 [Source:HGNC Symbol;Acc:HGNC:11323]	protein coding	0.75	8.00	29.92	0.00	0.00

Ensembl Gene ID	Ensembl Gene Name	Ensembl Gene Description	Gene Type	Log_2 Fold Change (Day 6 vs. pre-treatment)	Average Log_2 CPM	Likelihood Ratio Test Statistic	P-Value	FDR Adjusted P-Value
ENSG00000185477	GPRIN3	GPRIN family member 3 [Source:HGNC Symbol;Acc:HGNC:27733]	protein coding	0.75	4.97	14.35	0.00	0.00
ENSG00000239672	NME1	NME/NM23 nucleoside diphosphate kinase 1 [Source:HGNC Symbol;Acc:HGNC:7849]	protein coding	0.74	3.07	16.13	0.00	0.00
ENSG00000144867	SRPRB	SRP receptor beta subunit [Source:HGNC Symbol;Acc:HGNC:24085]	protein coding	0.74	5.75	23.07	0.00	0.00
ENSG00000123473	STIL	SCL/TAL1 interrupting locus [Source:HGNC Symbol;Acc:HGNC:10879]	protein coding	0.74	3.13	9.74	0.00	0.02
ENSG00000232613			antisense	0.74	2.20	7.85	0.01	0.05
ENSG00000146733	PSPH	phosphoserine phosphatase [Source:HGNC Symbol;Acc:HGNC:9577]	protein coding	0.74	2.38	8.77	0.00	0.03
ENSG00000110917	MLEC	malectin [Source:HGNC Symbol;Acc:HGNC:28973]	protein coding	0.74	7.02	28.25	0.00	0.00
ENSG00000100883	SRP54	signal recognition particle 54 [Source:HGNC Symbol;Acc:HGNC:11301]	protein coding	0.73	6.23	26.54	0.00	0.00
ENSG00000134153	EMC7	ER membrane protein complex subunit 7 [Source:HGNC Symbol;Acc:HGNC:24301]	protein coding	0.73	5.11	22.01	0.00	0.00
ENSG00000187922	LCN10	lipocalin 10 [Source:HGNC Symbol;Acc:HGNC:20892]	protein coding	-0.73	2.63	8.13	0.00	0.04
ENSG00000107833	NPM3	nucleophosmin/nucleoplasmin 3 [Source:HGNC Symbol;Acc:HGNC:7931]	protein coding	0.73	2.84	11.07	0.00	0.01
ENSG00000168374	ARF4	ADP ribosylation factor 4 [Source:HGNC Symbol;Acc:HGNC:655]	protein coding	0.73	6.55	30.22	0.00	0.00
ENSG00000111640	GAPDH	glyceraldehyde-3-phosphate dehydrogenase [Source:HGNC Symbol;Acc:HGNC:4141]	protein coding	0.73	8.93	20.76	0.00	0.00
ENSG00000144724	PTPRG	protein tyrosine phosphatase, receptor type G [Source:HGNC Symbol;Acc:HGNC:9671]	protein coding	0.72	2.67	8.08	0.00	0.04
ENSG00000002549	LAP3	leucine aminopeptidase 3 [Source:HGNC Symbol;Acc:HGNC:18449]	protein coding	0.72	5.80	27.19	0.00	0.00
ENSG00000114902	SPCS1	signal peptidase complex subunit 1 [Source:HGNC Symbol;Acc:HGNC:23401]	protein coding	0.72	6.74	32.13	0.00	0.00
ENSG00000198833	UBE2J1	ubiquitin conjugating enzyme E2 J1 [Source:HGNC Symbol;Acc:HGNC:17598]	protein coding	0.72	9.70	16.21	0.00	0.00
ENSG00000102158	MAGT1	magnesium transporter 1 [Source:HGNC Symbol;Acc:HGNC:28880]	protein coding	0.72	6.83	36.24	0.00	0.00
ENSG00000140525	FANCI	Fanconi anemia complementation group I [Source:HGNC Symbol;Acc:HGNC:25568]	protein coding	0.72	4.55	25.27	0.00	0.00
ENSG00000198792	TMEM184B	transmembrane protein 184B [Source:HGNC Symbol;Acc:HGNC:1310]	protein coding	0.72	4.95	10.93	0.00	0.01
ENSG00000007968	E2F2	E2F transcription factor 2 [Source:HGNC Symbol;Acc:HGNC:3114]	protein coding	0.72	4.92	13.78	0.00	0.00
ENSG00000240382	IGKV1-17	immunoglobulin kappa variable 1-17 [Source:HGNC Symbol;Acc:HGNC:5733]	IG V gene	0.71	5.38	12.86	0.00	0.01
ENSG00000138709	LARP1B	La ribonucleoprotein domain family member 1B [Source:HGNC Symbol;Acc:HGNC:24704]	protein coding	0.71	4.79	18.20	0.00	0.00

Ensembl Gene ID	Ensembl Gene Name	Ensembl Gene Description	Gene Type	Log_2 Fold Change (Day 6 vs. pre-treatment)	Average Log_2 CPM	Likelihood Ratio Test Statistic	P-Value	FDR Adjusted P-Value
ENSG00000164305	CASP3	caspase 3 [Source:HGNC Symbol;Acc:HGNC:1504]	protein coding	0.71	6.05	18.84	0.00	0.00
ENSG00000122188	LAX1	lymphocyte transmembrane adaptor 1 [Source:HGNC Symbol;Acc:HGNC:26005]	protein coding	0.71	6.08	14.35	0.00	0.00
ENSG00000012048	BRCA1	BRCA1, DNA repair associated [Source:HGNC Symbol;Acc:HGNC:1100]	protein coding	0.71	4.10	17.93	0.00	0.00
ENSG00000163527	STT3B	STT3B, catalytic subunit of the oligosaccharyltransferase complex [Source:HGNC Symbol;Acc:HGNC:30611]	protein coding	0.71	8.27	19.59	0.00	0.00
ENSG00000213047	DENND1B	DENN domain containing 1B [Source:HGNC Symbol;Acc:HGNC:28404]	protein coding	0.71	5.87	17.65	0.00	0.00
ENSG00000110063	DCPS	decapping enzyme, scavenger [Source:HGNC Symbol;Acc:HGNC:29812]	protein coding	0.71	4.95	12.28	0.00	0.01
ENSG00000119912	IDE	insulin degrading enzyme [Source:HGNC Symbol;Acc:HGNC:5381]	protein coding	0.70	5.83	24.61	0.00	0.00
ENSG00000113811	SELENOK	selenoprotein K [Source:HGNC Symbol;Acc:HGNC:30394]	protein coding	0.70	5.19	24.83	0.00	0.00
ENSG00000166851	PLK1	polo like kinase 1 [Source:HGNC Symbol;Acc:HGNC:9077]	protein coding	0.70	3.47	9.07	0.00	0.03
ENSG00000118363	SPCS2	signal peptidase complex subunit 2 [Source:HGNC Symbol;Acc:HGNC:28962]	protein coding	0.70	5.87	18.38	0.00	0.00
ENSG00000230006	ANKRD36BP2	ankyrin repeat domain 36B pseudogene 2 [Source:HGNC Symbol;Acc:HGNC:33607]	transcribed unprocessed pseudogene	0.70	5.15	9.03	0.00	0.03
ENSG00000037241	RPL26L1	ribosomal protein L26 like 1 [Source:HGNC Symbol;Acc:HGNC:17050]	protein coding	0.70	2.75	11.66	0.00	0.01
ENSG00000184432	COPB2	coatomer protein complex subunit beta 2 [Source:HGNC Symbol;Acc:HGNC:2232]	protein coding	0.70	7.32	26.70	0.00	0.00
ENSG00000127022	CANX	calnexin [Source:HGNC Symbol;Acc:HGNC:1473]	protein coding	0.69	9.13	24.72	0.00	0.00
ENSG00000095380	NANS	N-acetylneuraminate synthase [Source:HGNC Symbol;Acc:HGNC:19237]	protein coding	0.69	4.46	12.90	0.00	0.01
ENSG00000067167	TRAM1	translocation associated membrane protein 1 [Source:HGNC Symbol;Acc:HGNC:20568]	protein coding	0.69	8.46	19.79	0.00	0.00
ENSG00000086598	TMED2	transmembrane p24 trafficking protein 2 [Source:HGNC Symbol;Acc:HGNC:16996]	protein coding	0.69	7.60	24.17	0.00	0.00
ENSG00000129128	SPCS3	signal peptidase complex subunit 3 [Source:HGNC Symbol;Acc:HGNC:26212]	protein coding	0.69	8.25	19.91	0.00	0.00
ENSG00000198380	GFPT1	glutamine–fructose-6-phosphate transaminase 1 [Source:HGNC Symbol;Acc:HGNC:4241]	protein coding	0.69	5.97	20.34	0.00	0.00
ENSG00000049656	CLPTM1L	CLPTM1 like [Source:HGNC Symbol;Acc:HGNC:24308]	protein coding	0.69	7.62	16.21	0.00	0.00
ENSG00000211950	IGHV1-24	immunoglobulin heavy variable 1-24 [Source:HGNC Symbol;Acc:HGNC:5551]	IG V gene	0.68	4.87	21.22	0.00	0.00

Ensembl Gene ID	Ensembl Gene Name	Ensembl Gene Description	Gene Type	Log_2 Fold Change (Day 6 vs. pre-treatment)	Average Log_2 CPM	Likelihood Ratio Test Statistic	P-Value	FDR Adjusted P-Value
ENSG00000113621	TXNDC15	thioredoxin domain containing 15 [Source:HGNC Symbol;Acc:HGNC:20652]	protein coding	0.68	5.55	21.24	0.00	0.00
ENSG00000264522	OTUD7B	OTU deubiquitinase 7B [Source:HGNC Symbol;Acc:HGNC:16683]	protein coding	0.68	2.87	9.29	0.00	0.03
ENSG00000086062	B4GALT1	beta-1,4-galactosyltransferase 1 [Source:HGNC Symbol;Acc:HGNC:924]	protein coding	0.68	8.59	21.69	0.00	0.00
ENSG00000180879	SSR4	signal sequence receptor subunit 4 [Source:HGNC Symbol;Acc:HGNC:11326]	protein coding	0.68	7.44	15.91	0.00	0.00
ENSG00000211962	IGHV1-46	immunoglobulin heavy variable 1-46 [Source:HGNC Symbol;Acc:HGNC:5554]	IG V gene	0.67	6.18	8.36	0.00	0.04
ENSG00000040933	INPP4A	inositol polyphosphate-4-phosphatase type I A [Source:HGNC Symbol;Acc:HGNC:6074]	protein coding	0.67	6.54	15.19	0.00	0.00
ENSG00000211945	IGHV1-18	immunoglobulin heavy variable 1-18 [Source:HGNC Symbol;Acc:HGNC:5549]	IG V gene	0.67	6.51	16.04	0.00	0.00
ENSG00000137812	KNL1	kinetochore scaffold 1 [Source:HGNC Symbol;Acc:HGNC:24054]	protein coding	0.67	4.33	15.75	0.00	0.00
ENSG00000259772			lincRNA	0.67	4.06	9.98	0.00	0.02
ENSG00000085063	CD59	CD59 molecule [Source:HGNC Symbol;Acc:HGNC:1689]	protein coding	0.67	5.70	13.49	0.00	0.00
ENSG00000211649	IGLV7-46	immunoglobulin lambda variable 7-46 (gene/pseudogene) [Source:HGNC Symbol;Acc:HGNC:5930]	IG V gene	0.67	5.23	17.47	0.00	0.00
ENSG00000129636	ITFG1	integrin alpha FG-GAP repeat containing 1 [Source:HGNC Symbol;Acc:HGNC:30697]	protein coding	0.66	4.97	22.10	0.00	0.00
ENSG00000076003	MCM6	minichromosome maintenance complex component 6 [Source:HGNC Symbol;Acc:HGNC:6949]	protein coding	0.66	5.13	14.62	0.00	0.00
ENSG00000170348	TMED10	transmembrane p24 trafficking protein 10 [Source:HGNC Symbol;Acc:HGNC:16998]	protein coding	0.66	8.09	23.53	0.00	0.00
ENSG00000147649	MTDH	metadherin [Source:HGNC Symbol;Acc:HGNC:29608]	protein coding	0.66	8.52	27.32	0.00	0.00
ENSG00000242534	IGKV2D-28	immunoglobulin kappa variable 2D-28 [Source:HGNC Symbol;Acc:HGNC:5799]	IG V gene	0.66	4.07	11.57	0.00	0.01
ENSG00000196839	ADA	adenosine deaminase [Source:HGNC Symbol;Acc:HGNC:186]	protein coding	0.66	4.12	8.12	0.00	0.04
ENSG00000185745	IFIT1	interferon induced protein with tetratricopeptide repeats 1 [Source:HGNC Symbol;Acc:HGNC:5407]	protein coding	0.66	3.31	11.15	0.00	0.01
ENSG00000211967	IGHV3-53	immunoglobulin heavy variable 3-53 [Source:HGNC Symbol;Acc:HGNC:5610]	IG V gene	0.66	5.84	19.12	0.00	0.00
ENSG00000101310	SEC23B	Sec23 homolog B, coat complex II component [Source:HGNC Symbol;Acc:HGNC:10702]	protein coding	0.65	6.45	21.60	0.00	0.00
ENSG00000211668	IGLV2-11	immunoglobulin lambda variable 2-11 [Source:HGNC Symbol;Acc:HGNC:5887]	IG V gene	0.65	7.19	11.28	0.00	0.01
ENSG00000154511	FAM69A	family with sequence similarity 69 member A [Source:HGNC Symbol;Acc:HGNC:32213]	protein coding	0.65	5.29	20.44	0.00	0.00
ENSG00000231475	IGHV4-31	immunoglobulin heavy variable 4-31 [Source:HGNC Symbol;Acc:HGNC:5649]	IG V gene	0.65	4.75	18.89	0.00	0.00

Ensembl Gene ID	Ensembl Gene Name	Ensembl Gene Description	Gene Type	Log_2 Fold Change (Day 6 vs. pre-treatment)	Average Log_2 CPM	Likelihood Ratio Test Statistic	P-Value	FDR Adjusted P-Value
ENSG00000138760	SCARB2	scavenger receptor class B member 2 [Source:HGNC Symbol;Acc:HGNC:1665]	protein coding	0.65	5.92	16.50	0.00	0.00
ENSG00000142731	PLK4	polo like kinase 4 [Source:HGNC Symbol;Acc:HGNC:11397]	protein coding	0.65	2.96	9.38	0.00	0.03
ENSG00000154723	ATP5J	ATP synthase, H+ transporting, mitochondrial Fo complex subunit F6 [Source:HGNC Symbol;Acc:HGNC:847]	protein coding	0.65	5.19	22.87	0.00	0.00
ENSG00000279278			lincRNA	-0.65	3.58	11.79	0.00	0.01
ENSG00000165672	PRDX3	peroxiredoxin 3 [Source:HGNC Symbol;Acc:HGNC:9354]	protein coding	0.65	6.01	26.83	0.00	0.00
ENSG00000220008	LINGO3	leucine rich repeat and Ig domain containing 3 [Source:HGNC Symbol;Acc:HGNC:21206]	protein coding	-0.64	4.59	20.06	0.00	0.00
ENSG00000167325	RRM1	ribonucleotide reductase catalytic subunit M1 [Source:HGNC Symbol;Acc:HGNC:10451]	protein coding	0.64	5.23	22.88	0.00	0.00
ENSG00000108953	YWHAE	tyrosine 3-monooxygenase/tryptophan 5-monooxygenase activation protein epsilon [Source:HGNC Symbol;Acc:HGNC:12851]	protein coding	0.64	6.77	24.40	0.00	0.00
ENSG00000112893	MAN2A1	mannosidase alpha class 2A member 1 [Source:HGNC Symbol;Acc:HGNC:6824]	protein coding	0.64	7.49	23.15	0.00	0.00
ENSG00000182197	EXT1	exostosin glycosyltransferase 1 [Source:HGNC Symbol;Acc:HGNC:3512]	protein coding	0.64	3.13	10.87	0.00	0.01
ENSG00000087502	ERGIC2	ERGIC and golgi 2 [Source:HGNC Symbol;Acc:HGNC:30208]	protein coding	0.64	5.05	16.41	0.00	0.00
ENSG00000138768	USO1	USO1 vesicle transport factor [Source:HGNC Symbol;Acc:HGNC:30904]	protein coding	0.64	7.19	21.66	0.00	0.00
ENSG00000235587	GAPDHP65	glyceraldehyde 3 phosphate dehydrogenase pseudogene 65 [Source:HGNC Symbol;Acc:HGNC:4143]	processed pseudogene	0.64	2.83	7.83	0.01	0.05
ENSG00000105438	KDELR1	KDEL endoplasmic reticulum protein retention receptor 1 [Source:HGNC Symbol;Acc:HGNC:6304]	protein coding	0.64	6.02	17.55	0.00	0.00
ENSG00000129562	DAD1	defender against cell death 1 [Source:HGNC Symbol;Acc:HGNC:2664]	protein coding	0.64	6.22	25.90	0.00	0.00
ENSG00000197780	TAF13	TATA-box binding protein associated factor 13 [Source:HGNC Symbol;Acc:HGNC:11546]	protein coding	0.63	3.11	11.38	0.00	0.01
ENSG00000116649	SRM	spermidine synthase [Source:HGNC Symbol;Acc:HGNC:11296]	protein coding	0.63	5.90	18.87	0.00	0.00
ENSG00000069956	MAPK6	mitogen-activated protein kinase 6 [Source:HGNC Symbol;Acc:HGNC:6879]	protein coding	0.63	5.19	25.57	0.00	0.00
ENSG00000187837	HIST1H1C	histone cluster 1 H1 family member c [Source:HGNC Symbol;Acc:HGNC:4716]	protein coding	0.63	3.70	10.75	0.00	0.02
ENSG00000182934	SRPRA	SRP receptor alpha subunit [Source:HGNC Symbol;Acc:HGNC:11307]	protein coding	0.63	8.06	18.47	0.00	0.00
ENSG00000080546	SESN1	sestrin 1 [Source:HGNC Symbol;Acc:HGNC:21595]	protein coding	-0.63	6.64	41.99	0.00	0.00

Ensembl Gene ID	Ensembl Gene Name	Ensembl Gene Description	Gene Type	Log_2 Fold Change (Day 6 vs. pre-treatment)	Average Log_2 CPM	Likelihood Ratio Test Statistic	P-Value	FDR Adjusted P-Value
ENSG00000228903	RASA4CP	RAS p21 protein activator 4C, pseudogene [Source:HGNC Symbol;Acc:HGNC:44185]	transcribed unprocessed pseudogene	-0.63	3.34	11.21	0.00	0.01
ENSG00000106080	FKBP14	FK506 binding protein 14 [Source:HGNC Symbol;Acc:HGNC:18625]	protein coding	0.62	3.83	7.88	0.01	0.05
ENSG00000138073	PREB	prolactin regulatory element binding [Source:HGNC Symbol;Acc:HGNC:9356]	protein coding	0.62	6.58	16.13	0.00	0.00
ENSG00000104635	SLC39A14	solute carrier family 39 member 14 [Source:HGNC Symbol;Acc:HGNC:20858]	protein coding	0.62	4.46	14.83	0.00	0.00
ENSG00000172339	ALG14	ALG14, UDP-N-acetylglucosaminyltransferase subunit [Source:HGNC Symbol;Acc:HGNC:28287]	protein coding	0.62	3.14	9.69	0.00	0.02
ENSG00000143641	GALNT2	polypeptide N-acetylgalactosaminyltransferase 2 [Source:HGNC Symbol;Acc:HGNC:4124]	protein coding	0.62	6.04	23.63	0.00	0.00
ENSG00000228327			transcribed unprocessed pseudogene	-0.62	2.58	7.84	0.01	0.05
ENSG00000141753	IGFBP4	insulin like growth factor binding protein 4 [Source:HGNC Symbol;Acc:HGNC:5473]	protein coding	-0.62	3.41	11.36	0.00	0.01
ENSG00000104290	FZD3	frizzled class receptor 3 [Source:HGNC Symbol;Acc:HGNC:4041]	protein coding	0.62	3.15	9.43	0.00	0.03
ENSG00000170027	YWHAG	tyrosine 3-monooxygenase/tryptophan 5-monooxygenase activation protein gamma [Source:HGNC Symbol;Acc:HGNC:12852]	protein coding	0.62	6.80	15.53	0.00	0.00
ENSG00000069849	ATP1B3	ATPase Na+/K+ transporting subunit beta 3 [Source:HGNC Symbol;Acc:HGNC:806]	protein coding	0.62	5.52	21.64	0.00	0.00
ENSG00000145354	CISD2	CDGSH iron sulfur domain 2 [Source:HGNC Symbol;Acc:HGNC:24212]	protein coding	0.61	3.53	9.31	0.00	0.03
ENSG00000140743	CDR2	cerebellar degeneration related protein 2 [Source:HGNC Symbol;Acc:HGNC:1799]	protein coding	0.61	4.39	9.20	0.00	0.03
ENSG00000255026			antisense	-0.61	2.72	8.91	0.00	0.03
ENSG00000157020	SEC13	SEC13 homolog, nuclear pore and COPII coat complex component [Source:HGNC Symbol;Acc:HGNC:10697]	protein coding	0.61	6.35	17.17	0.00	0.00
ENSG00000198130	HIBCH	3-hydroxyisobutyryl-CoA hydrolase [Source:HGNC Symbol;Acc:HGNC:4908]	protein coding	0.61	5.20	22.01	0.00	0.00
ENSG00000113273	ARSB	arylsulfatase B [Source:HGNC Symbol;Acc:HGNC:714]	protein coding	0.61	4.25	18.58	0.00	0.00
ENSG00000163754	GYG1	glycogenin 1 [Source:HGNC Symbol;Acc:HGNC:4699]	protein coding	0.61	5.15	18.95	0.00	0.00
ENSG00000108578	BLMH	bleomycin hydrolase [Source:HGNC Symbol;Acc:HGNC:1059]	protein coding	0.61	4.98	22.91	0.00	0.00

Ensembl Gene ID	Ensembl Gene Name	Ensembl Gene Description	Gene Type	Log_2 Fold Change (Day 6 vs. pre-treatment)	Average Log_2 CPM	Likelihood Ratio Test Statistic	P-Value	FDR Adjusted P-Value
ENSG00000146376	ARHGAP18	Rho GTPase activating protein 18 [Source:HGNC Symbol;Acc:HGNC:21035]	protein coding	0.61	3.96	16.81	0.00	0.00
ENSG00000172115	CYCS	cytochrome c, somatic [Source:HGNC Symbol;Acc:HGNC:19986]	protein coding	0.61	6.05	31.13	0.00	0.00
ENSG00000167004	PDIA3	protein disulfide isomerase family A member 3 [Source:HGNC Symbol;Acc:HGNC:4606]	protein coding	0.61	7.92	27.04	0.00	0.00
ENSG00000154719	MRPL39	mitochondrial ribosomal protein L39 [Source:HGNC Symbol;Acc:HGNC:14027]	protein coding	0.61	4.21	21.36	0.00	0.00
ENSG00000106105	GARS	glycyl-tRNA synthetase [Source:HGNC Symbol;Acc:HGNC:4162]	protein coding	0.61	6.50	26.88	0.00	0.00
ENSG00000185522	LMNTD2	lamin tail domain containing 2 [Source:HGNC Symbol;Acc:HGNC:28561]	protein coding	-0.61	3.09	11.91	0.00	0.01
ENSG00000255733	IFNG-AS1	IFNG antisense RNA 1 [Source:HGNC Symbol;Acc:HGNC:43910]	antisense	0.60	4.49	12.08	0.00	0.01
ENSG00000100804	PSMB5	proteasome subunit beta 5 [Source:HGNC Symbol;Acc:HGNC:9542]	protein coding	0.60	4.11	13.08	0.00	0.01
ENSG00000145817	YIPF5	Yip1 domain family member 5 [Source:HGNC Symbol;Acc:HGNC:24877]	protein coding	0.60	5.19	28.67	0.00	0.00
ENSG00000112237	CCNC	cyclin C [Source:HGNC Symbol;Acc:HGNC:1581]	protein coding	0.60	6.31	18.68	0.00	0.00
ENSG00000119523	ALG2	ALG2, alpha-1,3/1,6-mannosyltransferase [Source:HGNC Symbol;Acc:HGNC:23159]	protein coding	0.60	5.10	15.47	0.00	0.00
ENSG00000106462	EZH2	enhancer of zeste 2 polycomb repressive complex 2 subunit [Source:HGNC Symbol;Acc:HGNC:3527]	protein coding	0.60	4.68	13.18	0.00	0.01
ENSG00000179222	MAGED1	MAGE family member D1 [Source:HGNC Symbol;Acc:HGNC:6813]	protein coding	0.60	6.26	16.37	0.00	0.00
ENSG00000108984	MAP2K6	mitogen-activated protein kinase kinase 6 [Source:HGNC Symbol;Acc:HGNC:6846]	protein coding	0.60	3.53	11.46	0.00	0.01
ENSG00000168653	NDUFS5	NADH:ubiquinone oxidoreductase subunit S5 [Source:HGNC Symbol;Acc:HGNC:7712]	protein coding	0.60	5.12	17.41	0.00	0.00
ENSG00000145703	IQGAP2	IQ motif containing GTPase activating protein 2 [Source:HGNC Symbol;Acc:HGNC:6111]	protein coding	0.60	6.35	21.25	0.00	0.00
ENSG00000117632	STMN1	stathmin 1 [Source:HGNC Symbol;Acc:HGNC:6510]	protein coding	0.59	5.74	23.69	0.00	0.00
ENSG00000280063			TEC	-0.59	3.21	9.21	0.00	0.03
ENSG00000274272			processed transcript	-0.59	3.99	15.02	0.00	0.00
ENSG00000182022	CHST15	carbohydrate sulfotransferase 15 [Source:HGNC Symbol;Acc:HGNC:18137]	protein coding	0.59	6.55	15.40	0.00	0.00
ENSG00000137806	NDUFAF1	NADH:ubiquinone oxidoreductase complex assembly factor 1 [Source:HGNC Symbol;Acc:HGNC:18828]	protein coding	0.59	4.03	13.32	0.00	0.01
ENSG00000198900	TOP1	topoisomerase (DNA) I [Source:HGNC Symbol;Acc:HGNC:11986]	protein coding	0.59	6.87	29.64	0.00	0.00
ENSG00000163507	KIAA1524	KIAA1524 [Source:HGNC Symbol;Acc:HGNC:29302]	protein coding	0.59	3.70	14.06	0.00	0.00

Ensembl Gene ID	Ensembl Gene Name	Ensembl Gene Description	Gene Type	Log_2 Fold Change (Day 6 vs. pre-treatment)	Average Log_2 CPM	Likelihood Ratio Test Statistic	P-Value	FDR Adjusted P-Value
ENSG00000198879	SFMBT2	Scm-like with four mbt domains 2 [Source:HGNC Symbol;Acc:HGNC:20256]	protein coding	0.59	5.00	14.75	0.00	0.00
ENSG00000166548	TK2	thymidine kinase 2, mitochondrial [Source:HGNC Symbol;Acc:HGNC:11831]	protein coding	0.59	3.09	8.10	0.00	0.04
ENSG00000165895	ARHGAP42	Rho GTPase activating protein 42 [Source:HGNC Symbol;Acc:HGNC:26545]	protein coding	0.59	3.93	10.66	0.00	0.02
ENSG00000049860	HEXB	hexosaminidase subunit beta [Source:HGNC Symbol;Acc:HGNC:4879]	protein coding	0.59	5.51	13.49	0.00	0.00
ENSG00000108826	MRPL27	mitochondrial ribosomal protein L27 [Source:HGNC Symbol;Acc:HGNC:14483]	protein coding	0.59	3.93	13.89	0.00	0.00
ENSG00000197157	SND1	staphylococcal nuclease and tudor domain containing 1 [Source:HGNC Symbol;Acc:HGNC:30646]	protein coding	0.59	7.99	18.86	0.00	0.00
ENSG00000088298	EDEM2	ER degradation enhancing alpha-mannosidase like protein 2 [Source:HGNC Symbol;Acc:HGNC:15877]	protein coding	0.58	5.88	16.64	0.00	0.00

Table 12: Genes differentially expressed at Day 6 compared to pre-treatment (B Cells, Trivalent Influenza Vaccine). Sorted by descending absolute log_2 fold change (Day 6 vs. pre-treatment). Gene model summaries and annotations are based on Ensembl Version 87.

Ensembl Gene ID	Ensembl Gene Name	Ensembl Gene Description	Gene Type	Log_2 Fold Change (Day 7 vs. pre-treatment)	Average Log_2 CPM	Likelihood Ratio Test Statistic	P-Value	FDR Adjusted P-Value
ENSG00000259997	IGHV1OR16-4	immunoglobulin heavy variable 1/OR16-4 (pseudogene) [Source:HGNC Symbol;Acc:HGNC:5573]	IG V pseudogene	3.52	0.83	20.05	0.00	0.00
ENSG00000254174	IGHV1-12	immunoglobulin heavy variable 1-12 (pseudogene) [Source:HGNC Symbol;Acc:HGNC:5546]	IG V pseudogene	2.56	1.00	25.25	0.00	0.00
ENSG00000148468	FAM171A1	family with sequence similarity 171 member A1 [Source:HGNC Symbol;Acc:HGNC:23522]	protein coding	2.35	0.71	22.10	0.00	0.00
ENSG00000261834	IGHV3OR16-15	immunoglobulin heavy variable 3/OR16-15 (pseudogene) [Source:HGNC Symbol;Acc:HGNC:5639]	IG V pseudogene	2.14	1.20	10.89	0.00	0.02
ENSG00000088340	FER1L4	fer-1 like family member 4, pseudogene [Source:HGNC Symbol;Acc:HGNC:15801]	transcribed unitary pseudogene	2.07	2.48	55.27	0.00	0.00
ENSG00000154277	UCHL1	ubiquitin C-terminal hydrolase L1 [Source:HGNC Symbol;Acc:HGNC:12513]	protein coding	2.04	1.68	29.62	0.00	0.00
ENSG00000153093	ACOXL	acyl-CoA oxidase-like [Source:HGNC Symbol;Acc:HGNC:25621]	protein coding	1.99	1.07	15.82	0.00	0.00
ENSG00000211896	IGHG1	immunoglobulin heavy constant gamma 1 (G1m marker) [Source:HGNC Symbol;Acc:HGNC:5525]	IG C gene	1.94	12.36	69.68	0.00	0.00
ENSG00000113140	SPARC	secreted protein acidic and cysteine rich [Source:HGNC Symbol;Acc:HGNC:11219]	protein coding	1.94	1.63	16.67	0.00	0.00
ENSG00000211662	IGLV3-21	immunoglobulin lambda variable 3-21 [Source:HGNC Symbol;Acc:HGNC:5905]	IG V gene	1.93	9.48	17.32	0.00	0.00

Ensembl Gene ID	Ensembl Gene Name	Ensembl Gene Description	Gene Type	Log_2 Fold Change (Day 7 vs. pre-treatment)	Average Log_2 CPM	Likelihood Ratio Test Statistic	P-Value	FDR Adjusted P-Value
ENSG00000111186	WNT5B	Wnt family member 5B [Source:HGNC Symbol;Acc:HGNC:16265]	protein coding	1.93	0.93	19.60	0.00	0.00
ENSG00000115884	SDC1	syndecan 1 [Source:HGNC Symbol;Acc:HGNC:10658]	protein coding	1.92	4.23	97.96	0.00	0.00
ENSG00000211938	IGHV3-7	immunoglobulin heavy variable 3-7 [Source:HGNC Symbol;Acc:HGNC:5620]	IG V gene	1.92	8.78	34.31	0.00	0.00
ENSG00000211653	IGLV1-40	immunoglobulin lambda variable 1-40 [Source:HGNC Symbol;Acc:HGNC:5877]	IG V gene	1.84	8.79	19.26	0.00	0.00
ENSG00000253755	IGHGP	immunoglobulin heavy constant gamma P (non-functional) [Source:HGNC Symbol;Acc:HGNC:5529]	IG C pseudogene	1.75	7.32	56.38	0.00	0.00
ENSG00000105974	CAV1	caveolin 1 [Source:HGNC Symbol;Acc:HGNC:1527]	protein coding	1.74	4.02	54.58	0.00	0.00
ENSG00000168078	PBK	PDZ binding kinase [Source:HGNC Symbol;Acc:HGNC:18282]	protein coding	1.51	1.22	13.15	0.00	0.01
ENSG00000211651	IGLV1-44	immunoglobulin lambda variable 1-44 [Source:HGNC Symbol;Acc:HGNC:5879]	IG V gene	1.47	6.67	17.03	0.00	0.00
ENSG00000197476			processed pseudogene	1.46	3.59	21.58	0.00	0.00
ENSG00000162676	GFI1	growth factor independent 1 transcriptional repressor [Source:HGNC Symbol;Acc:HGNC:4237]	protein coding	1.45	2.61	20.43	0.00	0.00
ENSG00000211670	IGLV3-9	immunoglobulin lambda variable 3-9 (gene/pseudogene) [Source:HGNC Symbol;Acc:HGNC:5918]	IG V gene	1.39	5.54	12.05	0.00	0.01
ENSG00000211677	IGLC2	immunoglobulin lambda constant 2 [Source:HGNC Symbol;Acc:HGNC:5856]	IG C gene	1.39	10.76	22.22	0.00	0.00
ENSG00000248571			antisense	1.36	1.97	25.72	0.00	0.00
ENSG00000123989	CHPF	chondroitin polymerizing factor [Source:HGNC Symbol;Acc:HGNC:24291]	protein coding	1.36	5.15	32.86	0.00	0.00
ENSG00000203914	HSP90B3P	heat shock protein 90 beta family member 3, pseudogene [Source:HGNC Symbol;Acc:HGNC:12100]	processed pseudogene	1.33	3.00	41.97	0.00	0.00
ENSG00000163053	SLC16A14	solute carrier family 16 member 14 [Source:HGNC Symbol;Acc:HGNC:26417]	protein coding	1.33	2.31	18.99	0.00	0.00
ENSG00000138166	DUSP5	dual specificity phosphatase 5 [Source:HGNC Symbol;Acc:HGNC:3071]	protein coding	1.32	4.78	40.42	0.00	0.00
ENSG00000184661	CDCA2	cell division cycle associated 2 [Source:HGNC Symbol;Acc:HGNC:14623]	protein coding	1.32	1.98	18.69	0.00	0.00
ENSG00000165304	MELK	maternal embryonic leucine zipper kinase [Source:HGNC Symbol;Acc:HGNC:16870]	protein coding	1.31	1.79	16.07	0.00	0.00
ENSG00000211673	IGLV3-1	immunoglobulin lambda variable 3-1 [Source:HGNC Symbol;Acc:HGNC:5896]	IG V gene	1.31	8.34	28.10	0.00	0.00
ENSG00000074842	MYDGF	myeloid derived growth factor [Source:HGNC Symbol;Acc:HGNC:16948]	protein coding	1.31	6.70	51.97	0.00	0.00
ENSG00000121807	CCR2	C-C motif chemokine receptor 2 [Source:HGNC Symbol;Acc:HGNC:1603]	protein coding	1.28	5.75	113.13	0.00	0.00

Ensembl Gene ID	Ensembl Gene Name	Ensembl Gene Description	Gene Type	Log_2 Fold Change (Day 7 vs. pre-treatment)	Average Log_2 CPM	Likelihood Ratio Test Statistic	P-Value	FDR Adjusted P-Value
ENSG00000224373	IGHV4-59	immunoglobulin heavy variable 4-59 [Source:HGNC Symbol;Acc:HGNC:5654]	IG V gene	1.26	6.63	110.60	0.00	0.00
ENSG00000136010	ALDH1L2	aldehyde dehydrogenase 1 family member L2 [Source:HGNC Symbol;Acc:HGNC:26777]	protein coding	1.26	3.98	10.09	0.00	0.03
ENSG00000178445	GLDC	glycine decarboxylase [Source:HGNC Symbol;Acc:HGNC:4313]	protein coding	1.25	5.15	45.43	0.00	0.00
ENSG00000100219	XBP1	X-box binding protein 1 [Source:HGNC Symbol;Acc:HGNC:12801]	protein coding	1.25	8.51	57.90	0.00	0.00
ENSG00000198722	UNC13B	unc-13 homolog B [Source:HGNC Symbol;Acc:HGNC:12566]	protein coding	1.25	2.40	17.08	0.00	0.00
ENSG00000270472	IGHV3OR16-9	immunoglobulin heavy variable 3/OR16-9 (non-functional) [Source:HGNC Symbol;Acc:HGNC:5644]	IG V gene	1.25	4.07	9.28	0.00	0.04
ENSG00000088826	SMOX	spermine oxidase [Source:HGNC Symbol;Acc:HGNC:15862]	protein coding	1.24	1.84	15.89	0.00	0.00
ENSG00000239975	IGKV1D-33	immunoglobulin kappa variable 1D-33 [Source:HGNC Symbol;Acc:HGNC:5753]	IG V gene	1.24	4.11	14.05	0.00	0.01
ENSG00000075420	FNDC3B	fibronectin type III domain containing 3B [Source:HGNC Symbol;Acc:HGNC:24670]	protein coding	1.22	5.48	37.77	0.00	0.00
ENSG00000167476	JSRP1	junctional sarcoplasmic reticulum protein 1 [Source:HGNC Symbol;Acc:HGNC:24963]	protein coding	1.21	2.90	22.10	0.00	0.00
ENSG00000183010	PYCR1	pyrroline-5-carboxylate reductase 1 [Source:HGNC Symbol;Acc:HGNC:9721]	protein coding	1.19	2.81	26.71	0.00	0.00
ENSG00000123485	HJURP	Holliday junction recognition protein [Source:HGNC Symbol;Acc:HGNC:25444]	protein coding	1.18	2.40	11.64	0.00	0.01
ENSG00000166598	HSP90B1	heat shock protein 90 beta family member 1 [Source:HGNC Symbol;Acc:HGNC:12028]	protein coding	1.18	10.71	49.00	0.00	0.00
ENSG00000090104	RGS1	regulator of G-protein signaling 1 [Source:HGNC Symbol;Acc:HGNC:9991]	protein coding	1.18	3.08	9.65	0.00	0.03
ENSG00000165409	TSHR	thyroid stimulating hormone receptor [Source:HGNC Symbol;Acc:HGNC:12373]	protein coding	1.17	2.52	16.42	0.00	0.00
ENSG00000216775			transcribed unprocessed pseudogene	1.16	1.53	10.83	0.00	0.02
ENSG00000239571	IGKV2D-30	immunoglobulin kappa variable 2D-30 [Source:HGNC Symbol;Acc:HGNC:5801]	IG V gene	1.15	3.68	11.53	0.00	0.02
ENSG00000186810	CXCR3	C-X-C motif chemokine receptor 3 [Source:HGNC Symbol;Acc:HGNC:4540]	protein coding	1.15	4.13	33.67	0.00	0.00
ENSG00000065485	PDIA5	protein disulfide isomerase family A member 5 [Source:HGNC Symbol;Acc:HGNC:24811]	protein coding	1.15	4.42	28.61	0.00	0.00
ENSG00000211973	IGHV1-69	immunoglobulin heavy variable 1-69 [Source:HGNC Symbol;Acc:HGNC:5558]	IG V gene	1.14	4.04	30.14	0.00	0.00
ENSG00000164045	CDC25A	cell division cycle 25A [Source:HGNC Symbol;Acc:HGNC:1725]	protein coding	1.14	1.85	10.27	0.00	0.03

Ensembl Gene ID	Ensembl Gene Name	Ensembl Gene Description	Gene Type	Log_2 Fold Change (Day 7 vs. pre-treatment)	Average Log_2 CPM	Likelihood Ratio Test Statistic	P-Value	FDR Adjusted P-Value
ENSG00000155660	PDIA4	protein disulfide isomerase family A member 4 [Source:HGNC Symbol;Acc:HGNC:30167]	protein coding	1.14	8.74	38.08	0.00	0.00
ENSG00000211648	IGLV1-47	immunoglobulin lambda variable 1-47 [Source:HGNC Symbol;Acc:HGNC:5880]	IG V gene	1.13	7.13	9.04	0.00	0.04
ENSG00000259706	HSP90B2P	heat shock protein 90 beta family member 2, pseudogene [Source:HGNC Symbol;Acc:HGNC:12099]	processed pseudogene	1.13	5.08	44.41	0.00	0.00
ENSG00000186594	MIR22HG	MIR22 host gene [Source:HGNC Symbol;Acc:HGNC:28219]	lincRNA	1.12	3.18	34.69	0.00	0.00
ENSG00000107104	KANK1	KN motif and ankyrin repeat domains 1 [Source:HGNC Symbol;Acc:HGNC:19309]	protein coding	1.12	3.11	27.33	0.00	0.00
ENSG00000280411	IGHV1-69-2	immunoglobulin heavy variable 1-69-2 [Source:HGNC Symbol;Acc:HGNC:5562]	IG V gene	1.10	5.46	18.73	0.00	0.00
ENSG00000137563	GGH	gamma-glutamyl hydrolase [Source:HGNC Symbol;Acc:HGNC:4248]	protein coding	1.10	3.75	30.15	0.00	0.00
ENSG00000026751	SLAMF7	SLAM family member 7 [Source:HGNC Symbol;Acc:HGNC:21394]	protein coding	1.09	7.60	38.74	0.00	0.00
ENSG00000128228	SDF2L1	stromal cell derived factor 2 like 1 [Source:HGNC Symbol;Acc:HGNC:10676]	protein coding	1.09	5.15	33.53	0.00	0.00
ENSG00000118985	ELL2	elongation factor for RNA polymerase II 2 [Source:HGNC Symbol;Acc:HGNC:17064]	protein coding	1.09	7.22	51.62	0.00	0.00
ENSG00000025039	RRAGD	Ras related GTP binding D [Source:HGNC Symbol;Acc:HGNC:19903]	protein coding	1.08	2.92	25.65	0.00	0.00
ENSG00000143333	RGS16	regulator of G-protein signaling 16 [Source:HGNC Symbol;Acc:HGNC:9997]	protein coding	1.08	2.27	13.74	0.00	0.01
ENSG00000171444	MCC	mutated in colorectal cancers [Source:HGNC Symbol;Acc:HGNC:6935]	protein coding	1.08	2.21	13.94	0.00	0.01
ENSG00000004468	CD38	CD38 molecule [Source:HGNC Symbol;Acc:HGNC:1667]	protein coding	1.08	7.71	54.90	0.00	0.00
ENSG00000118515	SGK1	serum/glucocorticoid regulated kinase 1 [Source:HGNC Symbol;Acc:HGNC:10810]	protein coding	1.08	4.48	75.17	0.00	0.00
ENSG00000135916	ITM2C	integral membrane protein 2C [Source:HGNC Symbol;Acc:HGNC:6175]	protein coding	1.07	8.80	38.84	0.00	0.00
ENSG00000180535	BHLHA15	basic helix-loop-helix family member a15 [Source:HGNC Symbol;Acc:HGNC:22265]	protein coding	1.07	3.45	19.57	0.00	0.00
ENSG00000171848	RRM2	ribonucleotide reductase regulatory subunit M2 [Source:HGNC Symbol;Acc:HGNC:10452]	protein coding	1.07	5.35	26.67	0.00	0.00
ENSG00000101439	CST3	cystatin C [Source:HGNC Symbol;Acc:HGNC:2475]	protein coding	-1.06	3.42	23.82	0.00	0.00
ENSG00000239264	TXNDC5	thioredoxin domain containing 5 [Source:HGNC Symbol;Acc:HGNC:21073]	protein coding	1.06	6.02	21.42	0.00	0.00
ENSG00000242766	IGKV1D-17	immunoglobulin kappa variable 1D-17 [Source:HGNC Symbol;Acc:HGNC:5749]	IG V gene	1.05	3.30	25.34	0.00	0.00
ENSG00000176890	TYMS	thymidylate synthetase [Source:HGNC Symbol;Acc:HGNC:12441]	protein coding	1.05	3.07	19.75	0.00	0.00

Ensembl Gene ID	Ensembl Gene Name	Ensembl Gene Description	Gene Type	Log_2 Fold Change (Day 7 vs. pre-treatment)	Average Log_2 CPM	Likelihood Ratio Test Statistic	P-Value	FDR Adjusted P-Value
ENSG00000184232	OAF	out at first homolog [Source:HGNC Symbol;Acc:HGNC:28752]	protein coding	1.04	2.50	12.83	0.00	0.01
ENSG00000048462	TNFRSF17	TNF receptor superfamily member 17 [Source:HGNC Symbol;Acc:HGNC:11913]	protein coding	1.04	6.48	48.75	0.00	0.00
ENSG00000145050	MANF	mesencephalic astrocyte derived neurotrophic factor [Source:HGNC Symbol;Acc:HGNC:15461]	protein coding	1.04	6.46	44.33	0.00	0.00
ENSG00000123131	PRDX4	peroxiredoxin 4 [Source:HGNC Symbol;Acc:HGNC:17169]	protein coding	1.03	5.30	27.07	0.00	0.00
ENSG00000153162	BMP6	bone morphogenetic protein 6 [Source:HGNC Symbol;Acc:HGNC:1073]	protein coding	1.03	2.57	12.60	0.00	0.01
ENSG00000102471	NDFIP2	Nedd4 family interacting protein 2 [Source:HGNC Symbol;Acc:HGNC:18537]	protein coding	1.03	2.09	13.72	0.00	0.01
ENSG00000132465	JCHAIN	joining chain of multimeric IgA and IgM [Source:HGNC Symbol;Acc:HGNC:5713]	protein coding	1.02	11.65	42.02	0.00	0.00
ENSG00000108641	B9D1	B9 domain containing 1 [Source:HGNC Symbol;Acc:HGNC:24123]	protein coding	1.02	2.11	10.72	0.00	0.02
ENSG00000093009	CDC45	cell division cycle 45 [Source:HGNC Symbol;Acc:HGNC:1739]	protein coding	1.02	1.85	10.37	0.00	0.02
ENSG00000102760	RGCC	regulator of cell cycle [Source:HGNC Symbol;Acc:HGNC:20369]	protein coding	1.02	2.50	18.10	0.00	0.00
ENSG00000171241	SHCBP1	SHC binding and spindle associated 1 [Source:HGNC Symbol;Acc:HGNC:29547]	protein coding	1.01	4.43	23.14	0.00	0.00
ENSG00000282600			IG V pseudogene	1.01	2.20	13.26	0.00	0.01
ENSG00000198794	SCAMP5	secretory carrier membrane protein 5 [Source:HGNC Symbol;Acc:HGNC:30386]	protein coding	1.00	3.50	34.11	0.00	0.00
ENSG00000243238	IGKV2-30	immunoglobulin kappa variable 2-30 [Source:HGNC Symbol;Acc:HGNC:5785]	IG V gene	0.99	5.43	12.25	0.00	0.01
ENSG00000085840	ORC1	origin recognition complex subunit 1 [Source:HGNC Symbol;Acc:HGNC:8487]	protein coding	0.99	2.43	12.68	0.00	0.01
ENSG00000242076	IGKV1-33	immunoglobulin kappa variable 1-33 [Source:HGNC Symbol;Acc:HGNC:5737]	IG V gene	0.99	4.57	27.16	0.00	0.00
ENSG00000166794	PPIB	peptidylprolyl isomerase B [Source:HGNC Symbol;Acc:HGNC:9255]	protein coding	0.98	7.68	35.77	0.00	0.00
ENSG00000079931	MOXD1	monooxygenase DBH like 1 [Source:HGNC Symbol;Acc:HGNC:21063]	protein coding	0.98	3.83	39.35	0.00	0.00
ENSG00000109805	NCAPG	non-SMC condensin I complex subunit G [Source:HGNC Symbol;Acc:HGNC:24304]	protein coding	0.98	3.06	19.31	0.00	0.00
ENSG00000070081	NUCB2	nucleobindin 2 [Source:HGNC Symbol;Acc:HGNC:8044]	protein coding	0.98	5.65	33.46	0.00	0.00
ENSG00000143476	DTL	denticleless E3 ubiquitin protein ligase homolog [Source:HGNC Symbol;Acc:HGNC:30288]	protein coding	0.98	2.88	15.70	0.00	0.00
ENSG00000044574	HSPA5	heat shock protein family A (Hsp70) member 5 [Source:HGNC Symbol;Acc:HGNC:5238]	protein coding	0.97	9.22	35.86	0.00	0.00
ENSG00000103257	SLC7A5	solute carrier family 7 member 5 [Source:HGNC Symbol;Acc:HGNC:11063]	protein coding	0.97	5.44	28.25	0.00	0.00

Ensembl Gene ID	Ensembl Gene Name	Ensembl Gene Description	Gene Type	Log_2 Fold Change (Day 7 vs. pre-treatment)	Average Log_2 CPM	Likelihood Ratio Test Statistic	P-Value	FDR Adjusted P-Value
ENSG00000168268	NT5DC2	5'-nucleotidase domain containing 2 [Source:HGNC Symbol;Acc:HGNC:25717]	protein coding	0.97	5.59	25.99	0.00	0.00
ENSG00000211659	IGLV3-25	immunoglobulin lambda variable 3-25 [Source:HGNC Symbol;Acc:HGNC:5908]	IG V gene	0.96	7.26	18.32	0.00	0.00
ENSG00000122952	ZWINT	ZW10 interacting kinetochore protein [Source:HGNC Symbol;Acc:HGNC:13195]	protein coding	0.96	3.27	14.55	0.00	0.00
ENSG00000167861	HID1	HID1 domain containing [Source:HGNC Symbol;Acc:HGNC:15736]	protein coding	0.96	4.25	17.94	0.00	0.00
ENSG00000174132	FAM174A	family with sequence similarity 174 member A [Source:HGNC Symbol;Acc:HGNC:24943]	protein coding	0.95	1.89	13.90	0.00	0.01
ENSG00000111424	VDR	vitamin D (1,25- dihydroxyvitamin D3) receptor [Source:HGNC Symbol;Acc:HGNC:12679]	protein coding	0.95	3.57	25.07	0.00	0.00
ENSG00000170476	MZB1	marginal zone B and B1 cell specific protein [Source:HGNC Symbol;Acc:HGNC:30125]	protein coding	0.95	8.50	30.34	0.00	0.00
ENSG00000185164	NOMO2	NODAL modulator 2 [Source:HGNC Symbol;Acc:HGNC:22652]	protein coding	0.95	4.34	17.56	0.00	0.00
ENSG00000111885	MAN1A1	mannosidase alpha class 1A member 1 [Source:HGNC Symbol;Acc:HGNC:6821]	protein coding	0.94	8.04	33.68	0.00	0.00
ENSG00000134285	FKBP11	FK506 binding protein 11 [Source:HGNC Symbol;Acc:HGNC:18624]	protein coding	0.94	6.33	25.23	0.00	0.00
ENSG00000119326	CTNNAL1	catenin alpha like 1 [Source:HGNC Symbol;Acc:HGNC:2512]	protein coding	0.93	2.32	16.37	0.00	0.00
ENSG00000173334	TRIB1	tribbles pseudokinase 1 [Source:HGNC Symbol;Acc:HGNC:16891]	protein coding	0.93	5.93	30.73	0.00	0.00
ENSG00000166562	SEC11C	SEC11 homolog C, signal peptidase complex subunit [Source:HGNC Symbol;Acc:HGNC:23400]	protein coding	0.93	7.37	31.49	0.00	0.00
ENSG00000172965	MIR4435-2HG	MIR4435-2 host gene [Source:HGNC Symbol;Acc:HGNC:35163]	lincRNA	0.93	2.53	14.47	0.00	0.00
ENSG00000166803	KIAA0101	KIAA0101 [Source:HGNC Symbol;Acc:HGNC:28961]	protein coding	0.93	1.53	8.88	0.00	0.04
ENSG00000254395	IGHV4-55	immunoglobulin heavy variable 4-55 (pseudogene) [Source:HGNC Symbol;Acc:HGNC:5653]	IG V pseudogene	0.92	4.12	17.39	0.00	0.00
ENSG00000094804	CDC6	cell division cycle 6 [Source:HGNC Symbol;Acc:HGNC:1744]	protein coding	0.92	3.24	18.72	0.00	0.00
ENSG00000169679	BUB1	BUB1 mitotic checkpoint serine/threonine kinase [Source:HGNC Symbol;Acc:HGNC:1148]	protein coding	0.92	4.49	18.39	0.00	0.00
ENSG00000175063	UBE2C	ubiquitin conjugating enzyme E2 C [Source:HGNC Symbol;Acc:HGNC:15937]	protein coding	0.91	2.20	9.06	0.00	0.04
ENSG00000077152	UBE2T	ubiquitin conjugating enzyme E2 T [Source:HGNC Symbol;Acc:HGNC:25009]	protein coding	0.91	2.15	10.82	0.00	0.02
ENSG00000103226	NOMO3	NODAL modulator 3 [Source:HGNC Symbol;Acc:HGNC:25242]	protein coding	0.91	3.27	21.79	0.00	0.00
ENSG00000113615	SEC24A	SEC24 homolog A, COPII coat complex component [Source:HGNC Symbol;Acc:HGNC:10703]	protein coding	0.91	6.60	34.02	0.00	0.00
ENSG00000068912	ERLEC1	endoplasmic reticulum lectin 1 [Source:HGNC Symbol;Acc:HGNC:25222]	protein coding	0.90	6.64	40.00	0.00	0.00

Ensembl Gene ID	Ensembl Gene Name	Ensembl Gene Description	Gene Type	Log_2 Fold Change (Day 7 vs. pre-treatment)	Average Log_2 CPM	Likelihood Ratio Test Statistic	P-Value	FDR Adjusted P-Value
ENSG00000137807	KIF23	kinesin family member 23 [Source:HGNC Symbol;Acc:HGNC:6392]	protein coding	0.90	2.22	9.39	0.00	0.04
ENSG00000258572			lincRNA	0.90	2.61	14.82	0.00	0.00
ENSG00000253691	IGKV2OR22-4	immunoglobulin kappa variable 2/OR22-4 (pseudogene) [Source:HGNC Symbol;Acc:HGNC:5813]	IG V pseudogene	0.90	3.56	11.91	0.00	0.01
ENSG00000196189	SEMA4A	semaphorin 4A [Source:HGNC Symbol;Acc:HGNC:10729]	protein coding	0.90	6.70	33.39	0.00	0.00
ENSG00000101057	MYBL2	MYB proto-oncogene like 2 [Source:HGNC Symbol;Acc:HGNC:7548]	protein coding	0.90	5.96	29.80	0.00	0.00
ENSG00000211946	IGHV3-20	immunoglobulin heavy variable 3-20 [Source:HGNC Symbol;Acc:HGNC:5585]	IG V gene	0.90	4.18	23.95	0.00	0.00
ENSG00000070214	SLC44A1	solute carrier family 44 member 1 [Source:HGNC Symbol;Acc:HGNC:18798]	protein coding	0.90	7.13	36.03	0.00	0.00
ENSG00000147459	DOCK5	dedicator of cytokinesis 5 [Source:HGNC Symbol;Acc:HGNC:23476]	protein coding	-0.89	3.71	15.93	0.00	0.00
ENSG00000136770	DNAJC1	DnaJ heat shock protein family (Hsp40) member C1 [Source:HGNC Symbol;Acc:HGNC:20090]	protein coding	0.89	4.91	36.86	0.00	0.00
ENSG00000128590	DNAJB9	DnaJ heat shock protein family (Hsp40) member B9 [Source:HGNC Symbol;Acc:HGNC:6968]	protein coding	0.89	5.28	27.73	0.00	0.00
ENSG00000131871	SELENOS	selenoprotein S [Source:HGNC Symbol;Acc:HGNC:30396]	protein coding	0.89	5.56	42.42	0.00	0.00
ENSG00000171155	C1GALT1C1	C1GALT1 specific chaperone 1 [Source:HGNC Symbol;Acc:HGNC:24338]	protein coding	0.88	4.07	30.36	0.00	0.00
ENSG00000253818	IGLV1-41	immunoglobulin lambda variable 1-41 (pseudogene) [Source:HGNC Symbol;Acc:HGNC:5878]	IG V pseudogene	0.88	3.91	15.30	0.00	0.00
ENSG00000071539	TRIP13	thyroid hormone receptor interactor 13 [Source:HGNC Symbol;Acc:HGNC:12307]	protein coding	0.87	2.56	16.14	0.00	0.00
ENSG00000118705	RPN2	ribophorin II [Source:HGNC Symbol;Acc:HGNC:10382]	protein coding	0.87	8.34	39.93	0.00	0.00
ENSG00000010310	GIPR	gastric inhibitory polypeptide receptor [Source:HGNC Symbol;Acc:HGNC:4271]	protein coding	0.87	2.28	10.01	0.00	0.03
ENSG00000173848	NET1	neuroepithelial cell transforming 1 [Source:HGNC Symbol;Acc:HGNC:14592]	protein coding	0.87	4.27	28.05	0.00	0.00
ENSG00000146670	CDCA5	cell division cycle associated 5 [Source:HGNC Symbol;Acc:HGNC:14626]	protein coding	0.87	2.72	13.29	0.00	0.01
ENSG00000211897	IGHG3	immunoglobulin heavy constant gamma 3 (G3m marker) [Source:HGNC Symbol;Acc:HGNC:5527]	IG C gene	0.86	9.09	20.28	0.00	0.00
ENSG00000136026	CKAP4	cytoskeleton associated protein 4 [Source:HGNC Symbol;Acc:HGNC:16991]	protein coding	0.86	6.65	38.36	0.00	0.00
ENSG00000179750	APOBEC3B	apolipoprotein B mRNA editing enzyme catalytic subunit 3B [Source:HGNC Symbol;Acc:HGNC:17352]	protein coding	0.86	2.89	13.05	0.00	0.01
ENSG00000153066	TXNDC11	thioredoxin domain containing 11 [Source:HGNC Symbol;Acc:HGNC:28030]	protein coding	0.86	7.71	28.79	0.00	0.00
ENSG00000139193	CD27	CD27 molecule [Source:HGNC Symbol;Acc:HGNC:11922]	protein coding	0.86	5.98	28.90	0.00	0.00

Ensembl Gene ID	Ensembl Gene Name	Ensembl Gene Description	Gene Type	Log_2 Fold Change (Day 7 vs. pre-treatment)	Average Log_2 CPM	Likelihood Ratio Test Statistic	P-Value	FDR Adjusted P-Value
ENSG00000178999	AURKB	aurora kinase B [Source:HGNC Symbol;Acc:HGNC:11390]	protein coding	0.85	2.35	13.12	0.00	0.01
ENSG00000074695	LMAN1	lectin, mannose binding 1 [Source:HGNC Symbol;Acc:HGNC:6631]	protein coding	0.85	7.89	28.71	0.00	0.00
ENSG00000211967	IGHV3-53	immunoglobulin heavy variable 3-53 [Source:HGNC Symbol;Acc:HGNC:5610]	IG V gene	0.84	5.97	12.80	0.00	0.01
ENSG00000075218	GTSE1	G2 and S-phase expressed 1 [Source:HGNC Symbol;Acc:HGNC:13698]	protein coding	0.84	2.41	12.94	0.00	0.01
ENSG00000111291	GPRC5D	G protein-coupled receptor class C group 5 member D [Source:HGNC Symbol;Acc:HGNC:13310]	protein coding	0.84	3.92	18.27	0.00	0.00
ENSG00000078900	TP73	tumor protein p73 [Source:HGNC Symbol;Acc:HGNC:12003]	protein coding	0.83	1.97	10.11	0.00	0.03
ENSG00000092621	PHGDH	phosphoglycerate dehydrogenase [Source:HGNC Symbol;Acc:HGNC:8923]	protein coding	0.83	3.90	10.87	0.00	0.02
ENSG00000271178	IGHV3OR16-13	immunoglobulin heavy variable 3/OR16-13 (non-functional) [Source:HGNC Symbol;Acc:HGNC:5637]	IG V gene	0.83	3.66	11.43	0.00	0.02
ENSG00000183508	FAM46C	family with sequence similarity 46 member C [Source:HGNC Symbol;Acc:HGNC:24712]	protein coding	0.83	8.49	31.35	0.00	0.00
ENSG00000087586	AURKA	aurora kinase A [Source:HGNC Symbol;Acc:HGNC:11393]	protein coding	0.83	2.40	9.45	0.00	0.03
ENSG00000089685	BIRC5	baculoviral IAP repeat containing 5 [Source:HGNC Symbol;Acc:HGNC:593]	protein coding	0.83	2.97	13.22	0.00	0.01
ENSG00000106803	SEC61B	Sec61 translocon beta subunit [Source:HGNC Symbol;Acc:HGNC:16993]	protein coding	0.82	6.11	44.26	0.00	0.00
ENSG00000211964	IGHV3-48	immunoglobulin heavy variable 3-48 [Source:HGNC Symbol;Acc:HGNC:5606]	IG V gene	0.82	7.39	22.69	0.00	0.00
ENSG00000163694	RBM47	RNA binding motif protein 47 [Source:HGNC Symbol;Acc:HGNC:30358]	protein coding	0.82	4.83	31.02	0.00	0.00
ENSG00000100629	CEP128	centrosomal protein 128 [Source:HGNC Symbol;Acc:HGNC:20359]	protein coding	0.81	4.29	18.83	0.00	0.00
ENSG00000198855	FICD	FIC domain containing [Source:HGNC Symbol;Acc:HGNC:18416]	protein coding	0.81	3.28	12.32	0.00	0.01
ENSG00000138180	CEP55	centrosomal protein 55 [Source:HGNC Symbol;Acc:HGNC:1161]	protein coding	0.81	2.47	9.11	0.00	0.04
ENSG00000150967	ABCB9	ATP binding cassette subfamily B member 9 [Source:HGNC Symbol;Acc:HGNC:50]	protein coding	0.80	4.02	15.47	0.00	0.00
ENSG00000074416	MGLL	monoglyceride lipase [Source:HGNC Symbol;Acc:HGNC:17038]	protein coding	0.80	3.72	24.90	0.00	0.00
ENSG00000125844	RRBP1	ribosome binding protein 1 [Source:HGNC Symbol;Acc:HGNC:10448]	protein coding	0.80	7.11	43.20	0.00	0.00
ENSG00000083444	PLOD1	procollagen-lysine,2-oxoglutarate 5-dioxygenase 1 [Source:HGNC Symbol;Acc:HGNC:9081]	protein coding	0.80	4.46	21.59	0.00	0.00
ENSG00000155304	HSPA13	heat shock protein family A (Hsp70) member 13 [Source:HGNC Symbol;Acc:HGNC:11375]	protein coding	0.80	6.17	27.91	0.00	0.00
ENSG00000117399	CDC20	cell division cycle 20 [Source:HGNC Symbol;Acc:HGNC:1723]	protein coding	0.79	3.40	14.69	0.00	0.00

Ensembl Gene ID	Ensembl Gene Name	Ensembl Gene Description	Gene Type	Log_2 Fold Change (Day 7 vs. pre-treatment)	Average Log_2 CPM	Likelihood Ratio Test Statistic	P-Value	FDR Adjusted P-Value
ENSG00000204634	TBC1D8	TBC1 domain family member 8 [Source:HGNC Symbol;Acc:HGNC:17791]	protein coding	-0.79	2.98	12.76	0.00	0.01
ENSG00000150961	SEC24D	SEC24 homolog D, COPII coat complex component [Source:HGNC Symbol;Acc:HGNC:10706]	protein coding	0.79	6.48	30.83	0.00	0.00
ENSG00000065308	TRAM2	translocation associated membrane protein 2 [Source:HGNC Symbol;Acc:HGNC:16855]	protein coding	0.79	7.05	34.02	0.00	0.00
ENSG00000165948	IFI27L1	interferon alpha inducible protein 27 like 1 [Source:HGNC Symbol;Acc:HGNC:19754]	protein coding	0.79	2.50	14.06	0.00	0.01
ENSG00000134910	STT3A	STT3A, catalytic subunit of the oligosaccharyltransferase complex [Source:HGNC Symbol;Acc:HGNC:6172]	protein coding	0.79	7.28	35.63	0.00	0.00
ENSG00000017483	SLC38A5	solute carrier family 38 member 5 [Source:HGNC Symbol;Acc:HGNC:18070]	protein coding	0.79	5.08	21.45	0.00	0.00
ENSG00000072571	HMMR	hyaluronan mediated motility receptor [Source:HGNC Symbol;Acc:HGNC:5012]	protein coding	0.79	2.77	10.76	0.00	0.02
ENSG00000132432	SEC61G	Sec61 translocon gamma subunit [Source:HGNC Symbol;Acc:HGNC:18277]	protein coding	0.78	5.28	30.24	0.00	0.00
ENSG00000227203	SUB1P1	SUB1 homolog, transcriptional regulator pseudogene 1 [Source:HGNC Symbol;Acc:HGNC:32300]	processed pseudogene	0.78	2.08	10.65	0.00	0.02
ENSG00000182054	IDH2	isocitrate dehydrogenase (NADP(+)) 2, mitochondrial [Source:HGNC Symbol;Acc:HGNC:5383]	protein coding	0.78	6.71	25.88	0.00	0.00
ENSG00000106415	GLCCI1	glucocorticoid induced 1 [Source:HGNC Symbol;Acc:HGNC:18713]	protein coding	0.78	6.60	31.84	0.00	0.00
ENSG00000224041	IGKV3D-15	immunoglobulin kappa variable 3D-15 (gene/pseudogene) [Source:HGNC Symbol;Acc:HGNC:5824]	IG V gene	0.78	4.67	39.34	0.00	0.00
ENSG00000111665	CDCA3	cell division cycle associated 3 [Source:HGNC Symbol;Acc:HGNC:14624]	protein coding	0.78	2.12	9.27	0.00	0.04
ENSG00000124788	ATXN1	ataxin 1 [Source:HGNC Symbol;Acc:HGNC:10548]	protein coding	0.77	4.94	28.09	0.00	0.00
ENSG00000185155	MIXL1	Mix paired-like homeobox [Source:HGNC Symbol;Acc:HGNC:13363]	protein coding	0.77	2.81	12.27	0.00	0.01
ENSG00000241755	IGKV1-9	immunoglobulin kappa variable 1-9 [Source:HGNC Symbol;Acc:HGNC:5744]	IG V gene	0.77	6.00	8.95	0.00	0.04
ENSG00000029153	ARNTL2	aryl hydrocarbon receptor nuclear translocator like 2 [Source:HGNC Symbol;Acc:HGNC:18984]	protein coding	0.77	2.75	11.49	0.00	0.02
ENSG00000004866	ST7	suppression of tumorigenicity 7 [Source:HGNC Symbol;Acc:HGNC:11351]	protein coding	0.77	2.90	17.04	0.00	0.00
ENSG00000112378	PERP	PERP, TP53 apoptosis effector [Source:HGNC Symbol;Acc:HGNC:17637]	protein coding	0.77	3.82	15.45	0.00	0.00
ENSG00000120725	SIL1	SIL1 nucleotide exchange factor [Source:HGNC Symbol;Acc:HGNC:24624]	protein coding	0.77	4.32	19.83	0.00	0.00
ENSG00000211892	IGHG4	immunoglobulin heavy constant gamma 4 (G4m marker) [Source:HGNC Symbol;Acc:HGNC:5528]	IG C gene	0.77	7.21	22.77	0.00	0.00
ENSG00000164611	PTTG1	pituitary tumor-transforming 1 [Source:HGNC Symbol;Acc:HGNC:9690]	protein coding	0.77	3.37	18.35	0.00	0.00

Ensembl Gene ID	Ensembl Gene Name	Ensembl Gene Description	Gene Type	Log_2 Fold Change (Day 7 vs. pre-treatment)	Average Log_2 CPM	Likelihood Ratio Test Statistic	P-Value	FDR Adjusted P-Value
ENSG00000185624	P4HB	prolyl 4-hydroxylase subunit beta [Source:HGNC Symbol;Acc:HGNC:8548]	protein coding	0.76	8.34	24.83	0.00	0.00
ENSG00000211943	IGHV3-15	immunoglobulin heavy variable 3-15 [Source:HGNC Symbol;Acc:HGNC:5582]	IG V gene	0.76	6.81	10.81	0.00	0.02
ENSG00000163902	RPN1	ribophorin I [Source:HGNC Symbol;Acc:HGNC:10381]	protein coding	0.76	7.90	36.02	0.00	0.00
ENSG00000138778	CENPE	centromere protein E [Source:HGNC Symbol;Acc:HGNC:1856]	protein coding	0.76	3.46	11.76	0.00	0.01
ENSG00000244038	DDOST	dolichyl-diphosphooligosaccharide–protein glycosyltransferase non-catalytic subunit [Source:HGNC Symbol;Acc:HGNC:2728]	protein coding	0.75	7.88	24.40	0.00	0.00
ENSG00000168701	TMEM208	transmembrane protein 208 [Source:HGNC Symbol;Acc:HGNC:25015]	protein coding	0.75	4.09	21.16	0.00	0.00
ENSG00000148773	MKI67	marker of proliferation Ki-67 [Source:HGNC Symbol;Acc:HGNC:7107]	protein coding	0.75	6.17	14.54	0.00	0.00
ENSG00000100342	APOL1	apolipoprotein L1 [Source:HGNC Symbol;Acc:HGNC:618]	protein coding	0.75	4.70	26.31	0.00	0.00
ENSG00000211679	IGLC3	immunoglobulin lambda constant 3 (Kern-Oz+ marker) [Source:HGNC Symbol;Acc:HGNC:5857]	IG C gene	0.75	9.17	14.18	0.00	0.01
ENSG00000011478	QPCTL	glutaminyl-peptide cyclotransferase like [Source:HGNC Symbol;Acc:HGNC:25952]	protein coding	0.74	4.32	24.49	0.00	0.00
ENSG00000198937	CCDC167	coiled-coil domain containing 167 [Source:HGNC Symbol;Acc:HGNC:21239]	protein coding	0.74	3.77	17.76	0.00	0.00
ENSG00000128595	CALU	calumenin [Source:HGNC Symbol;Acc:HGNC:1458]	protein coding	0.74	6.14	29.74	0.00	0.00
ENSG00000114850	SSR3	signal sequence receptor subunit 3 [Source:HGNC Symbol;Acc:HGNC:11325]	protein coding	0.74	7.74	26.81	0.00	0.00
ENSG00000172469	MANEA	mannosidase endo-alpha [Source:HGNC Symbol;Acc:HGNC:21072]	protein coding	0.74	5.85	18.64	0.00	0.00
ENSG00000189233	NUGGC	nuclear GTPase, germinal center associated [Source:HGNC Symbol;Acc:HGNC:33550]	protein coding	0.74	4.82	21.03	0.00	0.00
ENSG00000143870	PDIA6	protein disulfide isomerase family A member 6 [Source:HGNC Symbol;Acc:HGNC:30168]	protein coding	0.74	7.61	29.56	0.00	0.00
ENSG00000135069	PSAT1	phosphoserine aminotransferase 1 [Source:HGNC Symbol;Acc:HGNC:19129]	protein coding	0.74	3.74	10.49	0.00	0.02
ENSG00000184164	CRELD2	cysteine rich with EGF like domains 2 [Source:HGNC Symbol;Acc:HGNC:28150]	protein coding	0.74	5.99	18.80	0.00	0.00
ENSG00000109501	WFS1	wolframin ER transmembrane glycoprotein [Source:HGNC Symbol;Acc:HGNC:12762]	protein coding	0.74	4.11	13.41	0.00	0.01
ENSG00000101294	HM13	histocompatibility minor 13 [Source:HGNC Symbol;Acc:HGNC:16435]	protein coding	0.73	7.02	20.56	0.00	0.00
ENSG00000274422			lincRNA	-0.73	2.94	9.61	0.00	0.03
ENSG00000211644	IGLV1-51	immunoglobulin lambda variable 1-51 [Source:HGNC Symbol;Acc:HGNC:5882]	IG V gene	0.72	6.00	15.96	0.00	0.00
ENSG00000123352	SPATS2	spermatogenesis associated serine rich 2 [Source:HGNC Symbol;Acc:HGNC:18650]	protein coding	0.72	5.57	24.13	0.00	0.00

Ensembl Gene ID	Ensembl Gene Name	Ensembl Gene Description	Gene Type	Log_2 Fold Change (Day 7 vs. pre-treatment)	Average Log_2 CPM	Likelihood Ratio Test Statistic	P-Value	FDR Adjusted P-Value
ENSG00000002549	LAP3	leucine aminopeptidase 3 [Source:HGNC Symbol;Acc:HGNC:18449]	protein coding	0.71	5.86	21.75	0.00	0.00
ENSG00000102096	PIM2	Pim-2 proto-oncogene, serine/threonine kinase [Source:HGNC Symbol;Acc:HGNC:8987]	protein coding	0.71	7.88	32.56	0.00	0.00
ENSG00000211649	IGLV7-46	immunoglobulin lambda variable 7-46 (gene/pseudogene) [Source:HGNC Symbol;Acc:HGNC:5930]	IG V gene	0.71	5.22	14.31	0.00	0.00
ENSG00000149428	HYOU1	hypoxia up-regulated 1 [Source:HGNC Symbol;Acc:HGNC:16931]	protein coding	0.71	7.68	20.85	0.00	0.00
ENSG00000198876	DCAF12	DDB1 and CUL4 associated factor 12 [Source:HGNC Symbol;Acc:HGNC:19911]	protein coding	0.71	4.45	16.87	0.00	0.00
ENSG00000111206	FOXM1	forkhead box M1 [Source:HGNC Symbol;Acc:HGNC:3818]	protein coding	0.71	3.19	18.25	0.00	0.00
ENSG00000102580	DNAJC3	DnaJ heat shock protein family (Hsp40) member C3 [Source:HGNC Symbol;Acc:HGNC:9439]	protein coding	0.71	6.71	28.06	0.00	0.00
ENSG00000103512	NOMO1	NODAL modulator 1 [Source:HGNC Symbol;Acc:HGNC:30060]	protein coding	0.71	5.32	17.01	0.00	0.00
ENSG00000108829	LRRC59	leucine rich repeat containing 59 [Source:HGNC Symbol;Acc:HGNC:28817]	protein coding	0.70	6.50	23.64	0.00	0.00
ENSG00000241351	IGKV3-11	immunoglobulin kappa variable 3-11 [Source:HGNC Symbol;Acc:HGNC:5815]	IG V gene	0.70	7.41	28.67	0.00	0.00
ENSG00000134825	TMEM258	transmembrane protein 258 [Source:HGNC Symbol;Acc:HGNC:1164]	protein coding	0.70	5.70	32.65	0.00	0.00
ENSG00000198856	OSTC	oligosaccharyltransferase complex non-catalytic subunit [Source:HGNC Symbol;Acc:HGNC:24448]	protein coding	0.70	6.11	33.85	0.00	0.00
ENSG00000051341	POLQ	DNA polymerase theta [Source:HGNC Symbol;Acc:HGNC:9186]	protein coding	0.70	2.87	13.44	0.00	0.01
ENSG00000120708	TGFBI	transforming growth factor beta induced [Source:HGNC Symbol;Acc:HGNC:11771]	protein coding	-0.70	3.44	8.52	0.00	0.05
ENSG00000165272	AQP3	aquaporin 3 (Gill blood group) [Source:HGNC Symbol;Acc:HGNC:636]	protein coding	0.70	6.08	24.62	0.00	0.00
ENSG00000134057	CCNB1	cyclin B1 [Source:HGNC Symbol;Acc:HGNC:1579]	protein coding	0.70	3.37	16.04	0.00	0.00
ENSG00000197714	ZNF460	zinc finger protein 460 [Source:HGNC Symbol;Acc:HGNC:21628]	protein coding	-0.69	3.07	13.51	0.00	0.01
ENSG00000144724	PTPRG	protein tyrosine phosphatase, receptor type G [Source:HGNC Symbol;Acc:HGNC:9671]	protein coding	0.69	2.70	12.21	0.00	0.01
ENSG00000117143	UAP1	UDP-N-acetylglucosamine pyrophosphorylase 1 [Source:HGNC Symbol;Acc:HGNC:12457]	protein coding	0.69	5.59	35.74	0.00	0.00
ENSG00000184840	TMED9	transmembrane p24 trafficking protein 9 [Source:HGNC Symbol;Acc:HGNC:24878]	protein coding	0.69	6.90	31.51	0.00	0.00
ENSG00000160883	HK3	hexokinase 3 [Source:HGNC Symbol;Acc:HGNC:4925]	protein coding	-0.69	4.71	14.55	0.00	0.00
ENSG00000169223	LMAN2	lectin, mannose binding 2 [Source:HGNC Symbol;Acc:HGNC:16986]	protein coding	0.68	7.00	23.39	0.00	0.00
ENSG00000114902	SPCS1	signal peptidase complex subunit 1 [Source:HGNC Symbol;Acc:HGNC:23401]	protein coding	0.68	6.73	39.09	0.00	0.00

Ensembl Gene ID	Ensembl Gene Name	Ensembl Gene Description	Gene Type	Log_2 Fold Change (Day 7 vs. pre-treatment)	Average Log_2 CPM	Likelihood Ratio Test Statistic	P-Value	FDR Adjusted P-Value
ENSG00000090520	DNAJB11	DnaJ heat shock protein family (Hsp40) member B11 [Source:HGNC Symbol;Acc:HGNC:14889]	protein coding	0.68	6.03	23.10	0.00	0.00
ENSG00000115677	HDLBP	high density lipoprotein binding protein [Source:HGNC Symbol;Acc:HGNC:4857]	protein coding	0.68	8.52	16.85	0.00	0.00
ENSG00000260948			sense overlapping	-0.68	2.26	8.85	0.00	0.04
ENSG00000136240	KDELR2	KDEL endoplasmic reticulum protein retention receptor 2 [Source:HGNC Symbol;Acc:HGNC:6305]	protein coding	0.68	6.21	21.19	0.00	0.00
ENSG00000121073	SLC35B1	solute carrier family 35 member B1 [Source:HGNC Symbol;Acc:HGNC:20798]	protein coding	0.67	5.33	19.29	0.00	0.00
ENSG00000142675	CNKSR1	connector enhancer of kinase suppressor of Ras 1 [Source:HGNC Symbol;Acc:HGNC:19700]	protein coding	0.67	3.82	14.90	0.00	0.00
ENSG00000117411	B4GALT2	beta-1,4-galactosyltransferase 2 [Source:HGNC Symbol;Acc:HGNC:925]	protein coding	0.67	3.39	10.29	0.00	0.03
ENSG00000176658	MYO1D	myosin ID [Source:HGNC Symbol;Acc:HGNC:7598]	protein coding	0.67	5.94	25.17	0.00	0.00
ENSG00000143942	CHAC2	ChaC cation transport regulator homolog 2 [Source:HGNC Symbol;Acc:HGNC:32363]	protein coding	0.66	3.01	16.94	0.00	0.00
ENSG00000172339	ALG14	ALG14, UDP-N-acetylglucosaminyltransferase subunit [Source:HGNC Symbol;Acc:HGNC:28287]	protein coding	0.66	3.21	12.37	0.00	0.01
ENSG00000173540	GMPPB	GDP-mannose pyrophosphorylase B [Source:HGNC Symbol;Acc:HGNC:22932]	protein coding	0.66	5.40	23.17	0.00	0.00
ENSG00000224699	LAMTOR5-AS1	LAMTOR5 antisense RNA 1 [Source:HGNC Symbol;Acc:HGNC:40823]	processed transcript	-0.66	2.43	9.51	0.00	0.03
ENSG00000070540	WIPI1	WD repeat domain, phosphoinositide interacting 1 [Source:HGNC Symbol;Acc:HGNC:25471]	protein coding	0.66	4.19	14.01	0.00	0.01
ENSG00000088325	TPX2	TPX2, microtubule nucleation factor [Source:HGNC Symbol;Acc:HGNC:1249]	protein coding	0.66	3.62	12.38	0.00	0.01
ENSG00000133328	HRASLS2	HRAS like suppressor 2 [Source:HGNC Symbol;Acc:HGNC:17824]	protein coding	0.66	3.11	12.31	0.00	0.01
ENSG00000115902	SLC1A4	solute carrier family 1 member 4 [Source:HGNC Symbol;Acc:HGNC:10942]	protein coding	0.66	6.07	19.46	0.00	0.00
ENSG00000110063	DCPS	decapping enzyme, scavenger [Source:HGNC Symbol;Acc:HGNC:29812]	protein coding	0.65	4.93	27.22	0.00	0.00
ENSG00000157456	CCNB2	cyclin B2 [Source:HGNC Symbol;Acc:HGNC:1580]	protein coding	0.65	2.94	11.61	0.00	0.01
ENSG00000167900	TK1	thymidine kinase 1 [Source:HGNC Symbol;Acc:HGNC:11830]	protein coding	0.65	3.74	24.47	0.00	0.00
ENSG00000129235	TXNDC17	thioredoxin domain containing 17 [Source:HGNC Symbol;Acc:HGNC:28218]	protein coding	0.65	3.45	14.35	0.00	0.00
ENSG00000211632	IGKV3D-11	immunoglobulin kappa variable 3D-11 [Source:HGNC Symbol;Acc:HGNC:5823]	IG V gene	0.65	4.11	12.93	0.00	0.01
ENSG00000211952	IGHV4-28	immunoglobulin heavy variable 4-28 [Source:HGNC Symbol;Acc:HGNC:5645]	IG V gene	0.65	3.53	10.19	0.00	0.03
ENSG00000204386	NEU1	neuraminidase 1 [Source:HGNC Symbol;Acc:HGNC:7758]	protein coding	0.64	4.98	17.45	0.00	0.00

Ensembl Gene ID	Ensembl Gene Name	Ensembl Gene Description	Gene Type	Log_2 Fold Change (Day 7 vs. pre-treatment)	Average Log_2 CPM	Likelihood Ratio Test Statistic	P-Value	FDR Adjusted P-Value
ENSG00000057657	PRDM1	PR/SET domain 1 [Source:HGNC Symbol;Acc:HGNC:9346]	protein coding	0.64	6.14	30.36	0.00	0.00
ENSG00000146733	PSPH	phosphoserine phosphatase [Source:HGNC Symbol;Acc:HGNC:9577]	protein coding	0.64	2.35	8.97	0.00	0.04
ENSG00000279192	PWAR5	Prader Willi/Angelman region RNA 5 [Source:HGNC Symbol;Acc:HGNC:30090]	TEC	-0.64	3.24	10.37	0.00	0.02
ENSG00000140105	WARS	tryptophanyl-tRNA synthetase [Source:HGNC Symbol;Acc:HGNC:12729]	protein coding	0.64	7.78	13.81	0.00	0.01
ENSG00000180879	SSR4	signal sequence receptor subunit 4 [Source:HGNC Symbol;Acc:HGNC:11326]	protein coding	0.64	7.43	24.99	0.00	0.00
ENSG00000107719	PALD1	phosphatase domain containing, paladin 1 [Source:HGNC Symbol;Acc:HGNC:23530]	protein coding	0.64	2.77	10.80	0.00	0.02
ENSG00000140451	PIF1	PIF1 5'-to-3' DNA helicase [Source:HGNC Symbol;Acc:HGNC:26220]	protein coding	0.63	2.80	11.02	0.00	0.02
ENSG00000122862	SRGN	serglycin [Source:HGNC Symbol;Acc:HGNC:9361]	protein coding	0.63	7.13	22.51	0.00	0.00
ENSG00000071537	SEL1L	SEL1L ERAD E3 ligase adaptor subunit [Source:HGNC Symbol;Acc:HGNC:10717]	protein coding	0.63	7.49	23.73	0.00	0.00
ENSG00000136840	ST6GALNAC4	ST6 N-acetylgalactosaminide alpha-2,6-sialyltransferase 4 [Source:HGNC Symbol;Acc:HGNC:17846]	protein coding	0.63	5.46	22.48	0.00	0.00
ENSG00000187837	HIST1H1C	histone cluster 1 H1 family member c [Source:HGNC Symbol;Acc:HGNC:4716]	protein coding	0.63	3.73	12.05	0.00	0.01
ENSG00000211933	IGHV6-1	immunoglobulin heavy variable 6-1 [Source:HGNC Symbol;Acc:HGNC:5662]	IG V gene	0.62	6.10	15.55	0.00	0.00
ENSG00000182985	CADM1	cell adhesion molecule 1 [Source:HGNC Symbol;Acc:HGNC:5951]	protein coding	0.62	3.24	11.53	0.00	0.02
ENSG00000183087	GAS6	growth arrest specific 6 [Source:HGNC Symbol;Acc:HGNC:4168]	protein coding	0.62	3.58	8.89	0.00	0.04
ENSG00000058262	SEC61A1	Sec61 translocon alpha 1 subunit [Source:HGNC Symbol;Acc:HGNC:18276]	protein coding	0.62	8.26	21.98	0.00	0.00
ENSG00000086062	B4GALT1	beta-1,4-galactosyltransferase 1 [Source:HGNC Symbol;Acc:HGNC:924]	protein coding	0.62	8.55	20.78	0.00	0.00
ENSG00000278897			TEC	-0.62	3.53	15.27	0.00	0.00
ENSG00000113621	TXNDC15	thioredoxin domain containing 15 [Source:HGNC Symbol;Acc:HGNC:20652]	protein coding	0.61	5.53	22.30	0.00	0.00
ENSG00000136810	TXN	thioredoxin [Source:HGNC Symbol;Acc:HGNC:12435]	protein coding	0.61	5.05	26.75	0.00	0.00
ENSG00000182481	KPNA2	karyopherin subunit alpha 2 [Source:HGNC Symbol;Acc:HGNC:6395]	protein coding	0.61	4.98	30.75	0.00	0.00
ENSG00000186818	LILRB4	leukocyte immunoglobulin like receptor B4 [Source:HGNC Symbol;Acc:HGNC:6608]	protein coding	0.61	3.51	15.57	0.00	0.00
ENSG00000160712	IL6R	interleukin 6 receptor [Source:HGNC Symbol;Acc:HGNC:6019]	protein coding	0.61	6.44	12.94	0.00	0.01
ENSG00000134153	EMC7	ER membrane protein complex subunit 7 [Source:HGNC Symbol;Acc:HGNC:24301]	protein coding	0.61	5.04	26.53	0.00	0.00

Ensembl Gene ID	Ensembl Gene Name	Ensembl Gene Description	Gene Type	Log_2 Fold Change (Day 7 vs. pre-treatment)	Average Log_2 CPM	Likelihood Ratio Test Statistic	P-Value	FDR Adjusted P-Value
ENSG00000168374	ARF4	ADP ribosylation factor 4 [Source:HGNC Symbol;Acc:HGNC:655]	protein coding	0.61	6.51	28.29	0.00	0.00
ENSG00000065911	MTHFD2	methylenetetrahydrofolate dehydrogenase (NADP+ dependent) 2, methenyltetrahydrofolate cyclohydrolase [Source:HGNC Symbol;Acc:HGNC:7434]	protein coding	0.61	5.29	13.05	0.00	0.01
ENSG00000099337	KCNK6	potassium two pore domain channel subfamily K member 6 [Source:HGNC Symbol;Acc:HGNC:6281]	protein coding	0.60	5.17	13.91	0.00	0.01
ENSG00000233806	LINC01237	long intergenic non-protein coding RNA 1237 [Source:HGNC Symbol;Acc:HGNC:49793]	processed transcript	-0.60	3.53	14.07	0.00	0.01
ENSG00000173486	FKBP2	FK506 binding protein 2 [Source:HGNC Symbol;Acc:HGNC:3718]	protein coding	0.60	4.96	16.20	0.00	0.00
ENSG00000113811	SELENOK	selenoprotein K [Source:HGNC Symbol;Acc:HGNC:30394]	protein coding	0.60	5.15	23.44	0.00	0.00
ENSG00000173218	VANGL1	VANGL planar cell polarity protein 1 [Source:HGNC Symbol;Acc:HGNC:15512]	protein coding	0.60	2.79	9.79	0.00	0.03
ENSG00000179218	CALR	calreticulin [Source:HGNC Symbol;Acc:HGNC:1455]	protein coding	0.60	9.21	18.63	0.00	0.00
ENSG00000198018	ENTPD7	ectonucleoside triphosphate diphosphohydrolase 7 [Source:HGNC Symbol;Acc:HGNC:19745]	protein coding	0.60	3.24	8.45	0.00	0.05
ENSG00000154723	ATP5J	ATP synthase, H+ transporting, mitochondrial Fo complex subunit F6 [Source:HGNC Symbol;Acc:HGNC:847]	protein coding	0.60	5.17	33.59	0.00	0.00
ENSG00000164109	MAD2L1	MAD2 mitotic arrest deficient-like 1 (yeast) [Source:HGNC Symbol;Acc:HGNC:6763]	protein coding	0.60	3.16	14.36	0.00	0.00
ENSG00000120697	ALG5	ALG5, dolichyl-phosphate beta-glucosyltransferase [Source:HGNC Symbol;Acc:HGNC:20266]	protein coding	0.60	4.84	22.58	0.00	0.00
ENSG00000180747	SMG1P3	SMG1P3, nonsense mediated mRNA decay associated PI3K related kinase pseudogene 3 [Source:HGNC Symbol;Acc:HGNC:49860]	transcribed unprocessed pseudogene	-0.59	3.28	10.78	0.00	0.02
ENSG00000080986	NDC80	NDC80, kinetochore complex component [Source:HGNC Symbol;Acc:HGNC:16909]	protein coding	0.59	3.04	9.05	0.00	0.04
ENSG00000116741	RGS2	regulator of G-protein signaling 2 [Source:HGNC Symbol;Acc:HGNC:9998]	protein coding	0.59	5.80	9.60	0.00	0.03
ENSG00000116649	SRM	spermidine synthase [Source:HGNC Symbol;Acc:HGNC:11296]	protein coding	0.59	5.91	23.28	0.00	0.00
ENSG00000095380	NANS	N-acetylneuraminate synthase [Source:HGNC Symbol;Acc:HGNC:19237]	protein coding	0.59	4.43	16.83	0.00	0.00
ENSG00000259772			lincRNA	0.59	3.99	11.27	0.00	0.02
ENSG00000198792	TMEM184B	transmembrane protein 184B [Source:HGNC Symbol;Acc:HGNC:1310]	protein coding	0.59	4.85	21.21	0.00	0.00
ENSG00000113387	SUB1	SUB1 homolog, transcriptional regulator [Source:HGNC Symbol;Acc:HGNC:19985]	protein coding	0.59	8.19	26.38	0.00	0.00
ENSG00000166825	ANPEP	alanyl aminopeptidase, membrane [Source:HGNC Symbol;Acc:HGNC:500]	protein coding	-0.59	3.37	10.89	0.00	0.02

Ensembl Gene ID	Ensembl Gene Name	Ensembl Gene Description	Gene Type	Log_2 Fold Change (Day 7 vs. pre-treatment)	Average Log_2 CPM	Likelihood Ratio Test Statistic	P-Value	FDR Adjusted P-Value
ENSG00000144867	SRPRB	SRP receptor beta subunit [Source:HGNC Symbol;Acc:HGNC:24085]	protein coding	0.58	5.69	18.60	0.00	0.00

Table 13: Genes differentially expressed at Day 7 compared to pre-treatment (B Cells, Trivalent Influenza Vaccine). Sorted by descending absolute log_2 fold change (Day 7 vs. pre-treatment). Gene model summaries and annotations are based on Ensembl Version 87.

Ensembl Gene ID	Ensembl Gene Name	Ensembl Gene Description	Gene Type	Log_2 Fold Change (Day 8 vs. pre-treatment)	Average Log_2 CPM	Likelihood Ratio Test Statistic	P-Value	FDR Adjusted P-Value
ENSG00000254176	IGHV3-75	immunoglobulin heavy variable 3-75 (pseudogene) [Source:HGNC Symbol;Acc:HGNC:5625]	IG V pseudogene	6.80	0.23	23.47	0.00	0.00
ENSG00000253822	IGLV3-24	immunoglobulin lambda variable 3-24 (pseudogene) [Source:HGNC Symbol;Acc:HGNC:5907]	IG V pseudogene	2.55	2.44	11.39	0.00	0.04
ENSG00000254174	IGHV1-12	immunoglobulin heavy variable 1-12 (pseudogene) [Source:HGNC Symbol;Acc:HGNC:5546]	IG V pseudogene	2.29	1.09	16.81	0.00	0.01
ENSG00000211662	IGLV3-21	immunoglobulin lambda variable 3-21 [Source:HGNC Symbol;Acc:HGNC:5905]	IG V gene	1.99	9.68	11.78	0.00	0.03
ENSG00000148468	FAM171A1	family with sequence similarity 171 member A1 [Source:HGNC Symbol;Acc:HGNC:23522]	protein coding	1.95	0.48	11.46	0.00	0.04
ENSG00000211653	IGLV1-40	immunoglobulin lambda variable 1-40 [Source:HGNC Symbol;Acc:HGNC:5877]	IG V gene	1.74	8.24	21.63	0.00	0.00
ENSG00000211670	IGLV3-9	immunoglobulin lambda variable 3-9 (gene/pseudogene) [Source:HGNC Symbol;Acc:HGNC:5918]	IG V gene	1.73	6.23	15.45	0.00	0.01
ENSG00000115884	SDC1	syndecan 1 [Source:HGNC Symbol;Acc:HGNC:10658]	protein coding	1.69	4.06	57.81	0.00	0.00
ENSG00000088340	FER1L4	fer-1 like family member 4, pseudogene [Source:HGNC Symbol;Acc:HGNC:15801]	transcribed unitary pseudogene	1.68	2.13	27.53	0.00	0.00
ENSG00000154277	UCHL1	ubiquitin C-terminal hydrolase L1 [Source:HGNC Symbol;Acc:HGNC:12513]	protein coding	1.67	1.37	16.22	0.00	0.01
ENSG00000113140	SPARC	secreted protein acidic and cysteine rich [Source:HGNC Symbol;Acc:HGNC:11219]	protein coding	1.67	1.28	17.84	0.00	0.00
ENSG00000211896	IGHG1	immunoglobulin heavy constant gamma 1 (G1m marker) [Source:HGNC Symbol;Acc:HGNC:5525]	IG C gene	1.65	12.17	34.24	0.00	0.00
ENSG00000211645	IGLV1-50	immunoglobulin lambda variable 1-50 (non-functional) [Source:HGNC Symbol;Acc:HGNC:5881]	IG V gene	1.61	1.40	11.58	0.00	0.04
ENSG00000253755	IGHGP	immunoglobulin heavy constant gamma P (non-functional) [Source:HGNC Symbol;Acc:HGNC:5529]	IG C pseudogene	1.49	7.19	31.44	0.00	0.00
ENSG00000105974	CAV1	caveolin 1 [Source:HGNC Symbol;Acc:HGNC:1527]	protein coding	1.48	3.85	32.78	0.00	0.00
ENSG00000211677	IGLC2	immunoglobulin lambda constant 2 [Source:HGNC Symbol;Acc:HGNC:5856]	IG C gene	1.31	10.64	18.10	0.00	0.00

Ensembl Gene ID	Ensembl Gene Name	Ensembl Gene Description	Gene Type	Log_2 Fold Change (Day 8 vs. pre-treatment)	Average Log_2 CPM	Likelihood Ratio Test Statistic	P-Value	FDR Adjusted P-Value
ENSG00000211938	IGHV3-7	immunoglobulin heavy variable 3-7 [Source:HGNC Symbol;Acc:HGNC:5620]	IG V gene	1.31	8.14	24.24	0.00	0.00
ENSG00000282600			IG V pseudogene	1.29	2.30	18.76	0.00	0.00
ENSG00000162676	GFI1	growth factor independent 1 transcriptional repressor [Source:HGNC Symbol;Acc:HGNC:4237]	protein coding	1.27	2.42	13.59	0.00	0.02
ENSG00000163053	SLC16A14	solute carrier family 16 member 14 [Source:HGNC Symbol;Acc:HGNC:26417]	protein coding	1.23	2.18	15.44	0.00	0.01
ENSG00000224373	IGHV4-59	immunoglobulin heavy variable 4-59 [Source:HGNC Symbol;Acc:HGNC:5654]	IG V gene	1.23	6.63	55.25	0.00	0.00
ENSG00000121807	CCR2	C-C motif chemokine receptor 2 [Source:HGNC Symbol;Acc:HGNC:1603]	protein coding	1.20	5.72	82.34	0.00	0.00
ENSG00000186810	CXCR3	C-X-C motif chemokine receptor 3 [Source:HGNC Symbol;Acc:HGNC:4540]	protein coding	1.17	4.10	37.80	0.00	0.00
ENSG00000123989	CHPF	chondroitin polymerizing factor [Source:HGNC Symbol;Acc:HGNC:24291]	protein coding	1.14	4.95	22.08	0.00	0.00
ENSG00000248571			antisense	1.11	1.78	14.60	0.00	0.01
ENSG00000211673	IGLV3-1	immunoglobulin lambda variable 3-1 [Source:HGNC Symbol;Acc:HGNC:5896]	IG V gene	1.08	8.22	17.78	0.00	0.00
ENSG00000075420	FNDC3B	fibronectin type III domain containing 3B [Source:HGNC Symbol;Acc:HGNC:24670]	protein coding	1.04	5.34	27.28	0.00	0.00
ENSG00000183010	PYCR1	pyrroline-5-carboxylate reductase 1 [Source:HGNC Symbol;Acc:HGNC:9721]	protein coding	1.03	2.69	16.57	0.00	0.01
ENSG00000100219	XBP1	X-box binding protein 1 [Source:HGNC Symbol;Acc:HGNC:12801]	protein coding	1.01	8.34	34.37	0.00	0.00
ENSG00000184232	OAF	out at first homolog [Source:HGNC Symbol;Acc:HGNC:28752]	protein coding	1.00	2.35	14.21	0.00	0.01
ENSG00000180535	BHLHA15	basic helix-loop-helix family member a15 [Source:HGNC Symbol;Acc:HGNC:22265]	protein coding	0.97	3.30	19.44	0.00	0.00
ENSG00000173578	XCR1	X-C motif chemokine receptor 1 [Source:HGNC Symbol;Acc:HGNC:1625]	protein coding	0.96	2.51	11.64	0.00	0.04
ENSG00000178445	GLDC	glycine decarboxylase [Source:HGNC Symbol;Acc:HGNC:4313]	protein coding	0.96	4.90	23.14	0.00	0.00
ENSG00000211972	IGHV3-66	immunoglobulin heavy variable 3-66 [Source:HGNC Symbol;Acc:HGNC:5619]	IG V gene	0.96	4.62	13.04	0.00	0.02
ENSG00000203914	HSP90B3P	heat shock protein 90 beta family member 3, pseudogene [Source:HGNC Symbol;Acc:HGNC:12100]	processed pseudogene	0.96	2.68	18.22	0.00	0.00
ENSG00000074842	MYDGF	myeloid derived growth factor [Source:HGNC Symbol;Acc:HGNC:16948]	protein coding	0.95	6.42	23.39	0.00	0.00
ENSG00000198794	SCAMP5	secretory carrier membrane protein 5 [Source:HGNC Symbol;Acc:HGNC:30386]	protein coding	0.94	3.46	25.25	0.00	0.00
ENSG00000065485	PDIA5	protein disulfide isomerase family A member 5 [Source:HGNC Symbol;Acc:HGNC:24811]	protein coding	0.94	4.20	19.14	0.00	0.00
ENSG00000102471	NDFIP2	Nedd4 family interacting protein 2 [Source:HGNC Symbol;Acc:HGNC:18537]	protein coding	0.93	1.98	10.92	0.00	0.04

Ensembl Gene ID	Ensembl Gene Name	Ensembl Gene Description	Gene Type	Log_2 Fold Change (Day 8 vs. pre-treatment)	Average Log_2 CPM	Likelihood Ratio Test Statistic	P-Value	FDR Adjusted P-Value
ENSG00000143603	KCNN3	potassium calcium-activated channel subfamily N member 3 [Source:HGNC Symbol;Acc:HGNC:6292]	protein coding	0.92	2.96	12.47	0.00	0.03
ENSG00000211952	IGHV4-28	immunoglobulin heavy variable 4-28 [Source:HGNC Symbol;Acc:HGNC:5645]	IG V gene	0.91	3.69	14.50	0.00	0.01
ENSG00000167861	HID1	HID1 domain containing [Source:HGNC Symbol;Acc:HGNC:15736]	protein coding	0.91	4.16	15.72	0.00	0.01
ENSG00000259706	HSP90B2P	heat shock protein 90 beta family member 2, pseudogene [Source:HGNC Symbol;Acc:HGNC:12099]	processed pseudogene	0.91	4.91	27.79	0.00	0.00
ENSG00000123131	PRDX4	peroxiredoxin 4 [Source:HGNC Symbol;Acc:HGNC:17169]	protein coding	0.90	5.15	22.31	0.00	0.00
ENSG00000026751	SLAMF7	SLAM family member 7 [Source:HGNC Symbol;Acc:HGNC:21394]	protein coding	0.89	7.45	24.93	0.00	0.00
ENSG00000025039	RRAGD	Ras related GTP binding D [Source:HGNC Symbol;Acc:HGNC:19903]	protein coding	0.89	2.75	13.46	0.00	0.02
ENSG00000178127	NDUFV2	NADH:ubiquinone oxidoreductase core subunit V2 [Source:HGNC Symbol;Acc:HGNC:7717]	protein coding	-0.87	2.42	15.87	0.00	0.01
ENSG00000168209	DDIT4	DNA damage inducible transcript 4 [Source:HGNC Symbol;Acc:HGNC:24944]	protein coding	-0.86	4.56	32.30	0.00	0.00
ENSG00000166598	HSP90B1	heat shock protein 90 beta family member 1 [Source:HGNC Symbol;Acc:HGNC:12028]	protein coding	0.86	10.49	20.53	0.00	0.00
ENSG00000242766	IGKV1D-17	immunoglobulin kappa variable 1D-17 [Source:HGNC Symbol;Acc:HGNC:5749]	IG V gene	0.85	3.19	11.15	0.00	0.04
ENSG00000107104	KANK1	KN motif and ankyrin repeat domains 1 [Source:HGNC Symbol;Acc:HGNC:19309]	protein coding	0.85	2.80	17.62	0.00	0.00
ENSG00000132465	JCHAIN	joining chain of multimeric IgA and IgM [Source:HGNC Symbol;Acc:HGNC:5713]	protein coding	0.83	11.54	18.33	0.00	0.00
ENSG00000135916	ITM2C	integral membrane protein 2C [Source:HGNC Symbol;Acc:HGNC:6175]	protein coding	0.83	8.62	20.73	0.00	0.00
ENSG00000232176			processed pseudogene	-0.82	3.77	26.63	0.00	0.00
ENSG00000155660	PDIA4	protein disulfide isomerase family A member 4 [Source:HGNC Symbol;Acc:HGNC:30167]	protein coding	0.82	8.50	18.69	0.00	0.00
ENSG00000118985	ELL2	elongation factor for RNA polymerase II 2 [Source:HGNC Symbol;Acc:HGNC:17064]	protein coding	0.81	7.02	23.00	0.00	0.00
ENSG00000070081	NUCB2	nucleobindin 2 [Source:HGNC Symbol;Acc:HGNC:8044]	protein coding	0.81	5.51	22.32	0.00	0.00
ENSG00000185164	NOMO2	NODAL modulator 2 [Source:HGNC Symbol;Acc:HGNC:22652]	protein coding	0.80	4.19	15.42	0.00	0.01
ENSG00000254395	IGHV4-55	immunoglobulin heavy variable 4-55 (pseudogene) [Source:HGNC Symbol;Acc:HGNC:5653]	IG V pseudogene	0.80	4.02	11.02	0.00	0.04
ENSG00000181800	CELF2-AS1	CELF2 antisense RNA 1 [Source:HGNC Symbol;Acc:HGNC:23515]	antisense	-0.80	3.06	12.21	0.00	0.03
ENSG00000004468	CD38	CD38 molecule [Source:HGNC Symbol;Acc:HGNC:1667]	protein coding	0.78	7.50	27.02	0.00	0.00

Ensembl Gene ID	Ensembl Gene Name	Ensembl Gene Description	Gene Type	Log_2 Fold Change (Day 8 vs. pre-treatment)	Average Log_2 CPM	Likelihood Ratio Test Statistic	P-Value	FDR Adjusted P-Value
ENSG00000137563	GGH	gamma-glutamyl hydrolase [Source:HGNC Symbol;Acc:HGNC:4248]	protein coding	0.77	3.50	13.77	0.00	0.02
ENSG00000170476	MZB1	marginal zone B and B1 cell specific protein [Source:HGNC Symbol;Acc:HGNC:30125]	protein coding	0.77	8.34	20.64	0.00	0.00
ENSG00000103257	SLC7A5	solute carrier family 7 member 5 [Source:HGNC Symbol;Acc:HGNC:11063]	protein coding	0.77	5.30	14.15	0.00	0.01
ENSG00000211964	IGHV3-48	immunoglobulin heavy variable 3-48 [Source:HGNC Symbol;Acc:HGNC:5606]	IG V gene	0.77	7.33	17.24	0.00	0.00
ENSG00000118515	SGK1	serum/glucocorticoid regulated kinase 1 [Source:HGNC Symbol;Acc:HGNC:10810]	protein coding	0.76	4.30	26.58	0.00	0.00
ENSG00000048462	TNFRSF17	TNF receptor superfamily member 17 [Source:HGNC Symbol;Acc:HGNC:11913]	protein coding	0.76	6.28	26.80	0.00	0.00
ENSG00000168268	NT5DC2	5'-nucleotidase domain containing 2 [Source:HGNC Symbol;Acc:HGNC:25717]	protein coding	0.76	5.42	16.47	0.00	0.01
ENSG00000111291	GPRC5D	G protein-coupled receptor class C group 5 member D [Source:HGNC Symbol;Acc:HGNC:13310]	protein coding	0.75	3.85	15.54	0.00	0.01
ENSG00000145050	MANF	mesencephalic astrocyte derived neurotrophic factor [Source:HGNC Symbol;Acc:HGNC:15461]	protein coding	0.74	6.23	23.17	0.00	0.00
ENSG00000131871	SELENOS	selenoprotein S [Source:HGNC Symbol;Acc:HGNC:30396]	protein coding	0.73	5.44	26.75	0.00	0.00
ENSG00000134285	FKBP11	FK506 binding protein 11 [Source:HGNC Symbol;Acc:HGNC:18624]	protein coding	0.73	6.15	14.98	0.00	0.01
ENSG00000241666			antisense	-0.73	2.96	21.00	0.00	0.00
ENSG00000173334	TRIB1	tribbles pseudokinase 1 [Source:HGNC Symbol;Acc:HGNC:16891]	protein coding	0.73	5.80	14.47	0.00	0.01
ENSG00000079931	MOXD1	monooxygenase DBH like 1 [Source:HGNC Symbol;Acc:HGNC:21063]	protein coding	0.72	3.65	22.15	0.00	0.00
ENSG00000068912	ERLEC1	endoplasmic reticulum lectin 1 [Source:HGNC Symbol;Acc:HGNC:25222]	protein coding	0.72	6.51	21.62	0.00	0.00
ENSG00000198855	FICD	FIC domain containing [Source:HGNC Symbol;Acc:HGNC:18416]	protein coding	0.71	3.18	13.12	0.00	0.02
ENSG00000173848	NET1	neuroepithelial cell transforming 1 [Source:HGNC Symbol;Acc:HGNC:14592]	protein coding	0.71	4.15	18.99	0.00	0.00
ENSG00000143942	CHAC2	ChaC cation transport regulator homolog 2 [Source:HGNC Symbol;Acc:HGNC:32363]	protein coding	0.71	3.05	14.45	0.00	0.01
ENSG00000128228	SDF2L1	stromal cell derived factor 2 like 1 [Source:HGNC Symbol;Acc:HGNC:10676]	protein coding	0.70	4.82	14.31	0.00	0.01
ENSG00000128590	DNAJB9	DnaJ heat shock protein family (Hsp40) member B9 [Source:HGNC Symbol;Acc:HGNC:6968]	protein coding	0.70	5.18	13.44	0.00	0.02
ENSG00000111885	MAN1A1	mannosidase alpha class 1A member 1 [Source:HGNC Symbol;Acc:HGNC:6821]	protein coding	0.70	7.89	15.59	0.00	0.01
ENSG00000074695	LMAN1	lectin, mannose binding 1 [Source:HGNC Symbol;Acc:HGNC:6631]	protein coding	0.69	7.77	19.17	0.00	0.00
ENSG00000196189	SEMA4A	semaphorin 4A [Source:HGNC Symbol;Acc:HGNC:10729]	protein coding	0.69	6.55	16.71	0.00	0.01

Ensembl Gene ID	Ensembl Gene Name	Ensembl Gene Description	Gene Type	Log_2 Fold Change (Day 8 vs. pre-treatment)	Average Log_2 CPM	Likelihood Ratio Test Statistic	P-Value	FDR Adjusted P-Value
ENSG00000136026	CKAP4	cytoskeleton associated protein 4 [Source:HGNC Symbol;Acc:HGNC:16991]	protein coding	0.69	6.51	30.28	0.00	0.00
ENSG00000239264	TXNDC5	thioredoxin domain containing 5 [Source:HGNC Symbol;Acc:HGNC:21073]	protein coding	0.69	5.56	14.99	0.00	0.01
ENSG00000241351	IGKV3-11	immunoglobulin kappa variable 3-11 [Source:HGNC Symbol;Acc:HGNC:5815]	IG V gene	0.68	7.38	43.83	0.00	0.00
ENSG00000044574	HSPA5	heat shock protein family A (Hsp70) member 5 [Source:HGNC Symbol;Acc:HGNC:5238]	protein coding	0.68	9.00	16.14	0.00	0.01
ENSG00000111424	VDR	vitamin D (1,25- dihydroxyvitamin D3) receptor [Source:HGNC Symbol;Acc:HGNC:12679]	protein coding	0.68	3.39	11.34	0.00	0.04
ENSG00000211892	IGHG4	immunoglobulin heavy constant gamma 4 (G4m marker) [Source:HGNC Symbol;Acc:HGNC:5528]	IG C gene	0.68	7.19	21.94	0.00	0.00
ENSG00000211599	IGKV5-2	immunoglobulin kappa variable 5-2 [Source:HGNC Symbol;Acc:HGNC:5835]	IG V gene	0.67	4.23	17.41	0.00	0.00
ENSG00000166794	PPIB	peptidylprolyl isomerase B [Source:HGNC Symbol;Acc:HGNC:9255]	protein coding	0.67	7.46	16.42	0.00	0.01
ENSG00000113615	SEC24A	SEC24 homolog A, COPII coat complex component [Source:HGNC Symbol;Acc:HGNC:10703]	protein coding	0.67	6.46	14.62	0.00	0.01
ENSG00000196263	ZNF471	zinc finger protein 471 [Source:HGNC Symbol;Acc:HGNC:23226]	protein coding	-0.66	2.76	13.17	0.00	0.02
ENSG00000169962	TAS1R3	taste 1 receptor member 3 [Source:HGNC Symbol;Acc:HGNC:15661]	protein coding	0.66	3.80	14.00	0.00	0.01
ENSG00000183508	FAM46C	family with sequence similarity 46 member C [Source:HGNC Symbol;Acc:HGNC:24712]	protein coding	0.66	8.38	18.92	0.00	0.00
ENSG00000166562	SEC11C	SEC11 homolog C, signal peptidase complex subunit [Source:HGNC Symbol;Acc:HGNC:23400]	protein coding	0.65	7.17	14.11	0.00	0.01
ENSG00000139193	CD27	CD27 molecule [Source:HGNC Symbol;Acc:HGNC:11922]	protein coding	0.65	5.83	17.35	0.00	0.00
ENSG00000125844	RRBP1	ribosome binding protein 1 [Source:HGNC Symbol;Acc:HGNC:10448]	protein coding	0.64	6.98	21.28	0.00	0.00
ENSG00000150961	SEC24D	SEC24 homolog D, COPII coat complex component [Source:HGNC Symbol;Acc:HGNC:10706]	protein coding	0.64	6.35	20.99	0.00	0.00
ENSG00000118705	RPN2	ribophorin II [Source:HGNC Symbol;Acc:HGNC:10382]	protein coding	0.64	8.18	20.74	0.00	0.00
ENSG00000155304	HSPA13	heat shock protein family A (Hsp70) member 13 [Source:HGNC Symbol;Acc:HGNC:11375]	protein coding	0.63	6.06	17.82	0.00	0.00
ENSG00000074416	MGLL	monoglyceride lipase [Source:HGNC Symbol;Acc:HGNC:17038]	protein coding	0.63	3.62	11.47	0.00	0.04
ENSG00000120725	SIL1	SIL1 nucleotide exchange factor [Source:HGNC Symbol;Acc:HGNC:24624]	protein coding	0.62	4.22	12.17	0.00	0.03
ENSG00000070214	SLC44A1	solute carrier family 44 member 1 [Source:HGNC Symbol;Acc:HGNC:18798]	protein coding	0.62	6.94	18.48	0.00	0.00
ENSG00000153066	TXNDC11	thioredoxin domain containing 11 [Source:HGNC Symbol;Acc:HGNC:28030]	protein coding	0.61	7.51	15.94	0.00	0.01
ENSG00000065308	TRAM2	translocation associated membrane protein 2 [Source:HGNC Symbol;Acc:HGNC:16855]	protein coding	0.61	6.92	18.48	0.00	0.00

Ensembl Gene ID	Ensembl Gene Name	Ensembl Gene Description	Gene Type	Log_2 Fold Change (Day 8 vs. pre-treatment)	Average Log_2 CPM	Likelihood Ratio Test Statistic	P-Value	FDR Adjusted P-Value
ENSG00000108829	LRRC59	leucine rich repeat containing 59 [Source:HGNC Symbol;Acc:HGNC:28817]	protein coding	0.61	6.41	20.70	0.00	0.00
ENSG00000103226	NOMO3	NODAL modulator 3 [Source:HGNC Symbol;Acc:HGNC:25242]	protein coding	0.60	3.02	11.34	0.00	0.04
ENSG00000123352	SPATS2	spermatogenesis associated serine rich 2 [Source:HGNC Symbol;Acc:HGNC:18650]	protein coding	0.60	5.47	21.06	0.00	0.00
ENSG00000083444	PLOD1	procollagen-lysine,2-oxoglutarate 5-dioxygenase 1 [Source:HGNC Symbol;Acc:HGNC:9081]	protein coding	0.60	4.30	14.09	0.00	0.01
ENSG00000106803	SEC61B	Sec61 translocon beta subunit [Source:HGNC Symbol;Acc:HGNC:16993]	protein coding	0.60	5.97	17.94	0.00	0.00
ENSG00000279192	PWAR5	Prader Willi/Angelman region RNA 5 [Source:HGNC Symbol;Acc:HGNC:30090]	TEC	-0.60	3.27	10.73	0.00	0.05
ENSG00000100154	TTC28	tetratricopeptide repeat domain 28 [Source:HGNC Symbol;Acc:HGNC:29179]	protein coding	-0.59	3.05	10.81	0.00	0.04
ENSG00000134910	STT3A	STT3A, catalytic subunit of the oligosaccharyltransferase complex [Source:HGNC Symbol;Acc:HGNC:6172]	protein coding	0.59	7.13	21.84	0.00	0.00
ENSG00000099337	KCNK6	potassium two pore domain channel subfamily K member 6 [Source:HGNC Symbol;Acc:HGNC:6281]	protein coding	0.59	5.11	16.97	0.00	0.01
ENSG00000181722	ZBTB20	zinc finger and BTB domain containing 20 [Source:HGNC Symbol;Acc:HGNC:13503]	protein coding	-0.59	5.00	18.69	0.00	0.00
ENSG00000113621	TXNDC15	thioredoxin domain containing 15 [Source:HGNC Symbol;Acc:HGNC:20652]	protein coding	0.59	5.49	25.95	0.00	0.00
ENSG00000080546	SESN1	sestrin 1 [Source:HGNC Symbol;Acc:HGNC:21595]	protein coding	-0.59	6.66	43.86	0.00	0.00
ENSG00000100342	APOL1	apolipoprotein L1 [Source:HGNC Symbol;Acc:HGNC:618]	protein coding	0.59	4.58	20.57	0.00	0.00
ENSG00000017483	SLC38A5	solute carrier family 38 member 5 [Source:HGNC Symbol;Acc:HGNC:18070]	protein coding	0.59	4.91	13.22	0.00	0.02

Table 14: Genes differentially expressed at Day 8 compared to pre-treatment (B Cells, Trivalent Influenza Vaccine). Sorted by descending absolute log_2 fold change (Day 8 vs. pre-treatment). Gene model summaries and annotations are based on Ensembl Version 87.

Ensembl Gene ID	Ensembl Gene Name	Ensembl Gene Description	Gene Type	Log_2 Fold Change (Day 9 vs. pre-treatment)	Average Log_2 CPM	Likelihood Ratio Test Statistic	P-Value	FDR Adjusted P-Value
ENSG00000254176	IGHV3-75	immunoglobulin heavy variable 3-75 (pseudogene) [Source:HGNC Symbol;Acc:HGNC:5625]	IG V pseudogene	6.51	-0.25	18.08	0.00	0.02
ENSG00000158578	ALAS2	5'-aminolevulinate synthase 2 [Source:HGNC Symbol;Acc:HGNC:397]	protein coding	2.22	2.43	56.22	0.00	0.00
ENSG00000211670	IGLV3-9	immunoglobulin lambda variable 3-9 (gene/pseudogene) [Source:HGNC Symbol;Acc:HGNC:5918]	IG V gene	1.57	5.49	17.24	0.00	0.02
ENSG00000244734	HBB	hemoglobin subunit beta [Source:HGNC Symbol;Acc:HGNC:4827]	protein coding	1.55	9.71	51.73	0.00	0.00

Ensembl Gene ID	Ensembl Gene Name	Ensembl Gene Description	Gene Type	Log_2 Fold Change (Day 9 vs. pre-treatment)	Average Log_2 CPM	Likelihood Ratio Test Statistic	P-Value	FDR Adjusted P-Value
ENSG00000206172	HBA1	hemoglobin subunit alpha 1 [Source:HGNC Symbol;Acc:HGNC:4823]	protein coding	1.43	4.95	35.36	0.00	0.00
ENSG00000188536	HBA2	hemoglobin subunit alpha 2 [Source:HGNC Symbol;Acc:HGNC:4824]	protein coding	1.42	6.22	46.68	0.00	0.00
ENSG00000211653	IGLV1-40	immunoglobulin lambda variable 1-40 [Source:HGNC Symbol;Acc:HGNC:5877]	IG V gene	1.39	7.83	15.61	0.00	0.05
ENSG00000115884	SDC1	syndecan 1 [Source:HGNC Symbol;Acc:HGNC:10658]	protein coding	1.38	3.72	26.24	0.00	0.00
ENSG00000224373	IGHV4-59	immunoglobulin heavy variable 4-59 [Source:HGNC Symbol;Acc:HGNC:5654]	IG V gene	0.97	6.44	24.41	0.00	0.00
ENSG00000121807	CCR2	C-C motif chemokine receptor 2 [Source:HGNC Symbol;Acc:HGNC:1603]	protein coding	0.83	5.43	43.92	0.00	0.00
ENSG00000186810	CXCR3	C-X-C motif chemokine receptor 3 [Source:HGNC Symbol;Acc:HGNC:4540]	protein coding	0.79	3.82	20.45	0.00	0.01
ENSG00000211599	IGKV5-2	immunoglobulin kappa variable 5-2 [Source:HGNC Symbol;Acc:HGNC:5835]	IG V gene	0.78	4.42	21.46	0.00	0.00
ENSG00000211673	IGLV3-1	immunoglobulin lambda variable 3-1 [Source:HGNC Symbol;Acc:HGNC:5896]	IG V gene	0.71	7.79	18.28	0.00	0.02
ENSG00000079931	MOXD1	monooxygenase DBH like 1 [Source:HGNC Symbol;Acc:HGNC:21063]	protein coding	0.60	3.55	16.91	0.00	0.03

Table 15: Genes differentially expressed at Day 9 compared to pre-treatment (B Cells, Trivalent Influenza Vaccine). Sorted by descending absolute log_2 fold change (Day 9 vs. pre-treatment). Gene model summaries and annotations are based on Ensembl Version 87.

Ensembl Gene ID	Ensembl Gene Name	Ensembl Gene Description	Gene Type	Log_2 Fold Change (Day 10 vs. pre-treatment)	Average Log_2 CPM	Likelihood Ratio Test Statistic	P-Value	FDR Adjusted P-Value
ENSG00000211670	IGLV3-9	immunoglobulin lambda variable 3-9 (gene/pseudogene) [Source:HGNC Symbol;Acc:HGNC:5918]	IG V gene	1.55	5.12	27.34	0.00	0.00
ENSG00000101439	CST3	cystatin C [Source:HGNC Symbol;Acc:HGNC:2475]	protein coding	-0.87	3.47	20.32	0.00	0.02
ENSG00000211599	IGKV5-2	immunoglobulin kappa variable 5-2 [Source:HGNC Symbol;Acc:HGNC:5835]	IG V gene	0.84	4.55	22.26	0.00	0.01

Table 16: Genes differentially expressed at Day 10 compared to pre-treatment (B Cells, Trivalent Influenza Vaccine). Sorted by descending absolute log_2 fold change (Day 10 vs. pre-treatment). Gene model summaries and annotations are based on Ensembl Version 87.

Ensembl Gene ID	Ensembl Gene Name	Ensembl Gene Description	Gene Type	Log_2 Fold Change (Day 1 vs. pre-treatment)	Average Log_2 CPM	Likelihood Ratio Test Statistic	P-Value	FDR Adjusted P-Value
ENSG00000100336	APOL4	apolipoprotein L4 [Source:HGNC Symbol;Acc:HGNC:14867]	protein coding	2.57	1.62	43.61	0.00	0.00

Ensembl Gene ID	Ensembl Gene Name	Ensembl Gene Description	Gene Type	Log_2 Fold Change (Day 1 vs. pre-treatment)	Average Log_2 CPM	Likelihood Ratio Test Statistic	P-Value	FDR Adjusted P-Value
ENSG00000149131	SERPING1	serpin family G member 1 [Source:HGNC Symbol;Acc:HGNC:1228]	protein coding	1.83	3.81	51.22	0.00	0.00
ENSG00000152766	ANKRD22	ankyrin repeat domain 22 [Source:HGNC Symbol;Acc:HGNC:28321]	protein coding	1.79	2.54	30.20	0.00	0.00
ENSG00000123689	G0S2	G0/G1 switch 2 [Source:HGNC Symbol;Acc:HGNC:30229]	protein coding	-1.61	1.10	13.51	0.00	0.01
ENSG00000166278	C2	complement C2 [Source:HGNC Symbol;Acc:HGNC:1248]	protein coding	1.57	1.53	26.20	0.00	0.00
ENSG00000198019	FCGR1B	Fc fragment of IgG receptor Ib [Source:HGNC Symbol;Acc:HGNC:3614]	protein coding	1.54	1.88	31.19	0.00	0.00
ENSG00000168062	BATF2	basic leucine zipper ATF-like transcription factor 2 [Source:HGNC Symbol;Acc:HGNC:25163]	protein coding	1.40	2.13	26.84	0.00	0.00
ENSG00000183762	KREMEN1	kringle containing transmembrane protein 1 [Source:HGNC Symbol;Acc:HGNC:17550]	protein coding	1.39	2.19	27.23	0.00	0.00
ENSG00000150337	FCGR1A	Fc fragment of IgG receptor Ia [Source:HGNC Symbol;Acc:HGNC:3613]	protein coding	1.35	2.99	41.24	0.00	0.00
ENSG00000169245	CXCL10	C-X-C motif chemokine ligand 10 [Source:HGNC Symbol;Acc:HGNC:10637]	protein coding	1.33	2.72	15.25	0.00	0.00
ENSG00000272821			antisense	1.21	3.76	58.04	0.00	0.00
ENSG00000174705	SH3PXD2B	SH3 and PX domains 2B [Source:HGNC Symbol;Acc:HGNC:29242]	protein coding	1.20	2.02	12.73	0.00	0.01
ENSG00000225492	GBP1P1	guanylate binding protein 1 pseudogene 1 [Source:HGNC Symbol;Acc:HGNC:39561]	transcribed unprocessed pseudogene	1.19	1.74	8.77	0.00	0.05
ENSG00000205846	CLEC6A	C-type lectin domain family 6 member A [Source:HGNC Symbol;Acc:HGNC:14556]	protein coding	1.18	1.88	9.60	0.00	0.04
ENSG00000177989	ODF3B	outer dense fiber of sperm tails 3B [Source:HGNC Symbol;Acc:HGNC:34388]	protein coding	1.16	5.52	37.02	0.00	0.00
ENSG00000130489	SCO2	SCO2, cytochrome c oxidase assembly protein [Source:HGNC Symbol;Acc:HGNC:10604]	protein coding	1.04	4.48	53.67	0.00	0.00
ENSG00000123700	KCNJ2	potassium voltage-gated channel subfamily J member 2 [Source:HGNC Symbol;Acc:HGNC:6263]	protein coding	1.04	3.82	19.34	0.00	0.00
ENSG00000025708	TYMP	thymidine phosphorylase [Source:HGNC Symbol;Acc:HGNC:3148]	protein coding	1.01	7.97	44.15	0.00	0.00
ENSG00000234389			sense intronic	-0.97	2.31	23.22	0.00	0.00
ENSG00000162512	SDC3	syndecan 3 [Source:HGNC Symbol;Acc:HGNC:10660]	protein coding	0.97	2.92	21.90	0.00	0.00
ENSG00000125538	IL1B	interleukin 1 beta [Source:HGNC Symbol;Acc:HGNC:5992]	protein coding	0.96	3.79	19.58	0.00	0.00
ENSG00000111181	SLC6A12	solute carrier family 6 member 12 [Source:HGNC Symbol;Acc:HGNC:11045]	protein coding	0.95	2.79	23.06	0.00	0.00
ENSG00000115415	STAT1	signal transducer and activator of transcription 1 [Source:HGNC Symbol;Acc:HGNC:11362]	protein coding	0.95	9.11	43.00	0.00	0.00

Ensembl Gene ID	Ensembl Gene Name	Ensembl Gene Description	Gene Type	Log_2 Fold Change (Day 1 vs. pre-treatment)	Average Log_2 CPM	Likelihood Ratio Test Statistic	P-Value	FDR Adjusted P-Value
ENSG00000185339	TCN2	transcobalamin 2 [Source:HGNC Symbol;Acc:HGNC:11653]	protein coding	0.94	3.79	26.51	0.00	0.00
ENSG00000141574	SECTM1	secreted and transmembrane 1 [Source:HGNC Symbol;Acc:HGNC:10707]	protein coding	0.94	6.60	40.06	0.00	0.00
ENSG00000117228	GBP1	guanylate binding protein 1 [Source:HGNC Symbol;Acc:HGNC:4182]	protein coding	0.91	7.12	21.74	0.00	0.00
ENSG00000002549	LAP3	leucine aminopeptidase 3 [Source:HGNC Symbol;Acc:HGNC:18449]	protein coding	0.90	6.81	32.78	0.00	0.00
ENSG00000119922	IFIT2	interferon induced protein with tetratricopeptide repeats 2 [Source:HGNC Symbol;Acc:HGNC:5409]	protein coding	0.90	5.98	27.36	0.00	0.00
ENSG00000149798	CDC42EP2	CDC42 effector protein 2 [Source:HGNC Symbol;Acc:HGNC:16263]	protein coding	0.87	3.64	32.94	0.00	0.00
ENSG00000149557	FEZ1	fasciculation and elongation protein zeta 1 [Source:HGNC Symbol;Acc:HGNC:3659]	protein coding	-0.87	2.70	18.47	0.00	0.00
ENSG00000182487	NCF1B	neutrophil cytosolic factor 1B pseudogene [Source:HGNC Symbol;Acc:HGNC:32522]	transcribed unprocessed pseudogene	0.86	4.24	43.16	0.00	0.00
ENSG00000250138			unprocessed pseudogene	0.85	2.63	18.44	0.00	0.00
ENSG00000140105	WARS	tryptophanyl-tRNA synthetase [Source:HGNC Symbol;Acc:HGNC:12729]	protein coding	0.85	8.50	35.54	0.00	0.00
ENSG00000260401			sense overlapping	0.83	2.86	18.70	0.00	0.00
ENSG00000119917	IFIT3	interferon induced protein with tetratricopeptide repeats 3 [Source:HGNC Symbol;Acc:HGNC:5411]	protein coding	0.82	5.74	18.55	0.00	0.00
ENSG00000179331	RAB39A	RAB39A, member RAS oncogene family [Source:HGNC Symbol;Acc:HGNC:16521]	protein coding	0.82	2.34	16.97	0.00	0.00
ENSG00000158714	SLAMF8	SLAM family member 8 [Source:HGNC Symbol;Acc:HGNC:21391]	protein coding	0.82	3.40	12.21	0.00	0.01
ENSG00000135636	DYSF	dysferlin [Source:HGNC Symbol;Acc:HGNC:3097]	protein coding	0.81	6.79	23.39	0.00	0.00
ENSG00000088827	SIGLEC1	sialic acid binding Ig like lectin 1 [Source:HGNC Symbol;Acc:HGNC:11127]	protein coding	0.81	5.24	29.90	0.00	0.00
ENSG00000020577	SAMD4A	sterile alpha motif domain containing 4A [Source:HGNC Symbol;Acc:HGNC:23023]	protein coding	0.81	3.55	16.26	0.00	0.00
ENSG00000113494	PRLR	prolactin receptor [Source:HGNC Symbol;Acc:HGNC:9446]	protein coding	0.79	2.15	13.26	0.00	0.01
ENSG00000165178	NCF1C	neutrophil cytosolic factor 1C pseudogene [Source:HGNC Symbol;Acc:HGNC:32523]	unprocessed pseudogene	0.79	5.03	46.78	0.00	0.00
ENSG00000232810	TNF	tumor necrosis factor [Source:HGNC Symbol;Acc:HGNC:11892]	protein coding	0.78	2.72	21.52	0.00	0.00

Ensembl Gene ID	Ensembl Gene Name	Ensembl Gene Description	Gene Type	Log_2 Fold Change (Day 1 vs. pre-treatment)	Average Log_2 CPM	Likelihood Ratio Test Statistic	P-Value	FDR Adjusted P-Value
ENSG00000019169	MARCO	macrophage receptor with collagenous structure [Source:HGNC Symbol;Acc:HGNC:6895]	protein coding	0.78	4.17	25.58	0.00	0.00
ENSG00000158517	NCF1	neutrophil cytosolic factor 1 [Source:HGNC Symbol;Acc:HGNC:7660]	protein coding	0.78	5.71	30.84	0.00	0.00
ENSG00000171049	FPR2	formyl peptide receptor 2 [Source:HGNC Symbol;Acc:HGNC:3827]	protein coding	0.77	5.80	18.94	0.00	0.00
ENSG00000125148	MT2A	metallothionein 2A [Source:HGNC Symbol;Acc:HGNC:7406]	protein coding	0.76	3.80	20.08	0.00	0.00
ENSG00000134755	DSC2	desmocollin 2 [Source:HGNC Symbol;Acc:HGNC:3036]	protein coding	0.76	4.12	30.41	0.00	0.00
ENSG00000135604	STX11	syntaxin 11 [Source:HGNC Symbol;Acc:HGNC:11429]	protein coding	0.76	6.17	38.29	0.00	0.00
ENSG00000162614	NEXN	nexilin F-actin binding protein [Source:HGNC Symbol;Acc:HGNC:29557]	protein coding	0.76	2.94	14.37	0.00	0.01
ENSG00000168389	MFSD2A	major facilitator superfamily domain containing 2A [Source:HGNC Symbol;Acc:HGNC:25897]	protein coding	0.75	2.89	15.86	0.00	0.00
ENSG00000169136	ATF5	activating transcription factor 5 [Source:HGNC Symbol;Acc:HGNC:790]	protein coding	0.74	4.31	20.23	0.00	0.00
ENSG00000171451	DSEL	dermatan sulfate epimerase-like [Source:HGNC Symbol;Acc:HGNC:18144]	protein coding	-0.74	2.17	9.15	0.00	0.04
ENSG00000170962	PDGFD	platelet derived growth factor D [Source:HGNC Symbol;Acc:HGNC:30620]	protein coding	-0.74	3.78	23.66	0.00	0.00
ENSG00000186049	KRT73	keratin 73 [Source:HGNC Symbol;Acc:HGNC:28928]	protein coding	-0.73	2.29	9.08	0.00	0.04
ENSG00000139832	RAB20	RAB20, member RAS oncogene family [Source:HGNC Symbol;Acc:HGNC:18260]	protein coding	0.72	3.45	15.78	0.00	0.00
ENSG00000276070	CCL4L2	C-C motif chemokine ligand 4 like 2 [Source:HGNC Symbol;Acc:HGNC:24066]	protein coding	-0.71	1.99	9.35	0.00	0.04
ENSG00000157227	MMP14	matrix metallopeptidase 14 [Source:HGNC Symbol;Acc:HGNC:7160]	protein coding	0.71	2.72	13.28	0.00	0.01
ENSG00000181631	P2RY13	purinergic receptor P2Y13 [Source:HGNC Symbol;Acc:HGNC:4537]	protein coding	0.71	6.90	21.58	0.00	0.00
ENSG00000101335	MYL9	myosin light chain 9 [Source:HGNC Symbol;Acc:HGNC:15754]	protein coding	0.71	3.67	15.75	0.00	0.00
ENSG00000268849	SIGLEC22P	sialic acid binding Ig like lectin 22, pseudogene [Source:HGNC Symbol;Acc:HGNC:15611]	transcribed unprocessed pseudogene	0.71	2.61	9.99	0.00	0.03
ENSG00000128383	APOBEC3A	apolipoprotein B mRNA editing enzyme catalytic subunit 3A [Source:HGNC Symbol;Acc:HGNC:17343]	protein coding	0.71	5.78	22.90	0.00	0.00
ENSG00000173110	HSPA6	heat shock protein family A (Hsp70) member 6 [Source:HGNC Symbol;Acc:HGNC:5239]	protein coding	0.70	4.28	19.73	0.00	0.00
ENSG00000255819	KLRC4-KLRK1	KLRC4-KLRK1 readthrough [Source:HGNC Symbol;Acc:HGNC:48357]	protein coding	-0.70	2.09	8.91	0.00	0.05

Ensembl Gene ID	Ensembl Gene Name	Ensembl Gene Description	Gene Type	Log_2 Fold Change (Day 1 vs. pre-treatment)	Average Log_2 CPM	Likelihood Ratio Test Statistic	P-Value	FDR Adjusted P-Value
ENSG00000271109			lincRNA	-0.70	2.64	15.63	0.00	0.00
ENSG00000125347	IRF1	interferon regulatory factor 1 [Source:HGNC Symbol;Acc:HGNC:6116]	protein coding	0.70	8.00	36.06	0.00	0.00
ENSG00000156587	UBE2L6	ubiquitin conjugating enzyme E2 L6 [Source:HGNC Symbol;Acc:HGNC:12490]	protein coding	0.69	6.89	29.46	0.00	0.00
ENSG00000187608	ISG15	ISG15 ubiquitin-like modifier [Source:HGNC Symbol;Acc:HGNC:4053]	protein coding	0.69	4.39	23.80	0.00	0.00
ENSG00000127951	FGL2	fibrinogen like 2 [Source:HGNC Symbol;Acc:HGNC:3696]	protein coding	0.69	10.13	22.91	0.00	0.00
ENSG00000004809	SLC22A16	solute carrier family 22 member 16 [Source:HGNC Symbol;Acc:HGNC:20302]	protein coding	0.69	2.11	9.88	0.00	0.03
ENSG00000213694	S1PR3	sphingosine-1-phosphate receptor 3 [Source:HGNC Symbol;Acc:HGNC:3167]	protein coding	0.68	4.71	17.86	0.00	0.00
ENSG00000103196	CRISPLD2	cysteine rich secretory protein LCCL domain containing 2 [Source:HGNC Symbol;Acc:HGNC:25248]	protein coding	0.68	6.22	20.96	0.00	0.00
ENSG00000146070	PLA2G7	phospholipase A2 group VII [Source:HGNC Symbol;Acc:HGNC:9040]	protein coding	0.68	4.39	13.11	0.00	0.01
ENSG00000121858	TNFSF10	tumor necrosis factor superfamily member 10 [Source:HGNC Symbol;Acc:HGNC:11925]	protein coding	0.67	7.71	23.30	0.00	0.00
ENSG00000235568	NFAM1	NFAT activating protein with ITAM motif 1 [Source:HGNC Symbol;Acc:HGNC:29872]	protein coding	0.67	7.91	31.36	0.00	0.00
ENSG00000197122	SRC	SRC proto-oncogene, non-receptor tyrosine kinase [Source:HGNC Symbol;Acc:HGNC:11283]	protein coding	0.67	5.43	27.22	0.00	0.00
ENSG00000198814	GK	glycerol kinase [Source:HGNC Symbol;Acc:HGNC:4289]	protein coding	0.67	4.39	18.14	0.00	0.00
ENSG00000134326	CMPK2	cytidine/uridine monophosphate kinase 2 [Source:HGNC Symbol;Acc:HGNC:27015]	protein coding	0.67	4.51	14.58	0.00	0.01
ENSG00000116711	PLA2G4A	phospholipase A2 group IVA [Source:HGNC Symbol;Acc:HGNC:9035]	protein coding	0.66	3.08	15.55	0.00	0.00
ENSG00000188906	LRRK2	leucine rich repeat kinase 2 [Source:HGNC Symbol;Acc:HGNC:18618]	protein coding	0.66	8.22	19.62	0.00	0.00
ENSG00000138496	PARP9	poly(ADP-ribose) polymerase family member 9 [Source:HGNC Symbol;Acc:HGNC:24118]	protein coding	0.66	7.25	31.80	0.00	0.00
ENSG00000165092	ALDH1A1	aldehyde dehydrogenase 1 family member A1 [Source:HGNC Symbol;Acc:HGNC:402]	protein coding	0.66	5.68	15.97	0.00	0.00
ENSG00000204388	HSPA1B	heat shock protein family A (Hsp70) member 1B [Source:HGNC Symbol;Acc:HGNC:5233]	protein coding	0.66	2.84	10.70	0.00	0.02
ENSG00000145685	LHFPL2	lipoma HMGIC fusion partner-like 2 [Source:HGNC Symbol;Acc:HGNC:6588]	protein coding	0.65	4.87	15.20	0.00	0.00
ENSG00000137441	FGFBP2	fibroblast growth factor binding protein 2 [Source:HGNC Symbol;Acc:HGNC:29451]	protein coding	-0.65	6.37	19.40	0.00	0.00
ENSG00000163823	CCR1	C-C motif chemokine receptor 1 [Source:HGNC Symbol;Acc:HGNC:1602]	protein coding	0.65	6.70	23.59	0.00	0.00
ENSG00000180113	TDRD6	tudor domain containing 6 [Source:HGNC Symbol;Acc:HGNC:21339]	protein coding	0.65	2.44	9.13	0.00	0.04

Ensembl Gene ID	Ensembl Gene Name	Ensembl Gene Description	Gene Type	Log_2 Fold Change (Day 1 vs. pre-treatment)	Average Log_2 CPM	Likelihood Ratio Test Statistic	P-Value	FDR Adjusted P-Value
ENSG00000149573	MPZL2	myelin protein zero like 2 [Source:HGNC Symbol;Acc:HGNC:3496]	protein coding	0.65	3.73	24.20	0.00	0.00
ENSG00000143226	FCGR2A	Fc fragment of IgG receptor IIa [Source:HGNC Symbol;Acc:HGNC:3616]	protein coding	0.65	7.13	22.59	0.00	0.00
ENSG00000166527	CLEC4D	C-type lectin domain family 4 member D [Source:HGNC Symbol;Acc:HGNC:14554]	protein coding	0.64	4.46	14.67	0.00	0.01
ENSG00000105967	TFEC	transcription factor EC [Source:HGNC Symbol;Acc:HGNC:11754]	protein coding	0.64	6.36	25.85	0.00	0.00
ENSG00000226091	LINC00937	long intergenic non-protein coding RNA 937 [Source:HGNC Symbol;Acc:HGNC:48629]	lincRNA	0.64	4.19	17.57	0.00	0.00
ENSG00000137959	IFI44L	interferon induced protein 44 like [Source:HGNC Symbol;Acc:HGNC:17817]	protein coding	0.64	6.04	22.51	0.00	0.00
ENSG00000166002	SMCO4	single-pass membrane protein with coiled-coil domains 4 [Source:HGNC Symbol;Acc:HGNC:24810]	protein coding	0.63	4.43	18.27	0.00	0.00
ENSG00000103313	MEFV	Mediterranean fever [Source:HGNC Symbol;Acc:HGNC:6998]	protein coding	0.63	7.09	26.47	0.00	0.00
ENSG00000164023	SGMS2	sphingomyelin synthase 2 [Source:HGNC Symbol;Acc:HGNC:28395]	protein coding	0.63	5.09	16.42	0.00	0.00
ENSG00000185215	TNFAIP2	TNF alpha induced protein 2 [Source:HGNC Symbol;Acc:HGNC:11895]	protein coding	0.63	9.04	24.74	0.00	0.00
ENSG00000139318	DUSP6	dual specificity phosphatase 6 [Source:HGNC Symbol;Acc:HGNC:3072]	protein coding	0.63	8.04	23.09	0.00	0.00
ENSG00000171051	FPR1	formyl peptide receptor 1 [Source:HGNC Symbol;Acc:HGNC:3826]	protein coding	0.63	7.73	21.34	0.00	0.00
ENSG00000229754	CXCR2P1	C-X-C motif chemokine receptor 2 pseudogene 1 [Source:HGNC Symbol;Acc:HGNC:6028]	transcribed unprocessed pseudogene	0.62	4.73	13.47	0.00	0.01
ENSG00000170458	CD14	CD14 molecule [Source:HGNC Symbol;Acc:HGNC:1628]	protein coding	0.62	8.62	20.43	0.00	0.00
ENSG00000180061	TMEM150B	transmembrane protein 150B [Source:HGNC Symbol;Acc:HGNC:34415]	protein coding	0.62	3.55	18.36	0.00	0.00
ENSG00000188820	FAM26F	family with sequence similarity 26 member F [Source:HGNC Symbol;Acc:HGNC:33391]	protein coding	0.62	4.51	9.17	0.00	0.04
ENSG00000111331	OAS3	2'-5'-oligoadenylate synthetase 3 [Source:HGNC Symbol;Acc:HGNC:8088]	protein coding	0.62	7.19	29.97	0.00	0.00
ENSG00000248996			antisense	0.62	2.70	11.45	0.00	0.02
ENSG00000183019	MCEMP1	mast cell expressed membrane protein 1 [Source:HGNC Symbol;Acc:HGNC:27291]	protein coding	0.62	3.97	16.35	0.00	0.00
ENSG00000187840	EIF4EBP1	eukaryotic translation initiation factor 4E binding protein 1 [Source:HGNC Symbol;Acc:HGNC:3288]	protein coding	0.62	3.84	15.39	0.00	0.00
ENSG00000180340	FZD2	frizzled class receptor 2 [Source:HGNC Symbol;Acc:HGNC:4040]	protein coding	0.62	3.00	10.11	0.00	0.03
ENSG00000088826	SMOX	spermine oxidase [Source:HGNC Symbol;Acc:HGNC:15862]	protein coding	0.62	2.93	11.93	0.00	0.02

Ensembl Gene ID	Ensembl Gene Name	Ensembl Gene Description	Gene Type	Log_2 Fold Change (Day 1 vs. pre-treatment)	Average Log_2 CPM	Likelihood Ratio Test Statistic	P-Value	FDR Adjusted P-Value
ENSG00000197057	DTHD1	death domain containing 1 [Source:HGNC Symbol;Acc:HGNC:37261]	protein coding	-0.62	3.90	12.34	0.00	0.01
ENSG00000136689	IL1RN	interleukin 1 receptor antagonist [Source:HGNC Symbol;Acc:HGNC:6000]	protein coding	0.61	4.94	15.26	0.00	0.00
ENSG00000114450	GNB4	G protein subunit beta 4 [Source:HGNC Symbol;Acc:HGNC:20731]	protein coding	0.61	5.90	38.21	0.00	0.00
ENSG00000082397	EPB41L3	erythrocyte membrane protein band 4.1 like 3 [Source:HGNC Symbol;Acc:HGNC:3380]	protein coding	0.61	6.15	25.43	0.00	0.00
ENSG00000167633	KIR3DL1	killer cell immunoglobulin like receptor, three Ig domains and long cytoplasmic tail 1 [Source:HGNC Symbol;Acc:HGNC:6338]	protein coding	-0.61	2.85	9.16	0.00	0.04
ENSG00000140749	IGSF6	immunoglobulin superfamily member 6 [Source:HGNC Symbol;Acc:HGNC:5953]	protein coding	0.61	6.97	17.39	0.00	0.00
ENSG00000205730	ITPRIPL2	inositol 1,4,5-trisphosphate receptor interacting protein like 2 [Source:HGNC Symbol;Acc:HGNC:27257]	protein coding	0.61	4.58	30.15	0.00	0.00
ENSG00000133106	EPSTI1	epithelial stromal interaction 1 [Source:HGNC Symbol;Acc:HGNC:16465]	protein coding	0.61	5.86	27.87	0.00	0.00
ENSG00000185745	IFIT1	interferon induced protein with tetratricopeptide repeats 1 [Source:HGNC Symbol;Acc:HGNC:5407]	protein coding	0.61	4.52	12.62	0.00	0.01
ENSG00000117281	CD160	CD160 molecule [Source:HGNC Symbol;Acc:HGNC:17013]	protein coding	-0.61	4.67	14.87	0.00	0.01
ENSG00000254415	SIGLEC14	sialic acid binding Ig like lectin 14 [Source:HGNC Symbol;Acc:HGNC:32926]	protein coding	0.61	5.92	20.51	0.00	0.00
ENSG00000038945	MSR1	macrophage scavenger receptor 1 [Source:HGNC Symbol;Acc:HGNC:7376]	protein coding	0.60	3.32	15.95	0.00	0.00
ENSG00000186407	CD300E	CD300e molecule [Source:HGNC Symbol;Acc:HGNC:28874]	protein coding	0.60	7.94	21.98	0.00	0.00
ENSG00000128283	CDC42EP1	CDC42 effector protein 1 [Source:HGNC Symbol;Acc:HGNC:17014]	protein coding	0.60	2.96	10.06	0.00	0.03
ENSG00000146592	CREB5	cAMP responsive element binding protein 5 [Source:HGNC Symbol;Acc:HGNC:16844]	protein coding	0.60	6.18	20.79	0.00	0.00
ENSG00000136630	HLX	H2.0 like homeobox [Source:HGNC Symbol;Acc:HGNC:4978]	protein coding	0.60	3.42	15.13	0.00	0.00
ENSG00000110318	CEP126	centrosomal protein 126 [Source:HGNC Symbol;Acc:HGNC:29264]	protein coding	-0.60	3.29	10.88	0.00	0.02
ENSG00000242732	RGAG4	retrotransposon gag domain containing 4 [Source:HGNC Symbol;Acc:HGNC:29430]	protein coding	0.60	3.09	9.07	0.00	0.04
ENSG00000114853	ZBTB47	zinc finger and BTB domain containing 47 [Source:HGNC Symbol;Acc:HGNC:26955]	protein coding	0.59	3.31	15.53	0.00	0.00
ENSG00000187118	CMC1	C-X9-C motif containing 1 [Source:HGNC Symbol;Acc:HGNC:28783]	protein coding	-0.59	5.01	20.52	0.00	0.00
ENSG00000121807	CCR2	C-C motif chemokine receptor 2 [Source:HGNC Symbol;Acc:HGNC:1603]	protein coding	0.59	8.01	23.95	0.00	0.00
ENSG00000115919	KYNU	kynureninase [Source:HGNC Symbol;Acc:HGNC:6469]	protein coding	0.59	5.35	19.51	0.00	0.00

Ensembl Gene ID	Ensembl Gene Name	Ensembl Gene Description	Gene Type	Log_2 Fold Change (Day 1 vs. pre-treatment)	Average Log_2 CPM	Likelihood Ratio Test Statistic	P-Value	FDR Adjusted P-Value
ENSG00000150687	PRSS23	protease, serine 23 [Source:HGNC Symbol;Acc:HGNC:14370]	protein coding	-0.59	4.81	17.05	0.00	0.00
ENSG00000198736	MSRB1	methionine sulfoxide reductase B1 [Source:HGNC Symbol;Acc:HGNC:14133]	protein coding	0.59	5.95	32.48	0.00	0.00
ENSG00000197208	SLC22A4	solute carrier family 22 member 4 [Source:HGNC Symbol;Acc:HGNC:10968]	protein coding	0.59	2.30	9.24	0.00	0.04
ENSG00000143382	ADAMTSL4	ADAMTS like 4 [Source:HGNC Symbol;Acc:HGNC:19706]	protein coding	0.59	5.62	16.41	0.00	0.00

Table 17: Genes differentially expressed at Day 1 compared to pre-treatment (PBMC, Trivalent Influenza Vaccine). Sorted by descending absolute log_2 fold change (Day 1 vs. pre-treatment). Gene model summaries and annotations are based on Ensembl Version 87.

Ensembl Gene ID	Ensembl Gene Name	Ensembl Gene Description	Gene Type	Log_2 Fold Change (Day 2 vs. pre-treatment)	Average Log_2 CPM	Likelihood Ratio Test Statistic	P-Value	FDR Adjusted P-Value
ENSG00000122786	CALD1	caldesmon 1 [Source:HGNC Symbol;Acc:HGNC:1441]	protein coding	1.43	1.58	17.71	0.00	0.00
ENSG00000177324	BEND2	BEN domain containing 2 [Source:HGNC Symbol;Acc:HGNC:28509]	protein coding	1.26	2.43	44.85	0.00	0.00
ENSG00000101335	MYL9	myosin light chain 9 [Source:HGNC Symbol;Acc:HGNC:15754]	protein coding	1.23	4.00	88.66	0.00	0.00
ENSG00000004799	PDK4	pyruvate dehydrogenase kinase 4 [Source:HGNC Symbol;Acc:HGNC:8812]	protein coding	1.22	4.97	67.35	0.00	0.00
ENSG00000236304			antisense	1.15	3.16	44.73	0.00	0.00
ENSG00000162366	PDZK1IP1	PDZK1 interacting protein 1 [Source:HGNC Symbol;Acc:HGNC:16887]	protein coding	1.15	1.39	13.72	0.00	0.02
ENSG00000163430	FSTL1	follistatin like 1 [Source:HGNC Symbol;Acc:HGNC:3972]	protein coding	1.10	1.87	19.62	0.00	0.00
ENSG00000149131	SERPING1	serpin family G member 1 [Source:HGNC Symbol;Acc:HGNC:1228]	protein coding	1.10	3.08	28.33	0.00	0.00
ENSG00000088826	SMOX	spermine oxidase [Source:HGNC Symbol;Acc:HGNC:15862]	protein coding	1.06	3.19	40.32	0.00	0.00
ENSG00000171611	PTCRA	pre T-cell antigen receptor alpha [Source:HGNC Symbol;Acc:HGNC:21290]	protein coding	1.02	1.95	21.37	0.00	0.00
ENSG00000163737	PF4	platelet factor 4 [Source:HGNC Symbol;Acc:HGNC:8861]	protein coding	1.02	4.68	54.57	0.00	0.00
ENSG00000184702	SEPT5	septin 5 [Source:HGNC Symbol;Acc:HGNC:9164]	protein coding	1.00	3.41	28.28	0.00	0.00
ENSG00000161911	TREML1	triggering receptor expressed on myeloid cells like 1 [Source:HGNC Symbol;Acc:HGNC:20434]	protein coding	0.98	3.73	47.92	0.00	0.00
ENSG00000196787	HIST1H2AG	histone cluster 1 H2A family member g [Source:HGNC Symbol;Acc:HGNC:4737]	protein coding	0.98	2.15	18.55	0.00	0.00
ENSG00000120885	CLU	clusterin [Source:HGNC Symbol;Acc:HGNC:2095]	protein coding	0.97	6.04	64.05	0.00	0.00

Ensembl Gene ID	Ensembl Gene Name	Ensembl Gene Description	Gene Type	Log_2 Fold Change (Day 2 vs. pre-treatment)	Average Log_2 CPM	Likelihood Ratio Test Statistic	P-Value	FDR Adjusted P-Value
ENSG00000163735	CXCL5	C-X-C motif chemokine ligand 5 [Source:HGNC Symbol;Acc:HGNC:10642]	protein coding	0.97	3.27	26.07	0.00	0.00
ENSG00000088726	TMEM40	transmembrane protein 40 [Source:HGNC Symbol;Acc:HGNC:25620]	protein coding	0.97	2.40	17.75	0.00	0.00
ENSG00000278828	HIST1H3H	histone cluster 1 H3 family member h [Source:HGNC Symbol;Acc:HGNC:4775]	protein coding	0.96	3.71	20.67	0.00	0.00
ENSG00000168062	BATF2	basic leucine zipper ATF-like transcription factor 2 [Source:HGNC Symbol;Acc:HGNC:25163]	protein coding	0.94	1.74	11.46	0.00	0.04
ENSG00000005961	ITGA2B	integrin subunit alpha 2b [Source:HGNC Symbol;Acc:HGNC:6138]	protein coding	0.94	5.59	61.50	0.00	0.00
ENSG00000101162	TUBB1	tubulin beta 1 class VI [Source:HGNC Symbol;Acc:HGNC:16257]	protein coding	0.93	6.84	66.73	0.00	0.00
ENSG00000085733	CTTN	cortactin [Source:HGNC Symbol;Acc:HGNC:3338]	protein coding	0.92	3.56	48.87	0.00	0.00
ENSG00000205309	NT5M	5',3'-nucleotidase, mitochondrial [Source:HGNC Symbol;Acc:HGNC:15769]	protein coding	0.91	2.07	17.86	0.00	0.00
ENSG00000113140	SPARC	secreted protein acidic and cysteine rich [Source:HGNC Symbol;Acc:HGNC:11219]	protein coding	0.91	6.45	67.20	0.00	0.00
ENSG00000156265	MAP3K7CL	MAP3K7 C-terminal like [Source:HGNC Symbol;Acc:HGNC:16457]	protein coding	0.91	5.76	34.02	0.00	0.00
ENSG00000082781	ITGB5	integrin subunit beta 5 [Source:HGNC Symbol;Acc:HGNC:6160]	protein coding	0.89	3.67	42.31	0.00	0.00
ENSG00000151023	ENKUR	enkurin, TRPC channel interacting protein [Source:HGNC Symbol;Acc:HGNC:28388]	protein coding	0.88	2.52	21.13	0.00	0.00
ENSG00000180573	HIST1H2AC	histone cluster 1 H2A family member c [Source:HGNC Symbol;Acc:HGNC:4733]	protein coding	0.87	5.96	39.12	0.00	0.00
ENSG00000168497	SDPR	serum deprivation response [Source:HGNC Symbol;Acc:HGNC:10690]	protein coding	0.87	6.50	41.65	0.00	0.00
ENSG00000184678	HIST2H2BE	histone cluster 2 H2B family member e [Source:HGNC Symbol;Acc:HGNC:4760]	protein coding	0.87	4.58	24.47	0.00	0.00
ENSG00000163736	PPBP	pro-platelet basic protein [Source:HGNC Symbol;Acc:HGNC:9240]	protein coding	0.86	6.41	48.51	0.00	0.00
ENSG00000137198	GMPR	guanosine monophosphate reductase [Source:HGNC Symbol;Acc:HGNC:4376]	protein coding	0.85	2.47	19.28	0.00	0.00
ENSG00000250334	LINC00989	long intergenic non-protein coding RNA 989 [Source:HGNC Symbol;Acc:HGNC:48918]	lincRNA	0.85	2.58	12.49	0.00	0.03
ENSG00000154146	NRGN	neurogranin [Source:HGNC Symbol;Acc:HGNC:8000]	protein coding	0.84	6.99	39.11	0.00	0.00
ENSG00000169245	CXCL10	C-X-C motif chemokine ligand 10 [Source:HGNC Symbol;Acc:HGNC:10637]	protein coding	0.83	1.95	12.86	0.00	0.02
ENSG00000173210	ABLIM3	actin binding LIM protein family member 3 [Source:HGNC Symbol;Acc:HGNC:29132]	protein coding	0.83	3.30	28.35	0.00	0.00
ENSG00000165702	GFI1B	growth factor independent 1B transcriptional repressor [Source:HGNC Symbol;Acc:HGNC:4238]	protein coding	0.83	3.06	22.02	0.00	0.00
ENSG00000119862	LGALSL	galectin like [Source:HGNC Symbol;Acc:HGNC:25012]	protein coding	0.83	2.90	18.68	0.00	0.00

Ensembl Gene ID	Ensembl Gene Name	Ensembl Gene Description	Gene Type	Log_2 Fold Change (Day 2 vs. pre-treatment)	Average Log_2 CPM	Likelihood Ratio Test Statistic	P-Value	FDR Adjusted P-Value
ENSG00000166091	CMTM5	CKLF like MARVEL transmembrane domain containing 5 [Source:HGNC Symbol;Acc:HGNC:19176]	protein coding	0.81	2.12	14.93	0.00	0.01
ENSG00000140479	PCSK6	proprotein convertase subtilisin/kexin type 6 [Source:HGNC Symbol;Acc:HGNC:8569]	protein coding	0.79	2.46	16.81	0.00	0.01
ENSG00000119922	IFIT2	interferon induced protein with tetratricopeptide repeats 2 [Source:HGNC Symbol;Acc:HGNC:5409]	protein coding	0.79	6.00	11.79	0.00	0.03
ENSG00000128266	GNAZ	G protein subunit alpha z [Source:HGNC Symbol;Acc:HGNC:4395]	protein coding	0.79	2.95	17.02	0.00	0.01
ENSG00000198478	SH3BGRL2	SH3 domain binding glutamate rich protein like 2 [Source:HGNC Symbol;Acc:HGNC:15567]	protein coding	0.77	4.51	22.56	0.00	0.00
ENSG00000150337	FCGR1A	Fc fragment of IgG receptor Ia [Source:HGNC Symbol;Acc:HGNC:3613]	protein coding	0.75	2.57	11.47	0.00	0.04
ENSG00000187800	PEAR1	platelet endothelial aggregation receptor 1 [Source:HGNC Symbol;Acc:HGNC:33631]	protein coding	0.75	2.72	14.38	0.00	0.01
ENSG00000127920	GNG11	G protein subunit gamma 11 [Source:HGNC Symbol;Acc:HGNC:4403]	protein coding	0.73	4.52	28.04	0.00	0.00
ENSG00000130489	SCO2	SCO2, cytochrome c oxidase assembly protein [Source:HGNC Symbol;Acc:HGNC:10604]	protein coding	0.72	4.19	24.44	0.00	0.00
ENSG00000229754	CXCR2P1	C-X-C motif chemokine receptor 2 pseudogene 1 [Source:HGNC Symbol;Acc:HGNC:6028]	transcribed unprocessed pseudogene	0.72	4.77	39.58	0.00	0.00
ENSG00000187699	C2orf88	chromosome 2 open reading frame 88 [Source:HGNC Symbol;Acc:HGNC:28191]	protein coding	0.71	4.64	28.80	0.00	0.00
ENSG00000005249	PRKAR2B	protein kinase cAMP-dependent type II regulatory subunit beta [Source:HGNC Symbol;Acc:HGNC:9392]	protein coding	0.71	5.79	35.96	0.00	0.00
ENSG00000111644	ACRBP	acrosin binding protein [Source:HGNC Symbol;Acc:HGNC:17195]	protein coding	0.70	4.38	32.47	0.00	0.00
ENSG00000272821			antisense	0.69	3.35	18.98	0.00	0.00
ENSG00000119917	IFIT3	interferon induced protein with tetratricopeptide repeats 3 [Source:HGNC Symbol;Acc:HGNC:5411]	protein coding	0.69	5.69	11.09	0.00	0.04
ENSG00000254614			antisense	0.69	2.57	14.65	0.00	0.01
ENSG00000162512	SDC3	syndecan 3 [Source:HGNC Symbol;Acc:HGNC:10660]	protein coding	0.69	2.69	14.41	0.00	0.01
ENSG00000143995	MEIS1	Meis homeobox 1 [Source:HGNC Symbol;Acc:HGNC:7000]	protein coding	0.69	2.15	10.92	0.00	0.04
ENSG00000164181	ELOVL7	ELOVL fatty acid elongase 7 [Source:HGNC Symbol;Acc:HGNC:26292]	protein coding	0.68	2.70	12.72	0.00	0.02
ENSG00000259207	ITGB3	integrin subunit beta 3 [Source:HGNC Symbol;Acc:HGNC:6156]	protein coding	0.68	4.19	16.89	0.00	0.01
ENSG00000011105	TSPAN9	tetraspanin 9 [Source:HGNC Symbol;Acc:HGNC:21640]	protein coding	0.68	2.34	13.12	0.00	0.02
ENSG00000065534	MYLK	myosin light chain kinase [Source:HGNC Symbol;Acc:HGNC:7590]	protein coding	0.68	3.70	24.30	0.00	0.00

Ensembl Gene ID	Ensembl Gene Name	Ensembl Gene Description	Gene Type	Log_2 Fold Change (Day 2 vs. pre-treatment)	Average Log_2 CPM	Likelihood Ratio Test Statistic	P-Value	FDR Adjusted P-Value
ENSG00000165914	TTC7B	tetratricopeptide repeat domain 7B [Source:HGNC Symbol;Acc:HGNC:19858]	protein coding	0.68	2.80	17.22	0.00	0.01
ENSG00000177989	ODF3B	outer dense fiber of sperm tails 3B [Source:HGNC Symbol;Acc:HGNC:34388]	protein coding	0.67	5.13	11.32	0.00	0.04
ENSG00000141574	SECTM1	secreted and transmembrane 1 [Source:HGNC Symbol;Acc:HGNC:10707]	protein coding	0.66	6.41	26.62	0.00	0.00
ENSG00000073756	PTGS2	prostaglandin-endoperoxide synthase 2 [Source:HGNC Symbol;Acc:HGNC:9605]	protein coding	0.66	4.81	21.02	0.00	0.00
ENSG00000177191	B3GNT8	UDP-GlcNAc:betaGal beta-1,3-N-acetylglucosaminyltransferase 8 [Source:HGNC Symbol;Acc:HGNC:24139]	protein coding	0.66	2.60	11.05	0.00	0.04
ENSG00000143878	RHOB	ras homolog family member B [Source:HGNC Symbol;Acc:HGNC:668]	protein coding	0.66	6.38	33.98	0.00	0.00
ENSG00000183779	ZNF703	zinc finger protein 703 [Source:HGNC Symbol;Acc:HGNC:25883]	protein coding	0.65	4.29	17.25	0.00	0.01
ENSG00000173372	C1QA	complement C1q A chain [Source:HGNC Symbol;Acc:HGNC:1241]	protein coding	0.65	2.69	10.75	0.00	0.05
ENSG00000149564	ESAM	endothelial cell adhesion molecule [Source:HGNC Symbol;Acc:HGNC:17474]	protein coding	0.65	2.91	12.52	0.00	0.03
ENSG00000073737	DHRS9	dehydrogenase/reductase 9 [Source:HGNC Symbol;Acc:HGNC:16888]	protein coding	0.64	2.94	15.04	0.00	0.01
ENSG00000182487	NCF1B	neutrophil cytosolic factor 1B pseudogene [Source:HGNC Symbol;Acc:HGNC:32522]	transcribed unprocessed pseudogene	0.64	4.10	16.27	0.00	0.01
ENSG00000104267	CA2	carbonic anhydrase 2 [Source:HGNC Symbol;Acc:HGNC:1373]	protein coding	0.63	3.74	15.62	0.00	0.01
ENSG00000074416	MGLL	monoglyceride lipase [Source:HGNC Symbol;Acc:HGNC:17038]	protein coding	0.63	4.28	18.76	0.00	0.00
ENSG00000171223	JUNB	JunB proto-oncogene, AP-1 transcription factor subunit [Source:HGNC Symbol;Acc:HGNC:6205]	protein coding	0.63	7.47	21.10	0.00	0.00
ENSG00000108960	MMD	monocyte to macrophage differentiation associated [Source:HGNC Symbol;Acc:HGNC:7153]	protein coding	0.62	5.30	23.47	0.00	0.00
ENSG00000185340	GAS2L1	growth arrest specific 2 like 1 [Source:HGNC Symbol;Acc:HGNC:16955]	protein coding	0.62	4.07	16.73	0.00	0.01
ENSG00000142089	IFITM3	interferon induced transmembrane protein 3 [Source:HGNC Symbol;Acc:HGNC:5414]	protein coding	0.62	6.10	18.71	0.00	0.00
ENSG00000125148	MT2A	metallothionein 2A [Source:HGNC Symbol;Acc:HGNC:7406]	protein coding	0.62	3.66	20.28	0.00	0.00
ENSG00000173110	HSPA6	heat shock protein family A (Hsp70) member 6 [Source:HGNC Symbol;Acc:HGNC:5239]	protein coding	0.61	4.26	11.13	0.00	0.04
ENSG00000019169	MARCO	macrophage receptor with collagenous structure [Source:HGNC Symbol;Acc:HGNC:6895]	protein coding	0.59	3.99	20.55	0.00	0.00
ENSG00000188305	C19orf35	chromosome 19 open reading frame 35 [Source:HGNC Symbol;Acc:HGNC:24793]	protein coding	0.59	2.98	13.23	0.00	0.02

Ensembl Gene ID	Ensembl Gene Name	Ensembl Gene Description	Gene Type	Log_2 Fold Change (Day 2 vs. pre-treatment)	Average Log_2 CPM	Likelihood Ratio Test Statistic	P-Value	FDR Adjusted P-Value
ENSG00000198948	MFAP3L	microfibrillar associated protein 3 like [Source:HGNC Symbol;Acc:HGNC:29083]	protein coding	0.59	3.84	15.64	0.00	0.01
ENSG00000204420	C6orf25	chromosome 6 open reading frame 25 [Source:HGNC Symbol;Acc:HGNC:13937]	protein coding	0.59	4.40	12.84	0.00	0.02

Table 18: Genes differentially expressed at Day 2 compared to pre-treatment (PBMC, Trivalent Influenza Vaccine). Sorted by descending absolute log_2 fold change (Day 2 vs. pre-treatment). Gene model summaries and annotations are based on Ensembl Version 87.

Ensembl Gene ID	Ensembl Gene Name	Ensembl Gene Description	Gene Type	Log_2 Fold Change (Day 3 vs. pre-treatment)	Average Log_2 CPM	Likelihood Ratio Test Statistic	P-Value	FDR Adjusted P-Value
ENSG00000197561	ELANE	elastase, neutrophil expressed [Source:HGNC Symbol;Acc:HGNC:3309]	protein coding	1.15	1.17	14.25	0.00	0.04
ENSG00000162366	PDZK1IP1	PDZK1 interacting protein 1 [Source:HGNC Symbol;Acc:HGNC:16887]	protein coding	0.99	1.31	13.84	0.00	0.04
ENSG00000004799	PDK4	pyruvate dehydrogenase kinase 4 [Source:HGNC Symbol;Acc:HGNC:8812]	protein coding	0.98	4.82	26.85	0.00	0.00
ENSG00000182782	HCAR2	hydroxycarboxylic acid receptor 2 [Source:HGNC Symbol;Acc:HGNC:24827]	protein coding	0.78	2.11	14.87	0.00	0.04
ENSG00000126262	FFAR2	free fatty acid receptor 2 [Source:HGNC Symbol;Acc:HGNC:4501]	protein coding	0.77	3.88	27.74	0.00	0.00
ENSG00000260401			sense overlapping	0.75	2.78	20.44	0.00	0.00
ENSG00000125538	IL1B	interleukin 1 beta [Source:HGNC Symbol;Acc:HGNC:5992]	protein coding	0.74	3.54	37.47	0.00	0.00
ENSG00000173535	TNFRSF10C	TNF receptor superfamily member 10c [Source:HGNC Symbol;Acc:HGNC:11906]	protein coding	0.73	4.76	37.70	0.00	0.00
ENSG00000123700	KCNJ2	potassium voltage-gated channel subfamily J member 2 [Source:HGNC Symbol;Acc:HGNC:6263]	protein coding	0.69	3.52	30.09	0.00	0.00
ENSG00000232810	TNF	tumor necrosis factor [Source:HGNC Symbol;Acc:HGNC:11892]	protein coding	0.67	2.65	21.24	0.00	0.00
ENSG00000204388	HSPA1B	heat shock protein family A (Hsp70) member 1B [Source:HGNC Symbol;Acc:HGNC:5233]	protein coding	0.66	2.86	22.46	0.00	0.00
ENSG00000005381	MPO	myeloperoxidase [Source:HGNC Symbol;Acc:HGNC:7218]	protein coding	0.65	3.68	14.21	0.00	0.04
ENSG00000162551	ALPL	alkaline phosphatase, liver/bone/kidney [Source:HGNC Symbol;Acc:HGNC:438]	protein coding	0.64	3.69	19.17	0.00	0.01
ENSG00000119922	IFIT2	interferon induced protein with tetratricopeptide repeats 2 [Source:HGNC Symbol;Acc:HGNC:5409]	protein coding	0.63	5.76	18.78	0.00	0.01
ENSG00000149131	SERPING1	serpin family G member 1 [Source:HGNC Symbol;Acc:HGNC:1228]	protein coding	0.63	2.75	14.44	0.00	0.04
ENSG00000088827	SIGLEC1	sialic acid binding Ig like lectin 1 [Source:HGNC Symbol;Acc:HGNC:11127]	protein coding	0.62	5.12	25.60	0.00	0.00

Ensembl Gene ID	Ensembl Gene Name	Ensembl Gene Description	Gene Type	Log_2 Fold Change (Day 3 vs. pre-treatment)	Average Log_2 CPM	Likelihood Ratio Test Statistic	P-Value	FDR Adjusted P-Value
ENSG00000154447	SH3RF1	SH3 domain containing ring finger 1 [Source:HGNC Symbol;Acc:HGNC:17650]	protein coding	0.61	2.80	14.37	0.00	0.04
ENSG00000267481			sense intronic	-0.60	2.47	13.32	0.00	0.05
ENSG00000005961	ITGA2B	integrin subunit alpha 2b [Source:HGNC Symbol;Acc:HGNC:6138]	protein coding	0.59	5.38	26.61	0.00	0.00

Table 19: Genes differentially expressed at Day 3 compared to pre-treatment (PBMC, Trivalent Influenza Vaccine). Sorted by descending absolute log_2 fold change (Day 3 vs. pre-treatment). Gene model summaries and annotations are based on Ensembl Version 87.

Ensembl Gene ID	Ensembl Gene Name	Ensembl Gene Description	Gene Type	Log_2 Fold Change (Day 4 vs. pre-treatment)	Average Log_2 CPM	Likelihood Ratio Test Statistic	P-Value	FDR Adjusted P-Value
ENSG00000150687	PRSS23	protease, serine 23 [Source:HGNC Symbol;Acc:HGNC:14370]	protein coding	-0.72	4.80	21.38	0.00	0.01
ENSG00000275302	CCL4	C-C motif chemokine ligand 4 [Source:HGNC Symbol;Acc:HGNC:10630]	protein coding	-0.70	4.51	21.56	0.00	0.01

Table 20: Genes differentially expressed at Day 4 compared to pre-treatment (PBMC, Trivalent Influenza Vaccine). Sorted by descending absolute log_2 fold change (Day 4 vs. pre-treatment). Gene model summaries and annotations are based on Ensembl Version 87.

Ensembl Gene ID	Ensembl Gene Name	Ensembl Gene Description	Gene Type	Log_2 Fold Change (Day 5 vs. pre-treatment)	Average Log_2 CPM	Likelihood Ratio Test Statistic	P-Value	FDR Adjusted P-Value
ENSG00000211896	IGHG1	immunoglobulin heavy constant gamma 1 (G1m marker) [Source:HGNC Symbol;Acc:HGNC:5525]	IG C gene	2.26	9.34	36.76	0.00	0.00
ENSG00000211946	IGHV3-20	immunoglobulin heavy variable 3-20 [Source:HGNC Symbol;Acc:HGNC:5585]	IG V gene	2.04	1.37	21.27	0.00	0.00
ENSG00000253755	IGHGP	immunoglobulin heavy constant gamma P (non-functional) [Source:HGNC Symbol;Acc:HGNC:5529]	IG C pseudogene	2.04	4.00	32.08	0.00	0.00
ENSG00000178445	GLDC	glycine decarboxylase [Source:HGNC Symbol;Acc:HGNC:4313]	protein coding	1.77	2.03	14.28	0.00	0.04
ENSG00000211653	IGLV1-40	immunoglobulin lambda variable 1-40 [Source:HGNC Symbol;Acc:HGNC:5877]	IG V gene	1.74	4.09	29.55	0.00	0.00
ENSG00000101057	MYBL2	MYB proto-oncogene like 2 [Source:HGNC Symbol;Acc:HGNC:7548]	protein coding	1.60	3.47	24.63	0.00	0.00
ENSG00000211669	IGLV3-10	immunoglobulin lambda variable 3-10 [Source:HGNC Symbol;Acc:HGNC:5897]	IG V gene	1.48	2.74	15.07	0.00	0.03
ENSG00000211976	IGHV3-73	immunoglobulin heavy variable 3-73 [Source:HGNC Symbol;Acc:HGNC:5623]	IG V gene	1.48	1.20	16.99	0.00	0.01
ENSG00000232216	IGHV3-43	immunoglobulin heavy variable 3-43 [Source:HGNC Symbol;Acc:HGNC:5604]	IG V gene	1.46	1.84	22.21	0.00	0.00

Ensembl Gene ID	Ensembl Gene Name	Ensembl Gene Description	Gene Type	Log_2 Fold Change (Day 5 vs. pre-treatment)	Average Log_2 CPM	Likelihood Ratio Test Statistic	P-Value	FDR Adjusted P-Value
ENSG00000048462	TNFRSF17	TNF receptor superfamily member 17 [Source:HGNC Symbol;Acc:HGNC:11913]	protein coding	1.44	3.47	15.52	0.00	0.03
ENSG00000211933	IGHV6-1	immunoglobulin heavy variable 6-1 [Source:HGNC Symbol;Acc:HGNC:5662]	IG V gene	1.39	2.69	14.30	0.00	0.04
ENSG00000167900	TK1	thymidine kinase 1 [Source:HGNC Symbol;Acc:HGNC:11830]	protein coding	1.30	2.19	22.01	0.00	0.00
ENSG00000211662	IGLV3-21	immunoglobulin lambda variable 3-21 [Source:HGNC Symbol;Acc:HGNC:5905]	IG V gene	1.28	4.54	30.81	0.00	0.00
ENSG00000224373	IGHV4-59	immunoglobulin heavy variable 4-59 [Source:HGNC Symbol;Acc:HGNC:5654]	IG V gene	1.25	3.01	19.21	0.00	0.01
ENSG00000211659	IGLV3-25	immunoglobulin lambda variable 3-25 [Source:HGNC Symbol;Acc:HGNC:5908]	IG V gene	1.24	3.58	20.90	0.00	0.00
ENSG00000211967	IGHV3-53	immunoglobulin heavy variable 3-53 [Source:HGNC Symbol;Acc:HGNC:5610]	IG V gene	1.21	1.81	14.33	0.00	0.04
ENSG00000211945	IGHV1-18	immunoglobulin heavy variable 1-18 [Source:HGNC Symbol;Acc:HGNC:5549]	IG V gene	1.21	2.96	33.36	0.00	0.00
ENSG00000211660	IGLV2-23	immunoglobulin lambda variable 2-23 [Source:HGNC Symbol;Acc:HGNC:5890]	IG V gene	1.21	3.71	24.23	0.00	0.00
ENSG00000171848	RRM2	ribonucleotide reductase regulatory subunit M2 [Source:HGNC Symbol;Acc:HGNC:10452]	protein coding	1.16	3.78	19.73	0.00	0.01
ENSG00000170476	MZB1	marginal zone B and B1 cell specific protein [Source:HGNC Symbol;Acc:HGNC:30125]	protein coding	1.15	5.28	14.50	0.00	0.04
ENSG00000254709	IGLL5	immunoglobulin lambda like polypeptide 5 [Source:HGNC Symbol;Acc:HGNC:38476]	protein coding	1.12	2.09	15.87	0.00	0.02
ENSG00000123989	CHPF	chondroitin polymerizing factor [Source:HGNC Symbol;Acc:HGNC:24291]	protein coding	0.91	3.08	13.82	0.00	0.05
ENSG00000188536	HBA2	hemoglobin subunit alpha 2 [Source:HGNC Symbol;Acc:HGNC:4824]	protein coding	0.88	5.81	44.28	0.00	0.00
ENSG00000148773	MKI67	marker of proliferation Ki-67 [Source:HGNC Symbol;Acc:HGNC:7107]	protein coding	0.85	4.98	20.95	0.00	0.00
ENSG00000244437	IGKV3-15	immunoglobulin kappa variable 3-15 [Source:HGNC Symbol;Acc:HGNC:5816]	IG V gene	0.85	3.77	18.42	0.00	0.01
ENSG00000211962	IGHV1-46	immunoglobulin heavy variable 1-46 [Source:HGNC Symbol;Acc:HGNC:5554]	IG V gene	0.84	2.66	22.01	0.00	0.00
ENSG00000278196	IGLV2-8	immunoglobulin lambda variable 2-8 [Source:HGNC Symbol;Acc:HGNC:5895]	IG V gene	0.84	3.28	15.91	0.00	0.02
ENSG00000206172	HBA1	hemoglobin subunit alpha 1 [Source:HGNC Symbol;Acc:HGNC:4823]	protein coding	0.82	4.73	22.32	0.00	0.00
ENSG00000239951	IGKV3-20	immunoglobulin kappa variable 3-20 [Source:HGNC Symbol;Acc:HGNC:5817]	IG V gene	0.80	4.67	21.07	0.00	0.00
ENSG00000211934	IGHV1-2	immunoglobulin heavy variable 1-2 [Source:HGNC Symbol;Acc:HGNC:5550]	IG V gene	0.80	2.82	13.85	0.00	0.05
ENSG00000162551	ALPL	alkaline phosphatase, liver/bone/kidney [Source:HGNC Symbol;Acc:HGNC:438]	protein coding	0.77	3.77	26.72	0.00	0.00
ENSG00000173535	TNFRSF10C	TNF receptor superfamily member 10c [Source:HGNC Symbol;Acc:HGNC:11906]	protein coding	0.75	4.75	34.44	0.00	0.00

Ensembl Gene ID	Ensembl Gene Name	Ensembl Gene Description	Gene Type	Log_2 Fold Change (Day 5 vs. pre-treatment)	Average Log_2 CPM	Likelihood Ratio Test Statistic	P-Value	FDR Adjusted P-Value
ENSG00000211966	IGHV5-51	immunoglobulin heavy variable 5-51 [Source:HGNC Symbol;Acc:HGNC:5659]	IG V gene	0.73	3.59	13.83	0.00	0.05
ENSG00000138160	KIF11	kinesin family member 11 [Source:HGNC Symbol;Acc:HGNC:6388]	protein coding	0.72	3.19	16.58	0.00	0.02
ENSG00000211893	IGHG2	immunoglobulin heavy constant gamma 2 (G2m marker) [Source:HGNC Symbol;Acc:HGNC:5526]	IG C gene	0.68	5.86	17.02	0.00	0.01
ENSG00000133048	CHI3L1	chitinase 3 like 1 [Source:HGNC Symbol;Acc:HGNC:1932]	protein coding	0.66	3.43	14.48	0.00	0.04
ENSG00000124772	CPNE5	copine 5 [Source:HGNC Symbol;Acc:HGNC:2318]	protein coding	0.65	4.67	17.81	0.00	0.01
ENSG00000186529	CYP4F3	cytochrome P450 family 4 subfamily F member 3 [Source:HGNC Symbol;Acc:HGNC:2646]	protein coding	0.64	4.14	17.14	0.00	0.01
ENSG00000244734	HBB	hemoglobin subunit beta [Source:HGNC Symbol;Acc:HGNC:4827]	protein coding	0.63	9.51	22.66	0.00	0.00
ENSG00000162747	FCGR3B	Fc fragment of IgG receptor IIIb [Source:HGNC Symbol;Acc:HGNC:3620]	protein coding	0.61	6.90	17.59	0.00	0.01
ENSG00000239839	DEFA3	defensin alpha 3 [Source:HGNC Symbol;Acc:HGNC:2762]	protein coding	0.61	4.25	13.92	0.00	0.05
ENSG00000163464	CXCR1	C-X-C motif chemokine receptor 1 [Source:HGNC Symbol;Acc:HGNC:6026]	protein coding	0.60	5.62	18.57	0.00	0.01

Table 21: Genes differentially expressed at Day 5 compared to pre-treatment (PBMC, Trivalent Influenza Vaccine). Sorted by descending absolute log_2 fold change (Day 5 vs. pre-treatment). Gene model summaries and annotations are based on Ensembl Version 87.

Ensembl Gene ID	Ensembl Gene Name	Ensembl Gene Description	Gene Type	Log_2 Fold Change (Day 6 vs. pre-treatment)	Average Log_2 CPM	Likelihood Ratio Test Statistic	P-Value	FDR Adjusted P-Value
ENSG00000253822	IGLV3-24	immunoglobulin lambda variable 3-24 (pseudogene) [Source:HGNC Symbol;Acc:HGNC:5907]	IG V pseudogene	7.22	0.44	36.91	0.00	0.00
ENSG00000211896	IGHG1	immunoglobulin heavy constant gamma 1 (G1m marker) [Source:HGNC Symbol;Acc:HGNC:5525]	IG C gene	2.96	9.53	106.80	0.00	0.00
ENSG00000211938	IGHV3-7	immunoglobulin heavy variable 3-7 [Source:HGNC Symbol;Acc:HGNC:5620]	IG V gene	2.92	6.49	36.04	0.00	0.00
ENSG00000253818	IGLV1-41	immunoglobulin lambda variable 1-41 (pseudogene) [Source:HGNC Symbol;Acc:HGNC:5878]	IG V pseudogene	2.65	2.01	27.02	0.00	0.00
ENSG00000211653	IGLV1-40	immunoglobulin lambda variable 1-40 [Source:HGNC Symbol;Acc:HGNC:5877]	IG V gene	2.63	5.16	40.05	0.00	0.00
ENSG00000253755	IGHGP	immunoglobulin heavy constant gamma P (non-functional) [Source:HGNC Symbol;Acc:HGNC:5529]	IG C pseudogene	2.61	4.15	100.90	0.00	0.00
ENSG00000115884	SDC1	syndecan 1 [Source:HGNC Symbol;Acc:HGNC:10658]	protein coding	2.43	0.85	23.81	0.00	0.00
ENSG00000211651	IGLV1-44	immunoglobulin lambda variable 1-44 [Source:HGNC Symbol;Acc:HGNC:5879]	IG V gene	2.35	4.73	20.12	0.00	0.00

Ensembl Gene ID	Ensembl Gene Name	Ensembl Gene Description	Gene Type	Log_2 Fold Change (Day 6 vs. pre-treatment)	Average Log_2 CPM	Likelihood Ratio Test Statistic	P-Value	FDR Adjusted P-Value
ENSG00000105974	CAV1	caveolin 1 [Source:HGNC Symbol;Acc:HGNC:1527]	protein coding	2.32	1.31	24.15	0.00	0.00
ENSG00000239975	IGKV1D-33	immunoglobulin kappa variable 1D-33 [Source:HGNC Symbol;Acc:HGNC:5753]	IG V gene	2.20	0.92	14.69	0.00	0.02
ENSG00000271178	IGHV3OR16-13	immunoglobulin heavy variable 3/OR16-13 (non-functional) [Source:HGNC Symbol;Acc:HGNC:5637]	IG V gene	2.16	0.83	19.50	0.00	0.00
ENSG00000211662	IGLV3-21	immunoglobulin lambda variable 3-21 [Source:HGNC Symbol;Acc:HGNC:5905]	IG V gene	2.06	4.98	82.26	0.00	0.00
ENSG00000243238	IGKV2-30	immunoglobulin kappa variable 2-30 [Source:HGNC Symbol;Acc:HGNC:5785]	IG V gene	2.05	2.69	27.95	0.00	0.00
ENSG00000178445	GLDC	glycine decarboxylase [Source:HGNC Symbol;Acc:HGNC:4313]	protein coding	2.05	2.01	41.45	0.00	0.00
ENSG00000280411	IGHV1-69-2	immunoglobulin heavy variable 1-69-2 [Source:HGNC Symbol;Acc:HGNC:5562]	IG V gene	2.03	2.39	38.09	0.00	0.00
ENSG00000242076	IGKV1-33	immunoglobulin kappa variable 1-33 [Source:HGNC Symbol;Acc:HGNC:5737]	IG V gene	1.98	1.60	17.28	0.00	0.00
ENSG00000211644	IGLV1-51	immunoglobulin lambda variable 1-51 [Source:HGNC Symbol;Acc:HGNC:5882]	IG V gene	1.95	3.93	18.23	0.00	0.00
ENSG00000211659	IGLV3-25	immunoglobulin lambda variable 3-25 [Source:HGNC Symbol;Acc:HGNC:5908]	IG V gene	1.95	4.09	58.45	0.00	0.00
ENSG00000211648	IGLV1-47	immunoglobulin lambda variable 1-47 [Source:HGNC Symbol;Acc:HGNC:5880]	IG V gene	1.93	3.91	19.79	0.00	0.00
ENSG00000211677	IGLC2	immunoglobulin lambda constant 2 [Source:HGNC Symbol;Acc:HGNC:5856]	IG C gene	1.88	6.95	42.51	0.00	0.00
ENSG00000211967	IGHV3-53	immunoglobulin heavy variable 3-53 [Source:HGNC Symbol;Acc:HGNC:5610]	IG V gene	1.86	2.24	70.10	0.00	0.00
ENSG00000211972	IGHV3-66	immunoglobulin heavy variable 3-66 [Source:HGNC Symbol;Acc:HGNC:5619]	IG V gene	1.83	1.28	27.73	0.00	0.00
ENSG00000197476			processed pseudogene	1.81	0.87	15.36	0.00	0.01
ENSG00000048462	TNFRSF17	TNF receptor superfamily member 17 [Source:HGNC Symbol;Acc:HGNC:11913]	protein coding	1.80	3.32	94.77	0.00	0.00
ENSG00000253451	IGLV2-28	immunoglobulin lambda variable 2-28 (pseudogene) [Source:HGNC Symbol;Acc:HGNC:5891]	IG V pseudogene	1.72	2.22	19.58	0.00	0.00
ENSG00000211897	IGHG3	immunoglobulin heavy constant gamma 3 (G3m marker) [Source:HGNC Symbol;Acc:HGNC:5527]	IG C gene	1.71	6.03	59.05	0.00	0.00
ENSG00000211933	IGHV6-1	immunoglobulin heavy variable 6-1 [Source:HGNC Symbol;Acc:HGNC:5662]	IG V gene	1.70	2.85	41.79	0.00	0.00
ENSG00000211685	IGLC7	immunoglobulin lambda constant 7 [Source:HGNC Symbol;Acc:HGNC:5861]	IG C gene	1.69	1.33	16.84	0.00	0.01
ENSG00000211652	IGLV7-43	immunoglobulin lambda variable 7-43 [Source:HGNC Symbol;Acc:HGNC:5929]	IG V gene	1.65	2.62	15.70	0.00	0.01
ENSG00000241755	IGKV1-9	immunoglobulin kappa variable 1-9 [Source:HGNC Symbol;Acc:HGNC:5744]	IG V gene	1.65	2.76	19.22	0.00	0.00

Ensembl Gene ID	Ensembl Gene Name	Ensembl Gene Description	Gene Type	Log_2 Fold Change (Day 6 vs. pre-treatment)	Average Log_2 CPM	Likelihood Ratio Test Statistic	P-Value	FDR Adjusted P-Value
ENSG00000254395	IGHV4-55	immunoglobulin heavy variable 4-55 (pseudogene) [Source:HGNC Symbol;Acc:HGNC:5653]	IG V pseudogene	1.64	0.87	13.94	0.00	0.02
ENSG00000211673	IGLV3-1	immunoglobulin lambda variable 3-1 [Source:HGNC Symbol;Acc:HGNC:5896]	IG V gene	1.61	4.65	105.27	0.00	0.00
ENSG00000224373	IGHV4-59	immunoglobulin heavy variable 4-59 [Source:HGNC Symbol;Acc:HGNC:5654]	IG V gene	1.60	3.30	51.84	0.00	0.00
ENSG00000211955	IGHV3-33	immunoglobulin heavy variable 3-33 [Source:HGNC Symbol;Acc:HGNC:5596]	IG V gene	1.59	2.38	23.05	0.00	0.00
ENSG00000224650	IGHV3-74	immunoglobulin heavy variable 3-74 [Source:HGNC Symbol;Acc:HGNC:5624]	IG V gene	1.58	3.53	20.58	0.00	0.00
ENSG00000211976	IGHV3-73	immunoglobulin heavy variable 3-73 [Source:HGNC Symbol;Acc:HGNC:5623]	IG V gene	1.58	1.18	25.25	0.00	0.00
ENSG00000241294	IGKV2-24	immunoglobulin kappa variable 2-24 [Source:HGNC Symbol;Acc:HGNC:5781]	IG V gene	1.56	2.15	13.84	0.00	0.02
ENSG00000239264	TXNDC5	thioredoxin domain containing 5 [Source:HGNC Symbol;Acc:HGNC:21073]	protein coding	1.49	2.63	37.15	0.00	0.00
ENSG00000211679	IGLC3	immunoglobulin lambda constant 3 (Kern-Oz+ marker) [Source:HGNC Symbol;Acc:HGNC:5857]	IG C gene	1.48	6.05	46.32	0.00	0.00
ENSG00000170476	MZB1	marginal zone B and B1 cell specific protein [Source:HGNC Symbol;Acc:HGNC:30125]	protein coding	1.48	5.28	59.80	0.00	0.00
ENSG00000211649	IGLV7-46	immunoglobulin lambda variable 7-46 (gene/pseudogene) [Source:HGNC Symbol;Acc:HGNC:5930]	IG V gene	1.47	1.72	21.44	0.00	0.00
ENSG00000241351	IGKV3-11	immunoglobulin kappa variable 3-11 [Source:HGNC Symbol;Acc:HGNC:5815]	IG V gene	1.43	4.28	47.53	0.00	0.00
ENSG00000211669	IGLV3-10	immunoglobulin lambda variable 3-10 [Source:HGNC Symbol;Acc:HGNC:5897]	IG V gene	1.43	2.71	30.81	0.00	0.00
ENSG00000132465	JCHAIN	joining chain of multimeric IgA and IgM [Source:HGNC Symbol;Acc:HGNC:5713]	protein coding	1.42	8.45	26.33	0.00	0.00
ENSG00000251546	IGKV1D-39	immunoglobulin kappa variable 1D-39 [Source:HGNC Symbol;Acc:HGNC:5756]	IG V gene	1.42	1.00	16.45	0.00	0.01
ENSG00000101057	MYBL2	MYB proto-oncogene like 2 [Source:HGNC Symbol;Acc:HGNC:7548]	protein coding	1.41	3.19	70.95	0.00	0.00
ENSG00000242371	IGKV1-39	immunoglobulin kappa variable 1-39 (gene/pseudogene) [Source:HGNC Symbol;Acc:HGNC:5740]	IG V gene	1.39	0.86	15.43	0.00	0.01
ENSG00000211668	IGLV2-11	immunoglobulin lambda variable 2-11 [Source:HGNC Symbol;Acc:HGNC:5887]	IG V gene	1.37	3.80	24.35	0.00	0.00
ENSG00000211892	IGHG4	immunoglobulin heavy constant gamma 4 (G4m marker) [Source:HGNC Symbol;Acc:HGNC:5528]	IG C gene	1.36	4.01	27.27	0.00	0.00
ENSG00000232216	IGHV3-43	immunoglobulin heavy variable 3-43 [Source:HGNC Symbol;Acc:HGNC:5604]	IG V gene	1.35	1.62	19.38	0.00	0.00
ENSG00000136010	ALDH1L2	aldehyde dehydrogenase 1 family member L2 [Source:HGNC Symbol;Acc:HGNC:26777]	protein coding	1.31	1.15	14.61	0.00	0.02
ENSG00000211943	IGHV3-15	immunoglobulin heavy variable 3-15 [Source:HGNC Symbol;Acc:HGNC:5582]	IG V gene	1.27	3.39	23.10	0.00	0.00
ENSG00000211964	IGHV3-48	immunoglobulin heavy variable 3-48 [Source:HGNC Symbol;Acc:HGNC:5606]	IG V gene	1.21	3.79	25.82	0.00	0.00

Ensembl Gene ID	Ensembl Gene Name	Ensembl Gene Description	Gene Type	Log_2 Fold Change (Day 6 vs. pre-treatment)	Average Log_2 CPM	Likelihood Ratio Test Statistic	P-Value	FDR Adjusted P-Value
ENSG00000211935	IGHV1-3	immunoglobulin heavy variable 1-3 [Source:HGNC Symbol;Acc:HGNC:5552]	IG V gene	1.20	2.69	12.55	0.00	0.04
ENSG00000211941	IGHV3-11	immunoglobulin heavy variable 3-11 (gene/pseudogene) [Source:HGNC Symbol;Acc:HGNC:5580]	IG V gene	1.20	3.08	17.15	0.00	0.01
ENSG00000211947	IGHV3-21	immunoglobulin heavy variable 3-21 [Source:HGNC Symbol;Acc:HGNC:5586]	IG V gene	1.19	4.04	30.74	0.00	0.00
ENSG00000211592	IGKC	immunoglobulin kappa constant [Source:HGNC Symbol;Acc:HGNC:5716]	IG C gene	1.19	9.60	46.76	0.00	0.00
ENSG00000211945	IGHV1-18	immunoglobulin heavy variable 1-18 [Source:HGNC Symbol;Acc:HGNC:5549]	IG V gene	1.17	2.90	32.06	0.00	0.00
ENSG00000211899	IGHM	immunoglobulin heavy constant mu [Source:HGNC Symbol;Acc:HGNC:5541]	IG C gene	1.17	10.22	13.98	0.00	0.02
ENSG00000243466	IGKV1-5	immunoglobulin kappa variable 1-5 [Source:HGNC Symbol;Acc:HGNC:5741]	IG V gene	1.16	5.00	23.34	0.00	0.00
ENSG00000254709	IGLL5	immunoglobulin lambda like polypeptide 5 [Source:HGNC Symbol;Acc:HGNC:38476]	protein coding	1.16	2.05	17.35	0.00	0.00
ENSG00000244575	IGKV1-27	immunoglobulin kappa variable 1-27 [Source:HGNC Symbol;Acc:HGNC:5735]	IG V gene	1.15	2.64	22.35	0.00	0.00
ENSG00000211660	IGLV2-23	immunoglobulin lambda variable 2-23 [Source:HGNC Symbol;Acc:HGNC:5890]	IG V gene	1.08	3.57	52.43	0.00	0.00
ENSG00000282639			IG V gene	1.08	2.01	12.74	0.00	0.04
ENSG00000183508	FAM46C	family with sequence similarity 46 member C [Source:HGNC Symbol;Acc:HGNC:24712]	protein coding	1.07	5.99	25.65	0.00	0.00
ENSG00000211666	IGLV2-14	immunoglobulin lambda variable 2-14 [Source:HGNC Symbol;Acc:HGNC:5888]	IG V gene	1.05	4.10	16.05	0.00	0.01
ENSG00000239951	IGKV3-20	immunoglobulin kappa variable 3-20 [Source:HGNC Symbol;Acc:HGNC:5817]	IG V gene	1.02	4.76	44.31	0.00	0.00
ENSG00000244437	IGKV3-15	immunoglobulin kappa variable 3-15 [Source:HGNC Symbol;Acc:HGNC:5816]	IG V gene	1.01	3.89	43.40	0.00	0.00
ENSG00000211962	IGHV1-46	immunoglobulin heavy variable 1-46 [Source:HGNC Symbol;Acc:HGNC:5554]	IG V gene	0.98	2.74	22.60	0.00	0.00
ENSG00000211934	IGHV1-2	immunoglobulin heavy variable 1-2 [Source:HGNC Symbol;Acc:HGNC:5550]	IG V gene	0.93	2.98	23.64	0.00	0.00
ENSG00000197705	KLHL14	kelch like family member 14 [Source:HGNC Symbol;Acc:HGNC:29266]	protein coding	0.93	3.10	28.68	0.00	0.00
ENSG00000278196	IGLV2-8	immunoglobulin lambda variable 2-8 [Source:HGNC Symbol;Acc:HGNC:5895]	IG V gene	0.90	3.33	19.32	0.00	0.00
ENSG00000211959	IGHV4-39	immunoglobulin heavy variable 4-39 [Source:HGNC Symbol;Acc:HGNC:5651]	IG V gene	0.88	2.93	15.06	0.00	0.01
ENSG00000135916	ITM2C	integral membrane protein 2C [Source:HGNC Symbol;Acc:HGNC:6175]	protein coding	0.87	6.26	38.65	0.00	0.00
ENSG00000128438	TBC1D27	TBC1 domain family member 27 [Source:HGNC Symbol;Acc:HGNC:28104]	transcribed unprocessed pseudogene	0.86	3.24	23.50	0.00	0.00

Ensembl Gene ID	Ensembl Gene Name	Ensembl Gene Description	Gene Type	Log_2 Fold Change (Day 6 vs. pre-treatment)	Average Log_2 CPM	Likelihood Ratio Test Statistic	P-Value	FDR Adjusted P-Value
ENSG00000170456	DENND5B	DENN domain containing 5B [Source:HGNC Symbol;Acc:HGNC:28338]	protein coding	0.85	4.05	34.44	0.00	0.00
ENSG00000240505	TNFRSF13B	TNF receptor superfamily member 13B [Source:HGNC Symbol;Acc:HGNC:18153]	protein coding	0.85	2.98	24.39	0.00	0.00
ENSG00000270550	IGHV3-30	immunoglobulin heavy variable 3-30 [Source:HGNC Symbol;Acc:HGNC:5591]	IG V gene	0.81	3.23	28.08	0.00	0.00
ENSG00000123989	CHPF	chondroitin polymerizing factor [Source:HGNC Symbol;Acc:HGNC:24291]	protein coding	0.81	3.03	19.22	0.00	0.00
ENSG00000169679	BUB1	BUB1 mitotic checkpoint serine/threonine kinase [Source:HGNC Symbol;Acc:HGNC:1148]	protein coding	0.79	2.54	19.02	0.00	0.00
ENSG00000118985	ELL2	elongation factor for RNA polymerase II 2 [Source:HGNC Symbol;Acc:HGNC:17064]	protein coding	0.79	4.94	29.95	0.00	0.00
ENSG00000171241	SHCBP1	SHC binding and spindle associated 1 [Source:HGNC Symbol;Acc:HGNC:29547]	protein coding	0.77	2.34	15.31	0.00	0.01
ENSG00000282651			IG V gene	0.76	2.33	12.74	0.00	0.04
ENSG00000211966	IGHV5-51	immunoglobulin heavy variable 5-51 [Source:HGNC Symbol;Acc:HGNC:5659]	IG V gene	0.76	3.55	15.39	0.00	0.01
ENSG00000177301	KCNA2	potassium voltage-gated channel subfamily A member 2 [Source:HGNC Symbol;Acc:HGNC:6220]	protein coding	0.74	2.34	13.77	0.00	0.02
ENSG00000099958	DERL3	derlin 3 [Source:HGNC Symbol;Acc:HGNC:14236]	protein coding	0.74	4.17	29.11	0.00	0.00
ENSG00000110777	POU2AF1	POU class 2 associating factor 1 [Source:HGNC Symbol;Acc:HGNC:9211]	protein coding	0.73	5.43	45.79	0.00	0.00
ENSG00000171848	RRM2	ribonucleotide reductase regulatory subunit M2 [Source:HGNC Symbol;Acc:HGNC:10452]	protein coding	0.73	3.42	19.19	0.00	0.00
ENSG00000124772	CPNE5	copine 5 [Source:HGNC Symbol;Acc:HGNC:2318]	protein coding	0.71	4.70	33.15	0.00	0.00
ENSG00000211598	IGKV4-1	immunoglobulin kappa variable 4-1 [Source:HGNC Symbol;Acc:HGNC:5834]	IG V gene	0.70	4.78	27.50	0.00	0.00
ENSG00000211949	IGHV3-23	immunoglobulin heavy variable 3-23 [Source:HGNC Symbol;Acc:HGNC:5588]	IG V gene	0.66	4.80	12.43	0.00	0.05
ENSG00000169116	PARM1	prostate androgen-regulated mucin-like protein 1 [Source:HGNC Symbol;Acc:HGNC:24536]	protein coding	0.64	2.94	14.85	0.00	0.01
ENSG00000135862	LAMC1	laminin subunit gamma 1 [Source:HGNC Symbol;Acc:HGNC:6492]	protein coding	0.63	3.32	13.50	0.00	0.03
ENSG00000211893	IGHG2	immunoglobulin heavy constant gamma 2 (G2m marker) [Source:HGNC Symbol;Acc:HGNC:5526]	IG C gene	0.61	5.90	33.67	0.00	0.00
ENSG00000148773	MKI67	marker of proliferation Ki-67 [Source:HGNC Symbol;Acc:HGNC:7107]	protein coding	0.60	4.76	35.82	0.00	0.00
ENSG00000168209	DDIT4	DNA damage inducible transcript 4 [Source:HGNC Symbol;Acc:HGNC:24944]	protein coding	-0.60	4.39	17.65	0.00	0.00

Table 22: Genes differentially expressed at Day 6 compared to pre-treatment (PBMC, Trivalent Influenza Vaccine). Sorted by descending absolute log_2 fold change (Day 6 vs. pre-treatment). Gene model summaries and annotations are based on Ensembl Version 87.

Ensembl Gene ID	Ensembl Gene Name	Ensembl Gene Description	Gene Type	Log_2 Fold Change (Day 7 vs. pre-treatment)	Average Log_2 CPM	Likelihood Ratio Test Statistic	P-Value	FDR Adjusted P-Value
ENSG00000253822	IGLV3-24	immunoglobulin lambda variable 3-24 (pseudogene) [Source:HGNC Symbol;Acc:HGNC:5907]	IG V pseudogene	6.63	-0.13	30.91	0.00	0.00
ENSG00000211653	IGLV1-40	immunoglobulin lambda variable 1-40 [Source:HGNC Symbol;Acc:HGNC:5877]	IG V gene	2.73	5.27	34.49	0.00	0.00
ENSG00000211896	IGHG1	immunoglobulin heavy constant gamma 1 (G1m marker) [Source:HGNC Symbol;Acc:HGNC:5525]	IG C gene	2.57	9.09	125.81	0.00	0.00
ENSG00000211938	IGHV3-7	immunoglobulin heavy variable 3-7 [Source:HGNC Symbol;Acc:HGNC:5620]	IG V gene	2.36	5.22	64.18	0.00	0.00
ENSG00000115884	SDC1	syndecan 1 [Source:HGNC Symbol;Acc:HGNC:10658]	protein coding	2.27	0.78	22.88	0.00	0.00
ENSG00000211662	IGLV3-21	immunoglobulin lambda variable 3-21 [Source:HGNC Symbol;Acc:HGNC:5905]	IG V gene	2.26	5.84	22.17	0.00	0.00
ENSG00000253755	IGHGP	immunoglobulin heavy constant gamma P (non-functional) [Source:HGNC Symbol;Acc:HGNC:5529]	IG C pseudogene	2.18	3.65	120.81	0.00	0.00
ENSG00000211651	IGLV1-44	immunoglobulin lambda variable 1-44 [Source:HGNC Symbol;Acc:HGNC:5879]	IG V gene	2.06	3.16	36.57	0.00	0.00
ENSG00000105974	CAV1	caveolin 1 [Source:HGNC Symbol;Acc:HGNC:1527]	protein coding	2.05	0.95	29.68	0.00	0.00
ENSG00000271178	IGHV3OR16-13	immunoglobulin heavy variable 3/OR16-13 (non-functional) [Source:HGNC Symbol;Acc:HGNC:5637]	IG V gene	1.93	0.47	22.62	0.00	0.00
ENSG00000211972	IGHV3-66	immunoglobulin heavy variable 3-66 [Source:HGNC Symbol;Acc:HGNC:5619]	IG V gene	1.85	1.28	25.49	0.00	0.00
ENSG00000211659	IGLV3-25	immunoglobulin lambda variable 3-25 [Source:HGNC Symbol;Acc:HGNC:5908]	IG V gene	1.82	4.03	18.52	0.00	0.00
ENSG00000211673	IGLV3-1	immunoglobulin lambda variable 3-1 [Source:HGNC Symbol;Acc:HGNC:5896]	IG V gene	1.80	5.01	78.67	0.00	0.00
ENSG00000211677	IGLC2	immunoglobulin lambda constant 2 [Source:HGNC Symbol;Acc:HGNC:5856]	IG C gene	1.76	7.23	25.80	0.00	0.00
ENSG00000211670	IGLV3-9	immunoglobulin lambda variable 3-9 (gene/pseudogene) [Source:HGNC Symbol;Acc:HGNC:5918]	IG V gene	1.75	2.11	40.91	0.00	0.00
ENSG00000224373	IGHV4-59	immunoglobulin heavy variable 4-59 [Source:HGNC Symbol;Acc:HGNC:5654]	IG V gene	1.61	3.28	103.64	0.00	0.00
ENSG00000211967	IGHV3-53	immunoglobulin heavy variable 3-53 [Source:HGNC Symbol;Acc:HGNC:5610]	IG V gene	1.60	2.07	22.97	0.00	0.00
ENSG00000178445	GLDC	glycine decarboxylase [Source:HGNC Symbol;Acc:HGNC:4313]	protein coding	1.58	1.70	22.34	0.00	0.00
ENSG00000211950	IGHV1-24	immunoglobulin heavy variable 1-24 [Source:HGNC Symbol;Acc:HGNC:5551]	IG V gene	1.46	1.35	15.31	0.00	0.02
ENSG00000243238	IGKV2-30	immunoglobulin kappa variable 2-30 [Source:HGNC Symbol;Acc:HGNC:5785]	IG V gene	1.43	2.06	17.62	0.00	0.01
ENSG00000211933	IGHV6-1	immunoglobulin heavy variable 6-1 [Source:HGNC Symbol;Acc:HGNC:5662]	IG V gene	1.42	2.54	49.12	0.00	0.00
ENSG00000211897	IGHG3	immunoglobulin heavy constant gamma 3 (G3m marker) [Source:HGNC Symbol;Acc:HGNC:5527]	IG C gene	1.34	5.77	37.70	0.00	0.00
ENSG00000048462	TNFRSF17	TNF receptor superfamily member 17 [Source:HGNC Symbol;Acc:HGNC:11913]	protein coding	1.32	2.99	46.26	0.00	0.00

Ensembl Gene ID	Ensembl Gene Name	Ensembl Gene Description	Gene Type	Log_2 Fold Change (Day 7 vs. pre-treatment)	Average Log_2 CPM	Likelihood Ratio Test Statistic	P-Value	FDR Adjusted P-Value
ENSG00000211964	IGHV3-48	immunoglobulin heavy variable 3-48 [Source:HGNC Symbol;Acc:HGNC:5606]	IG V gene	1.31	3.82	65.91	0.00	0.00
ENSG00000211644	IGLV1-51	immunoglobulin lambda variable 1-51 [Source:HGNC Symbol;Acc:HGNC:5882]	IG V gene	1.25	2.54	34.58	0.00	0.00
ENSG00000170476	MZB1	marginal zone B and B1 cell specific protein [Source:HGNC Symbol;Acc:HGNC:30125]	protein coding	1.25	5.08	43.17	0.00	0.00
ENSG00000239264	TXNDC5	thioredoxin domain containing 5 [Source:HGNC Symbol;Acc:HGNC:21073]	protein coding	1.19	2.47	17.95	0.00	0.01
ENSG00000211679	IGLC3	immunoglobulin lambda constant 3 (Kern-Oz+ marker) [Source:HGNC Symbol;Acc:HGNC:5857]	IG C gene	1.18	5.76	87.44	0.00	0.00
ENSG00000211955	IGHV3-33	immunoglobulin heavy variable 3-33 [Source:HGNC Symbol;Acc:HGNC:5596]	IG V gene	1.16	1.80	21.55	0.00	0.00
ENSG00000101057	MYBL2	MYB proto-oncogene like 2 [Source:HGNC Symbol;Acc:HGNC:7548]	protein coding	1.16	2.98	37.28	0.00	0.00
ENSG00000132465	JCHAIN	joining chain of multimeric IgA and IgM [Source:HGNC Symbol;Acc:HGNC:5713]	protein coding	1.16	8.11	57.51	0.00	0.00
ENSG00000211892	IGHG4	immunoglobulin heavy constant gamma 4 (G4m marker) [Source:HGNC Symbol;Acc:HGNC:5528]	IG C gene	1.13	3.92	18.34	0.00	0.00
ENSG00000241351	IGKV3-11	immunoglobulin kappa variable 3-11 [Source:HGNC Symbol;Acc:HGNC:5815]	IG V gene	1.10	3.98	40.60	0.00	0.00
ENSG00000211943	IGHV3-15	immunoglobulin heavy variable 3-15 [Source:HGNC Symbol;Acc:HGNC:5582]	IG V gene	1.05	3.08	28.14	0.00	0.00
ENSG00000211945	IGHV1-18	immunoglobulin heavy variable 1-18 [Source:HGNC Symbol;Acc:HGNC:5549]	IG V gene	1.00	2.70	30.27	0.00	0.00
ENSG00000244437	IGKV3-15	immunoglobulin kappa variable 3-15 [Source:HGNC Symbol;Acc:HGNC:5816]	IG V gene	0.99	3.87	39.11	0.00	0.00
ENSG00000211959	IGHV4-39	immunoglobulin heavy variable 4-39 [Source:HGNC Symbol;Acc:HGNC:5651]	IG V gene	0.96	2.99	15.51	0.00	0.02
ENSG00000211934	IGHV1-2	immunoglobulin heavy variable 1-2 [Source:HGNC Symbol;Acc:HGNC:5550]	IG V gene	0.85	2.85	17.36	0.00	0.01
ENSG00000224650	IGHV3-74	immunoglobulin heavy variable 3-74 [Source:HGNC Symbol;Acc:HGNC:5624]	IG V gene	0.84	2.74	26.90	0.00	0.00
ENSG00000211935	IGHV1-3	immunoglobulin heavy variable 1-3 [Source:HGNC Symbol;Acc:HGNC:5552]	IG V gene	0.82	2.09	13.69	0.00	0.04
ENSG00000211899	IGHM	immunoglobulin heavy constant mu [Source:HGNC Symbol;Acc:HGNC:5541]	IG C gene	0.81	9.41	43.26	0.00	0.00
ENSG00000123989	CHPF	chondroitin polymerizing factor [Source:HGNC Symbol;Acc:HGNC:24291]	protein coding	0.80	3.04	22.26	0.00	0.00
ENSG00000211592	IGKC	immunoglobulin kappa constant [Source:HGNC Symbol;Acc:HGNC:5716]	IG C gene	0.79	9.26	41.03	0.00	0.00
ENSG00000184792	OSBP2	oxysterol binding protein 2 [Source:HGNC Symbol;Acc:HGNC:8504]	protein coding	0.78	2.35	17.64	0.00	0.01
ENSG00000197705	KLHL14	kelch like family member 14 [Source:HGNC Symbol;Acc:HGNC:29266]	protein coding	0.78	3.02	15.23	0.00	0.02
ENSG00000211947	IGHV3-21	immunoglobulin heavy variable 3-21 [Source:HGNC Symbol;Acc:HGNC:5586]	IG V gene	0.75	3.68	13.00	0.00	0.05

Ensembl Gene ID	Ensembl Gene Name	Ensembl Gene Description	Gene Type	Log_2 Fold Change (Day 7 vs. pre-treatment)	Average Log_2 CPM	Likelihood Ratio Test Statistic	P-Value	FDR Adjusted P-Value
ENSG00000239951	IGKV3-20	immunoglobulin kappa variable 3-20 [Source:HGNC Symbol;Acc:HGNC:5817]	IG V gene	0.74	4.56	13.77	0.00	0.04
ENSG00000128438	TBC1D27	TBC1 domain family member 27 [Source:HGNC Symbol;Acc:HGNC:28104]	transcribed unprocessed pseudogene	0.73	3.17	18.29	0.00	0.00
ENSG00000135916	ITM2C	integral membrane protein 2C [Source:HGNC Symbol;Acc:HGNC:6175]	protein coding	0.73	6.17	32.13	0.00	0.00
ENSG00000177606	JUN	Jun proto-oncogene, AP-1 transcription factor subunit [Source:HGNC Symbol;Acc:HGNC:6204]	protein coding	0.71	4.63	32.54	0.00	0.00
ENSG00000187608	ISG15	ISG15 ubiquitin-like modifier [Source:HGNC Symbol;Acc:HGNC:4053]	protein coding	0.70	4.44	17.35	0.00	0.01
ENSG00000124772	CPNE5	copine 5 [Source:HGNC Symbol;Acc:HGNC:2318]	protein coding	0.69	4.68	30.67	0.00	0.00
ENSG00000137198	GMPR	guanosine monophosphate reductase [Source:HGNC Symbol;Acc:HGNC:4376]	protein coding	0.67	2.38	13.30	0.00	0.04
ENSG00000088826	SMOX	spermine oxidase [Source:HGNC Symbol;Acc:HGNC:15862]	protein coding	0.65	2.91	15.11	0.00	0.02
ENSG00000272821			antisense	0.64	3.37	13.71	0.00	0.04
ENSG00000005961	ITGA2B	integrin subunit alpha 2b [Source:HGNC Symbol;Acc:HGNC:6138]	protein coding	0.64	5.35	23.61	0.00	0.00
ENSG00000110777	POU2AF1	POU class 2 associating factor 1 [Source:HGNC Symbol;Acc:HGNC:9211]	protein coding	0.63	5.35	28.50	0.00	0.00
ENSG00000099958	DERL3	derlin 3 [Source:HGNC Symbol;Acc:HGNC:14236]	protein coding	0.63	4.11	14.25	0.00	0.03
ENSG00000170456	DENND5B	DENN domain containing 5B [Source:HGNC Symbol;Acc:HGNC:28338]	protein coding	0.63	3.90	15.41	0.00	0.02
ENSG00000240505	TNFRSF13B	TNF receptor superfamily member 13B [Source:HGNC Symbol;Acc:HGNC:18153]	protein coding	0.62	2.83	12.98	0.00	0.05
ENSG00000012223	LTF	lactotransferrin [Source:HGNC Symbol;Acc:HGNC:6720]	protein coding	0.59	6.51	19.51	0.00	0.00
ENSG00000170345	FOS	Fos proto-oncogene, AP-1 transcription factor subunit [Source:HGNC Symbol;Acc:HGNC:3796]	protein coding	0.59	9.32	18.34	0.00	0.00

Table 23: Genes differentially expressed at Day 7 compared to pre-treatment (PBMC, Trivalent Influenza Vaccine). Sorted by descending absolute log_2 fold change (Day 7 vs. pre-treatment). Gene model summaries and annotations are based on Ensembl Version 87.

Ensembl Gene ID	Ensembl Gene Name	Ensembl Gene Description	Gene Type	Log_2 Fold Change (Day 8 vs. pre-treatment)	Average Log_2 CPM	Likelihood Ratio Test Statistic	P-Value	FDR Adjusted P-Value
ENSG00000280800			lincRNA	10.21	4.56	365.53	0.00	0.00
ENSG00000168298	HIST1H1E	histone cluster 1 H1 family member e [Source:HGNC Symbol;Acc:HGNC:4718]	protein coding	4.02	0.01	16.47	0.00	0.01

Ensembl Gene ID	Ensembl Gene Name	Ensembl Gene Description	Gene Type	Log_2 Fold Change (Day 8 vs. pre-treatment)	Average Log_2 CPM	Likelihood Ratio Test Statistic	P-Value	FDR Adjusted P-Value
ENSG00000211662	IGLV3-21	immunoglobulin lambda variable 3-21 [Source:HGNC Symbol;Acc:HGNC:5905]	IG V gene	2.95	6.14	36.91	0.00	0.00
ENSG00000211653	IGLV1-40	immunoglobulin lambda variable 1-40 [Source:HGNC Symbol;Acc:HGNC:5877]	IG V gene	2.91	4.48	127.21	0.00	0.00
ENSG00000211896	IGHG1	immunoglobulin heavy constant gamma 1 (G1m marker) [Source:HGNC Symbol;Acc:HGNC:5525]	IG C gene	2.45	8.07	141.83	0.00	0.00
ENSG00000211670	IGLV3-9	immunoglobulin lambda variable 3-9 (gene/pseudogene) [Source:HGNC Symbol;Acc:HGNC:5918]	IG V gene	2.43	3.03	57.90	0.00	0.00
ENSG00000115884	SDC1	syndecan 1 [Source:HGNC Symbol;Acc:HGNC:10658]	protein coding	2.32	0.35	14.30	0.00	0.03
ENSG00000211651	IGLV1-44	immunoglobulin lambda variable 1-44 [Source:HGNC Symbol;Acc:HGNC:5879]	IG V gene	2.05	3.14	30.10	0.00	0.00
ENSG00000253755	IGHGP	immunoglobulin heavy constant gamma P (non-functional) [Source:HGNC Symbol;Acc:HGNC:5529]	IG C pseudogene	1.95	3.43	42.34	0.00	0.00
ENSG00000211677	IGLC2	immunoglobulin lambda constant 2 [Source:HGNC Symbol;Acc:HGNC:5856]	IG C gene	1.87	6.85	33.15	0.00	0.00
ENSG00000224373	IGHV4-59	immunoglobulin heavy variable 4-59 [Source:HGNC Symbol;Acc:HGNC:5654]	IG V gene	1.84	2.66	62.28	0.00	0.00
ENSG00000211938	IGHV3-7	immunoglobulin heavy variable 3-7 [Source:HGNC Symbol;Acc:HGNC:5620]	IG V gene	1.83	4.34	57.39	0.00	0.00
ENSG00000241755	IGKV1-9	immunoglobulin kappa variable 1-9 [Source:HGNC Symbol;Acc:HGNC:5744]	IG V gene	1.75	2.11	20.12	0.00	0.00
ENSG00000211959	IGHV4-39	immunoglobulin heavy variable 4-39 [Source:HGNC Symbol;Acc:HGNC:5651]	IG V gene	1.72	3.49	18.49	0.00	0.01
ENSG00000126262	FFAR2	free fatty acid receptor 2 [Source:HGNC Symbol;Acc:HGNC:4501]	protein coding	1.46	4.53	17.32	0.00	0.01
ENSG00000182782	HCAR2	hydroxycarboxylic acid receptor 2 [Source:HGNC Symbol;Acc:HGNC:24827]	protein coding	1.33	2.43	14.37	0.00	0.03
ENSG00000173535	TNFRSF10C	TNF receptor superfamily member 10c [Source:HGNC Symbol;Acc:HGNC:11906]	protein coding	1.31	5.26	25.93	0.00	0.00
ENSG00000211897	IGHG3	immunoglobulin heavy constant gamma 3 (G3m marker) [Source:HGNC Symbol;Acc:HGNC:5527]	IG C gene	1.27	4.48	45.12	0.00	0.00
ENSG00000163464	CXCR1	C-X-C motif chemokine receptor 1 [Source:HGNC Symbol;Acc:HGNC:6026]	protein coding	1.24	6.18	24.38	0.00	0.00
ENSG00000162747	FCGR3B	Fc fragment of IgG receptor IIIb [Source:HGNC Symbol;Acc:HGNC:3620]	protein coding	1.22	7.50	21.31	0.00	0.00
ENSG00000211964	IGHV3-48	immunoglobulin heavy variable 3-48 [Source:HGNC Symbol;Acc:HGNC:5606]	IG V gene	1.22	3.42	35.12	0.00	0.00
ENSG00000211673	IGLV3-1	immunoglobulin lambda variable 3-1 [Source:HGNC Symbol;Acc:HGNC:5896]	IG V gene	1.21	3.73	22.46	0.00	0.00
ENSG00000157551	KCNJ15	potassium voltage-gated channel subfamily J member 15 [Source:HGNC Symbol;Acc:HGNC:6261]	protein coding	1.18	4.29	27.11	0.00	0.00
ENSG00000211679	IGLC3	immunoglobulin lambda constant 3 (Kern-Oz+ marker) [Source:HGNC Symbol;Acc:HGNC:5857]	IG C gene	1.15	5.46	44.96	0.00	0.00
ENSG00000241351	IGKV3-11	immunoglobulin kappa variable 3-11 [Source:HGNC Symbol;Acc:HGNC:5815]	IG V gene	1.12	3.55	32.94	0.00	0.00

Ensembl Gene ID	Ensembl Gene Name	Ensembl Gene Description	Gene Type	Log_2 Fold Change (Day 8 vs. pre-treatment)	Average Log_2 CPM	Likelihood Ratio Test Statistic	P-Value	FDR Adjusted P-Value
ENSG00000170476	MZB1	marginal zone B and B1 cell specific protein [Source:HGNC Symbol;Acc:HGNC:30125]	protein coding	1.10	4.70	42.24	0.00	0.00
ENSG00000211943	IGHV3-15	immunoglobulin heavy variable 3-15 [Source:HGNC Symbol;Acc:HGNC:5582]	IG V gene	1.10	3.12	13.67	0.00	0.04
ENSG00000149131	SERPING1	serpin family G member 1 [Source:HGNC Symbol;Acc:HGNC:1228]	protein coding	1.08	3.16	14.90	0.00	0.02
ENSG00000119922	IFIT2	interferon induced protein with tetratricopeptide repeats 2 [Source:HGNC Symbol;Acc:HGNC:5409]	protein coding	1.08	6.20	34.76	0.00	0.00
ENSG00000180871	CXCR2	C-X-C motif chemokine receptor 2 [Source:HGNC Symbol;Acc:HGNC:6027]	protein coding	1.02	7.02	17.93	0.00	0.01
ENSG00000048462	TNFRSF17	TNF receptor superfamily member 17 [Source:HGNC Symbol;Acc:HGNC:11913]	protein coding	1.01	2.45	15.79	0.00	0.02
ENSG00000185745	IFIT1	interferon induced protein with tetratricopeptide repeats 1 [Source:HGNC Symbol;Acc:HGNC:5407]	protein coding	1.00	4.99	22.03	0.00	0.00
ENSG00000101057	MYBL2	MYB proto-oncogene like 2 [Source:HGNC Symbol;Acc:HGNC:7548]	protein coding	1.00	2.47	16.41	0.00	0.01
ENSG00000138772	ANXA3	annexin A3 [Source:HGNC Symbol;Acc:HGNC:541]	protein coding	0.99	2.62	18.37	0.00	0.01
ENSG00000132465	JCHAIN	joining chain of multimeric IgA and IgM [Source:HGNC Symbol;Acc:HGNC:5713]	protein coding	0.99	7.80	37.12	0.00	0.00
ENSG00000123700	KCNJ2	potassium voltage-gated channel subfamily J member 2 [Source:HGNC Symbol;Acc:HGNC:6263]	protein coding	0.95	3.68	15.84	0.00	0.02
ENSG00000232176			processed pseudogene	0.94	4.22	20.72	0.00	0.00
ENSG00000196549	MME	membrane metalloendopeptidase [Source:HGNC Symbol;Acc:HGNC:7154]	protein coding	0.93	5.21	15.05	0.00	0.02
ENSG00000004799	PDK4	pyruvate dehydrogenase kinase 4 [Source:HGNC Symbol;Acc:HGNC:8812]	protein coding	0.91	4.76	15.26	0.00	0.02
ENSG00000163993	S100P	S100 calcium binding protein P [Source:HGNC Symbol;Acc:HGNC:10504]	protein coding	0.88	3.38	14.70	0.00	0.02
ENSG00000133048	CHI3L1	chitinase 3 like 1 [Source:HGNC Symbol;Acc:HGNC:1932]	protein coding	0.87	3.65	18.47	0.00	0.01
ENSG00000181409	AATK	apoptosis associated tyrosine kinase [Source:HGNC Symbol;Acc:HGNC:21]	protein coding	0.85	3.53	13.99	0.00	0.03
ENSG00000119917	IFIT3	interferon induced protein with tetratricopeptide repeats 3 [Source:HGNC Symbol;Acc:HGNC:5411]	protein coding	0.83	5.91	15.11	0.00	0.02
ENSG00000008516	MMP25	matrix metallopeptidase 25 [Source:HGNC Symbol;Acc:HGNC:14246]	protein coding	0.81	5.79	20.43	0.00	0.00
ENSG00000103569	AQP9	aquaporin 9 [Source:HGNC Symbol;Acc:HGNC:643]	protein coding	0.80	6.56	23.14	0.00	0.00
ENSG00000239951	IGKV3-20	immunoglobulin kappa variable 3-20 [Source:HGNC Symbol;Acc:HGNC:5817]	IG V gene	0.76	4.11	17.00	0.00	0.01
ENSG00000211899	IGHM	immunoglobulin heavy constant mu [Source:HGNC Symbol;Acc:HGNC:5541]	IG C gene	0.73	9.31	23.32	0.00	0.00

Ensembl Gene ID	Ensembl Gene Name	Ensembl Gene Description	Gene Type	Log_2 Fold Change (Day 8 vs. pre-treatment)	Average Log_2 CPM	Likelihood Ratio Test Statistic	P-Value	FDR Adjusted P-Value
ENSG00000173110	HSPA6	heat shock protein family A (Hsp70) member 6 [Source:HGNC Symbol;Acc:HGNC:5239]	protein coding	0.73	4.28	13.99	0.00	0.03
ENSG00000124772	CPNE5	copine 5 [Source:HGNC Symbol;Acc:HGNC:2318]	protein coding	0.72	4.31	23.84	0.00	0.00
ENSG00000125538	IL1B	interleukin 1 beta [Source:HGNC Symbol;Acc:HGNC:5992]	protein coding	0.71	3.57	19.14	0.00	0.00
ENSG00000062282	DGAT2	diacylglycerol O-acyltransferase 2 [Source:HGNC Symbol;Acc:HGNC:16940]	protein coding	0.71	4.16	15.55	0.00	0.02
ENSG00000188897			protein coding	0.70	3.44	14.32	0.00	0.03
ENSG00000170962	PDGFD	platelet derived growth factor D [Source:HGNC Symbol;Acc:HGNC:30620]	protein coding	-0.69	3.76	16.20	0.00	0.01
ENSG00000183508	FAM46C	family with sequence similarity 46 member C [Source:HGNC Symbol;Acc:HGNC:24712]	protein coding	0.68	5.56	14.10	0.00	0.03
ENSG00000171101	SIGLEC17P	sialic acid binding Ig like lectin 17, pseudogene [Source:HGNC Symbol;Acc:HGNC:15604]	transcribed unprocessed pseudogene	-0.66	3.21	14.09	0.00	0.03
ENSG00000275302	CCL4	C-C motif chemokine ligand 4 [Source:HGNC Symbol;Acc:HGNC:10630]	protein coding	-0.65	4.65	18.29	0.00	0.01
ENSG00000211592	IGKC	immunoglobulin kappa constant [Source:HGNC Symbol;Acc:HGNC:5716]	IG C gene	0.63	8.74	26.15	0.00	0.00
ENSG00000142089	IFITM3	interferon induced transmembrane protein 3 [Source:HGNC Symbol;Acc:HGNC:5414]	protein coding	0.61	6.01	17.46	0.00	0.01
ENSG00000171049	FPR2	formyl peptide receptor 2 [Source:HGNC Symbol;Acc:HGNC:3827]	protein coding	0.59	5.69	13.12	0.00	0.05
ENSG00000110848	CD69	CD69 molecule [Source:HGNC Symbol;Acc:HGNC:1694]	protein coding	-0.59	5.58	20.25	0.00	0.00

Table 24: Genes differentially expressed at Day 8 compared to pre-treatment (PBMC, Trivalent Influenza Vaccine). Sorted by descending absolute log_2 fold change (Day 8 vs. pre-treatment). Gene model summaries and annotations are based on Ensembl Version 87.

Ensembl Gene ID	Ensembl Gene Name	Ensembl Gene Description	Gene Type	Log_2 Fold Change (Day 9 vs. pre-treatment)	Average Log_2 CPM	Likelihood Ratio Test Statistic	P-Value	FDR Adjusted P-Value
ENSG00000211653	IGLV1-40	immunoglobulin lambda variable 1-40 [Source:HGNC Symbol;Acc:HGNC:5877]	IG V gene	1.90	4.03	24.64	0.00	0.00
ENSG00000211896	IGHG1	immunoglobulin heavy constant gamma 1 (G1m marker) [Source:HGNC Symbol;Acc:HGNC:5525]	IG C gene	1.56	8.33	48.17	0.00	0.00
ENSG00000211670	IGLV3-9	immunoglobulin lambda variable 3-9 (gene/pseudogene) [Source:HGNC Symbol;Acc:HGNC:5918]	IG V gene	1.55	2.19	17.29	0.00	0.03
ENSG00000158578	ALAS2	5'-aminolevulinate synthase 2 [Source:HGNC Symbol;Acc:HGNC:397]	protein coding	1.37	3.05	27.63	0.00	0.00

Ensembl Gene ID	Ensembl Gene Name	Ensembl Gene Description	Gene Type	Log_2 Fold Change (Day 9 vs. pre-treatment)	Average Log_2 CPM	Likelihood Ratio Test Statistic	P-Value	FDR Adjusted P-Value
ENSG00000211677	IGLC2	immunoglobulin lambda constant 2 [Source:HGNC Symbol;Acc:HGNC:5856]	IG C gene	1.20	6.39	19.91	0.00	0.01
ENSG00000224373	IGHV4-59	immunoglobulin heavy variable 4-59 [Source:HGNC Symbol;Acc:HGNC:5654]	IG V gene	1.15	2.87	19.21	0.00	0.01
ENSG00000253755	IGHGP	immunoglobulin heavy constant gamma P (non-functional) [Source:HGNC Symbol;Acc:HGNC:5529]	IG C pseudogene	1.13	2.92	20.06	0.00	0.01
ENSG00000188536	HBA2	hemoglobin subunit alpha 2 [Source:HGNC Symbol;Acc:HGNC:4824]	protein coding	1.01	5.94	35.66	0.00	0.00
ENSG00000211964	IGHV3-48	immunoglobulin heavy variable 3-48 [Source:HGNC Symbol;Acc:HGNC:5606]	IG V gene	0.93	3.49	21.60	0.00	0.01
ENSG00000004799	PDK4	pyruvate dehydrogenase kinase 4 [Source:HGNC Symbol;Acc:HGNC:8812]	protein coding	0.93	4.74	28.15	0.00	0.00
ENSG00000206172	HBA1	hemoglobin subunit alpha 1 [Source:HGNC Symbol;Acc:HGNC:4823]	protein coding	0.93	4.86	18.54	0.00	0.02
ENSG00000244734	HBB	hemoglobin subunit beta [Source:HGNC Symbol;Acc:HGNC:4827]	protein coding	0.75	9.64	31.93	0.00	0.00

Table 25: Genes differentially expressed at Day 9 compared to pre-treatment (PBMC, Trivalent Influenza Vaccine). Sorted by descending absolute log_2 fold change (Day 9 vs. pre-treatment). Gene model summaries and annotations are based on Ensembl Version 87.

Ensembl Gene ID	Ensembl Gene Name	Ensembl Gene Description	Gene Type	Log_2 Fold Change (Day 10 vs. pre-treatment)	Average Log_2 CPM	Likelihood Ratio Test Statistic	P-Value	FDR Adjusted P-Value
ENSG00000211653	IGLV1-40	immunoglobulin lambda variable 1-40 [Source:HGNC Symbol;Acc:HGNC:5877]	IG V gene	1.60	3.64	24.44	0.00	0.00
ENSG00000124102	PI3	peptidase inhibitor 3 [Source:HGNC Symbol;Acc:HGNC:8947]	protein coding	1.46	1.63	15.87	0.00	0.02
ENSG00000223609	HBD	hemoglobin subunit delta [Source:HGNC Symbol;Acc:HGNC:4829]	protein coding	1.46	1.28	15.91	0.00	0.02
ENSG00000158578	ALAS2	5'-aminolevulinate synthase 2 [Source:HGNC Symbol;Acc:HGNC:397]	protein coding	1.44	3.06	32.92	0.00	0.00
ENSG00000162551	ALPL	alkaline phosphatase, liver/bone/kidney [Source:HGNC Symbol;Acc:HGNC:438]	protein coding	1.38	4.14	35.71	0.00	0.00
ENSG00000188536	HBA2	hemoglobin subunit alpha 2 [Source:HGNC Symbol;Acc:HGNC:4824]	protein coding	1.30	6.26	29.29	0.00	0.00
ENSG00000206172	HBA1	hemoglobin subunit alpha 1 [Source:HGNC Symbol;Acc:HGNC:4823]	protein coding	1.26	5.17	25.63	0.00	0.00
ENSG00000211670	IGLV3-9	immunoglobulin lambda variable 3-9 (gene/pseudogene) [Source:HGNC Symbol;Acc:HGNC:5918]	IG V gene	1.19	1.64	17.68	0.00	0.01
ENSG00000173535	TNFRSF10C	TNF receptor superfamily member 10c [Source:HGNC Symbol;Acc:HGNC:11906]	protein coding	1.16	5.00	39.80	0.00	0.00
ENSG00000004939	SLC4A1	solute carrier family 4 member 1 (Diego blood group) [Source:HGNC Symbol;Acc:HGNC:11027]	protein coding	1.16	2.88	15.80	0.00	0.02

Ensembl Gene ID	Ensembl Gene Name	Ensembl Gene Description	Gene Type	Log_2 Fold Change (Day 10 vs. pre-treatment)	Average Log_2 CPM	Likelihood Ratio Test Statistic	P-Value	FDR Adjusted P-Value
ENSG00000162747	FCGR3B	Fc fragment of IgG receptor IIIb [Source:HGNC Symbol;Acc:HGNC:3620]	protein coding	1.15	7.14	45.32	0.00	0.00
ENSG00000163464	CXCR1	C-X-C motif chemokine receptor 1 [Source:HGNC Symbol;Acc:HGNC:6026]	protein coding	1.09	5.85	56.18	0.00	0.00
ENSG00000126262	FFAR2	free fatty acid receptor 2 [Source:HGNC Symbol;Acc:HGNC:4501]	protein coding	1.09	4.07	27.19	0.00	0.00
ENSG00000183762	KREMEN1	kringle containing transmembrane protein 1 [Source:HGNC Symbol;Acc:HGNC:17550]	protein coding	1.08	1.94	14.00	0.00	0.04
ENSG00000244734	HBB	hemoglobin subunit beta [Source:HGNC Symbol;Acc:HGNC:4827]	protein coding	1.07	9.90	35.63	0.00	0.00
ENSG00000182782	HCAR2	hydroxycarboxylic acid receptor 2 [Source:HGNC Symbol;Acc:HGNC:24827]	protein coding	1.05	2.23	24.30	0.00	0.00
ENSG00000180871	CXCR2	C-X-C motif chemokine receptor 2 [Source:HGNC Symbol;Acc:HGNC:6027]	protein coding	0.97	6.77	45.13	0.00	0.00
ENSG00000140932	CMTM2	CKLF like MARVEL transmembrane domain containing 2 [Source:HGNC Symbol;Acc:HGNC:19173]	protein coding	0.95	2.78	24.87	0.00	0.00
ENSG00000171236	LRG1	leucine rich alpha-2-glycoprotein 1 [Source:HGNC Symbol;Acc:HGNC:29480]	protein coding	0.95	3.16	39.46	0.00	0.00
ENSG00000182885	ADGRG3	adhesion G protein-coupled receptor G3 [Source:HGNC Symbol;Acc:HGNC:13728]	protein coding	0.92	3.66	25.03	0.00	0.00
ENSG00000133048	CHI3L1	chitinase 3 like 1 [Source:HGNC Symbol;Acc:HGNC:1932]	protein coding	0.92	3.51	35.76	0.00	0.00
ENSG00000108244	KRT23	keratin 23 [Source:HGNC Symbol;Acc:HGNC:6438]	protein coding	0.92	2.17	13.25	0.00	0.05
ENSG00000196549	MME	membrane metalloendopeptidase [Source:HGNC Symbol;Acc:HGNC:7154]	protein coding	0.88	5.02	25.52	0.00	0.00
ENSG00000004799	PDK4	pyruvate dehydrogenase kinase 4 [Source:HGNC Symbol;Acc:HGNC:8812]	protein coding	0.85	4.78	14.63	0.00	0.03
ENSG00000157551	KCNJ15	potassium voltage-gated channel subfamily J member 15 [Source:HGNC Symbol;Acc:HGNC:6261]	protein coding	0.82	4.08	23.35	0.00	0.00
ENSG00000186529	CYP4F3	cytochrome P450 family 4 subfamily F member 3 [Source:HGNC Symbol;Acc:HGNC:2646]	protein coding	0.80	4.20	16.38	0.00	0.02
ENSG00000008516	MMP25	matrix metallopeptidase 25 [Source:HGNC Symbol;Acc:HGNC:14246]	protein coding	0.79	5.73	28.83	0.00	0.00
ENSG00000148926	ADM	adrenomedullin [Source:HGNC Symbol;Acc:HGNC:259]	protein coding	0.78	2.37	15.75	0.00	0.02
ENSG00000173868	PHOSPHO1	phosphoethanolamine/phosphocholine phosphatase [Source:HGNC Symbol;Acc:HGNC:16815]	protein coding	0.76	4.15	15.42	0.00	0.02
ENSG00000119922	IFIT2	interferon induced protein with tetratricopeptide repeats 2 [Source:HGNC Symbol;Acc:HGNC:5409]	protein coding	0.65	5.77	40.22	0.00	0.00
ENSG00000239653	PSMD6-AS2	PSMD6 antisense RNA 2 [Source:HGNC Symbol;Acc:HGNC:44125]	antisense	-0.65	2.99	13.30	0.00	0.05
ENSG00000181409	AATK	apoptosis associated tyrosine kinase [Source:HGNC Symbol;Acc:HGNC:21]	protein coding	0.63	3.55	15.80	0.00	0.02
ENSG00000062282	DGAT2	diacylglycerol O-acyltransferase 2 [Source:HGNC Symbol;Acc:HGNC:16940]	protein coding	0.63	4.09	24.93	0.00	0.00

Ensembl Gene ID	Ensembl Gene Name	Ensembl Gene Description	Gene Type	Log_2 Fold Change (Day 10 vs. pre-treatment)	Average Log_2 CPM	Likelihood Ratio Test Statistic	P-Value	FDR Adjusted P-Value
ENSG00000257335	MGAM	maltase-glucoamylase [Source:HGNC Symbol;Acc:HGNC:7043]	protein coding	0.62	5.49	15.35	0.00	0.02
ENSG00000188897			protein coding	0.62	3.42	22.35	0.00	0.00

Table 26: Genes differentially expressed at Day 10 compared to pre-treatment (PBMC, Trivalent Influenza Vaccine). Sorted by descending absolute log_2 fold change (Day 10 vs. pre-treatment). Gene model summaries and annotations are based on Ensembl Version 87.

Cluster ID	Cluster Size	Gene ID	Gene Name	Gene Description	Log_2 FC TIV
BCLTP1-001	5	ENSG00000117228	GBP1	guanylate binding protein 1 [Source:HGNC Symbol;Acc:HGNC:4182]	0.89
BCLTP1-001	5	ENSG00000162645	GBP2	guanylate binding protein 2 [Source:HGNC Symbol;Acc:HGNC:4183]	0.61
BCLTP1-001	5	ENSG00000103226	NOMO3	NODAL modulator 3 [Source:HGNC Symbol;Acc:HGNC:25242]	0.13
BCLTP1-001	5	ENSG00000167325	RRM1	ribonucleotide reductase catalytic subunit M1 [Source:HGNC Symbol;Acc:HGNC:10451]	0.18
BCLTP1-001	5	ENSG00000115415	STAT1	signal transducer and activator of transcription 1 [Source:HGNC Symbol;Acc:HGNC:11362]	0.70

Table 27: Co-expressed gene clusters (B Cells, Day 1)

Cluster ID	Cluster Size	Gene ID	Gene Name	Gene Description	Log_2 FC TIV
BCLTP2-001	4	ENSG00000211599	IGKV5-2	immunoglobulin kappa variable 5-2 [Source:HGNC Symbol;Acc:HGNC:5835]	0.19
BCLTP2-001	4	ENSG00000186594	MIR22HG	MIR22 host gene [Source:HGNC Symbol;Acc:HGNC:28219]	0.53
BCLTP2-001	4	ENSG00000004799	PDK4	pyruvate dehydrogenase kinase 4 [Source:HGNC Symbol;Acc:HGNC:8812]	1.00
BCLTP2-001	4	ENSG00000004866	ST7	suppression of tumorigenicity 7 [Source:HGNC Symbol;Acc:HGNC:11351]	0.26
BCLTP2-002	6	ENSG00000206172	HBA1	hemoglobin subunit alpha 1 [Source:HGNC Symbol;Acc:HGNC:4823]	-0.78
BCLTP2-002	6	ENSG00000188536	HBA2	hemoglobin subunit alpha 2 [Source:HGNC Symbol;Acc:HGNC:4824]	-0.81
BCLTP2-002	6	ENSG00000244734	HBB	hemoglobin subunit beta [Source:HGNC Symbol;Acc:HGNC:4827]	-0.87
BCLTP2-002	6	ENSG00000166851	PLK1	polo like kinase 1 [Source:HGNC Symbol;Acc:HGNC:9077]	-0.09
BCLTP2-002	6	ENSG00000080546	SESN1	sestrin 1 [Source:HGNC Symbol;Acc:HGNC:21595]	-0.26
BCLTP2-002	6	ENSG00000196263	ZNF471	zinc finger protein 471 [Source:HGNC Symbol;Acc:HGNC:23226]	-0.15

Table 28: Co-expressed gene clusters (B Cells, Day 2)

Cluster ID	Cluster Size	Gene ID	Gene Name	Gene Description	Log_2 FC TIV
BCLTP3-001	2	ENSG00000010310	GIPR	gastric inhibitory polypeptide receptor [Source:HGNC Symbol;Acc:HGNC:4271]	-0.53
BCLTP3-001	2	ENSG00000171223	JUNB	JunB proto-oncogene, AP-1 transcription factor subunit [Source:HGNC Symbol;Acc:HGNC:6205]	-0.75
BCLTP3-002	7	ENSG00000011426	ANLN	anillin actin binding protein [Source:HGNC Symbol;Acc:HGNC:14082]	0.64
BCLTP3-002	7	ENSG00000007968	E2F2	E2F transcription factor 2 [Source:HGNC Symbol;Acc:HGNC:3114]	0.24
BCLTP3-002	7	ENSG00000244116	IGKV2-28	immunoglobulin kappa variable 2-28 [Source:HGNC Symbol;Acc:HGNC:5783]	0.36
BCLTP3-002	7	ENSG00000182481	KPNA2	karyopherin subunit alpha 2 [Source:HGNC Symbol;Acc:HGNC:6395]	0.41
BCLTP3-002	7	ENSG00000144182	LIPT1	lipoyltransferase 1 [Source:HGNC Symbol;Acc:HGNC:29569]	0.62
BCLTP3-002	7	ENSG00000104738	MCM4	minichromosome maintenance complex component 4 [Source:HGNC Symbol;Acc:HGNC:6947]	0.19
BCLTP3-002	7	ENSG00000131747	TOP2A	topoisomerase (DNA) II alpha [Source:HGNC Symbol;Acc:HGNC:11989]	0.44
BCLTP3-003	4	ENSG00000241666			-0.59
BCLTP3-003	4	ENSG00000168209	DDIT4	DNA damage inducible transcript 4 [Source:HGNC Symbol;Acc:HGNC:24944]	-1.00
BCLTP3-003	4	ENSG00000080546	SESN1	sestrin 1 [Source:HGNC Symbol;Acc:HGNC:21595]	-0.34

Cluster ID	Cluster Size	Gene ID	Gene Name	Gene Description	Log_2 FC TIV
BCLTP3-003	4	ENSG00000088826	SMOX	spermine oxidase [Source:HGNC Symbol;Acc:HGNC:15862]	-0.32
BCLTP3-004	5	ENSG00000227155			-0.59
BCLTP3-004	5	ENSG00000255026			-0.49
BCLTP3-004	5	ENSG00000271533			-0.55
BCLTP3-004	5	ENSG00000274272			-0.35
BCLTP3-004	5	ENSG00000280138			-0.61
BCLTP3-005	8	ENSG00000278897			-0.52
BCLTP3-005	8	ENSG00000279759			-0.59
BCLTP3-005	8	ENSG00000181800	CELF2-AS1	CELF2 antisense RNA 1 [Source:HGNC Symbol;Acc:HGNC:23515]	-0.76
BCLTP3-005	8	ENSG00000279192	PWAR5	Prader Willi/Angelman region RNA 5 [Source:HGNC Symbol;Acc:HGNC:30090]	-0.68
BCLTP3-005	8	ENSG00000180747	SMG1P3	SMG1P3, nonsense mediated mRNA decay associated PI3K related kinase pseudogene 3 [Source:HGNC Symbol;Acc:HGNC:49860]	-0.54
BCLTP3-005	8	ENSG00000100154	TTC28	tetratricopeptide repeat domain 28 [Source:HGNC Symbol;Acc:HGNC:29179]	-0.28
BCLTP3-005	8	ENSG00000181722	ZBTB20	zinc finger and BTB domain containing 20 [Source:HGNC Symbol;Acc:HGNC:13503]	-0.62
BCLTP3-005	8	ENSG00000197714	ZNF460	zinc finger protein 460 [Source:HGNC Symbol;Acc:HGNC:21628]	-0.81
BCLTP3-006	7	ENSG00000206172	HBA1	hemoglobin subunit alpha 1 [Source:HGNC Symbol;Acc:HGNC:4823]	-1.50
BCLTP3-006	7	ENSG00000188536	HBA2	hemoglobin subunit alpha 2 [Source:HGNC Symbol;Acc:HGNC:4824]	-1.77
BCLTP3-006	7	ENSG00000244734	HBB	hemoglobin subunit beta [Source:HGNC Symbol;Acc:HGNC:4827]	-1.64
BCLTP3-006	7	ENSG00000220008	LINGO3	leucine rich repeat and Ig domain containing 3 [Source:HGNC Symbol;Acc:HGNC:21206]	-0.44
BCLTP3-006	7	ENSG00000185522	LMNTD2	lamin tail domain containing 2 [Source:HGNC Symbol;Acc:HGNC:28561]	-0.43
BCLTP3-006	7	ENSG00000120725	SIL1	SIL1 nucleotide exchange factor [Source:HGNC Symbol;Acc:HGNC:24624]	-0.13
BCLTP3-006	7	ENSG00000196263	ZNF471	zinc finger protein 471 [Source:HGNC Symbol;Acc:HGNC:23226]	-0.31
BCLTP3-007	8	ENSG00000280063			-0.21
BCLTP3-007	8	ENSG00000110848	CD69	CD69 molecule [Source:HGNC Symbol;Acc:HGNC:1694]	-0.84
BCLTP3-007	8	ENSG00000161960	EIF4A1	eukaryotic translation initiation factor 4A1 [Source:HGNC Symbol;Acc:HGNC:3282]	-0.60
BCLTP3-007	8	ENSG00000178127	NDUFV2	NADH:ubiquinone oxidoreductase core subunit V2 [Source:HGNC Symbol;Acc:HGNC:7717]	-0.68
BCLTP3-007	8	ENSG00000100906	NFKBIA	NFKB inhibitor alpha [Source:HGNC Symbol;Acc:HGNC:7797]	-0.63
BCLTP3-007	8	ENSG00000090104	RGS1	regulator of G-protein signaling 1 [Source:HGNC Symbol;Acc:HGNC:9991]	-0.56
BCLTP3-007	8	ENSG00000116741	RGS2	regulator of G-protein signaling 2 [Source:HGNC Symbol;Acc:HGNC:9998]	-0.54
BCLTP3-007	8	ENSG00000239264	TXNDC5	thioredoxin domain containing 5 [Source:HGNC Symbol;Acc:HGNC:21073]	-0.27

Table 29: Co-expressed gene clusters (B Cells, Day 3)

Cluster ID	Cluster Size	Gene ID	Gene Name	Gene Description	Log_2 FC TIV
BCLTP4-001	2	ENSG00000074416	MGLL	monoglyceride lipase [Source:HGNC Symbol;Acc:HGNC:17038]	0.18
BCLTP4-001	2	ENSG00000149516	MS4A3	membrane spanning 4-domains A3 [Source:HGNC Symbol;Acc:HGNC:7317]	0.81
BCLTP4-002	2	ENSG00000138166	DUSP5	dual specificity phosphatase 5 [Source:HGNC Symbol;Acc:HGNC:3071]	0.53

Cluster ID	Cluster Size	Gene ID	Gene Name	Gene Description	Log_2 FC TIV
BCLTP4-002	2	ENSG00000170345	FOS	Fos proto-oncogene, AP-1 transcription factor subunit [Source:HGNC Symbol;Acc:HGNC:3796]	1.01
BCLTP4-003	8	ENSG00000260948			-0.24
BCLTP4-003	8	ENSG00000233806	LINC01237	long intergenic non-protein coding RNA 1237 [Source:HGNC Symbol;Acc:HGNC:49793]	-0.34
BCLTP4-003	8	ENSG00000178127	NDUFV2	NADH:ubiquinone oxidoreductase core subunit V2 [Source:HGNC Symbol;Acc:HGNC:7717]	-0.33
BCLTP4-003	8	ENSG00000279192	PWAR5	Prader Willi/Angelman region RNA 5 [Source:HGNC Symbol;Acc:HGNC:30090]	-0.61
BCLTP4-003	8	ENSG00000180747	SMG1P3	SMG1P3, nonsense mediated mRNA decay associated PI3K related kinase pseudogene 3 [Source:HGNC Symbol;Acc:HGNC:49860]	-0.67
BCLTP4-003	8	ENSG00000006638	TBXA2R	thromboxane A2 receptor [Source:HGNC Symbol;Acc:HGNC:11608]	-0.14
BCLTP4-003	8	ENSG00000181722	ZBTB20	zinc finger and BTB domain containing 20 [Source:HGNC Symbol;Acc:HGNC:13503]	-0.67
BCLTP4-003	8	ENSG00000197714	ZNF460	zinc finger protein 460 [Source:HGNC Symbol;Acc:HGNC:21628]	-0.76
BCLTP4-004	8	ENSG00000274272			-0.11
BCLTP4-004	8	ENSG00000142675	CNKSR1	connector enhancer of kinase suppressor of Ras 1 [Source:HGNC Symbol;Acc:HGNC:19700]	-0.07
BCLTP4-004	8	ENSG00000206172	HBA1	hemoglobin subunit alpha 1 [Source:HGNC Symbol;Acc:HGNC:4823]	-1.31
BCLTP4-004	8	ENSG00000188536	HBA2	hemoglobin subunit alpha 2 [Source:HGNC Symbol;Acc:HGNC:4824]	-1.31
BCLTP4-004	8	ENSG00000244734	HBB	hemoglobin subunit beta [Source:HGNC Symbol;Acc:HGNC:4827]	-1.36
BCLTP4-004	8	ENSG00000253691	IGKV2OR22-4	immunoglobulin kappa variable 2/OR22-4 (pseudogene) [Source:HGNC Symbol;Acc:HGNC:5813]	-0.27
BCLTP4-004	8	ENSG00000187922	LCN10	lipocalin 10 [Source:HGNC Symbol;Acc:HGNC:20892]	-0.22
BCLTP4-004	8	ENSG00000168701	TMEM208	transmembrane protein 208 [Source:HGNC Symbol;Acc:HGNC:25015]	-0.09

Table 30: Co-expressed gene clusters (B Cells, Day 4)

Cluster ID	Cluster Size	Gene ID	Gene Name	Gene Description	Log_2 FC TIV
BCLTP5-001	2	ENSG00000280411	IGHV1-69-2	immunoglobulin heavy variable 1-69-2 [Source:HGNC Symbol;Acc:HGNC:5562]	0.99
BCLTP5-001	2	ENSG00000135069	PSAT1	phosphoserine aminotransferase 1 [Source:HGNC Symbol;Acc:HGNC:19129]	0.69
BCLTP5-002	2	ENSG00000112984	KIF20A	kinesin family member 20A [Source:HGNC Symbol;Acc:HGNC:9787]	1.55
BCLTP5-002	2	ENSG00000088325	TPX2	TPX2, microtubule nucleation factor [Source:HGNC Symbol;Acc:HGNC:1249]	1.47
BCLTP5-003	2	ENSG00000101057	MYBL2	MYB proto-oncogene like 2 [Source:HGNC Symbol;Acc:HGNC:7548]	1.49
BCLTP5-003	2	ENSG00000085840	ORC1	origin recognition complex subunit 1 [Source:HGNC Symbol;Acc:HGNC:8487]	1.49
BCLTP5-004	2	ENSG00000007968	E2F2	E2F transcription factor 2 [Source:HGNC Symbol;Acc:HGNC:3114]	0.87
BCLTP5-004	2	ENSG00000227203	SUB1P1	SUB1 homolog, transcriptional regulator pseudogene 1 [Source:HGNC Symbol;Acc:HGNC:32300]	0.80
BCLTP5-005	3	ENSG00000140525	FANCI	Fanconi anemia complementation group I [Source:HGNC Symbol;Acc:HGNC:25568]	0.87
BCLTP5-005	3	ENSG00000128228	SDF2L1	stromal cell derived factor 2 like 1 [Source:HGNC Symbol;Acc:HGNC:10676]	1.20
BCLTP5-005	3	ENSG00000114850	SSR3	signal sequence receptor subunit 3 [Source:HGNC Symbol;Acc:HGNC:11325]	0.80
BCLTP5-006	2	ENSG00000110917	MLEC	malectin [Source:HGNC Symbol;Acc:HGNC:28973]	0.68

Cluster ID	Cluster Size	Gene ID	Gene Name	Gene Description	Log_2 FC TIV
BCLTP5-006	2	ENSG00000070214	SLC44A1	solute carrier family 44 member 1 [Source:HGNC Symbol;Acc:HGNC:18798]	0.94
BCLTP5-007	2	ENSG00000242076	IGKV1-33	immunoglobulin kappa variable 1-33 [Source:HGNC Symbol;Acc:HGNC:5737]	0.95
BCLTP5-007	2	ENSG00000147649	MTDH	metadherin [Source:HGNC Symbol;Acc:HGNC:29608]	0.64
BCLTP5-008	2	ENSG00000138778	CENPE	centromere protein E [Source:HGNC Symbol;Acc:HGNC:1856]	1.37
BCLTP5-008	2	ENSG00000122952	ZWINT	ZW10 interacting kinetochore protein [Source:HGNC Symbol;Acc:HGNC:13195]	1.59
BCLTP5-009	3	ENSG00000154723	ATP5J	ATP synthase, H+ transporting, mitochondrial Fo complex subunit F6 [Source:HGNC Symbol;Acc:HGNC:847]	0.61
BCLTP5-009	3	ENSG00000127022	CANX	calnexin [Source:HGNC Symbol;Acc:HGNC:1473]	0.63
BCLTP5-009	3	ENSG00000196189	SEMA4A	semaphorin 4A [Source:HGNC Symbol;Acc:HGNC:10729]	0.89
BCLTP5-010	3	ENSG00000094804	CDC6	cell division cycle 6 [Source:HGNC Symbol;Acc:HGNC:1744]	1.47
BCLTP5-010	3	ENSG00000132646	PCNA	proliferating cell nuclear antigen [Source:HGNC Symbol;Acc:HGNC:8729]	0.70
BCLTP5-010	3	ENSG00000071539	TRIP13	thyroid hormone receptor interactor 13 [Source:HGNC Symbol;Acc:HGNC:12307]	1.57
BCLTP5-011	2	ENSG00000126787	DLGAP5	DLG associated protein 5 [Source:HGNC Symbol;Acc:HGNC:16864]	1.52
BCLTP5-011	2	ENSG00000154839	SKA1	spindle and kinetochore associated complex subunit 1 [Source:HGNC Symbol;Acc:HGNC:28109]	1.04
BCLTP5-012	4	ENSG00000235162	C12orf75	chromosome 12 open reading frame 75 [Source:HGNC Symbol;Acc:HGNC:35164]	0.87
BCLTP5-012	4	ENSG00000168496	FEN1	flap structure-specific endonuclease 1 [Source:HGNC Symbol;Acc:HGNC:3650]	0.96
BCLTP5-012	4	ENSG00000259706	HSP90B2P	heat shock protein 90 beta family member 2, pseudogene [Source:HGNC Symbol;Acc:HGNC:12099]	1.05
BCLTP5-012	4	ENSG00000145050	MANF	mesencephalic astrocyte derived neurotrophic factor [Source:HGNC Symbol;Acc:HGNC:15461]	1.08
BCLTP5-013	3	ENSG00000258572			1.05
BCLTP5-013	3	ENSG00000166598	HSP90B1	heat shock protein 90 beta family member 1 [Source:HGNC Symbol;Acc:HGNC:12028]	1.11
BCLTP5-013	3	ENSG00000173848	NET1	neuroepithelial cell transforming 1 [Source:HGNC Symbol;Acc:HGNC:14592]	1.11
BCLTP5-014	2	ENSG00000089685	BIRC5	baculoviral IAP repeat containing 5 [Source:HGNC Symbol;Acc:HGNC:593]	1.67
BCLTP5-014	2	ENSG00000117399	CDC20	cell division cycle 20 [Source:HGNC Symbol;Acc:HGNC:1723]	1.63
BCLTP5-015	3	ENSG00000105974	CAV1	caveolin 1 [Source:HGNC Symbol;Acc:HGNC:1527]	1.55
BCLTP5-015	3	ENSG00000171241	SHCBP1	SHC binding and spindle associated 1 [Source:HGNC Symbol;Acc:HGNC:29547]	1.56
BCLTP5-015	3	ENSG00000123416	TUBA1B	tubulin alpha 1b [Source:HGNC Symbol;Acc:HGNC:18809]	0.65
BCLTP5-016	2	ENSG00000137563	GGH	gamma-glutamyl hydrolase [Source:HGNC Symbol;Acc:HGNC:4248]	1.36
BCLTP5-016	2	ENSG00000090889	KIF4A	kinesin family member 4A [Source:HGNC Symbol;Acc:HGNC:13339]	1.16
BCLTP5-017	3	ENSG00000184661	CDCA2	cell division cycle associated 2 [Source:HGNC Symbol;Acc:HGNC:14623]	1.84
BCLTP5-017	3	ENSG00000072571	HMMR	hyaluronan mediated motility receptor [Source:HGNC Symbol;Acc:HGNC:5012]	1.31
BCLTP5-017	3	ENSG00000108106	UBE2S	ubiquitin conjugating enzyme E2 S [Source:HGNC Symbol;Acc:HGNC:17895]	0.83
BCLTP5-018	3	ENSG00000241351	IGKV3-11	immunoglobulin kappa variable 3-11 [Source:HGNC Symbol;Acc:HGNC:5815]	0.61
BCLTP5-018	3	ENSG00000076003	MCM6	minichromosome maintenance complex component 6 [Source:HGNC Symbol;Acc:HGNC:6949]	0.91
BCLTP5-018	3	ENSG00000103257	SLC7A5	solute carrier family 7 member 5 [Source:HGNC Symbol;Acc:HGNC:11063]	0.94
BCLTP5-019	2	ENSG00000169679	BUB1	BUB1 mitotic checkpoint serine/threonine kinase [Source:HGNC Symbol;Acc:HGNC:1148]	1.63
BCLTP5-019	2	ENSG00000064763	FAR2	fatty acyl-CoA reductase 2 [Source:HGNC Symbol;Acc:HGNC:25531]	0.68

Cluster ID	Cluster Size	Gene ID	Gene Name	Gene Description	Log_2 FC TIV
BCLTP5-020	5	ENSG00000138160	KIF11	kinesin family member 11 [Source:HGNC Symbol;Acc:HGNC:6388]	1.14
BCLTP5-020	5	ENSG00000104738	MCM4	minichromosome maintenance complex component 4 [Source:HGNC Symbol;Acc:HGNC:6947]	1.07
BCLTP5-020	5	ENSG00000109805	NCAPG	non-SMC condensin I complex subunit G [Source:HGNC Symbol;Acc:HGNC:24304]	1.61
BCLTP5-020	5	ENSG00000171848	RRM2	ribonucleotide reductase regulatory subunit M2 [Source:HGNC Symbol;Acc:HGNC:10452]	1.83
BCLTP5-020	5	ENSG00000077152	UBE2T	ubiquitin conjugating enzyme E2 T [Source:HGNC Symbol;Acc:HGNC:25009]	1.10
BCLTP5-021	2	ENSG00000119326	CTNNAL1	catenin alpha like 1 [Source:HGNC Symbol;Acc:HGNC:2512]	1.49
BCLTP5-021	2	ENSG00000198900	TOP1	topoisomerase (DNA) I [Source:HGNC Symbol;Acc:HGNC:11986]	0.59
BCLTP5-022	2	ENSG00000249096			0.85
BCLTP5-022	2	ENSG00000160712	IL6R	interleukin 6 receptor [Source:HGNC Symbol;Acc:HGNC:6019]	0.94
BCLTP5-023	5	ENSG00000138180	CEP55	centrosomal protein 55 [Source:HGNC Symbol;Acc:HGNC:1161]	1.34
BCLTP5-023	5	ENSG00000182481	KPNA2	karyopherin subunit alpha 2 [Source:HGNC Symbol;Acc:HGNC:6395]	0.90
BCLTP5-023	5	ENSG00000186594	MIR22HG	MIR22 host gene [Source:HGNC Symbol;Acc:HGNC:28219]	0.76
BCLTP5-023	5	ENSG00000137804	NUSAP1	nucleolar and spindle associated protein 1 [Source:HGNC Symbol;Acc:HGNC:18538]	1.03
BCLTP5-023	5	ENSG00000100219	XBP1	X-box binding protein 1 [Source:HGNC Symbol;Acc:HGNC:12801]	1.21
BCLTP5-024	3	ENSG00000135476	ESPL1	extra spindle pole bodies like 1, separase [Source:HGNC Symbol;Acc:HGNC:16856]	1.37
BCLTP5-024	3	ENSG00000075218	GTSE1	G2 and S-phase expressed 1 [Source:HGNC Symbol;Acc:HGNC:13698]	1.40
BCLTP5-024	3	ENSG00000142945	KIF2C	kinesin family member 2C [Source:HGNC Symbol;Acc:HGNC:6393]	1.17
BCLTP5-025	5	ENSG00000178999	AURKB	aurora kinase B [Source:HGNC Symbol;Acc:HGNC:11390]	1.44
BCLTP5-025	5	ENSG00000188486	H2AFX	H2A histone family member X [Source:HGNC Symbol;Acc:HGNC:4739]	0.81
BCLTP5-025	5	ENSG00000148773	MKI67	marker of proliferation Ki-67 [Source:HGNC Symbol;Acc:HGNC:7107]	1.45
BCLTP5-025	5	ENSG00000117632	STMN1	stathmin 1 [Source:HGNC Symbol;Acc:HGNC:6510]	0.69
BCLTP5-025	5	ENSG00000131747	TOP2A	topoisomerase (DNA) II alpha [Source:HGNC Symbol;Acc:HGNC:11989]	1.23
BCLTP5-026	2	ENSG00000153093	ACOXL	acyl-CoA oxidase-like [Source:HGNC Symbol;Acc:HGNC:25621]	2.42
BCLTP5-026	2	ENSG00000244575	IGKV1-27	immunoglobulin kappa variable 1-27 [Source:HGNC Symbol;Acc:HGNC:5735]	0.89
BCLTP5-027	2	ENSG00000105011	ASF1B	anti-silencing function 1B histone chaperone [Source:HGNC Symbol;Acc:HGNC:20996]	1.22
BCLTP5-027	2	ENSG00000138166	DUSP5	dual specificity phosphatase 5 [Source:HGNC Symbol;Acc:HGNC:3071]	1.43
BCLTP5-028	2	ENSG00000211946	IGHV3-20	immunoglobulin heavy variable 3-20 [Source:HGNC Symbol;Acc:HGNC:5585]	0.98
BCLTP5-028	2	ENSG00000167476	JSRP1	junctional sarcoplasmic reticulum protein 1 [Source:HGNC Symbol;Acc:HGNC:24963]	0.99
BCLTP5-029	3	ENSG00000113368	LMNB1	lamin B1 [Source:HGNC Symbol;Acc:HGNC:6637]	0.82
BCLTP5-029	3	ENSG00000166851	PLK1	polo like kinase 1 [Source:HGNC Symbol;Acc:HGNC:9077]	1.09
BCLTP5-029	3	ENSG00000068489	PRR11	proline rich 11 [Source:HGNC Symbol;Acc:HGNC:25619]	0.94
BCLTP5-030	2	ENSG00000143228	NUF2	NUF2, NDC80 kinetochore complex component [Source:HGNC Symbol;Acc:HGNC:14621]	1.06
BCLTP5-030	2	ENSG00000076382	SPAG5	sperm associated antigen 5 [Source:HGNC Symbol;Acc:HGNC:13452]	1.25
BCLTP5-031	2	ENSG00000259772			0.66
BCLTP5-031	2	ENSG00000163751	CPA3	carboxypeptidase A3 [Source:HGNC Symbol;Acc:HGNC:2298]	1.39
BCLTP5-032	5	ENSG00000113273	ARSB	arylsulfatase B [Source:HGNC Symbol;Acc:HGNC:714]	0.59

Cluster ID	Cluster Size	Gene ID	Gene Name	Gene Description	Log_2 FC TIV
BCLTP5-032	5	ENSG00000172115	CYCS	cytochrome c, somatic [Source:HGNC Symbol;Acc:HGNC:19986]	0.52
BCLTP5-032	5	ENSG00000164032	H2AFZ	H2A histone family member Z [Source:HGNC Symbol;Acc:HGNC:4741]	0.64
BCLTP5-032	5	ENSG00000074416	MGLL	monoglyceride lipase [Source:HGNC Symbol;Acc:HGNC:17038]	0.93
BCLTP5-032	5	ENSG00000132432	SEC61G	Sec61 translocon gamma subunit [Source:HGNC Symbol;Acc:HGNC:18277]	0.90
BCLTP5-033	2	ENSG00000143476	DTL	denticleless E3 ubiquitin protein ligase homolog [Source:HGNC Symbol;Acc:HGNC:30288]	1.68
BCLTP5-033	2	ENSG00000211625	IGKV3D-20	immunoglobulin kappa variable 3D-20 [Source:HGNC Symbol;Acc:HGNC:5825]	0.61
BCLTP5-034	5	ENSG00000173540	GMPPB	GDP-mannose pyrophosphorylase B [Source:HGNC Symbol;Acc:HGNC:22932]	0.84
BCLTP5-034	5	ENSG00000112378	PERP	PERP, TP53 apoptosis effector [Source:HGNC Symbol;Acc:HGNC:17637]	0.95
BCLTP5-034	5	ENSG00000183010	PYCR1	pyrroline-5-carboxylate reductase 1 [Source:HGNC Symbol;Acc:HGNC:9721]	0.88
BCLTP5-034	5	ENSG00000101310	SEC23B	Sec23 homolog B, coat complex II component [Source:HGNC Symbol;Acc:HGNC:10702]	0.50
BCLTP5-034	5	ENSG00000165409	TSHR	thyroid stimulating hormone receptor [Source:HGNC Symbol;Acc:HGNC:12373]	1.21
BCLTP5-035	6	ENSG00000248571			1.41
BCLTP5-035	6	ENSG00000121807	CCR2	C-C motif chemokine receptor 2 [Source:HGNC Symbol;Acc:HGNC:1603]	1.46
BCLTP5-035	6	ENSG00000107104	KANK1	KN motif and ankyrin repeat domains 1 [Source:HGNC Symbol;Acc:HGNC:19309]	1.32
BCLTP5-035	6	ENSG00000107719	PALD1	phosphatase domain containing, paladin 1 [Source:HGNC Symbol;Acc:HGNC:23530]	0.74
BCLTP5-035	6	ENSG00000114902	SPCS1	signal peptidase complex subunit 1 [Source:HGNC Symbol;Acc:HGNC:23401]	0.60
BCLTP5-035	6	ENSG00000170348	TMED10	transmembrane p24 trafficking protein 10 [Source:HGNC Symbol;Acc:HGNC:16998]	0.54
BCLTP5-036	2	ENSG00000164045	CDC25A	cell division cycle 25A [Source:HGNC Symbol;Acc:HGNC:1725]	1.72
BCLTP5-036	2	ENSG00000029993	HMGB3	high mobility group box 3 [Source:HGNC Symbol;Acc:HGNC:5004]	1.42
BCLTP5-037	3	ENSG00000087586	AURKA	aurora kinase A [Source:HGNC Symbol;Acc:HGNC:11393]	1.41
BCLTP5-037	3	ENSG00000115163	CENPA	centromere protein A [Source:HGNC Symbol;Acc:HGNC:1851]	1.74
BCLTP5-037	3	ENSG00000197780	TAF13	TATA-box binding protein associated factor 13 [Source:HGNC Symbol;Acc:HGNC:11546]	0.59
BCLTP5-038	4	ENSG00000134057	CCNB1	cyclin B1 [Source:HGNC Symbol;Acc:HGNC:1579]	1.20
BCLTP5-038	4	ENSG00000093009	CDC45	cell division cycle 45 [Source:HGNC Symbol;Acc:HGNC:1739]	2.08
BCLTP5-038	4	ENSG00000024526	DEPDC1	DEP domain containing 1 [Source:HGNC Symbol;Acc:HGNC:22949]	1.66
BCLTP5-038	4	ENSG00000186185	KIF18B	kinesin family member 18B [Source:HGNC Symbol;Acc:HGNC:27102]	1.68
BCLTP5-039	4	ENSG00000151725	CENPU	centromere protein U [Source:HGNC Symbol;Acc:HGNC:21348]	1.24
BCLTP5-039	4	ENSG00000101003	GINS1	GINS complex subunit 1 [Source:HGNC Symbol;Acc:HGNC:28980]	1.14
BCLTP5-039	4	ENSG00000037241	RPL26L1	ribosomal protein L26 like 1 [Source:HGNC Symbol;Acc:HGNC:17050]	0.72
BCLTP5-039	4	ENSG00000167553	TUBA1C	tubulin alpha 1c [Source:HGNC Symbol;Acc:HGNC:20768]	0.76
BCLTP5-040	3	ENSG00000035499	DEPDC1B	DEP domain containing 1B [Source:HGNC Symbol;Acc:HGNC:24902]	1.27
BCLTP5-040	3	ENSG00000111206	FOXM1	forkhead box M1 [Source:HGNC Symbol;Acc:HGNC:3818]	1.37
BCLTP5-040	3	ENSG00000134291	TMEM106C	transmembrane protein 106C [Source:HGNC Symbol;Acc:HGNC:28775]	0.68
BCLTP5-041	2	ENSG00000146670	CDCA5	cell division cycle associated 5 [Source:HGNC Symbol;Acc:HGNC:14626]	1.94

Cluster ID	Cluster Size	Gene ID	Gene Name	Gene Description	Log_2 FC TIV
BCLTP5-041	2	ENSG00000100526	CDKN3	cyclin dependent kinase inhibitor 3 [Source:HGNC Symbol;Acc:HGNC:1791]	1.44
BCLTP5-042	5	ENSG00000123485	HJURP	Holliday junction recognition protein [Source:HGNC Symbol;Acc:HGNC:25444]	1.88
BCLTP5-042	5	ENSG00000137807	KIF23	kinesin family member 23 [Source:HGNC Symbol;Acc:HGNC:6392]	1.49
BCLTP5-042	5	ENSG00000117650	NEK2	NIMA related kinase 2 [Source:HGNC Symbol;Acc:HGNC:7745]	1.37
BCLTP5-042	5	ENSG00000168078	PBK	PDZ binding kinase [Source:HGNC Symbol;Acc:HGNC:18282]	2.09
BCLTP5-042	5	ENSG00000176890	TYMS	thymidylate synthetase [Source:HGNC Symbol;Acc:HGNC:12441]	1.82
BCLTP5-043	3	ENSG00000011426	ANLN	anillin actin binding protein [Source:HGNC Symbol;Acc:HGNC:14082]	1.85
BCLTP5-043	3	ENSG00000163170	BOLA3	bolA family member 3 [Source:HGNC Symbol;Acc:HGNC:24415]	0.79
BCLTP5-043	3	ENSG00000251546	IGKV1D-39	immunoglobulin kappa variable 1D-39 [Source:HGNC Symbol;Acc:HGNC:5756]	0.64
BCLTP5-044	4	ENSG00000170312	CDK1	cyclin dependent kinase 1 [Source:HGNC Symbol;Acc:HGNC:1722]	1.43
BCLTP5-044	4	ENSG00000101412	E2F1	E2F transcription factor 1 [Source:HGNC Symbol;Acc:HGNC:3113]	1.19
BCLTP5-044	4	ENSG00000211962	IGHV1-46	immunoglobulin heavy variable 1-46 [Source:HGNC Symbol;Acc:HGNC:5554]	0.46
BCLTP5-044	4	ENSG00000140105	WARS	tryptophanyl-tRNA synthetase [Source:HGNC Symbol;Acc:HGNC:12729]	0.50
BCLTP5-045	5	ENSG00000153162	BMP6	bone morphogenetic protein 6 [Source:HGNC Symbol;Acc:HGNC:1073]	1.23
BCLTP5-045	5	ENSG00000211685	IGLC7	immunoglobulin lambda constant 7 [Source:HGNC Symbol;Acc:HGNC:5861]	0.73
BCLTP5-045	5	ENSG00000121152	NCAPH	non-SMC condensin I complex subunit H [Source:HGNC Symbol;Acc:HGNC:1112]	1.37
BCLTP5-045	5	ENSG00000189233	NUGGC	nuclear GTPase, germinal center associated [Source:HGNC Symbol;Acc:HGNC:33550]	0.75
BCLTP5-045	5	ENSG00000198794	SCAMP5	secretory carrier membrane protein 5 [Source:HGNC Symbol;Acc:HGNC:30386]	1.09
BCLTP5-046	10	ENSG00000198018	ENTPD7	ectonucleoside triphosphate diphosphohydrolase 7 [Source:HGNC Symbol;Acc:HGNC:19745]	0.72
BCLTP5-046	10	ENSG00000174371	EXO1	exonuclease 1 [Source:HGNC Symbol;Acc:HGNC:3511]	1.51
BCLTP5-046	10	ENSG00000211896	IGHG1	immunoglobulin heavy constant gamma 1 (G1m marker) [Source:HGNC Symbol;Acc:HGNC:5525]	1.61
BCLTP5-046	10	ENSG00000211892	IGHG4	immunoglobulin heavy constant gamma 4 (G4m marker) [Source:HGNC Symbol;Acc:HGNC:5528]	0.86
BCLTP5-046	10	ENSG00000253755	IGHGP	immunoglobulin heavy constant gamma P (non-functional) [Source:HGNC Symbol;Acc:HGNC:5529]	1.44
BCLTP5-046	10	ENSG00000224373	IGHV4-59	immunoglobulin heavy variable 4-59 [Source:HGNC Symbol;Acc:HGNC:5654]	0.62
BCLTP5-046	10	ENSG00000211653	IGLV1-40	immunoglobulin lambda variable 1-40 [Source:HGNC Symbol;Acc:HGNC:5877]	0.96
BCLTP5-046	10	ENSG00000137812	KNL1	kinetochore scaffold 1 [Source:HGNC Symbol;Acc:HGNC:24054]	0.71
BCLTP5-046	10	ENSG00000146918	NCAPG2	non-SMC condensin II complex subunit G2 [Source:HGNC Symbol;Acc:HGNC:21904]	0.71
BCLTP5-046	10	ENSG00000129235	TXNDC17	thioredoxin domain containing 17 [Source:HGNC Symbol;Acc:HGNC:28218]	0.69
BCLTP5-047	2	ENSG00000162676	GFI1	growth factor independent 1 transcriptional repressor [Source:HGNC Symbol;Acc:HGNC:4237]	1.32
BCLTP5-047	2	ENSG00000146733	PSPH	phosphoserine phosphatase [Source:HGNC Symbol;Acc:HGNC:9577]	0.61
BCLTP5-048	4	ENSG00000163808	KIF15	kinesin family member 15 [Source:HGNC Symbol;Acc:HGNC:17273]	1.10

Cluster ID	Cluster Size	Gene ID	Gene Name	Gene Description	Log_2 FC TIV
BCLTP5-048	4	ENSG00000105976	MET	MET proto-oncogene, receptor tyrosine kinase [Source:HGNC Symbol;Acc:HGNC:7029]	1.03
BCLTP5-048	4	ENSG00000080986	NDC80	NDC80, kinetochore complex component [Source:HGNC Symbol;Acc:HGNC:16909]	0.98
BCLTP5-048	4	ENSG00000078900	TP73	tumor protein p73 [Source:HGNC Symbol;Acc:HGNC:12003]	1.07
BCLTP5-049	4	ENSG00000106080	FKBP14	FK506 binding protein 14 [Source:HGNC Symbol;Acc:HGNC:18625]	0.58
BCLTP5-049	4	ENSG00000183087	GAS6	growth arrest specific 6 [Source:HGNC Symbol;Acc:HGNC:4168]	0.68
BCLTP5-049	4	ENSG00000274576	IGHV2-70	immunoglobulin heavy variable 2-70 [Source:HGNC Symbol;Acc:HGNC:5577]	0.31
BCLTP5-049	4	ENSG00000149516	MS4A3	membrane spanning 4-domains A3 [Source:HGNC Symbol;Acc:HGNC:7317]	1.35
BCLTP5-050	4	ENSG00000175984	DENND2C	DENN domain containing 2C [Source:HGNC Symbol;Acc:HGNC:24748]	0.95
BCLTP5-050	4	ENSG00000139734	DIAPH3	diaphanous related formin 3 [Source:HGNC Symbol;Acc:HGNC:15480]	2.32
BCLTP5-050	4	ENSG00000211645	IGLV1-50	immunoglobulin lambda variable 1-50 (non-functional) [Source:HGNC Symbol;Acc:HGNC:5881]	1.36
BCLTP5-050	4	ENSG00000111186	WNT5B	Wnt family member 5B [Source:HGNC Symbol;Acc:HGNC:16265]	1.86
BCLTP5-051	3	ENSG00000172339	ALG14	ALG14, UDP-N-acetylglucosaminyltransferase subunit [Source:HGNC Symbol;Acc:HGNC:28287]	0.41
BCLTP5-051	3	ENSG00000134489	HRH4	histamine receptor H4 [Source:HGNC Symbol;Acc:HGNC:17383]	0.97
BCLTP5-051	3	ENSG00000173578	XCR1	X-C motif chemokine receptor 1 [Source:HGNC Symbol;Acc:HGNC:1625]	0.60
BCLTP5-052	5	ENSG00000182197	EXT1	exostosin glycosyltransferase 1 [Source:HGNC Symbol;Acc:HGNC:3512]	0.58
BCLTP5-052	5	ENSG00000213430	HSPD1P1	heat shock protein family D (Hsp60) member 1 pseudogene 1 [Source:HGNC Symbol;Acc:HGNC:35133]	0.85
BCLTP5-052	5	ENSG00000163507	KIAA1524	KIAA1524 [Source:HGNC Symbol;Acc:HGNC:29302]	0.66
BCLTP5-052	5	ENSG00000154719	MRPL39	mitochondrial ribosomal protein L39 [Source:HGNC Symbol;Acc:HGNC:14027]	0.33
BCLTP5-052	5	ENSG00000025039	RRAGD	Ras related GTP binding D [Source:HGNC Symbol;Acc:HGNC:19903]	1.28
BCLTP5-053	4	ENSG00000224041	IGKV3D-15	immunoglobulin kappa variable 3D-15 (gene/pseudogene) [Source:HGNC Symbol;Acc:HGNC:5824]	0.49
BCLTP5-053	4	ENSG00000211669	IGLV3-10	immunoglobulin lambda variable 3-10 [Source:HGNC Symbol;Acc:HGNC:5897]	0.89
BCLTP5-053	4	ENSG00000211662	IGLV3-21	immunoglobulin lambda variable 3-21 [Source:HGNC Symbol;Acc:HGNC:5905]	0.84
BCLTP5-053	4	ENSG00000211659	IGLV3-25	immunoglobulin lambda variable 3-25 [Source:HGNC Symbol;Acc:HGNC:5908]	0.45
BCLTP5-054	4	ENSG00000241666			-0.63
BCLTP5-054	4	ENSG00000271533			-0.68
BCLTP5-054	4	ENSG00000168209	DDIT4	DNA damage inducible transcript 4 [Source:HGNC Symbol;Acc:HGNC:24944]	-0.79
BCLTP5-054	4	ENSG00000080546	SESN1	sestrin 1 [Source:HGNC Symbol;Acc:HGNC:21595]	-0.59

Table 31: Co-expressed gene clusters (B Cells, Day 5)

Cluster ID	Cluster Size	Gene ID	Gene Name	Gene Description	Log_2 FC TIV
BCLTP6-001	2	ENSG00000105976	MET	MET proto-oncogene, receptor tyrosine kinase [Source:HGNC Symbol;Acc:HGNC:7029]	1.32
BCLTP6-001	2	ENSG00000137804	NUSAP1	nucleolar and spindle associated protein 1 [Source:HGNC Symbol;Acc:HGNC:18538]	0.97
BCLTP6-002	2	ENSG00000106105	GARS	glycyl-tRNA synthetase [Source:HGNC Symbol;Acc:HGNC:4162]	0.61

Cluster ID	Cluster Size	Gene ID	Gene Name	Gene Description	Log_2 FC TIV
BCLTP6-002	2	ENSG00000044574	HSPA5	heat shock protein family A (Hsp70) member 5 [Source:HGNC Symbol;Acc:HGNC:5238]	1.25
BCLTP6-003	2	ENSG00000068912	ERLEC1	endoplasmic reticulum lectin 1 [Source:HGNC Symbol;Acc:HGNC:25222]	1.11
BCLTP6-003	2	ENSG00000118705	RPN2	ribophorin II [Source:HGNC Symbol;Acc:HGNC:10382]	1.06
BCLTP6-004	3	ENSG00000197157	SND1	staphylococcal nuclease and tudor domain containing 1 [Source:HGNC Symbol;Acc:HGNC:30646]	0.59
BCLTP6-004	3	ENSG00000122862	SRGN	serglycin [Source:HGNC Symbol;Acc:HGNC:9361]	0.88
BCLTP6-004	3	ENSG00000134825	TMEM258	transmembrane protein 258 [Source:HGNC Symbol;Acc:HGNC:1164]	0.82
BCLTP6-005	2	ENSG00000070214	SLC44A1	solute carrier family 44 member 1 [Source:HGNC Symbol;Acc:HGNC:18798]	1.17
BCLTP6-005	2	ENSG00000086598	TMED2	transmembrane p24 trafficking protein 2 [Source:HGNC Symbol;Acc:HGNC:16996]	0.69
BCLTP6-006	2	ENSG00000166598	HSP90B1	heat shock protein 90 beta family member 1 [Source:HGNC Symbol;Acc:HGNC:12028]	1.48
BCLTP6-006	2	ENSG00000124783	SSR1	signal sequence receptor subunit 1 [Source:HGNC Symbol;Acc:HGNC:11323]	0.75
BCLTP6-007	2	ENSG00000114850	SSR3	signal sequence receptor subunit 3 [Source:HGNC Symbol;Acc:HGNC:11325]	1.01
BCLTP6-007	2	ENSG00000048462	TNFRSF17	TNF receptor superfamily member 17 [Source:HGNC Symbol;Acc:HGNC:11913]	1.26
BCLTP6-008	2	ENSG00000111885	MAN1A1	mannosidase alpha class 1A member 1 [Source:HGNC Symbol;Acc:HGNC:6821]	1.26
BCLTP6-008	2	ENSG00000100219	XBP1	X-box binding protein 1 [Source:HGNC Symbol;Acc:HGNC:12801]	1.51
BCLTP6-009	3	ENSG00000004468	CD38	CD38 molecule [Source:HGNC Symbol;Acc:HGNC:1667]	1.26
BCLTP6-009	3	ENSG00000198856	OSTC	oligosaccharyltransferase complex non-catalytic subunit [Source:HGNC Symbol;Acc:HGNC:24448]	0.89
BCLTP6-009	3	ENSG00000134910	STT3A	STT3A, catalytic subunit of the oligosaccharyltransferase complex [Source:HGNC Symbol;Acc:HGNC:6172]	1.04
BCLTP6-010	2	ENSG00000067167	TRAM1	translocation associated membrane protein 1 [Source:HGNC Symbol;Acc:HGNC:20568]	0.69
BCLTP6-010	2	ENSG00000117143	UAP1	UDP-N-acetylglucosamine pyrophosphorylase 1 [Source:HGNC Symbol;Acc:HGNC:12457]	0.95
BCLTP6-011	2	ENSG00000178999	AURKB	aurora kinase B [Source:HGNC Symbol;Acc:HGNC:11390]	1.22
BCLTP6-011	2	ENSG00000171241	SHCBP1	SHC binding and spindle associated 1 [Source:HGNC Symbol;Acc:HGNC:29547]	1.54
BCLTP6-012	2	ENSG00000137563	GGH	gamma-glutamyl hydrolase [Source:HGNC Symbol;Acc:HGNC:4248]	1.55
BCLTP6-012	2	ENSG00000103226	NOMO3	NODAL modulator 3 [Source:HGNC Symbol;Acc:HGNC:25242]	0.78
BCLTP6-013	2	ENSG00000127022	CANX	calnexin [Source:HGNC Symbol;Acc:HGNC:1473]	0.69
BCLTP6-013	2	ENSG00000164032	H2AFZ	H2A histone family member Z [Source:HGNC Symbol;Acc:HGNC:4741]	0.58
BCLTP6-014	2	ENSG00000186810	CXCR3	C-X-C motif chemokine receptor 3 [Source:HGNC Symbol;Acc:HGNC:4540]	1.27
BCLTP6-014	2	ENSG00000121073	SLC35B1	solute carrier family 35 member B1 [Source:HGNC Symbol;Acc:HGNC:20798]	0.82
BCLTP6-015	2	ENSG00000135916	ITM2C	integral membrane protein 2C [Source:HGNC Symbol;Acc:HGNC:6175]	1.17
BCLTP6-015	2	ENSG00000106803	SEC61B	Sec61 translocon beta subunit [Source:HGNC Symbol;Acc:HGNC:16993]	0.94
BCLTP6-016	3	ENSG00000058262	SEC61A1	Sec61 translocon alpha 1 subunit [Source:HGNC Symbol;Acc:HGNC:18276]	0.82
BCLTP6-016	3	ENSG00000144867	SRPRB	SRP receptor beta subunit [Source:HGNC Symbol;Acc:HGNC:24085]	0.75
BCLTP6-016	3	ENSG00000153066	TXNDC11	thioredoxin domain containing 11 [Source:HGNC Symbol;Acc:HGNC:28030]	1.07
BCLTP6-017	2	ENSG00000117724	CENPF	centromere protein F [Source:HGNC Symbol;Acc:HGNC:1857]	0.92

Cluster ID	Cluster Size	Gene ID	Gene Name	Gene Description	Log_2 FC TIV
BCLTP6-017	2	ENSG00000173334	TRIB1	tribbles pseudokinase 1 [Source:HGNC Symbol;Acc:HGNC:16891]	1.23
BCLTP6-018	2	ENSG00000101310	SEC23B	Sec23 homolog B, coat complex II component [Source:HGNC Symbol;Acc:HGNC:10702]	0.66
BCLTP6-018	2	ENSG00000131871	SELENOS	selenoprotein S [Source:HGNC Symbol;Acc:HGNC:30396]	1.03
BCLTP6-019	2	ENSG00000138709	LARP1B	La ribonucleoprotein domain family member 1B [Source:HGNC Symbol;Acc:HGNC:24704]	0.72
BCLTP6-019	2	ENSG00000057657	PRDM1	PR/SET domain 1 [Source:HGNC Symbol;Acc:HGNC:9346]	0.95
BCLTP6-020	4	ENSG00000183508	FAM46C	family with sequence similarity 46 member C [Source:HGNC Symbol;Acc:HGNC:24712]	1.03
BCLTP6-020	4	ENSG00000173540	GMPPB	GDP-mannose pyrophosphorylase B [Source:HGNC Symbol;Acc:HGNC:22932]	0.84
BCLTP6-020	4	ENSG00000155304	HSPA13	heat shock protein family A (Hsp70) member 13 [Source:HGNC Symbol;Acc:HGNC:11375]	1.04
BCLTP6-020	4	ENSG00000166794	PPIB	peptidylprolyl isomerase B [Source:HGNC Symbol;Acc:HGNC:9255]	1.17
BCLTP6-021	5	ENSG00000149428	HYOU1	hypoxia up-regulated 1 [Source:HGNC Symbol;Acc:HGNC:16931]	1.02
BCLTP6-021	5	ENSG00000074695	LMAN1	lectin, mannose binding 1 [Source:HGNC Symbol;Acc:HGNC:6631]	1.12
BCLTP6-021	5	ENSG00000155660	PDIA4	protein disulfide isomerase family A member 4 [Source:HGNC Symbol;Acc:HGNC:30167]	1.38
BCLTP6-021	5	ENSG00000143870	PDIA6	protein disulfide isomerase family A member 6 [Source:HGNC Symbol;Acc:HGNC:30168]	1.01
BCLTP6-021	5	ENSG00000112473	SLC39A7	solute carrier family 39 member 7 [Source:HGNC Symbol;Acc:HGNC:4927]	0.76
BCLTP6-022	3	ENSG00000102158	MAGT1	magnesium transporter 1 [Source:HGNC Symbol;Acc:HGNC:28880]	0.72
BCLTP6-022	3	ENSG00000167004	PDIA3	protein disulfide isomerase family A member 3 [Source:HGNC Symbol;Acc:HGNC:4606]	0.61
BCLTP6-022	3	ENSG00000198900	TOP1	topoisomerase (DNA) I [Source:HGNC Symbol;Acc:HGNC:11986]	0.59
BCLTP6-023	2	ENSG00000105974	CAV1	caveolin 1 [Source:HGNC Symbol;Acc:HGNC:1527]	1.96
BCLTP6-023	2	ENSG00000142945	KIF2C	kinesin family member 2C [Source:HGNC Symbol;Acc:HGNC:6393]	1.06
BCLTP6-024	2	ENSG00000090520	DNAJB11	DnaJ heat shock protein family (Hsp40) member B11 [Source:HGNC Symbol;Acc:HGNC:14889]	0.93
BCLTP6-024	2	ENSG00000002549	LAP3	leucine aminopeptidase 3 [Source:HGNC Symbol;Acc:HGNC:18449]	0.72
BCLTP6-025	3	ENSG00000232613			0.72
BCLTP6-025	3	ENSG00000106415	GLCCI1	glucocorticoid induced 1 [Source:HGNC Symbol;Acc:HGNC:18713]	0.97
BCLTP6-025	3	ENSG00000110917	MLEC	malectin [Source:HGNC Symbol;Acc:HGNC:28973]	0.74
BCLTP6-026	2	ENSG00000115884	SDC1	syndecan 1 [Source:HGNC Symbol;Acc:HGNC:10658]	1.74
BCLTP6-026	2	ENSG00000198833	UBE2J1	ubiquitin conjugating enzyme E2 J1 [Source:HGNC Symbol;Acc:HGNC:17598]	0.72
BCLTP6-027	3	ENSG00000120697	ALG5	ALG5, dolichyl-phosphate beta-glucosyltransferase [Source:HGNC Symbol;Acc:HGNC:20266]	0.76
BCLTP6-027	3	ENSG00000118985	ELL2	elongation factor for RNA polymerase II 2 [Source:HGNC Symbol;Acc:HGNC:17064]	1.37
BCLTP6-027	3	ENSG00000113387	SUB1	SUB1 homolog, transcriptional regulator [Source:HGNC Symbol;Acc:HGNC:19985]	0.76
BCLTP6-028	2	ENSG00000072571	HMMR	hyaluronan mediated motility receptor [Source:HGNC Symbol;Acc:HGNC:5012]	1.38
BCLTP6-028	2	ENSG00000132646	PCNA	proliferating cell nuclear antigen [Source:HGNC Symbol;Acc:HGNC:8729]	0.57
BCLTP6-029	2	ENSG00000170312	CDK1	cyclin dependent kinase 1 [Source:HGNC Symbol;Acc:HGNC:1722]	1.48
BCLTP6-029	2	ENSG00000104738	MCM4	minichromosome maintenance complex component 4 [Source:HGNC Symbol;Acc:HGNC:6947]	0.76
BCLTP6-030	2	ENSG00000029993	HMGB3	high mobility group box 3 [Source:HGNC Symbol;Acc:HGNC:5004]	1.41

Cluster ID	Cluster Size	Gene ID	Gene Name	Gene Description	Log_2 FC TIV
BCLTP6-030	2	ENSG00000122952	ZWINT	ZW10 interacting kinetochore protein [Source:HGNC Symbol;Acc:HGNC:13195]	1.58
BCLTP6-031	2	ENSG00000185624	P4HB	prolyl 4-hydroxylase subunit beta [Source:HGNC Symbol;Acc:HGNC:8548]	0.95
BCLTP6-031	2	ENSG00000163053	SLC16A14	solute carrier family 16 member 14 [Source:HGNC Symbol;Acc:HGNC:26417]	1.71
BCLTP6-032	3	ENSG00000108578	BLMH	bleomycin hydrolase [Source:HGNC Symbol;Acc:HGNC:1059]	0.62
BCLTP6-032	3	ENSG00000259706	HSP90B2P	heat shock protein 90 beta family member 2, pseudogene [Source:HGNC Symbol;Acc:HGNC:12099]	1.49
BCLTP6-032	3	ENSG00000147649	MTDH	metadherin [Source:HGNC Symbol;Acc:HGNC:29608]	0.66
BCLTP6-033	2	ENSG00000143641	GALNT2	polypeptide N-acetylgalactosaminyltransferase 2 [Source:HGNC Symbol;Acc:HGNC:4124]	0.62
BCLTP6-033	2	ENSG00000112893	MAN2A1	mannosidase alpha class 2A member 1 [Source:HGNC Symbol;Acc:HGNC:6824]	0.64
BCLTP6-034	2	ENSG00000198876	DCAF12	DDB1 and CUL4 associated factor 12 [Source:HGNC Symbol;Acc:HGNC:19911]	1.08
BCLTP6-034	2	ENSG00000145703	IQGAP2	IQ motif containing GTPase activating protein 2 [Source:HGNC Symbol;Acc:HGNC:6111]	0.60
BCLTP6-035	2	ENSG00000198937	CCDC167	coiled-coil domain containing 167 [Source:HGNC Symbol;Acc:HGNC:21239]	0.86
BCLTP6-035	2	ENSG00000108826	MRPL27	mitochondrial ribosomal protein L27 [Source:HGNC Symbol;Acc:HGNC:14483]	0.59
BCLTP6-036	2	ENSG00000167476	JSRP1	junctional sarcoplasmic reticulum protein 1 [Source:HGNC Symbol;Acc:HGNC:24963]	0.89
BCLTP6-036	2	ENSG00000122188	LAX1	lymphocyte transmembrane adaptor 1 [Source:HGNC Symbol;Acc:HGNC:26005]	0.72
BCLTP6-037	2	ENSG00000123131	PRDX4	peroxiredoxin 4 [Source:HGNC Symbol;Acc:HGNC:17169]	1.25
BCLTP6-037	2	ENSG00000182934	SRPRA	SRP receptor alpha subunit [Source:HGNC Symbol;Acc:HGNC:11307]	0.63
BCLTP6-038	2	ENSG00000165672	PRDX3	peroxiredoxin 3 [Source:HGNC Symbol;Acc:HGNC:9354]	0.65
BCLTP6-038	2	ENSG00000132432	SEC61G	Sec61 translocon gamma subunit [Source:HGNC Symbol;Acc:HGNC:18277]	0.99
BCLTP6-039	3	ENSG00000171155	C1GALT1C1	C1GALT1 specific chaperone 1 [Source:HGNC Symbol;Acc:HGNC:24338]	1.00
BCLTP6-039	3	ENSG00000136770	DNAJC1	DnaJ heat shock protein family (Hsp40) member C1 [Source:HGNC Symbol;Acc:HGNC:20090]	0.96
BCLTP6-039	3	ENSG00000071537	SEL1L	SEL1L ERAD E3 ligase adaptor subunit [Source:HGNC Symbol;Acc:HGNC:10717]	0.91
BCLTP6-040	3	ENSG00000211896	IGHG1	immunoglobulin heavy constant gamma 1 (G1m marker) [Source:HGNC Symbol;Acc:HGNC:5525]	2.15
BCLTP6-040	3	ENSG00000068489	PRR11	proline rich 11 [Source:HGNC Symbol;Acc:HGNC:25619]	0.83
BCLTP6-040	3	ENSG00000167325	RRM1	ribonucleotide reductase catalytic subunit M1 [Source:HGNC Symbol;Acc:HGNC:10451]	0.65
BCLTP6-041	2	ENSG00000076003	MCM6	minichromosome maintenance complex component 6 [Source:HGNC Symbol;Acc:HGNC:6949]	0.68
BCLTP6-041	2	ENSG00000175063	UBE2C	ubiquitin conjugating enzyme E2 C [Source:HGNC Symbol;Acc:HGNC:15937]	1.24
BCLTP6-042	3	ENSG00000049656	CLPTM1L	CLPTM1 like [Source:HGNC Symbol;Acc:HGNC:24308]	0.69
BCLTP6-042	3	ENSG00000167861	HID1	HID1 domain containing [Source:HGNC Symbol;Acc:HGNC:15736]	1.13
BCLTP6-042	3	ENSG00000170476	MZB1	marginal zone B and B1 cell specific protein [Source:HGNC Symbol;Acc:HGNC:30125]	1.07
BCLTP6-043	9	ENSG00000128595	CALU	calumenin [Source:HGNC Symbol;Acc:HGNC:1458]	1.06
BCLTP6-043	9	ENSG00000100629	CEP128	centrosomal protein 128 [Source:HGNC Symbol;Acc:HGNC:20359]	1.37
BCLTP6-043	9	ENSG00000102580	DNAJC3	DnaJ heat shock protein family (Hsp40) member C3 [Source:HGNC Symbol;Acc:HGNC:9439]	0.91
BCLTP6-043	9	ENSG00000172469	MANEA	mannosidase endo-alpha [Source:HGNC Symbol;Acc:HGNC:21072]	1.03
BCLTP6-043	9	ENSG00000065485	PDIA5	protein disulfide isomerase family A member 5 [Source:HGNC Symbol;Acc:HGNC:24811]	1.43

Cluster ID	Cluster Size	Gene ID	Gene Name	Gene Description	Log_2 FC TIV
BCLTP6-043	9	ENSG00000166562	SEC11C	SEC11 homolog C, signal peptidase complex subunit [Source:HGNC Symbol;Acc:HGNC:23400]	1.16
BCLTP6-043	9	ENSG00000113615	SEC24A	SEC24 homolog A, COPII coat complex component [Source:HGNC Symbol;Acc:HGNC:10703]	1.15
BCLTP6-043	9	ENSG00000123352	SPATS2	spermatogenesis associated serine rich 2 [Source:HGNC Symbol;Acc:HGNC:18650]	0.96
BCLTP6-043	9	ENSG00000138768	USO1	USO1 vesicle transport factor [Source:HGNC Symbol;Acc:HGNC:30904]	0.64
BCLTP6-044	2	ENSG00000178445	GLDC	glycine decarboxylase [Source:HGNC Symbol;Acc:HGNC:4313]	1.49
BCLTP6-044	2	ENSG00000104635	SLC39A14	solute carrier family 39 member 14 [Source:HGNC Symbol;Acc:HGNC:20858]	0.63
BCLTP6-045	5	ENSG00000070081	NUCB2	nucleobindin 2 [Source:HGNC Symbol;Acc:HGNC:8044]	1.19
BCLTP6-045	5	ENSG00000102096	PIM2	Pim-2 proto-oncogene, serine/threonine kinase [Source:HGNC Symbol;Acc:HGNC:8987]	0.82
BCLTP6-045	5	ENSG00000150961	SEC24D	SEC24 homolog D, COPII coat complex component [Source:HGNC Symbol;Acc:HGNC:10706]	1.05
BCLTP6-045	5	ENSG00000026751	SLAMF7	SLAM family member 7 [Source:HGNC Symbol;Acc:HGNC:21394]	1.33
BCLTP6-045	5	ENSG00000065308	TRAM2	translocation associated membrane protein 2 [Source:HGNC Symbol;Acc:HGNC:16855]	1.09
BCLTP6-046	2	ENSG00000174132	FAM174A	family with sequence similarity 174 member A [Source:HGNC Symbol;Acc:HGNC:24943]	1.14
BCLTP6-046	2	ENSG00000211973	IGHV1-69	immunoglobulin heavy variable 1-69 [Source:HGNC Symbol;Acc:HGNC:5558]	1.77
BCLTP6-047	3	ENSG00000237649	KIFC1	kinesin family member C1 [Source:HGNC Symbol;Acc:HGNC:6389]	1.11
BCLTP6-047	3	ENSG00000074416	MGLL	monoglyceride lipase [Source:HGNC Symbol;Acc:HGNC:17038]	0.94
BCLTP6-047	3	ENSG00000085840	ORC1	origin recognition complex subunit 1 [Source:HGNC Symbol;Acc:HGNC:8487]	1.26
BCLTP6-048	2	ENSG00000085063	CD59	CD59 molecule [Source:HGNC Symbol;Acc:HGNC:1689]	0.67
BCLTP6-048	2	ENSG00000100228	RAB36	RAB36, member RAS oncogene family [Source:HGNC Symbol;Acc:HGNC:9775]	0.85
BCLTP6-049	3	ENSG00000134285	FKBP11	FK506 binding protein 11 [Source:HGNC Symbol;Acc:HGNC:18624]	1.11
BCLTP6-049	3	ENSG00000182054	IDH2	isocitrate dehydrogenase (NADP(+)) 2, mitochondrial [Source:HGNC Symbol;Acc:HGNC:5383]	0.87
BCLTP6-049	3	ENSG00000115902	SLC1A4	solute carrier family 1 member 4 [Source:HGNC Symbol;Acc:HGNC:10942]	0.89
BCLTP6-050	2	ENSG00000112984	KIF20A	kinesin family member 20A [Source:HGNC Symbol;Acc:HGNC:9787]	1.50
BCLTP6-050	2	ENSG00000088325	TPX2	TPX2, microtubule nucleation factor [Source:HGNC Symbol;Acc:HGNC:1249]	1.43
BCLTP6-051	3	ENSG00000198130	HIBCH	3-hydroxyisobutyryl-CoA hydrolase [Source:HGNC Symbol;Acc:HGNC:4908]	0.62
BCLTP6-051	3	ENSG00000069956	MAPK6	mitogen-activated protein kinase 6 [Source:HGNC Symbol;Acc:HGNC:6879]	0.64
BCLTP6-051	3	ENSG00000118515	SGK1	serum/glucocorticoid regulated kinase 1 [Source:HGNC Symbol;Acc:HGNC:10810]	1.15
BCLTP6-052	2	ENSG00000213047	DENND1B	DENN domain containing 1B [Source:HGNC Symbol;Acc:HGNC:28404]	0.71
BCLTP6-052	2	ENSG00000239264	TXNDC5	thioredoxin domain containing 5 [Source:HGNC Symbol;Acc:HGNC:21073]	1.07
BCLTP6-053	3	ENSG00000100342	APOL1	apolipoprotein L1 [Source:HGNC Symbol;Acc:HGNC:618]	0.94
BCLTP6-053	3	ENSG00000129173	E2F8	E2F transcription factor 8 [Source:HGNC Symbol;Acc:HGNC:24727]	1.96
BCLTP6-053	3	ENSG00000180879	SSR4	signal sequence receptor subunit 4 [Source:HGNC Symbol;Acc:HGNC:11326]	0.68
BCLTP6-054	2	ENSG00000102471	NDFIP2	Nedd4 family interacting protein 2 [Source:HGNC Symbol;Acc:HGNC:18537]	1.29

Cluster ID	Cluster Size	Gene ID	Gene Name	Gene Description	Log_2 FC TIV
BCLTP6-054	2	ENSG00000189233	NUGGC	nuclear GTPase, germinal center associated [Source:HGNC Symbol;Acc:HGNC:33550]	1.00
BCLTP6-055	4	ENSG00000119912	IDE	insulin degrading enzyme [Source:HGNC Symbol;Acc:HGNC:5381]	0.71
BCLTP6-055	4	ENSG00000113811	SELENOK	selenoprotein K [Source:HGNC Symbol;Acc:HGNC:30394]	0.71
BCLTP6-055	4	ENSG00000114902	SPCS1	signal peptidase complex subunit 1 [Source:HGNC Symbol;Acc:HGNC:23401]	0.73
BCLTP6-055	4	ENSG00000170348	TMED10	transmembrane p24 trafficking protein 10 [Source:HGNC Symbol;Acc:HGNC:16998]	0.66
BCLTP6-056	3	ENSG00000164305	CASP3	caspase 3 [Source:HGNC Symbol;Acc:HGNC:1504]	0.71
BCLTP6-056	3	ENSG00000075420	FNDC3B	fibronectin type III domain containing 3B [Source:HGNC Symbol;Acc:HGNC:24670]	1.54
BCLTP6-056	3	ENSG00000168268	NT5DC2	5'-nucleotidase domain containing 2 [Source:HGNC Symbol;Acc:HGNC:25717]	1.13
BCLTP6-057	3	ENSG00000108829	LRRC59	leucine rich repeat containing 59 [Source:HGNC Symbol;Acc:HGNC:28817]	0.98
BCLTP6-057	3	ENSG00000111424	VDR	vitamin D (1,25- dihydroxyvitamin D3) receptor [Source:HGNC Symbol;Acc:HGNC:12679]	1.20
BCLTP6-057	3	ENSG00000108953	YWHAE	tyrosine 3-monooxygenase/tryptophan 5-monooxygenase activation protein epsilon [Source:HGNC Symbol;Acc:HGNC:12851]	0.64
BCLTP6-058	2	ENSG00000184661	CDCA2	cell division cycle associated 2 [Source:HGNC Symbol;Acc:HGNC:14623]	1.68
BCLTP6-058	2	ENSG00000131747	TOP2A	topoisomerase (DNA) II alpha [Source:HGNC Symbol;Acc:HGNC:11989]	1.18
BCLTP6-059	4	ENSG00000179218	CALR	calreticulin [Source:HGNC Symbol;Acc:HGNC:1455]	0.78
BCLTP6-059	4	ENSG00000074842	MYDGF	myeloid derived growth factor [Source:HGNC Symbol;Acc:HGNC:16948]	1.43
BCLTP6-059	4	ENSG00000204386	NEU1	neuraminidase 1 [Source:HGNC Symbol;Acc:HGNC:7758]	0.78
BCLTP6-059	4	ENSG00000163902	RPN1	ribophorin I [Source:HGNC Symbol;Acc:HGNC:10381]	0.91
BCLTP6-060	2	ENSG00000185745	IFIT1	interferon induced protein with tetratricopeptide repeats 1 [Source:HGNC Symbol;Acc:HGNC:5407]	0.65
BCLTP6-060	2	ENSG00000170027	YWHAG	tyrosine 3-monooxygenase/tryptophan 5-monooxygenase activation protein gamma [Source:HGNC Symbol;Acc:HGNC:12852]	0.62
BCLTP6-061	5	ENSG00000088298	EDEM2	ER degradation enhancing alpha-mannosidase like protein 2 [Source:HGNC Symbol;Acc:HGNC:15877]	0.59
BCLTP6-061	5	ENSG00000101294	HM13	histocompatibility minor 13 [Source:HGNC Symbol;Acc:HGNC:16435]	0.89
BCLTP6-061	5	ENSG00000105438	KDELR1	KDEL endoplasmic reticulum protein retention receptor 1 [Source:HGNC Symbol;Acc:HGNC:6304]	0.64
BCLTP6-061	5	ENSG00000169223	LMAN2	lectin, mannose binding 2 [Source:HGNC Symbol;Acc:HGNC:16986]	0.75
BCLTP6-061	5	ENSG00000184840	TMED9	transmembrane p24 trafficking protein 9 [Source:HGNC Symbol;Acc:HGNC:24878]	0.76
BCLTP6-062	2	ENSG00000108641	B9D1	B9 domain containing 1 [Source:HGNC Symbol;Acc:HGNC:24123]	1.32
BCLTP6-062	2	ENSG00000171444	MCC	mutated in colorectal cancers [Source:HGNC Symbol;Acc:HGNC:6935]	1.20
BCLTP6-063	3	ENSG00000145354	CISD2	CDGSH iron sulfur domain 2 [Source:HGNC Symbol;Acc:HGNC:24212]	0.63
BCLTP6-063	3	ENSG00000049860	HEXB	hexosaminidase subunit beta [Source:HGNC Symbol;Acc:HGNC:4879]	0.60
BCLTP6-063	3	ENSG00000132465	JCHAIN	joining chain of multimeric IgA and IgM [Source:HGNC Symbol;Acc:HGNC:5713]	1.22
BCLTP6-064	3	ENSG00000075218	GTSE1	G2 and S-phase expressed 1 [Source:HGNC Symbol;Acc:HGNC:13698]	1.23
BCLTP6-064	3	ENSG00000112378	PERP	PERP, TP53 apoptosis effector [Source:HGNC Symbol;Acc:HGNC:17637]	1.15
BCLTP6-064	3	ENSG00000071539	TRIP13	thyroid hormone receptor interactor 13 [Source:HGNC Symbol;Acc:HGNC:12307]	1.23
BCLTP6-065	4	ENSG00000087502	ERGIC2	ERGIC and golgi 2 [Source:HGNC Symbol;Acc:HGNC:30208]	0.65

Cluster ID	Cluster Size	Gene ID	Gene Name	Gene Description	Log_2 FC TIV
BCLTP6-065	4	ENSG00000198380	GFPT1	glutamine–fructose-6-phosphate transaminase 1 [Source:HGNC Symbol;Acc:HGNC:4241]	0.69
BCLTP6-065	4	ENSG00000129128	SPCS3	signal peptidase complex subunit 3 [Source:HGNC Symbol;Acc:HGNC:26212]	0.69
BCLTP6-065	4	ENSG00000163527	STT3B	STT3B, catalytic subunit of the oligosaccharyltransferase complex [Source:HGNC Symbol;Acc:HGNC:30611]	0.71
BCLTP6-066	3	ENSG00000280411	IGHV1-69-2	immunoglobulin heavy variable 1-69-2 [Source:HGNC Symbol;Acc:HGNC:5562]	1.53
BCLTP6-066	3	ENSG00000211685	IGLC7	immunoglobulin lambda constant 7 [Source:HGNC Symbol;Acc:HGNC:5861]	0.80
BCLTP6-066	3	ENSG00000186818	LILRB4	leukocyte immunoglobulin like receptor B4 [Source:HGNC Symbol;Acc:HGNC:6608]	1.08
BCLTP6-067	2	ENSG00000112312	GMNN	geminin, DNA replication inhibitor [Source:HGNC Symbol;Acc:HGNC:17493]	0.86
BCLTP6-067	2	ENSG00000254395	IGHV4-55	immunoglobulin heavy variable 4-55 (pseudogene) [Source:HGNC Symbol;Acc:HGNC:5653]	0.95
BCLTP6-068	4	ENSG00000106462	EZH2	enhancer of zeste 2 polycomb repressive complex 2 subunit [Source:HGNC Symbol;Acc:HGNC:3527]	0.61
BCLTP6-068	4	ENSG00000253691	IGKV2OR22-4	immunoglobulin kappa variable 2/OR22-4 (pseudogene) [Source:HGNC Symbol;Acc:HGNC:5813]	1.50
BCLTP6-068	4	ENSG00000169962	TAS1R3	taste 1 receptor member 3 [Source:HGNC Symbol;Acc:HGNC:15661]	0.39
BCLTP6-068	4	ENSG00000136810	TXN	thioredoxin [Source:HGNC Symbol;Acc:HGNC:12435]	0.80
BCLTP6-069	2	ENSG00000040933	INPP4A	inositol polyphosphate-4-phosphatase type I A [Source:HGNC Symbol;Acc:HGNC:6074]	0.67
BCLTP6-069	2	ENSG00000095380	NANS	N-acetylneuraminate synthase [Source:HGNC Symbol;Acc:HGNC:19237]	0.70
BCLTP6-070	2	ENSG00000065328	MCM10	minichromosome maintenance 10 replication initiation factor [Source:HGNC Symbol;Acc:HGNC:18043]	1.43
BCLTP6-070	2	ENSG00000148773	MKI67	marker of proliferation Ki-67 [Source:HGNC Symbol;Acc:HGNC:7107]	1.33
BCLTP6-071	3	ENSG00000169679	BUB1	BUB1 mitotic checkpoint serine/threonine kinase [Source:HGNC Symbol;Acc:HGNC:1148]	1.61
BCLTP6-071	3	ENSG00000145386	CCNA2	cyclin A2 [Source:HGNC Symbol;Acc:HGNC:1578]	1.05
BCLTP6-071	3	ENSG00000117632	STMN1	stathmin 1 [Source:HGNC Symbol;Acc:HGNC:6510]	0.60
BCLTP6-072	4	ENSG00000093009	CDC45	cell division cycle 45 [Source:HGNC Symbol;Acc:HGNC:1739]	1.67
BCLTP6-072	4	ENSG00000094804	CDC6	cell division cycle 6 [Source:HGNC Symbol;Acc:HGNC:1744]	1.52
BCLTP6-072	4	ENSG00000244116	IGKV2-28	immunoglobulin kappa variable 2-28 [Source:HGNC Symbol;Acc:HGNC:5783]	0.92
BCLTP6-072	4	ENSG00000164109	MAD2L1	MAD2 mitotic arrest deficient-like 1 (yeast) [Source:HGNC Symbol;Acc:HGNC:6763]	0.90
BCLTP6-073	4	ENSG00000089685	BIRC5	baculoviral IAP repeat containing 5 [Source:HGNC Symbol;Acc:HGNC:593]	1.55
BCLTP6-073	4	ENSG00000024526	DEPDC1	DEP domain containing 1 [Source:HGNC Symbol;Acc:HGNC:22949]	1.82
BCLTP6-073	4	ENSG00000109805	NCAPG	non-SMC condensin I complex subunit G [Source:HGNC Symbol;Acc:HGNC:24304]	1.60
BCLTP6-073	4	ENSG00000080986	NDC80	NDC80, kinetochore complex component [Source:HGNC Symbol;Acc:HGNC:16909]	0.95
BCLTP6-074	3	ENSG00000092853	CLSPN	claspin [Source:HGNC Symbol;Acc:HGNC:19715]	1.11
BCLTP6-074	3	ENSG00000242534	IGKV2D-28	immunoglobulin kappa variable 2D-28 [Source:HGNC Symbol;Acc:HGNC:5799]	0.67
BCLTP6-074	3	ENSG00000108106	UBE2S	ubiquitin conjugating enzyme E2 S [Source:HGNC Symbol;Acc:HGNC:17895]	0.53
BCLTP6-075	2	ENSG00000224373	IGHV4-59	immunoglobulin heavy variable 4-59 [Source:HGNC Symbol;Acc:HGNC:5654]	0.98
BCLTP6-075	2	ENSG00000242766	IGKV1D-17	immunoglobulin kappa variable 1D-17 [Source:HGNC Symbol;Acc:HGNC:5749]	1.26
BCLTP6-076	2	ENSG00000241351	IGKV3-11	immunoglobulin kappa variable 3-11 [Source:HGNC Symbol;Acc:HGNC:5815]	0.86
BCLTP6-076	2	ENSG00000166851	PLK1	polo like kinase 1 [Source:HGNC Symbol;Acc:HGNC:9077]	0.70

Cluster ID	Cluster Size	Gene ID	Gene Name	Gene Description	Log_2 FC TIV
BCLTP6-077	3	ENSG00000011426	ANLN	anillin actin binding protein [Source:HGNC Symbol;Acc:HGNC:14082]	1.36
BCLTP6-077	3	ENSG00000163808	KIF15	kinesin family member 15 [Source:HGNC Symbol;Acc:HGNC:17273]	0.89
BCLTP6-077	3	ENSG00000121152	NCAPH	non-SMC condensin I complex subunit H [Source:HGNC Symbol;Acc:HGNC:1112]	1.23
BCLTP6-078	2	ENSG00000185477	GPRIN3	GPRIN family member 3 [Source:HGNC Symbol;Acc:HGNC:27733]	0.76
BCLTP6-078	2	ENSG00000227203	SUB1P1	SUB1 homolog, transcriptional regulator pseudogene 1 [Source:HGNC Symbol;Acc:HGNC:32300]	0.76
BCLTP6-079	2	ENSG00000224041	IGKV3D-15	immunoglobulin kappa variable 3D-15 (gene/pseudogene) [Source:HGNC Symbol;Acc:HGNC:5824]	0.80
BCLTP6-079	2	ENSG00000154839	SKA1	spindle and kinetochore associated complex subunit 1 [Source:HGNC Symbol;Acc:HGNC:28109]	0.99
BCLTP6-080	5	ENSG00000198826	ARHGAP11A	Rho GTPase activating protein 11A [Source:HGNC Symbol;Acc:HGNC:15783]	0.91
BCLTP6-080	5	ENSG00000151725	CENPU	centromere protein U [Source:HGNC Symbol;Acc:HGNC:21348]	1.04
BCLTP6-080	5	ENSG00000128590	DNAJB9	DnaJ heat shock protein family (Hsp40) member B9 [Source:HGNC Symbol;Acc:HGNC:6968]	0.90
BCLTP6-080	5	ENSG00000182481	KPNA2	karyopherin subunit alpha 2 [Source:HGNC Symbol;Acc:HGNC:6395]	0.77
BCLTP6-080	5	ENSG00000197780	TAF13	TATA-box binding protein associated factor 13 [Source:HGNC Symbol;Acc:HGNC:11546]	0.63
BCLTP6-081	2	ENSG00000143942	CHAC2	ChaC cation transport regulator homolog 2 [Source:HGNC Symbol;Acc:HGNC:32363]	1.08
BCLTP6-081	2	ENSG00000119326	CTNNAL1	catenin alpha like 1 [Source:HGNC Symbol;Acc:HGNC:2512]	1.38
BCLTP6-082	5	ENSG00000117411	B4GALT2	beta-1,4-galactosyltransferase 2 [Source:HGNC Symbol;Acc:HGNC:925]	0.86
BCLTP6-082	5	ENSG00000180535	BHLHA15	basic helix-loop-helix family member a15 [Source:HGNC Symbol;Acc:HGNC:22265]	1.04
BCLTP6-082	5	ENSG00000211892	IGHG4	immunoglobulin heavy constant gamma 4 (G4m marker) [Source:HGNC Symbol;Acc:HGNC:5528]	1.09
BCLTP6-082	5	ENSG00000143603	KCNN3	potassium calcium-activated channel subfamily N member 3 [Source:HGNC Symbol;Acc:HGNC:6292]	1.03
BCLTP6-082	5	ENSG00000138760	SCARB2	scavenger receptor class B member 2 [Source:HGNC Symbol;Acc:HGNC:1665]	0.65
BCLTP6-083	6	ENSG00000124788	ATXN1	ataxin 1 [Source:HGNC Symbol;Acc:HGNC:10548]	0.99
BCLTP6-083	6	ENSG00000136026	CKAP4	cytoskeleton associated protein 4 [Source:HGNC Symbol;Acc:HGNC:16991]	1.06
BCLTP6-083	6	ENSG00000154511	FAM69A	family with sequence similarity 69 member A [Source:HGNC Symbol;Acc:HGNC:32213]	0.66
BCLTP6-083	6	ENSG00000115677	HDLBP	high density lipoprotein binding protein [Source:HGNC Symbol;Acc:HGNC:4857]	0.90
BCLTP6-083	6	ENSG00000228589	SPCS2P4	signal peptidase complex subunit 2 homolog (S. cerevisiae) pseudogene 4 [Source:HGNC Symbol;Acc:HGNC:45237]	0.84
BCLTP6-083	6	ENSG00000109501	WFS1	wolframin ER transmembrane glycoprotein [Source:HGNC Symbol;Acc:HGNC:12762]	0.81
BCLTP6-084	2	ENSG00000211677	IGLC2	immunoglobulin lambda constant 2 [Source:HGNC Symbol;Acc:HGNC:5856]	1.21
BCLTP6-084	2	ENSG00000092621	PHGDH	phosphoglycerate dehydrogenase [Source:HGNC Symbol;Acc:HGNC:8923]	0.98
BCLTP6-085	2	ENSG00000066279	ASPM	abnormal spindle microtubule assembly [Source:HGNC Symbol;Acc:HGNC:19048]	1.15
BCLTP6-085	2	ENSG00000211952	IGHV4-28	immunoglobulin heavy variable 4-28 [Source:HGNC Symbol;Acc:HGNC:5645]	0.46
BCLTP6-086	4	ENSG00000136010	ALDH1L2	aldehyde dehydrogenase 1 family member L2 [Source:HGNC Symbol;Acc:HGNC:26777]	1.58
BCLTP6-086	4	ENSG00000198855	FICD	FIC domain containing [Source:HGNC Symbol;Acc:HGNC:18416]	0.95
BCLTP6-086	4	ENSG00000100804	PSMB5	proteasome subunit beta 5 [Source:HGNC Symbol;Acc:HGNC:9542]	0.61

Cluster ID	Cluster Size	Gene ID	Gene Name	Gene Description	Log_2 FC TIV
BCLTP6-086	4	ENSG00000113621	TXNDC15	thioredoxin domain containing 15 [Source:HGNC Symbol;Acc:HGNC:20652]	0.68
BCLTP6-087	4	ENSG00000143476	DTL	denticleless E3 ubiquitin protein ligase homolog [Source:HGNC Symbol;Acc:HGNC:30288]	1.51
BCLTP6-087	4	ENSG00000123485	HJURP	Holliday junction recognition protein [Source:HGNC Symbol;Acc:HGNC:25444]	1.60
BCLTP6-087	4	ENSG00000211645	IGLV1-50	immunoglobulin lambda variable 1-50 (non-functional) [Source:HGNC Symbol;Acc:HGNC:5881]	1.62
BCLTP6-087	4	ENSG00000176890	TYMS	thymidylate synthetase [Source:HGNC Symbol;Acc:HGNC:12441]	1.47
BCLTP6-088	5	ENSG00000123975	CKS2	CDC28 protein kinase regulatory subunit 2 [Source:HGNC Symbol;Acc:HGNC:2000]	0.78
BCLTP6-088	5	ENSG00000129562	DAD1	defender against cell death 1 [Source:HGNC Symbol;Acc:HGNC:2664]	0.64
BCLTP6-088	5	ENSG00000227295	ELL2P1	elongation factor for RNA polymerase II 2 pseudogene 1 [Source:HGNC Symbol;Acc:HGNC:39343]	1.22
BCLTP6-088	5	ENSG00000203914	HSP90B3P	heat shock protein 90 beta family member 3, pseudogene [Source:HGNC Symbol;Acc:HGNC:12100]	1.50
BCLTP6-088	5	ENSG00000186594	MIR22HG	MIR22 host gene [Source:HGNC Symbol;Acc:HGNC:28219]	1.19
BCLTP6-089	2	ENSG00000131153	GINS2	GINS complex subunit 2 [Source:HGNC Symbol;Acc:HGNC:24575]	0.94
BCLTP6-089	2	ENSG00000154277	UCHL1	ubiquitin C-terminal hydrolase L1 [Source:HGNC Symbol;Acc:HGNC:12513]	1.88
BCLTP6-090	3	ENSG00000182197	EXT1	exostosin glycosyltransferase 1 [Source:HGNC Symbol;Acc:HGNC:3512]	0.64
BCLTP6-090	3	ENSG00000129636	ITFG1	integrin alpha FG-GAP repeat containing 1 [Source:HGNC Symbol;Acc:HGNC:30697]	0.67
BCLTP6-090	3	ENSG00000079931	MOXD1	monooxygenase DBH like 1 [Source:HGNC Symbol;Acc:HGNC:21063]	1.20
BCLTP6-091	3	ENSG00000172339	ALG14	ALG14, UDP-N-acetylglucosaminyltransferase subunit [Source:HGNC Symbol;Acc:HGNC:28287]	0.61
BCLTP6-091	3	ENSG00000185164	NOMO2	NODAL modulator 2 [Source:HGNC Symbol;Acc:HGNC:22652]	1.20
BCLTP6-091	3	ENSG00000143333	RGS16	regulator of G-protein signaling 16 [Source:HGNC Symbol;Acc:HGNC:9997]	1.22
BCLTP6-092	3	ENSG00000211938	IGHV3-7	immunoglobulin heavy variable 3-7 [Source:HGNC Symbol;Acc:HGNC:5620]	2.47
BCLTP6-092	3	ENSG00000107104	KANK1	KN motif and ankyrin repeat domains 1 [Source:HGNC Symbol;Acc:HGNC:19309]	1.31
BCLTP6-092	3	ENSG00000179222	MAGED1	MAGE family member D1 [Source:HGNC Symbol;Acc:HGNC:6813]	0.60
BCLTP6-093	4	ENSG00000126787	DLGAP5	DLG associated protein 5 [Source:HGNC Symbol;Acc:HGNC:16864]	1.57
BCLTP6-093	4	ENSG00000138160	KIF11	kinesin family member 11 [Source:HGNC Symbol;Acc:HGNC:6388]	0.93
BCLTP6-093	4	ENSG00000168078	PBK	PDZ binding kinase [Source:HGNC Symbol;Acc:HGNC:18282]	1.99
BCLTP6-093	4	ENSG00000171848	RRM2	ribonucleotide reductase regulatory subunit M2 [Source:HGNC Symbol;Acc:HGNC:10452]	1.68
BCLTP6-094	3	ENSG00000100526	CDKN3	cyclin dependent kinase inhibitor 3 [Source:HGNC Symbol;Acc:HGNC:1791]	1.38
BCLTP6-094	3	ENSG00000137807	KIF23	kinesin family member 23 [Source:HGNC Symbol;Acc:HGNC:6392]	1.55
BCLTP6-094	3	ENSG00000142731	PLK4	polo like kinase 4 [Source:HGNC Symbol;Acc:HGNC:11397]	0.69
BCLTP6-095	3	ENSG00000235587	GAPDHP65	glyceraldehyde 3 phosphate dehydrogenase pseudogene 65 [Source:HGNC Symbol;Acc:HGNC:4143]	0.65
BCLTP6-095	3	ENSG00000187837	HIST1H1C	histone cluster 1 H1 family member c [Source:HGNC Symbol;Acc:HGNC:4716]	0.63
BCLTP6-095	3	ENSG00000211649	IGLV7-46	immunoglobulin lambda variable 7-46 (gene/pseudogene) [Source:HGNC Symbol;Acc:HGNC:5930]	0.68
BCLTP6-096	4	ENSG00000110063	DCPS	decapping enzyme, scavenger [Source:HGNC Symbol;Acc:HGNC:29812]	0.71

Cluster ID	Cluster Size	Gene ID	Gene Name	Gene Description	Log_2 FC TIV
BCLTP6-096	4	ENSG00000211679	IGLC3	immunoglobulin lambda constant 3 (Kern-Oz+ marker) [Source:HGNC Symbol;Acc:HGNC:5857]	0.84
BCLTP6-096	4	ENSG00000167634	NLRP7	NLR family pyrin domain containing 7 [Source:HGNC Symbol;Acc:HGNC:22947]	1.23
BCLTP6-096	4	ENSG00000136161	RCBTB2	RCC1 and BTB domain containing protein 2 [Source:HGNC Symbol;Acc:HGNC:1914]	0.83
BCLTP6-097	2	ENSG00000104290	FZD3	frizzled class receptor 3 [Source:HGNC Symbol;Acc:HGNC:4041]	0.64
BCLTP6-097	2	ENSG00000133328	HRASLS2	HRAS like suppressor 2 [Source:HGNC Symbol;Acc:HGNC:17824]	0.99
BCLTP6-098	5	ENSG00000139193	CD27	CD27 molecule [Source:HGNC Symbol;Acc:HGNC:11922]	0.89
BCLTP6-098	5	ENSG00000138073	PREB	prolactin regulatory element binding [Source:HGNC Symbol;Acc:HGNC:9356]	0.63
BCLTP6-098	5	ENSG00000011478	QPCTL	glutaminyl-peptide cyclotransferase like [Source:HGNC Symbol;Acc:HGNC:25952]	0.79
BCLTP6-098	5	ENSG00000157020	SEC13	SEC13 homolog, nuclear pore and COPII coat complex component [Source:HGNC Symbol;Acc:HGNC:10697]	0.61
BCLTP6-098	5	ENSG00000120725	SIL1	SIL1 nucleotide exchange factor [Source:HGNC Symbol;Acc:HGNC:24624]	0.82
BCLTP6-099	7	ENSG00000123989	CHPF	chondroitin polymerizing factor [Source:HGNC Symbol;Acc:HGNC:24291]	1.44
BCLTP6-099	7	ENSG00000253755	IGHGP	immunoglobulin heavy constant gamma P (non-functional) [Source:HGNC Symbol;Acc:HGNC:5529]	1.85
BCLTP6-099	7	ENSG00000108984	MAP2K6	mitogen-activated protein kinase kinase 6 [Source:HGNC Symbol;Acc:HGNC:6846]	0.61
BCLTP6-099	7	ENSG00000128228	SDF2L1	stromal cell derived factor 2 like 1 [Source:HGNC Symbol;Acc:HGNC:10676]	1.23
BCLTP6-099	7	ENSG00000136868	SLC31A1	solute carrier family 31 member 1 [Source:HGNC Symbol;Acc:HGNC:11016]	0.82
BCLTP6-099	7	ENSG00000017483	SLC38A5	solute carrier family 38 member 5 [Source:HGNC Symbol;Acc:HGNC:18070]	0.97
BCLTP6-099	7	ENSG00000116649	SRM	spermidine synthase [Source:HGNC Symbol;Acc:HGNC:11296]	0.63
BCLTP6-100	2	ENSG00000211964	IGHV3-48	immunoglobulin heavy variable 3-48 [Source:HGNC Symbol;Acc:HGNC:5606]	0.54
BCLTP6-100	2	ENSG00000099337	KCNK6	potassium two pore domain channel subfamily K member 6 [Source:HGNC Symbol;Acc:HGNC:6281]	0.81
BCLTP6-101	4	ENSG00000146376	ARHGAP18	Rho GTPase activating protein 18 [Source:HGNC Symbol;Acc:HGNC:21035]	0.61
BCLTP6-101	4	ENSG00000148468	FAM171A1	family with sequence similarity 171 member A1 [Source:HGNC Symbol;Acc:HGNC:23522]	2.42
BCLTP6-101	4	ENSG00000244575	IGKV1-27	immunoglobulin kappa variable 1-27 [Source:HGNC Symbol;Acc:HGNC:5735]	1.00
BCLTP6-101	4	ENSG00000025039	RRAGD	Ras related GTP binding D [Source:HGNC Symbol;Acc:HGNC:19903]	1.39
BCLTP6-102	2	ENSG00000113140	SPARC	secreted protein acidic and cysteine rich [Source:HGNC Symbol;Acc:HGNC:11219]	2.23
BCLTP6-102	2	ENSG00000129235	TXNDC17	thioredoxin domain containing 17 [Source:HGNC Symbol;Acc:HGNC:28218]	0.60
BCLTP6-103	3	ENSG00000165272	AQP3	aquaporin 3 (Gill blood group) [Source:HGNC Symbol;Acc:HGNC:636]	0.94
BCLTP6-103	3	ENSG00000029153	ARNTL2	aryl hydrocarbon receptor nuclear translocator like 2 [Source:HGNC Symbol;Acc:HGNC:18984]	0.78
BCLTP6-103	3	ENSG00000167513	CDT1	chromatin licensing and DNA replication factor 1 [Source:HGNC Symbol;Acc:HGNC:24576]	0.71
BCLTP6-104	6	ENSG00000258572			0.97
BCLTP6-104	6	ENSG00000111665	CDCA3	cell division cycle associated 3 [Source:HGNC Symbol;Acc:HGNC:14624]	1.13
BCLTP6-104	6	ENSG00000135476	ESPL1	extra spindle pole bodies like 1, separase [Source:HGNC Symbol;Acc:HGNC:16856]	0.86
BCLTP6-104	6	ENSG00000140451	PIF1	PIF1 5'-to-3' DNA helicase [Source:HGNC Symbol;Acc:HGNC:26220]	0.69

Cluster ID	Cluster Size	Gene ID	Gene Name	Gene Description	Log_2 FC TIV
BCLTP6-104	6	ENSG00000088826	SMOX	spermine oxidase [Source:HGNC Symbol;Acc:HGNC:15862]	1.05
BCLTP6-104	6	ENSG00000076382	SPAG5	sperm associated antigen 5 [Source:HGNC Symbol;Acc:HGNC:13452]	0.90
BCLTP6-105	3	ENSG00000184164	CRELD2	cysteine rich with EGF like domains 2 [Source:HGNC Symbol;Acc:HGNC:28150]	0.79
BCLTP6-105	3	ENSG00000183087	GAS6	growth arrest specific 6 [Source:HGNC Symbol;Acc:HGNC:4168]	0.91
BCLTP6-105	3	ENSG00000004866	ST7	suppression of tumorigenicity 7 [Source:HGNC Symbol;Acc:HGNC:11351]	0.85
BCLTP6-106	2	ENSG00000271178	IGHV3OR16-13	immunoglobulin heavy variable 3/OR16-13 (non-functional) [Source:HGNC Symbol;Acc:HGNC:5637]	1.36
BCLTP6-106	2	ENSG00000185155	MIXL1	Mix paired-like homeobox [Source:HGNC Symbol;Acc:HGNC:13363]	1.15
BCLTP6-107	2	ENSG00000282600			0.81
BCLTP6-107	2	ENSG00000211943	IGHV3-15	immunoglobulin heavy variable 3-15 [Source:HGNC Symbol;Acc:HGNC:5582]	0.92
BCLTP6-108	3	ENSG00000088340	FER1L4	fer-1 like family member 4, pseudogene [Source:HGNC Symbol;Acc:HGNC:15801]	2.01
BCLTP6-108	3	ENSG00000144724	PTPRG	protein tyrosine phosphatase, receptor type G [Source:HGNC Symbol;Acc:HGNC:9671]	0.84
BCLTP6-108	3	ENSG00000198722	UNC13B	unc-13 homolog B [Source:HGNC Symbol;Acc:HGNC:12566]	1.47
BCLTP6-109	3	ENSG00000142675	CNKSR1	connector enhancer of kinase suppressor of Ras 1 [Source:HGNC Symbol;Acc:HGNC:19700]	0.67
BCLTP6-109	3	ENSG00000211651	IGLV1-44	immunoglobulin lambda variable 1-44 [Source:HGNC Symbol;Acc:HGNC:5879]	1.93
BCLTP6-109	3	ENSG00000198792	TMEM184B	transmembrane protein 184B [Source:HGNC Symbol;Acc:HGNC:1310]	0.72
BCLTP6-110	6	ENSG00000123080	CDKN2C	cyclin dependent kinase inhibitor 2C [Source:HGNC Symbol;Acc:HGNC:1789]	0.88
BCLTP6-110	6	ENSG00000140525	FANCI	Fanconi anemia complementation group I [Source:HGNC Symbol;Acc:HGNC:25568]	0.73
BCLTP6-110	6	ENSG00000051341	POLQ	DNA polymerase theta [Source:HGNC Symbol;Acc:HGNC:9186]	1.00
BCLTP6-110	6	ENSG00000037241	RPL26L1	ribosomal protein L26 like 1 [Source:HGNC Symbol;Acc:HGNC:17050]	0.71
BCLTP6-110	6	ENSG00000167900	TK1	thymidine kinase 1 [Source:HGNC Symbol;Acc:HGNC:11830]	1.01
BCLTP6-110	6	ENSG00000145817	YIPF5	Yip1 domain family member 5 [Source:HGNC Symbol;Acc:HGNC:24877]	0.61
BCLTP6-111	3	ENSG00000182022	CHST15	carbohydrate sulfotransferase 15 [Source:HGNC Symbol;Acc:HGNC:18137]	0.59
BCLTP6-111	3	ENSG00000162676	GFI1	growth factor independent 1 transcriptional repressor [Source:HGNC Symbol;Acc:HGNC:4237]	1.54
BCLTP6-111	3	ENSG00000173218	VANGL1	VANGL planar cell polarity protein 1 [Source:HGNC Symbol;Acc:HGNC:15512]	0.65
BCLTP6-112	4	ENSG00000203760	CENPW	centromere protein W [Source:HGNC Symbol;Acc:HGNC:21488]	0.89
BCLTP6-112	4	ENSG00000172115	CYCS	cytochrome c, somatic [Source:HGNC Symbol;Acc:HGNC:19986]	0.61
BCLTP6-112	4	ENSG00000224607	IGKV1D-27	immunoglobulin kappa variable 1D-27 (pseudogene) [Source:HGNC Symbol;Acc:HGNC:5751]	1.22
BCLTP6-112	4	ENSG00000166803	KIAA0101	KIAA0101 [Source:HGNC Symbol;Acc:HGNC:28961]	1.03
BCLTP6-113	3	ENSG00000012048	BRCA1	BRCA1, DNA repair associated [Source:HGNC Symbol;Acc:HGNC:1100]	0.72
BCLTP6-113	3	ENSG00000090889	KIF4A	kinesin family member 4A [Source:HGNC Symbol;Acc:HGNC:13339]	1.17
BCLTP6-113	3	ENSG00000078900	TP73	tumor protein p73 [Source:HGNC Symbol;Acc:HGNC:12003]	1.05
BCLTP6-114	5	ENSG00000064763	FAR2	fatty acyl-CoA reductase 2 [Source:HGNC Symbol;Acc:HGNC:25531]	0.91
BCLTP6-114	5	ENSG00000101003	GINS1	GINS complex subunit 1 [Source:HGNC Symbol;Acc:HGNC:28980]	0.89
BCLTP6-114	5	ENSG00000118193	KIF14	kinesin family member 14 [Source:HGNC Symbol;Acc:HGNC:19181]	1.08

Cluster ID	Cluster Size	Gene ID	Gene Name	Gene Description	Log_2 FC TIV
BCLTP6-114	5	ENSG00000184232	OAF	out at first homolog [Source:HGNC Symbol;Acc:HGNC:28752]	1.01
BCLTP6-114	5	ENSG00000183010	PYCR1	pyrroline-5-carboxylate reductase 1 [Source:HGNC Symbol;Acc:HGNC:9721]	1.14
BCLTP6-115	2	ENSG00000228327			-0.63
BCLTP6-115	2	ENSG00000185522	LMNTD2	lamin tail domain containing 2 [Source:HGNC Symbol;Acc:HGNC:28561]	-0.61
BCLTP6-116	2	ENSG00000182985	CADM1	cell adhesion molecule 1 [Source:HGNC Symbol;Acc:HGNC:5951]	1.13
BCLTP6-116	2	ENSG00000115415	STAT1	signal transducer and activator of transcription 1 [Source:HGNC Symbol;Acc:HGNC:11362]	0.46
BCLTP6-117	5	ENSG00000216775			1.47
BCLTP6-117	5	ENSG00000198018	ENTPD7	ectonucleoside triphosphate diphosphohydrolase 7 [Source:HGNC Symbol;Acc:HGNC:19745]	0.83
BCLTP6-117	5	ENSG00000168496	FEN1	flap structure-specific endonuclease 1 [Source:HGNC Symbol;Acc:HGNC:3650]	0.84
BCLTP6-117	5	ENSG00000186185	KIF18B	kinesin family member 18B [Source:HGNC Symbol;Acc:HGNC:27102]	1.29
BCLTP6-117	5	ENSG00000065911	MTHFD2	methylenetetrahydrofolate dehydrogenase (NADP+ dependent) 2, methenyltetrahydrofolate cyclohydrolase [Source:HGNC Symbol;Acc:HGNC:7434]	0.83
BCLTP6-118	2	ENSG00000185480	PARPBP	PARP1 binding protein [Source:HGNC Symbol;Acc:HGNC:26074]	0.92
BCLTP6-118	2	ENSG00000111186	WNT5B	Wnt family member 5B [Source:HGNC Symbol;Acc:HGNC:16265]	2.38
BCLTP6-119	3	ENSG00000243238	IGKV2-30	immunoglobulin kappa variable 2-30 [Source:HGNC Symbol;Acc:HGNC:5785]	1.46
BCLTP6-119	3	ENSG00000239571	IGKV2D-30	immunoglobulin kappa variable 2D-30 [Source:HGNC Symbol;Acc:HGNC:5801]	1.49
BCLTP6-119	3	ENSG00000211668	IGLV2-11	immunoglobulin lambda variable 2-11 [Source:HGNC Symbol;Acc:HGNC:5887]	0.66
BCLTP6-120	7	ENSG00000138166	DUSP5	dual specificity phosphatase 5 [Source:HGNC Symbol;Acc:HGNC:3071]	1.66
BCLTP6-120	7	ENSG00000111640	GAPDH	glyceraldehyde-3-phosphate dehydrogenase [Source:HGNC Symbol;Acc:HGNC:4141]	0.73
BCLTP6-120	7	ENSG00000163754	GYG1	glycogenin 1 [Source:HGNC Symbol;Acc:HGNC:4699]	0.61
BCLTP6-120	7	ENSG00000101057	MYBL2	MYB proto-oncogene like 2 [Source:HGNC Symbol;Acc:HGNC:7548]	1.25
BCLTP6-120	7	ENSG00000168653	NDUFS5	NADH:ubiquinone oxidoreductase subunit S5 [Source:HGNC Symbol;Acc:HGNC:7712]	0.60
BCLTP6-120	7	ENSG00000146733	PSPH	phosphoserine phosphatase [Source:HGNC Symbol;Acc:HGNC:9577]	0.74
BCLTP6-120	7	ENSG00000168701	TMEM208	transmembrane protein 208 [Source:HGNC Symbol;Acc:HGNC:25015]	0.82
BCLTP6-121	3	ENSG00000255733	IFNG-AS1	IFNG antisense RNA 1 [Source:HGNC Symbol;Acc:HGNC:43910]	0.62
BCLTP6-121	3	ENSG00000231475	IGHV4-31	immunoglobulin heavy variable 4-31 [Source:HGNC Symbol;Acc:HGNC:5649]	0.61
BCLTP6-121	3	ENSG00000239975	IGKV1D-33	immunoglobulin kappa variable 1D-33 [Source:HGNC Symbol;Acc:HGNC:5753]	1.58
BCLTP6-122	3	ENSG00000270472	IGHV3OR16-9	immunoglobulin heavy variable 3/OR16-9 (non-functional) [Source:HGNC Symbol;Acc:HGNC:5644]	1.56
BCLTP6-122	3	ENSG00000211648	IGLV1-47	immunoglobulin lambda variable 1-47 [Source:HGNC Symbol;Acc:HGNC:5880]	1.29
BCLTP6-122	3	ENSG00000177301	KCNA2	potassium voltage-gated channel subfamily A member 2 [Source:HGNC Symbol;Acc:HGNC:6220]	1.05
BCLTP6-123	6	ENSG00000153093	ACOXL	acyl-CoA oxidase-like [Source:HGNC Symbol;Acc:HGNC:25621]	2.20
BCLTP6-123	6	ENSG00000111291	GPRC5D	G protein-coupled receptor class C group 5 member D [Source:HGNC Symbol;Acc:HGNC:13310]	1.02
BCLTP6-123	6	ENSG00000172965	MIR4435-2HG	MIR4435-2 host gene [Source:HGNC Symbol;Acc:HGNC:35163]	1.25

Cluster ID	Cluster Size	Gene ID	Gene Name	Gene Description	Log_2 FC TIV
BCLTP6-123	6	ENSG00000239672	NME1	NME/NM23 nucleoside diphosphate kinase 1 [Source:HGNC Symbol;Acc:HGNC:7849]	0.76
BCLTP6-123	6	ENSG00000164611	PTTG1	pituitary tumor-transforming 1 [Source:HGNC Symbol;Acc:HGNC:9690]	1.20
BCLTP6-123	6	ENSG00000100883	SRP54	signal recognition particle 54 [Source:HGNC Symbol;Acc:HGNC:11301]	0.73
BCLTP6-124	4	ENSG00000248571			1.63
BCLTP6-124	4	ENSG00000113273	ARSB	arylsulfatase B [Source:HGNC Symbol;Acc:HGNC:714]	0.61
BCLTP6-124	4	ENSG00000121807	CCR2	C-C motif chemokine receptor 2 [Source:HGNC Symbol;Acc:HGNC:1603]	1.66
BCLTP6-124	4	ENSG00000137806	NDUFAF1	NADH:ubiquinone oxidoreductase complex assembly factor 1 [Source:HGNC Symbol;Acc:HGNC:18828]	0.60
BCLTP6-125	3	ENSG00000035499	DEPDC1B	DEP domain containing 1B [Source:HGNC Symbol;Acc:HGNC:24902]	0.94
BCLTP6-125	3	ENSG00000164104	HMGB2	high mobility group box 2 [Source:HGNC Symbol;Acc:HGNC:5000]	0.50
BCLTP6-125	3	ENSG00000211976	IGHV3-73	immunoglobulin heavy variable 3-73 [Source:HGNC Symbol;Acc:HGNC:5623]	0.84
BCLTP6-126	2	ENSG00000241755	IGKV1-9	immunoglobulin kappa variable 1-9 [Source:HGNC Symbol;Acc:HGNC:5744]	0.93
BCLTP6-126	2	ENSG00000211632	IGKV3D-11	immunoglobulin kappa variable 3D-11 [Source:HGNC Symbol;Acc:HGNC:5823]	0.74
BCLTP6-127	2	ENSG00000280063			-0.62
BCLTP6-127	2	ENSG00000180747	SMG1P3	SMG1P3, nonsense mediated mRNA decay associated PI3K related kinase pseudogene 3 [Source:HGNC Symbol;Acc:HGNC:49860]	-0.40
BCLTP6-128	6	ENSG00000134057	CCNB1	cyclin B1 [Source:HGNC Symbol;Acc:HGNC:1579]	0.97
BCLTP6-128	6	ENSG00000007968	E2F2	E2F transcription factor 2 [Source:HGNC Symbol;Acc:HGNC:3114]	0.72
BCLTP6-128	6	ENSG00000251546	IGKV1D-39	immunoglobulin kappa variable 1D-39 [Source:HGNC Symbol;Acc:HGNC:5756]	0.83
BCLTP6-128	6	ENSG00000117650	NEK2	NIMA related kinase 2 [Source:HGNC Symbol;Acc:HGNC:7745]	1.22
BCLTP6-128	6	ENSG00000123473	STIL	SCL/TAL1 interrupting locus [Source:HGNC Symbol;Acc:HGNC:10879]	0.77
BCLTP6-128	6	ENSG00000167553	TUBA1C	tubulin alpha 1c [Source:HGNC Symbol;Acc:HGNC:20768]	0.57
BCLTP6-129	3	ENSG00000179750	APOBEC3B	apolipoprotein B mRNA editing enzyme catalytic subunit 3B [Source:HGNC Symbol;Acc:HGNC:17352]	0.98
BCLTP6-129	3	ENSG00000087586	AURKA	aurora kinase A [Source:HGNC Symbol;Acc:HGNC:11393]	0.96
BCLTP6-129	3	ENSG00000164045	CDC25A	cell division cycle 25A [Source:HGNC Symbol;Acc:HGNC:1725]	1.66
BCLTP6-130	8	ENSG00000165895	ARHGAP42	Rho GTPase activating protein 42 [Source:HGNC Symbol;Acc:HGNC:26545]	0.59
BCLTP6-130	8	ENSG00000153162	BMP6	bone morphogenetic protein 6 [Source:HGNC Symbol;Acc:HGNC:1073]	1.06
BCLTP6-130	8	ENSG00000211897	IGHG3	immunoglobulin heavy constant gamma 3 (G3m marker) [Source:HGNC Symbol;Acc:HGNC:5527]	1.04
BCLTP6-130	8	ENSG00000211592	IGKC	immunoglobulin kappa constant [Source:HGNC Symbol;Acc:HGNC:5716]	0.78
BCLTP6-130	8	ENSG00000176658	MYO1D	myosin ID [Source:HGNC Symbol;Acc:HGNC:7598]	0.90
BCLTP6-130	8	ENSG00000163694	RBM47	RNA binding motif protein 47 [Source:HGNC Symbol;Acc:HGNC:30358]	1.07
BCLTP6-130	8	ENSG00000198794	SCAMP5	secretory carrier membrane protein 5 [Source:HGNC Symbol;Acc:HGNC:30386]	1.17
BCLTP6-130	8	ENSG00000103257	SLC7A5	solute carrier family 7 member 5 [Source:HGNC Symbol;Acc:HGNC:11063]	1.11
BCLTP6-131	7	ENSG00000197476			1.60
BCLTP6-131	7	ENSG00000150967	ABCB9	ATP binding cassette subfamily B member 9 [Source:HGNC Symbol;Acc:HGNC:50]	0.90
BCLTP6-131	7	ENSG00000140743	CDR2	cerebellar degeneration related protein 2 [Source:HGNC Symbol;Acc:HGNC:1799]	0.62

Cluster ID	Cluster Size	Gene ID	Gene Name	Gene Description	Log_2 FC TIV
BCLTP6-131	7	ENSG00000173486	FKBP2	FK506 binding protein 2 [Source:HGNC Symbol;Acc:HGNC:3718]	0.53
BCLTP6-131	7	ENSG00000103512	NOMO1	NODAL modulator 1 [Source:HGNC Symbol;Acc:HGNC:30060]	0.84
BCLTP6-131	7	ENSG00000102760	RGCC	regulator of cell cycle [Source:HGNC Symbol;Acc:HGNC:20369]	0.68
BCLTP6-131	7	ENSG00000136840	ST6GALNAC4	ST6 N-acetylgalactosaminide alpha-2,6-sialyltransferase 4 [Source:HGNC Symbol;Acc:HGNC:17846]	0.58
BCLTP6-132	4	ENSG00000086062	B4GALT1	beta-1,4-galactosyltransferase 1 [Source:HGNC Symbol;Acc:HGNC:924]	0.68
BCLTP6-132	4	ENSG00000083444	PLOD1	procollagen-lysine,2-oxoglutarate 5-dioxygenase 1 [Source:HGNC Symbol;Acc:HGNC:9081]	0.86
BCLTP6-132	4	ENSG00000186522	SEPT10	septin 10 [Source:HGNC Symbol;Acc:HGNC:14349]	1.01
BCLTP6-132	4	ENSG00000198879	SFMBT2	Scm-like with four mbt domains 2 [Source:HGNC Symbol;Acc:HGNC:20256]	0.59
BCLTP6-133	2	ENSG00000211662	IGLV3-21	immunoglobulin lambda variable 3-21 [Source:HGNC Symbol;Acc:HGNC:5905]	1.61
BCLTP6-133	2	ENSG00000137812	KNL1	kinetochore scaffold 1 [Source:HGNC Symbol;Acc:HGNC:24054]	0.68
BCLTP6-134	2	ENSG00000241666			-0.90
BCLTP6-134	2	ENSG00000006638	TBXA2R	thromboxane A2 receptor [Source:HGNC Symbol;Acc:HGNC:11608]	-0.82
BCLTP6-135	2	ENSG00000196839	ADA	adenosine deaminase [Source:HGNC Symbol;Acc:HGNC:186]	0.64
BCLTP6-135	2	ENSG00000010310	GIPR	gastric inhibitory polypeptide receptor [Source:HGNC Symbol;Acc:HGNC:4271]	0.65
BCLTP6-136	3	ENSG00000188486	H2AFX	H2A histone family member X [Source:HGNC Symbol;Acc:HGNC:4739]	0.48
BCLTP6-136	3	ENSG00000123416	TUBA1B	tubulin alpha 1b [Source:HGNC Symbol;Acc:HGNC:18809]	0.47
BCLTP6-136	3	ENSG00000140105	WARS	tryptophanyl-tRNA synthetase [Source:HGNC Symbol;Acc:HGNC:12729]	0.77
BCLTP6-137	4	ENSG00000101412	E2F1	E2F transcription factor 1 [Source:HGNC Symbol;Acc:HGNC:3113]	0.88
BCLTP6-137	4	ENSG00000211669	IGLV3-10	immunoglobulin lambda variable 3-10 [Source:HGNC Symbol;Acc:HGNC:5897]	1.00
BCLTP6-137	4	ENSG00000211659	IGLV3-25	immunoglobulin lambda variable 3-25 [Source:HGNC Symbol;Acc:HGNC:5908]	1.02
BCLTP6-137	4	ENSG00000077152	UBE2T	ubiquitin conjugating enzyme E2 T [Source:HGNC Symbol;Acc:HGNC:25009]	0.87
BCLTP6-138	3	ENSG00000156970	BUB1B	BUB1 mitotic checkpoint serine/threonine kinase B [Source:HGNC Symbol;Acc:HGNC:1149]	1.04
BCLTP6-138	3	ENSG00000175984	DENND2C	DENN domain containing 2C [Source:HGNC Symbol;Acc:HGNC:24748]	0.98
BCLTP6-138	3	ENSG00000139734	DIAPH3	diaphanous related formin 3 [Source:HGNC Symbol;Acc:HGNC:15480]	2.00
BCLTP6-139	4	ENSG00000222041	CYTOR	cytoskeleton regulator RNA [Source:HGNC Symbol;Acc:HGNC:28717]	1.05
BCLTP6-139	4	ENSG00000254174	IGHV1-12	immunoglobulin heavy variable 1-12 (pseudogene) [Source:HGNC Symbol;Acc:HGNC:5546]	1.76
BCLTP6-139	4	ENSG00000259997	IGHV1OR16-4	immunoglobulin heavy variable 1/OR16-4 (pseudogene) [Source:HGNC Symbol;Acc:HGNC:5573]	2.13
BCLTP6-139	4	ENSG00000211673	IGLV3-1	immunoglobulin lambda variable 3-1 [Source:HGNC Symbol;Acc:HGNC:5896]	1.06
BCLTP6-140	2	ENSG00000279873	LINC01126	long intergenic non-protein coding RNA 1126 [Source:HGNC Symbol;Acc:HGNC:49275]	-0.74
BCLTP6-140	2	ENSG00000233806	LINC01237	long intergenic non-protein coding RNA 1237 [Source:HGNC Symbol;Acc:HGNC:49793]	-0.50
BCLTP6-141	3	ENSG00000141753	IGFBP4	insulin like growth factor binding protein 4 [Source:HGNC Symbol;Acc:HGNC:5473]	-0.61
BCLTP6-141	3	ENSG00000228903	RASA4CP	RAS p21 protein activator 4C, pseudogene [Source:HGNC Symbol;Acc:HGNC:44185]	-0.65
BCLTP6-141	3	ENSG00000196263	ZNF471	zinc finger protein 471 [Source:HGNC Symbol;Acc:HGNC:23226]	-0.35

Cluster ID	Cluster Size	Gene ID	Gene Name	Gene Description	Log_2 FC TIV
BCLTP6-142	3	ENSG00000213430	HSPD1P1	heat shock protein family D (Hsp60) member 1 pseudogene 1 [Source:HGNC Symbol;Acc:HGNC:35133]	0.80
BCLTP6-142	3	ENSG00000163507	KIAA1524	KIAA1524 [Source:HGNC Symbol;Acc:HGNC:29302]	0.59
BCLTP6-142	3	ENSG00000154719	MRPL39	mitochondrial ribosomal protein L39 [Source:HGNC Symbol;Acc:HGNC:14027]	0.61
BCLTP6-143	4	ENSG00000211967	IGHV3-53	immunoglobulin heavy variable 3-53 [Source:HGNC Symbol;Acc:HGNC:5610]	0.67
BCLTP6-143	4	ENSG00000211972	IGHV3-66	immunoglobulin heavy variable 3-66 [Source:HGNC Symbol;Acc:HGNC:5619]	1.06
BCLTP6-143	4	ENSG00000240382	IGKV1-17	immunoglobulin kappa variable 1-17 [Source:HGNC Symbol;Acc:HGNC:5733]	0.71
BCLTP6-143	4	ENSG00000211670	IGLV3-9	immunoglobulin lambda variable 3-9 (gene/pseudogene) [Source:HGNC Symbol;Acc:HGNC:5918]	0.75
BCLTP6-144	3	ENSG00000279278			-0.65
BCLTP6-144	3	ENSG00000178127	NDUFV2	NADH:ubiquinone oxidoreductase core subunit V2 [Source:HGNC Symbol;Acc:HGNC:7717]	-0.40
BCLTP6-144	3	ENSG00000197714	ZNF460	zinc finger protein 460 [Source:HGNC Symbol;Acc:HGNC:21628]	-0.53
BCLTP6-145	2	ENSG00000254176	IGHV3-75	immunoglobulin heavy variable 3-75 (pseudogene) [Source:HGNC Symbol;Acc:HGNC:5625]	0.62
BCLTP6-145	2	ENSG00000253131	IGHV7-56	immunoglobulin heavy variable 7-56 (pseudogene) [Source:HGNC Symbol;Acc:HGNC:5667]	1.97
BCLTP6-146	2	ENSG00000211933	IGHV6-1	immunoglobulin heavy variable 6-1 [Source:HGNC Symbol;Acc:HGNC:5662]	0.79
BCLTP6-146	2	ENSG00000104889	RNASEH2A	ribonuclease H2 subunit A [Source:HGNC Symbol;Acc:HGNC:18518]	0.38
BCLTP6-147	4	ENSG00000241294	IGKV2-24	immunoglobulin kappa variable 2-24 [Source:HGNC Symbol;Acc:HGNC:5781]	1.04
BCLTP6-147	4	ENSG00000253818	IGLV1-41	immunoglobulin lambda variable 1-41 (pseudogene) [Source:HGNC Symbol;Acc:HGNC:5878]	1.43
BCLTP6-147	4	ENSG00000211644	IGLV1-51	immunoglobulin lambda variable 1-51 [Source:HGNC Symbol;Acc:HGNC:5882]	1.37
BCLTP6-147	4	ENSG00000107719	PALD1	phosphatase domain containing, paladin 1 [Source:HGNC Symbol;Acc:HGNC:23530]	0.53
BCLTP6-148	6	ENSG00000255026			-0.61
BCLTP6-148	6	ENSG00000274272			-0.60
BCLTP6-148	6	ENSG00000168209	DDIT4	DNA damage inducible transcript 4 [Source:HGNC Symbol;Acc:HGNC:24944]	-0.83
BCLTP6-148	6	ENSG00000220008	LINGO3	leucine rich repeat and Ig domain containing 3 [Source:HGNC Symbol;Acc:HGNC:21206]	-0.65
BCLTP6-148	6	ENSG00000172548	NIPAL4	NIPA like domain containing 4 [Source:HGNC Symbol;Acc:HGNC:28018]	-0.76
BCLTP6-148	6	ENSG00000080546	SESN1	sestrin 1 [Source:HGNC Symbol;Acc:HGNC:21595]	-0.62

Table 32: Co-expressed gene clusters (B Cells, Day 6)

Cluster ID	Cluster Size	Gene ID	Gene Name	Gene Description	Log_2 FC TIV
BCLTP7-001	2	ENSG00000198856	OSTC	oligosaccharyltransferase complex non-catalytic subunit [Source:HGNC Symbol;Acc:HGNC:24448]	0.69
BCLTP7-001	2	ENSG00000057657	PRDM1	PR/SET domain 1 [Source:HGNC Symbol;Acc:HGNC:9346]	0.64
BCLTP7-002	2	ENSG00000173540	GMPPB	GDP-mannose pyrophosphorylase B [Source:HGNC Symbol;Acc:HGNC:22932]	0.65
BCLTP7-002	2	ENSG00000163053	SLC16A14	solute carrier family 16 member 14 [Source:HGNC Symbol;Acc:HGNC:26417]	1.23
BCLTP7-003	2	ENSG00000134285	FKBP11	FK506 binding protein 11 [Source:HGNC Symbol;Acc:HGNC:18624]	0.93
BCLTP7-003	2	ENSG00000143333	RGS16	regulator of G-protein signaling 16 [Source:HGNC Symbol;Acc:HGNC:9997]	1.10

Cluster ID	Cluster Size	Gene ID	Gene Name	Gene Description	Log_2 FC TIV
BCLTP7-004	2	ENSG00000102580	DNAJC3	DnaJ heat shock protein family (Hsp40) member C3 [Source:HGNC Symbol;Acc:HGNC:9439]	0.70
BCLTP7-004	2	ENSG00000170476	MZB1	marginal zone B and B1 cell specific protein [Source:HGNC Symbol;Acc:HGNC:30125]	0.95
BCLTP7-005	2	ENSG00000163902	RPN1	ribophorin I [Source:HGNC Symbol;Acc:HGNC:10381]	0.76
BCLTP7-005	2	ENSG00000118705	RPN2	ribophorin II [Source:HGNC Symbol;Acc:HGNC:10382]	0.87
BCLTP7-006	2	ENSG00000074695	LMAN1	lectin, mannose binding 1 [Source:HGNC Symbol;Acc:HGNC:6631]	0.85
BCLTP7-006	2	ENSG00000153066	TXNDC11	thioredoxin domain containing 11 [Source:HGNC Symbol;Acc:HGNC:28030]	0.86
BCLTP7-007	3	ENSG00000143870	PDIA6	protein disulfide isomerase family A member 6 [Source:HGNC Symbol;Acc:HGNC:30168]	0.74
BCLTP7-007	3	ENSG00000197157	SND1	staphylococcal nuclease and tudor domain containing 1 [Source:HGNC Symbol;Acc:HGNC:30646]	0.48
BCLTP7-007	3	ENSG00000116649	SRM	spermidine synthase [Source:HGNC Symbol;Acc:HGNC:11296]	0.58
BCLTP7-008	2	ENSG00000135916	ITM2C	integral membrane protein 2C [Source:HGNC Symbol;Acc:HGNC:6175]	1.07
BCLTP7-008	2	ENSG00000074842	MYDGF	myeloid derived growth factor [Source:HGNC Symbol;Acc:HGNC:16948]	1.30
BCLTP7-009	2	ENSG00000128595	CALU	calumenin [Source:HGNC Symbol;Acc:HGNC:1458]	0.74
BCLTP7-009	2	ENSG00000113615	SEC24A	SEC24 homolog A, COPII coat complex component [Source:HGNC Symbol;Acc:HGNC:10703]	0.91
BCLTP7-010	2	ENSG00000166598	HSP90B1	heat shock protein 90 beta family member 1 [Source:HGNC Symbol;Acc:HGNC:12028]	1.18
BCLTP7-010	2	ENSG00000203914	HSP90B3P	heat shock protein 90 beta family member 3, pseudogene [Source:HGNC Symbol;Acc:HGNC:12100]	1.28
BCLTP7-011	2	ENSG00000164611	PTTG1	pituitary tumor-transforming 1 [Source:HGNC Symbol;Acc:HGNC:9690]	0.76
BCLTP7-011	2	ENSG00000104635	SLC39A14	solute carrier family 39 member 14 [Source:HGNC Symbol;Acc:HGNC:20858]	0.49
BCLTP7-012	2	ENSG00000002549	LAP3	leucine aminopeptidase 3 [Source:HGNC Symbol;Acc:HGNC:18449]	0.71
BCLTP7-012	2	ENSG00000228589	SPCS2P4	signal peptidase complex subunit 2 homolog (S. cerevisiae) pseudogene 4 [Source:HGNC Symbol;Acc:HGNC:45237]	0.69
BCLTP7-013	3	ENSG00000044574	HSPA5	heat shock protein family A (Hsp70) member 5 [Source:HGNC Symbol;Acc:HGNC:5238]	0.97
BCLTP7-013	3	ENSG00000070081	NUCB2	nucleobindin 2 [Source:HGNC Symbol;Acc:HGNC:8044]	0.97
BCLTP7-013	3	ENSG00000166794	PPIB	peptidylprolyl isomerase B [Source:HGNC Symbol;Acc:HGNC:9255]	0.98
BCLTP7-014	2	ENSG00000134153	EMC7	ER membrane protein complex subunit 7 [Source:HGNC Symbol;Acc:HGNC:24301]	0.60
BCLTP7-014	2	ENSG00000068912	ERLEC1	endoplasmic reticulum lectin 1 [Source:HGNC Symbol;Acc:HGNC:25222]	0.90
BCLTP7-015	2	ENSG00000017483	SLC38A5	solute carrier family 38 member 5 [Source:HGNC Symbol;Acc:HGNC:18070]	0.78
BCLTP7-015	2	ENSG00000182934	SRPRA	SRP receptor alpha subunit [Source:HGNC Symbol;Acc:HGNC:11307]	0.52
BCLTP7-016	2	ENSG00000119523	ALG2	ALG2, alpha-1,3/1,6-mannosyltransferase [Source:HGNC Symbol;Acc:HGNC:23159]	0.37
BCLTP7-016	2	ENSG00000142675	CNKSR1	connector enhancer of kinase suppressor of Ras 1 [Source:HGNC Symbol;Acc:HGNC:19700]	0.63
BCLTP7-017	2	ENSG00000125844	RRBP1	ribosome binding protein 1 [Source:HGNC Symbol;Acc:HGNC:10448]	0.80
BCLTP7-017	2	ENSG00000004866	ST7	suppression of tumorigenicity 7 [Source:HGNC Symbol;Acc:HGNC:11351]	0.74
BCLTP7-018	3	ENSG00000088298	EDEM2	ER degradation enhancing alpha-mannosidase like protein 2 [Source:HGNC Symbol;Acc:HGNC:15877]	0.49
BCLTP7-018	3	ENSG00000101294	HM13	histocompatibility minor 13 [Source:HGNC Symbol;Acc:HGNC:16435]	0.73
BCLTP7-018	3	ENSG00000121073	SLC35B1	solute carrier family 35 member B1 [Source:HGNC Symbol;Acc:HGNC:20798]	0.66

Cluster ID	Cluster Size	Gene ID	Gene Name	Gene Description	Log_2 FC TIV
BCLTP7-019	2	ENSG00000168374	ARF4	ADP ribosylation factor 4 [Source:HGNC Symbol;Acc:HGNC:655]	0.60
BCLTP7-019	2	ENSG00000113811	SELENOK	selenoprotein K [Source:HGNC Symbol;Acc:HGNC:30394]	0.59
BCLTP7-020	2	ENSG00000071539	TRIP13	thyroid hormone receptor interactor 13 [Source:HGNC Symbol;Acc:HGNC:12307]	0.90
BCLTP7-020	2	ENSG00000167553	TUBA1C	tubulin alpha 1c [Source:HGNC Symbol;Acc:HGNC:20768]	0.32
BCLTP7-021	4	ENSG00000184432	COPB2	coatomer protein complex subunit beta 2 [Source:HGNC Symbol;Acc:HGNC:2232]	0.49
BCLTP7-021	4	ENSG00000106105	GARS	glycyl-tRNA synthetase [Source:HGNC Symbol;Acc:HGNC:4162]	0.51
BCLTP7-021	4	ENSG00000136240	KDELR2	KDEL endoplasmic reticulum protein retention receptor 2 [Source:HGNC Symbol;Acc:HGNC:6305]	0.67
BCLTP7-021	4	ENSG00000112893	MAN2A1	mannosidase alpha class 2A member 1 [Source:HGNC Symbol;Acc:HGNC:6824]	0.45
BCLTP7-022	3	ENSG00000090520	DNAJB11	DnaJ heat shock protein family (Hsp40) member B11 [Source:HGNC Symbol;Acc:HGNC:14889]	0.68
BCLTP7-022	3	ENSG00000171444	MCC	mutated in colorectal cancers [Source:HGNC Symbol;Acc:HGNC:6935]	1.03
BCLTP7-022	3	ENSG00000166562	SEC11C	SEC11 homolog C, signal peptidase complex subunit [Source:HGNC Symbol;Acc:HGNC:23400]	0.93
BCLTP7-023	2	ENSG00000011478	QPCTL	glutaminyl-peptide cyclotransferase like [Source:HGNC Symbol;Acc:HGNC:25952]	0.73
BCLTP7-023	2	ENSG00000168701	TMEM208	transmembrane protein 208 [Source:HGNC Symbol;Acc:HGNC:25015]	0.73
BCLTP7-024	4	ENSG00000120697	ALG5	ALG5, dolichyl-phosphate beta-glucosyltransferase [Source:HGNC Symbol;Acc:HGNC:20266]	0.59
BCLTP7-024	4	ENSG00000259706	HSP90B2P	heat shock protein 90 beta family member 2, pseudogene [Source:HGNC Symbol;Acc:HGNC:12099]	1.13
BCLTP7-024	4	ENSG00000132432	SEC61G	Sec61 translocon gamma subunit [Source:HGNC Symbol;Acc:HGNC:18277]	0.77
BCLTP7-024	4	ENSG00000048462	TNFRSF17	TNF receptor superfamily member 17 [Source:HGNC Symbol;Acc:HGNC:11913]	1.04
BCLTP7-025	2	ENSG00000138073	PREB	prolactin regulatory element binding [Source:HGNC Symbol;Acc:HGNC:9356]	0.52
BCLTP7-025	2	ENSG00000184840	TMED9	transmembrane p24 trafficking protein 9 [Source:HGNC Symbol;Acc:HGNC:24878]	0.69
BCLTP7-026	3	ENSG00000127022	CANX	calnexin [Source:HGNC Symbol;Acc:HGNC:1473]	0.46
BCLTP7-026	3	ENSG00000123131	PRDX4	peroxiredoxin 4 [Source:HGNC Symbol;Acc:HGNC:17169]	1.03
BCLTP7-026	3	ENSG00000180879	SSR4	signal sequence receptor subunit 4 [Source:HGNC Symbol;Acc:HGNC:11326]	0.64
BCLTP7-027	2	ENSG00000165272	AQP3	aquaporin 3 (Gill blood group) [Source:HGNC Symbol;Acc:HGNC:636]	0.71
BCLTP7-027	2	ENSG00000108106	UBE2S	ubiquitin conjugating enzyme E2 S [Source:HGNC Symbol;Acc:HGNC:17895]	0.50
BCLTP7-028	5	ENSG00000111291	GPRC5D	G protein-coupled receptor class C group 5 member D [Source:HGNC Symbol;Acc:HGNC:13310]	0.83
BCLTP7-028	5	ENSG00000147649	MTDH	metadherin [Source:HGNC Symbol;Acc:HGNC:29608]	0.46
BCLTP7-028	5	ENSG00000168268	NT5DC2	5'-nucleotidase domain containing 2 [Source:HGNC Symbol;Acc:HGNC:25717]	0.96
BCLTP7-028	5	ENSG00000114850	SSR3	signal sequence receptor subunit 3 [Source:HGNC Symbol;Acc:HGNC:11325]	0.74
BCLTP7-028	5	ENSG00000067167	TRAM1	translocation associated membrane protein 1 [Source:HGNC Symbol;Acc:HGNC:20568]	0.49
BCLTP7-029	3	ENSG00000139193	CD27	CD27 molecule [Source:HGNC Symbol;Acc:HGNC:11922]	0.85
BCLTP7-029	3	ENSG00000182054	IDH2	isocitrate dehydrogenase (NADP(+)) 2, mitochondrial [Source:HGNC Symbol;Acc:HGNC:5383]	0.78
BCLTP7-029	3	ENSG00000095380	NANS	N-acetylneuraminate synthase [Source:HGNC Symbol;Acc:HGNC:19237]	0.57
BCLTP7-030	4	ENSG00000004468	CD38	CD38 molecule [Source:HGNC Symbol;Acc:HGNC:1667]	1.07
BCLTP7-030	4	ENSG00000172115	CYCS	cytochrome c, somatic [Source:HGNC Symbol;Acc:HGNC:19986]	0.43
BCLTP7-030	4	ENSG00000131871	SELENOS	selenoprotein S [Source:HGNC Symbol;Acc:HGNC:30396]	0.88

Cluster ID	Cluster Size	Gene ID	Gene Name	Gene Description	Log_2 FC TIV
BCLTP7-030	4	ENSG00000114902	SPCS1	signal peptidase complex subunit 1 [Source:HGNC Symbol;Acc:HGNC:23401]	0.68
BCLTP7-031	2	ENSG00000182481	KPNA2	karyopherin subunit alpha 2 [Source:HGNC Symbol;Acc:HGNC:6395]	0.62
BCLTP7-031	2	ENSG00000198794	SCAMP5	secretory carrier membrane protein 5 [Source:HGNC Symbol;Acc:HGNC:30386]	1.02
BCLTP7-032	2	ENSG00000100342	APOL1	apolipoprotein L1 [Source:HGNC Symbol;Acc:HGNC:618]	0.74
BCLTP7-032	2	ENSG00000083444	PLOD1	procollagen-lysine,2-oxoglutarate 5-dioxygenase 1 [Source:HGNC Symbol;Acc:HGNC:9081]	0.79
BCLTP7-033	2	ENSG00000198876	DCAF12	DDB1 and CUL4 associated factor 12 [Source:HGNC Symbol;Acc:HGNC:19911]	0.69
BCLTP7-033	2	ENSG00000198833	UBE2J1	ubiquitin conjugating enzyme E2 J1 [Source:HGNC Symbol;Acc:HGNC:17598]	0.53
BCLTP7-034	3	ENSG00000102760	RGCC	regulator of cell cycle [Source:HGNC Symbol;Acc:HGNC:20369]	0.96
BCLTP7-034	3	ENSG00000134910	STT3A	STT3A, catalytic subunit of the oligosaccharyltransferase complex [Source:HGNC Symbol;Acc:HGNC:6172]	0.79
BCLTP7-034	3	ENSG00000134825	TMEM258	transmembrane protein 258 [Source:HGNC Symbol;Acc:HGNC:1164]	0.69
BCLTP7-035	2	ENSG00000241755	IGKV1-9	immunoglobulin kappa variable 1-9 [Source:HGNC Symbol;Acc:HGNC:5744]	0.76
BCLTP7-035	2	ENSG00000143603	KCNN3	potassium calcium-activated channel subfamily N member 3 [Source:HGNC Symbol;Acc:HGNC:6292]	0.66
BCLTP7-036	4	ENSG00000110063	DCPS	decapping enzyme, scavenger [Source:HGNC Symbol;Acc:HGNC:29812]	0.64
BCLTP7-036	4	ENSG00000146733	PSPH	phosphoserine phosphatase [Source:HGNC Symbol;Acc:HGNC:9577]	0.63
BCLTP7-036	4	ENSG00000106803	SEC61B	Sec61 translocon beta subunit [Source:HGNC Symbol;Acc:HGNC:16993]	0.82
BCLTP7-036	4	ENSG00000136810	TXN	thioredoxin [Source:HGNC Symbol;Acc:HGNC:12435]	0.60
BCLTP7-037	5	ENSG00000183508	FAM46C	family with sequence similarity 46 member C [Source:HGNC Symbol;Acc:HGNC:24712]	0.83
BCLTP7-037	5	ENSG00000106415	GLCCI1	glucocorticoid induced 1 [Source:HGNC Symbol;Acc:HGNC:18713]	0.78
BCLTP7-037	5	ENSG00000163694	RBM47	RNA binding motif protein 47 [Source:HGNC Symbol;Acc:HGNC:30358]	0.80
BCLTP7-037	5	ENSG00000026751	SLAMF7	SLAM family member 7 [Source:HGNC Symbol;Acc:HGNC:21394]	1.09
BCLTP7-037	5	ENSG00000070214	SLC44A1	solute carrier family 44 member 1 [Source:HGNC Symbol;Acc:HGNC:18798]	0.89
BCLTP7-038	3	ENSG00000198380	GFPT1	glutamine–fructose-6-phosphate transaminase 1 [Source:HGNC Symbol;Acc:HGNC:4241]	0.55
BCLTP7-038	3	ENSG00000155304	HSPA13	heat shock protein family A (Hsp70) member 13 [Source:HGNC Symbol;Acc:HGNC:11375]	0.80
BCLTP7-038	3	ENSG00000071537	SEL1L	SEL1L ERAD E3 ligase adaptor subunit [Source:HGNC Symbol;Acc:HGNC:10717]	0.63
BCLTP7-039	4	ENSG00000179218	CALR	calreticulin [Source:HGNC Symbol;Acc:HGNC:1455]	0.60
BCLTP7-039	4	ENSG00000244038	DDOST	dolichyl-diphosphooligosaccharide–protein glycosyltransferase non-catalytic subunit [Source:HGNC Symbol;Acc:HGNC:2728]	0.75
BCLTP7-039	4	ENSG00000185624	P4HB	prolyl 4-hydroxylase subunit beta [Source:HGNC Symbol;Acc:HGNC:8548]	0.76
BCLTP7-039	4	ENSG00000122862	SRGN	serglycin [Source:HGNC Symbol;Acc:HGNC:9361]	0.62
BCLTP7-040	2	ENSG00000108829	LRRC59	leucine rich repeat containing 59 [Source:HGNC Symbol;Acc:HGNC:28817]	0.70
BCLTP7-040	2	ENSG00000103257	SLC7A5	solute carrier family 7 member 5 [Source:HGNC Symbol;Acc:HGNC:11063]	0.96
BCLTP7-041	3	ENSG00000049656	CLPTM1L	CLPTM1 like [Source:HGNC Symbol;Acc:HGNC:24308]	0.55
BCLTP7-041	3	ENSG00000211653	IGLV1-40	immunoglobulin lambda variable 1-40 [Source:HGNC Symbol;Acc:HGNC:5877]	1.90
BCLTP7-041	3	ENSG00000110917	MLEC	malectin [Source:HGNC Symbol;Acc:HGNC:28973]	0.54
BCLTP7-042	3	ENSG00000112237	CCNC	cyclin C [Source:HGNC Symbol;Acc:HGNC:1581]	0.43

Cluster ID	Cluster Size	Gene ID	Gene Name	Gene Description	Log_2 FC TIV
BCLTP7-042	3	ENSG00000184164	CRELD2	cysteine rich with EGF like domains 2 [Source:HGNC Symbol;Acc:HGNC:28150]	0.73
BCLTP7-042	3	ENSG00000239264	TXNDC5	thioredoxin domain containing 5 [Source:HGNC Symbol;Acc:HGNC:21073]	1.06
BCLTP7-043	2	ENSG00000169679	BUB1	BUB1 mitotic checkpoint serine/threonine kinase [Source:HGNC Symbol;Acc:HGNC:1148]	0.93
BCLTP7-043	2	ENSG00000122952	ZWINT	ZW10 interacting kinetochore protein [Source:HGNC Symbol;Acc:HGNC:13195]	0.99
BCLTP7-044	2	ENSG00000211952	IGHV4-28	immunoglobulin heavy variable 4-28 [Source:HGNC Symbol;Acc:HGNC:5645]	0.66
BCLTP7-044	2	ENSG00000146918	NCAPG2	non-SMC condensin II complex subunit G2 [Source:HGNC Symbol;Acc:HGNC:21904]	0.33
BCLTP7-045	5	ENSG00000197476			1.42
BCLTP7-045	5	ENSG00000149428	HYOU1	hypoxia up-regulated 1 [Source:HGNC Symbol;Acc:HGNC:16931]	0.71
BCLTP7-045	5	ENSG00000103512	NOMO1	NODAL modulator 1 [Source:HGNC Symbol;Acc:HGNC:30060]	0.70
BCLTP7-045	5	ENSG00000185164	NOMO2	NODAL modulator 2 [Source:HGNC Symbol;Acc:HGNC:22652]	0.93
BCLTP7-045	5	ENSG00000115902	SLC1A4	solute carrier family 1 member 4 [Source:HGNC Symbol;Acc:HGNC:10942]	0.65
BCLTP7-046	3	ENSG00000164045	CDC25A	cell division cycle 25A [Source:HGNC Symbol;Acc:HGNC:1725]	1.18
BCLTP7-046	3	ENSG00000211897	IGHG3	immunoglobulin heavy constant gamma 3 (G3m marker) [Source:HGNC Symbol;Acc:HGNC:5527]	0.87
BCLTP7-046	3	ENSG00000211892	IGHG4	immunoglobulin heavy constant gamma 4 (G4m marker) [Source:HGNC Symbol;Acc:HGNC:5528]	0.77
BCLTP7-047	2	ENSG00000172469	MANEA	mannosidase endo-alpha [Source:HGNC Symbol;Acc:HGNC:21072]	0.74
BCLTP7-047	2	ENSG00000138768	USO1	USO1 vesicle transport factor [Source:HGNC Symbol;Acc:HGNC:30904]	0.45
BCLTP7-048	2	ENSG00000253755	IGHGP	immunoglobulin heavy constant gamma P (non-functional) [Source:HGNC Symbol;Acc:HGNC:5529]	1.77
BCLTP7-048	2	ENSG00000186818	LILRB4	leukocyte immunoglobulin like receptor B4 [Source:HGNC Symbol;Acc:HGNC:6608]	0.65
BCLTP7-049	3	ENSG00000171155	C1GALT1C1	C1GALT1 specific chaperone 1 [Source:HGNC Symbol;Acc:HGNC:24338]	0.88
BCLTP7-049	3	ENSG00000075218	GTSE1	G2 and S-phase expressed 1 [Source:HGNC Symbol;Acc:HGNC:13698]	0.85
BCLTP7-049	3	ENSG00000100228	RAB36	RAB36, member RAS oncogene family [Source:HGNC Symbol;Acc:HGNC:9775]	0.49
BCLTP7-050	3	ENSG00000198937	CCDC167	coiled-coil domain containing 167 [Source:HGNC Symbol;Acc:HGNC:21239]	0.73
BCLTP7-050	3	ENSG00000111885	MAN1A1	mannosidase alpha class 1A member 1 [Source:HGNC Symbol;Acc:HGNC:6821]	0.94
BCLTP7-050	3	ENSG00000173334	TRIB1	tribbles pseudokinase 1 [Source:HGNC Symbol;Acc:HGNC:16891]	0.94
BCLTP7-051	3	ENSG00000102096	PIM2	Pim-2 proto-oncogene, serine/threonine kinase [Source:HGNC Symbol;Acc:HGNC:8987]	0.71
BCLTP7-051	3	ENSG00000117143	UAP1	UDP-N-acetylglucosamine pyrophosphorylase 1 [Source:HGNC Symbol;Acc:HGNC:12457]	0.68
BCLTP7-051	3	ENSG00000154277	UCHL1	ubiquitin C-terminal hydrolase L1 [Source:HGNC Symbol;Acc:HGNC:12513]	1.97
BCLTP7-052	2	ENSG00000131153	GINS2	GINS complex subunit 2 [Source:HGNC Symbol;Acc:HGNC:24575]	0.56
BCLTP7-052	2	ENSG00000101057	MYBL2	MYB proto-oncogene like 2 [Source:HGNC Symbol;Acc:HGNC:7548]	0.91
BCLTP7-053	2	ENSG00000149554	CHEK1	checkpoint kinase 1 [Source:HGNC Symbol;Acc:HGNC:1925]	0.55
BCLTP7-053	2	ENSG00000148773	MKI67	marker of proliferation Ki-67 [Source:HGNC Symbol;Acc:HGNC:7107]	0.76
BCLTP7-054	3	ENSG00000108641	B9D1	B9 domain containing 1 [Source:HGNC Symbol;Acc:HGNC:24123]	0.95

Cluster ID	Cluster Size	Gene ID	Gene Name	Gene Description	Log_2 FC TIV
BCLTP7-054	3	ENSG00000211964	IGHV3-48	immunoglobulin heavy variable 3-48 [Source:HGNC Symbol;Acc:HGNC:5606]	0.81
BCLTP7-054	3	ENSG00000204386	NEU1	neuraminidase 1 [Source:HGNC Symbol;Acc:HGNC:7758]	0.63
BCLTP7-055	3	ENSG00000087586	AURKA	aurora kinase A [Source:HGNC Symbol;Acc:HGNC:11393]	0.83
BCLTP7-055	3	ENSG00000112378	PERP	PERP, TP53 apoptosis effector [Source:HGNC Symbol;Acc:HGNC:17637]	0.79
BCLTP7-055	3	ENSG00000171241	SHCBP1	SHC binding and spindle associated 1 [Source:HGNC Symbol;Acc:HGNC:29547]	1.02
BCLTP7-056	2	ENSG00000108578	BLMH	bleomycin hydrolase [Source:HGNC Symbol;Acc:HGNC:1059]	0.36
BCLTP7-056	2	ENSG00000129235	TXNDC17	thioredoxin domain containing 17 [Source:HGNC Symbol;Acc:HGNC:28218]	0.65
BCLTP7-057	2	ENSG00000094804	CDC6	cell division cycle 6 [Source:HGNC Symbol;Acc:HGNC:1744]	0.96
BCLTP7-057	2	ENSG00000178445	GLDC	glycine decarboxylase [Source:HGNC Symbol;Acc:HGNC:4313]	1.28
BCLTP7-058	3	ENSG00000093009	CDC45	cell division cycle 45 [Source:HGNC Symbol;Acc:HGNC:1739]	1.01
BCLTP7-058	3	ENSG00000242766	IGKV1D-17	immunoglobulin kappa variable 1D-17 [Source:HGNC Symbol;Acc:HGNC:5749]	1.08
BCLTP7-058	3	ENSG00000165304	MELK	maternal embryonic leucine zipper kinase [Source:HGNC Symbol;Acc:HGNC:16870]	1.35
BCLTP7-059	3	ENSG00000154723	ATP5J	ATP synthase, H+ transporting, mitochondrial Fo complex subunit F6 [Source:HGNC Symbol;Acc:HGNC:847]	0.59
BCLTP7-059	3	ENSG00000132465	JCHAIN	joining chain of multimeric IgA and IgM [Source:HGNC Symbol;Acc:HGNC:5713]	1.02
BCLTP7-059	3	ENSG00000165672	PRDX3	peroxiredoxin 3 [Source:HGNC Symbol;Acc:HGNC:9354]	0.47
BCLTP7-060	2	ENSG00000123485	HJURP	Holliday junction recognition protein [Source:HGNC Symbol;Acc:HGNC:25444]	1.28
BCLTP7-060	2	ENSG00000131747	TOP2A	topoisomerase (DNA) II alpha [Source:HGNC Symbol;Acc:HGNC:11989]	0.59
BCLTP7-061	2	ENSG00000134057	CCNB1	cyclin B1 [Source:HGNC Symbol;Acc:HGNC:1579]	0.74
BCLTP7-061	2	ENSG00000137804	NUSAP1	nucleolar and spindle associated protein 1 [Source:HGNC Symbol;Acc:HGNC:18538]	0.55
BCLTP7-062	2	ENSG00000198855	FICD	FIC domain containing [Source:HGNC Symbol;Acc:HGNC:18416]	0.80
BCLTP7-062	2	ENSG00000100883	SRP54	signal recognition particle 54 [Source:HGNC Symbol;Acc:HGNC:11301]	0.53
BCLTP7-063	2	ENSG00000090104	RGS1	regulator of G-protein signaling 1 [Source:HGNC Symbol;Acc:HGNC:9991]	1.12
BCLTP7-063	2	ENSG00000116741	RGS2	regulator of G-protein signaling 2 [Source:HGNC Symbol;Acc:HGNC:9998]	0.58
BCLTP7-064	5	ENSG00000086062	B4GALT1	beta-1,4-galactosyltransferase 1 [Source:HGNC Symbol;Acc:HGNC:924]	0.62
BCLTP7-064	5	ENSG00000105438	KDELR1	KDEL endoplasmic reticulum protein retention receptor 1 [Source:HGNC Symbol;Acc:HGNC:6304]	0.48
BCLTP7-064	5	ENSG00000169223	LMAN2	lectin, mannose binding 2 [Source:HGNC Symbol;Acc:HGNC:16986]	0.68
BCLTP7-064	5	ENSG00000120725	SIL1	SIL1 nucleotide exchange factor [Source:HGNC Symbol;Acc:HGNC:24624]	0.74
BCLTP7-064	5	ENSG00000136840	ST6GALNAC4	ST6 N-acetylgalactosaminide alpha-2,6-sialyltransferase 4 [Source:HGNC Symbol;Acc:HGNC:17846]	0.62
BCLTP7-065	4	ENSG00000105974	CAV1	caveolin 1 [Source:HGNC Symbol;Acc:HGNC:1527]	1.77
BCLTP7-065	4	ENSG00000163754	GYG1	glycogenin 1 [Source:HGNC Symbol;Acc:HGNC:4699]	0.54
BCLTP7-065	4	ENSG00000074416	MGLL	monoglyceride lipase [Source:HGNC Symbol;Acc:HGNC:17038]	0.78
BCLTP7-065	4	ENSG00000100219	XBP1	X-box binding protein 1 [Source:HGNC Symbol;Acc:HGNC:12801]	1.25
BCLTP7-066	4	ENSG00000085063	CD59	CD59 molecule [Source:HGNC Symbol;Acc:HGNC:1689]	0.47
BCLTP7-066	4	ENSG00000111640	GAPDH	glyceraldehyde-3-phosphate dehydrogenase [Source:HGNC Symbol;Acc:HGNC:4141]	0.58
BCLTP7-066	4	ENSG00000137563	GGH	gamma-glutamyl hydrolase [Source:HGNC Symbol;Acc:HGNC:4248]	1.07

Cluster ID	Cluster Size	Gene ID	Gene Name	Gene Description	Log_2 FC TIV
BCLTP7-066	4	ENSG00000241351	IGKV3-11	immunoglobulin kappa variable 3-11 [Source:HGNC Symbol;Acc:HGNC:5815]	0.70
BCLTP7-067	3	ENSG00000153093	ACOXL	acyl-CoA oxidase-like [Source:HGNC Symbol;Acc:HGNC:25621]	1.84
BCLTP7-067	3	ENSG00000136026	CKAP4	cytoskeleton associated protein 4 [Source:HGNC Symbol;Acc:HGNC:16991]	0.86
BCLTP7-067	3	ENSG00000154511	FAM69A	family with sequence similarity 69 member A [Source:HGNC Symbol;Acc:HGNC:32213]	0.53
BCLTP7-068	2	ENSG00000282600			0.92
BCLTP7-068	2	ENSG00000113368	LMNB1	lamin B1 [Source:HGNC Symbol;Acc:HGNC:6637]	0.37
BCLTP7-069	2	ENSG00000165895	ARHGAP42	Rho GTPase activating protein 42 [Source:HGNC Symbol;Acc:HGNC:26545]	0.39
BCLTP7-069	2	ENSG00000185155	MIXL1	Mix paired-like homeobox [Source:HGNC Symbol;Acc:HGNC:13363]	0.78
BCLTP7-070	4	ENSG00000173486	FKBP2	FK506 binding protein 2 [Source:HGNC Symbol;Acc:HGNC:3718]	0.59
BCLTP7-070	4	ENSG00000115677	HDLBP	high density lipoprotein binding protein [Source:HGNC Symbol;Acc:HGNC:4857]	0.68
BCLTP7-070	4	ENSG00000167861	HID1	HID1 domain containing [Source:HGNC Symbol;Acc:HGNC:15736]	0.93
BCLTP7-070	4	ENSG00000144867	SRPRB	SRP receptor beta subunit [Source:HGNC Symbol;Acc:HGNC:24085]	0.58
BCLTP7-071	2	ENSG00000157456	CCNB2	cyclin B2 [Source:HGNC Symbol;Acc:HGNC:1580]	0.67
BCLTP7-071	2	ENSG00000166851	PLK1	polo like kinase 1 [Source:HGNC Symbol;Acc:HGNC:9077]	0.44
BCLTP7-072	2	ENSG00000136010	ALDH1L2	aldehyde dehydrogenase 1 family member L2 [Source:HGNC Symbol;Acc:HGNC:26777]	1.26
BCLTP7-072	2	ENSG00000087502	ERGIC2	ERGIC and golgi 2 [Source:HGNC Symbol;Acc:HGNC:30208]	0.46
BCLTP7-073	2	ENSG00000254174	IGHV1-12	immunoglobulin heavy variable 1-12 (pseudogene) [Source:HGNC Symbol;Acc:HGNC:5546]	2.21
BCLTP7-073	2	ENSG00000154719	MRPL39	mitochondrial ribosomal protein L39 [Source:HGNC Symbol;Acc:HGNC:14027]	0.34
BCLTP7-074	3	ENSG00000089685	BIRC5	baculoviral IAP repeat containing 5 [Source:HGNC Symbol;Acc:HGNC:593]	0.91
BCLTP7-074	3	ENSG00000007968	E2F2	E2F transcription factor 2 [Source:HGNC Symbol;Acc:HGNC:3114]	0.45
BCLTP7-074	3	ENSG00000176890	TYMS	thymidylate synthetase [Source:HGNC Symbol;Acc:HGNC:12441]	1.13
BCLTP7-075	2	ENSG00000140743	CDR2	cerebellar degeneration related protein 2 [Source:HGNC Symbol;Acc:HGNC:1799]	0.47
BCLTP7-075	2	ENSG00000211938	IGHV3-7	immunoglobulin heavy variable 3-7 [Source:HGNC Symbol;Acc:HGNC:5620]	1.93
BCLTP7-076	2	ENSG00000123975	CKS2	CDC28 protein kinase regulatory subunit 2 [Source:HGNC Symbol;Acc:HGNC:2000]	0.59
BCLTP7-076	2	ENSG00000107104	KANK1	KN motif and ankyrin repeat domains 1 [Source:HGNC Symbol;Acc:HGNC:19309]	1.05
BCLTP7-077	3	ENSG00000117724	CENPF	centromere protein F [Source:HGNC Symbol;Acc:HGNC:1857]	0.42
BCLTP7-077	3	ENSG00000143228	NUF2	NUF2, NDC80 kinetochore complex component [Source:HGNC Symbol;Acc:HGNC:14621]	0.52
BCLTP7-077	3	ENSG00000085840	ORC1	origin recognition complex subunit 1 [Source:HGNC Symbol;Acc:HGNC:8487]	1.02
BCLTP7-078	2	ENSG00000111665	CDCA3	cell division cycle associated 3 [Source:HGNC Symbol;Acc:HGNC:14624]	0.78
BCLTP7-078	2	ENSG00000068489	PRR11	proline rich 11 [Source:HGNC Symbol;Acc:HGNC:25619]	0.51
BCLTP7-079	5	ENSG00000172339	ALG14	ALG14, UDP-N-acetylglucosaminyltransferase subunit [Source:HGNC Symbol;Acc:HGNC:28287]	0.63
BCLTP7-079	5	ENSG00000143641	GALNT2	polypeptide N-acetylgalactosaminyltransferase 2 [Source:HGNC Symbol;Acc:HGNC:4124]	0.48
BCLTP7-079	5	ENSG00000211677	IGLC2	immunoglobulin lambda constant 2 [Source:HGNC Symbol;Acc:HGNC:5856]	1.43
BCLTP7-079	5	ENSG00000099337	KCNK6	potassium two pore domain channel subfamily K member 6 [Source:HGNC Symbol;Acc:HGNC:6281]	0.59

Cluster ID	Cluster Size	Gene ID	Gene Name	Gene Description	Log_2 FC TIV
BCLTP7-079	5	ENSG00000167004	PDIA3	protein disulfide isomerase family A member 3 [Source:HGNC Symbol;Acc:HGNC:4606]	0.47
BCLTP7-080	3	ENSG00000104290	FZD3	frizzled class receptor 3 [Source:HGNC Symbol;Acc:HGNC:4041]	0.44
BCLTP7-080	3	ENSG00000242076	IGKV1-33	immunoglobulin kappa variable 1-33 [Source:HGNC Symbol;Acc:HGNC:5737]	1.01
BCLTP7-080	3	ENSG00000111186	WNT5B	Wnt family member 5B [Source:HGNC Symbol;Acc:HGNC:16265]	2.24
BCLTP7-081	6	ENSG00000258572			0.86
BCLTP7-081	6	ENSG00000029153	ARNTL2	aryl hydrocarbon receptor nuclear translocator like 2 [Source:HGNC Symbol;Acc:HGNC:18984]	0.77
BCLTP7-081	6	ENSG00000180535	BHLHA15	basic helix-loop-helix family member a15 [Source:HGNC Symbol;Acc:HGNC:22265]	1.03
BCLTP7-081	6	ENSG00000176658	MYO1D	myosin ID [Source:HGNC Symbol;Acc:HGNC:7598]	0.66
BCLTP7-081	6	ENSG00000065308	TRAM2	translocation associated membrane protein 2 [Source:HGNC Symbol;Acc:HGNC:16855]	0.79
BCLTP7-081	6	ENSG00000108953	YWHAE	tyrosine 3-monooxygenase/tryptophan 5-monooxygenase activation protein epsilon [Source:HGNC Symbol;Acc:HGNC:12851]	0.44
BCLTP7-082	3	ENSG00000145386	CCNA2	cyclin A2 [Source:HGNC Symbol;Acc:HGNC:1578]	0.51
BCLTP7-082	3	ENSG00000211679	IGLC3	immunoglobulin lambda constant 3 (Kern-Oz+ marker) [Source:HGNC Symbol;Acc:HGNC:5857]	0.76
BCLTP7-082	3	ENSG00000076003	MCM6	minichromosome maintenance complex component 6 [Source:HGNC Symbol;Acc:HGNC:6949]	0.40
BCLTP7-083	2	ENSG00000119326	CTNNAL1	catenin alpha like 1 [Source:HGNC Symbol;Acc:HGNC:2512]	0.89
BCLTP7-083	2	ENSG00000198130	HIBCH	3-hydroxyisobutyryl-CoA hydrolase [Source:HGNC Symbol;Acc:HGNC:4908]	0.41
BCLTP7-084	3	ENSG00000128590	DNAJB9	DnaJ heat shock protein family (Hsp40) member B9 [Source:HGNC Symbol;Acc:HGNC:6968]	0.89
BCLTP7-084	3	ENSG00000069956	MAPK6	mitogen-activated protein kinase 6 [Source:HGNC Symbol;Acc:HGNC:6879]	0.44
BCLTP7-084	3	ENSG00000107833	NPM3	nucleophosmin/nucleoplasmin 3 [Source:HGNC Symbol;Acc:HGNC:7931]	0.34
BCLTP7-085	2	ENSG00000024526	DEPDC1	DEP domain containing 1 [Source:HGNC Symbol;Acc:HGNC:22949]	0.88
BCLTP7-085	2	ENSG00000144724	PTPRG	protein tyrosine phosphatase, receptor type G [Source:HGNC Symbol;Acc:HGNC:9671]	0.76
BCLTP7-086	2	ENSG00000138778	CENPE	centromere protein E [Source:HGNC Symbol;Acc:HGNC:1856]	0.77
BCLTP7-086	2	ENSG00000100629	CEP128	centrosomal protein 128 [Source:HGNC Symbol;Acc:HGNC:20359]	0.81
BCLTP7-087	3	ENSG00000129562	DAD1	defender against cell death 1 [Source:HGNC Symbol;Acc:HGNC:2664]	0.55
BCLTP7-087	3	ENSG00000165948	IFI27L1	interferon alpha inducible protein 27 like 1 [Source:HGNC Symbol;Acc:HGNC:19754]	0.76
BCLTP7-087	3	ENSG00000186594	MIR22HG	MIR22 host gene [Source:HGNC Symbol;Acc:HGNC:28219]	1.09
BCLTP7-088	3	ENSG00000064763	FAR2	fatty acyl-CoA reductase 2 [Source:HGNC Symbol;Acc:HGNC:25531]	0.76
BCLTP7-088	3	ENSG00000211645	IGLV1-50	immunoglobulin lambda variable 1-50 (non-functional) [Source:HGNC Symbol;Acc:HGNC:5881]	1.40
BCLTP7-088	3	ENSG00000160712	IL6R	interleukin 6 receptor [Source:HGNC Symbol;Acc:HGNC:6019]	0.60
BCLTP7-089	7	ENSG00000118985	ELL2	elongation factor for RNA polymerase II 2 [Source:HGNC Symbol;Acc:HGNC:17064]	1.09
BCLTP7-089	7	ENSG00000075420	FNDC3B	fibronectin type III domain containing 3B [Source:HGNC Symbol;Acc:HGNC:24670]	1.22
BCLTP7-089	7	ENSG00000145050	MANF	mesencephalic astrocyte derived neurotrophic factor [Source:HGNC Symbol;Acc:HGNC:15461]	1.03
BCLTP7-089	7	ENSG00000088826	SMOX	spermine oxidase [Source:HGNC Symbol;Acc:HGNC:15862]	1.20
BCLTP7-089	7	ENSG00000123352	SPATS2	spermatogenesis associated serine rich 2 [Source:HGNC Symbol;Acc:HGNC:18650]	0.72

Cluster ID	Cluster Size	Gene ID	Gene Name	Gene Description	Log_2 FC TIV
BCLTP7-089	7	ENSG00000113387	SUB1	SUB1 homolog, transcriptional regulator [Source:HGNC Symbol;Acc:HGNC:19985]	0.59
BCLTP7-089	7	ENSG00000111424	VDR	vitamin D (1,25- dihydroxyvitamin D3) receptor [Source:HGNC Symbol;Acc:HGNC:12679]	0.94
BCLTP7-090	3	ENSG00000211662	IGLV3-21	immunoglobulin lambda variable 3-21 [Source:HGNC Symbol;Acc:HGNC:5905]	1.98
BCLTP7-090	3	ENSG00000138760	SCARB2	scavenger receptor class B member 2 [Source:HGNC Symbol;Acc:HGNC:1665]	0.47
BCLTP7-090	3	ENSG00000145817	YIPF5	Yip1 domain family member 5 [Source:HGNC Symbol;Acc:HGNC:24877]	0.40
BCLTP7-091	3	ENSG00000138166	DUSP5	dual specificity phosphatase 5 [Source:HGNC Symbol;Acc:HGNC:3071]	1.34
BCLTP7-091	3	ENSG00000162676	GFI1	growth factor independent 1 transcriptional repressor [Source:HGNC Symbol;Acc:HGNC:4237]	1.43
BCLTP7-091	3	ENSG00000134291	TMEM106C	transmembrane protein 106C [Source:HGNC Symbol;Acc:HGNC:28775]	0.33
BCLTP7-092	4	ENSG00000280411	IGHV1-69-2	immunoglobulin heavy variable 1-69-2 [Source:HGNC Symbol;Acc:HGNC:5562]	1.12
BCLTP7-092	4	ENSG00000254395	IGHV4-55	immunoglobulin heavy variable 4-55 (pseudogene) [Source:HGNC Symbol;Acc:HGNC:5653]	0.95
BCLTP7-092	4	ENSG00000211592	IGKC	immunoglobulin kappa constant [Source:HGNC Symbol;Acc:HGNC:5716]	0.53
BCLTP7-092	4	ENSG00000211632	IGKV3D-11	immunoglobulin kappa variable 3D-11 [Source:HGNC Symbol;Acc:HGNC:5823]	0.66
BCLTP7-093	3	ENSG00000259772			0.57
BCLTP7-093	3	ENSG00000135069	PSAT1	phosphoserine aminotransferase 1 [Source:HGNC Symbol;Acc:HGNC:19129]	0.67
BCLTP7-093	3	ENSG00000070540	WIPI1	WD repeat domain, phosphoinositide interacting 1 [Source:HGNC Symbol;Acc:HGNC:25471]	0.62
BCLTP7-094	2	ENSG00000248571			1.42
BCLTP7-094	2	ENSG00000118515	SGK1	serum/glucocorticoid regulated kinase 1 [Source:HGNC Symbol;Acc:HGNC:10810]	1.07
BCLTP7-095	3	ENSG00000153162	BMP6	bone morphogenetic protein 6 [Source:HGNC Symbol;Acc:HGNC:1073]	0.96
BCLTP7-095	3	ENSG00000227295	ELL2P1	elongation factor for RNA polymerase II 2 pseudogene 1 [Source:HGNC Symbol;Acc:HGNC:39343]	0.68
BCLTP7-095	3	ENSG00000168653	NDUFS5	NADH:ubiquinone oxidoreductase subunit S5 [Source:HGNC Symbol;Acc:HGNC:7712]	0.36
BCLTP7-096	2	ENSG00000211659	IGLV3-25	immunoglobulin lambda variable 3-25 [Source:HGNC Symbol;Acc:HGNC:5908]	0.95
BCLTP7-096	2	ENSG00000102471	NDFIP2	Nedd4 family interacting protein 2 [Source:HGNC Symbol;Acc:HGNC:18537]	0.93
BCLTP7-097	5	ENSG00000124788	ATXN1	ataxin 1 [Source:HGNC Symbol;Acc:HGNC:10548]	0.76
BCLTP7-097	5	ENSG00000049860	HEXB	hexosaminidase subunit beta [Source:HGNC Symbol;Acc:HGNC:4879]	0.45
BCLTP7-097	5	ENSG00000173848	NET1	neuroepithelial cell transforming 1 [Source:HGNC Symbol;Acc:HGNC:14592]	0.84
BCLTP7-097	5	ENSG00000198792	TMEM184B	transmembrane protein 184B [Source:HGNC Symbol;Acc:HGNC:1310]	0.57
BCLTP7-097	5	ENSG00000173218	VANGL1	VANGL planar cell polarity protein 1 [Source:HGNC Symbol;Acc:HGNC:15512]	0.57
BCLTP7-098	2	ENSG00000243238	IGKV2-30	immunoglobulin kappa variable 2-30 [Source:HGNC Symbol;Acc:HGNC:5785]	0.95
BCLTP7-098	2	ENSG00000239571	IGKV2D-30	immunoglobulin kappa variable 2D-30 [Source:HGNC Symbol;Acc:HGNC:5801]	1.11
BCLTP7-099	6	ENSG00000117399	CDC20	cell division cycle 20 [Source:HGNC Symbol;Acc:HGNC:1723]	0.88
BCLTP7-099	6	ENSG00000143476	DTL	denticleless E3 ubiquitin protein ligase homolog [Source:HGNC Symbol;Acc:HGNC:30288]	1.04
BCLTP7-099	6	ENSG00000109805	NCAPG	non-SMC condensin I complex subunit G [Source:HGNC Symbol;Acc:HGNC:24304]	1.06
BCLTP7-099	6	ENSG00000171848	RRM2	ribonucleotide reductase regulatory subunit M2 [Source:HGNC Symbol;Acc:HGNC:10452]	1.09

Cluster ID	Cluster Size	Gene ID	Gene Name	Gene Description	Log_2 FC TIV
BCLTP7-099	6	ENSG00000154839	SKA1	spindle and kinetochore associated complex subunit 1 [Source:HGNC Symbol;Acc:HGNC:28109]	0.79
BCLTP7-099	6	ENSG00000077152	UBE2T	ubiquitin conjugating enzyme E2 T [Source:HGNC Symbol;Acc:HGNC:25009]	0.95
BCLTP7-100	3	ENSG00000113273	ARSB	arylsulfatase B [Source:HGNC Symbol;Acc:HGNC:714]	0.44
BCLTP7-100	3	ENSG00000182197	EXT1	exostosin glycosyltransferase 1 [Source:HGNC Symbol;Acc:HGNC:3512]	0.44
BCLTP7-100	3	ENSG00000184232	OAF	out at first homolog [Source:HGNC Symbol;Acc:HGNC:28752]	0.94
BCLTP7-101	3	ENSG00000167513	CDT1	chromatin licensing and DNA replication factor 1 [Source:HGNC Symbol;Acc:HGNC:24576]	0.34
BCLTP7-101	3	ENSG00000261834	IGHV3OR16-15	immunoglobulin heavy variable 3/OR16-15 (pseudogene) [Source:HGNC Symbol;Acc:HGNC:5639]	1.89
BCLTP7-101	3	ENSG00000122188	LAX1	lymphocyte transmembrane adaptor 1 [Source:HGNC Symbol;Acc:HGNC:26005]	0.46
BCLTP7-102	2	ENSG00000240382	IGKV1-17	immunoglobulin kappa variable 1-17 [Source:HGNC Symbol;Acc:HGNC:5733]	0.63
BCLTP7-102	2	ENSG00000211670	IGLV3-9	immunoglobulin lambda variable 3-9 (gene/pseudogene) [Source:HGNC Symbol;Acc:HGNC:5918]	1.49
BCLTP7-103	2	ENSG00000211972	IGHV3-66	immunoglobulin heavy variable 3-66 [Source:HGNC Symbol;Acc:HGNC:5619]	0.94
BCLTP7-103	2	ENSG00000211649	IGLV7-46	immunoglobulin lambda variable 7-46 (gene/pseudogene) [Source:HGNC Symbol;Acc:HGNC:5930]	0.73
BCLTP7-104	2	ENSG00000213430	HSPD1P1	heat shock protein family D (Hsp60) member 1 pseudogene 1 [Source:HGNC Symbol;Acc:HGNC:35133]	0.59
BCLTP7-104	2	ENSG00000079931	MOXD1	monooxygenase DBH like 1 [Source:HGNC Symbol;Acc:HGNC:21063]	0.96
BCLTP7-105	2	ENSG00000211935	IGHV1-3	immunoglobulin heavy variable 1-3 [Source:HGNC Symbol;Acc:HGNC:5552]	0.41
BCLTP7-105	2	ENSG00000259997	IGHV1OR16-4	immunoglobulin heavy variable 1/OR16-4 (pseudogene) [Source:HGNC Symbol;Acc:HGNC:5573]	2.21
BCLTP7-106	2	ENSG00000105011	ASF1B	anti-silencing function 1B histone chaperone [Source:HGNC Symbol;Acc:HGNC:20996]	0.58
BCLTP7-106	2	ENSG00000211644	IGLV1-51	immunoglobulin lambda variable 1-51 [Source:HGNC Symbol;Acc:HGNC:5882]	0.71
BCLTP7-107	2	ENSG00000271178	IGHV3OR16-13	immunoglobulin heavy variable 3/OR16-13 (non-functional) [Source:HGNC Symbol;Acc:HGNC:5637]	0.86
BCLTP7-107	2	ENSG00000211668	IGLV2-11	immunoglobulin lambda variable 2-11 [Source:HGNC Symbol;Acc:HGNC:5887]	0.26
BCLTP7-108	4	ENSG00000211946	IGHV3-20	immunoglobulin heavy variable 3-20 [Source:HGNC Symbol;Acc:HGNC:5585]	0.93
BCLTP7-108	4	ENSG00000166803	KIAA0101	KIAA0101 [Source:HGNC Symbol;Acc:HGNC:28961]	0.93
BCLTP7-108	4	ENSG00000164109	MAD2L1	MAD2 mitotic arrest deficient-like 1 (yeast) [Source:HGNC Symbol;Acc:HGNC:6763]	0.62
BCLTP7-108	4	ENSG00000078900	TP73	tumor protein p73 [Source:HGNC Symbol;Acc:HGNC:12003]	0.91
BCLTP7-109	7	ENSG00000129636	ITFG1	integrin alpha FG-GAP repeat containing 1 [Source:HGNC Symbol;Acc:HGNC:30697]	0.49
BCLTP7-109	7	ENSG00000179222	MAGED1	MAGE family member D1 [Source:HGNC Symbol;Acc:HGNC:6813]	0.56
BCLTP7-109	7	ENSG00000239672	NME1	NME/NM23 nucleoside diphosphate kinase 1 [Source:HGNC Symbol;Acc:HGNC:7849]	0.52
BCLTP7-109	7	ENSG00000103226	NOMO3	NODAL modulator 3 [Source:HGNC Symbol;Acc:HGNC:25242]	0.81
BCLTP7-109	7	ENSG00000104889	RNASEH2A	ribonuclease H2 subunit A [Source:HGNC Symbol;Acc:HGNC:18518]	0.20
BCLTP7-109	7	ENSG00000025039	RRAGD	Ras related GTP binding D [Source:HGNC Symbol;Acc:HGNC:19903]	1.02
BCLTP7-109	7	ENSG00000157020	SEC13	SEC13 homolog, nuclear pore and COPII coat complex component [Source:HGNC Symbol;Acc:HGNC:10697]	0.52
BCLTP7-110	4	ENSG00000178999	AURKB	aurora kinase B [Source:HGNC Symbol;Acc:HGNC:11390]	0.84
BCLTP7-110	4	ENSG00000211973	IGHV1-69	immunoglobulin heavy variable 1-69 [Source:HGNC Symbol;Acc:HGNC:5558]	1.16

Cluster ID	Cluster Size	Gene ID	Gene Name	Gene Description	Log_2 FC TIV
BCLTP7-110	4	ENSG00000211651	IGLV1-44	immunoglobulin lambda variable 1-44 [Source:HGNC Symbol;Acc:HGNC:5879]	1.48
BCLTP7-110	4	ENSG00000065328	MCM10	minichromosome maintenance 10 replication initiation factor [Source:HGNC Symbol;Acc:HGNC:18043]	0.98
BCLTP7-111	3	ENSG00000198018	ENTPD7	ectonucleoside triphosphate diphosphohydrolase 7 [Source:HGNC Symbol;Acc:HGNC:19745]	0.59
BCLTP7-111	3	ENSG00000177301	KCNA2	potassium voltage-gated channel subfamily A member 2 [Source:HGNC Symbol;Acc:HGNC:6220]	0.65
BCLTP7-111	3	ENSG00000065911	MTHFD2	methylenetetrahydrofolate dehydrogenase (NADP+ dependent) 2, methenyltetrahydrofolate cyclohydrolase [Source:HGNC Symbol;Acc:HGNC:7434]	0.60
BCLTP7-112	3	ENSG00000139734	DIAPH3	diaphanous related formin 3 [Source:HGNC Symbol;Acc:HGNC:15480]	1.48
BCLTP7-112	3	ENSG00000133328	HRASLS2	HRAS like suppressor 2 [Source:HGNC Symbol;Acc:HGNC:17824]	0.83
BCLTP7-112	3	ENSG00000163808	KIF15	kinesin family member 15 [Source:HGNC Symbol;Acc:HGNC:17273]	0.55
BCLTP7-113	5	ENSG00000150967	ABCB9	ATP binding cassette subfamily B member 9 [Source:HGNC Symbol;Acc:HGNC:50]	0.73
BCLTP7-113	5	ENSG00000117411	B4GALT2	beta-1,4-galactosyltransferase 2 [Source:HGNC Symbol;Acc:HGNC:925]	0.63
BCLTP7-113	5	ENSG00000010310	GIPR	gastric inhibitory polypeptide receptor [Source:HGNC Symbol;Acc:HGNC:4271]	0.82
BCLTP7-113	5	ENSG00000167476	JSRP1	junctional sarcoplasmic reticulum protein 1 [Source:HGNC Symbol;Acc:HGNC:24963]	1.03
BCLTP7-113	5	ENSG00000198879	SFMBT2	Scm-like with four mbt domains 2 [Source:HGNC Symbol;Acc:HGNC:20256]	0.34
BCLTP7-114	8	ENSG00000230006	ANKRD36BP2	ankyrin repeat domain 36B pseudogene 2 [Source:HGNC Symbol;Acc:HGNC:33607]	0.55
BCLTP7-114	8	ENSG00000184661	CDCA2	cell division cycle associated 2 [Source:HGNC Symbol;Acc:HGNC:14623]	1.38
BCLTP7-114	8	ENSG00000072571	HMMR	hyaluronan mediated motility receptor [Source:HGNC Symbol;Acc:HGNC:5012]	0.83
BCLTP7-114	8	ENSG00000138160	KIF11	kinesin family member 11 [Source:HGNC Symbol;Acc:HGNC:6388]	0.52
BCLTP7-114	8	ENSG00000112984	KIF20A	kinesin family member 20A [Source:HGNC Symbol;Acc:HGNC:9787]	0.76
BCLTP7-114	8	ENSG00000132646	PCNA	proliferating cell nuclear antigen [Source:HGNC Symbol;Acc:HGNC:8729]	0.35
BCLTP7-114	8	ENSG00000088325	TPX2	TPX2, microtubule nucleation factor [Source:HGNC Symbol;Acc:HGNC:1249]	0.70
BCLTP7-114	8	ENSG00000175063	UBE2C	ubiquitin conjugating enzyme E2 C [Source:HGNC Symbol;Acc:HGNC:15937]	0.98
BCLTP7-115	4	ENSG00000182985	CADM1	cell adhesion molecule 1 [Source:HGNC Symbol;Acc:HGNC:5951]	0.62
BCLTP7-115	4	ENSG00000145354	CISD2	CDGSH iron sulfur domain 2 [Source:HGNC Symbol;Acc:HGNC:24212]	0.47
BCLTP7-115	4	ENSG00000213047	DENND1B	DENN domain containing 1B [Source:HGNC Symbol;Acc:HGNC:28404]	0.56
BCLTP7-115	4	ENSG00000172965	MIR4435-2HG	MIR4435-2 host gene [Source:HGNC Symbol;Acc:HGNC:35163]	0.92
BCLTP7-116	4	ENSG00000249096			0.60
BCLTP7-116	4	ENSG00000179750	APOBEC3B	apolipoprotein B mRNA editing enzyme catalytic subunit 3B [Source:HGNC Symbol;Acc:HGNC:17352]	0.81
BCLTP7-116	4	ENSG00000123080	CDKN2C	cyclin dependent kinase inhibitor 2C [Source:HGNC Symbol;Acc:HGNC:1789]	0.48
BCLTP7-116	4	ENSG00000211685	IGLC7	immunoglobulin lambda constant 7 [Source:HGNC Symbol;Acc:HGNC:5861]	0.43
BCLTP7-117	9	ENSG00000143942	CHAC2	ChaC cation transport regulator homolog 2 [Source:HGNC Symbol;Acc:HGNC:32363]	0.62
BCLTP7-117	9	ENSG00000148468	FAM171A1	family with sequence similarity 171 member A1 [Source:HGNC Symbol;Acc:HGNC:23522]	2.23

Cluster ID	Cluster Size	Gene ID	Gene Name	Gene Description	Log_2 FC TIV
BCLTP7-117	9	ENSG00000174132	FAM174A	family with sequence similarity 174 member A [Source:HGNC Symbol;Acc:HGNC:24943]	0.92
BCLTP7-117	9	ENSG00000088340	FER1L4	fer-1 like family member 4, pseudogene [Source:HGNC Symbol;Acc:HGNC:15801]	2.09
BCLTP7-117	9	ENSG00000164032	H2AFZ	H2A histone family member Z [Source:HGNC Symbol;Acc:HGNC:4741]	0.42
BCLTP7-117	9	ENSG00000224373	IGHV4-59	immunoglobulin heavy variable 4-59 [Source:HGNC Symbol;Acc:HGNC:5654]	1.26
BCLTP7-117	9	ENSG00000115884	SDC1	syndecan 1 [Source:HGNC Symbol;Acc:HGNC:10658]	1.87
BCLTP7-117	9	ENSG00000227203	SUB1P1	SUB1 homolog, transcriptional regulator pseudogene 1 [Source:HGNC Symbol;Acc:HGNC:32300]	0.75
BCLTP7-117	9	ENSG00000167900	TK1	thymidine kinase 1 [Source:HGNC Symbol;Acc:HGNC:11830]	0.63
BCLTP7-118	5	ENSG00000156970	BUB1B	BUB1 mitotic checkpoint serine/threonine kinase B [Source:HGNC Symbol;Acc:HGNC:1149]	0.56
BCLTP7-118	5	ENSG00000255733	IFNG-AS1	IFNG antisense RNA 1 [Source:HGNC Symbol;Acc:HGNC:43910]	0.47
BCLTP7-118	5	ENSG00000239975	IGKV1D-33	immunoglobulin kappa variable 1D-33 [Source:HGNC Symbol;Acc:HGNC:5753]	1.25
BCLTP7-118	5	ENSG00000189233	NUGGC	nuclear GTPase, germinal center associated [Source:HGNC Symbol;Acc:HGNC:33550]	0.76
BCLTP7-118	5	ENSG00000165409	TSHR	thyroid stimulating hormone receptor [Source:HGNC Symbol;Acc:HGNC:12373]	1.25
BCLTP7-119	2	ENSG00000260948			-0.70
BCLTP7-119	2	ENSG00000204634	TBC1D8	TBC1 domain family member 8 [Source:HGNC Symbol;Acc:HGNC:17791]	-0.83
BCLTP7-120	3	ENSG00000186810	CXCR3	C-X-C motif chemokine receptor 3 [Source:HGNC Symbol;Acc:HGNC:4540]	1.09
BCLTP7-120	3	ENSG00000235587	GAPDHP65	glyceraldehyde 3 phosphate dehydrogenase pseudogene 65 [Source:HGNC Symbol;Acc:HGNC:4143]	0.51
BCLTP7-120	3	ENSG00000198722	UNC13B	unc-13 homolog B [Source:HGNC Symbol;Acc:HGNC:12566]	1.15
BCLTP7-121	7	ENSG00000146670	CDCA5	cell division cycle associated 5 [Source:HGNC Symbol;Acc:HGNC:14626]	1.01
BCLTP7-121	7	ENSG00000100526	CDKN3	cyclin dependent kinase inhibitor 3 [Source:HGNC Symbol;Acc:HGNC:1791]	1.13
BCLTP7-121	7	ENSG00000115163	CENPA	centromere protein A [Source:HGNC Symbol;Acc:HGNC:1851]	1.41
BCLTP7-121	7	ENSG00000169607	CKAP2L	cytoskeleton associated protein 2 like [Source:HGNC Symbol;Acc:HGNC:26877]	0.87
BCLTP7-121	7	ENSG00000137807	KIF23	kinesin family member 23 [Source:HGNC Symbol;Acc:HGNC:6392]	1.09
BCLTP7-121	7	ENSG00000080986	NDC80	NDC80, kinetochore complex component [Source:HGNC Symbol;Acc:HGNC:16909]	0.65
BCLTP7-121	7	ENSG00000117650	NEK2	NIMA related kinase 2 [Source:HGNC Symbol;Acc:HGNC:7745]	0.57
BCLTP7-122	6	ENSG00000170345	FOS	Fos proto-oncogene, AP-1 transcription factor subunit [Source:HGNC Symbol;Acc:HGNC:3796]	0.69
BCLTP7-122	6	ENSG00000244575	IGKV1-27	immunoglobulin kappa variable 1-27 [Source:HGNC Symbol;Acc:HGNC:5735]	0.50
BCLTP7-122	6	ENSG00000244116	IGKV2-28	immunoglobulin kappa variable 2-28 [Source:HGNC Symbol;Acc:HGNC:5783]	0.64
BCLTP7-122	6	ENSG00000242534	IGKV2D-28	immunoglobulin kappa variable 2D-28 [Source:HGNC Symbol;Acc:HGNC:5799]	0.37
BCLTP7-122	6	ENSG00000253691	IGKV2OR22-4	immunoglobulin kappa variable 2/OR22-4 (pseudogene) [Source:HGNC Symbol;Acc:HGNC:5813]	0.83
BCLTP7-122	6	ENSG00000185480	PARPBP	PARP1 binding protein [Source:HGNC Symbol;Acc:HGNC:26074]	0.24
BCLTP7-123	2	ENSG00000211945	IGHV1-18	immunoglobulin heavy variable 1-18 [Source:HGNC Symbol;Acc:HGNC:5549]	0.39
BCLTP7-123	2	ENSG00000253818	IGLV1-41	immunoglobulin lambda variable 1-41 (pseudogene) [Source:HGNC Symbol;Acc:HGNC:5878]	0.71
BCLTP7-124	3	ENSG00000101439	CST3	cystatin C [Source:HGNC Symbol;Acc:HGNC:2475]	-1.07
BCLTP7-124	3	ENSG00000160883	HK3	hexokinase 3 [Source:HGNC Symbol;Acc:HGNC:4925]	-0.66

Cluster ID	Cluster Size	Gene ID	Gene Name	Gene Description	Log_2 FC TIV
BCLTP7-124	3	ENSG00000196263	ZNF471	zinc finger protein 471 [Source:HGNC Symbol;Acc:HGNC:23226]	-0.56
BCLTP7-125	4	ENSG00000227155			-0.38
BCLTP7-125	4	ENSG00000278897			-0.62
BCLTP7-125	4	ENSG00000279192	PWAR5	Prader Willi/Angelman region RNA 5 [Source:HGNC Symbol;Acc:HGNC:30090]	-0.64
BCLTP7-125	4	ENSG00000197714	ZNF460	zinc finger protein 460 [Source:HGNC Symbol;Acc:HGNC:21628]	-0.70
BCLTP7-126	5	ENSG00000187837	HIST1H1C	histone cluster 1 H1 family member c [Source:HGNC Symbol;Acc:HGNC:4716]	0.61
BCLTP7-126	5	ENSG00000211967	IGHV3-53	immunoglobulin heavy variable 3-53 [Source:HGNC Symbol;Acc:HGNC:5610]	0.84
BCLTP7-126	5	ENSG00000211933	IGHV6-1	immunoglobulin heavy variable 6-1 [Source:HGNC Symbol;Acc:HGNC:5662]	0.61
BCLTP7-126	5	ENSG00000211673	IGLV3-1	immunoglobulin lambda variable 3-1 [Source:HGNC Symbol;Acc:HGNC:5896]	1.30
BCLTP7-126	5	ENSG00000004799	PDK4	pyruvate dehydrogenase kinase 4 [Source:HGNC Symbol;Acc:HGNC:8812]	0.47
BCLTP7-127	8	ENSG00000106080	FKBP14	FK506 binding protein 14 [Source:HGNC Symbol;Acc:HGNC:18625]	0.39
BCLTP7-127	8	ENSG00000185745	IFIT1	interferon induced protein with tetratricopeptide repeats 1 [Source:HGNC Symbol;Acc:HGNC:5407]	0.60
BCLTP7-127	8	ENSG00000270472	IGHV3OR16-9	immunoglobulin heavy variable 3/OR16-9 (non-functional) [Source:HGNC Symbol;Acc:HGNC:5644]	1.30
BCLTP7-127	8	ENSG00000211648	IGLV1-47	immunoglobulin lambda variable 1-47 [Source:HGNC Symbol;Acc:HGNC:5880]	1.17
BCLTP7-127	8	ENSG00000253822	IGLV3-24	immunoglobulin lambda variable 3-24 (pseudogene) [Source:HGNC Symbol;Acc:HGNC:5907]	1.42
BCLTP7-127	8	ENSG00000115415	STAT1	signal transducer and activator of transcription 1 [Source:HGNC Symbol;Acc:HGNC:11362]	0.39
BCLTP7-127	8	ENSG00000102595	UGGT2	UDP-glucose glycoprotein glucosyltransferase 2 [Source:HGNC Symbol;Acc:HGNC:15664]	0.45
BCLTP7-127	8	ENSG00000170027	YWHAG	tyrosine 3-monooxygenase/tryptophan 5-monooxygenase activation protein gamma [Source:HGNC Symbol;Acc:HGNC:12852]	0.46
BCLTP7-128	3	ENSG00000166825	ANPEP	alanyl aminopeptidase, membrane [Source:HGNC Symbol;Acc:HGNC:500]	-0.64
BCLTP7-128	3	ENSG00000147459	DOCK5	dedicator of cytokinesis 5 [Source:HGNC Symbol;Acc:HGNC:23476]	-0.93
BCLTP7-128	3	ENSG00000120708	TGFBI	transforming growth factor beta induced [Source:HGNC Symbol;Acc:HGNC:11771]	-0.74
BCLTP7-129	7	ENSG00000241666			-0.59
BCLTP7-129	7	ENSG00000274272			-0.35
BCLTP7-129	7	ENSG00000141753	IGFBP4	insulin like growth factor binding protein 4 [Source:HGNC Symbol;Acc:HGNC:5473]	-0.27
BCLTP7-129	7	ENSG00000224699	LAMTOR5-AS1	LAMTOR5 antisense RNA 1 [Source:HGNC Symbol;Acc:HGNC:40823]	-0.67
BCLTP7-129	7	ENSG00000187922	LCN10	lipocalin 10 [Source:HGNC Symbol;Acc:HGNC:20892]	-0.38
BCLTP7-129	7	ENSG00000220008	LINGO3	leucine rich repeat and Ig domain containing 3 [Source:HGNC Symbol;Acc:HGNC:21206]	-0.33
BCLTP7-129	7	ENSG00000185522	LMNTD2	lamin tail domain containing 2 [Source:HGNC Symbol;Acc:HGNC:28561]	-0.34
BCLTP7-130	6	ENSG00000274422			-0.76
BCLTP7-130	6	ENSG00000279759			-0.44
BCLTP7-130	6	ENSG00000279873	LINC01126	long intergenic non-protein coding RNA 1126 [Source:HGNC Symbol;Acc:HGNC:49275]	-0.35
BCLTP7-130	6	ENSG00000233806	LINC01237	long intergenic non-protein coding RNA 1237 [Source:HGNC Symbol;Acc:HGNC:49793]	-0.60
BCLTP7-130	6	ENSG00000180747	SMG1P3	SMG1P3, nonsense mediated mRNA decay associated PI3K related kinase pseudogene 3 [Source:HGNC Symbol;Acc:HGNC:49860]	-0.59

Cluster ID	Cluster Size	Gene ID	Gene Name	Gene Description	Log_2 FC TIV
BCLTP7-130	6	ENSG00000006638	TBXA2R	thromboxane A2 receptor [Source:HGNC Symbol;Acc:HGNC:11608]	-0.30

Table 33: Co-expressed gene clusters (B Cells, Day 7)

Cluster ID	Cluster Size	Gene ID	Gene Name	Gene Description	Log_2 FC TIV
BCLTP8-001	2	ENSG00000112473	SLC39A7	solute carrier family 39 member 7 [Source:HGNC Symbol;Acc:HGNC:4927]	0.43
BCLTP8-001	2	ENSG00000239264	TXNDC5	thioredoxin domain containing 5 [Source:HGNC Symbol;Acc:HGNC:21073]	0.67
BCLTP8-002	2	ENSG00000150961	SEC24D	SEC24 homolog D, COPII coat complex component [Source:HGNC Symbol;Acc:HGNC:10706]	0.64
BCLTP8-002	2	ENSG00000026751	SLAMF7	SLAM family member 7 [Source:HGNC Symbol;Acc:HGNC:21394]	0.89
BCLTP8-003	2	ENSG00000134285	FKBP11	FK506 binding protein 11 [Source:HGNC Symbol;Acc:HGNC:18624]	0.73
BCLTP8-003	2	ENSG00000143603	KCNN3	potassium calcium-activated channel subfamily N member 3 [Source:HGNC Symbol;Acc:HGNC:6292]	0.86
BCLTP8-004	2	ENSG00000168268	NT5DC2	5'-nucleotidase domain containing 2 [Source:HGNC Symbol;Acc:HGNC:25717]	0.74
BCLTP8-004	2	ENSG00000071537	SEL1L	SEL1L ERAD E3 ligase adaptor subunit [Source:HGNC Symbol;Acc:HGNC:10717]	0.48
BCLTP8-005	2	ENSG00000119912	IDE	insulin degrading enzyme [Source:HGNC Symbol;Acc:HGNC:5381]	0.42
BCLTP8-005	2	ENSG00000211653	IGLV1-40	immunoglobulin lambda variable 1-40 [Source:HGNC Symbol;Acc:HGNC:5877]	1.74
BCLTP8-006	3	ENSG00000163902	RPN1	ribophorin I [Source:HGNC Symbol;Acc:HGNC:10381]	0.53
BCLTP8-006	3	ENSG00000118705	RPN2	ribophorin II [Source:HGNC Symbol;Acc:HGNC:10382]	0.63
BCLTP8-006	3	ENSG00000124783	SSR1	signal sequence receptor subunit 1 [Source:HGNC Symbol;Acc:HGNC:11323]	0.41
BCLTP8-007	2	ENSG00000132465	JCHAIN	joining chain of multimeric IgA and IgM [Source:HGNC Symbol;Acc:HGNC:5713]	0.84
BCLTP8-007	2	ENSG00000048462	TNFRSF17	TNF receptor superfamily member 17 [Source:HGNC Symbol;Acc:HGNC:11913]	0.75
BCLTP8-008	2	ENSG00000106105	GARS	glycyl-tRNA synthetase [Source:HGNC Symbol;Acc:HGNC:4162]	0.37
BCLTP8-008	2	ENSG00000170476	MZB1	marginal zone B and B1 cell specific protein [Source:HGNC Symbol;Acc:HGNC:30125]	0.77
BCLTP8-009	2	ENSG00000068912	ERLEC1	endoplasmic reticulum lectin 1 [Source:HGNC Symbol;Acc:HGNC:25222]	0.72
BCLTP8-009	2	ENSG00000112893	MAN2A1	mannosidase alpha class 2A member 1 [Source:HGNC Symbol;Acc:HGNC:6824]	0.33
BCLTP8-010	2	ENSG00000145050	MANF	mesencephalic astrocyte derived neurotrophic factor [Source:HGNC Symbol;Acc:HGNC:15461]	0.74
BCLTP8-010	2	ENSG00000113387	SUB1	SUB1 homolog, transcriptional regulator [Source:HGNC Symbol;Acc:HGNC:19985]	0.43
BCLTP8-011	2	ENSG00000211662	IGLV3-21	immunoglobulin lambda variable 3-21 [Source:HGNC Symbol;Acc:HGNC:5905]	2.05
BCLTP8-011	2	ENSG00000163527	STT3B	STT3B, catalytic subunit of the oligosaccharyltransferase complex [Source:HGNC Symbol;Acc:HGNC:30611]	0.34
BCLTP8-012	3	ENSG00000244038	DDOST	dolichyl-diphosphooligosaccharide–protein glycosyltransferase non-catalytic subunit [Source:HGNC Symbol;Acc:HGNC:2728]	0.53
BCLTP8-012	3	ENSG00000102580	DNAJC3	DnaJ heat shock protein family (Hsp40) member C3 [Source:HGNC Symbol;Acc:HGNC:9439]	0.51
BCLTP8-012	3	ENSG00000167861	HID1	HID1 domain containing [Source:HGNC Symbol;Acc:HGNC:15736]	0.88
BCLTP8-013	2	ENSG00000074695	LMAN1	lectin, mannose binding 1 [Source:HGNC Symbol;Acc:HGNC:6631]	0.69

Cluster ID	Cluster Size	Gene ID	Gene Name	Gene Description	Log_2 FC TIV
BCLTP8-013	2	ENSG00000147649	MTDH	metadherin [Source:HGNC Symbol;Acc:HGNC:29608]	0.30
BCLTP8-014	5	ENSG00000123989	CHPF	chondroitin polymerizing factor [Source:HGNC Symbol;Acc:HGNC:24291]	1.13
BCLTP8-014	5	ENSG00000135916	ITM2C	integral membrane protein 2C [Source:HGNC Symbol;Acc:HGNC:6175]	0.83
BCLTP8-014	5	ENSG00000074842	MYDGF	myeloid derived growth factor [Source:HGNC Symbol;Acc:HGNC:16948]	0.94
BCLTP8-014	5	ENSG00000155660	PDIA4	protein disulfide isomerase family A member 4 [Source:HGNC Symbol;Acc:HGNC:30167]	0.82
BCLTP8-014	5	ENSG00000118363	SPCS2	signal peptidase complex subunit 2 [Source:HGNC Symbol;Acc:HGNC:28962]	0.29
BCLTP8-015	2	ENSG00000120697	ALG5	ALG5, dolichyl-phosphate beta-glucosyltransferase [Source:HGNC Symbol;Acc:HGNC:20266]	0.38
BCLTP8-015	2	ENSG00000074416	MGLL	monoglyceride lipase [Source:HGNC Symbol;Acc:HGNC:17038]	0.60
BCLTP8-016	2	ENSG00000069849	ATP1B3	ATPase Na+/K+ transporting subunit beta 3 [Source:HGNC Symbol;Acc:HGNC:806]	0.33
BCLTP8-016	2	ENSG00000105974	CAV1	caveolin 1 [Source:HGNC Symbol;Acc:HGNC:1527]	1.49
BCLTP8-017	2	ENSG00000104635	SLC39A14	solute carrier family 39 member 14 [Source:HGNC Symbol;Acc:HGNC:20858]	0.37
BCLTP8-017	2	ENSG00000123352	SPATS2	spermatogenesis associated serine rich 2 [Source:HGNC Symbol;Acc:HGNC:18650]	0.60
BCLTP8-018	3	ENSG00000044574	HSPA5	heat shock protein family A (Hsp70) member 5 [Source:HGNC Symbol;Acc:HGNC:5238]	0.68
BCLTP8-018	3	ENSG00000166794	PPIB	peptidylprolyl isomerase B [Source:HGNC Symbol;Acc:HGNC:9255]	0.67
BCLTP8-018	3	ENSG00000153066	TXNDC11	thioredoxin domain containing 11 [Source:HGNC Symbol;Acc:HGNC:28030]	0.61
BCLTP8-019	2	ENSG00000139193	CD27	CD27 molecule [Source:HGNC Symbol;Acc:HGNC:11922]	0.64
BCLTP8-019	2	ENSG00000121073	SLC35B1	solute carrier family 35 member B1 [Source:HGNC Symbol;Acc:HGNC:20798]	0.48
BCLTP8-020	3	ENSG00000100342	APOL1	apolipoprotein L1 [Source:HGNC Symbol;Acc:HGNC:618]	0.58
BCLTP8-020	3	ENSG00000136026	CKAP4	cytoskeleton associated protein 4 [Source:HGNC Symbol;Acc:HGNC:16991]	0.68
BCLTP8-020	3	ENSG00000198900	TOP1	topoisomerase (DNA) I [Source:HGNC Symbol;Acc:HGNC:11986]	0.34
BCLTP8-021	2	ENSG00000143942	CHAC2	ChaC cation transport regulator homolog 2 [Source:HGNC Symbol;Acc:HGNC:32363]	0.64
BCLTP8-021	2	ENSG00000083444	PLOD1	procollagen-lysine,2-oxoglutarate 5-dioxygenase 1 [Source:HGNC Symbol;Acc:HGNC:9081]	0.59
BCLTP8-022	2	ENSG00000164032	H2AFZ	H2A histone family member Z [Source:HGNC Symbol;Acc:HGNC:4741]	0.26
BCLTP8-022	2	ENSG00000115884	SDC1	syndecan 1 [Source:HGNC Symbol;Acc:HGNC:10658]	1.62
BCLTP8-023	4	ENSG00000155304	HSPA13	heat shock protein family A (Hsp70) member 13 [Source:HGNC Symbol;Acc:HGNC:11375]	0.62
BCLTP8-023	4	ENSG00000113615	SEC24A	SEC24 homolog A, COPII coat complex component [Source:HGNC Symbol;Acc:HGNC:10703]	0.66
BCLTP8-023	4	ENSG00000058262	SEC61A1	Sec61 translocon alpha 1 subunit [Source:HGNC Symbol;Acc:HGNC:18276]	0.47
BCLTP8-023	4	ENSG00000182934	SRPRA	SRP receptor alpha subunit [Source:HGNC Symbol;Acc:HGNC:11307]	0.39
BCLTP8-024	8	ENSG00000183508	FAM46C	family with sequence similarity 46 member C [Source:HGNC Symbol;Acc:HGNC:24712]	0.66
BCLTP8-024	8	ENSG00000106415	GLCCI1	glucocorticoid induced 1 [Source:HGNC Symbol;Acc:HGNC:18713]	0.58
BCLTP8-024	8	ENSG00000070081	NUCB2	nucleobindin 2 [Source:HGNC Symbol;Acc:HGNC:8044]	0.80
BCLTP8-024	8	ENSG00000198856	OSTC	oligosaccharyltransferase complex non-catalytic subunit [Source:HGNC Symbol;Acc:HGNC:24448]	0.50
BCLTP8-024	8	ENSG00000102096	PIM2	Pim-2 proto-oncogene, serine/threonine kinase [Source:HGNC Symbol;Acc:HGNC:8987]	0.52
BCLTP8-024	8	ENSG00000123131	PRDX4	peroxiredoxin 4 [Source:HGNC Symbol;Acc:HGNC:17169]	0.89

Cluster ID	Cluster Size	Gene ID	Gene Name	Gene Description	Log_2 FC TIV
BCLTP8-024	8	ENSG00000070214	SLC44A1	solute carrier family 44 member 1 [Source:HGNC Symbol;Acc:HGNC:18798]	0.61
BCLTP8-024	8	ENSG00000129128	SPCS3	signal peptidase complex subunit 3 [Source:HGNC Symbol;Acc:HGNC:26212]	0.38
BCLTP8-025	2	ENSG00000049860	HEXB	hexosaminidase subunit beta [Source:HGNC Symbol;Acc:HGNC:4879]	0.35
BCLTP8-025	2	ENSG00000079931	MOXD1	monooxygenase DBH like 1 [Source:HGNC Symbol;Acc:HGNC:21063]	0.70
BCLTP8-026	4	ENSG00000118985	ELL2	elongation factor for RNA polymerase II 2 [Source:HGNC Symbol;Acc:HGNC:17064]	0.81
BCLTP8-026	4	ENSG00000138709	LARP1B	La ribonucleoprotein domain family member 1B [Source:HGNC Symbol;Acc:HGNC:24704]	0.42
BCLTP8-026	4	ENSG00000163694	RBM47	RNA binding motif protein 47 [Source:HGNC Symbol;Acc:HGNC:30358]	0.51
BCLTP8-026	4	ENSG00000065308	TRAM2	translocation associated membrane protein 2 [Source:HGNC Symbol;Acc:HGNC:16855]	0.61
BCLTP8-027	5	ENSG00000153093	ACOXL	acyl-CoA oxidase-like [Source:HGNC Symbol;Acc:HGNC:25621]	1.39
BCLTP8-027	5	ENSG00000198937	CCDC167	coiled-coil domain containing 167 [Source:HGNC Symbol;Acc:HGNC:21239]	0.56
BCLTP8-027	5	ENSG00000049656	CLPTM1L	CLPTM1 like [Source:HGNC Symbol;Acc:HGNC:24308]	0.39
BCLTP8-027	5	ENSG00000173848	NET1	neuroepithelial cell transforming 1 [Source:HGNC Symbol;Acc:HGNC:14592]	0.69
BCLTP8-027	5	ENSG00000134910	STT3A	STT3A, catalytic subunit of the oligosaccharyltransferase complex [Source:HGNC Symbol;Acc:HGNC:6172]	0.59
BCLTP8-028	2	ENSG00000211964	IGHV3-48	immunoglobulin heavy variable 3-48 [Source:HGNC Symbol;Acc:HGNC:5606]	0.77
BCLTP8-028	2	ENSG00000198833	UBE2J1	ubiquitin conjugating enzyme E2 J1 [Source:HGNC Symbol;Acc:HGNC:17598]	0.40
BCLTP8-029	3	ENSG00000134153	EMC7	ER membrane protein complex subunit 7 [Source:HGNC Symbol;Acc:HGNC:24301]	0.37
BCLTP8-029	3	ENSG00000148468	FAM171A1	family with sequence similarity 171 member A1 [Source:HGNC Symbol;Acc:HGNC:23522]	1.79
BCLTP8-029	3	ENSG00000134825	TMEM258	transmembrane protein 258 [Source:HGNC Symbol;Acc:HGNC:1164]	0.44
BCLTP8-030	3	ENSG00000242766	IGKV1D-17	immunoglobulin kappa variable 1D-17 [Source:HGNC Symbol;Acc:HGNC:5749]	0.86
BCLTP8-030	3	ENSG00000095380	NANS	N-acetylneuraminate synthase [Source:HGNC Symbol;Acc:HGNC:19237]	0.47
BCLTP8-030	3	ENSG00000173334	TRIB1	tribbles pseudokinase 1 [Source:HGNC Symbol;Acc:HGNC:16891]	0.73
BCLTP8-031	3	ENSG00000112237	CCNC	cyclin C [Source:HGNC Symbol;Acc:HGNC:1581]	0.30
BCLTP8-031	3	ENSG00000211938	IGHV3-7	immunoglobulin heavy variable 3-7 [Source:HGNC Symbol;Acc:HGNC:5620]	1.33
BCLTP8-031	3	ENSG00000136840	ST6GALNAC4	ST6 N-acetylgalactosaminide alpha-2,6-sialyltransferase 4 [Source:HGNC Symbol;Acc:HGNC:17846]	0.55
BCLTP8-032	3	ENSG00000124788	ATXN1	ataxin 1 [Source:HGNC Symbol;Acc:HGNC:10548]	0.57
BCLTP8-032	3	ENSG00000185164	NOMO2	NODAL modulator 2 [Source:HGNC Symbol;Acc:HGNC:22652]	0.79
BCLTP8-032	3	ENSG00000115902	SLC1A4	solute carrier family 1 member 4 [Source:HGNC Symbol;Acc:HGNC:10942]	0.55
BCLTP8-033	3	ENSG00000103226	NOMO3	NODAL modulator 3 [Source:HGNC Symbol;Acc:HGNC:25242]	0.50
BCLTP8-033	3	ENSG00000113811	SELENOK	selenoprotein K [Source:HGNC Symbol;Acc:HGNC:30394]	0.44
BCLTP8-033	3	ENSG00000170348	TMED10	transmembrane p24 trafficking protein 10 [Source:HGNC Symbol;Acc:HGNC:16998]	0.38
BCLTP8-034	5	ENSG00000004468	CD38	CD38 molecule [Source:HGNC Symbol;Acc:HGNC:1667]	0.78
BCLTP8-034	5	ENSG00000129562	DAD1	defender against cell death 1 [Source:HGNC Symbol;Acc:HGNC:2664]	0.30
BCLTP8-034	5	ENSG00000259706	HSP90B2P	heat shock protein 90 beta family member 2, pseudogene [Source:HGNC Symbol;Acc:HGNC:12099]	0.90
BCLTP8-034	5	ENSG00000204386	NEU1	neuraminidase 1 [Source:HGNC Symbol;Acc:HGNC:7758]	0.55

Cluster ID	Cluster Size	Gene ID	Gene Name	Gene Description	Log_2 FC TIV
BCLTP8-034	5	ENSG00000131871	SELENOS	selenoprotein S [Source:HGNC Symbol;Acc:HGNC:30396]	0.73
BCLTP8-035	3	ENSG00000108578	BLMH	bleomycin hydrolase [Source:HGNC Symbol;Acc:HGNC:1059]	0.34
BCLTP8-035	3	ENSG00000106803	SEC61B	Sec61 translocon beta subunit [Source:HGNC Symbol;Acc:HGNC:16993]	0.59
BCLTP8-035	3	ENSG00000163053	SLC16A14	solute carrier family 16 member 14 [Source:HGNC Symbol;Acc:HGNC:26417]	1.14
BCLTP8-036	3	ENSG00000110917	MLEC	malectin [Source:HGNC Symbol;Acc:HGNC:28973]	0.38
BCLTP8-036	3	ENSG00000065911	MTHFD2	methylenetetrahydrofolate dehydrogenase (NADP+ dependent) 2, methenyltetrahydrofolate cyclohydrolase [Source:HGNC Symbol;Acc:HGNC:7434]	0.48
BCLTP8-036	3	ENSG00000103257	SLC7A5	solute carrier family 7 member 5 [Source:HGNC Symbol;Acc:HGNC:11063]	0.76
BCLTP8-037	4	ENSG00000168374	ARF4	ADP ribosylation factor 4 [Source:HGNC Symbol;Acc:HGNC:655]	0.43
BCLTP8-037	4	ENSG00000086062	B4GALT1	beta-1,4-galactosyltransferase 1 [Source:HGNC Symbol;Acc:HGNC:924]	0.40
BCLTP8-037	4	ENSG00000120725	SIL1	SIL1 nucleotide exchange factor [Source:HGNC Symbol;Acc:HGNC:24624]	0.59
BCLTP8-037	4	ENSG00000184840	TMED9	transmembrane p24 trafficking protein 9 [Source:HGNC Symbol;Acc:HGNC:24878]	0.45
BCLTP8-038	5	ENSG00000010310	GIPR	gastric inhibitory polypeptide receptor [Source:HGNC Symbol;Acc:HGNC:4271]	0.54
BCLTP8-038	5	ENSG00000111885	MAN1A1	mannosidase alpha class 1A member 1 [Source:HGNC Symbol;Acc:HGNC:6821]	0.70
BCLTP8-038	5	ENSG00000143870	PDIA6	protein disulfide isomerase family A member 6 [Source:HGNC Symbol;Acc:HGNC:30168]	0.53
BCLTP8-038	5	ENSG00000128228	SDF2L1	stromal cell derived factor 2 like 1 [Source:HGNC Symbol;Acc:HGNC:10676]	0.68
BCLTP8-038	5	ENSG00000108953	YWHAE	tyrosine 3-monooxygenase/tryptophan 5-monooxygenase activation protein epsilon [Source:HGNC Symbol;Acc:HGNC:12851]	0.32
BCLTP8-039	3	ENSG00000143641	GALNT2	polypeptide N-acetylgalactosaminyltransferase 2 [Source:HGNC Symbol;Acc:HGNC:4124]	0.38
BCLTP8-039	3	ENSG00000211677	IGLC2	immunoglobulin lambda constant 2 [Source:HGNC Symbol;Acc:HGNC:5856]	1.34
BCLTP8-039	3	ENSG00000002549	LAP3	leucine aminopeptidase 3 [Source:HGNC Symbol;Acc:HGNC:18449]	0.48
BCLTP8-040	5	ENSG00000180535	BHLHA15	basic helix-loop-helix family member a15 [Source:HGNC Symbol;Acc:HGNC:22265]	0.96
BCLTP8-040	5	ENSG00000087502	ERGIC2	ERGIC and golgi 2 [Source:HGNC Symbol;Acc:HGNC:30208]	0.48
BCLTP8-040	5	ENSG00000075420	FNDC3B	fibronectin type III domain containing 3B [Source:HGNC Symbol;Acc:HGNC:24670]	1.03
BCLTP8-040	5	ENSG00000108829	LRRC59	leucine rich repeat containing 59 [Source:HGNC Symbol;Acc:HGNC:28817]	0.61
BCLTP8-040	5	ENSG00000183010	PYCR1	pyrroline-5-carboxylate reductase 1 [Source:HGNC Symbol;Acc:HGNC:9721]	0.99
BCLTP8-041	2	ENSG00000171155	C1GALT1C1	C1GALT1 specific chaperone 1 [Source:HGNC Symbol;Acc:HGNC:24338]	0.55
BCLTP8-041	2	ENSG00000125844	RRBP1	ribosome binding protein 1 [Source:HGNC Symbol;Acc:HGNC:10448]	0.64
BCLTP8-042	5	ENSG00000154723	ATP5J	ATP synthase, H+ transporting, mitochondrial Fo complex subunit F6 [Source:HGNC Symbol;Acc:HGNC:847]	0.37
BCLTP8-042	5	ENSG00000203914	HSP90B3P	heat shock protein 90 beta family member 3, pseudogene [Source:HGNC Symbol;Acc:HGNC:12100]	0.94
BCLTP8-042	5	ENSG00000211896	IGHG1	immunoglobulin heavy constant gamma 1 (G1m marker) [Source:HGNC Symbol;Acc:HGNC:5525]	1.68
BCLTP8-042	5	ENSG00000102471	NDFIP2	Nedd4 family interacting protein 2 [Source:HGNC Symbol;Acc:HGNC:18537]	0.86
BCLTP8-042	5	ENSG00000100219	XBP1	X-box binding protein 1 [Source:HGNC Symbol;Acc:HGNC:12801]	1.02

Cluster ID	Cluster Size	Gene ID	Gene Name	Gene Description	Log_2 FC TIV
BCLTP8-043	2	ENSG00000241351	IGKV3-11	immunoglobulin kappa variable 3-11 [Source:HGNC Symbol;Acc:HGNC:5815]	0.68
BCLTP8-043	2	ENSG00000114902	SPCS1	signal peptidase complex subunit 1 [Source:HGNC Symbol;Acc:HGNC:23401]	0.51
BCLTP8-044	2	ENSG00000153162	BMP6	bone morphogenetic protein 6 [Source:HGNC Symbol;Acc:HGNC:1073]	0.81
BCLTP8-044	2	ENSG00000211892	IGHG4	immunoglobulin heavy constant gamma 4 (G4m marker) [Source:HGNC Symbol;Acc:HGNC:5528]	0.68
BCLTP8-045	5	ENSG00000064763	FAR2	fatty acyl-CoA reductase 2 [Source:HGNC Symbol;Acc:HGNC:25531]	0.69
BCLTP8-045	5	ENSG00000198380	GFPT1	glutamine–fructose-6-phosphate transaminase 1 [Source:HGNC Symbol;Acc:HGNC:4241]	0.46
BCLTP8-045	5	ENSG00000211952	IGHV4-28	immunoglobulin heavy variable 4-28 [Source:HGNC Symbol;Acc:HGNC:5645]	0.92
BCLTP8-045	5	ENSG00000138768	USO1	USO1 vesicle transport factor [Source:HGNC Symbol;Acc:HGNC:30904]	0.33
BCLTP8-045	5	ENSG00000111424	VDR	vitamin D (1,25- dihydroxyvitamin D3) receptor [Source:HGNC Symbol;Acc:HGNC:12679]	0.67
BCLTP8-046	3	ENSG00000128590	DNAJB9	DnaJ heat shock protein family (Hsp40) member B9 [Source:HGNC Symbol;Acc:HGNC:6968]	0.69
BCLTP8-046	3	ENSG00000211933	IGHV6-1	immunoglobulin heavy variable 6-1 [Source:HGNC Symbol;Acc:HGNC:5662]	0.42
BCLTP8-046	3	ENSG00000069956	MAPK6	mitogen-activated protein kinase 6 [Source:HGNC Symbol;Acc:HGNC:6879]	0.36
BCLTP8-047	2	ENSG00000253755	IGHGP	immunoglobulin heavy constant gamma P (non-functional) [Source:HGNC Symbol;Acc:HGNC:5529]	1.51
BCLTP8-047	2	ENSG00000146733	PSPH	phosphoserine phosphatase [Source:HGNC Symbol;Acc:HGNC:9577]	0.59
BCLTP8-048	4	ENSG00000174132	FAM174A	family with sequence similarity 174 member A [Source:HGNC Symbol;Acc:HGNC:24943]	0.69
BCLTP8-048	4	ENSG00000088340	FER1L4	fer-1 like family member 4, pseudogene [Source:HGNC Symbol;Acc:HGNC:15801]	1.71
BCLTP8-048	4	ENSG00000224373	IGHV4-59	immunoglobulin heavy variable 4-59 [Source:HGNC Symbol;Acc:HGNC:5654]	1.23
BCLTP8-048	4	ENSG00000113140	SPARC	secreted protein acidic and cysteine rich [Source:HGNC Symbol;Acc:HGNC:11219]	1.84
BCLTP8-049	2	ENSG00000107104	KANK1	KN motif and ankyrin repeat domains 1 [Source:HGNC Symbol;Acc:HGNC:19309]	0.81
BCLTP8-049	2	ENSG00000264522	OTUD7B	OTU deubiquitinase 7B [Source:HGNC Symbol;Acc:HGNC:16683]	0.51
BCLTP8-050	2	ENSG00000233806	LINC01237	long intergenic non-protein coding RNA 1237 [Source:HGNC Symbol;Acc:HGNC:49793]	-0.31
BCLTP8-050	2	ENSG00000181722	ZBTB20	zinc finger and BTB domain containing 20 [Source:HGNC Symbol;Acc:HGNC:13503]	-0.59
BCLTP8-051	6	ENSG00000111291	GPRC5D	G protein-coupled receptor class C group 5 member D [Source:HGNC Symbol;Acc:HGNC:13310]	0.73
BCLTP8-051	6	ENSG00000099337	KCNK6	potassium two pore domain channel subfamily K member 6 [Source:HGNC Symbol;Acc:HGNC:6281]	0.58
BCLTP8-051	6	ENSG00000184232	OAF	out at first homolog [Source:HGNC Symbol;Acc:HGNC:28752]	0.93
BCLTP8-051	6	ENSG00000037241	RPL26L1	ribosomal protein L26 like 1 [Source:HGNC Symbol;Acc:HGNC:17050]	0.44
BCLTP8-051	6	ENSG00000100883	SRP54	signal recognition particle 54 [Source:HGNC Symbol;Acc:HGNC:11301]	0.46
BCLTP8-051	6	ENSG00000145817	YIPF5	Yip1 domain family member 5 [Source:HGNC Symbol;Acc:HGNC:24877]	0.33
BCLTP8-052	4	ENSG00000115163	CENPA	centromere protein A [Source:HGNC Symbol;Acc:HGNC:1851]	0.89
BCLTP8-052	4	ENSG00000254395	IGHV4-55	immunoglobulin heavy variable 4-55 (pseudogene) [Source:HGNC Symbol;Acc:HGNC:5653]	0.83
BCLTP8-052	4	ENSG00000211592	IGKC	immunoglobulin kappa constant [Source:HGNC Symbol;Acc:HGNC:5716]	0.31

Cluster ID	Cluster Size	Gene ID	Gene Name	Gene Description	Log_2 FC TIV
BCLTP8-052	4	ENSG00000117632	STMN1	stathmin 1 [Source:HGNC Symbol;Acc:HGNC:6510]	0.19
BCLTP8-053	3	ENSG00000282600			1.26
BCLTP8-053	3	ENSG00000119523	ALG2	ALG2, alpha-1,3/1,6-mannosyltransferase [Source:HGNC Symbol;Acc:HGNC:23159]	0.43
BCLTP8-053	3	ENSG00000186810	CXCR3	C-X-C motif chemokine receptor 3 [Source:HGNC Symbol;Acc:HGNC:4540]	1.14
BCLTP8-054	3	ENSG00000280411	IGHV1-69-2	immunoglobulin heavy variable 1-69-2 [Source:HGNC Symbol;Acc:HGNC:5562]	0.73
BCLTP8-054	3	ENSG00000211632	IGKV3D-11	immunoglobulin kappa variable 3D-11 [Source:HGNC Symbol;Acc:HGNC:5823]	0.53
BCLTP8-054	3	ENSG00000211645	IGLV1-50	immunoglobulin lambda variable 1-50 (non-functional) [Source:HGNC Symbol;Acc:HGNC:5881]	1.78
BCLTP8-055	5	ENSG00000138166	DUSP5	dual specificity phosphatase 5 [Source:HGNC Symbol;Acc:HGNC:3071]	0.82
BCLTP8-055	5	ENSG00000162676	GFI1	growth factor independent 1 transcriptional repressor [Source:HGNC Symbol;Acc:HGNC:4237]	1.25
BCLTP8-055	5	ENSG00000163754	GYG1	glycogenin 1 [Source:HGNC Symbol;Acc:HGNC:4699]	0.28
BCLTP8-055	5	ENSG00000211670	IGLV3-9	immunoglobulin lambda variable 3-9 (gene/pseudogene) [Source:HGNC Symbol;Acc:HGNC:5918]	1.91
BCLTP8-055	5	ENSG00000197780	TAF13	TATA-box binding protein associated factor 13 [Source:HGNC Symbol;Acc:HGNC:11546]	0.49
BCLTP8-056	3	ENSG00000024526	DEPDC1	DEP domain containing 1 [Source:HGNC Symbol;Acc:HGNC:22949]	0.44
BCLTP8-056	3	ENSG00000254176	IGHV3-75	immunoglobulin heavy variable 3-75 (pseudogene) [Source:HGNC Symbol;Acc:HGNC:5625]	1.47
BCLTP8-056	3	ENSG00000240382	IGKV1-17	immunoglobulin kappa variable 1-17 [Source:HGNC Symbol;Acc:HGNC:5733]	0.45
BCLTP8-057	2	ENSG00000181800	CELF2-AS1	CELF2 antisense RNA 1 [Source:HGNC Symbol;Acc:HGNC:23515]	-0.82
BCLTP8-057	2	ENSG00000224699	LAMTOR5-AS1	LAMTOR5 antisense RNA 1 [Source:HGNC Symbol;Acc:HGNC:40823]	-0.44
BCLTP8-058	2	ENSG00000211673	IGLV3-1	immunoglobulin lambda variable 3-1 [Source:HGNC Symbol;Acc:HGNC:5896]	1.07
BCLTP8-058	2	ENSG00000104889	RNASEH2A	ribonuclease H2 subunit A [Source:HGNC Symbol;Acc:HGNC:18518]	0.25
BCLTP8-059	3	ENSG00000137806	NDUFAF1	NADH:ubiquinone oxidoreductase complex assembly factor 1 [Source:HGNC Symbol;Acc:HGNC:18828]	0.41
BCLTP8-059	3	ENSG00000107719	PALD1	phosphatase domain containing, paladin 1 [Source:HGNC Symbol;Acc:HGNC:23530]	0.37
BCLTP8-059	3	ENSG00000025039	RRAGD	Ras related GTP binding D [Source:HGNC Symbol;Acc:HGNC:19903]	0.82
BCLTP8-060	4	ENSG00000259772			0.52
BCLTP8-060	4	ENSG00000253822	IGLV3-24	immunoglobulin lambda variable 3-24 (pseudogene) [Source:HGNC Symbol;Acc:HGNC:5907]	1.76
BCLTP8-060	4	ENSG00000092621	PHGDH	phosphoglycerate dehydrogenase [Source:HGNC Symbol;Acc:HGNC:8923]	0.53
BCLTP8-060	4	ENSG00000170027	YWHAG	tyrosine 3-monooxygenase/tryptophan 5-monooxygenase activation protein gamma [Source:HGNC Symbol;Acc:HGNC:12852]	0.44
BCLTP8-061	3	ENSG00000274272			-0.29
BCLTP8-061	3	ENSG00000178127	NDUFV2	NADH:ubiquinone oxidoreductase core subunit V2 [Source:HGNC Symbol;Acc:HGNC:7717]	-0.93
BCLTP8-061	3	ENSG00000100154	TTC28	tetratricopeptide repeat domain 28 [Source:HGNC Symbol;Acc:HGNC:29179]	-0.64
BCLTP8-062	6	ENSG00000216775			1.15
BCLTP8-062	6	ENSG00000178445	GLDC	glycine decarboxylase [Source:HGNC Symbol;Acc:HGNC:4313]	1.01
BCLTP8-062	6	ENSG00000164109	MAD2L1	MAD2 mitotic arrest deficient-like 1 (yeast) [Source:HGNC Symbol;Acc:HGNC:6763]	0.45
BCLTP8-062	6	ENSG00000172965	MIR4435-2HG	MIR4435-2 host gene [Source:HGNC Symbol;Acc:HGNC:35163]	0.63

Cluster ID	Cluster Size	Gene ID	Gene Name	Gene Description	Log_2 FC TIV
BCLTP8-062	6	ENSG00000198794	SCAMP5	secretory carrier membrane protein 5 [Source:HGNC Symbol;Acc:HGNC:30386]	0.96
BCLTP8-062	6	ENSG00000169962	TAS1R3	taste 1 receptor member 3 [Source:HGNC Symbol;Acc:HGNC:15661]	0.65
BCLTP8-063	4	ENSG00000187837	HIST1H1C	histone cluster 1 H1 family member c [Source:HGNC Symbol;Acc:HGNC:4716]	0.31
BCLTP8-063	4	ENSG00000254174	IGHV1-12	immunoglobulin heavy variable 1-12 (pseudogene) [Source:HGNC Symbol;Acc:HGNC:5546]	1.70
BCLTP8-063	4	ENSG00000211967	IGHV3-53	immunoglobulin heavy variable 3-53 [Source:HGNC Symbol;Acc:HGNC:5610]	0.67
BCLTP8-063	4	ENSG00000211972	IGHV3-66	immunoglobulin heavy variable 3-66 [Source:HGNC Symbol;Acc:HGNC:5619]	0.96
BCLTP8-064	4	ENSG00000213430	HSPD1P1	heat shock protein family D (Hsp60) member 1 pseudogene 1 [Source:HGNC Symbol;Acc:HGNC:35133]	0.46
BCLTP8-064	4	ENSG00000211599	IGKV5-2	immunoglobulin kappa variable 5-2 [Source:HGNC Symbol;Acc:HGNC:5835]	0.63
BCLTP8-064	4	ENSG00000144182	LIPT1	lipoyltransferase 1 [Source:HGNC Symbol;Acc:HGNC:29569]	0.44
BCLTP8-064	4	ENSG00000173578	XCR1	X-C motif chemokine receptor 1 [Source:HGNC Symbol;Acc:HGNC:1625]	1.08
BCLTP8-065	3	ENSG00000248571			1.09
BCLTP8-065	3	ENSG00000224041	IGKV3D-15	immunoglobulin kappa variable 3D-15 (gene/pseudogene) [Source:HGNC Symbol;Acc:HGNC:5824]	0.56
BCLTP8-065	3	ENSG00000185480	PARPBP	PARP1 binding protein [Source:HGNC Symbol;Acc:HGNC:26074]	0.39
BCLTP8-066	8	ENSG00000232176			-0.83
BCLTP8-066	8	ENSG00000241666			-0.73
BCLTP8-066	8	ENSG00000168209	DDIT4	DNA damage inducible transcript 4 [Source:HGNC Symbol;Acc:HGNC:24944]	-0.86
BCLTP8-066	8	ENSG00000141753	IGFBP4	insulin like growth factor binding protein 4 [Source:HGNC Symbol;Acc:HGNC:5473]	-0.31
BCLTP8-066	8	ENSG00000172548	NIPAL4	NIPA like domain containing 4 [Source:HGNC Symbol;Acc:HGNC:28018]	-0.21
BCLTP8-066	8	ENSG00000080546	SESN1	sestrin 1 [Source:HGNC Symbol;Acc:HGNC:21595]	-0.59
BCLTP8-066	8	ENSG00000006638	TBXA2R	thromboxane A2 receptor [Source:HGNC Symbol;Acc:HGNC:11608]	-0.51
BCLTP8-066	8	ENSG00000196263	ZNF471	zinc finger protein 471 [Source:HGNC Symbol;Acc:HGNC:23226]	-0.67

Table 34: Co-expressed gene clusters (B Cells, Day 8)

Cluster ID	Cluster Size	Gene ID	Gene Name	Gene Description	Log_2 FC TIV
BCLTP9-001	2	ENSG00000211892	IGHG4	immunoglobulin heavy constant gamma 4 (G4m marker) [Source:HGNC Symbol;Acc:HGNC:5528]	0.30
BCLTP9-001	2	ENSG00000254176	IGHV3-75	immunoglobulin heavy variable 3-75 (pseudogene) [Source:HGNC Symbol;Acc:HGNC:5625]	1.45
BCLTP9-002	2	ENSG00000211670	IGLV3-9	immunoglobulin lambda variable 3-9 (gene/pseudogene) [Source:HGNC Symbol;Acc:HGNC:5918]	1.64
BCLTP9-002	2	ENSG00000113140	SPARC	secreted protein acidic and cysteine rich [Source:HGNC Symbol;Acc:HGNC:11219]	1.52
BCLTP9-003	2	ENSG00000124788	ATXN1	ataxin 1 [Source:HGNC Symbol;Acc:HGNC:10548]	0.38
BCLTP9-003	2	ENSG00000211653	IGLV1-40	immunoglobulin lambda variable 1-40 [Source:HGNC Symbol;Acc:HGNC:5877]	1.37
BCLTP9-004	3	ENSG00000108641	B9D1	B9 domain containing 1 [Source:HGNC Symbol;Acc:HGNC:24123]	0.65
BCLTP9-004	3	ENSG00000188536	HBA2	hemoglobin subunit alpha 2 [Source:HGNC Symbol;Acc:HGNC:4824]	1.40
BCLTP9-004	3	ENSG00000244734	HBB	hemoglobin subunit beta [Source:HGNC Symbol;Acc:HGNC:4827]	1.55

Cluster ID	Cluster Size	Gene ID	Gene Name	Gene Description	Log_2 FC TIV
BCLTP9-005	5	ENSG00000121807	CCR2	C-C motif chemokine receptor 2 [Source:HGNC Symbol;Acc:HGNC:1603]	0.83
BCLTP9-005	5	ENSG00000174132	FAM174A	family with sequence similarity 174 member A [Source:HGNC Symbol;Acc:HGNC:24943]	0.70
BCLTP9-005	5	ENSG00000088340	FER1L4	fer-1 like family member 4, pseudogene [Source:HGNC Symbol;Acc:HGNC:15801]	1.19
BCLTP9-005	5	ENSG00000224041	IGKV3D-15	immunoglobulin kappa variable 3D-15 (gene/pseudogene) [Source:HGNC Symbol;Acc:HGNC:5824]	0.31
BCLTP9-005	5	ENSG00000169962	TAS1R3	taste 1 receptor member 3 [Source:HGNC Symbol;Acc:HGNC:15661]	0.49
BCLTP9-006	4	ENSG00000158578	ALAS2	5'-aminolevulinate synthase 2 [Source:HGNC Symbol;Acc:HGNC:397]	1.83
BCLTP9-006	4	ENSG00000107104	KANK1	KN motif and ankyrin repeat domains 1 [Source:HGNC Symbol;Acc:HGNC:19309]	0.54
BCLTP9-006	4	ENSG00000179222	MAGED1	MAGE family member D1 [Source:HGNC Symbol;Acc:HGNC:6813]	0.22
BCLTP9-006	4	ENSG00000004866	ST7	suppression of tumorigenicity 7 [Source:HGNC Symbol;Acc:HGNC:11351]	0.43
BCLTP9-007	10	ENSG00000119523	ALG2	ALG2, alpha-1,3/1,6-mannosyltransferase [Source:HGNC Symbol;Acc:HGNC:23159]	0.28
BCLTP9-007	10	ENSG00000186810	CXCR3	C-X-C motif chemokine receptor 3 [Source:HGNC Symbol;Acc:HGNC:4540]	0.79
BCLTP9-007	10	ENSG00000134153	EMC7	ER membrane protein complex subunit 7 [Source:HGNC Symbol;Acc:HGNC:24301]	0.32
BCLTP9-007	10	ENSG00000206172	HBA1	hemoglobin subunit alpha 1 [Source:HGNC Symbol;Acc:HGNC:4823]	1.42
BCLTP9-007	10	ENSG00000049860	HEXB	hexosaminidase subunit beta [Source:HGNC Symbol;Acc:HGNC:4879]	0.31
BCLTP9-007	10	ENSG00000224373	IGHV4-59	immunoglobulin heavy variable 4-59 [Source:HGNC Symbol;Acc:HGNC:5654]	0.98
BCLTP9-007	10	ENSG00000079931	MOXD1	monooxygenase DBH like 1 [Source:HGNC Symbol;Acc:HGNC:21063]	0.60
BCLTP9-007	10	ENSG00000115884	SDC1	syndecan 1 [Source:HGNC Symbol;Acc:HGNC:10658]	1.39
BCLTP9-007	10	ENSG00000163053	SLC16A14	solute carrier family 16 member 14 [Source:HGNC Symbol;Acc:HGNC:26417]	0.90
BCLTP9-007	10	ENSG00000173218	VANGL1	VANGL planar cell polarity protein 1 [Source:HGNC Symbol;Acc:HGNC:15512]	0.32
BCLTP9-008	3	ENSG00000211673	IGLV3-1	immunoglobulin lambda variable 3-1 [Source:HGNC Symbol;Acc:HGNC:5896]	0.70
BCLTP9-008	3	ENSG00000129636	ITFG1	integrin alpha FG-GAP repeat containing 1 [Source:HGNC Symbol;Acc:HGNC:30697]	0.26
BCLTP9-008	3	ENSG00000025039	RRAGD	Ras related GTP binding D [Source:HGNC Symbol;Acc:HGNC:19903]	0.42

Table 35: Co-expressed gene clusters (B Cells, Day 9)

Cluster ID	Cluster Size	Gene ID	Gene Name	Gene Description	Log_2 FC TIV
BCLTP10-001	2	ENSG00000211599	IGKV5-2	immunoglobulin kappa variable 5-2 [Source:HGNC Symbol;Acc:HGNC:5835]	0.76
BCLTP10-001	2	ENSG00000179222	MAGED1	MAGE family member D1 [Source:HGNC Symbol;Acc:HGNC:6813]	0.14
BCLTP10-002	4	ENSG00000123080	CDKN2C	cyclin dependent kinase inhibitor 2C [Source:HGNC Symbol;Acc:HGNC:1789]	-0.16
BCLTP10-002	4	ENSG00000101439	CST3	cystatin C [Source:HGNC Symbol;Acc:HGNC:2475]	-0.88
BCLTP10-002	4	ENSG00000160883	HK3	hexokinase 3 [Source:HGNC Symbol;Acc:HGNC:4925]	-0.68
BCLTP10-002	4	ENSG00000166851	PLK1	polo like kinase 1 [Source:HGNC Symbol;Acc:HGNC:9077]	-0.28
BCLTP10-003	6	ENSG00000134153	EMC7	ER membrane protein complex subunit 7 [Source:HGNC Symbol;Acc:HGNC:24301]	0.14

Cluster ID	Cluster Size	Gene ID	Gene Name	Gene Description	Log_2 FC TIV
BCLTP10-003	6	ENSG00000231475	IGHV4-31	immunoglobulin heavy variable 4-31 [Source:HGNC Symbol;Acc:HGNC:5649]	0.29
BCLTP10-003	6	ENSG00000211670	IGLV3-9	immunoglobulin lambda variable 3-9 (gene/pseudogene) [Source:HGNC Symbol;Acc:HGNC:5918]	1.52
BCLTP10-003	6	ENSG00000120725	SIL1	SIL1 nucleotide exchange factor [Source:HGNC Symbol;Acc:HGNC:24624]	0.11
BCLTP10-003	6	ENSG00000114902	SPCS1	signal peptidase complex subunit 1 [Source:HGNC Symbol;Acc:HGNC:23401]	0.07
BCLTP10-003	6	ENSG00000154277	UCHL1	ubiquitin C-terminal hydrolase L1 [Source:HGNC Symbol;Acc:HGNC:12513]	0.36

Table 36: Co-expressed gene clusters (B Cells, Day 10)

Cluster ID	Cluster Size	Gene ID	Gene Name	Gene Description	Log_2 FC TIV
BCLALLTP-001	2	ENSG00000183508	FAM46C	family with sequence similarity 46 member C [Source:HGNC Symbol;Acc:HGNC:24712]	
BCLALLTP-001	2	ENSG00000106415	GLCCI1	glucocorticoid induced 1 [Source:HGNC Symbol;Acc:HGNC:18713]	
BCLALLTP-002	5	ENSG00000166598	HSP90B1	heat shock protein 90 beta family member 1 [Source:HGNC Symbol;Acc:HGNC:12028]	
BCLALLTP-002	5	ENSG00000044574	HSPA5	heat shock protein family A (Hsp70) member 5 [Source:HGNC Symbol;Acc:HGNC:5238]	
BCLALLTP-002	5	ENSG00000074695	LMAN1	lectin, mannose binding 1 [Source:HGNC Symbol;Acc:HGNC:6631]	
BCLALLTP-002	5	ENSG00000166794	PPIB	peptidylprolyl isomerase B [Source:HGNC Symbol;Acc:HGNC:9255]	
BCLALLTP-002	5	ENSG00000166562	SEC11C	SEC11 homolog C, signal peptidase complex subunit [Source:HGNC Symbol;Acc:HGNC:23400]	
BCLALLTP-003	2	ENSG00000106803	SEC61B	Sec61 translocon beta subunit [Source:HGNC Symbol;Acc:HGNC:16993]	
BCLALLTP-003	2	ENSG00000121073	SLC35B1	solute carrier family 35 member B1 [Source:HGNC Symbol;Acc:HGNC:20798]	
BCLALLTP-004	3	ENSG00000168374	ARF4	ADP ribosylation factor 4 [Source:HGNC Symbol;Acc:HGNC:655]	
BCLALLTP-004	3	ENSG00000124783	SSR1	signal sequence receptor subunit 1 [Source:HGNC Symbol;Acc:HGNC:11323]	
BCLALLTP-004	3	ENSG00000170348	TMED10	transmembrane p24 trafficking protein 10 [Source:HGNC Symbol;Acc:HGNC:16998]	
BCLALLTP-005	2	ENSG00000110063	DCPS	decapping enzyme, scavenger [Source:HGNC Symbol;Acc:HGNC:29812]	
BCLALLTP-005	2	ENSG00000176658	MYO1D	myosin ID [Source:HGNC Symbol;Acc:HGNC:7598]	
BCLALLTP-006	2	ENSG00000111640	GAPDH	glyceraldehyde-3-phosphate dehydrogenase [Source:HGNC Symbol;Acc:HGNC:4141]	
BCLALLTP-006	2	ENSG00000101057	MYBL2	MYB proto-oncogene like 2 [Source:HGNC Symbol;Acc:HGNC:7548]	
BCLALLTP-007	4	ENSG00000136770	DNAJC1	DnaJ heat shock protein family (Hsp40) member C1 [Source:HGNC Symbol;Acc:HGNC:20090]	
BCLALLTP-007	4	ENSG00000129128	SPCS3	signal peptidase complex subunit 3 [Source:HGNC Symbol;Acc:HGNC:26212]	
BCLALLTP-007	4	ENSG00000163527	STT3B	STT3B, catalytic subunit of the oligosaccharyltransferase complex [Source:HGNC Symbol;Acc:HGNC:30611]	
BCLALLTP-007	4	ENSG00000067167	TRAM1	translocation associated membrane protein 1 [Source:HGNC Symbol;Acc:HGNC:20568]	
BCLALLTP-008	8	ENSG00000259706	HSP90B2P	heat shock protein 90 beta family member 2, pseudogene [Source:HGNC Symbol;Acc:HGNC:12099]	
BCLALLTP-008	8	ENSG00000145050	MANF	mesencephalic astrocyte derived neurotrophic factor [Source:HGNC Symbol;Acc:HGNC:15461]	
BCLALLTP-008	8	ENSG00000198856	OSTC	oligosaccharyltransferase complex non-catalytic subunit [Source:HGNC Symbol;Acc:HGNC:24448]	

Cluster ID	Cluster Size	Gene ID	Gene Name	Gene Description	Log_2 FC TIV
BCLALLTP-008	8	ENSG00000131871	SELENOS	selenoprotein S [Source:HGNC Symbol;Acc:HGNC:30396]	
BCLALLTP-008	8	ENSG00000070214	SLC44A1	solute carrier family 44 member 1 [Source:HGNC Symbol;Acc:HGNC:18798]	
BCLALLTP-008	8	ENSG00000114850	SSR3	signal sequence receptor subunit 3 [Source:HGNC Symbol;Acc:HGNC:11325]	
BCLALLTP-008	8	ENSG00000113387	SUB1	SUB1 homolog, transcriptional regulator [Source:HGNC Symbol;Acc:HGNC:19985]	
BCLALLTP-008	8	ENSG00000048462	TNFRSF17	TNF receptor superfamily member 17 [Source:HGNC Symbol;Acc:HGNC:11913]	
BCLALLTP-009	2	ENSG00000243238	IGKV2-30	immunoglobulin kappa variable 2-30 [Source:HGNC Symbol;Acc:HGNC:5785]	
BCLALLTP-009	2	ENSG00000239571	IGKV2D-30	immunoglobulin kappa variable 2D-30 [Source:HGNC Symbol;Acc:HGNC:5801]	
BCLALLTP-010	3	ENSG00000106105	GARS	glycyl-tRNA synthetase [Source:HGNC Symbol;Acc:HGNC:4162]	
BCLALLTP-010	3	ENSG00000108829	LRRC59	leucine rich repeat containing 59 [Source:HGNC Symbol;Acc:HGNC:28817]	
BCLALLTP-010	3	ENSG00000110917	MLEC	malectin [Source:HGNC Symbol;Acc:HGNC:28973]	
BCLALLTP-011	2	ENSG00000182481	KPNA2	karyopherin subunit alpha 2 [Source:HGNC Symbol;Acc:HGNC:6395]	
BCLALLTP-011	2	ENSG00000167325	RRM1	ribonucleotide reductase catalytic subunit M1 [Source:HGNC Symbol;Acc:HGNC:10451]	
BCLALLTP-012	7	ENSG00000171155	C1GALT1C1	C1GALT1 specific chaperone 1 [Source:HGNC Symbol;Acc:HGNC:24338]	
BCLALLTP-012	7	ENSG00000075420	FNDC3B	fibronectin type III domain containing 3B [Source:HGNC Symbol;Acc:HGNC:24670]	
BCLALLTP-012	7	ENSG00000198380	GFPT1	glutamine–fructose-6-phosphate transaminase 1 [Source:HGNC Symbol;Acc:HGNC:4241]	
BCLALLTP-012	7	ENSG00000155304	HSPA13	heat shock protein family A (Hsp70) member 13 [Source:HGNC Symbol;Acc:HGNC:11375]	
BCLALLTP-012	7	ENSG00000172469	MANEA	mannosidase endo-alpha [Source:HGNC Symbol;Acc:HGNC:21072]	
BCLALLTP-012	7	ENSG00000071537	SEL1L	SEL1L ERAD E3 ligase adaptor subunit [Source:HGNC Symbol;Acc:HGNC:10717]	
BCLALLTP-012	7	ENSG00000138768	USO1	USO1 vesicle transport factor [Source:HGNC Symbol;Acc:HGNC:30904]	
BCLALLTP-013	3	ENSG00000135476	ESPL1	extra spindle pole bodies like 1, separase [Source:HGNC Symbol;Acc:HGNC:16856]	
BCLALLTP-013	3	ENSG00000237649	KIFC1	kinesin family member C1 [Source:HGNC Symbol;Acc:HGNC:6389]	
BCLALLTP-013	3	ENSG00000076382	SPAG5	sperm associated antigen 5 [Source:HGNC Symbol;Acc:HGNC:13452]	
BCLALLTP-014	3	ENSG00000165272	AQP3	aquaporin 3 (Gill blood group) [Source:HGNC Symbol;Acc:HGNC:636]	
BCLALLTP-014	3	ENSG00000160712	IL6R	interleukin 6 receptor [Source:HGNC Symbol;Acc:HGNC:6019]	
BCLALLTP-014	3	ENSG00000112378	PERP	PERP, TP53 apoptosis effector [Source:HGNC Symbol;Acc:HGNC:17637]	
BCLALLTP-015	2	ENSG00000198826	ARHGAP11A	Rho GTPase activating protein 11A [Source:HGNC Symbol;Acc:HGNC:15783]	
BCLALLTP-015	2	ENSG00000051341	POLQ	DNA polymerase theta [Source:HGNC Symbol;Acc:HGNC:9186]	
BCLALLTP-016	8	ENSG00000145386	CCNA2	cyclin A2 [Source:HGNC Symbol;Acc:HGNC:1578]	
BCLALLTP-016	8	ENSG00000117399	CDC20	cell division cycle 20 [Source:HGNC Symbol;Acc:HGNC:1723]	
BCLALLTP-016	8	ENSG00000072571	HMMR	hyaluronan mediated motility receptor [Source:HGNC Symbol;Acc:HGNC:5012]	
BCLALLTP-016	8	ENSG00000138160	KIF11	kinesin family member 11 [Source:HGNC Symbol;Acc:HGNC:6388]	
BCLALLTP-016	8	ENSG00000104738	MCM4	minichromosome maintenance complex component 4 [Source:HGNC Symbol;Acc:HGNC:6947]	
BCLALLTP-016	8	ENSG00000076003	MCM6	minichromosome maintenance complex component 6 [Source:HGNC Symbol;Acc:HGNC:6949]	

Cluster ID	Cluster Size	Gene ID	Gene Name	Gene Description	Log_2 FC TIV
BCLALLTP-016	8	ENSG00000132646	PCNA	proliferating cell nuclear antigen [Source:HGNC Symbol;Acc:HGNC:8729]	
BCLALLTP-016	8	ENSG00000131747	TOP2A	topoisomerase (DNA) II alpha [Source:HGNC Symbol;Acc:HGNC:11989]	
BCLALLTP-017	4	ENSG00000124788	ATXN1	ataxin 1 [Source:HGNC Symbol;Acc:HGNC:10548]	
BCLALLTP-017	4	ENSG00000140743	CDR2	cerebellar degeneration related protein 2 [Source:HGNC Symbol;Acc:HGNC:1799]	
BCLALLTP-017	4	ENSG00000211938	IGHV3-7	immunoglobulin heavy variable 3-7 [Source:HGNC Symbol;Acc:HGNC:5620]	
BCLALLTP-017	4	ENSG00000070540	WIPI1	WD repeat domain, phosphoinositide interacting 1 [Source:HGNC Symbol;Acc:HGNC:25471]	
BCLALLTP-018	2	ENSG00000198855	FICD	FIC domain containing [Source:HGNC Symbol;Acc:HGNC:18416]	
BCLALLTP-018	2	ENSG00000145817	YIPF5	Yip1 domain family member 5 [Source:HGNC Symbol;Acc:HGNC:24877]	
BCLALLTP-019	3	ENSG00000040933	INPP4A	inositol polyphosphate-4-phosphatase type I A [Source:HGNC Symbol;Acc:HGNC:6074]	
BCLALLTP-019	3	ENSG00000122188	LAX1	lymphocyte transmembrane adaptor 1 [Source:HGNC Symbol;Acc:HGNC:26005]	
BCLALLTP-019	3	ENSG00000136161	RCBTB2	RCC1 and BTB domain containing protein 2 [Source:HGNC Symbol;Acc:HGNC:1914]	
BCLALLTP-020	2	ENSG00000142731	PLK4	polo like kinase 4 [Source:HGNC Symbol;Acc:HGNC:11397]	
BCLALLTP-020	2	ENSG00000123473	STIL	SCL/TAL1 interrupting locus [Source:HGNC Symbol;Acc:HGNC:10879]	
BCLALLTP-021	5	ENSG00000184432	COPB2	coatomer protein complex subunit beta 2 [Source:HGNC Symbol;Acc:HGNC:2232]	
BCLALLTP-021	5	ENSG00000134153	EMC7	ER membrane protein complex subunit 7 [Source:HGNC Symbol;Acc:HGNC:24301]	
BCLALLTP-021	5	ENSG00000102158	MAGT1	magnesium transporter 1 [Source:HGNC Symbol;Acc:HGNC:28880]	
BCLALLTP-021	5	ENSG00000004866	ST7	suppression of tumorigenicity 7 [Source:HGNC Symbol;Acc:HGNC:11351]	
BCLALLTP-021	5	ENSG00000198900	TOP1	topoisomerase (DNA) I [Source:HGNC Symbol;Acc:HGNC:11986]	
BCLALLTP-022	8	ENSG00000066279	ASPM	abnormal spindle microtubule assembly [Source:HGNC Symbol;Acc:HGNC:19048]	
BCLALLTP-022	8	ENSG00000094804	CDC6	cell division cycle 6 [Source:HGNC Symbol;Acc:HGNC:1744]	
BCLALLTP-022	8	ENSG00000117724	CENPF	centromere protein F [Source:HGNC Symbol;Acc:HGNC:1857]	
BCLALLTP-022	8	ENSG00000106462	EZH2	enhancer of zeste 2 polycomb repressive complex 2 subunit [Source:HGNC Symbol;Acc:HGNC:3527]	
BCLALLTP-022	8	ENSG00000140525	FANCI	Fanconi anemia complementation group I [Source:HGNC Symbol;Acc:HGNC:25568]	
BCLALLTP-022	8	ENSG00000168496	FEN1	flap structure-specific endonuclease 1 [Source:HGNC Symbol;Acc:HGNC:3650]	
BCLALLTP-022	8	ENSG00000075218	GTSE1	G2 and S-phase expressed 1 [Source:HGNC Symbol;Acc:HGNC:13698]	
BCLALLTP-022	8	ENSG00000088325	TPX2	TPX2, microtubule nucleation factor [Source:HGNC Symbol;Acc:HGNC:1249]	
BCLALLTP-023	2	ENSG00000242076	IGKV1-33	immunoglobulin kappa variable 1-33 [Source:HGNC Symbol;Acc:HGNC:5737]	
BCLALLTP-023	2	ENSG00000239975	IGKV1D-33	immunoglobulin kappa variable 1D-33 [Source:HGNC Symbol;Acc:HGNC:5753]	
BCLALLTP-024	3	ENSG00000216775			
BCLALLTP-024	3	ENSG00000178445	GLDC	glycine decarboxylase [Source:HGNC Symbol;Acc:HGNC:4313]	
BCLALLTP-024	3	ENSG00000198794	SCAMP5	secretory carrier membrane protein 5 [Source:HGNC Symbol;Acc:HGNC:30386]	
BCLALLTP-025	2	ENSG00000211935	IGHV1-3	immunoglobulin heavy variable 1-3 [Source:HGNC Symbol;Acc:HGNC:5552]	
BCLALLTP-025	2	ENSG00000211644	IGLV1-51	immunoglobulin lambda variable 1-51 [Source:HGNC Symbol;Acc:HGNC:5882]	

Cluster ID	Cluster Size	Gene ID	Gene Name	Gene Description	Log_2 FC TIV
BCLALLTP-026	4	ENSG00000095380	NANS	N-acetylneuraminate synthase [Source:HGNC Symbol;Acc:HGNC:19237]	
BCLALLTP-026	4	ENSG00000083444	PLOD1	procollagen-lysine,2-oxoglutarate 5-dioxygenase 1 [Source:HGNC Symbol;Acc:HGNC:9081]	
BCLALLTP-026	4	ENSG00000136868	SLC31A1	solute carrier family 31 member 1 [Source:HGNC Symbol;Acc:HGNC:11016]	
BCLALLTP-026	4	ENSG00000103257	SLC7A5	solute carrier family 7 member 5 [Source:HGNC Symbol;Acc:HGNC:11063]	
BCLALLTP-027	2	ENSG00000242371	IGKV1-39	immunoglobulin kappa variable 1-39 (gene/pseudogene) [Source:HGNC Symbol;Acc:HGNC:5740]	
BCLALLTP-027	2	ENSG00000251546	IGKV1D-39	immunoglobulin kappa variable 1D-39 [Source:HGNC Symbol;Acc:HGNC:5756]	
BCLALLTP-028	3	ENSG00000157456	CCNB2	cyclin B2 [Source:HGNC Symbol;Acc:HGNC:1580]	
BCLALLTP-028	3	ENSG00000111206	FOXM1	forkhead box M1 [Source:HGNC Symbol;Acc:HGNC:3818]	
BCLALLTP-028	3	ENSG00000166803	KIAA0101	KIAA0101 [Source:HGNC Symbol;Acc:HGNC:28961]	
BCLALLTP-029	2	ENSG00000248571			
BCLALLTP-029	2	ENSG00000137806	NDUFAF1	NADH:ubiquinone oxidoreductase complex assembly factor 1 [Source:HGNC Symbol;Acc:HGNC:18828]	
BCLALLTP-030	7	ENSG00000012048	BRCA1	BRCA1, DNA repair associated [Source:HGNC Symbol;Acc:HGNC:1100]	
BCLALLTP-030	7	ENSG00000134057	CCNB1	cyclin B1 [Source:HGNC Symbol;Acc:HGNC:1579]	
BCLALLTP-030	7	ENSG00000024526	DEPDC1	DEP domain containing 1 [Source:HGNC Symbol;Acc:HGNC:22949]	
BCLALLTP-030	7	ENSG00000164109	MAD2L1	MAD2 mitotic arrest deficient-like 1 (yeast) [Source:HGNC Symbol;Acc:HGNC:6763]	
BCLALLTP-030	7	ENSG00000109805	NCAPG	non-SMC condensin I complex subunit G [Source:HGNC Symbol;Acc:HGNC:24304]	
BCLALLTP-030	7	ENSG00000137804	NUSAP1	nucleolar and spindle associated protein 1 [Source:HGNC Symbol;Acc:HGNC:18538]	
BCLALLTP-030	7	ENSG00000117632	STMN1	stathmin 1 [Source:HGNC Symbol;Acc:HGNC:6510]	
BCLALLTP-031	2	ENSG00000278857	IGKV1D-12	immunoglobulin kappa variable 1D-12 [Source:HGNC Symbol;Acc:HGNC:5746]	
BCLALLTP-031	2	ENSG00000211645	IGLV1-50	immunoglobulin lambda variable 1-50 (non-functional) [Source:HGNC Symbol;Acc:HGNC:5881]	
BCLALLTP-032	7	ENSG00000136010	ALDH1L2	aldehyde dehydrogenase 1 family member L2 [Source:HGNC Symbol;Acc:HGNC:26777]	
BCLALLTP-032	7	ENSG00000143641	GALNT2	polypeptide N-acetylgalactosaminyltransferase 2 [Source:HGNC Symbol;Acc:HGNC:4124]	
BCLALLTP-032	7	ENSG00000270472	IGHV3OR16-9	immunoglobulin heavy variable 3/OR16-9 (non-functional) [Source:HGNC Symbol;Acc:HGNC:5644]	
BCLALLTP-032	7	ENSG00000211648	IGLV1-47	immunoglobulin lambda variable 1-47 [Source:HGNC Symbol;Acc:HGNC:5880]	
BCLALLTP-032	7	ENSG00000099337	KCNK6	potassium two pore domain channel subfamily K member 6 [Source:HGNC Symbol;Acc:HGNC:6281]	
BCLALLTP-032	7	ENSG00000092621	PHGDH	phosphoglycerate dehydrogenase [Source:HGNC Symbol;Acc:HGNC:8923]	
BCLALLTP-032	7	ENSG00000109501	WFS1	wolframin ER transmembrane glycoprotein [Source:HGNC Symbol;Acc:HGNC:12762]	
BCLALLTP-033	3	ENSG00000150967	ABCB9	ATP binding cassette subfamily B member 9 [Source:HGNC Symbol;Acc:HGNC:50]	
BCLALLTP-033	3	ENSG00000107719	PALD1	phosphatase domain containing, paladin 1 [Source:HGNC Symbol;Acc:HGNC:23530]	
BCLALLTP-033	3	ENSG00000198722	UNC13B	unc-13 homolog B [Source:HGNC Symbol;Acc:HGNC:12566]	
BCLALLTP-034	2	ENSG00000222041	CYTOR	cytoskeleton regulator RNA [Source:HGNC Symbol;Acc:HGNC:28717]	
BCLALLTP-034	2	ENSG00000235587	GAPDHP65	glyceraldehyde 3 phosphate dehydrogenase pseudogene 65 [Source:HGNC Symbol;Acc:HGNC:4143]	
BCLALLTP-035	2	ENSG00000227155			
BCLALLTP-035	2	ENSG00000280138			
BCLALLTP-036	4	ENSG00000101003	GINS1	GINS complex subunit 1 [Source:HGNC Symbol;Acc:HGNC:28980]	

Cluster ID	Cluster Size	Gene ID	Gene Name	Gene Description	Log_2 FC TIV
BCLALLTP-036	4	ENSG00000118193	KIF14	kinesin family member 14 [Source:HGNC Symbol;Acc:HGNC:19181]	
BCLALLTP-036	4	ENSG00000137812	KNL1	kinetochore scaffold 1 [Source:HGNC Symbol;Acc:HGNC:24054]	
BCLALLTP-036	4	ENSG00000164611	PTTG1	pituitary tumor-transforming 1 [Source:HGNC Symbol;Acc:HGNC:9690]	
BCLALLTP-037	4	ENSG00000211952	IGHV4-28	immunoglobulin heavy variable 4-28 [Source:HGNC Symbol;Acc:HGNC:5645]	
BCLALLTP-037	4	ENSG00000211677	IGLC2	immunoglobulin lambda constant 2 [Source:HGNC Symbol;Acc:HGNC:5856]	
BCLALLTP-037	4	ENSG00000211653	IGLV1-40	immunoglobulin lambda variable 1-40 [Source:HGNC Symbol;Acc:HGNC:5877]	
BCLALLTP-037	4	ENSG00000211662	IGLV3-21	immunoglobulin lambda variable 3-21 [Source:HGNC Symbol;Acc:HGNC:5905]	
BCLALLTP-038	2	ENSG00000279759			
BCLALLTP-038	2	ENSG00000279873	LINC01126	long intergenic non-protein coding RNA 1126 [Source:HGNC Symbol;Acc:HGNC:49275]	
BCLALLTP-039	2	ENSG00000254176	IGHV3-75	immunoglobulin heavy variable 3-75 (pseudogene) [Source:HGNC Symbol;Acc:HGNC:5625]	
BCLALLTP-039	2	ENSG00000211670	IGLV3-9	immunoglobulin lambda variable 3-9 (gene/pseudogene) [Source:HGNC Symbol;Acc:HGNC:5918]	
BCLALLTP-040	5	ENSG00000108641	B9D1	B9 domain containing 1 [Source:HGNC Symbol;Acc:HGNC:24123]	
BCLALLTP-040	5	ENSG00000138709	LARP1B	La ribonucleoprotein domain family member 1B [Source:HGNC Symbol;Acc:HGNC:24704]	
BCLALLTP-040	5	ENSG00000069956	MAPK6	mitogen-activated protein kinase 6 [Source:HGNC Symbol;Acc:HGNC:6879]	
BCLALLTP-040	5	ENSG00000102471	NDFIP2	Nedd4 family interacting protein 2 [Source:HGNC Symbol;Acc:HGNC:18537]	
BCLALLTP-040	5	ENSG00000184232	OAF	out at first homolog [Source:HGNC Symbol;Acc:HGNC:28752]	
BCLALLTP-041	6	ENSG00000148468	FAM171A1	family with sequence similarity 171 member A1 [Source:HGNC Symbol;Acc:HGNC:23522]	
BCLALLTP-041	6	ENSG00000224373	IGHV4-59	immunoglobulin heavy variable 4-59 [Source:HGNC Symbol;Acc:HGNC:5654]	
BCLALLTP-041	6	ENSG00000242766	IGKV1D-17	immunoglobulin kappa variable 1D-17 [Source:HGNC Symbol;Acc:HGNC:5749]	
BCLALLTP-041	6	ENSG00000241351	IGKV3-11	immunoglobulin kappa variable 3-11 [Source:HGNC Symbol;Acc:HGNC:5815]	
BCLALLTP-041	6	ENSG00000115884	SDC1	syndecan 1 [Source:HGNC Symbol;Acc:HGNC:10658]	
BCLALLTP-041	6	ENSG00000113140	SPARC	secreted protein acidic and cysteine rich [Source:HGNC Symbol;Acc:HGNC:11219]	
BCLALLTP-042	3	ENSG00000271533			
BCLALLTP-042	3	ENSG00000274422			
BCLALLTP-042	3	ENSG00000279278			
BCLALLTP-043	3	ENSG00000187837	HIST1H1C	histone cluster 1 H1 family member c [Source:HGNC Symbol;Acc:HGNC:4716]	
BCLALLTP-043	3	ENSG00000211967	IGHV3-53	immunoglobulin heavy variable 3-53 [Source:HGNC Symbol;Acc:HGNC:5610]	
BCLALLTP-043	3	ENSG00000211972	IGHV3-66	immunoglobulin heavy variable 3-66 [Source:HGNC Symbol;Acc:HGNC:5619]	
BCLALLTP-044	4	ENSG00000110848	CD69	CD69 molecule [Source:HGNC Symbol;Acc:HGNC:1694]	
BCLALLTP-044	4	ENSG00000161960	EIF4A1	eukaryotic translation initiation factor 4A1 [Source:HGNC Symbol;Acc:HGNC:3282]	
BCLALLTP-044	4	ENSG00000171223	JUNB	JunB proto-oncogene, AP-1 transcription factor subunit [Source:HGNC Symbol;Acc:HGNC:6205]	
BCLALLTP-044	4	ENSG00000100906	NFKBIA	NFKB inhibitor alpha [Source:HGNC Symbol;Acc:HGNC:7797]	
BCLALLTP-045	4	ENSG00000143942	CHAC2	ChaC cation transport regulator homolog 2 [Source:HGNC Symbol;Acc:HGNC:32363]	
BCLALLTP-045	4	ENSG00000123975	CKS2	CDC28 protein kinase regulatory subunit 2 [Source:HGNC Symbol;Acc:HGNC:2000]	

Cluster ID	Cluster Size	Gene ID	Gene Name	Gene Description	Log_2 FC TIV
BCLALLTP-045	4	ENSG00000119326	CTNNAL1	catenin alpha like 1 [Source:HGNC Symbol;Acc:HGNC:2512]	
BCLALLTP-045	4	ENSG00000239672	NME1	NME/NM23 nucleoside diphosphate kinase 1 [Source:HGNC Symbol;Acc:HGNC:7849]	
BCLALLTP-046	2	ENSG00000211962	IGHV1-46	immunoglobulin heavy variable 1-46 [Source:HGNC Symbol;Acc:HGNC:5554]	
BCLALLTP-046	2	ENSG00000211976	IGHV3-73	immunoglobulin heavy variable 3-73 [Source:HGNC Symbol;Acc:HGNC:5623]	
BCLALLTP-047	2	ENSG00000090104	RGS1	regulator of G-protein signaling 1 [Source:HGNC Symbol;Acc:HGNC:9991]	
BCLALLTP-047	2	ENSG00000116741	RGS2	regulator of G-protein signaling 2 [Source:HGNC Symbol;Acc:HGNC:9998]	
BCLALLTP-048	4	ENSG00000172339	ALG14	ALG14, UDP-N-acetylglucosaminyltransferase subunit [Source:HGNC Symbol;Acc:HGNC:28287]	
BCLALLTP-048	4	ENSG00000113273	ARSB	arylsulfatase B [Source:HGNC Symbol;Acc:HGNC:714]	
BCLALLTP-048	4	ENSG00000182197	EXT1	exostosin glycosyltransferase 1 [Source:HGNC Symbol;Acc:HGNC:3512]	
BCLALLTP-048	4	ENSG00000025039	RRAGD	Ras related GTP binding D [Source:HGNC Symbol;Acc:HGNC:19903]	
BCLALLTP-049	3	ENSG00000163751	CPA3	carboxypeptidase A3 [Source:HGNC Symbol;Acc:HGNC:2298]	
BCLALLTP-049	3	ENSG00000134489	HRH4	histamine receptor H4 [Source:HGNC Symbol;Acc:HGNC:17383]	
BCLALLTP-049	3	ENSG00000149516	MS4A3	membrane spanning 4-domains A3 [Source:HGNC Symbol;Acc:HGNC:7317]	
BCLALLTP-050	5	ENSG00000282600			
BCLALLTP-050	5	ENSG00000211943	IGHV3-15	immunoglobulin heavy variable 3-15 [Source:HGNC Symbol;Acc:HGNC:5582]	
BCLALLTP-050	5	ENSG00000254395	IGHV4-55	immunoglobulin heavy variable 4-55 (pseudogene) [Source:HGNC Symbol;Acc:HGNC:5653]	
BCLALLTP-050	5	ENSG00000211679	IGLC3	immunoglobulin lambda constant 3 (Kern-Oz+ marker) [Source:HGNC Symbol;Acc:HGNC:5857]	
BCLALLTP-050	5	ENSG00000211666	IGLV2-14	immunoglobulin lambda variable 2-14 [Source:HGNC Symbol;Acc:HGNC:5888]	
BCLALLTP-051	3	ENSG00000141753	IGFBP4	insulin like growth factor binding protein 4 [Source:HGNC Symbol;Acc:HGNC:5473]	
BCLALLTP-051	3	ENSG00000172548	NIPAL4	NIPA like domain containing 4 [Source:HGNC Symbol;Acc:HGNC:28018]	
BCLALLTP-051	3	ENSG00000228903	RASA4CP	RAS p21 protein activator 4C, pseudogene [Source:HGNC Symbol;Acc:HGNC:44185]	
BCLALLTP-052	4	ENSG00000158578	ALAS2	5'-aminolevulinate synthase 2 [Source:HGNC Symbol;Acc:HGNC:397]	
BCLALLTP-052	4	ENSG00000206172	HBA1	hemoglobin subunit alpha 1 [Source:HGNC Symbol;Acc:HGNC:4823]	
BCLALLTP-052	4	ENSG00000188536	HBA2	hemoglobin subunit alpha 2 [Source:HGNC Symbol;Acc:HGNC:4824]	
BCLALLTP-052	4	ENSG00000244734	HBB	hemoglobin subunit beta [Source:HGNC Symbol;Acc:HGNC:4827]	

Table 37: Co-expressed gene clusters (B Cells, All post-treatment time points)

Cluster ID	Cluster Size	Gene ID	Gene Name	Gene Description	Log_2 FC TIV
PMCTP1-001	2	ENSG00000135604	STX11	syntaxin 11 [Source:HGNC Symbol;Acc:HGNC:11429]	0.76
PMCTP1-001	2	ENSG00000140105	WARS	tryptophanyl-tRNA synthetase [Source:HGNC Symbol;Acc:HGNC:12729]	0.85
PMCTP1-002	2	ENSG00000140749	IGSF6	immunoglobulin superfamily member 6 [Source:HGNC Symbol;Acc:HGNC:5953]	0.61
PMCTP1-002	2	ENSG00000188906	LRRK2	leucine rich repeat kinase 2 [Source:HGNC Symbol;Acc:HGNC:18618]	0.66

Cluster ID	Cluster Size	Gene ID	Gene Name	Gene Description	Log_2 FC TIV
PMCTP1-003	2	ENSG00000103196	CRISPLD2	cysteine rich secretory protein LCCL domain containing 2 [Source:HGNC Symbol;Acc:HGNC:25248]	0.68
PMCTP1-003	2	ENSG00000183019	MCEMP1	mast cell expressed membrane protein 1 [Source:HGNC Symbol;Acc:HGNC:27291]	0.63
PMCTP1-004	2	ENSG00000114450	GNB4	G protein subunit beta 4 [Source:HGNC Symbol;Acc:HGNC:20731]	0.61
PMCTP1-004	2	ENSG00000149573	MPZL2	myelin protein zero like 2 [Source:HGNC Symbol;Acc:HGNC:3496]	0.63
PMCTP1-005	2	ENSG00000115919	KYNU	kynureninase [Source:HGNC Symbol;Acc:HGNC:6469]	0.59
PMCTP1-005	2	ENSG00000145685	LHFPL2	lipoma HMGIC fusion partner-like 2 [Source:HGNC Symbol;Acc:HGNC:6588]	0.66
PMCTP1-006	3	ENSG00000171049	FPR2	formyl peptide receptor 2 [Source:HGNC Symbol;Acc:HGNC:3827]	0.77
PMCTP1-006	3	ENSG00000181631	P2RY13	purinergic receptor P2Y13 [Source:HGNC Symbol;Acc:HGNC:4537]	0.71
PMCTP1-006	3	ENSG00000166002	SMCO4	single-pass membrane protein with coiled-coil domains 4 [Source:HGNC Symbol;Acc:HGNC:24810]	0.63
PMCTP1-007	2	ENSG00000125347	IRF1	interferon regulatory factor 1 [Source:HGNC Symbol;Acc:HGNC:6116]	0.70
PMCTP1-007	2	ENSG00000138496	PARP9	poly(ADP-ribose) polymerase family member 9 [Source:HGNC Symbol;Acc:HGNC:24118]	0.66
PMCTP1-008	4	ENSG00000149798	CDC42EP2	CDC42 effector protein 2 [Source:HGNC Symbol;Acc:HGNC:16263]	0.84
PMCTP1-008	4	ENSG00000150337	FCGR1A	Fc fragment of IgG receptor Ia [Source:HGNC Symbol;Acc:HGNC:3613]	1.35
PMCTP1-008	4	ENSG00000111331	OAS3	2'-5'-oligoadenylate synthetase 3 [Source:HGNC Symbol;Acc:HGNC:8088]	0.62
PMCTP1-008	4	ENSG00000149131	SERPING1	serpin family G member 1 [Source:HGNC Symbol;Acc:HGNC:1228]	1.81
PMCTP1-009	4	ENSG00000152766	ANKRD22	ankyrin repeat domain 22 [Source:HGNC Symbol;Acc:HGNC:28321]	1.75
PMCTP1-009	4	ENSG00000121807	CCR2	C-C motif chemokine receptor 2 [Source:HGNC Symbol;Acc:HGNC:1603]	0.59
PMCTP1-009	4	ENSG00000082397	EPB41L3	erythrocyte membrane protein band 4.1 like 3 [Source:HGNC Symbol;Acc:HGNC:3380]	0.61
PMCTP1-009	4	ENSG00000002549	LAP3	leucine aminopeptidase 3 [Source:HGNC Symbol;Acc:HGNC:18449]	0.90
PMCTP1-010	3	ENSG00000136689	IL1RN	interleukin 1 receptor antagonist [Source:HGNC Symbol;Acc:HGNC:6000]	0.61
PMCTP1-010	3	ENSG00000038945	MSR1	macrophage scavenger receptor 1 [Source:HGNC Symbol;Acc:HGNC:7376]	0.53
PMCTP1-010	3	ENSG00000139832	RAB20	RAB20, member RAS oncogene family [Source:HGNC Symbol;Acc:HGNC:18260]	0.70
PMCTP1-011	5	ENSG00000168062	BATF2	basic leucine zipper ATF-like transcription factor 2 [Source:HGNC Symbol;Acc:HGNC:25163]	1.34
PMCTP1-011	5	ENSG00000133106	EPSTI1	epithelial stromal interaction 1 [Source:HGNC Symbol;Acc:HGNC:16465]	0.61
PMCTP1-011	5	ENSG00000116711	PLA2G4A	phospholipase A2 group IVA [Source:HGNC Symbol;Acc:HGNC:9035]	0.66
PMCTP1-011	5	ENSG00000115415	STAT1	signal transducer and activator of transcription 1 [Source:HGNC Symbol;Acc:HGNC:11362]	0.95
PMCTP1-011	5	ENSG00000156587	UBE2L6	ubiquitin conjugating enzyme E2 L6 [Source:HGNC Symbol;Acc:HGNC:12490]	0.69
PMCTP1-012	3	ENSG00000168389	MFSD2A	major facilitator superfamily domain containing 2A [Source:HGNC Symbol;Acc:HGNC:25897]	0.74
PMCTP1-012	3	ENSG00000141574	SECTM1	secreted and transmembrane 1 [Source:HGNC Symbol;Acc:HGNC:10707]	0.93
PMCTP1-012	3	ENSG00000180061	TMEM150B	transmembrane protein 150B [Source:HGNC Symbol;Acc:HGNC:34415]	0.62
PMCTP1-013	2	ENSG00000169136	ATF5	activating transcription factor 5 [Source:HGNC Symbol;Acc:HGNC:790]	0.74
PMCTP1-013	2	ENSG00000185339	TCN2	transcobalamin 2 [Source:HGNC Symbol;Acc:HGNC:11653]	0.93

Cluster ID	Cluster Size	Gene ID	Gene Name	Gene Description	Log_2 FC TIV
PMCTP1-014	2	ENSG00000103569	AQP9	aquaporin 9 [Source:HGNC Symbol;Acc:HGNC:643]	0.58
PMCTP1-014	2	ENSG00000204388	HSPA1B	heat shock protein family A (Hsp70) member 1B [Source:HGNC Symbol;Acc:HGNC:5233]	0.67
PMCTP1-015	2	ENSG00000134326	CMPK2	cytidine/uridine monophosphate kinase 2 [Source:HGNC Symbol;Acc:HGNC:27015]	0.66
PMCTP1-015	2	ENSG00000158714	SLAMF8	SLAM family member 8 [Source:HGNC Symbol;Acc:HGNC:21391]	0.82
PMCTP1-016	3	ENSG00000165092	ALDH1A1	aldehyde dehydrogenase 1 family member A1 [Source:HGNC Symbol;Acc:HGNC:402]	0.66
PMCTP1-016	3	ENSG00000127951	FGL2	fibrinogen like 2 [Source:HGNC Symbol;Acc:HGNC:3696]	0.69
PMCTP1-016	3	ENSG00000005381	MPO	myeloperoxidase [Source:HGNC Symbol;Acc:HGNC:7218]	0.40
PMCTP1-017	3	ENSG00000143382	ADAMTSL4	ADAMTS like 4 [Source:HGNC Symbol;Acc:HGNC:19706]	0.58
PMCTP1-017	3	ENSG00000170458	CD14	CD14 molecule [Source:HGNC Symbol;Acc:HGNC:1628]	0.62
PMCTP1-017	3	ENSG00000226091	LINC00937	long intergenic non-protein coding RNA 937 [Source:HGNC Symbol;Acc:HGNC:48629]	0.62
PMCTP1-018	2	ENSG00000134755	DSC2	desmocollin 2 [Source:HGNC Symbol;Acc:HGNC:3036]	0.74
PMCTP1-018	2	ENSG00000113494	PRLR	prolactin receptor [Source:HGNC Symbol;Acc:HGNC:9446]	0.77
PMCTP1-019	4	ENSG00000260401			0.81
PMCTP1-019	4	ENSG00000019169	MARCO	macrophage receptor with collagenous structure [Source:HGNC Symbol;Acc:HGNC:6895]	0.71
PMCTP1-019	4	ENSG00000213694	S1PR3	sphingosine-1-phosphate receptor 3 [Source:HGNC Symbol;Acc:HGNC:3167]	0.68
PMCTP1-019	4	ENSG00000174705	SH3PXD2B	SH3 and PX domains 2B [Source:HGNC Symbol;Acc:HGNC:29242]	1.23
PMCTP1-020	2	ENSG00000165178	NCF1C	neutrophil cytosolic factor 1C pseudogene [Source:HGNC Symbol;Acc:HGNC:32523]	0.79
PMCTP1-020	2	ENSG00000114853	ZBTB47	zinc finger and BTB domain containing 47 [Source:HGNC Symbol;Acc:HGNC:26955]	0.59
PMCTP1-021	2	ENSG00000154146	NRGN	neurogranin [Source:HGNC Symbol;Acc:HGNC:8000]	0.39
PMCTP1-021	2	ENSG00000088826	SMOX	spermine oxidase [Source:HGNC Symbol;Acc:HGNC:15862]	0.55
PMCTP1-022	3	ENSG00000248996			0.64
PMCTP1-022	3	ENSG00000178445	GLDC	glycine decarboxylase [Source:HGNC Symbol;Acc:HGNC:4313]	0.49
PMCTP1-022	3	ENSG00000157227	MMP14	matrix metallopeptidase 14 [Source:HGNC Symbol;Acc:HGNC:7160]	0.76
PMCTP1-023	4	ENSG00000177989	ODF3B	outer dense fiber of sperm tails 3B [Source:HGNC Symbol;Acc:HGNC:34388]	1.16
PMCTP1-023	4	ENSG00000088827	SIGLEC1	sialic acid binding Ig like lectin 1 [Source:HGNC Symbol;Acc:HGNC:11127]	0.80
PMCTP1-023	4	ENSG00000197208	SLC22A4	solute carrier family 22 member 4 [Source:HGNC Symbol;Acc:HGNC:10968]	0.58
PMCTP1-023	4	ENSG00000185215	TNFAIP2	TNF alpha induced protein 2 [Source:HGNC Symbol;Acc:HGNC:11895]	0.63
PMCTP1-024	5	ENSG00000136630	HLX	H2.0 like homeobox [Source:HGNC Symbol;Acc:HGNC:4978]	0.59
PMCTP1-024	5	ENSG00000173110	HSPA6	heat shock protein family A (Hsp70) member 6 [Source:HGNC Symbol;Acc:HGNC:5239]	0.70
PMCTP1-024	5	ENSG00000142089	IFITM3	interferon induced transmembrane protein 3 [Source:HGNC Symbol;Acc:HGNC:5414]	0.58
PMCTP1-024	5	ENSG00000187608	ISG15	ISG15 ubiquitin-like modifier [Source:HGNC Symbol;Acc:HGNC:4053]	0.68
PMCTP1-024	5	ENSG00000197122	SRC	SRC proto-oncogene, non-receptor tyrosine kinase [Source:HGNC Symbol;Acc:HGNC:11283]	0.67
PMCTP1-025	3	ENSG00000229754	CXCR2P1	C-X-C motif chemokine receptor 2 pseudogene 1 [Source:HGNC Symbol;Acc:HGNC:6028]	0.63
PMCTP1-025	3	ENSG00000225492	GBP1P1	guanylate binding protein 1 pseudogene 1 [Source:HGNC Symbol;Acc:HGNC:39561]	1.19
PMCTP1-025	3	ENSG00000211679	IGLC3	immunoglobulin lambda constant 3 (Kern-Oz+ marker) [Source:HGNC Symbol;Acc:HGNC:5857]	0.11
PMCTP1-026	6	ENSG00000169245	CXCL10	C-X-C motif chemokine ligand 10 [Source:HGNC Symbol;Acc:HGNC:10637]	1.31
PMCTP1-026	6	ENSG00000117228	GBP1	guanylate binding protein 1 [Source:HGNC Symbol;Acc:HGNC:4182]	0.93

Cluster ID	Cluster Size	Gene ID	Gene Name	Gene Description	Log_2 FC TIV
PMCTP1-026	6	ENSG00000185745	IFIT1	interferon induced protein with tetratricopeptide repeats 1 [Source:HGNC Symbol;Acc:HGNC:5407]	0.61
PMCTP1-026	6	ENSG00000119922	IFIT2	interferon induced protein with tetratricopeptide repeats 2 [Source:HGNC Symbol;Acc:HGNC:5409]	0.91
PMCTP1-026	6	ENSG00000119917	IFIT3	interferon induced protein with tetratricopeptide repeats 3 [Source:HGNC Symbol;Acc:HGNC:5411]	0.83
PMCTP1-026	6	ENSG00000121858	TNFSF10	tumor necrosis factor superfamily member 10 [Source:HGNC Symbol;Acc:HGNC:11925]	0.68
PMCTP1-027	2	ENSG00000166527	CLEC4D	C-type lectin domain family 4 member D [Source:HGNC Symbol;Acc:HGNC:14554]	0.65
PMCTP1-027	2	ENSG00000164023	SGMS2	sphingomyelin synthase 2 [Source:HGNC Symbol;Acc:HGNC:28395]	0.63
PMCTP1-028	5	ENSG00000128383	APOBEC3A	apolipoprotein B mRNA editing enzyme catalytic subunit 3A [Source:HGNC Symbol;Acc:HGNC:17343]	0.70
PMCTP1-028	5	ENSG00000198814	GK	glycerol kinase [Source:HGNC Symbol;Acc:HGNC:4289]	0.66
PMCTP1-028	5	ENSG00000125538	IL1B	interleukin 1 beta [Source:HGNC Symbol;Acc:HGNC:5992]	0.93
PMCTP1-028	5	ENSG00000020577	SAMD4A	sterile alpha motif domain containing 4A [Source:HGNC Symbol;Acc:HGNC:23023]	0.80
PMCTP1-028	5	ENSG00000254415	SIGLEC14	sialic acid binding Ig like lectin 14 [Source:HGNC Symbol;Acc:HGNC:32926]	0.61
PMCTP1-029	2	ENSG00000250138			0.73
PMCTP1-029	2	ENSG00000211946	IGHV3-20	immunoglobulin heavy variable 3-20 [Source:HGNC Symbol;Acc:HGNC:5585]	0.70
PMCTP1-030	9	ENSG00000272821			1.20
PMCTP1-030	9	ENSG00000198019	FCGR1B	Fc fragment of IgG receptor Ib [Source:HGNC Symbol;Acc:HGNC:3614]	1.54
PMCTP1-030	9	ENSG00000205730	ITPRIPL2	inositol 1,4,5-trisphosphate receptor interacting protein like 2 [Source:HGNC Symbol;Acc:HGNC:27257]	0.61
PMCTP1-030	9	ENSG00000183762	KREMEN1	kringle containing transmembrane protein 1 [Source:HGNC Symbol;Acc:HGNC:17550]	1.41
PMCTP1-030	9	ENSG00000198736	MSRB1	methionine sulfoxide reductase B1 [Source:HGNC Symbol;Acc:HGNC:14133]	0.58
PMCTP1-030	9	ENSG00000182487	NCF1B	neutrophil cytosolic factor 1B pseudogene [Source:HGNC Symbol;Acc:HGNC:32522]	0.86
PMCTP1-030	9	ENSG00000235568	NFAM1	NFAT activating protein with ITAM motif 1 [Source:HGNC Symbol;Acc:HGNC:29872]	0.67
PMCTP1-030	9	ENSG00000130489	SCO2	SCO2, cytochrome c oxidase assembly protein [Source:HGNC Symbol;Acc:HGNC:10604]	1.02
PMCTP1-030	9	ENSG00000025708	TYMP	thymidine phosphorylase [Source:HGNC Symbol;Acc:HGNC:3148]	1.01
PMCTP1-031	2	ENSG00000188820	FAM26F	family with sequence similarity 26 member F [Source:HGNC Symbol;Acc:HGNC:33391]	0.62
PMCTP1-031	2	ENSG00000171241	SHCBP1	SHC binding and spindle associated 1 [Source:HGNC Symbol;Acc:HGNC:29547]	0.16
PMCTP1-032	4	ENSG00000100336	APOL4	apolipoprotein L4 [Source:HGNC Symbol;Acc:HGNC:14867]	2.03
PMCTP1-032	4	ENSG00000158517	NCF1	neutrophil cytosolic factor 1 [Source:HGNC Symbol;Acc:HGNC:7660]	0.78
PMCTP1-032	4	ENSG00000143878	RHOB	ras homolog family member B [Source:HGNC Symbol;Acc:HGNC:668]	0.26
PMCTP1-032	4	ENSG00000232810	TNF	tumor necrosis factor [Source:HGNC Symbol;Acc:HGNC:11892]	0.77
PMCTP1-033	10	ENSG00000163823	CCR1	C-C motif chemokine receptor 1 [Source:HGNC Symbol;Acc:HGNC:1602]	0.65
PMCTP1-033	10	ENSG00000186407	CD300E	CD300e molecule [Source:HGNC Symbol;Acc:HGNC:28874]	0.60
PMCTP1-033	10	ENSG00000146592	CREB5	cAMP responsive element binding protein 5 [Source:HGNC Symbol;Acc:HGNC:16844]	0.60
PMCTP1-033	10	ENSG00000139318	DUSP6	dual specificity phosphatase 6 [Source:HGNC Symbol;Acc:HGNC:3072]	0.63
PMCTP1-033	10	ENSG00000135636	DYSF	dysferlin [Source:HGNC Symbol;Acc:HGNC:3097]	0.81
PMCTP1-033	10	ENSG00000143226	FCGR2A	Fc fragment of IgG receptor IIa [Source:HGNC Symbol;Acc:HGNC:3616]	0.64

Cluster ID	Cluster Size	Gene ID	Gene Name	Gene Description	Log_2 FC TIV
PMCTP1-033	10	ENSG00000171051	FPR1	formyl peptide receptor 1 [Source:HGNC Symbol;Acc:HGNC:3826]	0.63
PMCTP1-033	10	ENSG00000103313	MEFV	Mediterranean fever [Source:HGNC Symbol;Acc:HGNC:6998]	0.63
PMCTP1-033	10	ENSG00000162512	SDC3	syndecan 3 [Source:HGNC Symbol;Acc:HGNC:10660]	0.96
PMCTP1-033	10	ENSG00000105967	TFEC	transcription factor EC [Source:HGNC Symbol;Acc:HGNC:11754]	0.64
PMCTP1-034	3	ENSG00000275302	CCL4	C-C motif chemokine ligand 4 [Source:HGNC Symbol;Acc:HGNC:10630]	-0.46
PMCTP1-034	3	ENSG00000197057	DTHD1	death domain containing 1 [Source:HGNC Symbol;Acc:HGNC:37261]	-0.62
PMCTP1-034	3	ENSG00000211892	IGHG4	immunoglobulin heavy constant gamma 4 (G4m marker) [Source:HGNC Symbol;Acc:HGNC:5528]	-0.32
PMCTP1-035	2	ENSG00000205846	CLEC6A	C-type lectin domain family 6 member A [Source:HGNC Symbol;Acc:HGNC:14556]	1.21
PMCTP1-035	2	ENSG00000162614	NEXN	nexilin F-actin binding protein [Source:HGNC Symbol;Acc:HGNC:29557]	0.78
PMCTP1-036	4	ENSG00000120885	CLU	clusterin [Source:HGNC Symbol;Acc:HGNC:2095]	0.34
PMCTP1-036	4	ENSG00000149564	ESAM	endothelial cell adhesion molecule [Source:HGNC Symbol;Acc:HGNC:17474]	0.20
PMCTP1-036	4	ENSG00000101335	MYL9	myosin light chain 9 [Source:HGNC Symbol;Acc:HGNC:15754]	0.57
PMCTP1-036	4	ENSG00000163737	PF4	platelet factor 4 [Source:HGNC Symbol;Acc:HGNC:8861]	0.39
PMCTP1-037	6	ENSG00000110318	CEP126	centrosomal protein 126 [Source:HGNC Symbol;Acc:HGNC:29264]	-0.62
PMCTP1-037	6	ENSG00000187118	CMC1	C-X9-C motif containing 1 [Source:HGNC Symbol;Acc:HGNC:28783]	-0.58
PMCTP1-037	6	ENSG00000168209	DDIT4	DNA damage inducible transcript 4 [Source:HGNC Symbol;Acc:HGNC:24944]	-0.50
PMCTP1-037	6	ENSG00000171451	DSEL	dermatan sulfate epimerase-like [Source:HGNC Symbol;Acc:HGNC:18144]	-0.79
PMCTP1-037	6	ENSG00000167633	KIR3DL1	killer cell immunoglobulin like receptor, three Ig domains and long cytoplasmic tail 1 [Source:HGNC Symbol;Acc:HGNC:6338]	-0.62
PMCTP1-037	6	ENSG00000255819	KLRC4-KLRK1	KLRC4-KLRK1 readthrough [Source:HGNC Symbol;Acc:HGNC:48357]	-0.73
PMCTP1-038	4	ENSG00000128283	CDC42EP1	CDC42 effector protein 1 [Source:HGNC Symbol;Acc:HGNC:17014]	0.61
PMCTP1-038	4	ENSG00000180340	FZD2	frizzled class receptor 2 [Source:HGNC Symbol;Acc:HGNC:4040]	0.67
PMCTP1-038	4	ENSG00000101057	MYBL2	MYB proto-oncogene like 2 [Source:HGNC Symbol;Acc:HGNC:7548]	0.24
PMCTP1-038	4	ENSG00000242732	RGAG4	retrotransposon gag domain containing 4 [Source:HGNC Symbol;Acc:HGNC:29430]	0.65
PMCTP1-039	3	ENSG00000271109			-0.65
PMCTP1-039	3	ENSG00000280411	IGHV1-69-2	immunoglobulin heavy variable 1-69-2 [Source:HGNC Symbol;Acc:HGNC:5562]	-0.56
PMCTP1-039	3	ENSG00000197705	KLHL14	kelch like family member 14 [Source:HGNC Symbol;Acc:HGNC:29266]	-0.31
PMCTP1-040	6	ENSG00000197561	ELANE	elastase, neutrophil expressed [Source:HGNC Symbol;Acc:HGNC:3309]	0.45
PMCTP1-040	6	ENSG00000211967	IGHV3-53	immunoglobulin heavy variable 3-53 [Source:HGNC Symbol;Acc:HGNC:5610]	0.40
PMCTP1-040	6	ENSG00000242076	IGKV1-33	immunoglobulin kappa variable 1-33 [Source:HGNC Symbol;Acc:HGNC:5737]	0.86
PMCTP1-040	6	ENSG00000253818	IGLV1-41	immunoglobulin lambda variable 1-41 (pseudogene) [Source:HGNC Symbol;Acc:HGNC:5878]	0.84
PMCTP1-040	6	ENSG00000211659	IGLV3-25	immunoglobulin lambda variable 3-25 [Source:HGNC Symbol;Acc:HGNC:5908]	0.44
PMCTP1-040	6	ENSG00000268849	SIGLEC22P	sialic acid binding Ig like lectin 22, pseudogene [Source:HGNC Symbol;Acc:HGNC:15611]	0.78

Cluster ID	Cluster Size	Gene ID	Gene Name	Gene Description	Log_2 FC TIV
PMCTP1-041	5	ENSG00000276070	CCL4L2	C-C motif chemokine ligand 4 like 2 [Source:HGNC Symbol;Acc:HGNC:24066]	-0.76
PMCTP1-041	5	ENSG00000117281	CD160	CD160 molecule [Source:HGNC Symbol;Acc:HGNC:17013]	-0.61
PMCTP1-041	5	ENSG00000149557	FEZ1	fasciculation and elongation protein zeta 1 [Source:HGNC Symbol;Acc:HGNC:3659]	-0.93
PMCTP1-041	5	ENSG00000137441	FGFBP2	fibroblast growth factor binding protein 2 [Source:HGNC Symbol;Acc:HGNC:29451]	-0.66
PMCTP1-041	5	ENSG00000150687	PRSS23	protease, serine 23 [Source:HGNC Symbol;Acc:HGNC:14370]	-0.62
PMCTP1-042	3	ENSG00000133048	CHI3L1	chitinase 3 like 1 [Source:HGNC Symbol;Acc:HGNC:1932]	-0.16
PMCTP1-042	3	ENSG00000123689	G0S2	G0/G1 switch 2 [Source:HGNC Symbol;Acc:HGNC:30229]	-1.65
PMCTP1-042	3	ENSG00000186049	KRT73	keratin 73 [Source:HGNC Symbol;Acc:HGNC:28928]	-0.69
PMCTP1-043	4	ENSG00000177191	B3GNT8	UDP-GlcNAc:betaGal beta-1,3-N-acetylglucosaminyltransferase 8 [Source:HGNC Symbol;Acc:HGNC:24139]	0.42
PMCTP1-043	4	ENSG00000187840	EIF4EBP1	eukaryotic translation initiation factor 4E binding protein 1 [Source:HGNC Symbol;Acc:HGNC:3288]	0.63
PMCTP1-043	4	ENSG00000211972	IGHV3-66	immunoglobulin heavy variable 3-66 [Source:HGNC Symbol;Acc:HGNC:5619]	0.35
PMCTP1-043	4	ENSG00000177301	KCNA2	potassium voltage-gated channel subfamily A member 2 [Source:HGNC Symbol;Acc:HGNC:6220]	0.40

Table 38: Co-expressed gene clusters (PBMC, Day 1)

Cluster ID	Cluster Size	Gene ID	Gene Name	Gene Description	Log_2 FC TIV
PMCTP2-001	2	ENSG00000156265	MAP3K7CL	MAP3K7 C-terminal like [Source:HGNC Symbol;Acc:HGNC:16457]	0.89
PMCTP2-001	2	ENSG00000187800	PEAR1	platelet endothelial aggregation receptor 1 [Source:HGNC Symbol;Acc:HGNC:33631]	0.71
PMCTP2-002	2	ENSG00000151023	ENKUR	enkurin, TRPC channel interacting protein [Source:HGNC Symbol;Acc:HGNC:28388]	0.83
PMCTP2-002	2	ENSG00000082781	ITGB5	integrin subunit beta 5 [Source:HGNC Symbol;Acc:HGNC:6160]	0.88
PMCTP2-003	2	ENSG00000005381	MPO	myeloperoxidase [Source:HGNC Symbol;Acc:HGNC:7218]	0.53
PMCTP2-003	2	ENSG00000171611	PTCRA	pre T-cell antigen receptor alpha [Source:HGNC Symbol;Acc:HGNC:21290]	0.99
PMCTP2-004	2	ENSG00000180573	HIST1H2AC	histone cluster 1 H2A family member c [Source:HGNC Symbol;Acc:HGNC:4733]	0.86
PMCTP2-004	2	ENSG00000108960	MMD	monocyte to macrophage differentiation associated [Source:HGNC Symbol;Acc:HGNC:7153]	0.62
PMCTP2-005	3	ENSG00000185745	IFIT1	interferon induced protein with tetratricopeptide repeats 1 [Source:HGNC Symbol;Acc:HGNC:5407]	0.63
PMCTP2-005	3	ENSG00000119922	IFIT2	interferon induced protein with tetratricopeptide repeats 2 [Source:HGNC Symbol;Acc:HGNC:5409]	0.80
PMCTP2-005	3	ENSG00000119917	IFIT3	interferon induced protein with tetratricopeptide repeats 3 [Source:HGNC Symbol;Acc:HGNC:5411]	0.69
PMCTP2-006	2	ENSG00000173210	ABLIM3	actin binding LIM protein family member 3 [Source:HGNC Symbol;Acc:HGNC:29132]	0.82
PMCTP2-006	2	ENSG00000163735	CXCL5	C-X-C motif chemokine ligand 5 [Source:HGNC Symbol;Acc:HGNC:10642]	0.91
PMCTP2-007	2	ENSG00000177324	BEND2	BEN domain containing 2 [Source:HGNC Symbol;Acc:HGNC:28509]	1.27
PMCTP2-007	2	ENSG00000085733	CTTN	cortactin [Source:HGNC Symbol;Acc:HGNC:3338]	0.93
PMCTP2-008	2	ENSG00000205309	NT5M	5',3'-nucleotidase, mitochondrial [Source:HGNC Symbol;Acc:HGNC:15769]	0.85
PMCTP2-008	2	ENSG00000011105	TSPAN9	tetraspanin 9 [Source:HGNC Symbol;Acc:HGNC:21640]	0.62
PMCTP2-009	2	ENSG00000150337	FCGR1A	Fc fragment of IgG receptor Ia [Source:HGNC Symbol;Acc:HGNC:3613]	0.73
PMCTP2-009	2	ENSG00000173868	PHOSPHO1	phosphoethanolamine/phosphocholine phosphatase [Source:HGNC Symbol;Acc:HGNC:16815]	0.54

Cluster ID	Cluster Size	Gene ID	Gene Name	Gene Description	Log_2 FC TIV
PMCTP2-010	2	ENSG00000187699	C2orf88	chromosome 2 open reading frame 88 [Source:HGNC Symbol;Acc:HGNC:28191]	0.71
PMCTP2-010	2	ENSG00000065534	MYLK	myosin light chain kinase [Source:HGNC Symbol;Acc:HGNC:7590]	0.68
PMCTP2-011	2	ENSG00000278828	HIST1H3H	histone cluster 1 H3 family member h [Source:HGNC Symbol;Acc:HGNC:4775]	0.88
PMCTP2-011	2	ENSG00000184678	HIST2H2BE	histone cluster 2 H2B family member e [Source:HGNC Symbol;Acc:HGNC:4760]	0.82
PMCTP2-012	3	ENSG00000173110	HSPA6	heat shock protein family A (Hsp70) member 6 [Source:HGNC Symbol;Acc:HGNC:5239]	0.59
PMCTP2-012	3	ENSG00000142089	IFITM3	interferon induced transmembrane protein 3 [Source:HGNC Symbol;Acc:HGNC:5414]	0.62
PMCTP2-012	3	ENSG00000125538	IL1B	interleukin 1 beta [Source:HGNC Symbol;Acc:HGNC:5992]	0.57
PMCTP2-013	7	ENSG00000254614			0.63
PMCTP2-013	7	ENSG00000111644	ACRBP	acrosin binding protein [Source:HGNC Symbol;Acc:HGNC:17195]	0.68
PMCTP2-013	7	ENSG00000120885	CLU	clusterin [Source:HGNC Symbol;Acc:HGNC:2095]	0.94
PMCTP2-013	7	ENSG00000166091	CMTM5	CKLF like MARVEL transmembrane domain containing 5 [Source:HGNC Symbol;Acc:HGNC:19176]	0.76
PMCTP2-013	7	ENSG00000163737	PF4	platelet factor 4 [Source:HGNC Symbol;Acc:HGNC:8861]	0.94
PMCTP2-013	7	ENSG00000113140	SPARC	secreted protein acidic and cysteine rich [Source:HGNC Symbol;Acc:HGNC:11219]	0.89
PMCTP2-013	7	ENSG00000101162	TUBB1	tubulin beta 1 class VI [Source:HGNC Symbol;Acc:HGNC:16257]	0.92
PMCTP2-014	2	ENSG00000272821			0.72
PMCTP2-014	2	ENSG00000004799	PDK4	pyruvate dehydrogenase kinase 4 [Source:HGNC Symbol;Acc:HGNC:8812]	1.19
PMCTP2-015	4	ENSG00000127920	GNG11	G protein subunit gamma 11 [Source:HGNC Symbol;Acc:HGNC:4403]	0.73
PMCTP2-015	4	ENSG00000198948	MFAP3L	microfibrillar associated protein 3 like [Source:HGNC Symbol;Acc:HGNC:29083]	0.59
PMCTP2-015	4	ENSG00000163736	PPBP	pro-platelet basic protein [Source:HGNC Symbol;Acc:HGNC:9240]	0.86
PMCTP2-015	4	ENSG00000005249	PRKAR2B	protein kinase cAMP-dependent type II regulatory subunit beta [Source:HGNC Symbol;Acc:HGNC:9392]	0.70
PMCTP2-016	2	ENSG00000170458	CD14	CD14 molecule [Source:HGNC Symbol;Acc:HGNC:1628]	0.29
PMCTP2-016	2	ENSG00000177989	ODF3B	outer dense fiber of sperm tails 3B [Source:HGNC Symbol;Acc:HGNC:34388]	0.69
PMCTP2-017	2	ENSG00000229754	CXCR2P1	C-X-C motif chemokine receptor 2 pseudogene 1 [Source:HGNC Symbol;Acc:HGNC:6028]	0.72
PMCTP2-017	2	ENSG00000143878	RHOB	ras homolog family member B [Source:HGNC Symbol;Acc:HGNC:668]	0.65
PMCTP2-018	3	ENSG00000198814	GK	glycerol kinase [Source:HGNC Symbol;Acc:HGNC:4289]	0.31
PMCTP2-018	3	ENSG00000149131	SERPING1	serpin family G member 1 [Source:HGNC Symbol;Acc:HGNC:1228]	1.06
PMCTP2-018	3	ENSG00000156587	UBE2L6	ubiquitin conjugating enzyme E2 L6 [Source:HGNC Symbol;Acc:HGNC:12490]	0.30
PMCTP2-019	3	ENSG00000005961	ITGA2B	integrin subunit alpha 2b [Source:HGNC Symbol;Acc:HGNC:6138]	0.91
PMCTP2-019	3	ENSG00000101335	MYL9	myosin light chain 9 [Source:HGNC Symbol;Acc:HGNC:15754]	1.17
PMCTP2-019	3	ENSG00000161911	TREML1	triggering receptor expressed on myeloid cells like 1 [Source:HGNC Symbol;Acc:HGNC:20434]	0.94
PMCTP2-020	5	ENSG00000165092	ALDH1A1	aldehyde dehydrogenase 1 family member A1 [Source:HGNC Symbol;Acc:HGNC:402]	0.35
PMCTP2-020	5	ENSG00000127951	FGL2	fibrinogen like 2 [Source:HGNC Symbol;Acc:HGNC:3696]	0.34
PMCTP2-020	5	ENSG00000188906	LRRK2	leucine rich repeat kinase 2 [Source:HGNC Symbol;Acc:HGNC:18618]	0.40
PMCTP2-020	5	ENSG00000125148	MT2A	metallothionein 2A [Source:HGNC Symbol;Acc:HGNC:7406]	0.63
PMCTP2-020	5	ENSG00000115415	STAT1	signal transducer and activator of transcription 1 [Source:HGNC Symbol;Acc:HGNC:11362]	0.37

Cluster ID	Cluster Size	Gene ID	Gene Name	Gene Description	Log_2 FC TIV
PMCTP2-021	2	ENSG00000140479	PCSK6	proprotein convertase subtilisin/kexin type 6 [Source:HGNC Symbol;Acc:HGNC:8569]	0.76
PMCTP2-021	2	ENSG00000130489	SCO2	SCO2, cytochrome c oxidase assembly protein [Source:HGNC Symbol;Acc:HGNC:10604]	0.73
PMCTP2-022	3	ENSG00000100336	APOL4	apolipoprotein L4 [Source:HGNC Symbol;Acc:HGNC:14867]	1.03
PMCTP2-022	3	ENSG00000169245	CXCL10	C-X-C motif chemokine ligand 10 [Source:HGNC Symbol;Acc:HGNC:10637]	0.82
PMCTP2-022	3	ENSG00000143226	FCGR2A	Fc fragment of IgG receptor IIa [Source:HGNC Symbol;Acc:HGNC:3616]	0.48
PMCTP2-023	3	ENSG00000211653	IGLV1-40	immunoglobulin lambda variable 1-40 [Source:HGNC Symbol;Acc:HGNC:5877]	0.20
PMCTP2-023	3	ENSG00000154447	SH3RF1	SH3 domain containing ring finger 1 [Source:HGNC Symbol;Acc:HGNC:17650]	0.62
PMCTP2-023	3	ENSG00000183779	ZNF703	zinc finger protein 703 [Source:HGNC Symbol;Acc:HGNC:25883]	0.65
PMCTP2-024	3	ENSG00000196787	HIST1H2AG	histone cluster 1 H2A family member g [Source:HGNC Symbol;Acc:HGNC:4737]	0.89
PMCTP2-024	3	ENSG00000154146	NRGN	neurogranin [Source:HGNC Symbol;Acc:HGNC:8000]	0.84
PMCTP2-024	3	ENSG00000184792	OSBP2	oxysterol binding protein 2 [Source:HGNC Symbol;Acc:HGNC:8504]	0.51
PMCTP2-025	2	ENSG00000236304			1.07
PMCTP2-025	2	ENSG00000198478	SH3BGRL2	SH3 domain binding glutamate rich protein like 2 [Source:HGNC Symbol;Acc:HGNC:15567]	0.73
PMCTP2-026	3	ENSG00000168062	BATF2	basic leucine zipper ATF-like transcription factor 2 [Source:HGNC Symbol;Acc:HGNC:25163]	0.88
PMCTP2-026	3	ENSG00000133106	EPSTI1	epithelial stromal interaction 1 [Source:HGNC Symbol;Acc:HGNC:16465]	0.26
PMCTP2-026	3	ENSG00000138496	PARP9	poly(ADP-ribose) polymerase family member 9 [Source:HGNC Symbol;Acc:HGNC:24118]	0.28
PMCTP2-027	5	ENSG00000188305	C19orf35	chromosome 19 open reading frame 35 [Source:HGNC Symbol;Acc:HGNC:24793]	0.59
PMCTP2-027	5	ENSG00000103313	MEFV	Mediterranean fever [Source:HGNC Symbol;Acc:HGNC:6998]	0.45
PMCTP2-027	5	ENSG00000135604	STX11	syntaxin 11 [Source:HGNC Symbol;Acc:HGNC:11429]	0.42
PMCTP2-027	5	ENSG00000165914	TTC7B	tetratricopeptide repeat domain 7B [Source:HGNC Symbol;Acc:HGNC:19858]	0.68
PMCTP2-027	5	ENSG00000140105	WARS	tryptophanyl-tRNA synthetase [Source:HGNC Symbol;Acc:HGNC:12729]	0.41
PMCTP2-028	3	ENSG00000204420	C6orf25	chromosome 6 open reading frame 25 [Source:HGNC Symbol;Acc:HGNC:13937]	0.52
PMCTP2-028	3	ENSG00000123689	G0S2	G0/G1 switch 2 [Source:HGNC Symbol;Acc:HGNC:30229]	0.51
PMCTP2-028	3	ENSG00000250334	LINC00989	long intergenic non-protein coding RNA 989 [Source:HGNC Symbol;Acc:HGNC:48918]	0.74
PMCTP2-029	2	ENSG00000164181	ELOVL7	ELOVL fatty acid elongase 7 [Source:HGNC Symbol;Acc:HGNC:26292]	0.71
PMCTP2-029	2	ENSG00000105967	TFEC	transcription factor EC [Source:HGNC Symbol;Acc:HGNC:11754]	0.25
PMCTP2-030	3	ENSG00000137198	GMPR	guanosine monophosphate reductase [Source:HGNC Symbol;Acc:HGNC:4376]	0.83
PMCTP2-030	3	ENSG00000259207	ITGB3	integrin subunit beta 3 [Source:HGNC Symbol;Acc:HGNC:6156]	0.67
PMCTP2-030	3	ENSG00000168497	SDPR	serum deprivation response [Source:HGNC Symbol;Acc:HGNC:10690]	0.86
PMCTP2-031	3	ENSG00000187840	EIF4EBP1	eukaryotic translation initiation factor 4E binding protein 1 [Source:HGNC Symbol;Acc:HGNC:3288]	0.51
PMCTP2-031	3	ENSG00000185340	GAS2L1	growth arrest specific 2 like 1 [Source:HGNC Symbol;Acc:HGNC:16955]	0.63
PMCTP2-031	3	ENSG00000101057	MYBL2	MYB proto-oncogene like 2 [Source:HGNC Symbol;Acc:HGNC:7548]	0.37
PMCTP2-032	3	ENSG00000073737	DHRS9	dehydrogenase/reductase 9 [Source:HGNC Symbol;Acc:HGNC:16888]	0.63

Cluster ID	Cluster Size	Gene ID	Gene Name	Gene Description	Log_2 FC TIV
PMCTP2-032	3	ENSG00000171223	JUNB	JunB proto-oncogene, AP-1 transcription factor subunit [Source:HGNC Symbol;Acc:HGNC:6205]	0.63
PMCTP2-032	3	ENSG00000074416	MGLL	monoglyceride lipase [Source:HGNC Symbol;Acc:HGNC:17038]	0.62
PMCTP2-033	4	ENSG00000163430	FSTL1	follistatin like 1 [Source:HGNC Symbol;Acc:HGNC:3972]	1.15
PMCTP2-033	4	ENSG00000137959	IFI44L	interferon induced protein 44 like [Source:HGNC Symbol;Acc:HGNC:17817]	0.44
PMCTP2-033	4	ENSG00000143995	MEIS1	Meis homeobox 1 [Source:HGNC Symbol;Acc:HGNC:7000]	0.67
PMCTP2-033	4	ENSG00000162614	NEXN	nexilin F-actin binding protein [Source:HGNC Symbol;Acc:HGNC:29557]	0.33
PMCTP2-034	7	ENSG00000260401			0.48
PMCTP2-034	7	ENSG00000143382	ADAMTSL4	ADAMTS like 4 [Source:HGNC Symbol;Acc:HGNC:19706]	0.46
PMCTP2-034	7	ENSG00000186407	CD300E	CD300e molecule [Source:HGNC Symbol;Acc:HGNC:28874]	0.45
PMCTP2-034	7	ENSG00000019169	MARCO	macrophage receptor with collagenous structure [Source:HGNC Symbol;Acc:HGNC:6895]	0.56
PMCTP2-034	7	ENSG00000020577	SAMD4A	sterile alpha motif domain containing 4A [Source:HGNC Symbol;Acc:HGNC:23023]	0.50
PMCTP2-034	7	ENSG00000088827	SIGLEC1	sialic acid binding Ig like lectin 1 [Source:HGNC Symbol;Acc:HGNC:11127]	0.49
PMCTP2-034	7	ENSG00000254415	SIGLEC14	sialic acid binding Ig like lectin 14 [Source:HGNC Symbol;Acc:HGNC:32926]	0.42
PMCTP2-035	5	ENSG00000187608	ISG15	ISG15 ubiquitin-like modifier [Source:HGNC Symbol;Acc:HGNC:4053]	0.48
PMCTP2-035	5	ENSG00000158517	NCF1	neutrophil cytosolic factor 1 [Source:HGNC Symbol;Acc:HGNC:7660]	0.51
PMCTP2-035	5	ENSG00000182487	NCF1B	neutrophil cytosolic factor 1B pseudogene [Source:HGNC Symbol;Acc:HGNC:32522]	0.63
PMCTP2-035	5	ENSG00000235568	NFAM1	NFAT activating protein with ITAM motif 1 [Source:HGNC Symbol;Acc:HGNC:29872]	0.42
PMCTP2-035	5	ENSG00000141574	SECTM1	secreted and transmembrane 1 [Source:HGNC Symbol;Acc:HGNC:10707]	0.66
PMCTP2-036	5	ENSG00000177191	B3GNT8	UDP-GlcNAc:betaGal beta-1,3-N-acetylglucosaminyltransferase 8 [Source:HGNC Symbol;Acc:HGNC:24139]	0.65
PMCTP2-036	5	ENSG00000149564	ESAM	endothelial cell adhesion molecule [Source:HGNC Symbol;Acc:HGNC:17474]	0.58
PMCTP2-036	5	ENSG00000165702	GFI1B	growth factor independent 1B transcriptional repressor [Source:HGNC Symbol;Acc:HGNC:4238]	0.75
PMCTP2-036	5	ENSG00000128266	GNAZ	G protein subunit alpha z [Source:HGNC Symbol;Acc:HGNC:4395]	0.70
PMCTP2-036	5	ENSG00000184702	SEPT5	septin 5 [Source:HGNC Symbol;Acc:HGNC:9164]	0.92
PMCTP2-037	4	ENSG00000250138			0.54
PMCTP2-037	4	ENSG00000149573	MPZL2	myelin protein zero like 2 [Source:HGNC Symbol;Acc:HGNC:3496]	0.29
PMCTP2-037	4	ENSG00000162366	PDZK1IP1	PDZK1 interacting protein 1 [Source:HGNC Symbol;Acc:HGNC:16887]	0.86
PMCTP2-037	4	ENSG00000146070	PLA2G7	phospholipase A2 group VII [Source:HGNC Symbol;Acc:HGNC:9040]	0.21

Table 39: Co-expressed gene clusters (PBMC, Day 2)

Cluster ID	Cluster Size	Gene ID	Gene Name	Gene Description	Log_2 FC TIV
PMCTP3-001	2	ENSG00000119922	IFIT2	interferon induced protein with tetratricopeptide repeats 2 [Source:HGNC Symbol;Acc:HGNC:5409]	0.63
PMCTP3-001	2	ENSG00000101162	TUBB1	tubulin beta 1 class VI [Source:HGNC Symbol;Acc:HGNC:16257]	0.43
PMCTP3-002	2	ENSG00000125347	IRF1	interferon regulatory factor 1 [Source:HGNC Symbol;Acc:HGNC:6116]	0.23

Cluster ID	Cluster Size	Gene ID	Gene Name	Gene Description	Log_2 FC TIV
PMCTP3-002	2	ENSG00000005961	ITGA2B	integrin subunit alpha 2b [Source:HGNC Symbol;Acc:HGNC:6138]	0.59
PMCTP3-003	2	ENSG00000260401			0.75
PMCTP3-003	2	ENSG00000141574	SECTM1	secreted and transmembrane 1 [Source:HGNC Symbol;Acc:HGNC:10707]	0.53
PMCTP3-004	2	ENSG00000150337	FCGR1A	Fc fragment of IgG receptor Ia [Source:HGNC Symbol;Acc:HGNC:3613]	0.35
PMCTP3-004	2	ENSG00000005381	MPO	myeloperoxidase [Source:HGNC Symbol;Acc:HGNC:7218]	0.59
PMCTP3-005	2	ENSG00000169136	ATF5	activating transcription factor 5 [Source:HGNC Symbol;Acc:HGNC:790]	0.40
PMCTP3-005	2	ENSG00000149131	SERPING1	serpin family G member 1 [Source:HGNC Symbol;Acc:HGNC:1228]	0.59
PMCTP3-006	4	ENSG00000082397	EPB41L3	erythrocyte membrane protein band 4.1 like 3 [Source:HGNC Symbol;Acc:HGNC:3380]	0.32
PMCTP3-006	4	ENSG00000188906	LRRK2	leucine rich repeat kinase 2 [Source:HGNC Symbol;Acc:HGNC:18618]	0.21
PMCTP3-006	4	ENSG00000113494	PRLR	prolactin receptor [Source:HGNC Symbol;Acc:HGNC:9446]	0.44
PMCTP3-006	4	ENSG00000154447	SH3RF1	SH3 domain containing ring finger 1 [Source:HGNC Symbol;Acc:HGNC:17650]	0.59
PMCTP3-007	2	ENSG00000162551	ALPL	alkaline phosphatase, liver/bone/kidney [Source:HGNC Symbol;Acc:HGNC:438]	0.58
PMCTP3-007	2	ENSG00000163993	S100P	S100 calcium binding protein P [Source:HGNC Symbol;Acc:HGNC:10504]	0.43
PMCTP3-008	2	ENSG00000143226	FCGR2A	Fc fragment of IgG receptor IIa [Source:HGNC Symbol;Acc:HGNC:3616]	0.36
PMCTP3-008	2	ENSG00000004799	PDK4	pyruvate dehydrogenase kinase 4 [Source:HGNC Symbol;Acc:HGNC:8812]	0.99
PMCTP3-009	4	ENSG00000062282	DGAT2	diacylglycerol O-acyltransferase 2 [Source:HGNC Symbol;Acc:HGNC:16940]	0.38
PMCTP3-009	4	ENSG00000197561	ELANE	elastase, neutrophil expressed [Source:HGNC Symbol;Acc:HGNC:3309]	0.98
PMCTP3-009	4	ENSG00000183762	KREMEN1	kringle containing transmembrane protein 1 [Source:HGNC Symbol;Acc:HGNC:17550]	0.79
PMCTP3-009	4	ENSG00000198736	MSRB1	methionine sulfoxide reductase B1 [Source:HGNC Symbol;Acc:HGNC:14133]	0.40
PMCTP3-010	4	ENSG00000111644	ACRBP	acrosin binding protein [Source:HGNC Symbol;Acc:HGNC:17195]	0.29
PMCTP3-010	4	ENSG00000211964	IGHV3-48	immunoglobulin heavy variable 3-48 [Source:HGNC Symbol;Acc:HGNC:5606]	0.15
PMCTP3-010	4	ENSG00000157551	KCNJ15	potassium voltage-gated channel subfamily J member 15 [Source:HGNC Symbol;Acc:HGNC:6261]	0.52
PMCTP3-010	4	ENSG00000123700	KCNJ2	potassium voltage-gated channel subfamily J member 2 [Source:HGNC Symbol;Acc:HGNC:6263]	0.69
PMCTP3-011	4	ENSG00000126262	FFAR2	free fatty acid receptor 2 [Source:HGNC Symbol;Acc:HGNC:4501]	0.73
PMCTP3-011	4	ENSG00000182782	HCAR2	hydroxycarboxylic acid receptor 2 [Source:HGNC Symbol;Acc:HGNC:24827]	0.78
PMCTP3-011	4	ENSG00000173868	PHOSPHO1	phosphoethanolamine/phosphocholine phosphatase [Source:HGNC Symbol;Acc:HGNC:16815]	0.40
PMCTP3-011	4	ENSG00000173535	TNFRSF10C	TNF receptor superfamily member 10c [Source:HGNC Symbol;Acc:HGNC:11906]	0.71
PMCTP3-012	6	ENSG00000248996			0.41
PMCTP3-012	6	ENSG00000135636	DYSF	dysferlin [Source:HGNC Symbol;Acc:HGNC:3097]	0.29
PMCTP3-012	6	ENSG00000136689	IL1RN	interleukin 1 receptor antagonist [Source:HGNC Symbol;Acc:HGNC:6000]	0.30
PMCTP3-012	6	ENSG00000088827	SIGLEC1	sialic acid binding Ig like lectin 1 [Source:HGNC Symbol;Acc:HGNC:11127]	0.62
PMCTP3-012	6	ENSG00000197122	SRC	SRC proto-oncogene, non-receptor tyrosine kinase [Source:HGNC Symbol;Acc:HGNC:11283]	0.38
PMCTP3-012	6	ENSG00000183779	ZNF703	zinc finger protein 703 [Source:HGNC Symbol;Acc:HGNC:25883]	0.57

Cluster ID	Cluster Size	Gene ID	Gene Name	Gene Description	Log_2 FC TIV
PMCTP3-013	5	ENSG00000204388	HSPA1B	heat shock protein family A (Hsp70) member 1B [Source:HGNC Symbol;Acc:HGNC:5233]	0.65
PMCTP3-013	5	ENSG00000271178	IGHV3OR16-13	immunoglobulin heavy variable 3/OR16-13 (non-functional) [Source:HGNC Symbol;Acc:HGNC:5637]	1.05
PMCTP3-013	5	ENSG00000125538	IL1B	interleukin 1 beta [Source:HGNC Symbol;Acc:HGNC:5992]	0.73
PMCTP3-013	5	ENSG00000143878	RHOB	ras homolog family member B [Source:HGNC Symbol;Acc:HGNC:668]	0.39
PMCTP3-013	5	ENSG00000232810	TNF	tumor necrosis factor [Source:HGNC Symbol;Acc:HGNC:11892]	0.66
PMCTP3-014	4	ENSG00000267481			-0.58
PMCTP3-014	4	ENSG00000110848	CD69	CD69 molecule [Source:HGNC Symbol;Acc:HGNC:1694]	-0.43
PMCTP3-014	4	ENSG00000187118	CMC1	C-X9-C motif containing 1 [Source:HGNC Symbol;Acc:HGNC:28783]	-0.49
PMCTP3-014	4	ENSG00000255819	KLRC4-KLRK1	KLRC4-KLRK1 readthrough [Source:HGNC Symbol;Acc:HGNC:48357]	-0.44

Table 40: Co-expressed gene clusters (PBMC, Day 3)

Cluster ID	Cluster Size	Gene ID	Gene Name	Gene Description	Log_2 FC TIV
PMCTP4-001	4	ENSG00000275302	CCL4	C-C motif chemokine ligand 4 [Source:HGNC Symbol;Acc:HGNC:10630]	-0.68
PMCTP4-001	4	ENSG00000137441	FGFBP2	fibroblast growth factor binding protein 2 [Source:HGNC Symbol;Acc:HGNC:29451]	-0.57
PMCTP4-001	4	ENSG00000150687	PRSS23	protease, serine 23 [Source:HGNC Symbol;Acc:HGNC:14370]	-0.73
PMCTP4-001	4	ENSG00000171101	SIGLEC17P	sialic acid binding Ig like lectin 17, pseudogene [Source:HGNC Symbol;Acc:HGNC:15604]	-0.41

Table 41: Co-expressed gene clusters (PBMC, Day 4)

Cluster ID	Cluster Size	Gene ID	Gene Name	Gene Description	Log_2 FC TIV
PMCTP5-001	2	ENSG00000254709	IGLL5	immunoglobulin lambda like polypeptide 5 [Source:HGNC Symbol;Acc:HGNC:38476]	1.14
PMCTP5-001	2	ENSG00000177301	KCNA2	potassium voltage-gated channel subfamily A member 2 [Source:HGNC Symbol;Acc:HGNC:6220]	0.61
PMCTP5-002	2	ENSG00000282639			0.92
PMCTP5-002	2	ENSG00000171848	RRM2	ribonucleotide reductase regulatory subunit M2 [Source:HGNC Symbol;Acc:HGNC:10452]	1.18
PMCTP5-003	4	ENSG00000243238	IGKV2-30	immunoglobulin kappa variable 2-30 [Source:HGNC Symbol;Acc:HGNC:5785]	1.49
PMCTP5-003	4	ENSG00000241351	IGKV3-11	immunoglobulin kappa variable 3-11 [Source:HGNC Symbol;Acc:HGNC:5815]	1.03
PMCTP5-003	4	ENSG00000170476	MZB1	marginal zone B and B1 cell specific protein [Source:HGNC Symbol;Acc:HGNC:30125]	1.19
PMCTP5-003	4	ENSG00000048462	TNFRSF17	TNF receptor superfamily member 17 [Source:HGNC Symbol;Acc:HGNC:11913]	1.48
PMCTP5-004	3	ENSG00000211946	IGHV3-20	immunoglobulin heavy variable 3-20 [Source:HGNC Symbol;Acc:HGNC:5585]	1.88
PMCTP5-004	3	ENSG00000211653	IGLV1-40	immunoglobulin lambda variable 1-40 [Source:HGNC Symbol;Acc:HGNC:5877]	1.71
PMCTP5-004	3	ENSG00000211662	IGLV3-21	immunoglobulin lambda variable 3-21 [Source:HGNC Symbol;Acc:HGNC:5905]	1.27
PMCTP5-005	4	ENSG00000123989	CHPF	chondroitin polymerizing factor [Source:HGNC Symbol;Acc:HGNC:24291]	0.93
PMCTP5-005	4	ENSG00000099958	DERL3	derlin 3 [Source:HGNC Symbol;Acc:HGNC:14236]	0.68
PMCTP5-005	4	ENSG00000211893	IGHG2	immunoglobulin heavy constant gamma 2 (G2m marker) [Source:HGNC Symbol;Acc:HGNC:5526]	0.70

Cluster ID	Cluster Size	Gene ID	Gene Name	Gene Description	Log_2 FC TIV
PMCTP5-005	4	ENSG00000115884	SDC1	syndecan 1 [Source:HGNC Symbol;Acc:HGNC:10658]	1.93
PMCTP5-006	4	ENSG00000124772	CPNE5	copine 5 [Source:HGNC Symbol;Acc:HGNC:2318]	0.65
PMCTP5-006	4	ENSG00000211896	IGHG1	immunoglobulin heavy constant gamma 1 (G1m marker) [Source:HGNC Symbol;Acc:HGNC:5525]	2.32
PMCTP5-006	4	ENSG00000253755	IGHGP	immunoglobulin heavy constant gamma P (non-functional) [Source:HGNC Symbol;Acc:HGNC:5529]	2.02
PMCTP5-006	4	ENSG00000244437	IGKV3-15	immunoglobulin kappa variable 3-15 [Source:HGNC Symbol;Acc:HGNC:5816]	0.87
PMCTP5-007	2	ENSG00000162551	ALPL	alkaline phosphatase, liver/bone/kidney [Source:HGNC Symbol;Acc:HGNC:438]	0.75
PMCTP5-007	2	ENSG00000108244	KRT23	keratin 23 [Source:HGNC Symbol;Acc:HGNC:6438]	0.48
PMCTP5-008	4	ENSG00000163464	CXCR1	C-X-C motif chemokine receptor 1 [Source:HGNC Symbol;Acc:HGNC:6026]	0.59
PMCTP5-008	4	ENSG00000180871	CXCR2	C-X-C motif chemokine receptor 2 [Source:HGNC Symbol;Acc:HGNC:6027]	0.55
PMCTP5-008	4	ENSG00000162747	FCGR3B	Fc fragment of IgG receptor IIIb [Source:HGNC Symbol;Acc:HGNC:3620]	0.61
PMCTP5-008	4	ENSG00000119922	IFIT2	interferon induced protein with tetratricopeptide repeats 2 [Source:HGNC Symbol;Acc:HGNC:5409]	0.28
PMCTP5-009	3	ENSG00000211945	IGHV1-18	immunoglobulin heavy variable 1-18 [Source:HGNC Symbol;Acc:HGNC:5549]	1.13
PMCTP5-009	3	ENSG00000211976	IGHV3-73	immunoglobulin heavy variable 3-73 [Source:HGNC Symbol;Acc:HGNC:5623]	1.40
PMCTP5-009	3	ENSG00000138160	KIF11	kinesin family member 11 [Source:HGNC Symbol;Acc:HGNC:6388]	0.70
PMCTP5-010	2	ENSG00000186529	CYP4F3	cytochrome P450 family 4 subfamily F member 3 [Source:HGNC Symbol;Acc:HGNC:2646]	0.61
PMCTP5-010	2	ENSG00000211967	IGHV3-53	immunoglobulin heavy variable 3-53 [Source:HGNC Symbol;Acc:HGNC:5610]	1.19
PMCTP5-011	2	ENSG00000133048	CHI3L1	chitinase 3 like 1 [Source:HGNC Symbol;Acc:HGNC:1932]	0.62
PMCTP5-011	2	ENSG00000125148	MT2A	metallothionein 2A [Source:HGNC Symbol;Acc:HGNC:7406]	0.18
PMCTP5-012	3	ENSG00000211972	IGHV3-66	immunoglobulin heavy variable 3-66 [Source:HGNC Symbol;Acc:HGNC:5619]	0.97
PMCTP5-012	3	ENSG00000211660	IGLV2-23	immunoglobulin lambda variable 2-23 [Source:HGNC Symbol;Acc:HGNC:5890]	1.25
PMCTP5-012	3	ENSG00000278196	IGLV2-8	immunoglobulin lambda variable 2-8 [Source:HGNC Symbol;Acc:HGNC:5895]	0.84
PMCTP5-013	3	ENSG00000182885	ADGRG3	adhesion G protein-coupled receptor G3 [Source:HGNC Symbol;Acc:HGNC:13728]	0.52
PMCTP5-013	3	ENSG00000211950	IGHV1-24	immunoglobulin heavy variable 1-24 [Source:HGNC Symbol;Acc:HGNC:5551]	1.19
PMCTP5-013	3	ENSG00000173535	TNFRSF10C	TNF receptor superfamily member 10c [Source:HGNC Symbol;Acc:HGNC:11906]	0.75
PMCTP5-014	4	ENSG00000224373	IGHV4-59	immunoglobulin heavy variable 4-59 [Source:HGNC Symbol;Acc:HGNC:5654]	1.27
PMCTP5-014	4	ENSG00000242076	IGKV1-33	immunoglobulin kappa variable 1-33 [Source:HGNC Symbol;Acc:HGNC:5737]	1.53
PMCTP5-014	4	ENSG00000239951	IGKV3-20	immunoglobulin kappa variable 3-20 [Source:HGNC Symbol;Acc:HGNC:5817]	0.82
PMCTP5-014	4	ENSG00000211669	IGLV3-10	immunoglobulin lambda variable 3-10 [Source:HGNC Symbol;Acc:HGNC:5897]	1.50
PMCTP5-015	2	ENSG00000239839	DEFA3	defensin alpha 3 [Source:HGNC Symbol;Acc:HGNC:2762]	0.63
PMCTP5-015	2	ENSG00000004799	PDK4	pyruvate dehydrogenase kinase 4 [Source:HGNC Symbol;Acc:HGNC:8812]	0.61
PMCTP5-016	3	ENSG00000206172	HBA1	hemoglobin subunit alpha 1 [Source:HGNC Symbol;Acc:HGNC:4823]	0.80
PMCTP5-016	3	ENSG00000188536	HBA2	hemoglobin subunit alpha 2 [Source:HGNC Symbol;Acc:HGNC:4824]	0.88
PMCTP5-016	3	ENSG00000124102	PI3	peptidase inhibitor 3 [Source:HGNC Symbol;Acc:HGNC:8947]	0.82
PMCTP5-017	3	ENSG00000211934	IGHV1-2	immunoglobulin heavy variable 1-2 [Source:HGNC Symbol;Acc:HGNC:5550]	0.72

Cluster ID	Cluster Size	Gene ID	Gene Name	Gene Description	Log_2 FC TIV
PMCTP5-017	3	ENSG00000211652	IGLV7-43	immunoglobulin lambda variable 7-43 [Source:HGNC Symbol;Acc:HGNC:5929]	1.43
PMCTP5-017	3	ENSG00000162366	PDZK1IP1	PDZK1 interacting protein 1 [Source:HGNC Symbol;Acc:HGNC:16887]	0.56
PMCTP5-018	2	ENSG00000158578	ALAS2	5'-aminolevulinate synthase 2 [Source:HGNC Symbol;Acc:HGNC:397]	0.89
PMCTP5-018	2	ENSG00000244734	HBB	hemoglobin subunit beta [Source:HGNC Symbol;Acc:HGNC:4827]	0.63
PMCTP5-019	6	ENSG00000211943	IGHV3-15	immunoglobulin heavy variable 3-15 [Source:HGNC Symbol;Acc:HGNC:5582]	0.73
PMCTP5-019	6	ENSG00000211933	IGHV6-1	immunoglobulin heavy variable 6-1 [Source:HGNC Symbol;Acc:HGNC:5662]	1.37
PMCTP5-019	6	ENSG00000253818	IGLV1-41	immunoglobulin lambda variable 1-41 (pseudogene) [Source:HGNC Symbol;Acc:HGNC:5878]	1.20
PMCTP5-019	6	ENSG00000197705	KLHL14	kelch like family member 14 [Source:HGNC Symbol;Acc:HGNC:29266]	0.63
PMCTP5-019	6	ENSG00000135862	LAMC1	laminin subunit gamma 1 [Source:HGNC Symbol;Acc:HGNC:6492]	0.45
PMCTP5-019	6	ENSG00000169116	PARM1	prostate androgen-regulated mucin-like protein 1 [Source:HGNC Symbol;Acc:HGNC:24536]	0.40

Table 42: Co-expressed gene clusters (PBMC, Day 5)

Cluster ID	Cluster Size	Gene ID	Gene Name	Gene Description	Log_2 FC TIV
PMCTP6-001	3	ENSG00000211947	IGHV3-21	immunoglobulin heavy variable 3-21 [Source:HGNC Symbol;Acc:HGNC:5586]	1.21
PMCTP6-001	3	ENSG00000211592	IGKC	immunoglobulin kappa constant [Source:HGNC Symbol;Acc:HGNC:5716]	1.20
PMCTP6-001	3	ENSG00000211679	IGLC3	immunoglobulin lambda constant 3 (Kern-Oz+ marker) [Source:HGNC Symbol;Acc:HGNC:5857]	1.50
PMCTP6-002	2	ENSG00000270550	IGHV3-30	immunoglobulin heavy variable 3-30 [Source:HGNC Symbol;Acc:HGNC:5591]	0.78
PMCTP6-002	2	ENSG00000239264	TXNDC5	thioredoxin domain containing 5 [Source:HGNC Symbol;Acc:HGNC:21073]	1.49
PMCTP6-003	2	ENSG00000243238	IGKV2-30	immunoglobulin kappa variable 2-30 [Source:HGNC Symbol;Acc:HGNC:5785]	2.08
PMCTP6-003	2	ENSG00000211668	IGLV2-11	immunoglobulin lambda variable 2-11 [Source:HGNC Symbol;Acc:HGNC:5887]	1.38
PMCTP6-004	3	ENSG00000197476			1.59
PMCTP6-004	3	ENSG00000170476	MZB1	marginal zone B and B1 cell specific protein [Source:HGNC Symbol;Acc:HGNC:30125]	1.48
PMCTP6-004	3	ENSG00000240505	TNFRSF13B	TNF receptor superfamily member 13B [Source:HGNC Symbol;Acc:HGNC:18153]	0.87
PMCTP6-005	2	ENSG00000211673	IGLV3-1	immunoglobulin lambda variable 3-1 [Source:HGNC Symbol;Acc:HGNC:5896]	1.56
PMCTP6-005	2	ENSG00000197705	KLHL14	kelch like family member 14 [Source:HGNC Symbol;Acc:HGNC:29266]	0.91
PMCTP6-006	2	ENSG00000170456	DENND5B	DENN domain containing 5B [Source:HGNC Symbol;Acc:HGNC:28338]	0.85
PMCTP6-006	2	ENSG00000211677	IGLC2	immunoglobulin lambda constant 2 [Source:HGNC Symbol;Acc:HGNC:5856]	1.89
PMCTP6-007	3	ENSG00000211893	IGHG2	immunoglobulin heavy constant gamma 2 (G2m marker) [Source:HGNC Symbol;Acc:HGNC:5526]	0.63
PMCTP6-007	3	ENSG00000211955	IGHV3-33	immunoglobulin heavy variable 3-33 [Source:HGNC Symbol;Acc:HGNC:5596]	1.52
PMCTP6-007	3	ENSG00000271178	IGHV3OR16-13	immunoglobulin heavy variable 3/OR16-13 (non-functional) [Source:HGNC Symbol;Acc:HGNC:5637]	2.06
PMCTP6-008	3	ENSG00000124772	CPNE5	copine 5 [Source:HGNC Symbol;Acc:HGNC:2318]	0.72

Cluster ID	Cluster Size	Gene ID	Gene Name	Gene Description	Log_2 FC TIV
PMCTP6-008	3	ENSG00000211669	IGLV3-10	immunoglobulin lambda variable 3-10 [Source:HGNC Symbol;Acc:HGNC:5897]	1.53
PMCTP6-008	3	ENSG00000211662	IGLV3-21	immunoglobulin lambda variable 3-21 [Source:HGNC Symbol;Acc:HGNC:5905]	2.07
PMCTP6-009	2	ENSG00000211945	IGHV1-18	immunoglobulin heavy variable 1-18 [Source:HGNC Symbol;Acc:HGNC:5549]	1.10
PMCTP6-009	2	ENSG00000110777	POU2AF1	POU class 2 associating factor 1 [Source:HGNC Symbol;Acc:HGNC:9211]	0.73
PMCTP6-010	4	ENSG00000105974	CAV1	caveolin 1 [Source:HGNC Symbol;Acc:HGNC:1527]	2.25
PMCTP6-010	4	ENSG00000118985	ELL2	elongation factor for RNA polymerase II 2 [Source:HGNC Symbol;Acc:HGNC:17064]	0.78
PMCTP6-010	4	ENSG00000211938	IGHV3-7	immunoglobulin heavy variable 3-7 [Source:HGNC Symbol;Acc:HGNC:5620]	3.02
PMCTP6-010	4	ENSG00000132465	JCHAIN	joining chain of multimeric IgA and IgM [Source:HGNC Symbol;Acc:HGNC:5713]	1.45
PMCTP6-011	5	ENSG00000099958	DERL3	derlin 3 [Source:HGNC Symbol;Acc:HGNC:14236]	0.75
PMCTP6-011	5	ENSG00000224373	IGHV4-59	immunoglobulin heavy variable 4-59 [Source:HGNC Symbol;Acc:HGNC:5654]	1.62
PMCTP6-011	5	ENSG00000241351	IGKV3-11	immunoglobulin kappa variable 3-11 [Source:HGNC Symbol;Acc:HGNC:5815]	1.45
PMCTP6-011	5	ENSG00000115884	SDC1	syndecan 1 [Source:HGNC Symbol;Acc:HGNC:10658]	2.42
PMCTP6-011	5	ENSG00000048462	TNFRSF17	TNF receptor superfamily member 17 [Source:HGNC Symbol;Acc:HGNC:11913]	1.84
PMCTP6-012	2	ENSG00000282639			1.05
PMCTP6-012	2	ENSG00000211653	IGLV1-40	immunoglobulin lambda variable 1-40 [Source:HGNC Symbol;Acc:HGNC:5877]	2.63
PMCTP6-013	2	ENSG00000138772	ANXA3	annexin A3 [Source:HGNC Symbol;Acc:HGNC:541]	0.37
PMCTP6-013	2	ENSG00000211964	IGHV3-48	immunoglobulin heavy variable 3-48 [Source:HGNC Symbol;Acc:HGNC:5606]	1.19
PMCTP6-014	3	ENSG00000239975	IGKV1D-33	immunoglobulin kappa variable 1D-33 [Source:HGNC Symbol;Acc:HGNC:5753]	2.24
PMCTP6-014	3	ENSG00000211648	IGLV1-47	immunoglobulin lambda variable 1-47 [Source:HGNC Symbol;Acc:HGNC:5880]	1.96
PMCTP6-014	3	ENSG00000211666	IGLV2-14	immunoglobulin lambda variable 2-14 [Source:HGNC Symbol;Acc:HGNC:5888]	1.07
PMCTP6-015	5	ENSG00000239839	DEFA3	defensin alpha 3 [Source:HGNC Symbol;Acc:HGNC:2762]	0.59
PMCTP6-015	5	ENSG00000123700	KCNJ2	potassium voltage-gated channel subfamily J member 2 [Source:HGNC Symbol;Acc:HGNC:6263]	0.43
PMCTP6-015	5	ENSG00000135862	LAMC1	laminin subunit gamma 1 [Source:HGNC Symbol;Acc:HGNC:6492]	0.59
PMCTP6-015	5	ENSG00000012223	LTF	lactotransferrin [Source:HGNC Symbol;Acc:HGNC:6720]	0.37
PMCTP6-015	5	ENSG00000232810	TNF	tumor necrosis factor [Source:HGNC Symbol;Acc:HGNC:11892]	0.39
PMCTP6-016	2	ENSG00000211966	IGHV5-51	immunoglobulin heavy variable 5-51 [Source:HGNC Symbol;Acc:HGNC:5659]	0.80
PMCTP6-016	2	ENSG00000243466	IGKV1-5	immunoglobulin kappa variable 1-5 [Source:HGNC Symbol;Acc:HGNC:5741]	1.17
PMCTP6-017	3	ENSG00000100336	APOL4	apolipoprotein L4 [Source:HGNC Symbol;Acc:HGNC:14867]	0.93
PMCTP6-017	3	ENSG00000123989	CHPF	chondroitin polymerizing factor [Source:HGNC Symbol;Acc:HGNC:24291]	0.84
PMCTP6-017	3	ENSG00000242076	IGKV1-33	immunoglobulin kappa variable 1-33 [Source:HGNC Symbol;Acc:HGNC:5737]	2.06
PMCTP6-018	6	ENSG00000211896	IGHG1	immunoglobulin heavy constant gamma 1 (G1m marker) [Source:HGNC Symbol;Acc:HGNC:5525]	2.96
PMCTP6-018	6	ENSG00000211897	IGHG3	immunoglobulin heavy constant gamma 3 (G3m marker) [Source:HGNC Symbol;Acc:HGNC:5527]	1.72
PMCTP6-018	6	ENSG00000253755	IGHGP	immunoglobulin heavy constant gamma P (non-functional) [Source:HGNC Symbol;Acc:HGNC:5529]	2.61
PMCTP6-018	6	ENSG00000244437	IGKV3-15	immunoglobulin kappa variable 3-15 [Source:HGNC Symbol;Acc:HGNC:5816]	1.04
PMCTP6-018	6	ENSG00000148773	MKI67	marker of proliferation Ki-67 [Source:HGNC Symbol;Acc:HGNC:7107]	0.60

Cluster ID	Cluster Size	Gene ID	Gene Name	Gene Description	Log_2 FC TIV
PMCTP6-018	6	ENSG00000101057	MYBL2	MYB proto-oncogene like 2 [Source:HGNC Symbol;Acc:HGNC:7548]	1.46
PMCTP6-019	4	ENSG00000211976	IGHV3-73	immunoglobulin heavy variable 3-73 [Source:HGNC Symbol;Acc:HGNC:5623]	1.50
PMCTP6-019	4	ENSG00000251546	IGKV1D-39	immunoglobulin kappa variable 1D-39 [Source:HGNC Symbol;Acc:HGNC:5756]	1.32
PMCTP6-019	4	ENSG00000239951	IGKV3-20	immunoglobulin kappa variable 3-20 [Source:HGNC Symbol;Acc:HGNC:5817]	1.05
PMCTP6-019	4	ENSG00000211660	IGLV2-23	immunoglobulin lambda variable 2-23 [Source:HGNC Symbol;Acc:HGNC:5890]	1.17
PMCTP6-020	5	ENSG00000137959	IFI44L	interferon induced protein 44 like [Source:HGNC Symbol;Acc:HGNC:17817]	0.25
PMCTP6-020	5	ENSG00000211899	IGHM	immunoglobulin heavy constant mu [Source:HGNC Symbol;Acc:HGNC:5541]	1.22
PMCTP6-020	5	ENSG00000211935	IGHV1-3	immunoglobulin heavy variable 1-3 [Source:HGNC Symbol;Acc:HGNC:5552]	1.08
PMCTP6-020	5	ENSG00000253451	IGLV2-28	immunoglobulin lambda variable 2-28 (pseudogene) [Source:HGNC Symbol;Acc:HGNC:5891]	1.78
PMCTP6-020	5	ENSG00000156587	UBE2L6	ubiquitin conjugating enzyme E2 L6 [Source:HGNC Symbol;Acc:HGNC:12490]	0.23
PMCTP6-021	5	ENSG00000136010	ALDH1L2	aldehyde dehydrogenase 1 family member L2 [Source:HGNC Symbol;Acc:HGNC:26777]	1.26
PMCTP6-021	5	ENSG00000211972	IGHV3-66	immunoglobulin heavy variable 3-66 [Source:HGNC Symbol;Acc:HGNC:5619]	1.70
PMCTP6-021	5	ENSG00000242371	IGKV1-39	immunoglobulin kappa variable 1-39 (gene/pseudogene) [Source:HGNC Symbol;Acc:HGNC:5740]	1.33
PMCTP6-021	5	ENSG00000125538	IL1B	interleukin 1 beta [Source:HGNC Symbol;Acc:HGNC:5992]	0.54
PMCTP6-021	5	ENSG00000177301	KCNA2	potassium voltage-gated channel subfamily A member 2 [Source:HGNC Symbol;Acc:HGNC:6220]	0.74
PMCTP6-022	3	ENSG00000211892	IGHG4	immunoglobulin heavy constant gamma 4 (G4m marker) [Source:HGNC Symbol;Acc:HGNC:5528]	1.37
PMCTP6-022	3	ENSG00000280411	IGHV1-69-2	immunoglobulin heavy variable 1-69-2 [Source:HGNC Symbol;Acc:HGNC:5562]	2.02
PMCTP6-022	3	ENSG00000171241	SHCBP1	SHC binding and spindle associated 1 [Source:HGNC Symbol;Acc:HGNC:29547]	0.79
PMCTP6-023	2	ENSG00000211949	IGHV3-23	immunoglobulin heavy variable 3-23 [Source:HGNC Symbol;Acc:HGNC:5588]	0.66
PMCTP6-023	2	ENSG00000088726	TMEM40	transmembrane protein 40 [Source:HGNC Symbol;Acc:HGNC:25620]	0.17
PMCTP6-024	4	ENSG00000224650	IGHV3-74	immunoglobulin heavy variable 3-74 [Source:HGNC Symbol;Acc:HGNC:5624]	1.64
PMCTP6-024	4	ENSG00000254395	IGHV4-55	immunoglobulin heavy variable 4-55 (pseudogene) [Source:HGNC Symbol;Acc:HGNC:5653]	1.55
PMCTP6-024	4	ENSG00000211651	IGLV1-44	immunoglobulin lambda variable 1-44 [Source:HGNC Symbol;Acc:HGNC:5879]	2.58
PMCTP6-024	4	ENSG00000211644	IGLV1-51	immunoglobulin lambda variable 1-51 [Source:HGNC Symbol;Acc:HGNC:5882]	2.04
PMCTP6-025	5	ENSG00000198814	GK	glycerol kinase [Source:HGNC Symbol;Acc:HGNC:4289]	0.12
PMCTP6-025	5	ENSG00000182782	HCAR2	hydroxycarboxylic acid receptor 2 [Source:HGNC Symbol;Acc:HGNC:24827]	0.61
PMCTP6-025	5	ENSG00000211943	IGHV3-15	immunoglobulin heavy variable 3-15 [Source:HGNC Symbol;Acc:HGNC:5582]	1.25
PMCTP6-025	5	ENSG00000241294	IGKV2-24	immunoglobulin kappa variable 2-24 [Source:HGNC Symbol;Acc:HGNC:5781]	1.52
PMCTP6-025	5	ENSG00000253818	IGLV1-41	immunoglobulin lambda variable 1-41 (pseudogene) [Source:HGNC Symbol;Acc:HGNC:5878]	1.82
PMCTP6-026	6	ENSG00000232176			0.42
PMCTP6-026	6	ENSG00000183508	FAM46C	family with sequence similarity 46 member C [Source:HGNC Symbol;Acc:HGNC:24712]	1.07
PMCTP6-026	6	ENSG00000211946	IGHV3-20	immunoglobulin heavy variable 3-20 [Source:HGNC Symbol;Acc:HGNC:5585]	1.43

Cluster ID	Cluster Size	Gene ID	Gene Name	Gene Description	Log_2 FC TIV
PMCTP6-026	6	ENSG00000241755	IGKV1-9	immunoglobulin kappa variable 1-9 [Source:HGNC Symbol;Acc:HGNC:5744]	1.57
PMCTP6-026	6	ENSG00000211598	IGKV4-1	immunoglobulin kappa variable 4-1 [Source:HGNC Symbol;Acc:HGNC:5834]	0.69
PMCTP6-026	6	ENSG00000171848	RRM2	ribonucleotide reductase regulatory subunit M2 [Source:HGNC Symbol;Acc:HGNC:10452]	0.72
PMCTP6-027	2	ENSG00000211649	IGLV7-46	immunoglobulin lambda variable 7-46 (gene/pseudogene) [Source:HGNC Symbol;Acc:HGNC:5930]	1.55
PMCTP6-027	2	ENSG00000197208	SLC22A4	solute carrier family 22 member 4 [Source:HGNC Symbol;Acc:HGNC:10968]	0.27
PMCTP6-028	7	ENSG00000282651			0.83
PMCTP6-028	7	ENSG00000211934	IGHV1-2	immunoglobulin heavy variable 1-2 [Source:HGNC Symbol;Acc:HGNC:5550]	0.84
PMCTP6-028	7	ENSG00000211962	IGHV1-46	immunoglobulin heavy variable 1-46 [Source:HGNC Symbol;Acc:HGNC:5554]	0.98
PMCTP6-028	7	ENSG00000232216	IGHV3-43	immunoglobulin heavy variable 3-43 [Source:HGNC Symbol;Acc:HGNC:5604]	1.22
PMCTP6-028	7	ENSG00000211967	IGHV3-53	immunoglobulin heavy variable 3-53 [Source:HGNC Symbol;Acc:HGNC:5610]	1.88
PMCTP6-028	7	ENSG00000211659	IGLV3-25	immunoglobulin lambda variable 3-25 [Source:HGNC Symbol;Acc:HGNC:5908]	2.12
PMCTP6-028	7	ENSG00000167900	TK1	thymidine kinase 1 [Source:HGNC Symbol;Acc:HGNC:11830]	0.81
PMCTP6-029	3	ENSG00000197561	ELANE	elastase, neutrophil expressed [Source:HGNC Symbol;Acc:HGNC:3309]	0.70
PMCTP6-029	3	ENSG00000278196	IGLV2-8	immunoglobulin lambda variable 2-8 [Source:HGNC Symbol;Acc:HGNC:5895]	0.90
PMCTP6-029	3	ENSG00000169116	PARM1	prostate androgen-regulated mucin-like protein 1 [Source:HGNC Symbol;Acc:HGNC:24536]	0.65
PMCTP6-030	4	ENSG00000278828	HIST1H3H	histone cluster 1 H3 family member h [Source:HGNC Symbol;Acc:HGNC:4775]	0.09
PMCTP6-030	4	ENSG00000184678	HIST2H2BE	histone cluster 2 H2B family member e [Source:HGNC Symbol;Acc:HGNC:4760]	0.10
PMCTP6-030	4	ENSG00000253822	IGLV3-24	immunoglobulin lambda variable 3-24 (pseudogene) [Source:HGNC Symbol;Acc:HGNC:5907]	1.94
PMCTP6-030	4	ENSG00000156265	MAP3K7CL	MAP3K7 C-terminal like [Source:HGNC Symbol;Acc:HGNC:16457]	0.10
PMCTP6-031	6	ENSG00000062282	DGAT2	diacylglycerol O-acyltransferase 2 [Source:HGNC Symbol;Acc:HGNC:16940]	0.27
PMCTP6-031	6	ENSG00000211950	IGHV1-24	immunoglobulin heavy variable 1-24 [Source:HGNC Symbol;Acc:HGNC:5551]	1.39
PMCTP6-031	6	ENSG00000211685	IGLC7	immunoglobulin lambda constant 7 [Source:HGNC Symbol;Acc:HGNC:5861]	1.87
PMCTP6-031	6	ENSG00000254709	IGLL5	immunoglobulin lambda like polypeptide 5 [Source:HGNC Symbol;Acc:HGNC:38476]	1.23
PMCTP6-031	6	ENSG00000183762	KREMEN1	kringle containing transmembrane protein 1 [Source:HGNC Symbol;Acc:HGNC:17550]	0.64
PMCTP6-031	6	ENSG00000128438	TBC1D27	TBC1 domain family member 27 [Source:HGNC Symbol;Acc:HGNC:28104]	0.89
PMCTP6-032	4	ENSG00000122786	CALD1	caldesmon 1 [Source:HGNC Symbol;Acc:HGNC:1441]	0.62
PMCTP6-032	4	ENSG00000211933	IGHV6-1	immunoglobulin heavy variable 6-1 [Source:HGNC Symbol;Acc:HGNC:5662]	1.59
PMCTP6-032	4	ENSG00000244575	IGKV1-27	immunoglobulin kappa variable 1-27 [Source:HGNC Symbol;Acc:HGNC:5735]	1.00
PMCTP6-032	4	ENSG00000211652	IGLV7-43	immunoglobulin lambda variable 7-43 [Source:HGNC Symbol;Acc:HGNC:5929]	1.59
PMCTP6-033	4	ENSG00000146592	CREB5	cAMP responsive element binding protein 5 [Source:HGNC Symbol;Acc:HGNC:16844]	-0.03
PMCTP6-033	4	ENSG00000168209	DDIT4	DNA damage inducible transcript 4 [Source:HGNC Symbol;Acc:HGNC:24944]	-0.61
PMCTP6-033	4	ENSG00000183019	MCEMP1	mast cell expressed membrane protein 1 [Source:HGNC Symbol;Acc:HGNC:27291]	-0.24

Cluster ID	Cluster Size	Gene ID	Gene Name	Gene Description	Log_2 FC TIV
PMCTP6-033	4	ENSG00000149573	MPZL2	myelin protein zero like 2 [Source:HGNC Symbol;Acc:HGNC:3496]	-0.07

Table 43: Co-expressed gene clusters (PBMC, Day 6)

Cluster ID	Cluster Size	Gene ID	Gene Name	Gene Description	Log_2 FC TIV
PMCTP7-001	2	ENSG00000211896	IGHG1	immunoglobulin heavy constant gamma 1 (G1m marker) [Source:HGNC Symbol;Acc:HGNC:5525]	2.57
PMCTP7-001	2	ENSG00000253755	IGHGP	immunoglobulin heavy constant gamma P (non-functional) [Source:HGNC Symbol;Acc:HGNC:5529]	2.25
PMCTP7-002	2	ENSG00000270550	IGHV3-30	immunoglobulin heavy variable 3-30 [Source:HGNC Symbol;Acc:HGNC:5591]	0.38
PMCTP7-002	2	ENSG00000187608	ISG15	ISG15 ubiquitin-like modifier [Source:HGNC Symbol;Acc:HGNC:4053]	0.68
PMCTP7-003	3	ENSG00000178445	GLDC	glycine decarboxylase [Source:HGNC Symbol;Acc:HGNC:4313]	1.50
PMCTP7-003	3	ENSG00000211938	IGHV3-7	immunoglobulin heavy variable 3-7 [Source:HGNC Symbol;Acc:HGNC:5620]	2.35
PMCTP7-003	3	ENSG00000132465	JCHAIN	joining chain of multimeric IgA and IgM [Source:HGNC Symbol;Acc:HGNC:5713]	1.16
PMCTP7-004	3	ENSG00000271178	IGHV3OR16-13	immunoglobulin heavy variable 3/OR16-13 (non-functional) [Source:HGNC Symbol;Acc:HGNC:5637]	1.94
PMCTP7-004	3	ENSG00000211679	IGLC3	immunoglobulin lambda constant 3 (Kern-Oz+ marker) [Source:HGNC Symbol;Acc:HGNC:5857]	1.19
PMCTP7-004	3	ENSG00000101057	MYBL2	MYB proto-oncogene like 2 [Source:HGNC Symbol;Acc:HGNC:7548]	1.20
PMCTP7-005	2	ENSG00000211935	IGHV1-3	immunoglobulin heavy variable 1-3 [Source:HGNC Symbol;Acc:HGNC:5552]	0.85
PMCTP7-005	2	ENSG00000224373	IGHV4-59	immunoglobulin heavy variable 4-59 [Source:HGNC Symbol;Acc:HGNC:5654]	1.67
PMCTP7-006	3	ENSG00000211893	IGHG2	immunoglobulin heavy constant gamma 2 (G2m marker) [Source:HGNC Symbol;Acc:HGNC:5526]	0.47
PMCTP7-006	3	ENSG00000211897	IGHG3	immunoglobulin heavy constant gamma 3 (G3m marker) [Source:HGNC Symbol;Acc:HGNC:5527]	1.37
PMCTP7-006	3	ENSG00000211592	IGKC	immunoglobulin kappa constant [Source:HGNC Symbol;Acc:HGNC:5716]	0.80
PMCTP7-007	6	ENSG00000124772	CPNE5	copine 5 [Source:HGNC Symbol;Acc:HGNC:2318]	0.70
PMCTP7-007	6	ENSG00000241351	IGKV3-11	immunoglobulin kappa variable 3-11 [Source:HGNC Symbol;Acc:HGNC:5815]	1.14
PMCTP7-007	6	ENSG00000170476	MZB1	marginal zone B and B1 cell specific protein [Source:HGNC Symbol;Acc:HGNC:30125]	1.26
PMCTP7-007	6	ENSG00000110777	POU2AF1	POU class 2 associating factor 1 [Source:HGNC Symbol;Acc:HGNC:9211]	0.64
PMCTP7-007	6	ENSG00000115884	SDC1	syndecan 1 [Source:HGNC Symbol;Acc:HGNC:10658]	2.30
PMCTP7-007	6	ENSG00000167900	TK1	thymidine kinase 1 [Source:HGNC Symbol;Acc:HGNC:11830]	0.58
PMCTP7-008	2	ENSG00000224650	IGHV3-74	immunoglobulin heavy variable 3-74 [Source:HGNC Symbol;Acc:HGNC:5624]	0.82
PMCTP7-008	2	ENSG00000211685	IGLC7	immunoglobulin lambda constant 7 [Source:HGNC Symbol;Acc:HGNC:5861]	1.09
PMCTP7-009	3	ENSG00000211947	IGHV3-21	immunoglobulin heavy variable 3-21 [Source:HGNC Symbol;Acc:HGNC:5586]	0.82
PMCTP7-009	3	ENSG00000239951	IGKV3-20	immunoglobulin kappa variable 3-20 [Source:HGNC Symbol;Acc:HGNC:5817]	0.76
PMCTP7-009	3	ENSG00000148773	MKI67	marker of proliferation Ki-67 [Source:HGNC Symbol;Acc:HGNC:7107]	0.28
PMCTP7-010	3	ENSG00000123989	CHPF	chondroitin polymerizing factor [Source:HGNC Symbol;Acc:HGNC:24291]	0.81
PMCTP7-010	3	ENSG00000240505	TNFRSF13B	TNF receptor superfamily member 13B [Source:HGNC Symbol;Acc:HGNC:18153]	0.64

Cluster ID	Cluster Size	Gene ID	Gene Name	Gene Description	Log_2 FC TIV
PMCTP7-010	3	ENSG00000048462	TNFRSF17	TNF receptor superfamily member 17 [Source:HGNC Symbol;Acc:HGNC:11913]	1.33
PMCTP7-011	3	ENSG00000272821			0.64
PMCTP7-011	3	ENSG00000211653	IGLV1-40	immunoglobulin lambda variable 1-40 [Source:HGNC Symbol;Acc:HGNC:5877]	2.75
PMCTP7-011	3	ENSG00000211662	IGLV3-21	immunoglobulin lambda variable 3-21 [Source:HGNC Symbol;Acc:HGNC:5905]	2.35
PMCTP7-012	5	ENSG00000211945	IGHV1-18	immunoglobulin heavy variable 1-18 [Source:HGNC Symbol;Acc:HGNC:5549]	1.03
PMCTP7-012	5	ENSG00000211955	IGHV3-33	immunoglobulin heavy variable 3-33 [Source:HGNC Symbol;Acc:HGNC:5596]	1.13
PMCTP7-012	5	ENSG00000211972	IGHV3-66	immunoglobulin heavy variable 3-66 [Source:HGNC Symbol;Acc:HGNC:5619]	1.86
PMCTP7-012	5	ENSG00000244437	IGKV3-15	immunoglobulin kappa variable 3-15 [Source:HGNC Symbol;Acc:HGNC:5816]	1.05
PMCTP7-012	5	ENSG00000177606	JUN	Jun proto-oncogene, AP-1 transcription factor subunit [Source:HGNC Symbol;Acc:HGNC:6204]	0.75
PMCTP7-013	2	ENSG00000177301	KCNA2	potassium voltage-gated channel subfamily A member 2 [Source:HGNC Symbol;Acc:HGNC:6220]	0.68
PMCTP7-013	2	ENSG00000128438	TBC1D27	TBC1 domain family member 27 [Source:HGNC Symbol;Acc:HGNC:28104]	0.74
PMCTP7-014	2	ENSG00000137198	GMPR	guanosine monophosphate reductase [Source:HGNC Symbol;Acc:HGNC:4376]	0.64
PMCTP7-014	2	ENSG00000184792	OSBP2	oxysterol binding protein 2 [Source:HGNC Symbol;Acc:HGNC:8504]	0.77
PMCTP7-015	3	ENSG00000196787	HIST1H2AG	histone cluster 1 H2A family member g [Source:HGNC Symbol;Acc:HGNC:4737]	0.70
PMCTP7-015	3	ENSG00000211644	IGLV1-51	immunoglobulin lambda variable 1-51 [Source:HGNC Symbol;Acc:HGNC:5882]	1.16
PMCTP7-015	3	ENSG00000011105	TSPAN9	tetraspanin 9 [Source:HGNC Symbol;Acc:HGNC:21640]	0.44
PMCTP7-016	3	ENSG00000211933	IGHV6-1	immunoglobulin heavy variable 6-1 [Source:HGNC Symbol;Acc:HGNC:5662]	1.35
PMCTP7-016	3	ENSG00000211673	IGLV3-1	immunoglobulin lambda variable 3-1 [Source:HGNC Symbol;Acc:HGNC:5896]	1.75
PMCTP7-016	3	ENSG00000205309	NT5M	5',3'-nucleotidase, mitochondrial [Source:HGNC Symbol;Acc:HGNC:15769]	0.55
PMCTP7-017	4	ENSG00000282651			0.52
PMCTP7-017	4	ENSG00000099958	DERL3	derlin 3 [Source:HGNC Symbol;Acc:HGNC:14236]	0.63
PMCTP7-017	4	ENSG00000211950	IGHV1-24	immunoglobulin heavy variable 1-24 [Source:HGNC Symbol;Acc:HGNC:5551]	1.60
PMCTP7-017	4	ENSG00000211669	IGLV3-10	immunoglobulin lambda variable 3-10 [Source:HGNC Symbol;Acc:HGNC:5897]	0.87
PMCTP7-018	2	ENSG00000223609	HBD	hemoglobin subunit delta [Source:HGNC Symbol;Acc:HGNC:4829]	0.93
PMCTP7-018	2	ENSG00000211934	IGHV1-2	immunoglobulin heavy variable 1-2 [Source:HGNC Symbol;Acc:HGNC:5550]	0.74
PMCTP7-019	3	ENSG00000211964	IGHV3-48	immunoglobulin heavy variable 3-48 [Source:HGNC Symbol;Acc:HGNC:5606]	1.28
PMCTP7-019	3	ENSG00000197705	KLHL14	kelch like family member 14 [Source:HGNC Symbol;Acc:HGNC:29266]	0.76
PMCTP7-019	3	ENSG00000135862	LAMC1	laminin subunit gamma 1 [Source:HGNC Symbol;Acc:HGNC:6492]	0.47
PMCTP7-020	2	ENSG00000211651	IGLV1-44	immunoglobulin lambda variable 1-44 [Source:HGNC Symbol;Acc:HGNC:5879]	2.03
PMCTP7-020	2	ENSG00000211666	IGLV2-14	immunoglobulin lambda variable 2-14 [Source:HGNC Symbol;Acc:HGNC:5888]	0.53
PMCTP7-021	3	ENSG00000232216	IGHV3-43	immunoglobulin heavy variable 3-43 [Source:HGNC Symbol;Acc:HGNC:5604]	0.75
PMCTP7-021	3	ENSG00000211659	IGLV3-25	immunoglobulin lambda variable 3-25 [Source:HGNC Symbol;Acc:HGNC:5908]	1.87
PMCTP7-021	3	ENSG00000012223	LTF	lactotransferrin [Source:HGNC Symbol;Acc:HGNC:6720]	0.59

Cluster ID	Cluster Size	Gene ID	Gene Name	Gene Description	Log_2 FC TIV
PMCTP7-022	3	ENSG00000165702	GFI1B	growth factor independent 1B transcriptional repressor [Source:HGNC Symbol;Acc:HGNC:4238]	0.46
PMCTP7-022	3	ENSG00000005961	ITGA2B	integrin subunit alpha 2b [Source:HGNC Symbol;Acc:HGNC:6138]	0.65
PMCTP7-022	3	ENSG00000088826	SMOX	spermine oxidase [Source:HGNC Symbol;Acc:HGNC:15862]	0.65
PMCTP7-023	4	ENSG00000138772	ANXA3	annexin A3 [Source:HGNC Symbol;Acc:HGNC:541]	0.55
PMCTP7-023	4	ENSG00000133048	CHI3L1	chitinase 3 like 1 [Source:HGNC Symbol;Acc:HGNC:1932]	0.30
PMCTP7-023	4	ENSG00000211943	IGHV3-15	immunoglobulin heavy variable 3-15 [Source:HGNC Symbol;Acc:HGNC:5582]	1.01
PMCTP7-023	4	ENSG00000143878	RHOB	ras homolog family member B [Source:HGNC Symbol;Acc:HGNC:668]	0.40
PMCTP7-024	3	ENSG00000211966	IGHV5-51	immunoglobulin heavy variable 5-51 [Source:HGNC Symbol;Acc:HGNC:5659]	0.45
PMCTP7-024	3	ENSG00000243238	IGKV2-30	immunoglobulin kappa variable 2-30 [Source:HGNC Symbol;Acc:HGNC:5785]	1.47
PMCTP7-024	3	ENSG00000171223	JUNB	JunB proto-oncogene, AP-1 transcription factor subunit [Source:HGNC Symbol;Acc:HGNC:6205]	0.57
PMCTP7-025	2	ENSG00000211962	IGHV1-46	immunoglobulin heavy variable 1-46 [Source:HGNC Symbol;Acc:HGNC:5554]	0.67
PMCTP7-025	2	ENSG00000211967	IGHV3-53	immunoglobulin heavy variable 3-53 [Source:HGNC Symbol;Acc:HGNC:5610]	1.59
PMCTP7-026	4	ENSG00000170345	FOS	Fos proto-oncogene, AP-1 transcription factor subunit [Source:HGNC Symbol;Acc:HGNC:3796]	0.59
PMCTP7-026	4	ENSG00000211892	IGHG4	immunoglobulin heavy constant gamma 4 (G4m marker) [Source:HGNC Symbol;Acc:HGNC:5528]	1.10
PMCTP7-026	4	ENSG00000280411	IGHV1-69-2	immunoglobulin heavy variable 1-69-2 [Source:HGNC Symbol;Acc:HGNC:5562]	1.51
PMCTP7-026	4	ENSG00000211959	IGHV4-39	immunoglobulin heavy variable 4-39 [Source:HGNC Symbol;Acc:HGNC:5651]	1.10
PMCTP7-027	3	ENSG00000100336	APOL4	apolipoprotein L4 [Source:HGNC Symbol;Acc:HGNC:14867]	0.56
PMCTP7-027	3	ENSG00000105974	CAV1	caveolin 1 [Source:HGNC Symbol;Acc:HGNC:1527]	1.93
PMCTP7-027	3	ENSG00000169116	PARM1	prostate androgen-regulated mucin-like protein 1 [Source:HGNC Symbol;Acc:HGNC:24536]	0.49
PMCTP7-028	5	ENSG00000136010	ALDH1L2	aldehyde dehydrogenase 1 family member L2 [Source:HGNC Symbol;Acc:HGNC:26777]	0.77
PMCTP7-028	5	ENSG00000170456	DENND5B	DENN domain containing 5B [Source:HGNC Symbol;Acc:HGNC:28338]	0.62
PMCTP7-028	5	ENSG00000118985	ELL2	elongation factor for RNA polymerase II 2 [Source:HGNC Symbol;Acc:HGNC:17064]	0.54
PMCTP7-028	5	ENSG00000211677	IGLC2	immunoglobulin lambda constant 2 [Source:HGNC Symbol;Acc:HGNC:5856]	1.84
PMCTP7-028	5	ENSG00000135916	ITM2C	integral membrane protein 2C [Source:HGNC Symbol;Acc:HGNC:6175]	0.72
PMCTP7-029	3	ENSG00000254395	IGHV4-55	immunoglobulin heavy variable 4-55 (pseudogene) [Source:HGNC Symbol;Acc:HGNC:5653]	1.28
PMCTP7-029	3	ENSG00000211670	IGLV3-9	immunoglobulin lambda variable 3-9 (gene/pseudogene) [Source:HGNC Symbol;Acc:HGNC:5918]	1.63
PMCTP7-029	3	ENSG00000004799	PDK4	pyruvate dehydrogenase kinase 4 [Source:HGNC Symbol;Acc:HGNC:8812]	0.57
PMCTP7-030	3	ENSG00000253822	IGLV3-24	immunoglobulin lambda variable 3-24 (pseudogene) [Source:HGNC Symbol;Acc:HGNC:5907]	1.44
PMCTP7-030	3	ENSG00000113494	PRLR	prolactin receptor [Source:HGNC Symbol;Acc:HGNC:9446]	0.03
PMCTP7-030	3	ENSG00000180113	TDRD6	tudor domain containing 6 [Source:HGNC Symbol;Acc:HGNC:21339]	0.05

Table 44: Co-expressed gene clusters (PBMC, Day 7)

Cluster ID	Cluster Size	Gene ID	Gene Name	Gene Description	Log_2 FC TIV
PMCTP8-001	2	ENSG00000171051	FPR1	formyl peptide receptor 1 [Source:HGNC Symbol;Acc:HGNC:3826]	0.38
PMCTP8-001	2	ENSG00000157551	KCNJ15	potassium voltage-gated channel subfamily J member 15 [Source:HGNC Symbol;Acc:HGNC:6261]	1.18
PMCTP8-002	2	ENSG00000211677	IGLC2	immunoglobulin lambda constant 2 [Source:HGNC Symbol;Acc:HGNC:5856]	1.91
PMCTP8-002	2	ENSG00000211673	IGLV3-1	immunoglobulin lambda variable 3-1 [Source:HGNC Symbol;Acc:HGNC:5896]	1.18
PMCTP8-003	2	ENSG00000211899	IGHM	immunoglobulin heavy constant mu [Source:HGNC Symbol;Acc:HGNC:5541]	0.73
PMCTP8-003	2	ENSG00000244437	IGKV3-15	immunoglobulin kappa variable 3-15 [Source:HGNC Symbol;Acc:HGNC:5816]	0.70
PMCTP8-004	2	ENSG00000143226	FCGR2A	Fc fragment of IgG receptor IIa [Source:HGNC Symbol;Acc:HGNC:3616]	0.41
PMCTP8-004	2	ENSG00000101057	MYBL2	MYB proto-oncogene like 2 [Source:HGNC Symbol;Acc:HGNC:7548]	1.01
PMCTP8-005	2	ENSG00000224373	IGHV4-59	immunoglobulin heavy variable 4-59 [Source:HGNC Symbol;Acc:HGNC:5654]	1.83
PMCTP8-005	2	ENSG00000121858	TNFSF10	tumor necrosis factor superfamily member 10 [Source:HGNC Symbol;Acc:HGNC:11925]	0.17
PMCTP8-006	3	ENSG00000171049	FPR2	formyl peptide receptor 2 [Source:HGNC Symbol;Acc:HGNC:3827]	0.59
PMCTP8-006	3	ENSG00000196549	MME	membrane metalloendopeptidase [Source:HGNC Symbol;Acc:HGNC:7154]	0.93
PMCTP8-006	3	ENSG00000163993	S100P	S100 calcium binding protein P [Source:HGNC Symbol;Acc:HGNC:10504]	0.88
PMCTP8-007	4	ENSG00000163464	CXCR1	C-X-C motif chemokine receptor 1 [Source:HGNC Symbol;Acc:HGNC:6026]	1.24
PMCTP8-007	4	ENSG00000180871	CXCR2	C-X-C motif chemokine receptor 2 [Source:HGNC Symbol;Acc:HGNC:6027]	1.03
PMCTP8-007	4	ENSG00000162747	FCGR3B	Fc fragment of IgG receptor IIIb [Source:HGNC Symbol;Acc:HGNC:3620]	1.24
PMCTP8-007	4	ENSG00000128438	TBC1D27	TBC1 domain family member 27 [Source:HGNC Symbol;Acc:HGNC:28104]	0.64
PMCTP8-008	2	ENSG00000211896	IGHG1	immunoglobulin heavy constant gamma 1 (G1m marker) [Source:HGNC Symbol;Acc:HGNC:5525]	2.45
PMCTP8-008	2	ENSG00000211955	IGHV3-33	immunoglobulin heavy variable 3-33 [Source:HGNC Symbol;Acc:HGNC:5596]	1.11
PMCTP8-009	4	ENSG00000133048	CHI3L1	chitinase 3 like 1 [Source:HGNC Symbol;Acc:HGNC:1932]	0.86
PMCTP8-009	4	ENSG00000062282	DGAT2	diacylglycerol O-acyltransferase 2 [Source:HGNC Symbol;Acc:HGNC:16940]	0.71
PMCTP8-009	4	ENSG00000182782	HCAR2	hydroxycarboxylic acid receptor 2 [Source:HGNC Symbol;Acc:HGNC:24827]	1.36
PMCTP8-009	4	ENSG00000211666	IGLV2-14	immunoglobulin lambda variable 2-14 [Source:HGNC Symbol;Acc:HGNC:5888]	0.63
PMCTP8-010	2	ENSG00000211964	IGHV3-48	immunoglobulin heavy variable 3-48 [Source:HGNC Symbol;Acc:HGNC:5606]	1.17
PMCTP8-010	2	ENSG00000211670	IGLV3-9	immunoglobulin lambda variable 3-9 (gene/pseudogene) [Source:HGNC Symbol;Acc:HGNC:5918]	2.26
PMCTP8-011	2	ENSG00000232176			0.96
PMCTP8-011	2	ENSG00000135862	LAMC1	laminin subunit gamma 1 [Source:HGNC Symbol;Acc:HGNC:6492]	0.42
PMCTP8-012	2	ENSG00000211653	IGLV1-40	immunoglobulin lambda variable 1-40 [Source:HGNC Symbol;Acc:HGNC:5877]	2.86
PMCTP8-012	2	ENSG00000170476	MZB1	marginal zone B and B1 cell specific protein [Source:HGNC Symbol;Acc:HGNC:30125]	1.09
PMCTP8-013	2	ENSG00000241755	IGKV1-9	immunoglobulin kappa variable 1-9 [Source:HGNC Symbol;Acc:HGNC:5744]	1.63
PMCTP8-013	2	ENSG00000211662	IGLV3-21	immunoglobulin lambda variable 3-21 [Source:HGNC Symbol;Acc:HGNC:5905]	2.94
PMCTP8-014	2	ENSG00000248996			0.37

Cluster ID	Cluster Size	Gene ID	Gene Name	Gene Description	Log_2 FC TIV
PMCTP8-014	2	ENSG00000142089	IFITM3	interferon induced transmembrane protein 3 [Source:HGNC Symbol;Acc:HGNC:5414]	0.59
PMCTP8-015	4	ENSG00000244734	HBB	hemoglobin subunit beta [Source:HGNC Symbol;Acc:HGNC:4827]	0.56
PMCTP8-015	4	ENSG00000173110	HSPA6	heat shock protein family A (Hsp70) member 6 [Source:HGNC Symbol;Acc:HGNC:5239]	0.72
PMCTP8-015	4	ENSG00000173868	PHOSPHO1	phosphoethanolamine/phosphocholine phosphatase [Source:HGNC Symbol;Acc:HGNC:16815]	0.65
PMCTP8-015	4	ENSG00000173535	TNFRSF10C	TNF receptor superfamily member 10c [Source:HGNC Symbol;Acc:HGNC:11906]	1.30
PMCTP8-016	2	ENSG00000253755	IGHGP	immunoglobulin heavy constant gamma P (non-functional) [Source:HGNC Symbol;Acc:HGNC:5529]	1.95
PMCTP8-016	2	ENSG00000270550	IGHV3-30	immunoglobulin heavy variable 3-30 [Source:HGNC Symbol;Acc:HGNC:5591]	0.46
PMCTP8-017	4	ENSG00000103569	AQP9	aquaporin 9 [Source:HGNC Symbol;Acc:HGNC:643]	0.81
PMCTP8-017	4	ENSG00000198814	GK	glycerol kinase [Source:HGNC Symbol;Acc:HGNC:4289]	0.32
PMCTP8-017	4	ENSG00000204388	HSPA1B	heat shock protein family A (Hsp70) member 1B [Source:HGNC Symbol;Acc:HGNC:5233]	0.55
PMCTP8-017	4	ENSG00000162366	PDZK1IP1	PDZK1 interacting protein 1 [Source:HGNC Symbol;Acc:HGNC:16887]	0.90
PMCTP8-018	4	ENSG00000128383	APOBEC3A	apolipoprotein B mRNA editing enzyme catalytic subunit 3A [Source:HGNC Symbol;Acc:HGNC:17343]	0.48
PMCTP8-018	4	ENSG00000211897	IGHG3	immunoglobulin heavy constant gamma 3 (G3m marker) [Source:HGNC Symbol;Acc:HGNC:5527]	1.28
PMCTP8-018	4	ENSG00000271178	IGHV3OR16-13	immunoglobulin heavy variable 3/OR16-13 (non-functional) [Source:HGNC Symbol;Acc:HGNC:5637]	1.04
PMCTP8-018	4	ENSG00000048462	TNFRSF17	TNF receptor superfamily member 17 [Source:HGNC Symbol;Acc:HGNC:11913]	1.06
PMCTP8-019	2	ENSG00000211946	IGHV3-20	immunoglobulin heavy variable 3-20 [Source:HGNC Symbol;Acc:HGNC:5585]	0.77
PMCTP8-019	2	ENSG00000211959	IGHV4-39	immunoglobulin heavy variable 4-39 [Source:HGNC Symbol;Acc:HGNC:5651]	1.43
PMCTP8-020	5	ENSG00000198019	FCGR1B	Fc fragment of IgG receptor Ib [Source:HGNC Symbol;Acc:HGNC:3614]	0.57
PMCTP8-020	5	ENSG00000211938	IGHV3-7	immunoglobulin heavy variable 3-7 [Source:HGNC Symbol;Acc:HGNC:5620]	1.78
PMCTP8-020	5	ENSG00000211598	IGKV4-1	immunoglobulin kappa variable 4-1 [Source:HGNC Symbol;Acc:HGNC:5834]	0.34
PMCTP8-020	5	ENSG00000132465	JCHAIN	joining chain of multimeric IgA and IgM [Source:HGNC Symbol;Acc:HGNC:5713]	0.99
PMCTP8-020	5	ENSG00000197705	KLHL14	kelch like family member 14 [Source:HGNC Symbol;Acc:HGNC:29266]	0.59
PMCTP8-021	3	ENSG00000146592	CREB5	cAMP responsive element binding protein 5 [Source:HGNC Symbol;Acc:HGNC:16844]	0.36
PMCTP8-021	3	ENSG00000211943	IGHV3-15	immunoglobulin heavy variable 3-15 [Source:HGNC Symbol;Acc:HGNC:5582]	1.05
PMCTP8-021	3	ENSG00000073756	PTGS2	prostaglandin-endoperoxide synthase 2 [Source:HGNC Symbol;Acc:HGNC:9605]	0.48
PMCTP8-022	5	ENSG00000111644	ACRBP	acrosin binding protein [Source:HGNC Symbol;Acc:HGNC:17195]	0.26
PMCTP8-022	5	ENSG00000125347	IRF1	interferon regulatory factor 1 [Source:HGNC Symbol;Acc:HGNC:6116]	0.18
PMCTP8-022	5	ENSG00000074416	MGLL	monoglyceride lipase [Source:HGNC Symbol;Acc:HGNC:17038]	0.29
PMCTP8-022	5	ENSG00000171611	PTCRA	pre T-cell antigen receptor alpha [Source:HGNC Symbol;Acc:HGNC:21290]	0.68
PMCTP8-022	5	ENSG00000149131	SERPING1	serpin family G member 1 [Source:HGNC Symbol;Acc:HGNC:1228]	0.97
PMCTP8-023	4	ENSG00000169679	BUB1	BUB1 mitotic checkpoint serine/threonine kinase [Source:HGNC Symbol;Acc:HGNC:1148]	0.23
PMCTP8-023	4	ENSG00000211592	IGKC	immunoglobulin kappa constant [Source:HGNC Symbol;Acc:HGNC:5716]	0.63

Cluster ID	Cluster Size	Gene ID	Gene Name	Gene Description	Log_2 FC TIV
PMCTP8-023	4	ENSG00000239951	IGKV3-20	immunoglobulin kappa variable 3-20 [Source:HGNC Symbol;Acc:HGNC:5817]	0.78
PMCTP8-023	4	ENSG00000183762	KREMEN1	kringle containing transmembrane protein 1 [Source:HGNC Symbol;Acc:HGNC:17550]	1.17
PMCTP8-024	6	ENSG00000163823	CCR1	C-C motif chemokine receptor 1 [Source:HGNC Symbol;Acc:HGNC:1602]	0.23
PMCTP8-024	6	ENSG00000124772	CPNE5	copine 5 [Source:HGNC Symbol;Acc:HGNC:2318]	0.71
PMCTP8-024	6	ENSG00000241351	IGKV3-11	immunoglobulin kappa variable 3-11 [Source:HGNC Symbol;Acc:HGNC:5815]	1.16
PMCTP8-024	6	ENSG00000136689	IL1RN	interleukin 1 receptor antagonist [Source:HGNC Symbol;Acc:HGNC:6000]	0.54
PMCTP8-024	6	ENSG00000110777	POU2AF1	POU class 2 associating factor 1 [Source:HGNC Symbol;Acc:HGNC:9211]	0.54
PMCTP8-024	6	ENSG00000115884	SDC1	syndecan 1 [Source:HGNC Symbol;Acc:HGNC:10658]	2.17
PMCTP8-025	2	ENSG00000168298	HIST1H1E	histone cluster 1 H1 family member e [Source:HGNC Symbol;Acc:HGNC:4718]	2.07
PMCTP8-025	2	ENSG00000242371	IGKV1-39	immunoglobulin kappa variable 1-39 (gene/pseudogene) [Source:HGNC Symbol;Acc:HGNC:5740]	0.39
PMCTP8-026	5	ENSG00000196787	HIST1H2AG	histone cluster 1 H2A family member g [Source:HGNC Symbol;Acc:HGNC:4737]	0.60
PMCTP8-026	5	ENSG00000185745	IFIT1	interferon induced protein with tetratricopeptide repeats 1 [Source:HGNC Symbol;Acc:HGNC:5407]	0.98
PMCTP8-026	5	ENSG00000119922	IFIT2	interferon induced protein with tetratricopeptide repeats 2 [Source:HGNC Symbol;Acc:HGNC:5409]	1.07
PMCTP8-026	5	ENSG00000119917	IFIT3	interferon induced protein with tetratricopeptide repeats 3 [Source:HGNC Symbol;Acc:HGNC:5411]	0.82
PMCTP8-026	5	ENSG00000239975	IGKV1D-33	immunoglobulin kappa variable 1D-33 [Source:HGNC Symbol;Acc:HGNC:5753]	1.51
PMCTP8-027	3	ENSG00000211679	IGLC3	immunoglobulin lambda constant 3 (Kern-Oz+ marker) [Source:HGNC Symbol;Acc:HGNC:5857]	1.15
PMCTP8-027	3	ENSG00000211651	IGLV1-44	immunoglobulin lambda variable 1-44 [Source:HGNC Symbol;Acc:HGNC:5879]	2.01
PMCTP8-027	3	ENSG00000004799	PDK4	pyruvate dehydrogenase kinase 4 [Source:HGNC Symbol;Acc:HGNC:8812]	0.88
PMCTP8-028	6	ENSG00000138772	ANXA3	annexin A3 [Source:HGNC Symbol;Acc:HGNC:541]	0.98
PMCTP8-028	6	ENSG00000134755	DSC2	desmocollin 2 [Source:HGNC Symbol;Acc:HGNC:3036]	0.34
PMCTP8-028	6	ENSG00000254395	IGHV4-55	immunoglobulin heavy variable 4-55 (pseudogene) [Source:HGNC Symbol;Acc:HGNC:5653]	1.25
PMCTP8-028	6	ENSG00000140749	IGSF6	immunoglobulin superfamily member 6 [Source:HGNC Symbol;Acc:HGNC:5953]	0.21
PMCTP8-028	6	ENSG00000125538	IL1B	interleukin 1 beta [Source:HGNC Symbol;Acc:HGNC:5992]	0.71
PMCTP8-028	6	ENSG00000232810	TNF	tumor necrosis factor [Source:HGNC Symbol;Acc:HGNC:11892]	0.72
PMCTP8-029	3	ENSG00000280800			2.60
PMCTP8-029	3	ENSG00000121807	CCR2	C-C motif chemokine receptor 2 [Source:HGNC Symbol;Acc:HGNC:1603]	0.04
PMCTP8-029	3	ENSG00000211644	IGLV1-51	immunoglobulin lambda variable 1-51 [Source:HGNC Symbol;Acc:HGNC:5882]	0.36
PMCTP8-030	15	ENSG00000234389			-0.53
PMCTP8-030	15	ENSG00000275302	CCL4	C-C motif chemokine ligand 4 [Source:HGNC Symbol;Acc:HGNC:10630]	-0.64
PMCTP8-030	15	ENSG00000276070	CCL4L2	C-C motif chemokine ligand 4 like 2 [Source:HGNC Symbol;Acc:HGNC:24066]	-0.55
PMCTP8-030	15	ENSG00000117281	CD160	CD160 molecule [Source:HGNC Symbol;Acc:HGNC:17013]	-0.53
PMCTP8-030	15	ENSG00000110848	CD69	CD69 molecule [Source:HGNC Symbol;Acc:HGNC:1694]	-0.58
PMCTP8-030	15	ENSG00000110318	CEP126	centrosomal protein 126 [Source:HGNC Symbol;Acc:HGNC:29264]	-0.52
PMCTP8-030	15	ENSG00000187118	CMC1	C-X9-C motif containing 1 [Source:HGNC Symbol;Acc:HGNC:28783]	-0.52
PMCTP8-030	15	ENSG00000171451	DSEL	dermatan sulfate epimerase-like [Source:HGNC Symbol;Acc:HGNC:18144]	-0.70

Cluster ID	Cluster Size	Gene ID	Gene Name	Gene Description	Log_2 FC TIV
PMCTP8-030	15	ENSG00000197057	DTHD1	death domain containing 1 [Source:HGNC Symbol;Acc:HGNC:37261]	-0.47
PMCTP8-030	15	ENSG00000137441	FGFBP2	fibroblast growth factor binding protein 2 [Source:HGNC Symbol;Acc:HGNC:29451]	-0.49
PMCTP8-030	15	ENSG00000167633	KIR3DL1	killer cell immunoglobulin like receptor, three Ig domains and long cytoplasmic tail 1 [Source:HGNC Symbol;Acc:HGNC:6338]	-0.69
PMCTP8-030	15	ENSG00000255819	KLRC4-KLRK1	KLRC4-KLRK1 readthrough [Source:HGNC Symbol;Acc:HGNC:48357]	-0.56
PMCTP8-030	15	ENSG00000170962	PDGFD	platelet derived growth factor D [Source:HGNC Symbol;Acc:HGNC:30620]	-0.68
PMCTP8-030	15	ENSG00000150687	PRSS23	protease, serine 23 [Source:HGNC Symbol;Acc:HGNC:14370]	-0.53
PMCTP8-030	15	ENSG00000171101	SIGLEC17P	sialic acid binding Ig like lectin 17, pseudogene [Source:HGNC Symbol;Acc:HGNC:15604]	-0.65

Table 45: Co-expressed gene clusters (PBMC, Day 8)

Cluster ID	Cluster Size	Gene ID	Gene Name	Gene Description	Log_2 FC TIV
PMCTP9-001	2	ENSG00000211896	IGHG1	immunoglobulin heavy constant gamma 1 (G1m marker) [Source:HGNC Symbol;Acc:HGNC:5525]	1.55
PMCTP9-001	2	ENSG00000253755	IGHGP	immunoglobulin heavy constant gamma P (non-functional) [Source:HGNC Symbol;Acc:HGNC:5529]	1.12
PMCTP9-002	3	ENSG00000158578	ALAS2	5'-aminolevulinate synthase 2 [Source:HGNC Symbol;Acc:HGNC:397]	1.24
PMCTP9-002	3	ENSG00000206172	HBA1	hemoglobin subunit alpha 1 [Source:HGNC Symbol;Acc:HGNC:4823]	0.91
PMCTP9-002	3	ENSG00000188536	HBA2	hemoglobin subunit alpha 2 [Source:HGNC Symbol;Acc:HGNC:4824]	1.00
PMCTP9-003	2	ENSG00000244734	HBB	hemoglobin subunit beta [Source:HGNC Symbol;Acc:HGNC:4827]	0.75
PMCTP9-003	2	ENSG00000169116	PARM1	prostate androgen-regulated mucin-like protein 1 [Source:HGNC Symbol;Acc:HGNC:24536]	0.31
PMCTP9-004	2	ENSG00000211677	IGLC2	immunoglobulin lambda constant 2 [Source:HGNC Symbol;Acc:HGNC:5856]	1.21
PMCTP9-004	2	ENSG00000211662	IGLV3-21	immunoglobulin lambda variable 3-21 [Source:HGNC Symbol;Acc:HGNC:5905]	1.76
PMCTP9-005	7	ENSG00000118985	ELL2	elongation factor for RNA polymerase II 2 [Source:HGNC Symbol;Acc:HGNC:17064]	0.17
PMCTP9-005	7	ENSG00000183508	FAM46C	family with sequence similarity 46 member C [Source:HGNC Symbol;Acc:HGNC:24712]	0.43
PMCTP9-005	7	ENSG00000211964	IGHV3-48	immunoglobulin heavy variable 3-48 [Source:HGNC Symbol;Acc:HGNC:5606]	0.94
PMCTP9-005	7	ENSG00000211653	IGLV1-40	immunoglobulin lambda variable 1-40 [Source:HGNC Symbol;Acc:HGNC:5877]	1.83
PMCTP9-005	7	ENSG00000211670	IGLV3-9	immunoglobulin lambda variable 3-9 (gene/pseudogene) [Source:HGNC Symbol;Acc:HGNC:5918]	1.31
PMCTP9-005	7	ENSG00000132465	JCHAIN	joining chain of multimeric IgA and IgM [Source:HGNC Symbol;Acc:HGNC:5713]	0.61
PMCTP9-005	7	ENSG00000197705	KLHL14	kelch like family member 14 [Source:HGNC Symbol;Acc:HGNC:29266]	0.35
PMCTP9-006	4	ENSG00000197561	ELANE	elastase, neutrophil expressed [Source:HGNC Symbol;Acc:HGNC:3309]	0.72
PMCTP9-006	4	ENSG00000211967	IGHV3-53	immunoglobulin heavy variable 3-53 [Source:HGNC Symbol;Acc:HGNC:5610]	0.97
PMCTP9-006	4	ENSG00000251546	IGKV1D-39	immunoglobulin kappa variable 1D-39 [Source:HGNC Symbol;Acc:HGNC:5756]	0.76
PMCTP9-006	4	ENSG00000004799	PDK4	pyruvate dehydrogenase kinase 4 [Source:HGNC Symbol;Acc:HGNC:8812]	0.93

Table 46: Co-expressed gene clusters (PBMC, Day 9)

Cluster ID	Cluster Size	Gene ID	Gene Name	Gene Description	Log_2 FC TIV
PMCTP10-001	2	ENSG00000180871	CXCR2	C-X-C motif chemokine receptor 2 [Source:HGNC Symbol;Acc:HGNC:6027]	0.97
PMCTP10-001	2	ENSG00000162747	FCGR3B	Fc fragment of IgG receptor IIIb [Source:HGNC Symbol;Acc:HGNC:3620]	1.15
PMCTP10-002	2	ENSG00000163464	CXCR1	C-X-C motif chemokine receptor 1 [Source:HGNC Symbol;Acc:HGNC:6026]	1.08
PMCTP10-002	2	ENSG00000171236	LRG1	leucine rich alpha-2-glycoprotein 1 [Source:HGNC Symbol;Acc:HGNC:29480]	0.93
PMCTP10-003	2	ENSG00000062282	DGAT2	diacylglycerol O-acyltransferase 2 [Source:HGNC Symbol;Acc:HGNC:16940]	0.64
PMCTP10-003	2	ENSG00000008516	MMP25	matrix metallopeptidase 25 [Source:HGNC Symbol;Acc:HGNC:14246]	0.80
PMCTP10-004	4	ENSG00000158578	ALAS2	5'-aminolevulinate synthase 2 [Source:HGNC Symbol;Acc:HGNC:397]	1.29
PMCTP10-004	4	ENSG00000206172	HBA1	hemoglobin subunit alpha 1 [Source:HGNC Symbol;Acc:HGNC:4823]	1.25
PMCTP10-004	4	ENSG00000188536	HBA2	hemoglobin subunit alpha 2 [Source:HGNC Symbol;Acc:HGNC:4824]	1.29
PMCTP10-004	4	ENSG00000223609	HBD	hemoglobin subunit delta [Source:HGNC Symbol;Acc:HGNC:4829]	1.11
PMCTP10-005	2	ENSG00000124772	CPNE5	copine 5 [Source:HGNC Symbol;Acc:HGNC:2318]	0.31
PMCTP10-005	2	ENSG00000211653	IGLV1-40	immunoglobulin lambda variable 1-40 [Source:HGNC Symbol;Acc:HGNC:5877]	1.57
PMCTP10-006	2	ENSG00000181409	AATK	apoptosis associated tyrosine kinase [Source:HGNC Symbol;Acc:HGNC:21]	0.68
PMCTP10-006	2	ENSG00000183762	KREMEN1	kringle containing transmembrane protein 1 [Source:HGNC Symbol;Acc:HGNC:17550]	1.10
PMCTP10-007	2	ENSG00000173868	PHOSPHO1	phosphoethanolamine/phosphocholine phosphatase [Source:HGNC Symbol;Acc:HGNC:16815]	0.76
PMCTP10-007	2	ENSG00000004939	SLC4A1	solute carrier family 4 member 1 (Diego blood group) [Source:HGNC Symbol;Acc:HGNC:11027]	1.09
PMCTP10-008	6	ENSG00000182885	ADGRG3	adhesion G protein-coupled receptor G3 [Source:HGNC Symbol;Acc:HGNC:13728]	0.91
PMCTP10-008	6	ENSG00000162551	ALPL	alkaline phosphatase, liver/bone/kidney [Source:HGNC Symbol;Acc:HGNC:438]	1.37
PMCTP10-008	6	ENSG00000126262	FFAR2	free fatty acid receptor 2 [Source:HGNC Symbol;Acc:HGNC:4501]	1.08
PMCTP10-008	6	ENSG00000244734	HBB	hemoglobin subunit beta [Source:HGNC Symbol;Acc:HGNC:4827]	1.07
PMCTP10-008	6	ENSG00000182782	HCAR2	hydroxycarboxylic acid receptor 2 [Source:HGNC Symbol;Acc:HGNC:24827]	1.08
PMCTP10-008	6	ENSG00000173535	TNFRSF10C	TNF receptor superfamily member 10c [Source:HGNC Symbol;Acc:HGNC:11906]	1.16
PMCTP10-009	4	ENSG00000186529	CYP4F3	cytochrome P450 family 4 subfamily F member 3 [Source:HGNC Symbol;Acc:HGNC:2646]	0.78
PMCTP10-009	4	ENSG00000278196	IGLV2-8	immunoglobulin lambda variable 2-8 [Source:HGNC Symbol;Acc:HGNC:5895]	0.68
PMCTP10-009	4	ENSG00000257335	MGAM	maltase-glucoamylase [Source:HGNC Symbol;Acc:HGNC:7043]	0.63
PMCTP10-009	4	ENSG00000196549	MME	membrane metalloendopeptidase [Source:HGNC Symbol;Acc:HGNC:7154]	0.87
PMCTP10-010	4	ENSG00000148926	ADM	adrenomedullin [Source:HGNC Symbol;Acc:HGNC:259]	0.75
PMCTP10-010	4	ENSG00000119922	IFIT2	interferon induced protein with tetratricopeptide repeats 2 [Source:HGNC Symbol;Acc:HGNC:5409]	0.64
PMCTP10-010	4	ENSG00000157551	KCNJ15	potassium voltage-gated channel subfamily J member 15 [Source:HGNC Symbol;Acc:HGNC:6261]	0.81
PMCTP10-010	4	ENSG00000123700	KCNJ2	potassium voltage-gated channel subfamily J member 2 [Source:HGNC Symbol;Acc:HGNC:6263]	0.58
PMCTP10-011	2	ENSG00000103569	AQP9	aquaporin 9 [Source:HGNC Symbol;Acc:HGNC:643]	0.58
PMCTP10-011	2	ENSG00000140932	CMTM2	CKLF like MARVEL transmembrane domain containing 2 [Source:HGNC Symbol;Acc:HGNC:19173]	0.94
PMCTP10-012	3	ENSG00000239839	DEFA3	defensin alpha 3 [Source:HGNC Symbol;Acc:HGNC:2762]	0.48

Cluster ID	Cluster Size	Gene ID	Gene Name	Gene Description	Log_2 FC TIV
PMCTP10-012	3	ENSG00000198814	GK	glycerol kinase [Source:HGNC Symbol;Acc:HGNC:4289]	0.12
PMCTP10-012	3	ENSG00000211670	IGLV3-9	immunoglobulin lambda variable 3-9 (gene/pseudogene) [Source:HGNC Symbol;Acc:HGNC:5918]	1.14
PMCTP10-013	4	ENSG00000123689	G0S2	G0/G1 switch 2 [Source:HGNC Symbol;Acc:HGNC:30229]	1.13
PMCTP10-013	4	ENSG00000211967	IGHV3-53	immunoglobulin heavy variable 3-53 [Source:HGNC Symbol;Acc:HGNC:5610]	0.63
PMCTP10-013	4	ENSG00000004799	PDK4	pyruvate dehydrogenase kinase 4 [Source:HGNC Symbol;Acc:HGNC:8812]	0.81
PMCTP10-013	4	ENSG00000073756	PTGS2	prostaglandin-endoperoxide synthase 2 [Source:HGNC Symbol;Acc:HGNC:9605]	0.73

Table 47: Co-expressed gene clusters (PBMC, Day 10)

Cluster ID	Cluster Size	Gene ID	Gene Name	Gene Description	Log_2 FC TIV
PMCALLTP-001	3	ENSG00000163464	CXCR1	C-X-C motif chemokine receptor 1 [Source:HGNC Symbol;Acc:HGNC:6026]	
PMCALLTP-001	3	ENSG00000180871	CXCR2	C-X-C motif chemokine receptor 2 [Source:HGNC Symbol;Acc:HGNC:6027]	
PMCALLTP-001	3	ENSG00000162747	FCGR3B	Fc fragment of IgG receptor IIIb [Source:HGNC Symbol;Acc:HGNC:3620]	
PMCALLTP-002	2	ENSG00000158517	NCF1	neutrophil cytosolic factor 1 [Source:HGNC Symbol;Acc:HGNC:7660]	
PMCALLTP-002	2	ENSG00000182487	NCF1B	neutrophil cytosolic factor 1B pseudogene [Source:HGNC Symbol;Acc:HGNC:32522]	
PMCALLTP-003	3	ENSG00000185745	IFIT1	interferon induced protein with tetratricopeptide repeats 1 [Source:HGNC Symbol;Acc:HGNC:5407]	
PMCALLTP-003	3	ENSG00000119922	IFIT2	interferon induced protein with tetratricopeptide repeats 2 [Source:HGNC Symbol;Acc:HGNC:5409]	
PMCALLTP-003	3	ENSG00000119917	IFIT3	interferon induced protein with tetratricopeptide repeats 3 [Source:HGNC Symbol;Acc:HGNC:5411]	
PMCALLTP-004	2	ENSG00000272821			
PMCALLTP-004	2	ENSG00000130489	SCO2	SCO2, cytochrome c oxidase assembly protein [Source:HGNC Symbol;Acc:HGNC:10604]	
PMCALLTP-005	2	ENSG00000140932	CMTM2	CKLF like MARVEL transmembrane domain containing 2 [Source:HGNC Symbol;Acc:HGNC:19173]	
PMCALLTP-005	2	ENSG00000196549	MME	membrane metalloendopeptidase [Source:HGNC Symbol;Acc:HGNC:7154]	
PMCALLTP-006	2	ENSG00000278828	HIST1H3H	histone cluster 1 H3 family member h [Source:HGNC Symbol;Acc:HGNC:4775]	
PMCALLTP-006	2	ENSG00000184678	HIST2H2BE	histone cluster 2 H2B family member e [Source:HGNC Symbol;Acc:HGNC:4760]	
PMCALLTP-007	4	ENSG00000275302	CCL4	C-C motif chemokine ligand 4 [Source:HGNC Symbol;Acc:HGNC:10630]	
PMCALLTP-007	4	ENSG00000117281	CD160	CD160 molecule [Source:HGNC Symbol;Acc:HGNC:17013]	
PMCALLTP-007	4	ENSG00000137441	FGFBP2	fibroblast growth factor binding protein 2 [Source:HGNC Symbol;Acc:HGNC:29451]	
PMCALLTP-007	4	ENSG00000150687	PRSS23	protease, serine 23 [Source:HGNC Symbol;Acc:HGNC:14370]	
PMCALLTP-008	2	ENSG00000211947	IGHV3-21	immunoglobulin heavy variable 3-21 [Source:HGNC Symbol;Acc:HGNC:5586]	
PMCALLTP-008	2	ENSG00000239951	IGKV3-20	immunoglobulin kappa variable 3-20 [Source:HGNC Symbol;Acc:HGNC:5817]	
PMCALLTP-009	3	ENSG00000186407	CD300E	CD300e molecule [Source:HGNC Symbol;Acc:HGNC:28874]	
PMCALLTP-009	3	ENSG00000139318	DUSP6	dual specificity phosphatase 6 [Source:HGNC Symbol;Acc:HGNC:3072]	
PMCALLTP-009	3	ENSG00000019169	MARCO	macrophage receptor with collagenous structure [Source:HGNC Symbol;Acc:HGNC:6895]	
PMCALLTP-010	7	ENSG00000211896	IGHG1	immunoglobulin heavy constant gamma 1 (G1m marker) [Source:HGNC Symbol;Acc:HGNC:5525]	

Cluster ID	Cluster Size	Gene ID	Gene Name	Gene Description	Log_2 FC TIV
PMCALLTP-010	7	ENSG00000211897	IGHG3	immunoglobulin heavy constant gamma 3 (G3m marker) [Source:HGNC Symbol;Acc:HGNC:5527]	
PMCALLTP-010	7	ENSG00000253755	IGHGP	immunoglobulin heavy constant gamma P (non-functional) [Source:HGNC Symbol;Acc:HGNC:5529]	
PMCALLTP-010	7	ENSG00000211592	IGKC	immunoglobulin kappa constant [Source:HGNC Symbol;Acc:HGNC:5716]	
PMCALLTP-010	7	ENSG00000241351	IGKV3-11	immunoglobulin kappa variable 3-11 [Source:HGNC Symbol;Acc:HGNC:5815]	
PMCALLTP-010	7	ENSG00000170476	MZB1	marginal zone B and B1 cell specific protein [Source:HGNC Symbol;Acc:HGNC:30125]	
PMCALLTP-010	7	ENSG00000048462	TNFRSF17	TNF receptor superfamily member 17 [Source:HGNC Symbol;Acc:HGNC:11913]	
PMCALLTP-011	2	ENSG00000170345	FOS	Fos proto-oncogene, AP-1 transcription factor subunit [Source:HGNC Symbol;Acc:HGNC:3796]	
PMCALLTP-011	2	ENSG00000177606	JUN	Jun proto-oncogene, AP-1 transcription factor subunit [Source:HGNC Symbol;Acc:HGNC:6204]	
PMCALLTP-012	2	ENSG00000224650	IGHV3-74	immunoglobulin heavy variable 3-74 [Source:HGNC Symbol;Acc:HGNC:5624]	
PMCALLTP-012	2	ENSG00000211644	IGLV1-51	immunoglobulin lambda variable 1-51 [Source:HGNC Symbol;Acc:HGNC:5882]	
PMCALLTP-013	3	ENSG00000123989	CHPF	chondroitin polymerizing factor [Source:HGNC Symbol;Acc:HGNC:24291]	
PMCALLTP-013	3	ENSG00000099958	DERL3	derlin 3 [Source:HGNC Symbol;Acc:HGNC:14236]	
PMCALLTP-013	3	ENSG00000242076	IGKV1-33	immunoglobulin kappa variable 1-33 [Source:HGNC Symbol;Acc:HGNC:5737]	
PMCALLTP-014	8	ENSG00000173210	ABLIM3	actin binding LIM protein family member 3 [Source:HGNC Symbol;Acc:HGNC:29132]	
PMCALLTP-014	8	ENSG00000163735	CXCL5	C-X-C motif chemokine ligand 5 [Source:HGNC Symbol;Acc:HGNC:10642]	
PMCALLTP-014	8	ENSG00000259207	ITGB3	integrin subunit beta 3 [Source:HGNC Symbol;Acc:HGNC:6156]	
PMCALLTP-014	8	ENSG00000156265	MAP3K7CL	MAP3K7 C-terminal like [Source:HGNC Symbol;Acc:HGNC:16457]	
PMCALLTP-014	8	ENSG00000163736	PPBP	pro-platelet basic protein [Source:HGNC Symbol;Acc:HGNC:9240]	
PMCALLTP-014	8	ENSG00000005249	PRKAR2B	protein kinase cAMP-dependent type II regulatory subunit beta [Source:HGNC Symbol;Acc:HGNC:9392]	
PMCALLTP-014	8	ENSG00000168497	SDPR	serum deprivation response [Source:HGNC Symbol;Acc:HGNC:10690]	
PMCALLTP-014	8	ENSG00000198478	SH3BGRL2	SH3 domain binding glutamate rich protein like 2 [Source:HGNC Symbol;Acc:HGNC:15567]	
PMCALLTP-015	2	ENSG00000110318	CEP126	centrosomal protein 126 [Source:HGNC Symbol;Acc:HGNC:29264]	
PMCALLTP-015	2	ENSG00000255819	KLRC4-KLRK1	KLRC4-KLRK1 readthrough [Source:HGNC Symbol;Acc:HGNC:48357]	
PMCALLTP-016	7	ENSG00000103313	MEFV	Mediterranean fever [Source:HGNC Symbol;Acc:HGNC:6998]	
PMCALLTP-016	7	ENSG00000020577	SAMD4A	sterile alpha motif domain containing 4A [Source:HGNC Symbol;Acc:HGNC:23023]	
PMCALLTP-016	7	ENSG00000162512	SDC3	syndecan 3 [Source:HGNC Symbol;Acc:HGNC:10660]	
PMCALLTP-016	7	ENSG00000254415	SIGLEC14	sialic acid binding Ig like lectin 14 [Source:HGNC Symbol;Acc:HGNC:32926]	
PMCALLTP-016	7	ENSG00000166002	SMCO4	single-pass membrane protein with coiled-coil domains 4 [Source:HGNC Symbol;Acc:HGNC:24810]	
PMCALLTP-016	7	ENSG00000135604	STX11	syntaxin 11 [Source:HGNC Symbol;Acc:HGNC:11429]	
PMCALLTP-016	7	ENSG00000140105	WARS	tryptophanyl-tRNA synthetase [Source:HGNC Symbol;Acc:HGNC:12729]	
PMCALLTP-017	3	ENSG00000146592	CREB5	cAMP responsive element binding protein 5 [Source:HGNC Symbol;Acc:HGNC:16844]	
PMCALLTP-017	3	ENSG00000103196	CRISPLD2	cysteine rich secretory protein LCCL domain containing 2 [Source:HGNC Symbol;Acc:HGNC:25248]	

Cluster ID	Cluster Size	Gene ID	Gene Name	Gene Description	Log_2 FC TIV
PMCALLTP-017	3	ENSG00000188906	LRRK2	leucine rich repeat kinase 2 [Source:HGNC Symbol;Acc:HGNC:18618]	
PMCALLTP-018	6	ENSG00000248996			
PMCALLTP-018	6	ENSG00000128383	APOBEC3A	apolipoprotein B mRNA editing enzyme catalytic subunit 3A [Source:HGNC Symbol;Acc:HGNC:17343]	
PMCALLTP-018	6	ENSG00000135636	DYSF	dysferlin [Source:HGNC Symbol;Acc:HGNC:3097]	
PMCALLTP-018	6	ENSG00000143226	FCGR2A	Fc fragment of IgG receptor IIa [Source:HGNC Symbol;Acc:HGNC:3616]	
PMCALLTP-018	6	ENSG00000136689	IL1RN	interleukin 1 receptor antagonist [Source:HGNC Symbol;Acc:HGNC:6000]	
PMCALLTP-018	6	ENSG00000141574	SECTM1	secreted and transmembrane 1 [Source:HGNC Symbol;Acc:HGNC:10707]	
PMCALLTP-019	5	ENSG00000158578	ALAS2	5'-aminolevulinate synthase 2 [Source:HGNC Symbol;Acc:HGNC:397]	
PMCALLTP-019	5	ENSG00000206172	HBA1	hemoglobin subunit alpha 1 [Source:HGNC Symbol;Acc:HGNC:4823]	
PMCALLTP-019	5	ENSG00000188536	HBA2	hemoglobin subunit alpha 2 [Source:HGNC Symbol;Acc:HGNC:4824]	
PMCALLTP-019	5	ENSG00000244734	HBB	hemoglobin subunit beta [Source:HGNC Symbol;Acc:HGNC:4827]	
PMCALLTP-019	5	ENSG00000004939	SLC4A1	solute carrier family 4 member 1 (Diego blood group) [Source:HGNC Symbol;Acc:HGNC:11027]	
PMCALLTP-020	4	ENSG00000241755	IGKV1-9	immunoglobulin kappa variable 1-9 [Source:HGNC Symbol;Acc:HGNC:5744]	
PMCALLTP-020	4	ENSG00000211677	IGLC2	immunoglobulin lambda constant 2 [Source:HGNC Symbol;Acc:HGNC:5856]	
PMCALLTP-020	4	ENSG00000211653	IGLV1-40	immunoglobulin lambda variable 1-40 [Source:HGNC Symbol;Acc:HGNC:5877]	
PMCALLTP-020	4	ENSG00000211662	IGLV3-21	immunoglobulin lambda variable 3-21 [Source:HGNC Symbol;Acc:HGNC:5905]	
PMCALLTP-021	9	ENSG00000178445	GLDC	glycine decarboxylase [Source:HGNC Symbol;Acc:HGNC:4313]	
PMCALLTP-021	9	ENSG00000211899	IGHM	immunoglobulin heavy constant mu [Source:HGNC Symbol;Acc:HGNC:5541]	
PMCALLTP-021	9	ENSG00000211955	IGHV3-33	immunoglobulin heavy variable 3-33 [Source:HGNC Symbol;Acc:HGNC:5596]	
PMCALLTP-021	9	ENSG00000271178	IGHV3OR16-13	immunoglobulin heavy variable 3/OR16-13 (non-functional) [Source:HGNC Symbol;Acc:HGNC:5637]	
PMCALLTP-021	9	ENSG00000211679	IGLC3	immunoglobulin lambda constant 3 (Kern-Oz+ marker) [Source:HGNC Symbol;Acc:HGNC:5857]	
PMCALLTP-021	9	ENSG00000211651	IGLV1-44	immunoglobulin lambda variable 1-44 [Source:HGNC Symbol;Acc:HGNC:5879]	
PMCALLTP-021	9	ENSG00000211666	IGLV2-14	immunoglobulin lambda variable 2-14 [Source:HGNC Symbol;Acc:HGNC:5888]	
PMCALLTP-021	9	ENSG00000253451	IGLV2-28	immunoglobulin lambda variable 2-28 (pseudogene) [Source:HGNC Symbol;Acc:HGNC:5891]	
PMCALLTP-021	9	ENSG00000101057	MYBL2	MYB proto-oncogene like 2 [Source:HGNC Symbol;Acc:HGNC:7548]	
PMCALLTP-022	12	ENSG00000143382	ADAMTSL4	ADAMTS like 4 [Source:HGNC Symbol;Acc:HGNC:19706]	
PMCALLTP-022	12	ENSG00000170458	CD14	CD14 molecule [Source:HGNC Symbol;Acc:HGNC:1628]	
PMCALLTP-022	12	ENSG00000180340	FZD2	frizzled class receptor 2 [Source:HGNC Symbol;Acc:HGNC:4040]	
PMCALLTP-022	12	ENSG00000185340	GAS2L1	growth arrest specific 2 like 1 [Source:HGNC Symbol;Acc:HGNC:16955]	
PMCALLTP-022	12	ENSG00000226091	LINC00937	long intergenic non-protein coding RNA 937 [Source:HGNC Symbol;Acc:HGNC:48629]	
PMCALLTP-022	12	ENSG00000157227	MMP14	matrix metallopeptidase 14 [Source:HGNC Symbol;Acc:HGNC:7160]	
PMCALLTP-022	12	ENSG00000235568	NFAM1	NFAT activating protein with ITAM motif 1 [Source:HGNC Symbol;Acc:HGNC:29872]	
PMCALLTP-022	12	ENSG00000177989	ODF3B	outer dense fiber of sperm tails 3B [Source:HGNC Symbol;Acc:HGNC:34388]	

Cluster ID	Cluster Size	Gene ID	Gene Name	Gene Description	Log_2 FC TIV
PMCALLTP-022	12	ENSG00000197122	SRC	SRC proto-oncogene, non-receptor tyrosine kinase [Source:HGNC Symbol;Acc:HGNC:11283]	
PMCALLTP-022	12	ENSG00000185215	TNFAIP2	TNF alpha induced protein 2 [Source:HGNC Symbol;Acc:HGNC:11895]	
PMCALLTP-022	12	ENSG00000025708	TYMP	thymidine phosphorylase [Source:HGNC Symbol;Acc:HGNC:3148]	
PMCALLTP-022	12	ENSG00000114853	ZBTB47	zinc finger and BTB domain containing 47 [Source:HGNC Symbol;Acc:HGNC:26955]	
PMCALLTP-023	6	ENSG00000163823	CCR1	C-C motif chemokine receptor 1 [Source:HGNC Symbol;Acc:HGNC:1602]	
PMCALLTP-023	6	ENSG00000150337	FCGR1A	Fc fragment of IgG receptor Ia [Source:HGNC Symbol;Acc:HGNC:3613]	
PMCALLTP-023	6	ENSG00000198019	FCGR1B	Fc fragment of IgG receptor Ib [Source:HGNC Symbol;Acc:HGNC:3614]	
PMCALLTP-023	6	ENSG00000140749	IGSF6	immunoglobulin superfamily member 6 [Source:HGNC Symbol;Acc:HGNC:5953]	
PMCALLTP-023	6	ENSG00000181631	P2RY13	purinergic receptor P2Y13 [Source:HGNC Symbol;Acc:HGNC:4537]	
PMCALLTP-023	6	ENSG00000121858	TNFSF10	tumor necrosis factor superfamily member 10 [Source:HGNC Symbol;Acc:HGNC:11925]	
PMCALLTP-024	14	ENSG00000169136	ATF5	activating transcription factor 5 [Source:HGNC Symbol;Acc:HGNC:790]	
PMCALLTP-024	14	ENSG00000168062	BATF2	basic leucine zipper ATF-like transcription factor 2 [Source:HGNC Symbol;Acc:HGNC:25163]	
PMCALLTP-024	14	ENSG00000134326	CMPK2	cytidine/uridine monophosphate kinase 2 [Source:HGNC Symbol;Acc:HGNC:27015]	
PMCALLTP-024	14	ENSG00000169245	CXCL10	C-X-C motif chemokine ligand 10 [Source:HGNC Symbol;Acc:HGNC:10637]	
PMCALLTP-024	14	ENSG00000133106	EPSTI1	epithelial stromal interaction 1 [Source:HGNC Symbol;Acc:HGNC:16465]	
PMCALLTP-024	14	ENSG00000137959	IFI44L	interferon induced protein 44 like [Source:HGNC Symbol;Acc:HGNC:17817]	
PMCALLTP-024	14	ENSG00000125347	IRF1	interferon regulatory factor 1 [Source:HGNC Symbol;Acc:HGNC:6116]	
PMCALLTP-024	14	ENSG00000002549	LAP3	leucine aminopeptidase 3 [Source:HGNC Symbol;Acc:HGNC:18449]	
PMCALLTP-024	14	ENSG00000125148	MT2A	metallothionein 2A [Source:HGNC Symbol;Acc:HGNC:7406]	
PMCALLTP-024	14	ENSG00000111331	OAS3	2'-5'-oligoadenylate synthetase 3 [Source:HGNC Symbol;Acc:HGNC:8088]	
PMCALLTP-024	14	ENSG00000138496	PARP9	poly(ADP-ribose) polymerase family member 9 [Source:HGNC Symbol;Acc:HGNC:24118]	
PMCALLTP-024	14	ENSG00000149131	SERPING1	serpin family G member 1 [Source:HGNC Symbol;Acc:HGNC:1228]	
PMCALLTP-024	14	ENSG00000115415	STAT1	signal transducer and activator of transcription 1 [Source:HGNC Symbol;Acc:HGNC:11362]	
PMCALLTP-024	14	ENSG00000156587	UBE2L6	ubiquitin conjugating enzyme E2 L6 [Source:HGNC Symbol;Acc:HGNC:12490]	
PMCALLTP-025	2	ENSG00000205846	CLEC6A	C-type lectin domain family 6 member A [Source:HGNC Symbol;Acc:HGNC:14556]	
PMCALLTP-025	2	ENSG00000225492	GBP1P1	guanylate binding protein 1 pseudogene 1 [Source:HGNC Symbol;Acc:HGNC:39561]	
PMCALLTP-026	4	ENSG00000211892	IGHG4	immunoglobulin heavy constant gamma 4 (G4m marker) [Source:HGNC Symbol;Acc:HGNC:5528]	
PMCALLTP-026	4	ENSG00000280411	IGHV1-69-2	immunoglobulin heavy variable 1-69-2 [Source:HGNC Symbol;Acc:HGNC:5562]	
PMCALLTP-026	4	ENSG00000211943	IGHV3-15	immunoglobulin heavy variable 3-15 [Source:HGNC Symbol;Acc:HGNC:5582]	
PMCALLTP-026	4	ENSG00000254395	IGHV4-55	immunoglobulin heavy variable 4-55 (pseudogene) [Source:HGNC Symbol;Acc:HGNC:5653]	
PMCALLTP-027	8	ENSG00000148926	ADM	adrenomedullin [Source:HGNC Symbol;Acc:HGNC:259]	
PMCALLTP-027	8	ENSG00000138772	ANXA3	annexin A3 [Source:HGNC Symbol;Acc:HGNC:541]	
PMCALLTP-027	8	ENSG00000103569	AQP9	aquaporin 9 [Source:HGNC Symbol;Acc:HGNC:643]	

Cluster ID	Cluster Size	Gene ID	Gene Name	Gene Description	Log_2 FC TIV
PMCALLTP-027	8	ENSG00000126262	FFAR2	free fatty acid receptor 2 [Source:HGNC Symbol;Acc:HGNC:4501]	
PMCALLTP-027	8	ENSG00000182782	HCAR2	hydroxycarboxylic acid receptor 2 [Source:HGNC Symbol;Acc:HGNC:24827]	
PMCALLTP-027	8	ENSG00000157551	KCNJ15	potassium voltage-gated channel subfamily J member 15 [Source:HGNC Symbol;Acc:HGNC:6261]	
PMCALLTP-027	8	ENSG00000171236	LRG1	leucine rich alpha-2-glycoprotein 1 [Source:HGNC Symbol;Acc:HGNC:29480]	
PMCALLTP-027	8	ENSG00000163993	S100P	S100 calcium binding protein P [Source:HGNC Symbol;Acc:HGNC:10504]	

Table 48: Co-expressed gene clusters (PBMC, All post-treatment time points)

Category Type	Categories	Distinct #Genes In Sets	Median #Genes Per Set
Blood Transcription Modules	346	2819	12
MSigDB Biological Processes	4653	15371	34
MSigDB Cellular Components	584	12118	43
MSigDB Immunological Signatures	4872	19090	199
MSigDB KEGG Pathways	186	5227	54
MSigDB Molecular Functions	929	14346	30
MSigDB Reactome Pathways	674	5792	27

Table 49: Overview of gene sets used for the enrichment analysis (RNA-Seq). Genes within gene sets are fltered to reflect only those that exist in filtered Ensembl version 87 anotations obtained using biomaRt.

Gene Set Name	Gene Set Genes #	DE Genes N (%)	Up-reg. DE Genes N (%)	Down-reg. DE Genes N (%)	Jaccard Index	P-Value	FDR Adjusted P-Value
GO INTERFERON GAMMA MEDIATED SIGNALING PATHWAY	67	3 (4.5)	3 (4.5)	0 (0)	0.045	<0.0001	0.0931
GO CELLULAR RESPONSE TO INTERFERON GAMMA	118	3 (2.5)	3 (2.5)	0 (0)	0.025	<0.0001	0.0931
GO RESPONSE TO INTERFERON GAMMA	140	3 (2.1)	3 (2.1)	0 (0)	0.021	<0.0001	0.0931
GO CYTOKINE MEDIATED SIGNALING PATHWAY	448	3 (0.7)	3 (0.7)	0 (0)	0.007	<0.0001	0.0931
GO CELLULAR RESPONSE TO CYTOKINE STIMULUS	602	3 (0.5)	3 (0.5)	0 (0)	0.005	<0.0001	0.0931

Table 50: Enriched MSigDB Biological Processes (B Cells, Trivalent Influenza Vaccine, Day 1). Results are sorted by FDR adjusted p-value and Jaccard similarity index.

Gene Set Name	Gene Set Genes #	DE Genes N (%)	Up-reg. DE Genes N (%)	Down-reg. DE Genes N (%)	Jaccard Index	P-Value	FDR Adjusted P-Value
GSE1740 MCSF VS MCSF AND IFNG DAY2 DERIVED MACROPH...	181	3 (1.7)	3 (1.7)	0 (0)	0.017	<0.0001	0.0253
GSE18791 UNSTIM VS NEWCATSLE VIRUS DC 18H DN	184	3 (1.6)	3 (1.6)	0 (0)	0.016	<0.0001	0.0253
GSE2770 IL12 AND TGFB VS IL4 TREATED ACT CD4 TCELL...	184	3 (1.6)	3 (1.6)	0 (0)	0.016	<0.0001	0.0253
GSE3400 UNTREATED VS IFNB TREATED MEF DN	184	3 (1.6)	3 (1.6)	0 (0)	0.016	<0.0001	0.0253
GSE1112 HY CD8AB VS HY CD8AA THYMOCYTE RTOC CULTUR...	189	3 (1.6)	3 (1.6)	0 (0)	0.016	<0.0001	0.0253
GSE14000 UNSTIM VS 4H LPS DC TRANSLATED RNA DN	198	3 (1.5)	3 (1.5)	0 (0)	0.015	<0.0001	0.0253
GSE24634 IL4 VS CTRL TREATED NAIVE CD4 TCELL DAY3 ...	198	3 (1.5)	3 (1.5)	0 (0)	0.015	<0.0001	0.0253
GSE1432 CTRL VS IFNG 24H MICROGLIA DN	199	3 (1.5)	3 (1.5)	0 (0)	0.015	<0.0001	0.0253
GSE41978 ID2 KO VS BIM KO KLRG1 LOW EFFECTOR CD8 T...	199	3 (1.5)	3 (1.5)	0 (0)	0.015	<0.0001	0.0253
GSE24634 IL4 VS CTRL TREATED NAIVE CD4 TCELL DAY10...	200	3 (1.5)	3 (1.5)	0 (0)	0.015	<0.0001	0.0253
GSE37533 PPARG1 FOXP3 VS FOXP3 TRANSDUCED CD4 TCEL...	200	3 (1.5)	3 (1.5)	0 (0)	0.015	<0.0001	0.0253
GSE42021 TREG PLN VS CD24INT TREG THYMUS DN	200	3 (1.5)	3 (1.5)	0 (0)	0.015	<0.0001	0.0253
GSE42021 TREG VS TCONV PLN UP	200	3 (1.5)	3 (1.5)	0 (0)	0.015	<0.0001	0.0253
GSE1432 1H VS 24H IFNG MICROGLIA DN	201	3 (1.5)	3 (1.5)	0 (0)	0.015	<0.0001	0.0253
GSE1432 1H VS 6H IFNG MICROGLIA DN	202	3 (1.5)	3 (1.5)	0 (0)	0.015	<0.0001	0.0253
GSE1432 CTRL VS IFNG 6H MICROGLIA DN	201	3 (1.5)	3 (1.5)	0 (0)	0.015	<0.0001	0.0253
GSE37533 PPARG1 FOXP3 VS PPARG2 FOXP3 TRANSDUCED C...	201	3 (1.5)	3 (1.5)	0 (0)	0.015	<0.0001	0.0253
GSE6269 FLU VS STREP PNEUMO INF PBMC UP	169	2 (1.2)	2 (1.2)	0 (0)	0.012	<0.0001	0.0253
GSE1791 CTRL VS NEUROMEDINU IN T CELL LINE 12H DN	172	2 (1.2)	2 (1.2)	0 (0)	0.012	0.0003	0.0253
GSE34156 UNTREATED VS 6H NOD2 LIGAND TREATED MONOC...	173	2 (1.2)	2 (1.2)	0 (0)	0.011	0.0002	0.0253
GSE43863 NAIVE VS MEMORY TH1 CD4 TCELL D150 LCMV U...	180	2 (1.1)	2 (1.1)	0 (0)	0.011	0.0003	0.0253
GSE2706 R848 VS LPS 2H STIM DC DN	184	2 (1.1)	2 (1.1)	0 (0)	0.011	0.0003	0.0253
GSE29617 DAY3 VS DAY7 TIV FLU VACCINE PBMC 2008 UP	186	2 (1.1)	2 (1.1)	0 (0)	0.011	0.0003	0.0253
GSE1740 UNSTIM VS IFNA STIMULATED MCSF DERIVED MAC...	186	2 (1.1)	2 (1.1)	0 (0)	0.011	0.0004	0.0253
GSE9988 LOW LPS VS CTRL TREATED MONOCYTE UP	187	2 (1.1)	2 (1.1)	0 (0)	0.011	0.0003	0.0253
GSE9988 LPS VS CTRL TREATED MONOCYTE UP	189	2 (1.1)	2 (1.1)	0 (0)	0.011	0.0002	0.0253
GSE24081 CONTROLLER VS PROGRESSOR HIV SPECIFIC CD8...	192	2 (1)	2 (1)	0 (0)	0.010	0.0003	0.0253
GSE3982 EOSINOPHIL VS NEUTROPHIL DN	192	2 (1)	2 (1)	0 (0)	0.010	0.0003	0.0253
GSE42724 NAIVE BCELL VS PLASMABLAST UP	192	2 (1)	2 (1)	0 (0)	0.010	0.0003	0.0253
GSE9960 GRAM NEG VS GRAM POS SEPSIS PBMC UP	192	2 (1)	2 (1)	0 (0)	0.010	0.0003	0.0253
GSE17974 IL4 AND ANTI IL12 VS UNTREATED 48H ACT CD...	191	2 (1)	2 (1)	0 (0)	0.010	0.0004	0.0253
GSE2706 UNSTIM VS 8H R848 DC DN	194	2 (1)	2 (1)	0 (0)	0.010	0.0003	0.0253
GSE2706 2H VS 8H R848 STIM DC DN	196	2 (1)	2 (1)	0 (0)	0.010	0.0004	0.0253
GSE14000 UNSTIM VS 4H LPS DC DN	197	2 (1)	2 (1)	0 (0)	0.010	0.0003	0.0253
GSE18791 CTRL VS NEWCASTLE VIRUS DC 6H DN	197	2 (1)	2 (1)	0 (0)	0.010	0.0003	0.0253

Gene Set Name	Gene Set Genes #	DE Genes N (%)	Up-reg. DE Genes N (%)	Down-reg. DE Genes N (%)	Jaccard Index	P-Value	FDR Adjusted P-Value
GSE22886 CD4 TCELL VS BCELL NAIVE UP	197	2 (1)	2 (1)	0 (0)	0.010	0.0003	0.0253
GSE22886 TCELL VS BCELL NAIVE UP	198	2 (1)	2 (1)	0 (0)	0.010	0.0003	0.0253
GSE18791 UNSTIM VS NEWCATSLE VIRUS DC 6H DN	198	2 (1)	2 (1)	0 (0)	0.010	0.0004	0.0253
GSE10325 MYELOID VS LUPUS MYELOID DN	199	2 (1)	2 (1)	0 (0)	0.010	0.0002	0.0253
GSE13484 12H VS 3H YF17D VACCINE STIM PBMC DN	199	2 (1)	2 (1)	0 (0)	0.010	0.0002	0.0253
GSE1460 INTRATHYMIC T PROGENITOR VS CD4 THYMOCYTE ...	199	2 (1)	2 (1)	0 (0)	0.010	0.0002	0.0253
GSE17721 LPS VS CPG 24H BMDC UP	200	2 (1)	2 (1)	0 (0)	0.010	0.0002	0.0253
GSE19888 ADENOSINE A3R INH VS TCELL MEMBRANES ACT ...	200	2 (1)	2 (1)	0 (0)	0.010	0.0002	0.0253
GSE24634 IL4 VS CTRL TREATED NAIVE CD4 TCELL DAY7 ...	199	2 (1)	2 (1)	0 (0)	0.010	0.0002	0.0253
GSE18281 CORTICAL VS MEDULLARY THYMOCYTE UP	199	2 (1)	2 (1)	0 (0)	0.010	0.0003	0.0253
GSE21360 PRIMARY VS TERTIARY MEMORY CD8 TCELL DN	199	2 (1)	2 (1)	0 (0)	0.010	0.0003	0.0253
GSE22140 GERMFREE VS SPF ARTHRITIC MOUSE CD4 TCELL...	199	2 (1)	2 (1)	0 (0)	0.010	0.0003	0.0253
GSE22196 HEALTHY VS OBESE MOUSE SKIN GAMMADELTA TC...	199	2 (1)	2 (1)	0 (0)	0.010	0.0003	0.0253
GSE22886 CTRL VS LPS 24H DC DN	200	2 (1)	2 (1)	0 (0)	0.010	0.0003	0.0253
GSE339 CD4POS VS CD4CD8DN DC UP	200	2 (1)	2 (1)	0 (0)	0.010	0.0003	0.0253

Table 51: Enriched MSigDB Immunological Signatures (B Cells, Trivalent Influenza Vaccine, Day 1). Results are sorted by FDR adjusted p-value and Jaccard similarity index. Top 50 results are listed.

Gene Set Name	Gene Set Genes #	DE Genes N (%)	Up-reg. DE Genes N (%)	Down-reg. DE Genes N (%)	Jaccard Index	P-Value	FDR Adjusted P-Value
REACTOME INTERFERON GAMMA SIGNALING	59	3 (5.1)	3 (5.1)	0 (0)	0.051	0.0002	0.0505
REACTOME INTERFERON ALPHA BETA SIGNALING	62	2 (3.2)	2 (3.2)	0 (0)	0.032	0.0003	0.0505
REACTOME INTERFERON SIGNALING	153	3 (2)	3 (2)	0 (0)	0.020	0.0002	0.0505
REACTOME CYTOKINE SIGNALING IN IMMUNE SYSTEM	265	3 (1.1)	3 (1.1)	0 (0)	0.011	0.0003	0.0505

Table 52: Enriched MSigDB Reactome Pathways (B Cells, Trivalent Influenza Vaccine, Day 1). Results are sorted by FDR adjusted p-value and Jaccard similarity index.

Gene Set Name	Gene Set Genes #	DE Genes N (%)	Up-reg. DE Genes N (%)	Down-reg. DE Genes N (%)	Jaccard Index	P-Value	FDR Adjusted P-Value
GO OXYGEN TRANSPORT	15	2 (13.3)	0 (0)	2 (13.3)	0.071	<0.0001	0.0931
GO GAS TRANSPORT	19	2 (10.5)	0 (0)	2 (10.5)	0.062	<0.0001	0.0931
GO HYDROGEN PEROXIDE CATABOLIC PROCESS	20	2 (10)	0 (0)	2 (10)	0.061	<0.0001	0.0931
GO HYDROGEN PEROXIDE METABOLIC PROCESS	30	2 (6.7)	0 (0)	2 (6.7)	0.046	<0.0001	0.0931
GO REACTIVE OXYGEN SPECIES METABOLIC PROCESS	96	3 (3.1)	0 (0)	3 (3.1)	0.028	<0.0001	0.0931

Table 53: Enriched MSigDB Biological Processes (B Cells, Trivalent Influenza Vaccine, Day 3). Results are sorted by FDR adjusted p-value and Jaccard similarity index.

Gene Set Name	Gene Set Genes #	DE Genes N (%)	Up-reg. DE Genes N (%)	Down-reg. DE Genes N (%)	Jaccard Index	P-Value	FDR Adjusted P-Value

Gene Set Name	Gene Set Genes #	DE Genes N (%)	Up-reg. DE Genes N (%)	Down-reg. DE Genes N (%)	Jaccard Index	P-Value	FDR Adjusted P-Value
GO HEMOGLOBIN COMPLEX	12	2 (16.7)	0 (0)	2 (16.7)	0.080	<0.0001	0.0584
GO ENDOCYTIC VESICLE LUMEN	17	2 (11.8)	0 (0)	2 (11.8)	0.067	0.0002	0.0584

Table 54: Enriched MSigDB Cellular Components (B Cells, Trivalent Influenza Vaccine, Day 3). Results are sorted by FDR adjusted p-value and Jaccard similarity index.

Gene Set Name	Gene Set Genes #	DE Genes N (%)	Up-reg. DE Genes N (%)	Down-reg. DE Genes N (%)	Jaccard Index	P-Value	FDR Adjusted P-Value
GSE29949 MICROGLIA VS DC BRAIN UP	199	4 (2)	1 (0.5)	3 (1.5)	0.019	<0.0001	0.0886
GSE9006 HEALTHY VS TYPE 1 DIABETES PBMC 4MONTH POS...	200	4 (2)	0 (0)	4 (2)	0.019	<0.0001	0.0886
GSE4590 SMALL VS LARGE PRE BCELL DN	155	3 (1.9)	1 (0.6)	2 (1.3)	0.018	<0.0001	0.0886
GSE36476 CTRL VS TSST ACT 40H MEMORY CD4 TCELL OLD...	194	3 (1.5)	1 (0.5)	2 (1)	0.015	0.0002	0.0886
GSE36476 CTRL VS TSST ACT 72H MEMORY CD4 TCELL OLD...	195	3 (1.5)	1 (0.5)	2 (1)	0.015	0.0002	0.0886
GSE13306 TREG VS TCONV UP	199	3 (1.5)	0 (0)	3 (1.5)	0.014	0.0002	0.0886
GSE17721 LPS VS CPG 1H BMDC DN	199	3 (1.5)	0 (0)	3 (1.5)	0.014	0.0002	0.0886
GSE14769 UNSTIM VS 40MIN LPS BMDM DN	201	3 (1.5)	0 (0)	3 (1.5)	0.014	0.0002	0.0886
GSE32986 CURDLAN HIGHDOSE VS GMCSF AND CURDLAN HIG...	201	3 (1.5)	0 (0)	3 (1.5)	0.014	0.0002	0.0886
GSE9006 TYPE 1 DIABETES AT DX VS 4MONTH POST DX PB...	201	3 (1.5)	0 (0)	3 (1.5)	0.014	0.0002	0.0886
GSE19401 NAIVE VS IMMUNIZED MOUSE PLN FOLLICULAR D...	202	3 (1.5)	0 (0)	3 (1.5)	0.014	0.0002	0.0886

Table 55: Enriched MSigDB Immunological Signatures (B Cells, Trivalent Influenza Vaccine, Day 3). Results are sorted by FDR adjusted p-value and Jaccard similarity index.

Gene Set Name	Gene Set Genes #	DE Genes N (%)	Up-reg. DE Genes N (%)	Down-reg. DE Genes N (%)	Jaccard Index	P-Value	FDR Adjusted P-Value
GO OXYGEN TRANSPORTER ACTIVITY	14	2 (14.3)	0 (0)	2 (14.3)	0.074	<0.0001	0.0929
GO OXIDOREDUCTASE ACTIVITY ACTING ON PEROXIDE AS A...	41	2 (4.9)	0 (0)	2 (4.9)	0.037	0.0002	0.0929

Table 56: Enriched MSigDB Molecular Functions (B Cells, Trivalent Influenza Vaccine, Day 3). Results are sorted by FDR adjusted p-value and Jaccard similarity index.

Gene Set Name	Gene Set Genes #	DE Genes N (%)	Up-reg. DE Genes N (%)	Down-reg. DE Genes N (%)	Jaccard Index	P-Value	FDR Adjusted P-Value
GO OXYGEN TRANSPORT	15	2 (13.3)	0 (0)	2 (13.3)	0.111	<0.0001	0.0665
GO GAS TRANSPORT	19	2 (10.5)	0 (0)	2 (10.5)	0.091	<0.0001	0.0665
GO HYDROGEN PEROXIDE CATABOLIC PROCESS	20	2 (10)	0 (0)	2 (10)	0.087	<0.0001	0.0665
GO HYDROGEN PEROXIDE METABOLIC PROCESS	30	2 (6.7)	0 (0)	2 (6.7)	0.061	<0.0001	0.0665
GO BICARBONATE TRANSPORT	44	2 (4.5)	0 (0)	2 (4.5)	0.043	<0.0001	0.0665
GO RESPONSE TO TOXIC SUBSTANCE	241	3 (1.2)	1 (0.4)	2 (0.8)	0.012	<0.0001	0.0665

Gene Set Name	Gene Set Genes #	DE Genes N (%)	Up-reg. DE Genes N (%)	Down-reg. DE Genes N (%)	Jaccard Index	P-Value	FDR Adjusted P-Value
GO RESPONSE TO INORGANIC SUBSTANCE	484	3 (0.6)	1 (0.2)	2 (0.4)	0.006	<0.0001	0.0665
GO PROTEIN HETEROOLIGOMERIZATION	113	2 (1.8)	0 (0)	2 (1.8)	0.017	0.0002	0.0931
GO RESPONSE TO REACTIVE OXYGEN SPECIES	191	3 (1.6)	1 (0.5)	2 (1)	0.015	0.0002	0.0931
GO RESPONSE TO OXIDATIVE STRESS	352	3 (0.9)	1 (0.3)	2 (0.6)	0.009	0.0002	0.0931

Table 57: Enriched MSigDB Biological Processes (B Cells, Trivalent Influenza Vaccine, Day 4). Results are sorted by FDR adjusted p-value and Jaccard similarity index.

Gene Set Name	Gene Set Genes #	DE Genes N (%)	Up-reg. DE Genes N (%)	Down-reg. DE Genes N (%)	Jaccard Index	P-Value	FDR Adjusted P-Value
GO HEMOGLOBIN COMPLEX	12	2 (16.7)	0 (0)	2 (16.7)	0.133	<0.0001	0.0584
GO ENDOCYTIC VESICLE LUMEN	17	2 (11.8)	0 (0)	2 (11.8)	0.100	0.0002	0.0584

Table 58: Enriched MSigDB Cellular Components (B Cells, Trivalent Influenza Vaccine, Day 4). Results are sorted by FDR adjusted p-value and Jaccard similarity index.

Gene Set Name	Gene Set Genes #	DE Genes N (%)	Up-reg. DE Genes N (%)	Down-reg. DE Genes N (%)	Jaccard Index	P-Value	FDR Adjusted P-Value
GO OXYGEN TRANSPORTER ACTIVITY	14	2 (14.3)	0 (0)	2 (14.3)	0.118	<0.0001	0.0464
GO OXIDOREDUCTASE ACTIVITY ACTING ON PEROXIDE AS A...	41	2 (4.9)	0 (0)	2 (4.9)	0.045	<0.0001	0.0464
GO OXYGEN BINDING	47	2 (4.3)	0 (0)	2 (4.3)	0.040	0.0002	0.0619
GO ANTIOXIDANT ACTIVITY	69	2 (2.9)	0 (0)	2 (2.9)	0.028	0.0003	0.0697

Table 59: Enriched MSigDB Molecular Functions (B Cells, Trivalent Influenza Vaccine, Day 4). Results are sorted by FDR adjusted p-value and Jaccard similarity index.

Gene Set Name	Gene Set Genes #	DE Genes N (%)	Up-reg. DE Genes N (%)	Down-reg. DE Genes N (%)	Jaccard Index	P-Value	FDR Adjusted P-Value
cell cycle (I) (M4.1)	139	57 (41)	57 (41)	0 (0)	0.277	<0.0001	0.0019
cell cycle and transcription (M4.0)	318	70 (22)	70 (22)	0 (0)	0.188	<0.0001	0.0019
PLK1 signaling events (M4.2)	32	19 (59.4)	19 (59.4)	0 (0)	0.139	<0.0001	0.0019
cell cycle (III) (M103)	51	17 (33.3)	17 (33.3)	0 (0)	0.108	<0.0001	0.0019
mitotic cell cycle in stimulated CD4 T cells (M4.5...	33	14 (42.4)	14 (42.4)	0 (0)	0.098	<0.0001	0.0019
mitotic cell division (M6)	27	12 (44.4)	12 (44.4)	0 (0)	0.086	<0.0001	0.0019
cell division (stimulated CD4+ T cells) (M46)	27	10 (37)	10 (37)	0 (0)	0.071	<0.0001	0.0019
Ran mediated mitosis (M15)	13	9 (69.2)	9 (69.2)	0 (0)	0.070	<0.0001	0.0019
mitotic cell cycle (M4.7)	20	9 (45)	9 (45)	0 (0)	0.067	<0.0001	0.0019
cell division in stimulated CD4 T cells (M4.6)	19	8 (42.1)	8 (42.1)	0 (0)	0.059	<0.0001	0.0019
mitotic cell cycle - DNA replication (M4.4)	28	8 (28.6)	8 (28.6)	0 (0)	0.056	<0.0001	0.0019
C-MYC transcriptional network (M4.12)	12	7 (58.3)	7 (58.3)	0 (0)	0.054	<0.0001	0.0019
cell cycle (II) (M4.10)	14	7 (50)	7 (50)	0 (0)	0.053	<0.0001	0.0019
E2F transcription factor network (M8)	14	7 (50)	7 (50)	0 (0)	0.053	<0.0001	0.0019
transcription regulation in cell development (M49)	45	8 (17.8)	8 (17.8)	0 (0)	0.050	<0.0001	0.0019

Gene Set Name	Gene Set Genes #	DE Genes N (%)	Up-reg. DE Genes N (%)	Down-reg. DE Genes N (%)	Jaccard Index	P-Value	FDR Adjusted P-Value
cell division - E2F transcription network (M4.8)	19	6 (31.6)	6 (31.6)	0 (0)	0.044	<0.0001	0.0019
E2F1 targets (Q3) (M10.0)	31	6 (19.4)	6 (19.4)	0 (0)	0.040	<0.0001	0.0019
E2F1 targets (Q4) (M10.1)	20	5 (25)	5 (25)	0 (0)	0.036	<0.0001	0.0019
mismatch repair (I) (M22.0)	27	6 (22.2)	6 (22.2)	0 (0)	0.041	0.0002	0.0035
mitotic cell cycle in stimulated CD4 T cells (M4.1...	11	4 (36.4)	4 (36.4)	0 (0)	0.030	0.0002	0.0035
mitotic cell cycle in stimulated CD4 T cells (M4.9...	15	4 (26.7)	4 (26.7)	0 (0)	0.030	0.0009	0.0148
Rho GTPase cycle (M4.14)	9	3 (33.3)	3 (33.3)	0 (0)	0.023	0.0021	0.033

Table 60: Enriched Blood Transcription Modules (B Cells, Trivalent Influenza Vaccine, Day 5). Results are sorted by FDR adjusted p-value and Jaccard similarity index.

Gene Set Name	Gene Set Genes #	DE Genes N (%)	Up-reg. DE Genes N (%)	Down-reg. DE Genes N (%)	Jaccard Index	P-Value	FDR Adjusted P-Value
GO SISTER CHROMATID SEGREGATION	177	26 (14.7)	26 (14.7)	0 (0)	0.095	<0.0001	0.004
GO MITOTIC SISTER CHROMATID SEGREGATION	92	18 (19.6)	18 (19.6)	0 (0)	0.091	<0.0001	0.004
GO CHROMOSOME SEGREGATION	272	32 (11.8)	32 (11.8)	0 (0)	0.088	<0.0001	0.004
GO NUCLEAR CHROMOSOME SEGREGATION	228	28 (12.3)	28 (12.3)	0 (0)	0.086	<0.0001	0.004
GO MITOTIC NUCLEAR DIVISION	362	36 (9.9)	36 (9.9)	0 (0)	0.080	<0.0001	0.004
GO REGULATION OF CHROMOSOME SEGREGATION	84	14 (16.7)	14 (16.7)	0 (0)	0.072	<0.0001	0.004
GO CELL CYCLE PHASE TRANSITION	254	25 (9.8)	25 (9.8)	0 (0)	0.071	<0.0001	0.004
GO CELL DIVISION	461	38 (8.2)	38 (8.2)	0 (0)	0.070	<0.0001	0.004
GO REGULATION OF NUCLEAR DIVISION	162	18 (11.1)	18 (11.1)	0 (0)	0.067	<0.0001	0.004
GO ORGANELLE FISSION	496	39 (7.9)	39 (7.9)	0 (0)	0.067	<0.0001	0.004
GO MITOTIC CELL CYCLE	767	55 (7.2)	55 (7.2)	0 (0)	0.066	<0.0001	0.004
GO REGULATION OF TRANSCRIPTION INVOLVED IN G1 S TR...	26	9 (34.6)	9 (34.6)	0 (0)	0.064	<0.0001	0.004
GO CELL CYCLE G1 S PHASE TRANSITION	110	14 (12.7)	14 (12.7)	0 (0)	0.064	<0.0001	0.004
GO G1 S TRANSITION OF MITOTIC CELL CYCLE	110	14 (12.7)	14 (12.7)	0 (0)	0.064	<0.0001	0.004
GO SISTER CHROMATID COHESION	111	14 (12.6)	14 (12.6)	0 (0)	0.063	<0.0001	0.004
GO CELL CYCLE CHECKPOINT	196	19 (9.7)	19 (9.7)	0 (0)	0.063	<0.0001	0.004
GO REGULATION OF SISTER CHROMATID SEGREGATION	66	11 (16.7)	11 (16.7)	0 (0)	0.061	<0.0001	0.004
GO MITOTIC SPINDLE ORGANIZATION	69	11 (15.9)	11 (15.9)	0 (0)	0.060	<0.0001	0.004
GO REGULATION OF CELL DIVISION	271	21 (7.7)	21 (7.7)	0 (0)	0.056	<0.0001	0.004
GO CHROMOSOME CONDENSATION	31	8 (25.8)	8 (25.8)	0 (0)	0.054	<0.0001	0.004
GO CELL CYCLE PROCESS	1082	62 (5.7)	61 (5.6)	1 (0.1)	0.054	<0.0001	0.004
GO DNA CONFORMATION CHANGE	270	20 (7.4)	20 (7.4)	0 (0)	0.053	<0.0001	0.004
GO ANAPHASE PROMOTING COMPLEX DEPENDENT CATABOLIC ...	77	10 (13)	10 (13)	0 (0)	0.052	<0.0001	0.004
GO REGULATION OF MICROTUBULE POLYMERIZATION OR DEP...	179	15 (8.4)	15 (8.4)	0 (0)	0.052	<0.0001	0.004
GO POSITIVE REGULATION OF MITOTIC CELL CYCLE	123	12 (9.8)	12 (9.8)	0 (0)	0.051	<0.0001	0.004
GO DNA REPLICATION	209	16 (7.7)	16 (7.7)	0 (0)	0.051	<0.0001	0.004
GO DNA INTEGRITY CHECKPOINT	148	13 (8.8)	13 (8.8)	0 (0)	0.050	<0.0001	0.004
GO POSITIVE REGULATION OF CELL CYCLE PROCESS	247	17 (6.9)	17 (6.9)	0 (0)	0.048	<0.0001	0.004
GO MITOTIC CELL CYCLE CHECKPOINT	140	12 (8.6)	12 (8.6)	0 (0)	0.048	<0.0001	0.004
GO CELL CYCLE	1316	65 (4.9)	64 (4.9)	1 (0.1)	0.047	<0.0001	0.004
GO DNA PACKAGING	191	14 (7.3)	14 (7.3)	0 (0)	0.046	<0.0001	0.004
GO REGULATION OF CELL CYCLE PROCESS	558	30 (5.4)	30 (5.4)	0 (0)	0.046	<0.0001	0.004
GO REGULATION OF MITOTIC CELL CYCLE	468	26 (5.6)	26 (5.6)	0 (0)	0.046	<0.0001	0.004

Gene Set Name	Gene Set Genes #	DE Genes N (%)	Up-reg. DE Genes N (%)	Down-reg. DE Genes N (%)	Jaccard Index	P-Value	FDR Adjusted P-Value
GO NEGATIVE REGULATION OF PROTEIN COMPLEX DISASSEM...	173	13 (7.5)	13 (7.5)	0 (0)	0.046	<0.0001	0.004
GO CYTOKINESIS	84	9 (10.7)	9 (10.7)	0 (0)	0.045	<0.0001	0.004
GO CYTOSKELETON DEPENDENT CYTOKINESIS	39	7 (17.9)	7 (17.9)	0 (0)	0.045	<0.0001	0.004
GO REGULATION OF CELL CYCLE PHASE TRANSITION	322	19 (5.9)	19 (5.9)	0 (0)	0.044	<0.0001	0.004
GO MICROTUBULE CYTOSKELETON ORGANIZATION INVOLVED ...	41	7 (17.1)	7 (17.1)	0 (0)	0.044	<0.0001	0.004
GO MITOTIC SPINDLE ASSEMBLY	41	7 (17.1)	7 (17.1)	0 (0)	0.044	<0.0001	0.004
GO MICROTUBULE CYTOSKELETON ORGANIZATION	349	20 (5.7)	20 (5.7)	0 (0)	0.044	<0.0001	0.004
GO CELL CYCLE G2 M PHASE TRANSITION	138	11 (8)	11 (8)	0 (0)	0.044	<0.0001	0.004
GO SPINDLE ASSEMBLY	70	8 (11.4)	8 (11.4)	0 (0)	0.043	<0.0001	0.004
GO REGULATION OF MICROTUBULE BASED PROCESS	244	15 (6.1)	15 (6.1)	0 (0)	0.043	<0.0001	0.004
GO REGULATION OF PROTEIN COMPLEX DISASSEMBLY	221	14 (6.3)	14 (6.3)	0 (0)	0.042	<0.0001	0.004
GO REGULATION OF PROTEASOMAL UBIQUITIN DEPENDENT P...	148	11 (7.4)	11 (7.4)	0 (0)	0.042	<0.0001	0.004
GO DNA DEPENDENT DNA REPLICATION	99	9 (9.1)	9 (9.1)	0 (0)	0.042	<0.0001	0.004
GO SPINDLE CHECKPOINT	25	6 (24)	6 (24)	0 (0)	0.042	<0.0001	0.004
GO NEGATIVE REGULATION OF CYTOSKELETON ORGANIZATIO...	224	14 (6.2)	14 (6.2)	0 (0)	0.042	<0.0001	0.004
GO NEGATIVE REGULATION OF MITOTIC CELL CYCLE	199	13 (6.5)	13 (6.5)	0 (0)	0.042	<0.0001	0.004
GO POSITIVE REGULATION OF MITOTIC NUCLEAR DIVISION	51	7 (13.7)	7 (13.7)	0 (0)	0.042	<0.0001	0.004

Table 61: Enriched MSigDB Biological Processes (B Cells, Trivalent Influenza Vaccine, Day 5). Results are sorted by FDR adjusted p-value and Jaccard similarity index. Top 50 results are listed.

Gene Set Name	Gene Set Genes #	DE Genes N (%)	Up-reg. DE Genes N (%)	Down-reg. DE Genes N (%)	Jaccard Index	P-Value	FDR Adjusted P-Value
GO CONDENSED CHROMOSOME CENTROMERIC REGION	102	17 (16.7)	17 (16.7)	0 (0)	0.081	<0.0001	0.0024
GO CONDENSED CHROMOSOME	196	24 (12.2)	24 (12.2)	0 (0)	0.081	<0.0001	0.0024
GO KINETOCHORE	120	16 (13.3)	16 (13.3)	0 (0)	0.070	<0.0001	0.0024
GO CHROMOSOME CENTROMERIC REGION	174	19 (10.9)	19 (10.9)	0 (0)	0.068	<0.0001	0.0024
GO CONDENSED NUCLEAR CHROMOSOME CENTROMERIC REGION	18	8 (44.4)	8 (44.4)	0 (0)	0.060	<0.0001	0.0024
GO CHROMOSOMAL REGION	330	25 (7.6)	25 (7.6)	0 (0)	0.058	<0.0001	0.0024
GO SPINDLE POLE	126	13 (10.3)	13 (10.3)	0 (0)	0.055	<0.0001	0.0024
GO SPINDLE	289	21 (7.3)	21 (7.3)	0 (0)	0.054	<0.0001	0.0024
GO SPINDLE MICROTUBULE	58	9 (15.5)	9 (15.5)	0 (0)	0.052	<0.0001	0.0024
GO CONDENSED NUCLEAR CHROMOSOME	86	10 (11.6)	10 (11.6)	0 (0)	0.050	<0.0001	0.0024
GO CONDENSED CHROMOSOME OUTER KINETOCHORE	12	6 (50)	6 (50)	0 (0)	0.046	<0.0001	0.0024
GO MIDBODY	132	11 (8.3)	11 (8.3)	0 (0)	0.045	<0.0001	0.0024
GO SPINDLE MIDZONE	27	6 (22.2)	6 (22.2)	0 (0)	0.041	<0.0001	0.0024
GO MITOTIC SPINDLE	55	7 (12.7)	7 (12.7)	0 (0)	0.041	<0.0001	0.0024
GO CHROMOSOME	880	39 (4.4)	39 (4.4)	0 (0)	0.040	<0.0001	0.0024
GO NUCLEAR CHROMOSOME	524	24 (4.6)	24 (4.6)	0 (0)	0.038	<0.0001	0.0024
GO MICROTUBULE	406	17 (4.2)	17 (4.2)	0 (0)	0.033	<0.0001	0.0024
GO CENTROSOME	487	19 (3.9)	19 (3.9)	0 (0)	0.032	<0.0001	0.0024
GO MICROTUBULE ORGANIZING CENTER	623	21 (3.4)	21 (3.4)	0 (0)	0.029	<0.0001	0.0024
GO MICROTUBULE CYTOSKELETON	1068	32 (3)	32 (3)	0 (0)	0.028	<0.0001	0.0024
GO REPLICATION FORK	62	5 (8.1)	5 (8.1)	0 (0)	0.028	<0.0001	0.0024
GO SUPRAMOLECULAR FIBER	671	18 (2.7)	18 (2.7)	0 (0)	0.023	<0.0001	0.0024

Gene Set Name	Gene Set Genes #	DE Genes N (%)	Up-reg. DE Genes N (%)	Down-reg. DE Genes N (%)	Jaccard Index	P-Value	FDR Adjusted P-Value
GO CYTOSKELETAL PART	1437	34 (2.4)	34 (2.4)	0 (0)	0.022	<0.0001	0.0024
GO CYTOSKELETON	1967	35 (1.8)	35 (1.8)	0 (0)	0.017	<0.0001	0.0024
GO NUCLEAR REPLICATION FORK	39	4 (10.3)	4 (10.3)	0 (0)	0.025	0.0002	0.0047
GO MICROTUBULE ASSOCIATED COMPLEX	145	7 (4.8)	7 (4.8)	0 (0)	0.027	0.0003	0.0063
GO DNA PACKAGING COMPLEX	105	5 (4.8)	5 (4.8)	0 (0)	0.022	0.0003	0.0063
GO GERM CELL NUCLEUS	19	3 (15.8)	3 (15.8)	0 (0)	0.021	0.0003	0.0063
GO PROTEIN DNA COMPLEX	172	6 (3.5)	6 (3.5)	0 (0)	0.021	0.0004	0.0081
GO ANAPHASE PROMOTING COMPLEX	22	3 (13.6)	3 (13.6)	0 (0)	0.021	0.0005	0.0097
GO KINESIN COMPLEX	54	4 (7.4)	4 (7.4)	0 (0)	0.023	0.0007	0.0132
GO CYTOPLASMIC MICROTUBULE	57	4 (7)	4 (7)	0 (0)	0.023	0.001	0.0182
GO CHROMOSOME TELOMERIC REGION	162	6 (3.7)	6 (3.7)	0 (0)	0.021	0.0011	0.0195
GO ENDOPLASMIC RETICULUM CHAPERONE COMPLEX	11	2 (18.2)	2 (18.2)	0 (0)	0.015	0.0021	0.0361
GO MCM COMPLEX	11	2 (18.2)	2 (18.2)	0 (0)	0.015	0.0024	0.04
GO NUCLEAR CHROMOSOME TELOMERIC REGION	132	5 (3.8)	5 (3.8)	0 (0)	0.020	0.0026	0.0422
GO NUCLEAR UBIQUITIN LIGASE COMPLEX	42	3 (7.1)	3 (7.1)	0 (0)	0.018	0.0032	0.0492
GO INTERCELLULAR BRIDGE	44	3 (6.8)	3 (6.8)	0 (0)	0.018	0.0032	0.0492
GO MALE GERM CELL NUCLEUS	15	2 (13.3)	2 (13.3)	0 (0)	0.015	0.0041	0.0614
GO MICROTUBULE ORGANIZING CENTER PART	144	5 (3.5)	5 (3.5)	0 (0)	0.019	0.0051	0.0745
GO CENTRIOLE	101	4 (4)	4 (4)	0 (0)	0.018	0.007	0.0997

Table 62: Enriched MSigDB Cellular Components (B Cells, Trivalent Influenza Vaccine, Day 5). Results are sorted by FDR adjusted p-value and Jaccard similarity index.

Gene Set Name	Gene Set Genes #	DE Genes N (%)	Up-reg. DE Genes N (%)	Down-reg. DE Genes N (%)	Jaccard Index	P-Value	FDR Adjusted P-Value
GSE15750 DAY6 VS DAY10 TRAF6KO EFF CD8 TCELL UP	201	48 (23.9)	48 (23.9)	0 (0)	0.173	<0.0001	0.0015
GSE15750 DAY6 VS DAY10 EFF CD8 TCELL UP	201	45 (22.4)	45 (22.4)	0 (0)	0.161	<0.0001	0.0015
GSE36476 CTRL VS TSST ACT 72H MEMORY CD4 TCELL OLD...	201	41 (20.4)	41 (20.4)	0 (0)	0.144	<0.0001	0.0015
GSE39556 CD8A DC VS NK CELL MOUSE 3H POST POLYIC I...	201	41 (20.4)	41 (20.4)	0 (0)	0.144	<0.0001	0.0015
GSE36476 CTRL VS TSST ACT 72H MEMORY CD4 TCELL YOU...	201	40 (19.9)	40 (19.9)	0 (0)	0.140	<0.0001	0.0015
GSE29614 CTRL VS DAY7 TIV FLU VACCINE PBMC DN	183	37 (20.2)	37 (20.2)	0 (0)	0.137	<0.0001	0.0015
GSE24634 TEFF VS TCONV DAY7 IN CULTURE UP	198	38 (19.2)	38 (19.2)	0 (0)	0.134	<0.0001	0.0015
GSE36476 CTRL VS TSST ACT 40H MEMORY CD4 TCELL OLD...	200	38 (19)	38 (19)	0 (0)	0.133	<0.0001	0.0015
GSE21063 WT VS NFATC1 KO 8H ANTI IGM STIM BCELL UP	199	37 (18.6)	37 (18.6)	0 (0)	0.129	<0.0001	0.0015
GSE30962 PRIMARY VS SECONDARY ACUTE LCMV INF CD8 T...	199	37 (18.6)	37 (18.6)	0 (0)	0.129	<0.0001	0.0015
GSE13547 CTRL VS ANTI IGM STIM BCELL 12H UP	182	35 (19.2)	35 (19.2)	0 (0)	0.129	<0.0001	0.0015
GOLDRATH EFF VS MEMORY CD8 TCELL UP	200	37 (18.5)	37 (18.5)	0 (0)	0.129	<0.0001	0.0015
GSE25088 WT VS STAT6 KO MACROPHAGE IL4 STIM DN	197	36 (18.3)	36 (18.3)	0 (0)	0.126	<0.0001	0.0015
GSE2405 S AUREUS VS UNTREATED NEUTROPHIL DN	198	36 (18.2)	36 (18.2)	0 (0)	0.126	<0.0001	0.0015
GSE45365 HEALTHY VS MCMV INFECTION CD11B DC DN	190	35 (18.4)	35 (18.4)	0 (0)	0.125	<0.0001	0.0015
GSE36476 CTRL VS TSST ACT 40H MEMORY CD4 TCELL YOU...	200	36 (18)	36 (18)	0 (0)	0.125	<0.0001	0.0015
GSE24634 TREG VS TCONV POST DAY7 IL4 CONVERSION UP	196	35 (17.9)	35 (17.9)	0 (0)	0.123	<0.0001	0.0015
GSE14415 NATURAL TREG VS TCONV DN	181	31 (17.1)	31 (17.1)	0 (0)	0.113	<0.0001	0.0015
GSE14415 INDUCED VS NATURAL TREG DN	178	30 (16.9)	30 (16.9)	0 (0)	0.110	<0.0001	0.0015
GSE33292 WT VS TCF1 KO DN3 THYMOCYTE DN	200	32 (16)	32 (16)	0 (0)	0.110	<0.0001	0.0015
GSE40274 CTRL VS EOS TRANSDUCED ACTIVATED CD4 TCEL...	172	29 (16.9)	29 (16.9)	0 (0)	0.109	<0.0001	0.0015

Gene Set Name	Gene Set Genes #	DE Genes N (%)	Up-reg. DE Genes N (%)	Down-reg. DE Genes N (%)	Jaccard Index	P-Value	FDR Adjusted P-Value
GSE25088 WT VS STAT6 KO MACROPHAGE DN	196	31 (15.8)	31 (15.8)	0 (0)	0.107	<0.0001	0.0015
GSE39110 DAY3 VS DAY6 POST IMMUNIZATION CD8 TCELL ...	201	31 (15.4)	31 (15.4)	0 (0)	0.105	<0.0001	0.0015
GSE24634 IL4 VS CTRL TREATED NAIVE CD4 TCELL DAY7 ...	195	30 (15.4)	30 (15.4)	0 (0)	0.104	<0.0001	0.0015
GSE13411 PLASMA CELL VS MEMORY BCELL UP	190	29 (15.3)	29 (15.3)	0 (0)	0.102	<0.0001	0.0015
GSE29614 DAY3 VS DAY7 TIV FLU VACCINE PBMC DN	179	28 (15.6)	28 (15.6)	0 (0)	0.102	<0.0001	0.0015
GSE12845 IGD POS BLOOD VS PRE GC TONSIL BCELL DN	202	30 (14.9)	29 (14.4)	1 (0.5)	0.101	<0.0001	0.0015
GSE13547 2H VS 12 H ANTI IGM STIM BCELL UP	173	26 (15)	26 (15)	0 (0)	0.096	<0.0001	0.0015
GOLDRATH NAIVE VS EFF CD8 TCELL DN	198	28 (14.1)	28 (14.1)	0 (0)	0.095	<0.0001	0.0015
GSE45365 WT VS IFNAR KO BCELL MCMV INFECTION DN	188	27 (14.4)	27 (14.4)	0 (0)	0.095	<0.0001	0.0015
GSE23568 ID3 KO VS WT CD8 TCELL UP	200	28 (14)	28 (14)	0 (0)	0.095	<0.0001	0.0015
GSE45365 WT VS IFNAR KO BCELL DN	181	25 (13.8)	25 (13.8)	0 (0)	0.089	<0.0001	0.0015
GSE14415 TCONV VS FOXP3 KO INDUCED TREG DN	183	25 (13.7)	25 (13.7)	0 (0)	0.089	<0.0001	0.0015
GSE37532 WT VS PPARG KO VISCERAL ADIPOSE TISSUE TR...	196	26 (13.3)	26 (13.3)	0 (0)	0.088	<0.0001	0.0015
GSE23568 CTRL VS ID3 TRANSDUCED CD8 TCELL DN	199	26 (13.1)	26 (13.1)	0 (0)	0.087	<0.0001	0.0015
GSE2405 HEAT KILLED LYSATE VS LIVE A PHAGOCYTOPHIL...	199	26 (13.1)	26 (13.1)	0 (0)	0.087	<0.0001	0.0015
GSE27241 WT VS RORGT KO TH17 POLARIZED CD4 TCELL U...	164	23 (14)	23 (14)	0 (0)	0.087	<0.0001	0.0015
GSE13547 CTRL VS ANTI IGM STIM BCELL 2H UP	180	24 (13.3)	24 (13.3)	0 (0)	0.086	<0.0001	0.0015
GSE29614 CTRL VS TIV FLU VACCINE PBMC 2007 DN	172	23 (13.4)	23 (13.4)	0 (0)	0.084	<0.0001	0.0015
GSE24634 TEFF VS TCONV DAY10 IN CULTURE UP	200	25 (12.5)	25 (12.5)	0 (0)	0.084	<0.0001	0.0015
GSE28726 NAIVE CD4 TCELL VS NAIVE VA24NEG NKTCELL ...	201	25 (12.4)	25 (12.4)	0 (0)	0.083	<0.0001	0.0015
KAECH DAY8 EFF VS MEMORY CD8 TCELL UP	202	25 (12.4)	25 (12.4)	0 (0)	0.083	<0.0001	0.0015
GSE21063 CTRL VS ANTI IGM STIM BCELL NFATC1 KO 8H ...	196	24 (12.2)	24 (12.2)	0 (0)	0.081	<0.0001	0.0015
GSE28726 NAIVE VS ACTIVATED CD4 TCELL DN	199	24 (12.1)	24 (12.1)	0 (0)	0.080	<0.0001	0.0015
GSE22313 HEALTHY VS SLE MOUSE CD4 TCELL DN	200	24 (12)	23 (11.5)	1 (0.5)	0.080	<0.0001	0.0015
GSE23502 WT VS HDC KO MYELOID DERIVED SUPPRESSOR C...	200	24 (12)	24 (12)	0 (0)	0.080	<0.0001	0.0015
GSE29164 CD8 TCELL VS CD8 TCELL AND IL12 TREATED M...	188	23 (12.2)	23 (12.2)	0 (0)	0.080	<0.0001	0.0015
GSE13547 CTRL VS ANTI IGM STIM ZFX KO BCELL 2H UP	162	21 (13)	21 (13)	0 (0)	0.079	<0.0001	0.0015
GSE17301 ACD3 ACD28 VS ACD3 ACD28 AND IFNA5 STIM C...	194	23 (11.9)	23 (11.9)	0 (0)	0.078	<0.0001	0.0015
GSE19941 LPS VS LPS AND IL10 STIM IL10 KO MACROPHA...	182	22 (12.1)	22 (12.1)	0 (0)	0.077	<0.0001	0.0015

Table 63: Enriched MSigDB Immunological Signatures (B Cells, Trivalent Influenza Vaccine, Day 5). Results are sorted by FDR adjusted p-value and Jaccard similarity index. Top 50 results are listed.

Gene Set Name	Gene Set Genes #	DE Genes N (%)	Up-reg. DE Genes N (%)	Down-reg. DE Genes N (%)	Jaccard Index	P-Value	FDR Adjusted P-Value
KEGG CELL CYCLE	124	18 (14.5)	18 (14.5)	0 (0)	0.078	<0.0001	0.0093
KEGG OOCYTE MEIOSIS	112	9 (8)	9 (8)	0 (0)	0.040	<0.0001	0.0093
KEGG PROGESTERONE MEDIATED OOCYTE MATURATION	87	6 (6.9)	6 (6.9)	0 (0)	0.029	0.0002	0.0124
KEGG P53 SIGNALING PATHWAY	68	5 (7.4)	4 (5.9)	1 (1.5)	0.027	0.0006	0.0223
KEGG DNA REPLICATION	36	4 (11.1)	4 (11.1)	0 (0)	0.026	0.0005	0.0223

Table 64: Enriched MSigDB KEGG Pathways (B Cells, Trivalent Influenza Vaccine, Day 5). Results are sorted by FDR adjusted p-value and Jaccard similarity index.

Gene Set Name	Gene Set Genes #	DE Genes N (%)	Up-reg. DE Genes N (%)	Down-reg. DE Genes N (%)	Jaccard Index	P-Value	FDR Adjusted P-Value
GO KINASE BINDING	608	14 (2.3)	14 (2.3)	0 (0)	0.019	<0.0001	0.0232
GO ENZYME BINDING	1744	29 (1.7)	29 (1.7)	0 (0)	0.016	<0.0001	0.0232
GO ADENYL NUCLEOTIDE BINDING	1502	24 (1.6)	24 (1.6)	0 (0)	0.015	<0.0001	0.0232
GO RIBONUCLEOTIDE BINDING	1845	28 (1.5)	28 (1.5)	0 (0)	0.014	<0.0001	0.0232
GO HISTONE KINASE ACTIVITY	19	4 (21.1)	4 (21.1)	0 (0)	0.029	0.0002	0.0372
GO DNA BINDING BENDING	20	3 (15)	3 (15)	0 (0)	0.021	0.0004	0.0619
GO DNA HELICASE ACTIVITY	53	4 (7.5)	4 (7.5)	0 (0)	0.023	0.0005	0.0664
GO MICROTUBULE BINDING	201	7 (3.5)	7 (3.5)	0 (0)	0.022	0.0006	0.0697
GO RNA DNA HYBRID RIBONUCLEASE ACTIVITY	7	2 (28.6)	2 (28.6)	0 (0)	0.015	0.0007	0.0722

Table 65: Enriched MSigDB Molecular Functions (B Cells, Trivalent Influenza Vaccine, Day 5). Results are sorted by FDR adjusted p-value and Jaccard similarity index.

Gene Set Name	Gene Set Genes #	DE Genes N (%)	Up-reg. DE Genes N (%)	Down-reg. DE Genes N (%)	Jaccard Index	P-Value	FDR Adjusted P-Value
REACTOME CELL CYCLE MITOTIC	308	32 (10.4)	32 (10.4)	0 (0)	0.080	<0.0001	0.0024
REACTOME DNA REPLICATION	188	22 (11.7)	22 (11.7)	0 (0)	0.076	<0.0001	0.0024
REACTOME CELL CYCLE	400	36 (9)	36 (9)	0 (0)	0.074	<0.0001	0.0024
REACTOME MITOTIC M M G1 PHASES	168	20 (11.9)	20 (11.9)	0 (0)	0.073	<0.0001	0.0024
REACTOME G1 S SPECIFIC TRANSCRIPTION	17	9 (52.9)	9 (52.9)	0 (0)	0.068	<0.0001	0.0024
REACTOME E2F MEDIATED REGULATION OF DNA REPLICATIO...	33	10 (30.3)	10 (30.3)	0 (0)	0.068	<0.0001	0.0024
REACTOME MITOTIC G1 G1 S PHASES	130	15 (11.5)	15 (11.5)	0 (0)	0.063	<0.0001	0.0024
REACTOME MITOTIC PROMETAPHASE	86	12 (14)	12 (14)	0 (0)	0.061	<0.0001	0.0024
REACTOME G1 S TRANSITION	106	12 (11.3)	12 (11.3)	0 (0)	0.055	<0.0001	0.0024
REACTOME G2 M CHECKPOINTS	42	8 (19)	8 (19)	0 (0)	0.051	<0.0001	0.0024
REACTOME CELL CYCLE CHECKPOINTS	112	11 (9.8)	11 (9.8)	0 (0)	0.049	<0.0001	0.0024
REACTOME REGULATION OF MITOTIC CELL CYCLE	77	9 (11.7)	9 (11.7)	0 (0)	0.047	<0.0001	0.0024
REACTOME S PHASE	106	9 (8.5)	9 (8.5)	0 (0)	0.041	<0.0001	0.0024
REACTOME ACTIVATION OF THE PRE REPLICATIVE COMPLEX	30	6 (20)	6 (20)	0 (0)	0.041	<0.0001	0.0024
REACTOME ACTIVATION OF ATR IN RESPONSE TO REPLICAT...	36	6 (16.7)	6 (16.7)	0 (0)	0.039	<0.0001	0.0024
REACTOME SYNTHESIS OF DNA	90	8 (8.9)	8 (8.9)	0 (0)	0.039	<0.0001	0.0024
REACTOME M G1 TRANSITION	78	7 (9)	7 (9)	0 (0)	0.036	<0.0001	0.0024
REACTOME G0 AND EARLY G1	23	5 (21.7)	5 (21.7)	0 (0)	0.035	<0.0001	0.0024
REACTOME DNA STRAND ELONGATION	30	5 (16.7)	5 (16.7)	0 (0)	0.034	<0.0001	0.0024
REACTOME ASSEMBLY OF THE PRE REPLICATIVE COMPLEX	63	6 (9.5)	6 (9.5)	0 (0)	0.033	<0.0001	0.0024
REACTOME APC C CDC20 MEDIATED DEGRADATION OF MITOT...	65	6 (9.2)	6 (9.2)	0 (0)	0.033	<0.0001	0.0024
REACTOME E2F ENABLED INHIBITION OF PRE REPLICATION...	10	4 (40)	4 (40)	0 (0)	0.031	<0.0001	0.0024
REACTOME UNWINDING OF DNA	11	4 (36.4)	4 (36.4)	0 (0)	0.030	<0.0001	0.0024
REACTOME ASSOCIATION OF LICENSING FACTORS WITH THE...	13	4 (30.8)	4 (30.8)	0 (0)	0.030	<0.0001	0.0024
REACTOME CYCLIN A B1 ASSOCIATED EVENTS DURING G2 M...	15	4 (26.7)	4 (26.7)	0 (0)	0.030	<0.0001	0.0024
REACTOME APC CDC20 MEDIATED DEGRADATION OF NEK2A	21	4 (19)	4 (19)	0 (0)	0.028	<0.0001	0.0024
REACTOME DEPOSITION OF NEW CENPA CONTAINING NUCLEO...	62	5 (8.1)	5 (8.1)	0 (0)	0.028	<0.0001	0.0024
REACTOME CDC6 ASSOCIATION WITH THE ORC ORIGIN COMP...	11	3 (27.3)	3 (27.3)	0 (0)	0.023	<0.0001	0.0024
REACTOME CHROMOSOME MAINTENANCE	118	7 (5.9)	7 (5.9)	0 (0)	0.030	0.0002	0.0043
REACTOME APC C CDH1 MEDIATED DEGRADATION OF CDC20 ...	64	5 (7.8)	5 (7.8)	0 (0)	0.027	0.0002	0.0043
REACTOME PHOSPHORYLATION OF THE APC C	17	3 (17.6)	3 (17.6)	0 (0)	0.022	0.0002	0.0043

Gene Set Name	Gene Set Genes #	DE Genes N (%)	Up-reg. DE Genes N (%)	Down-reg. DE Genes N (%)	Jaccard Index	P-Value	FDR Adjusted P-Value
REACTOME ORC1 REMOVAL FROM CHROMATIN	65	5 (7.7)	5 (7.7)	0 (0)	0.027	0.0003	0.0063
REACTOME APC C CDC20 MEDIATED DEGRADATION OF CYCLI...	19	3 (15.8)	3 (15.8)	0 (0)	0.021	0.0006	0.0123
REACTOME INHIBITION OF THE PROTEOLYTIC ACTIVITY OF...	18	3 (16.7)	3 (16.7)	0 (0)	0.022	0.0007	0.0139
REACTOME MITOTIC G2 G2 M PHASES	78	5 (6.4)	5 (6.4)	0 (0)	0.025	0.0014	0.027
REACTOME ACTIVATION OF CHAPERONE GENES BY ATF6 ALP...	9	2 (22.2)	2 (22.2)	0 (0)	0.015	0.0029	0.0543
REACTOME RECRUITMENT OF NUMA TO MITOTIC CENTROSOME...	10	2 (20)	2 (20)	0 (0)	0.015	0.0046	0.0812
REACTOME ACTIVATION OF CHAPERONES BY ATF6 ALPHA	11	2 (18.2)	2 (18.2)	0 (0)	0.015	0.0046	0.0812
REACTOME HORMONE SENSITIVE LIPASE HSL MEDIATED TRI...	12	2 (16.7)	2 (16.7)	0 (0)	0.015	0.0047	0.0812
REACTOME G2 M DNA DAMAGE CHECKPOINT	10	2 (20)	2 (20)	0 (0)	0.015	0.005	0.0842
REACTOME RECRUITMENT OF MITOTIC CENTROSOME PROTEIN...	63	4 (6.3)	4 (6.3)	0 (0)	0.022	0.0056	0.092

Table 66: Enriched MSigDB Reactome Pathways (B Cells, Trivalent Influenza Vaccine, Day 5). Results are sorted by FDR adjusted p-value and Jaccard similarity index.

Gene Set Name	Gene Set Genes #	DE Genes N (%)	Up-reg. DE Genes N (%)	Down-reg. DE Genes N (%)	Jaccard Index	P-Value	FDR Adjusted P-Value
cell cycle and transcription (M4.0)	318	98 (30.8)	98 (30.8)	0 (0)	0.139	<0.0001	0.0018
cell cycle (I) (M4.1)	139	74 (53.2)	74 (53.2)	0 (0)	0.135	<0.0001	0.0018
PLK1 signaling events (M4.2)	32	23 (71.9)	23 (71.9)	0 (0)	0.047	<0.0001	0.0018
cell cycle (III) (M103)	51	20 (39.2)	20 (39.2)	0 (0)	0.039	<0.0001	0.0018
mitotic cell cycle in stimulated CD4 T cells (M4.5...	33	18 (54.5)	18 (54.5)	0 (0)	0.036	<0.0001	0.0018
cell division (stimulated CD4+ T cells) (M46)	27	15 (55.6)	15 (55.6)	0 (0)	0.030	<0.0001	0.0018
mitotic cell division (M6)	27	15 (55.6)	15 (55.6)	0 (0)	0.030	<0.0001	0.0018
E2F transcription factor network (M8)	14	12 (85.7)	12 (85.7)	0 (0)	0.025	<0.0001	0.0018
mitotic cell cycle (M4.7)	20	12 (60)	12 (60)	0 (0)	0.024	<0.0001	0.0018
cell cycle (II) (M4.10)	14	11 (78.6)	11 (78.6)	0 (0)	0.023	<0.0001	0.0018
cell division in stimulated CD4 T cells (M4.6)	19	11 (57.9)	11 (57.9)	0 (0)	0.022	<0.0001	0.0018
mitotic cell cycle in stimulated CD4 T cells (M4.9...	15	10 (66.7)	10 (66.7)	0 (0)	0.021	<0.0001	0.0018
Plasma cell surface signature (S3)	23	10 (43.5)	10 (43.5)	0 (0)	0.020	<0.0001	0.0018
mitotic cell cycle - DNA replication (M4.4)	28	10 (35.7)	10 (35.7)	0 (0)	0.020	<0.0001	0.0018
C-MYC transcriptional network (M4.12)	12	9 (75)	9 (75)	0 (0)	0.018	<0.0001	0.0018
Ran mediated mitosis (M15)	13	9 (69.2)	9 (69.2)	0 (0)	0.018	<0.0001	0.0018
E2F1 targets (Q3) (M10.0)	31	8 (25.8)	8 (25.8)	0 (0)	0.016	<0.0001	0.0018
mitotic cell cycle in stimulated CD4 T cells (M4.1...	11	6 (54.5)	6 (54.5)	0 (0)	0.012	<0.0001	0.0018
plasma cells, immunoglobulins (M156.1)	22	6 (27.3)	6 (27.3)	0 (0)	0.012	<0.0001	0.0018
transcription regulation in cell development (M49)	45	10 (22.2)	10 (22.2)	0 (0)	0.019	0.0003	0.0052
Rho GTPase cycle (M4.14)	9	5 (55.6)	5 (55.6)	0 (0)	0.010	0.0004	0.0066
mismatch repair (I) (M22.0)	27	6 (22.2)	6 (22.2)	0 (0)	0.012	0.0017	0.0267
plasma cells and B cells, immunoglobulins (M156.0)	30	5 (16.7)	5 (16.7)	0 (0)	0.010	0.0046	0.0692

Table 67: Enriched Blood Transcription Modules (B Cells, Trivalent Influenza Vaccine, Day 6). Results are sorted by FDR adjusted p-value and Jaccard similarity index.

Gene Set Name	Gene Set Genes #	DE Genes N (%)	Up-reg. DE Genes N (%)	Down-reg. DE Genes N (%)	Jaccard Index	P-Value	FDR Adjusted P-Value
GO MITOTIC CELL CYCLE	767	81 (10.6)	81 (10.6)	0 (0)	0.069	<0.0001	0.004
GO CELL CYCLE PROCESS	1082	93 (8.6)	92 (8.5)	1 (0.1)	0.063	<0.0001	0.004
GO MITOTIC NUCLEAR DIVISION	362	48 (13.3)	48 (13.3)	0 (0)	0.060	<0.0001	0.004
GO CELL CYCLE	1316	101 (7.7)	100 (7.6)	1 (0.1)	0.059	<0.0001	0.004
GO CHROMOSOME SEGREGATION	272	42 (15.4)	42 (15.4)	0 (0)	0.059	<0.0001	0.004
GO ORGANELLE FISSION	496	53 (10.7)	53 (10.7)	0 (0)	0.057	<0.0001	0.004
GO RESPONSE TO ENDOPLASMIC RETICULUM STRESS	234	38 (16.2)	38 (16.2)	0 (0)	0.056	<0.0001	0.004
GO CELL DIVISION	461	50 (10.8)	50 (10.8)	0 (0)	0.056	<0.0001	0.004
GO SISTER CHROMATID SEGREGATION	177	34 (19.2)	34 (19.2)	0 (0)	0.054	<0.0001	0.004
GO NUCLEAR CHROMOSOME SEGREGATION	228	36 (15.8)	36 (15.8)	0 (0)	0.053	<0.0001	0.004
GO REGULATION OF CELL CYCLE	948	66 (7)	65 (6.9)	1 (0.1)	0.048	<0.0001	0.004
GO CELL CYCLE PHASE TRANSITION	254	32 (12.6)	32 (12.6)	0 (0)	0.045	<0.0001	0.004
GO RESPONSE TO TOPOLOGICALLY INCORRECT PROTEIN	164	28 (17.1)	28 (17.1)	0 (0)	0.045	<0.0001	0.004
GO CELLULAR RESPONSE TO TOPOLOGICALLY INCORRECT PR...	123	26 (21.1)	26 (21.1)	0 (0)	0.045	<0.0001	0.004
GO CELLULAR RESPONSE TO STRESS	1563	84 (5.4)	82 (5.2)	2 (0.1)	0.043	<0.0001	0.004
GO REGULATION OF CELL CYCLE PROCESS	558	42 (7.5)	42 (7.5)	0 (0)	0.042	<0.0001	0.004
GO REGULATION OF MITOTIC CELL CYCLE	468	37 (7.9)	37 (7.9)	0 (0)	0.041	<0.0001	0.004
GO MICROTUBULE CYTOSKELETON ORGANIZATION	349	32 (9.2)	32 (9.2)	0 (0)	0.040	<0.0001	0.004
GO MITOTIC SISTER CHROMATID SEGREGATION	92	22 (23.9)	22 (23.9)	0 (0)	0.040	<0.0001	0.004
GO CARBOHYDRATE DERIVATIVE BIOSYNTHETIC PROCESS	592	40 (6.8)	40 (6.8)	0 (0)	0.039	<0.0001	0.004
GO REGULATION OF CELL DIVISION	271	28 (10.3)	28 (10.3)	0 (0)	0.039	<0.0001	0.004
GO NEGATIVE REGULATION OF CELL CYCLE	433	34 (7.9)	33 (7.6)	1 (0.2)	0.038	<0.0001	0.004
GO IRE1 MEDIATED UNFOLDED PROTEIN RESPONSE	57	20 (35.1)	20 (35.1)	0 (0)	0.038	<0.0001	0.004
GO MICROTUBULE BASED PROCESS	522	37 (7.1)	37 (7.1)	0 (0)	0.038	<0.0001	0.004
GO ORGANELLE LOCALIZATION	415	33 (8)	33 (8)	0 (0)	0.038	<0.0001	0.004
GO ESTABLISHMENT OF LOCALIZATION IN CELL	1676	79 (4.7)	79 (4.7)	0 (0)	0.038	<0.0001	0.004
GO CHROMOSOME ORGANIZATION	1007	53 (5.3)	53 (5.3)	0 (0)	0.037	<0.0001	0.004
GO CELL CYCLE CHECKPOINT	196	24 (12.2)	24 (12.2)	0 (0)	0.037	<0.0001	0.004
GO PROTEASOMAL PROTEIN CATABOLIC PROCESS	272	26 (9.6)	26 (9.6)	0 (0)	0.036	<0.0001	0.004
GO REGULATION OF NUCLEAR DIVISION	162	22 (13.6)	22 (13.6)	0 (0)	0.035	<0.0001	0.004
GO SINGLE ORGANISM CELLULAR LOCALIZATION	898	47 (5.2)	47 (5.2)	0 (0)	0.035	<0.0001	0.004
GO GLYCOPROTEIN METABOLIC PROCESS	353	28 (7.9)	28 (7.9)	0 (0)	0.035	<0.0001	0.004
GO REGULATION OF CELL CYCLE PHASE TRANSITION	322	27 (8.4)	27 (8.4)	0 (0)	0.035	<0.0001	0.004
GO DNA METABOLIC PROCESS	751	41 (5.5)	40 (5.3)	1 (0.1)	0.034	<0.0001	0.004
GO CELLULAR MACROMOLECULE LOCALIZATION	1237	57 (4.6)	57 (4.6)	0 (0)	0.034	<0.0001	0.004
GO REGULATION OF MICROTUBULE BASED PROCESS	244	24 (9.8)	24 (9.8)	0 (0)	0.034	<0.0001	0.004
GO GOLGI VESICLE TRANSPORT	321	26 (8.1)	26 (8.1)	0 (0)	0.033	<0.0001	0.004
GO PROTEIN LOCALIZATION	1814	74 (4.1)	74 (4.1)	0 (0)	0.033	<0.0001	0.004
GO CARBOHYDRATE DERIVATIVE METABOLIC PROCESS	1043	49 (4.7)	49 (4.7)	0 (0)	0.033	<0.0001	0.004
GO GLYCOSYLATION	263	24 (9.1)	24 (9.1)	0 (0)	0.033	<0.0001	0.004
GO PROTEIN COMPLEX SUBUNIT ORGANIZATION	1523	64 (4.2)	63 (4.1)	1 (0.1)	0.033	<0.0001	0.004
GO SISTER CHROMATID COHESION	111	19 (17.1)	19 (17.1)	0 (0)	0.033	<0.0001	0.004
GO DNA REPLICATION	209	22 (10.5)	22 (10.5)	0 (0)	0.033	<0.0001	0.004
GO REGULATION OF MICROTUBULE POLYMERIZATION OR DEP...	179	21 (11.7)	21 (11.7)	0 (0)	0.033	<0.0001	0.004
GO INTRACELLULAR PROTEIN TRANSPORT	780	40 (5.1)	40 (5.1)	0 (0)	0.033	<0.0001	0.004
GO POSITIVE REGULATION OF CELL CYCLE PROCESS	247	23 (9.3)	23 (9.3)	0 (0)	0.033	<0.0001	0.004
GO PROTEIN FOLDING	217	22 (10.1)	22 (10.1)	0 (0)	0.032	<0.0001	0.004

Gene Set Name	Gene Set Genes #	DE Genes N (%)	Up-reg. DE Genes N (%)	Down-reg. DE Genes N (%)	Jaccard Index	P-Value	FDR Adjusted P-Value
GO REGULATION OF ORGANELLE ORGANIZATION	1180	52 (4.4)	52 (4.4)	0 (0)	0.032	<0.0001	0.004
GO PROTEIN COMPLEX ASSEMBLY	1132	50 (4.4)	50 (4.4)	0 (0)	0.032	<0.0001	0.004
GO PROTEIN COMPLEX BIOGENESIS	1132	50 (4.4)	50 (4.4)	0 (0)	0.032	<0.0001	0.004

Table 68: Enriched MSigDB Biological Processes (B Cells, Trivalent Influenza Vaccine, Day 6). Results are sorted by FDR adjusted p-value and Jaccard similarity index. Top 50 results are listed.

Gene Set Name	Gene Set Genes #	DE Genes N (%)	Up-reg. DE Genes N (%)	Down-reg. DE Genes N (%)	Jaccard Index	P-Value	FDR Adjusted P-Value
GO ENDOPLASMIC RETICULUM PART	1162	101 (8.7)	101 (8.7)	0 (0)	0.065	<0.0001	0.0019
GO NUCLEAR OUTER MEMBRANE ENDOPLASMIC RETICULUM ME...	1005	85 (8.5)	85 (8.5)	0 (0)	0.061	<0.0001	0.0019
GO ENDOPLASMIC RETICULUM	1631	111 (6.8)	111 (6.8)	0 (0)	0.055	<0.0001	0.0019
GO CONDENSED CHROMOSOME	196	29 (14.8)	29 (14.8)	0 (0)	0.045	<0.0001	0.0019
GO CHROMOSOME	880	56 (6.4)	56 (6.4)	0 (0)	0.043	<0.0001	0.0019
GO ENDOPLASMIC RETICULUM LUMEN	202	27 (13.4)	27 (13.4)	0 (0)	0.041	<0.0001	0.0019
GO SPINDLE	289	30 (10.4)	30 (10.4)	0 (0)	0.040	<0.0001	0.0019
GO CHROMOSOMAL REGION	330	31 (9.4)	31 (9.4)	0 (0)	0.040	<0.0001	0.0019
GO CHROMOSOME CENTROMERIC REGION	174	25 (14.4)	25 (14.4)	0 (0)	0.040	<0.0001	0.0019
GO CONDENSED CHROMOSOME CENTROMERIC REGION	102	22 (21.6)	22 (21.6)	0 (0)	0.039	<0.0001	0.0019
GO MICROTUBULE CYTOSKELETON	1068	57 (5.3)	57 (5.3)	0 (0)	0.038	<0.0001	0.0019
GO KINETOCHORE	120	22 (18.3)	22 (18.3)	0 (0)	0.038	<0.0001	0.0019
GO CENTROSOME	487	32 (6.6)	32 (6.6)	0 (0)	0.034	<0.0001	0.0019
GO MICROTUBULE ORGANIZING CENTER	623	36 (5.8)	36 (5.8)	0 (0)	0.034	<0.0001	0.0019
GO NUCLEAR CHROMOSOME	524	29 (5.5)	29 (5.5)	0 (0)	0.030	<0.0001	0.0019
GO MIDBODY	132	17 (12.9)	17 (12.9)	0 (0)	0.028	<0.0001	0.0019
GO PIGMENT GRANULE	102	16 (15.7)	16 (15.7)	0 (0)	0.028	<0.0001	0.0019
GO ENDOPLASMIC RETICULUM GOLGI INTERMEDIATE COMPAR...	106	16 (15.1)	16 (15.1)	0 (0)	0.028	<0.0001	0.0019
GO SPINDLE POLE	126	16 (12.7)	16 (12.7)	0 (0)	0.027	<0.0001	0.0019
GO ROUGH ENDOPLASMIC RETICULUM	71	14 (19.7)	14 (19.7)	0 (0)	0.026	<0.0001	0.0019
GO INTRINSIC COMPONENT OF ENDOPLASMIC RETICULUM ME...	135	14 (10.4)	14 (10.4)	0 (0)	0.023	<0.0001	0.0019
GO MICROTUBULE ASSOCIATED COMPLEX	145	14 (9.7)	14 (9.7)	0 (0)	0.023	<0.0001	0.0019
GO KINESIN COMPLEX	54	11 (20.4)	11 (20.4)	0 (0)	0.021	<0.0001	0.0019
GO SPINDLE MICROTUBULE	58	11 (19)	11 (19)	0 (0)	0.021	<0.0001	0.0019
GO CONDENSED NUCLEAR CHROMOSOME	86	11 (12.8)	11 (12.8)	0 (0)	0.020	<0.0001	0.0019
GO ENDOPLASMIC RETICULUM CHAPERONE COMPLEX	11	9 (81.8)	9 (81.8)	0 (0)	0.019	<0.0001	0.0019
GO ER TO GOLGI TRANSPORT VESICLE MEMBRANE	53	9 (17)	9 (17)	0 (0)	0.017	<0.0001	0.0019
GO CONDENSED CHROMOSOME OUTER KINETOCHORE	12	8 (66.7)	8 (66.7)	0 (0)	0.016	<0.0001	0.0019
GO CONDENSED NUCLEAR CHROMOSOME CENTROMERIC REGION	18	8 (44.4)	8 (44.4)	0 (0)	0.016	<0.0001	0.0019
GO SMOOTH ENDOPLASMIC RETICULUM	33	8 (24.2)	8 (24.2)	0 (0)	0.016	<0.0001	0.0019
GO SPINDLE MIDZONE	27	7 (25.9)	7 (25.9)	0 (0)	0.014	<0.0001	0.0019
GO MICROTUBULE	406	27 (6.7)	27 (6.7)	0 (0)	0.031	0.0002	0.0032
GO ER TO GOLGI TRANSPORT VESICLE	69	10 (14.5)	10 (14.5)	0 (0)	0.018	0.0002	0.0032
GO ENDOPLASMIC RETICULUM GOLGI INTERMEDIATE COMPAR...	64	9 (14.1)	9 (14.1)	0 (0)	0.017	0.0002	0.0032
GO MITOTIC SPINDLE	55	8 (14.5)	8 (14.5)	0 (0)	0.015	0.0002	0.0032
GO ROUGH ENDOPLASMIC RETICULUM MEMBRANE	21	6 (28.6)	6 (28.6)	0 (0)	0.012	0.0002	0.0032

Gene Set Name	Gene Set Genes #	DE Genes N (%)	Up-reg. DE Genes N (%)	Down-reg. DE Genes N (%)	Jaccard Index	P-Value	FDR Adjusted P-Value
GO GOLGI MEMBRANE	703	37 (5.3)	37 (5.3)	0 (0)	0.032	0.0003	0.0047
GO ENDOCYTIC VESICLE LUMEN	17	4 (23.5)	4 (23.5)	0 (0)	0.008	0.0004	0.0061
GO ENDOPLASMIC RETICULUM QUALITY CONTROL COMPARTME...	13	4 (30.8)	4 (30.8)	0 (0)	0.008	0.0005	0.0075
GO CYTOSKELETAL PART	1437	58 (4)	58 (4)	0 (0)	0.031	0.0006	0.0085
GO PRONUCLEUS	15	4 (26.7)	4 (26.7)	0 (0)	0.008	0.0006	0.0085
GO GOLGI APPARATUS PART	890	41 (4.6)	41 (4.6)	0 (0)	0.031	0.0009	0.0122
GO CYTOPLASMIC MICROTUBULE	57	7 (12.3)	7 (12.3)	0 (0)	0.013	0.0009	0.0122
GO COATED VESICLE MEMBRANE	137	11 (8)	11 (8)	0 (0)	0.018	0.001	0.0133
GO GOLGI APPARATUS	1445	58 (4)	58 (4)	0 (0)	0.031	0.0013	0.0169
GO COATED VESICLE	233	15 (6.4)	15 (6.4)	0 (0)	0.021	0.0014	0.0178
GO INTRACELLULAR VESICLE	1258	50 (4)	49 (3.9)	1 (0.1)	0.030	0.0016	0.0199
GO SUPRAMOLECULAR FIBER	671	29 (4.3)	29 (4.3)	0 (0)	0.026	0.0019	0.0231
GO COPI COATED VESICLE	23	4 (17.4)	4 (17.4)	0 (0)	0.008	0.0039	0.0465
GO MICROTUBULE END	22	4 (18.2)	4 (18.2)	0 (0)	0.008	0.0043	0.0502

Table 69: Enriched MSigDB Cellular Components (B Cells, Trivalent Influenza Vaccine, Day 6). Results are sorted by FDR adjusted p-value and Jaccard similarity index. Top 50 results are listed.

Gene Set Name	Gene Set Genes #	DE Genes N (%)	Up-reg. DE Genes N (%)	Down-reg. DE Genes N (%)	Jaccard Index	P-Value	FDR Adjusted P-Value
GSE29614 CTRL VS DAY7 TIV FLU VACCINE PBMC DN	183	99 (54.1)	99 (54.1)	0 (0)	0.175	<0.0001	0.0012
GSE29614 DAY3 VS DAY7 TIV FLU VACCINE PBMC DN	179	89 (49.7)	89 (49.7)	0 (0)	0.155	<0.0001	0.0012
GSE29164 CD8 TCELL VS CD8 TCELL AND IL12 TREATED M...	188	72 (38.3)	72 (38.3)	0 (0)	0.120	<0.0001	0.0012
GSE13411 PLASMA CELL VS MEMORY BCELL UP	190	70 (36.8)	70 (36.8)	0 (0)	0.116	<0.0001	0.0012
GSE15750 DAY6 VS DAY10 EFF CD8 TCELL UP	201	71 (35.3)	71 (35.3)	0 (0)	0.116	<0.0001	0.0012
GSE15750 DAY6 VS DAY10 TRAF6KO EFF CD8 TCELL UP	201	66 (32.8)	66 (32.8)	0 (0)	0.107	<0.0001	0.0012
GSE22886 NAIVE BCELL VS BLOOD PLASMA CELL DN	194	62 (32)	62 (32)	0 (0)	0.101	<0.0001	0.0012
GSE30962 PRIMARY VS SECONDARY ACUTE LCMV INF CD8 T...	199	62 (31.2)	62 (31.2)	0 (0)	0.100	<0.0001	0.0012
GSE36476 CTRL VS TSST ACT 72H MEMORY CD4 TCELL YOU...	201	60 (29.9)	60 (29.9)	0 (0)	0.096	<0.0001	0.0012
GSE24634 TEFF VS TCONV DAY7 IN CULTURE UP	198	59 (29.8)	59 (29.8)	0 (0)	0.095	<0.0001	0.0012
GOLDRATH EFF VS MEMORY CD8 TCELL UP	200	58 (29)	58 (29)	0 (0)	0.093	<0.0001	0.0012
GSE12845 IGD POS VS NEG BLOOD BCELL DN	186	56 (30.1)	56 (30.1)	0 (0)	0.091	<0.0001	0.0012
GSE22886 NAIVE BCELL VS BM PLASMA CELL DN	195	56 (28.7)	56 (28.7)	0 (0)	0.090	<0.0001	0.0012
GSE39110 DAY3 VS DAY6 POST IMMUNIZATION CD8 TCELL ...	201	55 (27.4)	55 (27.4)	0 (0)	0.087	<0.0001	0.0012
GSE39556 CD8A DC VS NK CELL MOUSE 3H POST POLYIC I...	201	55 (27.4)	55 (27.4)	0 (0)	0.087	<0.0001	0.0012
GSE13547 CTRL VS ANTI IGM STIM BCELL 12H UP	182	53 (29.1)	53 (29.1)	0 (0)	0.087	<0.0001	0.0012
GOLDRATH NAIVE VS EFF CD8 TCELL DN	198	54 (27.3)	54 (27.3)	0 (0)	0.086	<0.0001	0.0012
GSE12845 IGD POS BLOOD VS PRE GC TONSIL BCELL DN	202	54 (26.7)	53 (26.2)	1 (0.5)	0.086	<0.0001	0.0012
GSE14415 INDUCED VS NATURAL TREG DN	178	52 (29.2)	52 (29.2)	0 (0)	0.085	<0.0001	0.0012
GSE25088 WT VS STAT6 KO MACROPHAGE IL4 STIM DN	197	53 (26.9)	53 (26.9)	0 (0)	0.085	<0.0001	0.0012
GSE36476 CTRL VS TSST ACT 72H MEMORY CD4 TCELL OLD...	201	53 (26.4)	53 (26.4)	0 (0)	0.084	<0.0001	0.0012
GSE21063 WT VS NFATC1 KO 8H ANTI IGM STIM BCELL UP	199	52 (26.1)	52 (26.1)	0 (0)	0.083	<0.0001	0.0012
GSE36476 CTRL VS TSST ACT 40H MEMORY CD4 TCELL YOU...	200	52 (26)	52 (26)	0 (0)	0.082	<0.0001	0.0012
GSE36476 CTRL VS TSST ACT 40H MEMORY CD4 TCELL OLD...	200	51 (25.5)	51 (25.5)	0 (0)	0.081	<0.0001	0.0012
GSE14415 TCONV VS FOXP3 KO INDUCED TREG DN	183	48 (26.2)	48 (26.2)	0 (0)	0.078	<0.0001	0.0012

Gene Set Name	Gene Set Genes #	DE Genes N (%)	Up-reg. DE Genes N (%)	Down-reg. DE Genes N (%)	Jaccard Index	P-Value	FDR Adjusted P-Value
GSE2405 S AUREUS VS UNTREATED NEUTROPHIL DN	198	49 (24.7)	49 (24.7)	0 (0)	0.077	<0.0001	0.0012
GSE33292 WT VS TCF1 KO DN3 THYMOCYTE DN	200	49 (24.5)	49 (24.5)	0 (0)	0.077	<0.0001	0.0012
GSE14415 NATURAL TREG VS TCONV DN	181	47 (26)	47 (26)	0 (0)	0.076	<0.0001	0.0012
GSE25088 WT VS STAT6 KO MACROPHAGE DN	196	48 (24.5)	48 (24.5)	0 (0)	0.076	<0.0001	0.0012
GSE29617 CTRL VS DAY7 TIV FLU VACCINE PBMC 2008 DN	187	47 (25.1)	47 (25.1)	0 (0)	0.075	<0.0001	0.0012
GSE20727 CTRL VS ROS INHIBITOR TREATED DC DN	191	47 (24.6)	47 (24.6)	0 (0)	0.075	<0.0001	0.0012
GSE45365 HEALTHY VS MCMV INFECTION CD11B DC DN	190	46 (24.2)	46 (24.2)	0 (0)	0.073	<0.0001	0.0012
GSE24634 IL4 VS CTRL TREATED NAIVE CD4 TCELL DAY7 ...	195	46 (23.6)	46 (23.6)	0 (0)	0.073	<0.0001	0.0012
GSE29614 CTRL VS TIV FLU VACCINE PBMC 2007 DN	172	44 (25.6)	44 (25.6)	0 (0)	0.072	<0.0001	0.0012
GSE24634 TREG VS TCONV POST DAY7 IL4 CONVERSION UP	196	45 (23)	45 (23)	0 (0)	0.071	<0.0001	0.0012
GSE40274 CTRL VS FOXP3 AND HELIOS TRANSDUCED ACTIV...	198	45 (22.7)	45 (22.7)	0 (0)	0.071	<0.0001	0.0012
GSE40273 EOS KO VS WT TREG UP	199	44 (22.1)	44 (22.1)	0 (0)	0.069	<0.0001	0.0012
GSE45365 WT VS IFNAR KO BCELL MCMV INFECTION DN	188	43 (22.9)	43 (22.9)	0 (0)	0.069	<0.0001	0.0012
GSE13547 2H VS 12 H ANTI IGM STIM BCELL UP	173	42 (24.3)	42 (24.3)	0 (0)	0.068	<0.0001	0.0012
GSE20727 CTRL VS ROS INH AND DNFB ALLERGEN TREATED...	194	43 (22.2)	43 (22.2)	0 (0)	0.068	<0.0001	0.0012
GSE23568 CTRL VS ID3 TRANSDUCED CD8 TCELL DN	199	43 (21.6)	43 (21.6)	0 (0)	0.067	<0.0001	0.0012
GSE13547 CTRL VS ANTI IGM STIM BCELL 2H UP	180	41 (22.8)	41 (22.8)	0 (0)	0.066	<0.0001	0.0012
GSE22886 IGG IGA MEMORY BCELL VS BLOOD PLASMA CELL...	197	42 (21.3)	42 (21.3)	0 (0)	0.066	<0.0001	0.0012
KAECH DAY8 EFF VS MEMORY CD8 TCELL UP	202	42 (20.8)	42 (20.8)	0 (0)	0.065	<0.0001	0.0012
GSE45365 WT VS IFNAR KO BCELL DN	181	40 (22.1)	40 (22.1)	0 (0)	0.064	<0.0001	0.0012
GSE10239 NAIVE VS DAY4.5 EFF CD8 TCELL DN	199	40 (20.1)	40 (20.1)	0 (0)	0.062	<0.0001	0.0012
GSE2405 HEAT KILLED LYSATE VS LIVE A PHAGOCYTOPHIL...	199	40 (20.1)	40 (20.1)	0 (0)	0.062	<0.0001	0.0012
GSE40274 CTRL VS FOXP3 TRANSDUCED ACTIVATED CD4 TC...	201	40 (19.9)	40 (19.9)	0 (0)	0.062	<0.0001	0.0012
GSE5679 CTRL VS PPARG LIGAND ROSIGLITAZONE TREATED...	201	40 (19.9)	40 (19.9)	0 (0)	0.062	<0.0001	0.0012
GSE40274 CTRL VS EOS TRANSDUCED ACTIVATED CD4 TCEL...	172	38 (22.1)	38 (22.1)	0 (0)	0.062	<0.0001	0.0012

Table 70: Enriched MSigDB Immunological Signatures (B Cells, Trivalent Influenza Vaccine, Day 6). Results are sorted by FDR adjusted p-value and Jaccard similarity index. Top 50 results are listed.

Gene Set Name	Gene Set Genes #	DE Genes N (%)	Up-reg. DE Genes N (%)	Down-reg. DE Genes N (%)	Jaccard Index	P-Value	FDR Adjusted P-Value
KEGG CELL CYCLE	124	23 (18.5)	23 (18.5)	0 (0)	0.039	<0.0001	0.0046
KEGG N GLYCAN BIOSYNTHESIS	46	13 (28.3)	13 (28.3)	0 (0)	0.025	<0.0001	0.0046
KEGG PROTEIN EXPORT	23	11 (47.8)	11 (47.8)	0 (0)	0.022	<0.0001	0.0046
KEGG P53 SIGNALING PATHWAY	68	11 (16.2)	10 (14.7)	1 (1.5)	0.020	<0.0001	0.0046
KEGG OOCYTE MEIOSIS	112	12 (10.7)	12 (10.7)	0 (0)	0.021	0.0005	0.0186
KEGG VIBRIO CHOLERAE INFECTION	53	6 (11.3)	6 (11.3)	0 (0)	0.011	0.003	0.093

Table 71: Enriched MSigDB KEGG Pathways (B Cells, Trivalent Influenza Vaccine, Day 6). Results are sorted by FDR adjusted p-value and Jaccard similarity index.

Gene Set Name	Gene Set Genes #	DE Genes N (%)	Up-reg. DE Genes N (%)	Down-reg. DE Genes N (%)	Jaccard Index	P-Value	FDR Adjusted P-Value
GO TRANSFERASE ACTIVITY TRANSFERRING HEXOSYL GROUP...	201	21 (10.4)	21 (10.4)	0 (0)	0.032	<0.0001	0.0186

Gene Set Name	Gene Set Genes #	DE Genes N (%)	Up-reg. DE Genes N (%)	Down-reg. DE Genes N (%)	Jaccard Index	P-Value	FDR Adjusted P-Value
GO TRANSFERASE ACTIVITY TRANSFERRING GLYCOSYL GROU...	279	21 (7.5)	21 (7.5)	0 (0)	0.028	<0.0001	0.0186
GO TUBULIN BINDING	272	19 (7)	19 (7)	0 (0)	0.026	<0.0001	0.0186
GO MICROTUBULE BINDING	201	15 (7.5)	15 (7.5)	0 (0)	0.022	0.0002	0.0186
GO ISOMERASE ACTIVITY	161	12 (7.5)	12 (7.5)	0 (0)	0.019	0.0002	0.0186
GO MICROTUBULE MOTOR ACTIVITY	76	10 (13.2)	10 (13.2)	0 (0)	0.018	<0.0001	0.0186
GO UNFOLDED PROTEIN BINDING	94	10 (10.6)	10 (10.6)	0 (0)	0.018	<0.0001	0.0186
GO INTRAMOLECULAR OXIDOREDUCTASE ACTIVITY TRANSPOS...	22	7 (31.8)	7 (31.8)	0 (0)	0.014	0.0002	0.0186
GO INTRAMOLECULAR OXIDOREDUCTASE ACTIVITY	53	7 (13.2)	7 (13.2)	0 (0)	0.013	0.0002	0.0186
GO HISTONE KINASE ACTIVITY	19	5 (26.3)	5 (26.3)	0 (0)	0.010	0.0002	0.0186
GO MISFOLDED PROTEIN BINDING	12	4 (33.3)	4 (33.3)	0 (0)	0.008	0.0005	0.0422
GO KINASE BINDING	608	29 (4.8)	29 (4.8)	0 (0)	0.027	0.0007	0.0464
GO MOTOR ACTIVITY	129	11 (8.5)	11 (8.5)	0 (0)	0.018	0.0007	0.0464
GO MANNOSIDASE ACTIVITY	15	4 (26.7)	4 (26.7)	0 (0)	0.008	0.0007	0.0464
GO CARBOHYDRATE BINDING	275	15 (5.5)	15 (5.5)	0 (0)	0.020	0.0009	0.0523
GO UDP GLYCOSYLTRANSFERASE ACTIVITY	138	11 (8)	11 (8)	0 (0)	0.018	0.0009	0.0523
GO ATP DEPENDENT MICROTUBULE MOTOR ACTIVITY	18	4 (22.2)	4 (22.2)	0 (0)	0.008	0.0016	0.0874

Table 72: Enriched MSigDB Molecular Functions (B Cells, Trivalent Influenza Vaccine, Day 6). Results are sorted by FDR adjusted p-value and Jaccard similarity index.

Gene Set Name	Gene Set Genes #	DE Genes N (%)	Up-reg. DE Genes N (%)	Down-reg. DE Genes N (%)	Jaccard Index	P-Value	FDR Adjusted P-Value
REACTOME CELL CYCLE MITOTIC	308	48 (15.6)	48 (15.6)	0 (0)	0.065	<0.0001	0.0023
REACTOME CELL CYCLE	400	51 (12.8)	51 (12.8)	0 (0)	0.061	<0.0001	0.0023
REACTOME DNA REPLICATION	188	32 (17)	32 (17)	0 (0)	0.050	<0.0001	0.0023
REACTOME METABOLISM OF PROTEINS	424	43 (10.1)	43 (10.1)	0 (0)	0.050	<0.0001	0.0023
REACTOME MITOTIC M M G1 PHASES	168	28 (16.7)	28 (16.7)	0 (0)	0.045	<0.0001	0.0023
REACTOME ASPARAGINE N LINKED GLYCOSYLATION	81	24 (29.6)	24 (29.6)	0 (0)	0.044	<0.0001	0.0023
REACTOME POST TRANSLATIONAL PROTEIN MODIFICATION	181	28 (15.5)	28 (15.5)	0 (0)	0.044	<0.0001	0.0023
REACTOME DIABETES PATHWAYS	129	23 (17.8)	22 (17.1)	1 (0.8)	0.039	<0.0001	0.0023
REACTOME UNFOLDED PROTEIN RESPONSE	78	18 (23.1)	18 (23.1)	0 (0)	0.033	<0.0001	0.0023
REACTOME SRP DEPENDENT COTRANSLATIONAL PROTEIN TAR...	108	18 (16.7)	18 (16.7)	0 (0)	0.031	<0.0001	0.0023
REACTOME MITOTIC G1 G1 S PHASES	130	18 (13.8)	18 (13.8)	0 (0)	0.030	<0.0001	0.0023
REACTOME TRANSLATION	145	18 (12.4)	18 (12.4)	0 (0)	0.029	<0.0001	0.0023
REACTOME ACTIVATION OF CHAPERONE GENES BY XBP1S	46	15 (32.6)	15 (32.6)	0 (0)	0.029	<0.0001	0.0023
REACTOME MITOTIC PROMETAPHASE	86	16 (18.6)	16 (18.6)	0 (0)	0.029	<0.0001	0.0023
REACTOME CELL CYCLE CHECKPOINTS	112	16 (14.3)	16 (14.3)	0 (0)	0.028	<0.0001	0.0023
REACTOME G1 S TRANSITION	106	15 (14.2)	15 (14.2)	0 (0)	0.026	<0.0001	0.0023
REACTOME REGULATION OF MITOTIC CELL CYCLE	77	12 (15.6)	12 (15.6)	0 (0)	0.022	<0.0001	0.0023
REACTOME G2 M CHECKPOINTS	42	11 (26.2)	11 (26.2)	0 (0)	0.021	<0.0001	0.0023
REACTOME E2F MEDIATED REGULATION OF DNA REPLICATIO...	33	10 (30.3)	10 (30.3)	0 (0)	0.020	<0.0001	0.0023
REACTOME G1 S SPECIFIC TRANSCRIPTION	17	9 (52.9)	9 (52.9)	0 (0)	0.018	<0.0001	0.0023
REACTOME M G1 TRANSITION	78	10 (12.8)	10 (12.8)	0 (0)	0.018	<0.0001	0.0023
REACTOME TRANSPORT TO THE GOLGI AND SUBSEQUENT MOD...	33	9 (27.3)	9 (27.3)	0 (0)	0.018	<0.0001	0.0023
REACTOME ACTIVATION OF ATR IN RESPONSE TO REPLICAT...	36	8 (22.2)	8 (22.2)	0 (0)	0.016	<0.0001	0.0023
REACTOME KINESINS	24	7 (29.2)	7 (29.2)	0 (0)	0.014	<0.0001	0.0023

Gene Set Name	Gene Set Genes #	DE Genes N (%)	Up-reg. DE Genes N (%)	Down-reg. DE Genes N (%)	Jaccard Index	P-Value	FDR Adjusted P-Value
REACTOME CYCLIN A B1 ASSOCIATED EVENTS DURING G2 M...	15	6 (40)	6 (40)	0 (0)	0.012	<0.0001	0.0023
REACTOME N GLYCAN TRIMMING IN THE ER AND CALNEXIN ...	13	6 (46.2)	6 (46.2)	0 (0)	0.012	<0.0001	0.0023
REACTOME ANTIGEN PRESENTATION FOLDING ASSEMBLY AND...	20	6 (30)	6 (30)	0 (0)	0.012	<0.0001	0.0023
REACTOME ASSOCIATION OF LICENSING FACTORS WITH THE...	13	5 (38.5)	5 (38.5)	0 (0)	0.010	<0.0001	0.0023
REACTOME CALNEXIN CALRETICULIN CYCLE	11	5 (45.5)	5 (45.5)	0 (0)	0.010	<0.0001	0.0023
REACTOME MITOTIC G2 G2 M PHASES	78	11 (14.1)	11 (14.1)	0 (0)	0.020	0.0002	0.0042
REACTOME G0 AND EARLY G1	23	6 (26.1)	6 (26.1)	0 (0)	0.012	0.0002	0.0042
REACTOME UNWINDING OF DNA	11	5 (45.5)	5 (45.5)	0 (0)	0.010	0.0002	0.0042
REACTOME ACTIVATION OF THE PRE REPLICATIVE COMPLEX	30	6 (20)	6 (20)	0 (0)	0.012	0.0003	0.0058
REACTOME APC CDC20 MEDIATED DEGRADATION OF NEK2A	21	5 (23.8)	5 (23.8)	0 (0)	0.010	0.0003	0.0058
REACTOME ACTIVATION OF CHAPERONE GENES BY ATF6 ALP...	9	4 (44.4)	4 (44.4)	0 (0)	0.008	0.0003	0.0058
REACTOME DNA STRAND ELONGATION	30	6 (20)	6 (20)	0 (0)	0.012	0.0004	0.0073
REACTOME ACTIVATION OF CHAPERONES BY ATF6 ALPHA	11	4 (36.4)	4 (36.4)	0 (0)	0.008	0.0004	0.0073
REACTOME S PHASE	106	11 (10.4)	11 (10.4)	0 (0)	0.019	0.0005	0.0089
REACTOME SYNTHESIS OF DNA	90	10 (11.1)	10 (11.1)	0 (0)	0.018	0.0006	0.0096
REACTOME APC C CDC20 MEDIATED DEGRADATION OF MITOT...	65	8 (12.3)	8 (12.3)	0 (0)	0.015	0.0006	0.0096
REACTOME CDC6 ASSOCIATION WITH THE ORC ORIGIN COMP...	11	4 (36.4)	4 (36.4)	0 (0)	0.008	0.0006	0.0096
REACTOME E2F ENABLED INHIBITION OF PRE REPLICATION...	10	4 (40)	4 (40)	0 (0)	0.008	0.0006	0.0096
REACTOME ASSEMBLY OF THE PRE REPLICATIVE COMPLEX	63	8 (12.7)	8 (12.7)	0 (0)	0.015	0.0007	0.0107
REACTOME SYNTHESIS SECRETION AND INACTIVATION OF G...	13	4 (30.8)	4 (30.8)	0 (0)	0.008	0.0007	0.0107
REACTOME SYNTHESIS SECRETION AND DEACYLATION OF GH...	15	4 (26.7)	4 (26.7)	0 (0)	0.008	0.0012	0.018
REACTOME METABOLISM OF NUCLEOTIDES	72	8 (11.1)	8 (11.1)	0 (0)	0.015	0.0014	0.0205
REACTOME PHOSPHORYLATION OF THE APC C	17	4 (23.5)	4 (23.5)	0 (0)	0.008	0.0018	0.0258
REACTOME APC C CDH1 MEDIATED DEGRADATION OF CDC20 ...	64	7 (10.9)	7 (10.9)	0 (0)	0.013	0.0019	0.0261
REACTOME AMINO ACID SYNTHESIS AND INTERCONVERSION ...	16	4 (25)	4 (25)	0 (0)	0.008	0.0019	0.0261
REACTOME SYNTHESIS SECRETION AND INACTIVATION OF G...	18	4 (22.2)	4 (22.2)	0 (0)	0.008	0.002	0.027

Table 73: Enriched MSigDB Reactome Pathways (B Cells, Trivalent Influenza Vaccine, Day 6). Results are sorted by FDR adjusted p-value and Jaccard similarity index. Top 50 results are listed.

Gene Set Name	Gene Set Genes #	DE Genes N (%)	Up-reg. DE Genes N (%)	Down-reg. DE Genes N (%)	Jaccard Index	P-Value	FDR Adjusted P-Value
cell cycle (I) (M4.1)	139	33 (23.7)	33 (23.7)	0 (0)	0.081	<0.0001	0.0031
cell cycle and transcription (M4.0)	318	46 (14.5)	44 (13.8)	2 (0.6)	0.080	<0.0001	0.0031
PLK1 signaling events (M4.2)	32	12 (37.5)	12 (37.5)	0 (0)	0.037	<0.0001	0.0031
mitotic cell cycle in stimulated CD4 T cells (M4.5...	33	11 (33.3)	11 (33.3)	0 (0)	0.034	<0.0001	0.0031
cell division (stimulated CD4+ T cells) (M46)	27	10 (37)	10 (37)	0 (0)	0.031	<0.0001	0.0031
cell cycle (III) (M103)	51	9 (17.6)	9 (17.6)	0 (0)	0.026	<0.0001	0.0031
mitotic cell division (M6)	27	8 (29.6)	8 (29.6)	0 (0)	0.025	<0.0001	0.0031
cell cycle (II) (M4.10)	14	7 (50)	7 (50)	0 (0)	0.023	<0.0001	0.0031
E2F transcription factor network (M8)	14	7 (50)	7 (50)	0 (0)	0.023	<0.0001	0.0031
Plasma cell surface signature (S3)	23	7 (30.4)	7 (30.4)	0 (0)	0.022	<0.0001	0.0031
plasma cells, immunoglobulins (M156.1)	22	6 (27.3)	6 (27.3)	0 (0)	0.019	<0.0001	0.0031
mitotic cell cycle (M4.7)	20	5 (25)	5 (25)	0 (0)	0.016	0.0002	0.0058
mitotic cell cycle in stimulated CD4 T cells (M4.1...	11	4 (36.4)	4 (36.4)	0 (0)	0.013	0.0003	0.008

Gene Set Name	Gene Set Genes #	DE Genes N (%)	Up-reg. DE Genes N (%)	Down-reg. DE Genes N (%)	Jaccard Index	P-Value	FDR Adjusted P-Value
Ran mediated mitosis (M15)	13	4 (30.8)	4 (30.8)	0 (0)	0.013	0.0009	0.0222
cell division in stimulated CD4 T cells (M4.6)	19	4 (21.1)	4 (21.1)	0 (0)	0.013	0.0023	0.053
plasma cells and B cells, immunoglobulins (M156.0)	30	4 (13.3)	4 (13.3)	0 (0)	0.012	0.0043	0.0875
C-MYC transcriptional network (M4.12)	12	3 (25)	3 (25)	0 (0)	0.010	0.0043	0.0875

Table 74: Enriched Blood Transcription Modules (B Cells, Trivalent Influenza Vaccine, Day 7). Results are sorted by FDR adjusted p-value and Jaccard similarity index.

Gene Set Name	Gene Set Genes #	DE Genes N (%)	Up-reg. DE Genes N (%)	Down-reg. DE Genes N (%)	Jaccard Index	P-Value	FDR Adjusted P-Value
GO RESPONSE TO ENDOPLASMIC RETICULUM STRESS	234	29 (12.4)	29 (12.4)	0 (0)	0.057	<0.0001	0.0097
GO CELLULAR RESPONSE TO TOPOLOGICALLY INCORRECT PR...	123	20 (16.3)	20 (16.3)	0 (0)	0.049	<0.0001	0.0097
GO RESPONSE TO TOPOLOGICALLY INCORRECT PROTEIN	164	21 (12.8)	21 (12.8)	0 (0)	0.047	<0.0001	0.0097
GO IRE1 MEDIATED UNFOLDED PROTEIN RESPONSE	57	15 (26.3)	15 (26.3)	0 (0)	0.043	<0.0001	0.0097
GO PROTEIN FOLDING	217	19 (8.8)	19 (8.8)	0 (0)	0.038	<0.0001	0.0097
GO MITOTIC NUCLEAR DIVISION	362	24 (6.6)	24 (6.6)	0 (0)	0.037	<0.0001	0.0097
GO ORGANELLE FISSION	496	27 (5.4)	27 (5.4)	0 (0)	0.035	<0.0001	0.0097
GO MITOTIC CELL CYCLE	767	36 (4.7)	36 (4.7)	0 (0)	0.035	<0.0001	0.0097
GO CELL CYCLE PHASE TRANSITION	254	18 (7.1)	18 (7.1)	0 (0)	0.033	<0.0001	0.0097
GO PROTEASOMAL PROTEIN CATABOLIC PROCESS	272	18 (6.6)	18 (6.6)	0 (0)	0.032	<0.0001	0.0097
GO CELL CYCLE PROCESS	1082	43 (4)	43 (4)	0 (0)	0.032	<0.0001	0.0097
GO CELLULAR RESPONSE TO STRESS	1563	56 (3.6)	56 (3.6)	0 (0)	0.031	<0.0001	0.0097
GO CHROMOSOME SEGREGATION	272	17 (6.2)	17 (6.2)	0 (0)	0.030	<0.0001	0.0097
GO CELL DIVISION	461	22 (4.8)	22 (4.8)	0 (0)	0.030	<0.0001	0.0097
GO NUCLEAR CHROMOSOME SEGREGATION	228	15 (6.6)	15 (6.6)	0 (0)	0.029	<0.0001	0.0097
GO REGULATION OF NUCLEAR DIVISION	162	13 (8)	13 (8)	0 (0)	0.029	<0.0001	0.0097
GO CELL CYCLE	1316	45 (3.4)	45 (3.4)	0 (0)	0.029	<0.0001	0.0097
GO SISTER CHROMATID SEGREGATION	177	13 (7.3)	13 (7.3)	0 (0)	0.028	<0.0001	0.0097
GO CELL REDOX HOMEOSTASIS	67	10 (14.9)	10 (14.9)	0 (0)	0.028	<0.0001	0.0097
GO CARBOHYDRATE DERIVATIVE BIOSYNTHETIC PROCESS	592	24 (4.1)	24 (4.1)	0 (0)	0.028	<0.0001	0.0097
GO ERAD PATHWAY	74	10 (13.5)	10 (13.5)	0 (0)	0.027	<0.0001	0.0097
GO PROTEIN N LINKED GLYCOSYLATION	75	10 (13.3)	10 (13.3)	0 (0)	0.027	<0.0001	0.0097
GO REGULATION OF CELL DIVISION	271	15 (5.5)	15 (5.5)	0 (0)	0.027	<0.0001	0.0097
GO CELL CYCLE CHECKPOINT	196	13 (6.6)	13 (6.6)	0 (0)	0.027	<0.0001	0.0097
GO REGULATION OF CELL CYCLE	948	32 (3.4)	32 (3.4)	0 (0)	0.026	<0.0001	0.0097
GO CELLULAR HOMEOSTASIS	678	25 (3.7)	24 (3.5)	1 (0.1)	0.026	<0.0001	0.0097
GO POSITIVE REGULATION OF CELL CYCLE PROCESS	247	14 (5.7)	14 (5.7)	0 (0)	0.026	<0.0001	0.0097
GO MITOTIC SISTER CHROMATID SEGREGATION	92	10 (10.9)	10 (10.9)	0 (0)	0.026	<0.0001	0.0097
GO PROTEIN CATABOLIC PROCESS	582	22 (3.8)	22 (3.8)	0 (0)	0.025	<0.0001	0.0097
GO GLYCOSYLATION	263	14 (5.3)	14 (5.3)	0 (0)	0.025	<0.0001	0.0097
GO ER ASSOCIATED UBIQUITIN DEPENDENT PROTEIN CATAB...	61	9 (14.8)	9 (14.8)	0 (0)	0.025	<0.0001	0.0097
GO REGULATION OF CELL CYCLE PROCESS	558	21 (3.8)	21 (3.8)	0 (0)	0.025	<0.0001	0.0097
GO POSITIVE REGULATION OF CELL CYCLE	331	15 (4.5)	15 (4.5)	0 (0)	0.024	<0.0001	0.0097
GO PEPTIDYL ASPARAGINE MODIFICATION	39	8 (20.5)	8 (20.5)	0 (0)	0.024	<0.0001	0.0097
GO REGULATION OF CHROMOSOME SEGREGATION	84	9 (10.7)	9 (10.7)	0 (0)	0.024	<0.0001	0.0097
GO REGULATION OF MICROTUBULE POLYMERIZATION OR DEP...	179	11 (6.1)	11 (6.1)	0 (0)	0.023	<0.0001	0.0097

Gene Set Name	Gene Set Genes #	DE Genes N (%)	Up-reg. DE Genes N (%)	Down-reg. DE Genes N (%)	Jaccard Index	P-Value	FDR Adjusted P-Value
GO ALPHA AMINO ACID METABOLIC PROCESS	228	12 (5.3)	12 (5.3)	0 (0)	0.023	<0.0001	0.0097
GO CELL CYCLE G1 S PHASE TRANSITION	110	9 (8.2)	9 (8.2)	0 (0)	0.022	<0.0001	0.0097
GO G1 S TRANSITION OF MITOTIC CELL CYCLE	110	9 (8.2)	9 (8.2)	0 (0)	0.022	<0.0001	0.0097
GO SISTER CHROMATID COHESION	111	9 (8.1)	9 (8.1)	0 (0)	0.022	<0.0001	0.0097
GO REGULATION OF SISTER CHROMATID SEGREGATION	66	8 (12.1)	8 (12.1)	0 (0)	0.022	<0.0001	0.0097
GO PROTEIN EXIT FROM ENDOPLASMIC RETICULUM	20	7 (35)	7 (35)	0 (0)	0.022	<0.0001	0.0097
GO MITOTIC SPINDLE ORGANIZATION	69	8 (11.6)	8 (11.6)	0 (0)	0.022	<0.0001	0.0097
GO REGULATION OF TRANSCRIPTION INVOLVED IN G1 S TR...	26	6 (23.1)	6 (23.1)	0 (0)	0.019	<0.0001	0.0097
GO ER NUCLEUS SIGNALING PATHWAY	34	6 (17.6)	6 (17.6)	0 (0)	0.018	<0.0001	0.0097
GO NEGATIVE REGULATION OF RESPONSE TO ENDOPLASMIC ...	39	6 (15.4)	6 (15.4)	0 (0)	0.018	<0.0001	0.0097
GO RETROGRADE PROTEIN TRANSPORT ER TO CYTOSOL	16	5 (31.2)	5 (31.2)	0 (0)	0.016	<0.0001	0.0097
GO ENDOPLASMIC RETICULUM TO CYTOSOL TRANSPORT	23	5 (21.7)	5 (21.7)	0 (0)	0.016	<0.0001	0.0097
GO REGULATION OF MITOTIC CELL CYCLE	468	18 (3.8)	18 (3.8)	0 (0)	0.024	0.0002	0.0172
GO POSITIVE REGULATION OF MITOTIC CELL CYCLE	123	9 (7.3)	9 (7.3)	0 (0)	0.022	0.0002	0.0172

Table 75: Enriched MSigDB Biological Processes (B Cells, Trivalent Influenza Vaccine, Day 7). Results are sorted by FDR adjusted p-value and Jaccard similarity index. Top 50 results are listed.

Gene Set Name	Gene Set Genes #	DE Genes N (%)	Up-reg. DE Genes N (%)	Down-reg. DE Genes N (%)	Jaccard Index	P-Value	FDR Adjusted P-Value
GO ENDOPLASMIC RETICULUM PART	1162	67 (5.8)	67 (5.8)	0 (0)	0.048	<0.0001	0.0034
GO NUCLEAR OUTER MEMBRANE ENDOPLASMIC RETICULUM ME...	1005	55 (5.5)	55 (5.5)	0 (0)	0.044	<0.0001	0.0034
GO ENDOPLASMIC RETICULUM LUMEN	202	21 (10.4)	21 (10.4)	0 (0)	0.043	<0.0001	0.0034
GO ENDOPLASMIC RETICULUM	1631	75 (4.6)	75 (4.6)	0 (0)	0.040	<0.0001	0.0034
GO ENDOPLASMIC RETICULUM CHAPERONE COMPLEX	11	9 (81.8)	9 (81.8)	0 (0)	0.029	<0.0001	0.0034
GO INTRINSIC COMPONENT OF ENDOPLASMIC RETICULUM ME...	135	12 (8.9)	12 (8.9)	0 (0)	0.028	<0.0001	0.0034
GO ROUGH ENDOPLASMIC RETICULUM	71	10 (14.1)	10 (14.1)	0 (0)	0.028	<0.0001	0.0034
GO CONDENSED CHROMOSOME	196	13 (6.6)	13 (6.6)	0 (0)	0.027	<0.0001	0.0034
GO CHROMOSOME CENTROMERIC REGION	174	12 (6.9)	12 (6.9)	0 (0)	0.026	<0.0001	0.0034
GO CONDENSED CHROMOSOME CENTROMERIC REGION	102	10 (9.8)	10 (9.8)	0 (0)	0.025	<0.0001	0.0034
GO PIGMENT GRANULE	102	10 (9.8)	10 (9.8)	0 (0)	0.025	<0.0001	0.0034
GO ENDOPLASMIC RETICULUM GOLGI INTERMEDIATE COMPAR...	106	10 (9.4)	9 (8.5)	1 (0.9)	0.025	<0.0001	0.0034
GO SMOOTH ENDOPLASMIC RETICULUM	33	7 (21.2)	7 (21.2)	0 (0)	0.021	<0.0001	0.0034
GO CONDENSED NUCLEAR CHROMOSOME CENTROMERIC REGION	18	5 (27.8)	5 (27.8)	0 (0)	0.016	<0.0001	0.0034
GO ROUGH ENDOPLASMIC RETICULUM MEMBRANE	21	5 (23.8)	5 (23.8)	0 (0)	0.016	<0.0001	0.0034
GO CONDENSED CHROMOSOME OUTER KINETOCHORE	12	4 (33.3)	4 (33.3)	0 (0)	0.013	<0.0001	0.0034
GO ENDOCYTIC VESICLE LUMEN	17	4 (23.5)	4 (23.5)	0 (0)	0.013	<0.0001	0.0034
GO KINETOCHORE	120	9 (7.5)	9 (7.5)	0 (0)	0.022	0.0003	0.0097
GO CHROMOSOMAL REGION	330	14 (4.2)	14 (4.2)	0 (0)	0.023	0.0004	0.0123
GO VESICLE LUMEN	106	7 (6.6)	6 (5.7)	1 (0.9)	0.017	0.0005	0.0146
GO DERLIN 1 RETROTRANSLOCATION COMPLEX	11	3 (27.3)	3 (27.3)	0 (0)	0.010	0.001	0.0278
GO SPINDLE POLE	126	8 (6.3)	8 (6.3)	0 (0)	0.019	0.0014	0.0341
GO MIDBODY	132	8 (6.1)	8 (6.1)	0 (0)	0.019	0.0014	0.0341
GO SPINDLE MIDZONE	27	4 (14.8)	4 (14.8)	0 (0)	0.012	0.0014	0.0341
GO GOLGI APPARATUS PART	890	27 (3)	26 (2.9)	1 (0.1)	0.023	0.0019	0.0411

Gene Set Name	Gene Set Genes #	DE Genes N (%)	Up-reg. DE Genes N (%)	Down-reg. DE Genes N (%)	Jaccard Index	P-Value	FDR Adjusted P-Value
GO INTRINSIC COMPONENT OF ORGANELLE MEMBRANE	281	12 (4.3)	12 (4.3)	0 (0)	0.021	0.0018	0.0411
GO CYTOPLASMIC MICROTUBULE	57	5 (8.8)	5 (8.8)	0 (0)	0.014	0.0019	0.0411
GO CENTROSOME	487	17 (3.5)	17 (3.5)	0 (0)	0.022	0.0022	0.0459
GO GOLGI APPARATUS	1445	37 (2.6)	36 (2.5)	1 (0.1)	0.022	0.0029	0.0584
GO GOLGI MEMBRANE	703	22 (3.1)	22 (3.1)	0 (0)	0.022	0.0035	0.0681

Table 76: Enriched MSigDB Cellular Components (B Cells, Trivalent Influenza Vaccine, Day 7). Results are sorted by FDR adjusted p-value and Jaccard similarity index.

Gene Set Name	Gene Set Genes #	DE Genes N (%)	Up-reg. DE Genes N (%)	Down-reg. DE Genes N (%)	Jaccard Index	P-Value	FDR Adjusted P-Value
GSE29614 CTRL VS DAY7 TIV FLU VACCINE PBMC DN	183	74 (40.4)	74 (40.4)	0 (0)	0.180	<0.0001	0.0018
GSE29614 DAY3 VS DAY7 TIV FLU VACCINE PBMC DN	179	71 (39.7)	71 (39.7)	0 (0)	0.173	<0.0001	0.0018
GSE29164 CD8 TCELL VS CD8 TCELL AND IL12 TREATED M...	188	64 (34)	64 (34)	0 (0)	0.150	<0.0001	0.0018
GSE13411 PLASMA CELL VS MEMORY BCELL UP	190	58 (30.5)	58 (30.5)	0 (0)	0.133	<0.0001	0.0018
GSE22886 NAIVE BCELL VS BM PLASMA CELL DN	195	48 (24.6)	47 (24.1)	1 (0.5)	0.107	<0.0001	0.0018
GSE29617 CTRL VS DAY7 TIV FLU VACCINE PBMC 2008 DN	187	42 (22.5)	42 (22.5)	0 (0)	0.094	<0.0001	0.0018
GSE12845 IGD POS VS NEG BLOOD BCELL DN	186	39 (21)	39 (21)	0 (0)	0.087	<0.0001	0.0018
GSE20727 CTRL VS ROS INHIBITOR TREATED DC DN	191	39 (20.4)	39 (20.4)	0 (0)	0.086	<0.0001	0.0018
GSE22886 NAIVE BCELL VS BLOOD PLASMA CELL DN	194	38 (19.6)	38 (19.6)	0 (0)	0.083	<0.0001	0.0018
GSE36476 CTRL VS TSST ACT 72H MEMORY CD4 TCELL YOU...	201	34 (16.9)	34 (16.9)	0 (0)	0.072	<0.0001	0.0018
GSE29614 CTRL VS TIV FLU VACCINE PBMC 2007 DN	172	32 (18.6)	32 (18.6)	0 (0)	0.072	<0.0001	0.0018
GSE13411 IGM MEMORY BCELL VS PLASMA CELL DN	190	33 (17.4)	33 (17.4)	0 (0)	0.072	<0.0001	0.0018
GSE22886 IGG IGA MEMORY BCELL VS BM PLASMA CELL DN	190	32 (16.8)	31 (16.3)	1 (0.5)	0.069	<0.0001	0.0018
GSE24634 TEFF VS TCONV DAY7 IN CULTURE UP	198	32 (16.2)	32 (16.2)	0 (0)	0.068	<0.0001	0.0018
GSE13411 NAIVE BCELL VS PLASMA CELL DN	188	31 (16.5)	31 (16.5)	0 (0)	0.067	<0.0001	0.0018
GSE22886 IGM MEMORY BCELL VS BM PLASMA CELL DN	194	31 (16)	31 (16)	0 (0)	0.067	<0.0001	0.0018
GSE15750 DAY6 VS DAY10 EFF CD8 TCELL UP	201	31 (15.4)	31 (15.4)	0 (0)	0.066	<0.0001	0.0018
GSE15750 DAY6 VS DAY10 TRAF6KO EFF CD8 TCELL UP	201	31 (15.4)	31 (15.4)	0 (0)	0.066	<0.0001	0.0018
GSE12845 IGD POS BLOOD VS PRE GC TONSIL BCELL DN	202	31 (15.3)	31 (15.3)	0 (0)	0.065	<0.0001	0.0018
GSE45365 HEALTHY VS MCMV INFECTION CD11B DC DN	190	30 (15.8)	30 (15.8)	0 (0)	0.065	<0.0001	0.0018
GSE22886 IGG IGA MEMORY BCELL VS BLOOD PLASMA CELL...	197	29 (14.7)	29 (14.7)	0 (0)	0.062	<0.0001	0.0018
GSE40273 EOS KO VS WT TREG UP	199	29 (14.6)	29 (14.6)	0 (0)	0.061	<0.0001	0.0018
GSE10325 BCELL VS LUPUS BCELL DN	200	29 (14.5)	29 (14.5)	0 (0)	0.061	<0.0001	0.0018
GSE36476 CTRL VS TSST ACT 40H MEMORY CD4 TCELL YOU...	200	29 (14.5)	29 (14.5)	0 (0)	0.061	<0.0001	0.0018
GSE39556 CD8A DC VS NK CELL MOUSE 3H POST POLYIC I...	201	29 (14.4)	29 (14.4)	0 (0)	0.061	<0.0001	0.0018
GSE25088 WT VS STAT6 KO MACROPHAGE IL4 STIM DN	197	28 (14.2)	28 (14.2)	0 (0)	0.059	<0.0001	0.0018
GSE36476 CTRL VS TSST ACT 72H MEMORY CD4 TCELL OLD...	201	28 (13.9)	28 (13.9)	0 (0)	0.059	<0.0001	0.0018
GSE12366 PLASMA CELL VS MEMORY BCELL UP	185	27 (14.6)	26 (14.1)	1 (0.5)	0.059	<0.0001	0.0018
GSE40666 UNTREATED VS IFNA STIM STAT4 KO EFFECTOR ...	196	27 (13.8)	27 (13.8)	0 (0)	0.057	<0.0001	0.0018
GOLDRATH EFF VS MEMORY CD8 TCELL UP	200	27 (13.5)	26 (13)	1 (0.5)	0.057	<0.0001	0.0018
GSE3982 CENT MEMORY CD4 TCELL VS TH1 DN	200	27 (13.5)	26 (13)	1 (0.5)	0.057	<0.0001	0.0018
GSE40274 CTRL VS FOXP3 AND HELIOS TRANSDUCED ACTIV...	198	26 (13.1)	26 (13.1)	0 (0)	0.055	<0.0001	0.0018
GSE41867 DAY6 VS DAY8 LCMV ARMSTRONG EFFECTOR CD8 ...	201	26 (12.9)	26 (12.9)	0 (0)	0.054	<0.0001	0.0018
GSE13547 CTRL VS ANTI IGM STIM BCELL 12H UP	182	25 (13.7)	25 (13.7)	0 (0)	0.054	<0.0001	0.0018
GSE29618 PDC VS MDC DAY7 FLU VACCINE UP	194	25 (12.9)	24 (12.4)	1 (0.5)	0.053	<0.0001	0.0018

Gene Set Name	Gene Set Genes #	DE Genes N (%)	Up-reg. DE Genes N (%)	Down-reg. DE Genes N (%)	Jaccard Index	P-Value	FDR Adjusted P-Value
GSE24634 TREG VS TCONV POST DAY7 IL4 CONVERSION UP	196	25 (12.8)	25 (12.8)	0 (0)	0.053	<0.0001	0.0018
GOLDRATH NAIVE VS EFF CD8 TCELL DN	198	25 (12.6)	24 (12.1)	1 (0.5)	0.052	<0.0001	0.0018
GSE30962 PRIMARY VS SECONDARY ACUTE LCMV INF CD8 T...	199	25 (12.6)	25 (12.6)	0 (0)	0.052	<0.0001	0.0018
GSE33292 WT VS TCF1 KO DN3 THYMOCYTE DN	200	25 (12.5)	25 (12.5)	0 (0)	0.052	<0.0001	0.0018
GSE36476 CTRL VS TSST ACT 40H MEMORY CD4 TCELL OLD...	200	25 (12.5)	23 (11.5)	2 (1)	0.052	<0.0001	0.0018
GSE12366 PLASMA CELL VS NAIVE BCELL UP	188	24 (12.8)	23 (12.2)	1 (0.5)	0.051	<0.0001	0.0018
GSE13411 SWITCHED MEMORY BCELL VS PLASMA CELL DN	190	24 (12.6)	23 (12.1)	1 (0.5)	0.051	<0.0001	0.0018
GSE25088 WT VS STAT6 KO MACROPHAGE DN	196	24 (12.2)	24 (12.2)	0 (0)	0.051	<0.0001	0.0018
GSE21063 WT VS NFATC1 KO 8H ANTI IGM STIM BCELL UP	199	24 (12.1)	24 (12.1)	0 (0)	0.050	<0.0001	0.0018
GSE39110 DAY3 VS DAY6 POST IMMUNIZATION CD8 TCELL ...	201	24 (11.9)	24 (11.9)	0 (0)	0.050	<0.0001	0.0018
GSE29617 DAY3 VS DAY7 TIV FLU VACCINE PBMC 2008 DN	180	23 (12.8)	23 (12.8)	0 (0)	0.050	<0.0001	0.0018
GSE24634 IL4 VS CTRL TREATED NAIVE CD4 TCELL DAY7 ...	195	23 (11.8)	23 (11.8)	0 (0)	0.048	<0.0001	0.0018
GSE29618 BCELL VS PDC DAY7 FLU VACCINE DN	202	23 (11.4)	21 (10.4)	2 (1)	0.048	<0.0001	0.0018
GSE11386 NAIVE VS MEMORY BCELL UP	183	22 (12)	22 (12)	0 (0)	0.047	<0.0001	0.0018
GSE14415 TCONV VS FOXP3 KO INDUCED TREG DN	183	22 (12)	22 (12)	0 (0)	0.047	<0.0001	0.0018

Table 77: Enriched MSigDB Immunological Signatures (B Cells, Trivalent Influenza Vaccine, Day 7). Results are sorted by FDR adjusted p-value and Jaccard similarity index. Top 50 results are listed.

Gene Set Name	Gene Set Genes #	DE Genes N (%)	Up-reg. DE Genes N (%)	Down-reg. DE Genes N (%)	Jaccard Index	P-Value	FDR Adjusted P-Value
KEGG N GLYCAN BIOSYNTHESIS	46	9 (19.6)	9 (19.6)	0 (0)	0.026	<0.0001	0.0093
KEGG PROTEIN EXPORT	23	7 (30.4)	7 (30.4)	0 (0)	0.022	<0.0001	0.0093
KEGG CELL CYCLE	124	10 (8.1)	10 (8.1)	0 (0)	0.024	0.0002	0.0124
KEGG GLUTATHIONE METABOLISM	49	5 (10.2)	4 (8.2)	1 (2)	0.014	0.001	0.0465
KEGG P53 SIGNALING PATHWAY	68	6 (8.8)	6 (8.8)	0 (0)	0.016	0.0016	0.0478
KEGG VIBRIO CHOLERAE INFECTION	53	5 (9.4)	5 (9.4)	0 (0)	0.014	0.0015	0.0478
KEGG GLYCINE SERINE AND THREONINE METABOLISM	31	4 (12.9)	4 (12.9)	0 (0)	0.012	0.0018	0.0478
KEGG OOCYTE MEIOSIS	112	7 (6.2)	7 (6.2)	0 (0)	0.017	0.0043	0.1

Table 78: Enriched MSigDB KEGG Pathways (B Cells, Trivalent Influenza Vaccine, Day 7). Results are sorted by FDR adjusted p-value and Jaccard similarity index.

Gene Set Name	Gene Set Genes #	DE Genes N (%)	Up-reg. DE Genes N (%)	Down-reg. DE Genes N (%)	Jaccard Index	P-Value	FDR Adjusted P-Value
GO TRANSFERASE ACTIVITY TRANSFERRING HEXOSYL GROUP...	201	12 (6)	12 (6)	0 (0)	0.024	<0.0001	0.0372
GO UNFOLDED PROTEIN BINDING	94	8 (8.5)	8 (8.5)	0 (0)	0.021	0.0002	0.0372
GO INTRAMOLECULAR OXIDOREDUCTASE ACTIVITY TRANSPOS...	22	6 (27.3)	6 (27.3)	0 (0)	0.019	<0.0001	0.0372
GO INTRAMOLECULAR OXIDOREDUCTASE ACTIVITY	53	6 (11.3)	6 (11.3)	0 (0)	0.017	0.0002	0.0372
GO MISFOLDED PROTEIN BINDING	12	4 (33.3)	4 (33.3)	0 (0)	0.013	0.0002	0.0372
GO TRANSFERASE ACTIVITY TRANSFERRING GLYCOSYL GROU...	279	13 (4.7)	13 (4.7)	0 (0)	0.023	0.0003	0.0464
GO ISOMERASE ACTIVITY	161	9 (5.6)	9 (5.6)	0 (0)	0.020	0.0004	0.0464
GO CHAPERONE BINDING	81	7 (8.6)	7 (8.6)	0 (0)	0.019	0.0004	0.0464

Gene Set Name	Gene Set Genes #	DE Genes N (%)	Up-reg. DE Genes N (%)	Down-reg. DE Genes N (%)	Jaccard Index	P-Value	FDR Adjusted P-Value
GO CYSTEINE TYPE ENDOPEPTIDASE INHIBITOR ACTIVITY	55	5 (9.1)	4 (7.3)	1 (1.8)	0.014	0.0006	0.0619

Table 79: Enriched MSigDB Molecular Functions (B Cells, Trivalent Influenza Vaccine, Day 7). Results are sorted by FDR adjusted p-value and Jaccard similarity index.

Gene Set Name	Gene Set Genes #	DE Genes N (%)	Up-reg. DE Genes N (%)	Down-reg. DE Genes N (%)	Jaccard Index	P-Value	FDR Adjusted P-Value
REACTOME DIABETES PATHWAYS	129	16 (12.4)	16 (12.4)	0 (0)	0.038	<0.0001	0.0045
REACTOME UNFOLDED PROTEIN RESPONSE	78	14 (17.9)	14 (17.9)	0 (0)	0.038	<0.0001	0.0045
REACTOME ASPARAGINE N LINKED GLYCOSYLATION	81	14 (17.3)	14 (17.3)	0 (0)	0.038	<0.0001	0.0045
REACTOME POST TRANSLATIONAL PROTEIN MODIFICATION	181	16 (8.8)	16 (8.8)	0 (0)	0.034	<0.0001	0.0045
REACTOME METABOLISM OF PROTEINS	424	24 (5.7)	24 (5.7)	0 (0)	0.034	<0.0001	0.0045
REACTOME CELL CYCLE MITOTIC	308	20 (6.5)	20 (6.5)	0 (0)	0.034	<0.0001	0.0045
REACTOME ACTIVATION OF CHAPERONE GENES BY XBP1S	46	11 (23.9)	11 (23.9)	0 (0)	0.033	<0.0001	0.0045
REACTOME CELL CYCLE	400	21 (5.2)	21 (5.2)	0 (0)	0.031	<0.0001	0.0045
REACTOME SRP DEPENDENT COTRANSLATIONAL PROTEIN TAR...	108	11 (10.2)	11 (10.2)	0 (0)	0.028	<0.0001	0.0045
REACTOME TRANSLATION	145	11 (7.6)	11 (7.6)	0 (0)	0.025	<0.0001	0.0045
REACTOME E2F MEDIATED REGULATION OF DNA REPLICATIO...	33	7 (21.2)	7 (21.2)	0 (0)	0.021	<0.0001	0.0045
REACTOME G1 S SPECIFIC TRANSCRIPTION	17	6 (35.3)	6 (35.3)	0 (0)	0.019	<0.0001	0.0045
REACTOME TRANSPORT TO THE GOLGI AND SUBSEQUENT MOD...	33	6 (18.2)	6 (18.2)	0 (0)	0.018	<0.0001	0.0045
REACTOME ACTIVATION OF CHAPERONE GENES BY ATF6 ALP...	9	4 (44.4)	4 (44.4)	0 (0)	0.013	<0.0001	0.0045
REACTOME ACTIVATION OF CHAPERONES BY ATF6 ALPHA	11	4 (36.4)	4 (36.4)	0 (0)	0.013	<0.0001	0.0045
REACTOME CELL CYCLE CHECKPOINTS	112	9 (8)	9 (8)	0 (0)	0.022	0.0003	0.0126
REACTOME G2 M CHECKPOINTS	42	6 (14.3)	6 (14.3)	0 (0)	0.018	0.0004	0.015
REACTOME AMINO ACID SYNTHESIS AND INTERCONVERSION ...	16	4 (25)	4 (25)	0 (0)	0.013	0.0004	0.015
REACTOME REGULATION OF MITOTIC CELL CYCLE	77	7 (9.1)	7 (9.1)	0 (0)	0.019	0.0005	0.0177
REACTOME MITOTIC M M G1 PHASES	168	11 (6.5)	11 (6.5)	0 (0)	0.024	0.0007	0.0236
REACTOME E2F ENABLED INHIBITION OF PRE REPLICATION...	10	3 (30)	3 (30)	0 (0)	0.010	0.0009	0.0289
REACTOME DNA REPLICATION	188	11 (5.9)	11 (5.9)	0 (0)	0.023	0.0011	0.0337
REACTOME MITOTIC PROMETAPHASE	86	7 (8.1)	7 (8.1)	0 (0)	0.018	0.0019	0.0557
REACTOME MITOTIC G1 G1 S PHASES	130	8 (6.2)	8 (6.2)	0 (0)	0.019	0.0039	0.0973
REACTOME G1 S TRANSITION	106	7 (6.6)	7 (6.6)	0 (0)	0.017	0.0039	0.0973
REACTOME CYCLIN A B1 ASSOCIATED EVENTS DURING G2 M...	15	3 (20)	3 (20)	0 (0)	0.009	0.0038	0.0973
REACTOME APC C CDC20 MEDIATED DEGRADATION OF CYCLI...	19	3 (15.8)	3 (15.8)	0 (0)	0.009	0.0039	0.0973

Table 80: Enriched MSigDB Reactome Pathways (B Cells, Trivalent Influenza Vaccine, Day 7). Results are sorted by FDR adjusted p-value and Jaccard similarity index.

Gene Set Name	Gene Set Genes #	DE Genes N (%)	Up-reg. DE Genes N (%)	Down-reg. DE Genes N (%)	Jaccard Index	P-Value	FDR Adjusted P-Value
Plasma cell surface signature (S3)	23	7 (30.4)	7 (30.4)	0 (0)	0.051	<0.0001	0.0173
plasma cells, immunoglobulins (M156.1)	22	5 (22.7)	5 (22.7)	0 (0)	0.036	<0.0001	0.0173

Table 81: Enriched Blood Transcription Modules (B Cells, Trivalent Influenza Vaccine, Day 8). Results are sorted by FDR adjusted p-value and Jaccard similarity index.

Gene Set Name	Gene Set Genes #	DE Genes N (%)	Up-reg. DE Genes N (%)	Down-reg. DE Genes N (%)	Jaccard Index	P-Value	FDR Adjusted P-Value
GO RESPONSE TO ENDOPLASMIC RETICULUM STRESS	234	15 (6.4)	15 (6.4)	0 (0)	0.044	<0.0001	0.031
GO CELLULAR RESPONSE TO TOPOLOGICALLY INCORRECT PR...	123	10 (8.1)	10 (8.1)	0 (0)	0.043	<0.0001	0.031
GO RESPONSE TO TOPOLOGICALLY INCORRECT PROTEIN	164	11 (6.7)	11 (6.7)	0 (0)	0.040	<0.0001	0.031
GO ER ASSOCIATED UBIQUITIN DEPENDENT PROTEIN CATAB...	61	7 (11.5)	7 (11.5)	0 (0)	0.040	<0.0001	0.031
GO CELL REDOX HOMEOSTASIS	67	7 (10.4)	7 (10.4)	0 (0)	0.039	<0.0001	0.031
GO ERAD PATHWAY	74	7 (9.5)	7 (9.5)	0 (0)	0.037	<0.0001	0.031
GO IRE1 MEDIATED UNFOLDED PROTEIN RESPONSE	57	6 (10.5)	6 (10.5)	0 (0)	0.035	<0.0001	0.031
GO PROTEIN FOLDING	217	11 (5.1)	11 (5.1)	0 (0)	0.034	<0.0001	0.031
GO PROTEIN EXIT FROM ENDOPLASMIC RETICULUM	20	4 (20)	4 (20)	0 (0)	0.029	<0.0001	0.031
GO ER NUCLEUS SIGNALING PATHWAY	34	4 (11.8)	4 (11.8)	0 (0)	0.026	<0.0001	0.031
GO PROTEASOMAL PROTEIN CATABOLIC PROCESS	272	8 (2.9)	8 (2.9)	0 (0)	0.021	<0.0001	0.031
GO CELLULAR HOMEOSTASIS	678	16 (2.4)	16 (2.4)	0 (0)	0.020	<0.0001	0.031
GO CELLULAR RESPONSE TO STRESS	1563	23 (1.5)	21 (1.3)	2 (0.1)	0.014	<0.0001	0.031
GO HOMEOSTATIC PROCESS	1333	19 (1.4)	19 (1.4)	0 (0)	0.013	<0.0001	0.031
GO CELLULAR RESPONSE TO ORGANIC SUBSTANCE	1848	23 (1.2)	22 (1.2)	1 (0.1)	0.012	<0.0001	0.031
GO RETROGRADE PROTEIN TRANSPORT ER TO CYTOSOL	16	3 (18.8)	3 (18.8)	0 (0)	0.022	0.0003	0.0872

Table 82: Enriched MSigDB Biological Processes (B Cells, Trivalent Influenza Vaccine, Day 8). Results are sorted by FDR adjusted p-value and Jaccard similarity index.

Gene Set Name	Gene Set Genes #	DE Genes N (%)	Up-reg. DE Genes N (%)	Down-reg. DE Genes N (%)	Jaccard Index	P-Value	FDR Adjusted P-Value
GO ENDOPLASMIC RETICULUM CHAPERONE COMPLEX	11	5 (45.5)	5 (45.5)	0 (0)	0.039	<0.0001	0.0117
GO ENDOPLASMIC RETICULUM LUMEN	202	12 (5.9)	12 (5.9)	0 (0)	0.039	<0.0001	0.0117
GO ENDOPLASMIC RETICULUM PART	1162	33 (2.8)	33 (2.8)	0 (0)	0.026	<0.0001	0.0117
GO NUCLEAR OUTER MEMBRANE ENDOPLASMIC RETICULUM ME...	1005	26 (2.6)	26 (2.6)	0 (0)	0.024	<0.0001	0.0117
GO ENDOPLASMIC RETICULUM	1631	39 (2.4)	39 (2.4)	0 (0)	0.023	<0.0001	0.0117
GO PIGMENT GRANULE	102	5 (4.9)	5 (4.9)	0 (0)	0.023	0.0003	0.0292
GO ENDOPLASMIC RETICULUM GOLGI INTERMEDIATE COMPAR...	106	5 (4.7)	5 (4.7)	0 (0)	0.022	0.0007	0.0584
GO ROUGH ENDOPLASMIC RETICULUM	71	4 (5.6)	4 (5.6)	0 (0)	0.021	0.0009	0.0657
GO SMOOTH ENDOPLASMIC RETICULUM	33	3 (9.1)	3 (9.1)	0 (0)	0.020	0.0012	0.0779

Table 83: Enriched MSigDB Cellular Components (B Cells, Trivalent Influenza Vaccine, Day 8). Results are sorted by FDR adjusted p-value and Jaccard similarity index.

Gene Set Name	Gene Set Genes #	DE Genes N (%)	Up-reg. DE Genes N (%)	Down-reg. DE Genes N (%)	Jaccard Index	P-Value	FDR Adjusted P-Value
GSE29614 DAY3 VS DAY7 TIV FLU VACCINE PBMC DN	179	39 (21.8)	39 (21.8)	0 (0)	0.149	<0.0001	0.008
GSE29614 CTRL VS DAY7 TIV FLU VACCINE PBMC DN	183	35 (19.1)	35 (19.1)	0 (0)	0.130	<0.0001	0.008
GSE29164 CD8 TCELL VS CD8 TCELL AND IL12 TREATED M...	188	31 (16.5)	31 (16.5)	0 (0)	0.112	<0.0001	0.008
GSE22886 NAIVE BCELL VS BM PLASMA CELL DN	195	24 (12.3)	24 (12.3)	0 (0)	0.082	<0.0001	0.008
GSE29617 CTRL VS DAY7 TIV FLU VACCINE PBMC 2008 DN	187	23 (12.3)	23 (12.3)	0 (0)	0.081	<0.0001	0.008
GSE13411 PLASMA CELL VS MEMORY BCELL UP	190	22 (11.6)	22 (11.6)	0 (0)	0.076	<0.0001	0.008
GSE12845 IGD POS VS NEG BLOOD BCELL DN	186	20 (10.8)	20 (10.8)	0 (0)	0.070	<0.0001	0.008

Gene Set Name	Gene Set Genes #	DE Genes N (%)	Up-reg. DE Genes N (%)	Down-reg. DE Genes N (%)	Jaccard Index	P-Value	FDR Adjusted P-Value
GSE29617 DAY3 VS DAY7 TIV FLU VACCINE PBMC 2008 DN	180	18 (10)	18 (10)	0 (0)	0.064	<0.0001	0.008
GSE22886 IGG IGA MEMORY BCELL VS BM PLASMA CELL DN	190	17 (8.9)	17 (8.9)	0 (0)	0.058	<0.0001	0.008
GSE20727 CTRL VS ROS INHIBITOR TREATED DC DN	191	17 (8.9)	17 (8.9)	0 (0)	0.058	<0.0001	0.008
GSE22886 NAIVE BCELL VS BLOOD PLASMA CELL DN	194	16 (8.2)	16 (8.2)	0 (0)	0.053	<0.0001	0.008
GSE29614 CTRL VS TIV FLU VACCINE PBMC 2007 DN	172	14 (8.1)	14 (8.1)	0 (0)	0.050	<0.0001	0.008
GSE40273 EOS KO VS WT TREG UP	199	15 (7.5)	15 (7.5)	0 (0)	0.049	<0.0001	0.008
GSE29618 PDC VS MDC DAY7 FLU VACCINE UP	194	14 (7.2)	14 (7.2)	0 (0)	0.046	<0.0001	0.008
GSE10325 BCELL VS LUPUS BCELL DN	200	14 (7)	14 (7)	0 (0)	0.046	<0.0001	0.008
GSE41867 DAY6 VS DAY8 LCMV ARMSTRONG EFFECTOR CD8 ...	201	14 (7)	14 (7)	0 (0)	0.045	<0.0001	0.008
GSE13411 NAIVE BCELL VS PLASMA CELL DN	188	13 (6.9)	13 (6.9)	0 (0)	0.044	<0.0001	0.008
GSE13411 IGM MEMORY BCELL VS PLASMA CELL DN	190	13 (6.8)	13 (6.8)	0 (0)	0.044	<0.0001	0.008
GSE40666 UNTREATED VS IFNA STIM STAT4 KO EFFECTOR ...	196	13 (6.6)	13 (6.6)	0 (0)	0.043	<0.0001	0.008
GSE22886 IGM MEMORY BCELL VS BM PLASMA CELL DN	194	12 (6.2)	12 (6.2)	0 (0)	0.040	<0.0001	0.008
GSE339 CD4POS VS CD8POS DC DN	199	12 (6)	12 (6)	0 (0)	0.039	<0.0001	0.008
GSE29618 MONOCYTE VS PDC DAY7 FLU VACCINE DN	197	11 (5.6)	11 (5.6)	0 (0)	0.036	<0.0001	0.008
GSE3982 CENT MEMORY CD4 TCELL VS TH1 DN	200	11 (5.5)	10 (5)	1 (0.5)	0.035	<0.0001	0.008
GSE29618 BCELL VS PDC DAY7 FLU VACCINE DN	202	11 (5.4)	11 (5.4)	0 (0)	0.035	<0.0001	0.008
GSE12366 PLASMA CELL VS MEMORY BCELL UP	185	10 (5.4)	10 (5.4)	0 (0)	0.034	<0.0001	0.008
GSE12366 GC BCELL VS PLASMA CELL DN	186	10 (5.4)	10 (5.4)	0 (0)	0.034	<0.0001	0.008
GSE22886 IGG IGA MEMORY BCELL VS BLOOD PLASMA CELL...	197	10 (5.1)	10 (5.1)	0 (0)	0.033	<0.0001	0.008
GSE29618 PDC VS MDC UP	197	10 (5.1)	10 (5.1)	0 (0)	0.033	<0.0001	0.008
GSE40274 CTRL VS FOXP3 AND HELIOS TRANSDUCED ACTIV...	198	10 (5.1)	10 (5.1)	0 (0)	0.032	<0.0001	0.008
GSE29618 BCELL VS PDC DN	200	10 (5)	10 (5)	0 (0)	0.032	<0.0001	0.008
GSE43260 BTLA POS VS NEG INTRATUMORAL CD8 TCELL UP	200	10 (5)	10 (5)	0 (0)	0.032	<0.0001	0.008
GSE32164 RESTING DIFFERENTIATED VS ALTERNATIVELY A...	202	10 (5)	9 (4.5)	1 (0.5)	0.032	<0.0001	0.008
GSE12366 PLASMA CELL VS NAIVE BCELL UP	188	9 (4.8)	9 (4.8)	0 (0)	0.030	<0.0001	0.008
GSE13411 SWITCHED MEMORY BCELL VS PLASMA CELL DN	190	9 (4.7)	9 (4.7)	0 (0)	0.030	<0.0001	0.008
GSE29618 MONOCYTE VS PDC DN	192	9 (4.7)	9 (4.7)	0 (0)	0.030	<0.0001	0.008
GSE21670 UNTREATED VS TGFB TREATED STAT3 KO CD4 TC...	197	9 (4.6)	9 (4.6)	0 (0)	0.029	<0.0001	0.008
GSE41867 MEMORY VS EXHAUSTED CD8 TCELL DAY30 LCMV ...	198	9 (4.5)	9 (4.5)	0 (0)	0.029	<0.0001	0.008
GSE40274 FOXP3 VS FOXP3 AND EOS TRANSDUCED ACTIVAT...	202	9 (4.5)	8 (4)	1 (0.5)	0.029	<0.0001	0.008
GSE6269 E COLI VS STAPH AUREUS INF PBMC DN	174	8 (4.6)	8 (4.6)	0 (0)	0.028	<0.0001	0.008
GSE22886 NAIVE VS IGG IGA MEMORY BCELL DN	193	8 (4.1)	7 (3.6)	1 (0.5)	0.026	<0.0001	0.008
GSE29617 CTRL VS TIV FLU VACCINE PBMC 2008 DN	196	8 (4.1)	8 (4.1)	0 (0)	0.026	<0.0001	0.008
GSE29164 DAY3 VS DAY7 UNTREATED MELANOMA DN	199	8 (4)	8 (4)	0 (0)	0.026	<0.0001	0.008
GSE32164 ALTERNATIVELY ACT M2 VS CMYC INHIBITED MA...	200	8 (4)	8 (4)	0 (0)	0.026	<0.0001	0.008
GSE7460 FOXP3 MUT VS WT ACT WITH TGFB TCONV UP	200	8 (4)	8 (4)	0 (0)	0.026	<0.0001	0.008
GSE15330 HSC VS LYMPHOID PRIMED MULTIPOTENT PROGEN...	183	7 (3.8)	7 (3.8)	0 (0)	0.024	<0.0001	0.008
GSE17974 IL4 AND ANTI IL12 VS UNTREATED 12H ACT CD...	192	7 (3.6)	7 (3.6)	0 (0)	0.023	<0.0001	0.008
GSE22886 NAIVE VS IGM MEMORY BCELL DN	195	7 (3.6)	6 (3.1)	1 (0.5)	0.023	<0.0001	0.008
GSE24634 IL4 VS CTRL TREATED NAIVE CD4 TCELL DAY3 ...	198	7 (3.5)	7 (3.5)	0 (0)	0.022	<0.0001	0.008
GSE360 CTRL VS M TUBERCULOSIS DC DN	199	7 (3.5)	7 (3.5)	0 (0)	0.022	<0.0001	0.008
GOLDRATH NAIVE VS MEMORY CD8 TCELL DN	200	7 (3.5)	7 (3.5)	0 (0)	0.022	<0.0001	0.008

Table 84: Enriched MSigDB Immunological Signatures (B Cells, Trivalent Influenza Vaccine, Day 8). Results are sorted by FDR adjusted p-value and Jaccard similarity index. Top 50 results are listed.

Gene Set Name	Gene Set Genes #	DE Genes N (%)	Up-reg. DE Genes N (%)	Down-reg. DE Genes N (%)	Jaccard Index	P-Value	FDR Adjusted P-Value
KEGG PROTEIN EXPORT	23	3 (13)	3 (13)	0 (0)	0.021	0.0003	0.0558

Table 85: Enriched MSigDB KEGG Pathways (B Cells, Trivalent Influenza Vaccine, Day 8). Results are sorted by FDR adjusted p-value and Jaccard similarity index.

Gene Set Name	Gene Set Genes #	DE Genes N (%)	Up-reg. DE Genes N (%)	Down-reg. DE Genes N (%)	Jaccard Index	P-Value	FDR Adjusted P-Value
GO INTRAMOLECULAR OXIDOREDUCTASE ACTIVITY TRANSPOS...	22	4 (18.2)	4 (18.2)	0 (0)	0.029	<0.0001	0.031
GO UNFOLDED PROTEIN BINDING	94	6 (6.4)	6 (6.4)	0 (0)	0.029	<0.0001	0.031
GO ISOMERASE ACTIVITY	161	6 (3.7)	6 (3.7)	0 (0)	0.022	<0.0001	0.031
GO INTRAMOLECULAR OXIDOREDUCTASE ACTIVITY	53	4 (7.5)	4 (7.5)	0 (0)	0.024	0.0002	0.0372
GO G PROTEIN COUPLED CHEMOATTRACTANT RECEPTOR ACTI...	25	3 (12)	3 (12)	0 (0)	0.021	0.0002	0.0372
GO MISFOLDED PROTEIN BINDING	12	3 (25)	3 (25)	0 (0)	0.023	0.0003	0.0464

Table 86: Enriched MSigDB Molecular Functions (B Cells, Trivalent Influenza Vaccine, Day 8). Results are sorted by FDR adjusted p-value and Jaccard similarity index.

Gene Set Name	Gene Set Genes #	DE Genes N (%)	Up-reg. DE Genes N (%)	Down-reg. DE Genes N (%)	Jaccard Index	P-Value	FDR Adjusted P-Value
REACTOME UNFOLDED PROTEIN RESPONSE	78	6 (7.7)	6 (7.7)	0 (0)	0.031	<0.0001	0.0168
REACTOME DIABETES PATHWAYS	129	7 (5.4)	7 (5.4)	0 (0)	0.029	<0.0001	0.0168
REACTOME ACTIVATION OF CHAPERONE GENES BY ATF6 ALP...	9	3 (33.3)	3 (33.3)	0 (0)	0.024	<0.0001	0.0168
REACTOME ACTIVATION OF CHAPERONES BY ATF6 ALPHA	11	3 (27.3)	3 (27.3)	0 (0)	0.023	<0.0001	0.0168
REACTOME ASPARAGINE N LINKED GLYCOSYLATION	81	5 (6.2)	5 (6.2)	0 (0)	0.025	0.0002	0.027
REACTOME ACTIVATION OF CHAPERONE GENES BY XBP1S	46	4 (8.7)	4 (8.7)	0 (0)	0.025	0.0003	0.0337

Table 87: Enriched MSigDB Reactome Pathways (B Cells, Trivalent Influenza Vaccine, Day 8). Results are sorted by FDR adjusted p-value and Jaccard similarity index.

Gene Set Name	Gene Set Genes #	DE Genes N (%)	Up-reg. DE Genes N (%)	Down-reg. DE Genes N (%)	Jaccard Index	P-Value	FDR Adjusted P-Value
GO OXYGEN TRANSPORT	15	3 (20)	3 (20)	0 (0)	0.115	<0.0001	0.0291
GO GAS TRANSPORT	19	3 (15.8)	3 (15.8)	0 (0)	0.100	<0.0001	0.0291
GO HYDROGEN PEROXIDE CATABOLIC PROCESS	20	3 (15)	3 (15)	0 (0)	0.097	<0.0001	0.0291
GO T CELL MIGRATION	14	2 (14.3)	2 (14.3)	0 (0)	0.077	<0.0001	0.0291
GO HYDROGEN PEROXIDE METABOLIC PROCESS	30	3 (10)	3 (10)	0 (0)	0.073	<0.0001	0.0291
GO BICARBONATE TRANSPORT	44	3 (6.8)	3 (6.8)	0 (0)	0.054	<0.0001	0.0291
GO LYMPHOCYTE CHEMOTAXIS	37	2 (5.4)	2 (5.4)	0 (0)	0.041	<0.0001	0.0291
GO RESPONSE TO HYDROGEN PEROXIDE	109	4 (3.7)	4 (3.7)	0 (0)	0.034	<0.0001	0.0291
GO REACTIVE OXYGEN SPECIES METABOLIC PROCESS	96	3 (3.1)	3 (3.1)	0 (0)	0.028	<0.0001	0.0291
GO PROTEIN HETEROOLIGOMERIZATION	113	3 (2.7)	3 (2.7)	0 (0)	0.024	<0.0001	0.0291
GO RESPONSE TO REACTIVE OXYGEN SPECIES	191	4 (2.1)	4 (2.1)	0 (0)	0.020	<0.0001	0.0291
GO RESPONSE TO TOXIC SUBSTANCE	241	4 (1.7)	4 (1.7)	0 (0)	0.016	<0.0001	0.0291

Gene Set Name	Gene Set Genes #	DE Genes N (%)	Up-reg. DE Genes N (%)	Down-reg. DE Genes N (%)	Jaccard Index	P-Value	FDR Adjusted P-Value
GO RECEPTOR MEDIATED ENDOCYTOSIS	213	3 (1.4)	3 (1.4)	0 (0)	0.013	<0.0001	0.0291
GO RESPONSE TO OXIDATIVE STRESS	352	4 (1.1)	4 (1.1)	0 (0)	0.011	<0.0001	0.0291
GO POSITIVE REGULATION OF CELL DEATH	606	5 (0.8)	5 (0.8)	0 (0)	0.008	<0.0001	0.0291
GO RESPONSE TO INORGANIC SUBSTANCE	484	4 (0.8)	4 (0.8)	0 (0)	0.008	<0.0001	0.0291
GO DETOXIFICATION	76	3 (3.9)	3 (3.9)	0 (0)	0.035	0.0002	0.0547
GO LYMPHOCYTE MIGRATION	48	2 (4.2)	2 (4.2)	0 (0)	0.033	0.0004	0.0979
GO RESPONSE TO OXYGEN CONTAINING COMPOUND	1385	5 (0.4)	5 (0.4)	0 (0)	0.004	0.0004	0.0979

Table 88: Enriched MSigDB Biological Processes (B Cells, Trivalent Influenza Vaccine, Day 9). Results are sorted by FDR adjusted p-value and Jaccard similarity index.

Gene Set Name	Gene Set Genes #	DE Genes N (%)	Up-reg. DE Genes N (%)	Down-reg. DE Genes N (%)	Jaccard Index	P-Value	FDR Adjusted P-Value
GO HEMOGLOBIN COMPLEX	12	3 (25)	3 (25)	0 (0)	0.130	<0.0001	0.0146
GO ENDOCYTIC VESICLE LUMEN	17	3 (17.6)	3 (17.6)	0 (0)	0.107	<0.0001	0.0146
GO VESICLE LUMEN	106	3 (2.8)	3 (2.8)	0 (0)	0.026	<0.0001	0.0146
GO BLOOD MICROPARTICLE	118	3 (2.5)	3 (2.5)	0 (0)	0.023	<0.0001	0.0146
GO CYTOSOLIC SMALL RIBOSOMAL SUBUNIT	43	2 (4.7)	2 (4.7)	0 (0)	0.036	0.0006	0.0584
GO CYTOSOLIC PART	220	3 (1.4)	3 (1.4)	0 (0)	0.013	0.0007	0.0584
GO ENDOCYTIC VESICLE	255	3 (1.2)	3 (1.2)	0 (0)	0.011	0.0006	0.0584
GO SMALL RIBOSOMAL SUBUNIT	67	2 (3)	2 (3)	0 (0)	0.025	0.0009	0.0657

Table 89: Enriched MSigDB Cellular Components (B Cells, Trivalent Influenza Vaccine, Day 9). Results are sorted by FDR adjusted p-value and Jaccard similarity index.

Gene Set Name	Gene Set Genes #	DE Genes N (%)	Up-reg. DE Genes N (%)	Down-reg. DE Genes N (%)	Jaccard Index	P-Value	FDR Adjusted P-Value
GO OXYGEN TRANSPORTER ACTIVITY	14	3 (21.4)	3 (21.4)	0 (0)	0.120	<0.0001	0.0186
GO OXIDOREDUCTASE ACTIVITY ACTING ON PEROXIDE AS A...	41	3 (7.3)	3 (7.3)	0 (0)	0.058	<0.0001	0.0186
GO OXYGEN BINDING	47	3 (6.4)	3 (6.4)	0 (0)	0.052	<0.0001	0.0186
GO ANTIOXIDANT ACTIVITY	69	3 (4.3)	3 (4.3)	0 (0)	0.037	<0.0001	0.0186
GO TETRAPYRROLE BINDING	133	3 (2.3)	3 (2.3)	0 (0)	0.021	<0.0001	0.0186
GO CHEMOKINE BINDING	21	2 (9.5)	2 (9.5)	0 (0)	0.061	0.0002	0.0232
GO G PROTEIN COUPLED CHEMOATTRACTANT RECEPTOR ACTI...	25	2 (8)	2 (8)	0 (0)	0.054	0.0002	0.0232
GO IRON ION BINDING	162	3 (1.9)	3 (1.9)	0 (0)	0.017	0.0002	0.0232
GO OXIDOREDUCTASE ACTIVITY	712	4 (0.6)	4 (0.6)	0 (0)	0.005	0.0005	0.0516
GO CYTOKINE BINDING	92	2 (2.2)	2 (2.2)	0 (0)	0.019	0.001	0.0929

Table 90: Enriched MSigDB Molecular Functions (B Cells, Trivalent Influenza Vaccine, Day 9). Results are sorted by FDR adjusted p-value and Jaccard similarity index.

Gene Set Name	Gene Set Genes #	DE Genes N (%)	Up-reg. DE Genes N (%)	Down-reg. DE Genes N (%)	Jaccard Index	P-Value	FDR Adjusted P-Value
enriched in monocytes (II) (M11.0)	183	22 (12)	22 (12)	0 (0)	0.074	<0.0001	0.0058
Monocyte surface signature (S4)	84	11 (13.1)	11 (13.1)	0 (0)	0.053	<0.0001	0.0058
cell cycle and transcription (M4.0)	318	19 (6)	19 (6)	0 (0)	0.044	<0.0001	0.0058
enriched in activated dendritic cells (II) (M165)	35	7 (20)	7 (20)	0 (0)	0.043	<0.0001	0.0058
TLR and inflammatory signaling (M16)	43	7 (16.3)	7 (16.3)	0 (0)	0.041	<0.0001	0.0058
formyl peptide receptor mediated neutrophil respon...	10	5 (50)	5 (50)	0 (0)	0.036	<0.0001	0.0058
suppression of MAPK signaling (M56)	12	4 (33.3)	4 (33.3)	0 (0)	0.028	0.0002	0.0099
enriched in neutrophils (I) (M37.1)	47	6 (12.8)	5 (10.6)	1 (2.1)	0.034	0.0003	0.0115
antiviral IFN signature (M75)	22	5 (22.7)	5 (22.7)	0 (0)	0.033	0.0003	0.0115
myeloid cell enriched receptors and transporters (...	30	5 (16.7)	5 (16.7)	0 (0)	0.031	0.0007	0.0242
enriched in activated dendritic cells/monocytes (M...	16	4 (25)	3 (18.8)	1 (6.2)	0.027	0.0008	0.0252
RIG-1 like receptor signaling (M68)	9	3 (33.3)	3 (33.3)	0 (0)	0.021	0.001	0.0288
G protein coupled receptors cluster (M155)	10	3 (30)	3 (30)	0 (0)	0.021	0.0013	0.0346
enriched in monocytes (III) (M73)	11	3 (27.3)	3 (27.3)	0 (0)	0.021	0.0019	0.047
type I interferon response (M127)	12	3 (25)	3 (25)	0 (0)	0.021	0.0026	0.06
innate antiviral response (M150)	12	3 (25)	3 (25)	0 (0)	0.021	0.0032	0.0692
chemokines and inflammatory molecules in myeloid c...	18	3 (16.7)	2 (11.1)	1 (5.6)	0.020	0.0049	0.0997

Table 91: Enriched Blood Transcription Modules (PBMC, Trivalent Influenza Vaccine, Day 1). Results are sorted by FDR adjusted p-value and Jaccard similarity index.

Gene Set Name	Gene Set Genes #	DE Genes N (%)	Up-reg. DE Genes N (%)	Down-reg. DE Genes N (%)	Jaccard Index	P-Value	FDR Adjusted P-Value
GO INNATE IMMUNE RESPONSE	589	27 (4.6)	25 (4.2)	2 (0.3)	0.039	<0.0001	0.0122
GO DEFENSE RESPONSE TO VIRUS	165	11 (6.7)	11 (6.7)	0 (0)	0.038	<0.0001	0.0122
GO RESPONSE TO INTERFERON GAMMA	140	10 (7.1)	9 (6.4)	1 (0.7)	0.038	<0.0001	0.0122
GO INTERFERON GAMMA MEDIATED SIGNALING PATHWAY	67	7 (10.4)	7 (10.4)	0 (0)	0.036	<0.0001	0.0122
GO RESPONSE TO TYPE I INTERFERON	67	7 (10.4)	7 (10.4)	0 (0)	0.036	<0.0001	0.0122
GO RESPONSE TO VIRUS	248	13 (5.2)	13 (5.2)	0 (0)	0.035	<0.0001	0.0122
GO CELLULAR RESPONSE TO INTERFERON GAMMA	118	8 (6.8)	7 (5.9)	1 (0.8)	0.033	<0.0001	0.0122
GO RESPONSE TO HEAT	89	7 (7.9)	7 (7.9)	0 (0)	0.032	<0.0001	0.0122
GO IMMUNE RESPONSE	1052	37 (3.5)	35 (3.3)	2 (0.2)	0.032	<0.0001	0.0122
GO CYTOKINE MEDIATED SIGNALING PATHWAY	448	18 (4)	17 (3.8)	1 (0.2)	0.032	<0.0001	0.0122
GO IMMUNE EFFECTOR PROCESS	456	18 (3.9)	17 (3.7)	1 (0.2)	0.031	<0.0001	0.0122
GO DEFENSE RESPONSE	1197	40 (3.3)	37 (3.1)	3 (0.3)	0.031	<0.0001	0.0122
GO REGULATION OF ACUTE INFLAMMATORY RESPONSE	74	6 (8.1)	6 (8.1)	0 (0)	0.030	<0.0001	0.0122
GO POSITIVE REGULATION OF LEUKOCYTE MIGRATION	109	7 (6.4)	6 (5.5)	1 (0.9)	0.029	<0.0001	0.0122
GO RESPONSE TO TEMPERATURE STIMULUS	148	8 (5.4)	8 (5.4)	0 (0)	0.029	<0.0001	0.0122
GO DEFENSE RESPONSE TO OTHER ORGANISM	483	17 (3.5)	16 (3.3)	1 (0.2)	0.028	<0.0001	0.0122
GO REGULATION OF FEVER GENERATION	11	4 (36.4)	4 (36.4)	0 (0)	0.028	<0.0001	0.0122
GO NEGATIVE REGULATION OF VIRAL GENOME REPLICATION	49	5 (10.2)	5 (10.2)	0 (0)	0.028	<0.0001	0.0122
GO POSITIVE REGULATION OF MONOCYTE CHEMOTAXIS	15	4 (26.7)	4 (26.7)	0 (0)	0.027	<0.0001	0.0122
GO REGULATION OF HEAT GENERATION	15	4 (26.7)	4 (26.7)	0 (0)	0.027	<0.0001	0.0122
GO RESPONSE TO CYTOKINE	710	22 (3.1)	21 (3)	1 (0.1)	0.027	<0.0001	0.0122
GO POSITIVE REGULATION OF CYTOKINE SECRETION	96	6 (6.2)	6 (6.2)	0 (0)	0.027	<0.0001	0.0122
GO REGULATION OF MONOCYTE CHEMOTAXIS	20	4 (20)	4 (20)	0 (0)	0.026	<0.0001	0.0122

Gene Set Name	Gene Set Genes #	DE Genes N (%)	Up-reg. DE Genes N (%)	Down-reg. DE Genes N (%)	Jaccard Index	P-Value	FDR Adjusted P-Value
GO REGULATION OF LEUKOCYTE MIGRATION	148	7 (4.7)	6 (4.1)	1 (0.7)	0.025	<0.0001	0.0122
GO CELLULAR RESPONSE TO CYTOKINE STIMULUS	602	18 (3)	17 (2.8)	1 (0.2)	0.025	<0.0001	0.0122
GO REGULATION OF IMMUNE RESPONSE	821	23 (2.8)	21 (2.6)	2 (0.2)	0.025	<0.0001	0.0122
GO INFLAMMATORY RESPONSE	451	13 (2.9)	12 (2.7)	1 (0.2)	0.023	<0.0001	0.0122
GO POSITIVE REGULATION OF DEFENSE RESPONSE	363	11 (3)	10 (2.8)	1 (0.3)	0.023	<0.0001	0.0122
GO RESPONSE TO BIOTIC STIMULUS	863	22 (2.5)	21 (2.4)	1 (0.1)	0.022	<0.0001	0.0122
GO POSITIVE REGULATION OF IMMUNE SYSTEM PROCESS	833	21 (2.5)	20 (2.4)	1 (0.1)	0.022	<0.0001	0.0122
GO REGULATION OF DEFENSE RESPONSE	757	19 (2.5)	18 (2.4)	1 (0.1)	0.022	<0.0001	0.0122
GO POSITIVE REGULATION OF IMMUNE RESPONSE	529	14 (2.6)	14 (2.6)	0 (0)	0.021	<0.0001	0.0122
GO REGULATION OF ENDOTHELIAL CELL DEVELOPMENT	12	3 (25)	3 (25)	0 (0)	0.021	<0.0001	0.0122
GO REGULATION OF ESTABLISHMENT OF ENDOTHELIAL BARR...	12	3 (25)	3 (25)	0 (0)	0.021	<0.0001	0.0122
GO REGULATION OF IMMUNE SYSTEM PROCESS	1369	30 (2.2)	27 (2)	3 (0.2)	0.020	<0.0001	0.0122
GO IMMUNE SYSTEM PROCESS	1932	41 (2.1)	39 (2)	2 (0.1)	0.020	<0.0001	0.0122
GO RESPONSE TO EXTERNAL STIMULUS	1798	34 (1.9)	31 (1.7)	3 (0.2)	0.018	<0.0001	0.0122
GO POSITIVE REGULATION OF RESPONSE TO STIMULUS	1898	28 (1.5)	25 (1.3)	3 (0.2)	0.014	<0.0001	0.0122
GO NEGATIVE REGULATION OF VIRAL PROCESS	91	6 (6.6)	6 (6.6)	0 (0)	0.027	0.0002	0.0202
GO POSITIVE REGULATION OF ACUTE INFLAMMATORY RESPO...	28	4 (14.3)	4 (14.3)	0 (0)	0.025	0.0002	0.0202
GO POSITIVE REGULATION OF INFLAMMATORY RESPONSE	112	6 (5.4)	5 (4.5)	1 (0.9)	0.025	0.0002	0.0202
GO POSITIVE REGULATION OF CHEMOTAXIS	120	6 (5)	5 (4.2)	1 (0.8)	0.024	0.0002	0.0202
GO POSITIVE REGULATION OF LEUKOCYTE CHEMOTAXIS	81	5 (6.2)	5 (6.2)	0 (0)	0.024	0.0002	0.0202
GO REGULATION OF INNATE IMMUNE RESPONSE	357	10 (2.8)	10 (2.8)	0 (0)	0.021	0.0002	0.0202
GO REGULATION OF CYTOKINE PRODUCTION	561	14 (2.5)	14 (2.5)	0 (0)	0.021	0.0002	0.0202
GO CELLULAR RESPONSE TO ORGANIC SUBSTANCE	1848	28 (1.5)	25 (1.4)	3 (0.2)	0.014	0.0002	0.0202
GO REGULATION OF CYTOKINE SECRETION	147	7 (4.8)	7 (4.8)	0 (0)	0.025	0.0003	0.0258
GO REGULATION OF VIRAL GENOME REPLICATION	75	5 (6.7)	5 (6.7)	0 (0)	0.024	0.0003	0.0258
GO PHAGOCYTOSIS	163	7 (4.3)	7 (4.3)	0 (0)	0.024	0.0003	0.0258
GO CELLULAR RESPONSE TO HEAT	36	4 (11.1)	4 (11.1)	0 (0)	0.024	0.0003	0.0258

Table 92: Enriched MSigDB Biological Processes (PBMC, Trivalent Influenza Vaccine, Day 1). Results are sorted by FDR adjusted p-value and Jaccard similarity index. Top 50 results are listed.

Gene Set Name	Gene Set Genes #	DE Genes N (%)	Up-reg. DE Genes N (%)	Down-reg. DE Genes N (%)	Jaccard Index	P-Value	FDR Adjusted P-Value
GO ENDOCYTIC VESICLE MEMBRANE	150	7 (4.7)	7 (4.7)	0 (0)	0.025	0.0002	0.0584
GO ENDOCYTIC VESICLE	255	9 (3.5)	9 (3.5)	0 (0)	0.024	0.0002	0.0584
GO EXTRACELLULAR SPACE	1339	22 (1.6)	19 (1.4)	3 (0.2)	0.015	0.0003	0.0584

Table 93: Enriched MSigDB Cellular Components (PBMC, Trivalent Influenza Vaccine, Day 1). Results are sorted by FDR adjusted p-value and Jaccard similarity index.

Gene Set Name	Gene Set Genes #	DE Genes N (%)	Up-reg. DE Genes N (%)	Down-reg. DE Genes N (%)	Jaccard Index	P-Value	FDR Adjusted P-Value
GSE13485 CTRL VS DAY7 YF17D VACCINE PBMC DN	202	33 (16.3)	33 (16.3)	0 (0)	0.109	<0.0001	0.002

Gene Set Name	Gene Set Genes #	DE Genes N (%)	Up-reg. DE Genes N (%)	Down-reg. DE Genes N (%)	Jaccard Index	P-Value	FDR Adjusted P-Value
GSE13485 DAY3 VS DAY7 YF17D VACCINE PBMC DN	199	27 (13.6)	27 (13.6)	0 (0)	0.088	<0.0001	0.002
GSE13485 PRE VS POST YF17D VACCINATION PBMC DN	199	27 (13.6)	27 (13.6)	0 (0)	0.088	<0.0001	0.002
GSE14000 UNSTIM VS 4H LPS DC DN	197	26 (13.2)	26 (13.2)	0 (0)	0.085	<0.0001	0.002
GSE13485 DAY1 VS DAY7 YF17D VACCINE PBMC DN	199	26 (13.1)	26 (13.1)	0 (0)	0.084	<0.0001	0.002
GSE42724 NAIVE BCELL VS PLASMABLAST UP	192	25 (13)	25 (13)	0 (0)	0.083	<0.0001	0.002
GSE13485 CTRL VS DAY3 YF17D VACCINE PBMC DN	198	25 (12.6)	25 (12.6)	0 (0)	0.081	<0.0001	0.002
GSE19888 ADENOSINE A3R INH PRETREAT AND ACT BY A3R...	202	25 (12.4)	25 (12.4)	0 (0)	0.080	<0.0001	0.002
GSE11057 PBMC VS MEM CD4 TCELL UP	193	24 (12.4)	23 (11.9)	1 (0.5)	0.079	<0.0001	0.002
GSE19888 ADENOSINE A3R INH VS ACT WITH INHIBITOR P...	200	24 (12)	24 (12)	0 (0)	0.077	<0.0001	0.002
GSE10325 LUPUS CD4 TCELL VS LUPUS MYELOID DN	202	24 (11.9)	24 (11.9)	0 (0)	0.077	<0.0001	0.002
GSE18791 CTRL VS NEWCASTLE VIRUS DC 8H DN	193	23 (11.9)	23 (11.9)	0 (0)	0.075	<0.0001	0.002
GSE40685 TREG VS FOXP3 KO TREG PRECURSOR DN	197	22 (11.2)	22 (11.2)	0 (0)	0.071	<0.0001	0.002
GSE10325 MYELOID VS LUPUS MYELOID DN	199	22 (11.1)	22 (11.1)	0 (0)	0.070	<0.0001	0.002
GSE40685 TREG VS FOXP3 KO TREG PRECURSOR UP	195	21 (10.8)	20 (10.3)	1 (0.5)	0.068	<0.0001	0.002
GSE18791 UNSTIM VS NEWCATSLE VIRUS DC 10H DN	198	21 (10.6)	21 (10.6)	0 (0)	0.067	<0.0001	0.002
GSE1432 CTRL VS IFNG 24H MICROGLIA DN	199	21 (10.6)	21 (10.6)	0 (0)	0.067	<0.0001	0.002
GSE24634 IL4 VS CTRL TREATED NAIVE CD4 TCELL DAY10...	200	21 (10.5)	21 (10.5)	0 (0)	0.067	<0.0001	0.002
GSE1432 CTRL VS IFNG 6H MICROGLIA DN	201	21 (10.4)	21 (10.4)	0 (0)	0.067	<0.0001	0.002
GSE14000 UNSTIM VS 4H LPS DC TRANSLATED RNA DN	198	20 (10.1)	20 (10.1)	0 (0)	0.064	<0.0001	0.002
GSE18791 UNSTIM VS NEWCATSLE VIRUS DC 6H DN	198	20 (10.1)	20 (10.1)	0 (0)	0.064	<0.0001	0.002
GSE22140 GERMFREE VS SPF ARTHRITIC MOUSE CD4 TCELL...	199	20 (10.1)	20 (10.1)	0 (0)	0.064	<0.0001	0.002
GSE2706 UNSTIM VS 8H R848 DC DN	194	19 (9.8)	17 (8.8)	2 (1)	0.061	<0.0001	0.002
GSE18791 CTRL VS NEWCASTLE VIRUS DC 6H DN	197	19 (9.6)	19 (9.6)	0 (0)	0.061	<0.0001	0.002
GSE22886 CTRL VS LPS 24H DC DN	200	19 (9.5)	18 (9)	1 (0.5)	0.060	<0.0001	0.002
GSE22140 GERMFREE VS SPF MOUSE CD4 TCELL UP	201	19 (9.5)	19 (9.5)	0 (0)	0.060	<0.0001	0.002
GSE34156 TLR1 TLR2 LIGAND VS NOD2 AND TLR1 TLR2 LI...	198	18 (9.1)	18 (9.1)	0 (0)	0.057	<0.0001	0.002
GSE22196 HEALTHY VS OBESE MOUSE SKIN GAMMADELTA TC...	199	18 (9)	18 (9)	0 (0)	0.057	<0.0001	0.002
GSE10325 LUPUS BCELL VS LUPUS MYELOID DN	200	18 (9)	18 (9)	0 (0)	0.057	<0.0001	0.002
GSE13484 UNSTIM VS YF17D VACCINE STIM PBMC DN	200	18 (9)	18 (9)	0 (0)	0.057	<0.0001	0.002
GSE42021 TREG VS TCONV PLN UP	200	18 (9)	18 (9)	0 (0)	0.057	<0.0001	0.002
GSE7509 UNSTIM VS IFNA STIM IMMATURE DC DN	171	16 (9.4)	15 (8.8)	1 (0.6)	0.055	<0.0001	0.002
GSE10325 BCELL VS MYELOID DN	198	17 (8.6)	17 (8.6)	0 (0)	0.054	<0.0001	0.002
GSE24634 IL4 VS CTRL TREATED NAIVE CD4 TCELL DAY3 ...	198	17 (8.6)	17 (8.6)	0 (0)	0.054	<0.0001	0.002
GSE18791 CTRL VS NEWCASTLE VIRUS DC 4H DN	183	16 (8.7)	16 (8.7)	0 (0)	0.053	<0.0001	0.002
GSE21546 WT VS SAP1A KO DP THYMOCYTES UP	194	16 (8.2)	16 (8.2)	0 (0)	0.051	<0.0001	0.002
GSE13485 DAY7 VS DAY21 YF17D VACCINE PBMC UP	195	16 (8.2)	16 (8.2)	0 (0)	0.051	<0.0001	0.002
GSE34156 UNTREATED VS 6H TLR1 TLR2 LIGAND TREATED ...	195	16 (8.2)	16 (8.2)	0 (0)	0.051	<0.0001	0.002
GSE18791 CTRL VS NEWCASTLE VIRUS DC 10H DN	198	16 (8.1)	16 (8.1)	0 (0)	0.051	<0.0001	0.002
GSE18281 CORTICAL VS MEDULLARY THYMOCYTE UP	199	16 (8)	16 (8)	0 (0)	0.050	<0.0001	0.002
GSE21360 PRIMARY VS TERTIARY MEMORY CD8 TCELL DN	199	16 (8)	16 (8)	0 (0)	0.050	<0.0001	0.002
GSE42021 CD24HI VS CD24INT TREG THYMUS DN	199	16 (8)	16 (8)	0 (0)	0.050	<0.0001	0.002
GSE18281 SUBCAPSULAR VS CENTRAL CORTICAL REGION OF...	200	16 (8)	16 (8)	0 (0)	0.050	<0.0001	0.002
GSE29618 MONOCYTE VS PDC UP	200	16 (8)	15 (7.5)	1 (0.5)	0.050	<0.0001	0.002
GSE8835 CD4 VS CD8 TCELL CLL PATIENT UP	200	16 (8)	16 (8)	0 (0)	0.050	<0.0001	0.002
GSE37533 PPARG1 FOXP3 VS PPARG2 FOXP3 TRANSDUCED C...	201	16 (8)	16 (8)	0 (0)	0.050	<0.0001	0.002
GSE1432 1H VS 6H IFNG MICROGLIA DN	202	16 (7.9)	16 (7.9)	0 (0)	0.050	<0.0001	0.002
GSE18791 UNSTIM VS NEWCATSLE VIRUS DC 18H DN	184	15 (8.2)	15 (8.2)	0 (0)	0.049	<0.0001	0.002

Gene Set Name	Gene Set Genes #	DE Genes N (%)	Up-reg. DE Genes N (%)	Down-reg. DE Genes N (%)	Jaccard Index	P-Value	FDR Adjusted P-Value
GSE18791 CTRL VS NEWCASTLE VIRUS DC 16H DN	188	15 (8)	15 (8)	0 (0)	0.049	<0.0001	0.002
GSE2706 UNSTIM VS 2H LPS AND R848 DC DN	190	15 (7.9)	15 (7.9)	0 (0)	0.048	<0.0001	0.002

Table 94: Enriched MSigDB Immunological Signatures (PBMC, Trivalent Influenza Vaccine, Day 1). Results are sorted by FDR adjusted p-value and Jaccard similarity index. Top 50 results are listed.

Gene Set Name	Gene Set Genes #	DE Genes N (%)	Up-reg. DE Genes N (%)	Down-reg. DE Genes N (%)	Jaccard Index	P-Value	FDR Adjusted P-Value
KEGG LEISHMANIA INFECTION	69	6 (8.7)	6 (8.7)	0 (0)	0.030	0.0002	0.0372

Table 95: Enriched MSigDB KEGG Pathways (PBMC, Trivalent Influenza Vaccine, Day 1). Results are sorted by FDR adjusted p-value and Jaccard similarity index.

Gene Set Name	Gene Set Genes #	DE Genes N (%)	Up-reg. DE Genes N (%)	Down-reg. DE Genes N (%)	Jaccard Index	P-Value	FDR Adjusted P-Value
GO IGG BINDING	10	3 (30)	3 (30)	0 (0)	0.021	<0.0001	0.0619
GO SUPEROXIDE GENERATING NADPH OXIDASE ACTIVITY	11	3 (27.3)	3 (27.3)	0 (0)	0.021	0.0002	0.0619
GO CYTOKINE RECEPTOR BINDING	271	8 (3)	7 (2.6)	1 (0.4)	0.020	0.0002	0.0619
GO OXIDOREDUCTASE ACTIVITY ACTING ON NAD P H OXYGE...	16	3 (18.8)	3 (18.8)	0 (0)	0.020	0.0003	0.0697
GO IMMUNOGLOBULIN BINDING	21	3 (14.3)	3 (14.3)	0 (0)	0.020	0.0004	0.0743
GO CYTOKINE ACTIVITY	218	7 (3.2)	6 (2.8)	1 (0.5)	0.020	0.0006	0.0929

Table 96: Enriched MSigDB Molecular Functions (PBMC, Trivalent Influenza Vaccine, Day 1). Results are sorted by FDR adjusted p-value and Jaccard similarity index.

Gene Set Name	Gene Set Genes #	DE Genes N (%)	Up-reg. DE Genes N (%)	Down-reg. DE Genes N (%)	Jaccard Index	P-Value	FDR Adjusted P-Value
REACTOME INTERFERON SIGNALING	153	12 (7.8)	12 (7.8)	0 (0)	0.043	<0.0001	0.0135
REACTOME CYTOKINE SIGNALING IN IMMUNE SYSTEM	265	15 (5.7)	15 (5.7)	0 (0)	0.039	<0.0001	0.0135
REACTOME INTERFERON GAMMA SIGNALING	59	7 (11.9)	7 (11.9)	0 (0)	0.037	<0.0001	0.0135
REACTOME INTERFERON ALPHA BETA SIGNALING	62	7 (11.3)	7 (11.3)	0 (0)	0.037	<0.0001	0.0135
REACTOME IMMUNE SYSTEM	897	23 (2.6)	21 (2.3)	2 (0.2)	0.023	<0.0001	0.0135
REACTOME G ALPHA I SIGNALLING EVENTS	194	8 (4.1)	8 (4.1)	0 (0)	0.025	0.0006	0.0674

Table 97: Enriched MSigDB Reactome Pathways (PBMC, Trivalent Influenza Vaccine, Day 1). Results are sorted by FDR adjusted p-value and Jaccard similarity index.

Gene Set Name	Gene Set Genes #	DE Genes N (%)	Up-reg. DE Genes N (%)	Down-reg. DE Genes N (%)	Jaccard Index	P-Value	FDR Adjusted P-Value
platelet activation - actin binding (M196)	16	5 (31.2)	5 (31.2)	0 (0)	0.053	<0.0001	0.0086
TBA (M104)	10	4 (40)	4 (40)	0 (0)	0.045	<0.0001	0.0086

Gene Set Name	Gene Set Genes #	DE Genes N (%)	Up-reg. DE Genes N (%)	Down-reg. DE Genes N (%)	Jaccard Index	P-Value	FDR Adjusted P-Value
platelet activation and blood coagulation (M199)	11	4 (36.4)	4 (36.4)	0 (0)	0.044	<0.0001	0.0086
chemokine cluster (I) (M27.0)	25	4 (16)	4 (16)	0 (0)	0.038	<0.0001	0.0086
enriched in myeloid cells and monocytes (M81)	35	5 (14.3)	5 (14.3)	0 (0)	0.044	0.0004	0.0231
G protein mediated calcium signaling (M159)	10	3 (30)	3 (30)	0 (0)	0.033	0.0004	0.0231
TBA (M193)	10	3 (30)	3 (30)	0 (0)	0.033	0.0006	0.0297
enriched in activated dendritic cells (II) (M165)	35	4 (11.4)	4 (11.4)	0 (0)	0.035	0.0017	0.0623
cytoskeleton/actin (SRF transcription targets) (M1...	14	3 (21.4)	3 (21.4)	0 (0)	0.032	0.0016	0.0623
TBA (M131)	14	3 (21.4)	3 (21.4)	0 (0)	0.032	0.0018	0.0623
cell adhesion (M51)	37	4 (10.8)	4 (10.8)	0 (0)	0.035	0.0027	0.0836
cytoskeleton/actin (SRF transcription targets) (M1...	16	3 (18.8)	3 (18.8)	0 (0)	0.031	0.0029	0.0836

Table 98: Enriched Blood Transcription Modules (PBMC, Trivalent Influenza Vaccine, Day 2). Results are sorted by FDR adjusted p-value and Jaccard similarity index.

Gene Set Name	Gene Set Genes #	DE Genes N (%)	Up-reg. DE Genes N (%)	Down-reg. DE Genes N (%)	Jaccard Index	P-Value	FDR Adjusted P-Value
GO PLATELET DEGRANULATION	107	7 (6.5)	7 (6.5)	0 (0)	0.038	<0.0001	0.0274
GO PLATELET ACTIVATION	142	7 (4.9)	7 (4.9)	0 (0)	0.032	<0.0001	0.0274
GO REGULATED EXOCYTOSIS	224	8 (3.6)	8 (3.6)	0 (0)	0.027	<0.0001	0.0274
GO HEMOSTASIS	312	10 (3.2)	10 (3.2)	0 (0)	0.026	<0.0001	0.0274
GO POSITIVE REGULATION OF LOCOMOTION	422	11 (2.6)	11 (2.6)	0 (0)	0.022	<0.0001	0.0274
GO WOUND HEALING	473	11 (2.3)	11 (2.3)	0 (0)	0.020	<0.0001	0.0274
GO RESPONSE TO WOUNDING	565	12 (2.1)	12 (2.1)	0 (0)	0.019	<0.0001	0.0274
GO IMMUNE EFFECTOR PROCESS	456	10 (2.2)	10 (2.2)	0 (0)	0.019	<0.0001	0.0274
GO DEFENSE RESPONSE TO OTHER ORGANISM	483	10 (2.1)	10 (2.1)	0 (0)	0.018	<0.0001	0.0274
GO RESPONSE TO BIOTIC STIMULUS	863	16 (1.9)	16 (1.9)	0 (0)	0.017	<0.0001	0.0274
GO DEFENSE RESPONSE	1197	21 (1.8)	21 (1.8)	0 (0)	0.017	<0.0001	0.0274
GO INNATE IMMUNE RESPONSE	589	11 (1.9)	11 (1.9)	0 (0)	0.017	<0.0001	0.0274
GO IMMUNE RESPONSE	1052	16 (1.5)	16 (1.5)	0 (0)	0.014	<0.0001	0.0274
GO IMMUNE SYSTEM PROCESS	1932	24 (1.2)	24 (1.2)	0 (0)	0.012	<0.0001	0.0274
GO REGULATION OF IMMUNE SYSTEM PROCESS	1369	17 (1.2)	17 (1.2)	0 (0)	0.012	<0.0001	0.0274
GO CELLULAR RESPONSE TO ORGANIC SUBSTANCE	1848	21 (1.1)	21 (1.1)	0 (0)	0.011	<0.0001	0.0274
GO RESPONSE TO EXTERNAL STIMULUS	1798	20 (1.1)	20 (1.1)	0 (0)	0.011	<0.0001	0.0274
GO RESPONSE TO INTERFERON ALPHA	20	3 (15)	3 (15)	0 (0)	0.030	0.0002	0.0405
GO POSITIVE REGULATION OF LEUKOCYTE MIGRATION	109	5 (4.6)	5 (4.6)	0 (0)	0.027	0.0002	0.0405
GO CHEMOKINE MEDIATED SIGNALING PATHWAY	71	4 (5.6)	4 (5.6)	0 (0)	0.027	0.0002	0.0405
GO REGULATION OF BODY FLUID LEVELS	508	10 (2)	10 (2)	0 (0)	0.017	0.0002	0.0405
GO CYTOKINE MEDIATED SIGNALING PATHWAY	448	9 (2)	9 (2)	0 (0)	0.017	0.0002	0.0405
GO RESPONSE TO CYTOKINE	710	12 (1.7)	12 (1.7)	0 (0)	0.015	0.0002	0.0405
GO INTEGRIN MEDIATED SIGNALING PATHWAY	82	4 (4.9)	4 (4.9)	0 (0)	0.025	0.0003	0.0465
GO REGULATION OF LEUKOCYTE MIGRATION	148	5 (3.4)	5 (3.4)	0 (0)	0.022	0.0003	0.0465
GO LEUKOCYTE MEDIATED IMMUNITY	158	5 (3.2)	5 (3.2)	0 (0)	0.021	0.0003	0.0465
GO RESPONSE TO MOLECULE OF BACTERIAL ORIGIN	320	7 (2.2)	7 (2.2)	0 (0)	0.018	0.0003	0.0465
GO CELL ACTIVATION	567	11 (1.9)	11 (1.9)	0 (0)	0.017	0.0003	0.0465
GO SECRETION BY CELL	488	9 (1.8)	9 (1.8)	0 (0)	0.016	0.0003	0.0465
GO SECRETION	590	10 (1.7)	10 (1.7)	0 (0)	0.015	0.0003	0.0465

Gene Set Name	Gene Set Genes #	DE Genes N (%)	Up-reg. DE Genes N (%)	Down-reg. DE Genes N (%)	Jaccard Index	P-Value	FDR Adjusted P-Value
GO HUMORAL IMMUNE RESPONSE MEDIATED BY CIRCULATING...	38	3 (7.9)	3 (7.9)	0 (0)	0.025	0.0004	0.0547
GO PLATELET AGGREGATION	39	3 (7.7)	3 (7.7)	0 (0)	0.025	0.0004	0.0547
GO POSITIVE REGULATION OF LEUKOCYTE CHEMOTAXIS	81	4 (4.9)	4 (4.9)	0 (0)	0.025	0.0004	0.0547
GO EXOCYTOSIS	310	8 (2.6)	8 (2.6)	0 (0)	0.021	0.0004	0.0547
GO REGULATION OF BONE RESORPTION	33	3 (9.1)	3 (9.1)	0 (0)	0.026	0.0005	0.0665
GO REGULATION OF LEUKOCYTE CHEMOTAXIS	95	4 (4.2)	4 (4.2)	0 (0)	0.023	0.0006	0.0775
GO POSITIVE REGULATION OF ENDOTHELIAL CELL MIGRATI...	67	4 (6)	4 (6)	0 (0)	0.027	0.0007	0.0835
GO RESPONSE TO BACTERIUM	502	8 (1.6)	8 (1.6)	0 (0)	0.014	0.0007	0.0835
GO REGULATION OF CELLULAR COMPONENT MOVEMENT	775	11 (1.4)	11 (1.4)	0 (0)	0.013	0.0007	0.0835
GO RESPONSE TO METAL ION	338	7 (2.1)	7 (2.1)	0 (0)	0.017	0.0008	0.0931

Table 99: Enriched MSigDB Biological Processes (PBMC, Trivalent Influenza Vaccine, Day 2). Results are sorted by FDR adjusted p-value and Jaccard similarity index.

Gene Set Name	Gene Set Genes #	DE Genes N (%)	Up-reg. DE Genes N (%)	Down-reg. DE Genes N (%)	Jaccard Index	P-Value	FDR Adjusted P-Value
GO PLATELET ALPHA GRANULE	75	8 (10.7)	8 (10.7)	0 (0)	0.053	<0.0001	0.0117
GO PLATELET ALPHA GRANULE LUMEN	55	5 (9.1)	5 (9.1)	0 (0)	0.038	<0.0001	0.0117
GO SECRETORY GRANULE LUMEN	85	5 (5.9)	5 (5.9)	0 (0)	0.031	<0.0001	0.0117
GO SECRETORY GRANULE	353	10 (2.8)	10 (2.8)	0 (0)	0.024	<0.0001	0.0117
GO SECRETORY VESICLE	463	11 (2.4)	11 (2.4)	0 (0)	0.021	<0.0001	0.0117
GO ACTIN FILAMENT BUNDLE	56	4 (7.1)	4 (7.1)	0 (0)	0.030	0.0002	0.0167
GO VESICLE LUMEN	106	5 (4.7)	5 (4.7)	0 (0)	0.027	0.0002	0.0167
GO PLATELET ALPHA GRANULE MEMBRANE	13	3 (23.1)	3 (23.1)	0 (0)	0.032	0.0004	0.0292
GO PROTEIN COMPLEX INVOLVED IN CELL ADHESION	30	3 (10)	3 (10)	0 (0)	0.027	0.0006	0.035
GO EXTRACELLULAR SPACE	1339	16 (1.2)	16 (1.2)	0 (0)	0.011	0.0006	0.035
GO ACTOMYOSIN	62	4 (6.5)	4 (6.5)	0 (0)	0.028	0.0007	0.0372
GO DNA PACKAGING COMPLEX	105	4 (3.8)	4 (3.8)	0 (0)	0.022	0.001	0.0487
GO CYTOPLASMIC VESICLE PART	600	10 (1.7)	10 (1.7)	0 (0)	0.015	0.0012	0.0539
GO CELL SURFACE	729	11 (1.5)	11 (1.5)	0 (0)	0.014	0.0015	0.0626
GO INTRACELLULAR VESICLE	1258	15 (1.2)	15 (1.2)	0 (0)	0.011	0.002	0.0779
GO BLOOD MICROPARTICLE	118	4 (3.4)	4 (3.4)	0 (0)	0.020	0.0025	0.0912

Table 100: Enriched MSigDB Cellular Components (PBMC, Trivalent Influenza Vaccine, Day 2). Results are sorted by FDR adjusted p-value and Jaccard similarity index.

Gene Set Name	Gene Set Genes #	DE Genes N (%)	Up-reg. DE Genes N (%)	Down-reg. DE Genes N (%)	Jaccard Index	P-Value	FDR Adjusted P-Value
GSE9006 HEALTHY VS TYPE 1 DIABETES PBMC AT DX UP	197	16 (8.1)	16 (8.1)	0 (0)	0.061	<0.0001	0.009
GSE11057 PBMC VS MEM CD4 TCELL UP	193	13 (6.7)	13 (6.7)	0 (0)	0.049	<0.0001	0.009
GSE13485 DAY3 VS DAY7 YF17D VACCINE PBMC DN	199	12 (6)	12 (6)	0 (0)	0.044	<0.0001	0.009
GSE13485 CTRL VS DAY7 YF17D VACCINE PBMC DN	202	11 (5.4)	11 (5.4)	0 (0)	0.040	<0.0001	0.009
GSE45365 WT VS IFNAR KO BCELL MCMV INFECTION DN	188	10 (5.3)	10 (5.3)	0 (0)	0.038	<0.0001	0.009
GSE45365 HEALTHY VS MCMV INFECTION CD11B DC DN	190	10 (5.3)	10 (5.3)	0 (0)	0.038	<0.0001	0.009

Gene Set Name	Gene Set Genes #	DE Genes N (%)	Up-reg. DE Genes N (%)	Down-reg. DE Genes N (%)	Jaccard Index	P-Value	FDR Adjusted P-Value
GSE9509 LPS VS LPS AND IL10 STIM IL10 KO MACROPHAG...	190	10 (5.3)	10 (5.3)	0 (0)	0.038	<0.0001	0.009
GSE18791 CTRL VS NEWCASTLE VIRUS DC 8H DN	193	9 (4.7)	9 (4.7)	0 (0)	0.034	<0.0001	0.009
GSE13485 DAY7 VS DAY21 YF17D VACCINE PBMC UP	195	9 (4.6)	9 (4.6)	0 (0)	0.034	<0.0001	0.009
GSE13485 CTRL VS DAY3 YF17D VACCINE PBMC DN	198	9 (4.5)	9 (4.5)	0 (0)	0.033	<0.0001	0.009
GSE22886 CTRL VS LPS 24H DC DN	200	9 (4.5)	9 (4.5)	0 (0)	0.033	<0.0001	0.009
GSE7400 CTRL VS CSF3 IN VIVO TREATED PBMC UP	200	9 (4.5)	9 (4.5)	0 (0)	0.033	<0.0001	0.009
GSE29617 CTRL VS DAY7 TIV FLU VACCINE PBMC 2008 UP	189	8 (4.2)	8 (4.2)	0 (0)	0.030	<0.0001	0.009
GSE40685 TREG VS FOXP3 KO TREG PRECURSOR UP	195	8 (4.1)	8 (4.1)	0 (0)	0.030	<0.0001	0.009
GSE18791 UNSTIM VS NEWCATSLE VIRUS DC 10H DN	198	8 (4)	8 (4)	0 (0)	0.029	<0.0001	0.009
GSE10325 MYELOID VS LUPUS MYELOID DN	199	8 (4)	8 (4)	0 (0)	0.029	<0.0001	0.009
GSE13485 PRE VS POST YF17D VACCINATION PBMC DN	199	8 (4)	8 (4)	0 (0)	0.029	<0.0001	0.009
GSE37534 UNTREATED VS PIOGLITAZONE TREATED CD4 TCE...	199	8 (4)	8 (4)	0 (0)	0.029	<0.0001	0.009
GSE360 HIGH DOSE B MALAYI VS M TUBERCULOSIS DC DN	200	8 (4)	8 (4)	0 (0)	0.029	<0.0001	0.009
GSE22140 GERMFREE VS SPF MOUSE CD4 TCELL UP	201	8 (4)	8 (4)	0 (0)	0.029	<0.0001	0.009
GSE5589 LPS VS LPS AND IL10 STIM IL10 KO MACROPHAG...	202	8 (4)	8 (4)	0 (0)	0.029	<0.0001	0.009
GSE18791 UNSTIM VS NEWCATSLE VIRUS DC 18H DN	184	7 (3.8)	7 (3.8)	0 (0)	0.027	<0.0001	0.009
GSE29615 CTRL VS DAY7 LAIV FLU VACCINE PBMC UP	184	7 (3.8)	7 (3.8)	0 (0)	0.027	<0.0001	0.009
GSE42724 NAIVE BCELL VS PLASMABLAST UP	192	7 (3.6)	7 (3.6)	0 (0)	0.026	<0.0001	0.009
GSE21546 WT VS SAP1A KO DP THYMOCYTES UP	194	7 (3.6)	7 (3.6)	0 (0)	0.026	<0.0001	0.009
GSE13485 DAY1 VS DAY7 YF17D VACCINE PBMC DN	199	7 (3.5)	7 (3.5)	0 (0)	0.025	<0.0001	0.009
GSE18281 CORTICAL VS MEDULLARY THYMOCYTE UP	199	7 (3.5)	7 (3.5)	0 (0)	0.025	<0.0001	0.009
GSE21360 PRIMARY VS TERTIARY MEMORY CD8 TCELL DN	199	7 (3.5)	7 (3.5)	0 (0)	0.025	<0.0001	0.009
GSE22140 HEALTHY VS ARTHRITIC MOUSE CD4 TCELL UP	198	7 (3.5)	7 (3.5)	0 (0)	0.025	<0.0001	0.009
GSE22196 HEALTHY VS OBESE MOUSE SKIN GAMMADELTA TC...	199	7 (3.5)	7 (3.5)	0 (0)	0.025	<0.0001	0.009
GSE24634 IL4 VS CTRL TREATED NAIVE CD4 TCELL DAY3 ...	198	7 (3.5)	7 (3.5)	0 (0)	0.025	<0.0001	0.009
GSE360 LOW DOSE B MALAYI VS M TUBERCULOSIS DC DN	199	7 (3.5)	7 (3.5)	0 (0)	0.025	<0.0001	0.009
GSE3982 CTRL VS LPS 48H DC DN	198	7 (3.5)	7 (3.5)	0 (0)	0.025	<0.0001	0.009
GSE41978 ID2 KO VS BIM KO KLRG1 LOW EFFECTOR CD8 T...	199	7 (3.5)	7 (3.5)	0 (0)	0.025	<0.0001	0.009
GSE43955 TH0 VS TGFB IL6 TH17 ACT CD4 TCELL 1H UP	199	7 (3.5)	7 (3.5)	0 (0)	0.025	<0.0001	0.009
GSE19888 ADENOSINE A3R INH VS ACT WITH INHIBITOR P...	200	7 (3.5)	7 (3.5)	0 (0)	0.025	<0.0001	0.009
GSE21670 STAT3 KO VS WT CD4 TCELL IL6 TREATED DN	200	7 (3.5)	7 (3.5)	0 (0)	0.025	<0.0001	0.009
GSE34205 HEALTHY VS FLU INF INFANT PBMC DN	200	7 (3.5)	7 (3.5)	0 (0)	0.025	<0.0001	0.009
GSE36392 TYPE 2 MYELOID VS NEUTROPHIL IL25 TREATED...	200	7 (3.5)	7 (3.5)	0 (0)	0.025	<0.0001	0.009
GSE42021 TREG VS TCONV PLN UP	200	7 (3.5)	7 (3.5)	0 (0)	0.025	<0.0001	0.009
GSE39556 UNTREATED VS 3H POLYIC INJ MOUSE NK CELL ...	202	7 (3.5)	7 (3.5)	0 (0)	0.025	<0.0001	0.009
GSE37605 TREG VS TCONV NOD FOXP3 FUSION GFP DN	165	6 (3.6)	6 (3.6)	0 (0)	0.025	<0.0001	0.009
GSE7509 UNSTIM VS IFNA STIM IMMATURE DC DN	171	6 (3.5)	6 (3.5)	0 (0)	0.024	<0.0001	0.009
GSE37605 FOXP3 FUSION GFP VS IRES GFP TREG C57BL6 ...	172	6 (3.5)	6 (3.5)	0 (0)	0.024	<0.0001	0.009
GSE21546 ELK1 KO VS SAP1A KO AND ELK1 KO DP THYMOC...	191	6 (3.1)	6 (3.1)	0 (0)	0.022	<0.0001	0.009
GSE40685 TREG VS FOXP3 KO TREG PRECURSOR DN	197	6 (3)	6 (3)	0 (0)	0.022	<0.0001	0.009
GSE36888 STAT5 AB KNOCKIN VS WT TCELL IL2 TREATED ...	198	6 (3)	6 (3)	0 (0)	0.022	<0.0001	0.009
GSE14000 UNSTIM VS 16H LPS DC DN	200	6 (3)	6 (3)	0 (0)	0.022	<0.0001	0.009
GSE5503 LIVER DC VS MLN DC ACTIVATED ALLOGENIC TCE...	200	6 (3)	6 (3)	0 (0)	0.022	<0.0001	0.009
GSE7219 WT VS NIK NFKB2 KO LPS AND ANTI CD40 STIM ...	200	6 (3)	6 (3)	0 (0)	0.022	<0.0001	0.009

Table 101: Enriched MSigDB Immunological Signatures (PBMC, Trivalent Influenza Vaccine, Day 2). Results are sorted by FDR adjusted p-value and Jaccard similarity index. Top 50 results are listed.

Gene Set Name	Gene Set Genes #	DE Genes N (%)	Up-reg. DE Genes N (%)	Down-reg. DE Genes N (%)	Jaccard Index	P-Value	FDR Adjusted P-Value
KEGG SYSTEMIC LUPUS ERYTHEMATOSUS	133	6 (4.5)	6 (4.5)	0 (0)	0.029	<0.0001	0.0186

Table 102: Enriched MSigDB KEGG Pathways (PBMC, Trivalent Influenza Vaccine, Day 2). Results are sorted by FDR adjusted p-value and Jaccard similarity index.

Gene Set Name	Gene Set Genes #	DE Genes N (%)	Up-reg. DE Genes N (%)	Down-reg. DE Genes N (%)	Jaccard Index	P-Value	FDR Adjusted P-Value
GO CXCR CHEMOKINE RECEPTOR BINDING	17	4 (23.5)	4 (23.5)	0 (0)	0.042	<0.0001	0.0464
GO CHEMOKINE ACTIVITY	47	4 (8.5)	4 (8.5)	0 (0)	0.032	<0.0001	0.0464
GO CHEMOKINE RECEPTOR BINDING	57	4 (7)	4 (7)	0 (0)	0.029	0.0002	0.0619
GO CYTOKINE ACTIVITY	218	6 (2.8)	6 (2.8)	0 (0)	0.020	0.0003	0.0697

Table 103: Enriched MSigDB Molecular Functions (PBMC, Trivalent Influenza Vaccine, Day 2). Results are sorted by FDR adjusted p-value and Jaccard similarity index.

Gene Set Name	Gene Set Genes #	DE Genes N (%)	Up-reg. DE Genes N (%)	Down-reg. DE Genes N (%)	Jaccard Index	P-Value	FDR Adjusted P-Value
REACTOME RESPONSE TO ELEVATED PLATELET CYTOSOLIC C...	81	7 (8.6)	7 (8.6)	0 (0)	0.045	<0.0001	0.0168
REACTOME SMOOTH MUSCLE CONTRACTION	23	4 (17.4)	4 (17.4)	0 (0)	0.039	<0.0001	0.0168
REACTOME PLATELET ACTIVATION SIGNALING AND AGGREGA...	200	10 (5)	10 (5)	0 (0)	0.037	<0.0001	0.0168
REACTOME HEMOSTASIS	452	13 (2.9)	13 (2.9)	0 (0)	0.025	<0.0001	0.0168
REACTOME MUSCLE CONTRACTION	46	4 (8.7)	4 (8.7)	0 (0)	0.032	0.0006	0.0674
REACTOME CHEMOKINE RECEPTORS BIND CHEMOKINES	54	4 (7.4)	4 (7.4)	0 (0)	0.030	0.0005	0.0674

Table 104: Enriched MSigDB Reactome Pathways (PBMC, Trivalent Influenza Vaccine, Day 2). Results are sorted by FDR adjusted p-value and Jaccard similarity index.

Gene Set Name	Gene Set Genes #	DE Genes N (%)	Up-reg. DE Genes N (%)	Down-reg. DE Genes N (%)	Jaccard Index	P-Value	FDR Adjusted P-Value
proinflammatory cytokines and chemokines (M29)	8	2 (25)	2 (25)	0 (0)	0.080	0.0003	0.0461
antiviral IFN signature (M75)	22	3 (13.6)	3 (13.6)	0 (0)	0.079	0.0004	0.0461
enriched in activated dendritic cells (II) (M165)	35	3 (8.6)	3 (8.6)	0 (0)	0.059	0.0003	0.0461
enriched in neutrophils (I) (M37.1)	47	3 (6.4)	3 (6.4)	0 (0)	0.048	0.0009	0.0778

Table 105: Enriched Blood Transcription Modules (PBMC, Trivalent Influenza Vaccine, Day 3). Results are sorted by FDR adjusted p-value and Jaccard similarity index.

Gene Set Name	Gene Set Genes #	DE Genes N (%)	Up-reg. DE Genes N (%)	Down-reg. DE Genes N (%)	Jaccard Index	P-Value	FDR Adjusted P-Value
GO REGULATION OF CHEMOKINE BIOSYNTHETIC PROCESS	12	3 (25)	3 (25)	0 (0)	0.107	<0.0001	0.0108
GO MODULATION OF GROWTH OF SYMBIONT INVOLVED IN IN...	16	3 (18.8)	3 (18.8)	0 (0)	0.094	<0.0001	0.0108
GO POSITIVE REGULATION OF INTERLEUKIN 8 PRODUCTION	45	5 (11.1)	5 (11.1)	0 (0)	0.085	<0.0001	0.0108

Gene Set Name	Gene Set Genes #	DE Genes N (%)	Up-reg. DE Genes N (%)	Down-reg. DE Genes N (%)	Jaccard Index	P-Value	FDR Adjusted P-Value
GO NEGATIVE REGULATION OF LIPID CATABOLIC PROCESS	21	3 (14.3)	3 (14.3)	0 (0)	0.081	<0.0001	0.0108
GO INFLAMMATORY RESPONSE TO ANTIGENIC STIMULUS	25	3 (12)	3 (12)	0 (0)	0.073	<0.0001	0.0108
GO REGULATION OF FEVER GENERATION	11	2 (18.2)	2 (18.2)	0 (0)	0.071	<0.0001	0.0108
GO REGULATION OF INTERLEUKIN 8 BIOSYNTHETIC PROCES...	12	2 (16.7)	2 (16.7)	0 (0)	0.069	<0.0001	0.0108
GO POSITIVE REGULATION OF ACUTE INFLAMMATORY RESPO...	28	3 (10.7)	3 (10.7)	0 (0)	0.068	<0.0001	0.0108
GO REGULATION OF INTERLEUKIN 8 PRODUCTION	61	5 (8.2)	5 (8.2)	0 (0)	0.067	<0.0001	0.0108
GO NEGATIVE REGULATION OF EXTRINSIC APOPTOTIC SIGN...	32	3 (9.4)	3 (9.4)	0 (0)	0.062	<0.0001	0.0108
GO NEGATIVE REGULATION OF SIGNAL TRANSDUCTION IN A...	32	3 (9.4)	3 (9.4)	0 (0)	0.062	<0.0001	0.0108
GO CHRONIC INFLAMMATORY RESPONSE	15	2 (13.3)	2 (13.3)	0 (0)	0.062	<0.0001	0.0108
GO REGULATION OF HEAT GENERATION	15	2 (13.3)	2 (13.3)	0 (0)	0.062	<0.0001	0.0108
GO NEGATIVE REGULATION OF GLUCOSE TRANSPORT	17	2 (11.8)	2 (11.8)	0 (0)	0.059	<0.0001	0.0108
GO REGULATION OF CHEMOKINE PRODUCTION	65	4 (6.2)	4 (6.2)	0 (0)	0.050	<0.0001	0.0108
GO POSITIVE REGULATION OF CHEMOKINE PRODUCTION	49	3 (6.1)	3 (6.1)	0 (0)	0.046	<0.0001	0.0108
GO REGULATION OF ACUTE INFLAMMATORY RESPONSE	74	4 (5.4)	4 (5.4)	0 (0)	0.045	<0.0001	0.0108
GO REGULATION OF LIPID CATABOLIC PROCESS	52	3 (5.8)	3 (5.8)	0 (0)	0.044	<0.0001	0.0108
GO NEGATIVE REGULATION OF EXTRINSIC APOPTOTIC SIGN...	98	4 (4.1)	4 (4.1)	0 (0)	0.035	<0.0001	0.0108
GO NEGATIVE REGULATION OF LIPID METABOLIC PROCESS	80	3 (3.8)	3 (3.8)	0 (0)	0.031	<0.0001	0.0108
GO POSITIVE REGULATION OF CYTOKINE SECRETION	96	3 (3.1)	3 (3.1)	0 (0)	0.027	<0.0001	0.0108
GO REGULATION OF EXTRINSIC APOPTOTIC SIGNALING PAT...	153	4 (2.6)	4 (2.6)	0 (0)	0.024	<0.0001	0.0108
GO CELLULAR RESPONSE TO ACID CHEMICAL	175	4 (2.3)	4 (2.3)	0 (0)	0.021	<0.0001	0.0108
GO NEGATIVE REGULATION OF APOPTOTIC SIGNALING PATH...	200	4 (2)	4 (2)	0 (0)	0.019	<0.0001	0.0108
GO RESPONSE TO MOLECULE OF BACTERIAL ORIGIN	320	6 (1.9)	6 (1.9)	0 (0)	0.018	<0.0001	0.0108
GO REGULATION OF INFLAMMATORY RESPONSE	291	5 (1.7)	5 (1.7)	0 (0)	0.016	<0.0001	0.0108
GO POSITIVE REGULATION OF CYTOKINE PRODUCTION	369	5 (1.4)	5 (1.4)	0 (0)	0.013	<0.0001	0.0108
GO INFLAMMATORY RESPONSE	451	6 (1.3)	6 (1.3)	0 (0)	0.013	<0.0001	0.0108
GO RESPONSE TO BACTERIUM	502	6 (1.2)	6 (1.2)	0 (0)	0.012	<0.0001	0.0108
GO REGULATION OF RESPONSE TO WOUNDING	412	5 (1.2)	5 (1.2)	0 (0)	0.012	<0.0001	0.0108
GO REGULATION OF IMMUNE EFFECTOR PROCESS	426	5 (1.2)	5 (1.2)	0 (0)	0.011	<0.0001	0.0108
GO POSITIVE REGULATION OF IMMUNE SYSTEM PROCESS	833	8 (1)	8 (1)	0 (0)	0.009	<0.0001	0.0108
GO RESPONSE TO BIOTIC STIMULUS	863	8 (0.9)	8 (0.9)	0 (0)	0.009	<0.0001	0.0108
GO REGULATION OF DEFENSE RESPONSE	757	7 (0.9)	7 (0.9)	0 (0)	0.009	<0.0001	0.0108
GO RESPONSE TO LIPID	887	8 (0.9)	8 (0.9)	0 (0)	0.009	<0.0001	0.0108
GO REGULATION OF CYTOKINE PRODUCTION	561	5 (0.9)	5 (0.9)	0 (0)	0.009	<0.0001	0.0108
GO DEFENSE RESPONSE	1197	10 (0.8)	10 (0.8)	0 (0)	0.008	<0.0001	0.0108
GO REGULATION OF IMMUNE SYSTEM PROCESS	1369	9 (0.7)	9 (0.7)	0 (0)	0.006	<0.0001	0.0108
GO REGULATION OF CELL DEATH	1472	9 (0.6)	9 (0.6)	0 (0)	0.006	<0.0001	0.0108
GO RESPONSE TO OXYGEN CONTAINING COMPOUND	1385	8 (0.6)	8 (0.6)	0 (0)	0.006	<0.0001	0.0108
GO RESPONSE TO EXTERNAL STIMULUS	1798	10 (0.6)	10 (0.6)	0 (0)	0.005	<0.0001	0.0108
GO REGULATION OF RESPONSE TO STRESS	1468	8 (0.5)	8 (0.5)	0 (0)	0.005	<0.0001	0.0108
GO IMMUNE SYSTEM PROCESS	1932	9 (0.5)	9 (0.5)	0 (0)	0.005	<0.0001	0.0108
GO LIPID STORAGE	27	3 (11.1)	3 (11.1)	0 (0)	0.070	0.0002	0.0155
GO REGULATION OF ENDOTHELIAL CELL DEVELOPMENT	12	2 (16.7)	2 (16.7)	0 (0)	0.069	0.0002	0.0155
GO REGULATION OF ESTABLISHMENT OF ENDOTHELIAL BARR...	12	2 (16.7)	2 (16.7)	0 (0)	0.069	0.0002	0.0155
GO REGULATION OF VITAMIN METABOLIC PROCESS	12	2 (16.7)	2 (16.7)	0 (0)	0.069	0.0002	0.0155
GO REGULATION OF HETEROTYPIC CELL CELL ADHESION	18	2 (11.1)	2 (11.1)	0 (0)	0.057	0.0002	0.0155
GO REGULATION OF EXTRINSIC APOPTOTIC SIGNALING PAT...	46	3 (6.5)	3 (6.5)	0 (0)	0.048	0.0002	0.0155

Gene Set Name	Gene Set Genes #	DE Genes N (%)	Up-reg. DE Genes N (%)	Down-reg. DE Genes N (%)	Jaccard Index	P-Value	FDR Adjusted P-Value
GO POSITIVE REGULATION OF CYTOKINE BIOSYNTHETIC PR...	58	3 (5.2)	3 (5.2)	0 (0)	0.041	0.0002	0.0155

Table 106: Enriched MSigDB Biological Processes (PBMC, Trivalent Influenza Vaccine, Day 3). Results are sorted by FDR adjusted p-value and Jaccard similarity index. Top 50 results are listed.

Gene Set Name	Gene Set Genes #	DE Genes N (%)	Up-reg. DE Genes N (%)	Down-reg. DE Genes N (%)	Jaccard Index	P-Value	FDR Adjusted P-Value
GO SECRETORY GRANULE	353	5 (1.4)	5 (1.4)	0 (0)	0.014	<0.0001	0.0195
GO SECRETORY VESICLE	463	5 (1.1)	5 (1.1)	0 (0)	0.011	<0.0001	0.0195
GO EXTRACELLULAR SPACE	1339	8 (0.6)	8 (0.6)	0 (0)	0.006	<0.0001	0.0195
GO BLOOD MICROPARTICLE	118	3 (2.5)	3 (2.5)	0 (0)	0.022	0.0002	0.0292

Table 107: Enriched MSigDB Cellular Components (PBMC, Trivalent Influenza Vaccine, Day 3). Results are sorted by FDR adjusted p-value and Jaccard similarity index.

Gene Set Name	Gene Set Genes #	DE Genes N (%)	Up-reg. DE Genes N (%)	Down-reg. DE Genes N (%)	Jaccard Index	P-Value	FDR Adjusted P-Value
GSE9006 HEALTHY VS TYPE 1 DIABETES PBMC AT DX DN	191	5 (2.6)	5 (2.6)	0 (0)	0.024	<0.0001	0.0375
GSE18791 UNSTIM VS NEWCATSLE VIRUS DC 10H DN	198	5 (2.5)	5 (2.5)	0 (0)	0.024	<0.0001	0.0375
GSE13485 DAY3 VS DAY7 YF17D VACCINE PBMC DN	199	5 (2.5)	5 (2.5)	0 (0)	0.024	<0.0001	0.0375
GSE29615 CTRL VS LAIV FLU VACCINE PBMC UP	189	4 (2.1)	4 (2.1)	0 (0)	0.020	<0.0001	0.0375
GSE9988 LPS VS CTRL TREATED MONOCYTE UP	189	4 (2.1)	4 (2.1)	0 (0)	0.020	<0.0001	0.0375
GSE18791 CTRL VS NEWCASTLE VIRUS DC 8H DN	193	4 (2.1)	4 (2.1)	0 (0)	0.019	<0.0001	0.0375
GSE18791 CTRL VS NEWCASTLE VIRUS DC 12H DN	194	4 (2.1)	4 (2.1)	0 (0)	0.019	<0.0001	0.0375
GSE22886 NAIVE CD8 TCELL VS NEUTROPHIL DN	194	4 (2.1)	4 (2.1)	0 (0)	0.019	<0.0001	0.0375
GSE40685 TREG VS FOXP3 KO TREG PRECURSOR DN	197	4 (2)	4 (2)	0 (0)	0.019	<0.0001	0.0375
GSE9988 ANTI TREM1 VS LPS MONOCYTE DN	197	4 (2)	4 (2)	0 (0)	0.019	<0.0001	0.0375
GSE10325 MYELOID VS LUPUS MYELOID DN	199	4 (2)	4 (2)	0 (0)	0.019	<0.0001	0.0375
GSE13484 UNSTIM VS YF17D VACCINE STIM PBMC DN	200	4 (2)	4 (2)	0 (0)	0.019	<0.0001	0.0375
GSE43955 10H VS 30H ACT CD4 TCELL UP	202	4 (2)	4 (2)	0 (0)	0.018	<0.0001	0.0375
GSE18791 CTRL VS NEWCASTLE VIRUS DC 16H DN	188	4 (2.1)	4 (2.1)	0 (0)	0.020	0.0002	0.065
GSE9988 ANTI TREM1 VS LOW LPS MONOCYTE DN	192	4 (2.1)	4 (2.1)	0 (0)	0.019	0.0002	0.065
GSE9988 ANTI TREM1 VS ANTI TREM1 AND LPS MONOCYTE ...	186	3 (1.6)	3 (1.6)	0 (0)	0.015	0.0005	0.0758
GSE2706 R848 VS R848 AND LPS 2H STIM DC DN	188	3 (1.6)	3 (1.6)	0 (0)	0.015	0.0006	0.0758
GSE2706 UNSTIM VS 2H R848 DC DN	190	3 (1.6)	3 (1.6)	0 (0)	0.015	0.0004	0.0758
GSE30971 CTRL VS LPS STIM MACROPHAGE WBP7 HET 2H U...	190	3 (1.6)	3 (1.6)	0 (0)	0.015	0.0007	0.0758
GSE9988 LPS VS VEHICLE TREATED MONOCYTE UP	189	3 (1.6)	3 (1.6)	0 (0)	0.015	0.0007	0.0758
GSE9960 HEALTHY VS GRAM POS SEPSIS PBMC DN	193	3 (1.6)	3 (1.6)	0 (0)	0.014	0.0006	0.0758
GSE17974 IL4 AND ANTI IL12 VS UNTREATED 6H ACT CD4...	193	3 (1.6)	3 (1.6)	0 (0)	0.014	0.0007	0.0758
GSE22886 NAIVE BCELL VS NEUTROPHIL DN	197	3 (1.5)	3 (1.5)	0 (0)	0.014	0.0004	0.0758
GSE22886 NAIVE TCELL VS NEUTROPHIL DN	197	3 (1.5)	3 (1.5)	0 (0)	0.014	0.0004	0.0758
GSE1432 CTRL VS IFNG 1H MICROGLIA DN	197	3 (1.5)	3 (1.5)	0 (0)	0.014	0.0007	0.0758
GSE36392 TYPE 2 MYELOID VS EOSINOPHIL IL25 TREATED...	199	3 (1.5)	3 (1.5)	0 (0)	0.014	0.0005	0.0758

Gene Set Name	Gene Set Genes #	DE Genes N (%)	Up-reg. DE Genes N (%)	Down-reg. DE Genes N (%)	Jaccard Index	P-Value	FDR Adjusted P-Value
GSE13485 DAY1 VS DAY7 YF17D VACCINE PBMC DN	199	3 (1.5)	3 (1.5)	0 (0)	0.014	0.0007	0.0758
GSE17721 LPS VS CPG 1H BMDC DN	199	3 (1.5)	3 (1.5)	0 (0)	0.014	0.0007	0.0758
GSE19198 6H VS 24H IL21 TREATED TCELL DN	198	3 (1.5)	3 (1.5)	0 (0)	0.014	0.0007	0.0758
GSE21360 PRIMARY VS TERTIARY MEMORY CD8 TCELL DN	199	3 (1.5)	3 (1.5)	0 (0)	0.014	0.0007	0.0758
GSE21774 CD62L POS CD56 BRIGHT VS CD62L NEG CD56 D...	199	3 (1.5)	3 (1.5)	0 (0)	0.014	0.0007	0.0758
GSE17721 CTRL VS PAM3CSK4 4H BMDC DN	200	3 (1.5)	3 (1.5)	0 (0)	0.014	0.0004	0.0758
GSE18281 SUBCAPSULAR VS CENTRAL CORTICAL REGION OF...	200	3 (1.5)	3 (1.5)	0 (0)	0.014	0.0004	0.0758
GSE35825 UNTREATED VS IFNG STIM MACROPHAGE UP	200	3 (1.5)	3 (1.5)	0 (0)	0.014	0.0006	0.0758
GSE46606 IRF4 KO VS WT UNSTIM BCELL DN	200	3 (1.5)	3 (1.5)	0 (0)	0.014	0.0006	0.0758
GSE17721 LPS VS CPG 12H BMDC UP	200	3 (1.5)	3 (1.5)	0 (0)	0.014	0.0007	0.0758
GSE34205 HEALTHY VS FLU INF INFANT PBMC DN	200	3 (1.5)	3 (1.5)	0 (0)	0.014	0.0007	0.0758
GSE34392 ST2 KO VS WT DAY8 LCMV EFFECTOR CD8 TCELL...	200	3 (1.5)	3 (1.5)	0 (0)	0.014	0.0007	0.0758
GSE17721 CPG VS GARDIQUIMOD 12H BMDC DN	202	3 (1.5)	3 (1.5)	0 (0)	0.014	0.0003	0.0758
GSE16522 ANTI CD3CD28 STIM VS UNSTIM MEMORY CD8 TC...	202	3 (1.5)	3 (1.5)	0 (0)	0.014	0.0004	0.0758
GSE26488 CTRL VS PEPTIDE INJECTION HDAC7 DELTAP TG...	202	3 (1.5)	3 (1.5)	0 (0)	0.014	0.0004	0.0758
GSE22140 GERMFREE VS SPF MOUSE CD4 TCELL UP	201	3 (1.5)	3 (1.5)	0 (0)	0.014	0.0005	0.0758
GSE24142 DN2 VS DN3 THYMOCYTE UP	202	3 (1.5)	3 (1.5)	0 (0)	0.014	0.0006	0.0758
GSE9006 TYPE 1 DIABETES AT DX VS 4MONTH POST DX PB...	201	3 (1.5)	3 (1.5)	0 (0)	0.014	0.0006	0.0758
GSE22140 HEALTHY VS ARTHRITIC GERMFREE MOUSE CD4 T...	203	3 (1.5)	3 (1.5)	0 (0)	0.014	0.0006	0.0758
GSE29615 CTRL VS DAY7 LAIV FLU VACCINE PBMC UP	184	3 (1.6)	3 (1.6)	0 (0)	0.015	0.0008	0.0779
GSE42724 NAIVE BCELL VS PLASMABLAST UP	192	3 (1.6)	3 (1.6)	0 (0)	0.014	0.0008	0.0779
GSE2706 UNSTIM VS 8H R848 DC DN	194	3 (1.5)	3 (1.5)	0 (0)	0.014	0.0008	0.0779
GSE41176 UNSTIM VS ANTI IGM STIM BCELL 1H UP	195	3 (1.5)	3 (1.5)	0 (0)	0.014	0.0008	0.0779
GSE14000 UNSTIM VS 4H LPS DC DN	197	3 (1.5)	3 (1.5)	0 (0)	0.014	0.0008	0.0779

Table 108: Enriched MSigDB Immunological Signatures (PBMC, Trivalent Influenza Vaccine, Day 3). Results are sorted by FDR adjusted p-value and Jaccard similarity index. Top 50 results are listed.

Gene Set Name	Gene Set Genes #	DE Genes N (%)	Up-reg. DE Genes N (%)	Down-reg. DE Genes N (%)	Jaccard Index	P-Value	FDR Adjusted P-Value
KEGG APOPTOSIS	89	3 (3.4)	3 (3.4)	0 (0)	0.029	<0.0001	0.0186
KEGG HEMATOPOIETIC CELL LINEAGE	85	3 (3.5)	3 (3.5)	0 (0)	0.030	0.0003	0.0279

Table 109: Enriched MSigDB KEGG Pathways (PBMC, Trivalent Influenza Vaccine, Day 3). Results are sorted by FDR adjusted p-value and Jaccard similarity index.

Gene Set Name	Gene Set Genes #	DE Genes N (%)	Up-reg. DE Genes N (%)	Down-reg. DE Genes N (%)	Jaccard Index	P-Value	FDR Adjusted P-Value
plasma cells and B cells, immunoglobulins (M156.0)	30	6 (20)	6 (20)	0 (0)	0.091	<0.0001	0.0115
plasma cells, immunoglobulins (M156.1)	22	5 (22.7)	5 (22.7)	0 (0)	0.085	<0.0001	0.0115
enriched in neutrophils (I) (M37.1)	47	6 (12.8)	6 (12.8)	0 (0)	0.072	<0.0001	0.0115

Table 110: Enriched Blood Transcription Modules (PBMC, Trivalent Influenza Vaccine, Day 5). Results are sorted by FDR adjusted p-value and Jaccard similarity index.

Gene Set Name	Gene Set Genes #	DE Genes N (%)	Up-reg. DE Genes N (%)	Down-reg. DE Genes N (%)	Jaccard Index	P-Value	FDR Adjusted P-Value
GO OXYGEN TRANSPORT	15	3 (20)	3 (20)	0 (0)	0.056	<0.0001	0.0517
GO GAS TRANSPORT	19	3 (15.8)	3 (15.8)	0 (0)	0.052	<0.0001	0.0517
GO HYDROGEN PEROXIDE CATABOLIC PROCESS	20	3 (15)	3 (15)	0 (0)	0.051	<0.0001	0.0517
GO HYDROGEN PEROXIDE METABOLIC PROCESS	30	3 (10)	3 (10)	0 (0)	0.043	<0.0001	0.0517
GO BICARBONATE TRANSPORT	44	3 (6.8)	3 (6.8)	0 (0)	0.036	<0.0001	0.0517
GO PROTEIN HETEROOLIGOMERIZATION	113	4 (3.5)	4 (3.5)	0 (0)	0.026	<0.0001	0.0517
GO RESPONSE TO TOXIC SUBSTANCE	241	5 (2.1)	5 (2.1)	0 (0)	0.018	<0.0001	0.0517
GO PROTEIN COMPLEX ASSEMBLY	1132	8 (0.7)	8 (0.7)	0 (0)	0.007	<0.0001	0.0517
GO PROTEIN COMPLEX BIOGENESIS	1132	8 (0.7)	8 (0.7)	0 (0)	0.007	<0.0001	0.0517
GO RECEPTOR MEDIATED ENDOCYTOSIS	213	4 (1.9)	4 (1.9)	0 (0)	0.016	0.0002	0.0846
GO PROTEIN OLIGOMERIZATION	432	5 (1.2)	5 (1.2)	0 (0)	0.011	0.0002	0.0846

Table 111: Enriched MSigDB Biological Processes (PBMC, Trivalent Influenza Vaccine, Day 5). Results are sorted by FDR adjusted p-value and Jaccard similarity index.

Gene Set Name	Gene Set Genes #	DE Genes N (%)	Up-reg. DE Genes N (%)	Down-reg. DE Genes N (%)	Jaccard Index	P-Value	FDR Adjusted P-Value
GO HEMOGLOBIN COMPLEX	12	3 (25)	3 (25)	0 (0)	0.059	<0.0001	0.0146
GO ENDOCYTIC VESICLE LUMEN	17	3 (17.6)	3 (17.6)	0 (0)	0.054	<0.0001	0.0146
GO VESICLE LUMEN	106	4 (3.8)	4 (3.8)	0 (0)	0.028	<0.0001	0.0146
GO BLOOD MICROPARTICLE	118	4 (3.4)	4 (3.4)	0 (0)	0.026	<0.0001	0.0146
GO CYTOSOLIC PART	220	4 (1.8)	4 (1.8)	0 (0)	0.015	0.0004	0.0467
GO ANCHORED COMPONENT OF MEMBRANE	149	3 (2)	3 (2)	0 (0)	0.016	0.0008	0.0779

Table 112: Enriched MSigDB Cellular Components (PBMC, Trivalent Influenza Vaccine, Day 5). Results are sorted by FDR adjusted p-value and Jaccard similarity index.

Gene Set Name	Gene Set Genes #	DE Genes N (%)	Up-reg. DE Genes N (%)	Down-reg. DE Genes N (%)	Jaccard Index	P-Value	FDR Adjusted P-Value
GSE29614 CTRL VS DAY7 TIV FLU VACCINE PBMC DN	183	6 (3.3)	6 (3.3)	0 (0)	0.027	<0.0001	0.0212
GSE29615 CTRL VS LAIV FLU VACCINE PBMC UP	189	6 (3.2)	6 (3.2)	0 (0)	0.027	<0.0001	0.0212
GSE13411 PLASMA CELL VS MEMORY BCELL UP	190	6 (3.2)	6 (3.2)	0 (0)	0.026	<0.0001	0.0212
GSE9006 HEALTHY VS TYPE 1 DIABETES PBMC AT DX DN	191	6 (3.1)	6 (3.1)	0 (0)	0.026	<0.0001	0.0212
GSE29614 CTRL VS TIV FLU VACCINE PBMC 2007 DN	172	5 (2.9)	5 (2.9)	0 (0)	0.024	<0.0001	0.0212
GSE29614 DAY3 VS DAY7 TIV FLU VACCINE PBMC DN	179	5 (2.8)	5 (2.8)	0 (0)	0.023	<0.0001	0.0212
GSE29615 CTRL VS DAY7 LAIV FLU VACCINE PBMC UP	184	5 (2.7)	5 (2.7)	0 (0)	0.023	<0.0001	0.0212
GSE29164 CD8 TCELL VS CD8 TCELL AND IL12 TREATED M...	188	5 (2.7)	5 (2.7)	0 (0)	0.022	<0.0001	0.0212
GSE17301 ACD3 ACD28 VS ACD3 ACD28 AND IFNA5 STIM C...	194	5 (2.6)	5 (2.6)	0 (0)	0.022	<0.0001	0.0212
GSE25088 WT VS STAT6 KO MACROPHAGE IL4 STIM DN	197	5 (2.5)	5 (2.5)	0 (0)	0.021	<0.0001	0.0212
GSE13547 CTRL VS ANTI IGM STIM BCELL 2H UP	180	4 (2.2)	4 (2.2)	0 (0)	0.018	<0.0001	0.0212
GSE29617 DAY3 VS DAY7 TIV FLU VACCINE PBMC 2008 DN	180	4 (2.2)	4 (2.2)	0 (0)	0.018	<0.0001	0.0212
GSE13547 CTRL VS ANTI IGM STIM BCELL 12H UP	182	4 (2.2)	4 (2.2)	0 (0)	0.018	<0.0001	0.0212
GSE29617 CTRL VS DAY7 TIV FLU VACCINE PBMC 2008 DN	187	4 (2.1)	4 (2.1)	0 (0)	0.018	<0.0001	0.0212
GSE17301 CTRL VS 48H ACD3 ACD28 IFNA2 STIM CD8 TCE...	198	4 (2)	4 (2)	0 (0)	0.017	<0.0001	0.0212

Gene Set Name	Gene Set Genes #	DE Genes N (%)	Up-reg. DE Genes N (%)	Down-reg. DE Genes N (%)	Jaccard Index	P-Value	FDR Adjusted P-Value
GSE28726 NAIVE VS ACTIVATED CD4 TCELL DN	199	4 (2)	4 (2)	0 (0)	0.017	<0.0001	0.0212
GSE36476 CTRL VS TSST ACT 40H MEMORY CD4 TCELL YOU...	200	4 (2)	4 (2)	0 (0)	0.017	<0.0001	0.0212
GSE15750 DAY6 VS DAY10 EFF CD8 TCELL UP	201	4 (2)	4 (2)	0 (0)	0.017	<0.0001	0.0212
GSE15750 DAY6 VS DAY10 TRAF6KO EFF CD8 TCELL UP	201	4 (2)	4 (2)	0 (0)	0.017	<0.0001	0.0212
GSE28726 NAIVE CD4 TCELL VS NAIVE VA24NEG NKTCELL ...	201	4 (2)	4 (2)	0 (0)	0.017	<0.0001	0.0212
GSE36476 CTRL VS TSST ACT 72H MEMORY CD4 TCELL OLD...	201	4 (2)	4 (2)	0 (0)	0.017	<0.0001	0.0212
GSE36476 CTRL VS TSST ACT 72H MEMORY CD4 TCELL YOU...	201	4 (2)	4 (2)	0 (0)	0.017	<0.0001	0.0212
GSE369 SOCS3 KO VS IFNG KO LIVER DN	203	4 (2)	4 (2)	0 (0)	0.017	<0.0001	0.0212
GSE32901 NAIVE VS TH17 NEG CD4 TCELL DN	123	3 (2.4)	3 (2.4)	0 (0)	0.018	0.0002	0.0348
GSE24634 IL4 VS CTRL TREATED NAIVE CD4 TCELL DAY7 ...	195	4 (2.1)	4 (2.1)	0 (0)	0.017	0.0002	0.0348
GSE24634 TREG VS TCONV POST DAY7 IL4 CONVERSION UP	196	4 (2)	4 (2)	0 (0)	0.017	0.0002	0.0348
GSE25088 WT VS STAT6 KO MACROPHAGE DN	196	4 (2)	4 (2)	0 (0)	0.017	0.0002	0.0348
GOLDRATH EFF VS MEMORY CD8 TCELL UP	200	4 (2)	4 (2)	0 (0)	0.017	0.0002	0.0348
GSE6269 E COLI VS STREP PNEUMO INF PBMC DN	164	3 (1.8)	3 (1.8)	0 (0)	0.015	0.0004	0.0629
GSE20727 CTRL VS ROS INHIBITOR TREATED DC DN	191	3 (1.6)	3 (1.6)	0 (0)	0.013	0.0004	0.0629
KAECH DAY8 EFF VS MEMORY CD8 TCELL UP	202	3 (1.5)	3 (1.5)	0 (0)	0.012	0.0004	0.0629
GSE32901 NAIVE VS TH17 NEG CD4 TCELL UP	168	3 (1.8)	3 (1.8)	0 (0)	0.015	0.0006	0.0713
GSE14415 NATURAL TREG VS TCONV DN	181	3 (1.7)	3 (1.7)	0 (0)	0.014	0.0006	0.0713
GSE19941 LPS VS LPS AND IL10 STIM IL10 KO MACROPHA...	182	3 (1.6)	3 (1.6)	0 (0)	0.014	0.0006	0.0713
GSE22886 NAIVE BCELL VS NEUTROPHIL DN	197	3 (1.5)	3 (1.5)	0 (0)	0.013	0.0006	0.0713
GSE2405 HEAT KILLED LYSATE VS LIVE A PHAGOCYTOPHIL...	199	3 (1.5)	3 (1.5)	0 (0)	0.013	0.0005	0.0713
GSE411 UNSTIM VS 100MIN IL6 STIM MACROPHAGE UP	200	3 (1.5)	3 (1.5)	0 (0)	0.013	0.0005	0.0713
GSE3337 4H VS 16H IFNG IN CD8POS DC UP	199	3 (1.5)	3 (1.5)	0 (0)	0.013	0.0006	0.0713
GSE7219 WT VS NIK NFKB2 KO LPS AND ANTI CD40 STIM ...	200	3 (1.5)	3 (1.5)	0 (0)	0.013	0.0006	0.0713
KAECH DAY8 EFF VS DAY15 EFF CD8 TCELL UP	201	3 (1.5)	3 (1.5)	0 (0)	0.013	0.0006	0.0713
GSE39556 CD8A DC VS NK CELL UP	202	3 (1.5)	3 (1.5)	0 (0)	0.012	0.0005	0.0713
GSE13547 2H VS 12 H ANTI IGM STIM BCELL UP	173	3 (1.7)	3 (1.7)	0 (0)	0.014	0.0007	0.0722
GSE13485 CTRL VS DAY7 YF17D VACCINE PBMC UP	179	3 (1.7)	3 (1.7)	0 (0)	0.014	0.0008	0.0722
GSE13485 PRE VS POST YF17D VACCINATION PBMC UP	184	3 (1.6)	3 (1.6)	0 (0)	0.013	0.0007	0.0722
GSE45365 HEALTHY VS MCMV INFECTION CD11B DC DN	190	3 (1.6)	3 (1.6)	0 (0)	0.013	0.0008	0.0722
GSE2405 S AUREUS VS UNTREATED NEUTROPHIL DN	198	3 (1.5)	3 (1.5)	0 (0)	0.013	0.0008	0.0722
GSE23568 CTRL VS ID3 TRANSDUCED CD8 TCELL DN	199	3 (1.5)	3 (1.5)	0 (0)	0.013	0.0007	0.0722
GSE32164 ALTERNATIVELY ACT M2 VS CMYC INHIBITED MA...	200	3 (1.5)	3 (1.5)	0 (0)	0.013	0.0007	0.0722
GSE360 L DONOVANI VS M TUBERCULOSIS DC DN	199	3 (1.5)	3 (1.5)	0 (0)	0.013	0.0007	0.0722
GSE36476 CTRL VS TSST ACT 40H MEMORY CD4 TCELL OLD...	200	3 (1.5)	3 (1.5)	0 (0)	0.013	0.0007	0.0722

Table 113: Enriched MSigDB Immunological Signatures (PBMC, Trivalent Influenza Vaccine, Day 5). Results are sorted by FDR adjusted p-value and Jaccard similarity index. Top 50 results are listed.

Gene Set Name	Gene Set Genes #	DE Genes N (%)	Up-reg. DE Genes N (%)	Down-reg. DE Genes N (%)	Jaccard Index	P-Value	FDR Adjusted P-Value
GO OXYGEN TRANSPORTER ACTIVITY	14	3 (21.4)	3 (21.4)	0 (0)	0.057	<0.0001	0.0232
GO OXYGEN BINDING	47	4 (8.5)	4 (8.5)	0 (0)	0.047	<0.0001	0.0232
GO OXIDOREDUCTASE ACTIVITY ACTING ON PEROXIDE AS A...	41	3 (7.3)	3 (7.3)	0 (0)	0.037	<0.0001	0.0232
GO ANTIOXIDANT ACTIVITY	69	3 (4.3)	3 (4.3)	0 (0)	0.028	<0.0001	0.0232

Gene Set Name	Gene Set Genes #	DE Genes N (%)	Up-reg. DE Genes N (%)	Down-reg. DE Genes N (%)	Jaccard Index	P-Value	FDR Adjusted P-Value
GO TETRAPYRROLE BINDING	133	4 (3)	4 (3)	0 (0)	0.023	0.0002	0.0265
GO IRON ION BINDING	162	4 (2.5)	4 (2.5)	0 (0)	0.020	0.0002	0.0265
GO OXIDOREDUCTASE ACTIVITY	712	6 (0.8)	6 (0.8)	0 (0)	0.008	0.0002	0.0265

Table 114: Enriched MSigDB Molecular Functions (PBMC, Trivalent Influenza Vaccine, Day 5). Results are sorted by FDR adjusted p-value and Jaccard similarity index.

Gene Set Name	Gene Set Genes #	DE Genes N (%)	Up-reg. DE Genes N (%)	Down-reg. DE Genes N (%)	Jaccard Index	P-Value	FDR Adjusted P-Value
plasma cells, immunoglobulins (M156.1)	22	14 (63.6)	14 (63.6)	0 (0)	0.135	<0.0001	0.0173
plasma cells and B cells, immunoglobulins (M156.0)	30	10 (33.3)	10 (33.3)	0 (0)	0.086	<0.0001	0.0173
enriched in B cells (II) (M47.1)	36	4 (11.1)	4 (11.1)	0 (0)	0.031	0.0003	0.0346

Table 115: Enriched Blood Transcription Modules (PBMC, Trivalent Influenza Vaccine, Day 6). Results are sorted by FDR adjusted p-value and Jaccard similarity index.

Gene Set Name	Gene Set Genes #	DE Genes N (%)	Up-reg. DE Genes N (%)	Down-reg. DE Genes N (%)	Jaccard Index	P-Value	FDR Adjusted P-Value
GO IMMUNOGLOBULIN COMPLEX	6	2 (33.3)	2 (33.3)	0 (0)	0.020	<0.0001	0.0584

Table 116: Enriched MSigDB Cellular Components (PBMC, Trivalent Influenza Vaccine, Day 6). Results are sorted by FDR adjusted p-value and Jaccard similarity index.

Gene Set Name	Gene Set Genes #	DE Genes N (%)	Up-reg. DE Genes N (%)	Down-reg. DE Genes N (%)	Jaccard Index	P-Value	FDR Adjusted P-Value
GSE29614 DAY3 VS DAY7 TIV FLU VACCINE PBMC DN	179	17 (9.5)	17 (9.5)	0 (0)	0.066	<0.0001	0.0139
GSE29614 CTRL VS DAY7 TIV FLU VACCINE PBMC DN	183	16 (8.7)	16 (8.7)	0 (0)	0.061	<0.0001	0.0139
GSE29614 CTRL VS TIV FLU VACCINE PBMC 2007 DN	172	10 (5.8)	10 (5.8)	0 (0)	0.039	<0.0001	0.0139
GSE29617 DAY3 VS DAY7 TIV FLU VACCINE PBMC 2008 DN	180	10 (5.6)	10 (5.6)	0 (0)	0.038	<0.0001	0.0139
GSE29617 CTRL VS DAY7 TIV FLU VACCINE PBMC 2008 DN	187	9 (4.8)	9 (4.8)	0 (0)	0.033	<0.0001	0.0139
GSE29164 CD8 TCELL VS CD8 TCELL AND IL12 TREATED M...	188	7 (3.7)	7 (3.7)	0 (0)	0.025	<0.0001	0.0139
GSE13411 PLASMA CELL VS MEMORY BCELL UP	190	7 (3.7)	7 (3.7)	0 (0)	0.025	<0.0001	0.0139
GSE20727 CTRL VS ROS INHIBITOR TREATED DC DN	191	7 (3.7)	7 (3.7)	0 (0)	0.025	<0.0001	0.0139
GSE10325 LUPUS CD4 TCELL VS LUPUS BCELL DN	184	6 (3.3)	6 (3.3)	0 (0)	0.022	<0.0001	0.0139
GSE369 SOCS3 KO VS IFNG KO LIVER DN	203	6 (3)	6 (3)	0 (0)	0.021	<0.0001	0.0139
GSE4984 UNTREATED VS GALECTIN1 TREATED DC DN	175	5 (2.9)	5 (2.9)	0 (0)	0.019	<0.0001	0.0139
GSE7509 UNSTIM VS IFNA STIM IMMATURE DC UP	177	5 (2.8)	5 (2.8)	0 (0)	0.019	<0.0001	0.0139
GSE29618 BCELL VS MONOCYTE UP	180	5 (2.8)	5 (2.8)	0 (0)	0.018	<0.0001	0.0139
GSE29618 BCELL VS MDC DAY7 FLU VACCINE UP	182	5 (2.7)	5 (2.7)	0 (0)	0.018	<0.0001	0.0139
GSE29618 BCELL VS MDC UP	184	5 (2.7)	5 (2.7)	0 (0)	0.018	<0.0001	0.0139
GSE29618 BCELL VS MONOCYTE DAY7 FLU VACCINE UP	184	5 (2.7)	5 (2.7)	0 (0)	0.018	<0.0001	0.0139
GSE12845 IGD POS VS NEG BLOOD BCELL DN	186	5 (2.7)	5 (2.7)	0 (0)	0.018	<0.0001	0.0139
GSE3982 MEMORY CD4 TCELL VS BCELL DN	193	5 (2.6)	5 (2.6)	0 (0)	0.018	<0.0001	0.0139

Gene Set Name	Gene Set Genes #	DE Genes N (%)	Up-reg. DE Genes N (%)	Down-reg. DE Genes N (%)	Jaccard Index	P-Value	FDR Adjusted P-Value
GSE22886 NAIVE BCELL VS BM PLASMA CELL DN	195	5 (2.6)	5 (2.6)	0 (0)	0.018	<0.0001	0.0139
GSE3982 BCELL VS NKCELL UP	198	5 (2.5)	5 (2.5)	0 (0)	0.017	<0.0001	0.0139
GSE23502 WT VS HDC KO MYELOID DERIVED SUPPRESSOR C...	200	5 (2.5)	5 (2.5)	0 (0)	0.017	<0.0001	0.0139
GSE7219 WT VS NIK NFKB2 KO LPS AND ANTI CD40 STIM ...	200	5 (2.5)	4 (2)	1 (0.5)	0.017	<0.0001	0.0139
GSE15750 DAY6 VS DAY10 TRAF6KO EFF CD8 TCELL UP	201	5 (2.5)	5 (2.5)	0 (0)	0.017	<0.0001	0.0139
GSE41867 DAY6 VS DAY8 LCMV ARMSTRONG EFFECTOR CD8 ...	201	5 (2.5)	5 (2.5)	0 (0)	0.017	<0.0001	0.0139
GSE12003 4D VS 8D CULTURE MIR223 KO BM PROGENITOR ...	164	4 (2.4)	3 (1.8)	1 (0.6)	0.016	<0.0001	0.0139
GSE13547 CTRL VS ANTI IGM STIM BCELL 2H UP	180	4 (2.2)	4 (2.2)	0 (0)	0.015	<0.0001	0.0139
GSE13547 CTRL VS ANTI IGM STIM BCELL 12H UP	182	4 (2.2)	4 (2.2)	0 (0)	0.015	<0.0001	0.0139
GSE11386 NAIVE VS MEMORY BCELL UP	183	4 (2.2)	4 (2.2)	0 (0)	0.015	<0.0001	0.0139
GSE10325 BCELL VS MYELOID UP	186	4 (2.2)	4 (2.2)	0 (0)	0.014	<0.0001	0.0139
GSE10325 CD4 TCELL VS BCELL DN	185	4 (2.2)	4 (2.2)	0 (0)	0.014	<0.0001	0.0139
GSE12366 GC BCELL VS PLASMA CELL DN	186	4 (2.2)	4 (2.2)	0 (0)	0.014	<0.0001	0.0139
GSE13411 IGM MEMORY BCELL VS PLASMA CELL DN	190	4 (2.1)	4 (2.1)	0 (0)	0.014	<0.0001	0.0139
GSE16266 LPS VS HEATSHOCK AND LPS STIM MEF UP	199	4 (2)	4 (2)	0 (0)	0.014	<0.0001	0.0139
GSE22432 CDC VS COMMON DC PROGENITOR UP	201	4 (2)	4 (2)	0 (0)	0.014	<0.0001	0.0139
KAECH DAY8 EFF VS MEMORY CD8 TCELL UP	202	4 (2)	4 (2)	0 (0)	0.014	<0.0001	0.0139
GSE32901 NAIVE VS TH17 NEG CD4 TCELL DN	123	4 (3.3)	4 (3.3)	0 (0)	0.019	0.0002	0.0217
GSE32164 ALTERNATIVELY ACT M2 VS CMYC INHIBITED MA...	200	5 (2.5)	5 (2.5)	0 (0)	0.017	0.0002	0.0217
GSE22886 IGG IGA MEMORY BCELL VS BM PLASMA CELL DN	190	4 (2.1)	4 (2.1)	0 (0)	0.014	0.0002	0.0217
GSE22886 NAIVE BCELL VS BLOOD PLASMA CELL DN	194	4 (2.1)	4 (2.1)	0 (0)	0.014	0.0002	0.0217
GSE28449 WT VS LRF KO GERMINAL CENTER BCELL UP	196	4 (2)	3 (1.5)	1 (0.5)	0.014	0.0002	0.0217
GOLDRATH NAIVE VS EFF CD8 TCELL DN	198	4 (2)	4 (2)	0 (0)	0.014	0.0002	0.0217
GSE25088 WT VS STAT6 KO MACROPHAGE IL4 STIM DN	197	4 (2)	4 (2)	0 (0)	0.014	0.0002	0.0217
GSE37605 C57BL6 VS NOD FOXP3 IRES GFP TREG DN	198	4 (2)	4 (2)	0 (0)	0.014	0.0002	0.0217
GSE30962 ACUTE VS CHRONIC LCMV PRIMARY INF CD8 TCE...	199	4 (2)	3 (1.5)	1 (0.5)	0.014	0.0002	0.0217
KAECH NAIVE VS DAY8 EFF CD8 TCELL DN	200	4 (2)	4 (2)	0 (0)	0.014	0.0002	0.0217
GSE15750 DAY6 VS DAY10 EFF CD8 TCELL UP	201	4 (2)	4 (2)	0 (0)	0.014	0.0003	0.0298
GSE1925 CTRL VS IFNG PRIMED MACROPHAGE UP	200	4 (2)	4 (2)	0 (0)	0.014	0.0003	0.0298
GSE21379 WT VS SAP KO CD4 TCELL DN	200	4 (2)	4 (2)	0 (0)	0.014	0.0003	0.0298
GSE33425 CD161 HIGH VS INT CD8 TCELL DN	199	4 (2)	4 (2)	0 (0)	0.014	0.0003	0.0298
GSE13547 2H VS 12 H ANTI IGM STIM BCELL UP	173	4 (2.3)	4 (2.3)	0 (0)	0.015	0.0004	0.0368

Table 117: Enriched MSigDB Immunological Signatures (PBMC, Trivalent Influenza Vaccine, Day 6). Results are sorted by FDR adjusted p-value and Jaccard similarity index. Top 50 results are listed.

Gene Set Name	Gene Set Genes #	DE Genes N (%)	Up-reg. DE Genes N (%)	Down-reg. DE Genes N (%)	Jaccard Index	P-Value	FDR Adjusted P-Value
GO IMMUNOGLOBULIN RECEPTOR BINDING	7	2 (28.6)	2 (28.6)	0 (0)	0.020	<0.0001	0.0929

Table 118: Enriched MSigDB Molecular Functions (PBMC, Trivalent Influenza Vaccine, Day 6). Results are sorted by FDR adjusted p-value and Jaccard similarity index.

Gene Set Name	Gene Set Genes #	DE Genes N (%)	Up-reg. DE Genes N (%)	Down-reg. DE Genes N (%)	Jaccard Index	P-Value	FDR Adjusted P-Value
plasma cells, immunoglobulins (M156.1)	22	10 (45.5)	10 (45.5)	0 (0)	0.135	<0.0001	0.0115
plasma cells and B cells, immunoglobulins (M156.0)	30	7 (23.3)	7 (23.3)	0 (0)	0.082	<0.0001	0.0115
enriched in B cells (II) (M47.1)	36	4 (11.1)	4 (11.1)	0 (0)	0.043	<0.0001	0.0115

Table 119: Enriched Blood Transcription Modules (PBMC, Trivalent Influenza Vaccine, Day 7). Results are sorted by FDR adjusted p-value and Jaccard similarity index.

Gene Set Name	Gene Set Genes #	DE Genes N (%)	Up-reg. DE Genes N (%)	Down-reg. DE Genes N (%)	Jaccard Index	P-Value	FDR Adjusted P-Value
GSE29614 DAY3 VS DAY7 TIV FLU VACCINE PBMC DN	179	12 (6.7)	12 (6.7)	0 (0)	0.052	<0.0001	0.0271
GSE29614 CTRL VS DAY7 TIV FLU VACCINE PBMC DN	183	11 (6)	11 (6)	0 (0)	0.047	<0.0001	0.0271
GSE29617 DAY3 VS DAY7 TIV FLU VACCINE PBMC 2008 DN	180	10 (5.6)	10 (5.6)	0 (0)	0.043	<0.0001	0.0271
GSE29617 CTRL VS DAY7 TIV FLU VACCINE PBMC 2008 DN	187	8 (4.3)	8 (4.3)	0 (0)	0.033	<0.0001	0.0271
GSE29614 CTRL VS TIV FLU VACCINE PBMC 2007 DN	172	6 (3.5)	6 (3.5)	0 (0)	0.026	<0.0001	0.0271
GSE29164 CD8 TCELL VS CD8 TCELL AND IL12 TREATED M...	188	6 (3.2)	6 (3.2)	0 (0)	0.025	<0.0001	0.0271
GSE4984 UNTREATED VS GALECTIN1 TREATED DC DN	175	5 (2.9)	5 (2.9)	0 (0)	0.022	<0.0001	0.0271
GSE10325 LUPUS CD4 TCELL VS LUPUS BCELL DN	184	5 (2.7)	5 (2.7)	0 (0)	0.021	<0.0001	0.0271
GSE45365 WT VS IFNAR KO BCELL MCMV INFECTION DN	188	5 (2.7)	5 (2.7)	0 (0)	0.020	<0.0001	0.0271
GSE3982 MEMORY CD4 TCELL VS BCELL DN	193	5 (2.6)	5 (2.6)	0 (0)	0.020	<0.0001	0.0271
GSE22886 NAIVE BCELL VS BM PLASMA CELL DN	195	5 (2.6)	5 (2.6)	0 (0)	0.020	<0.0001	0.0271
GSE5589 WT VS IL10 KO LPS STIM MACROPHAGE 180MIN D...	150	4 (2.7)	4 (2.7)	0 (0)	0.019	<0.0001	0.0271
GSE29618 BCELL VS MDC DAY7 FLU VACCINE UP	182	4 (2.2)	4 (2.2)	0 (0)	0.017	<0.0001	0.0271
GSE10325 CD4 TCELL VS BCELL DN	185	4 (2.2)	4 (2.2)	0 (0)	0.017	<0.0001	0.0271
GSE10325 BCELL VS MYELOID UP	186	4 (2.2)	4 (2.2)	0 (0)	0.016	<0.0001	0.0271
GSE13411 PLASMA CELL VS MEMORY BCELL UP	190	4 (2.1)	4 (2.1)	0 (0)	0.016	<0.0001	0.0271
GSE22886 IGG IGA MEMORY BCELL VS BM PLASMA CELL DN	190	4 (2.1)	4 (2.1)	0 (0)	0.016	<0.0001	0.0271
GSE22432 CDC VS COMMON DC PROGENITOR UP	201	4 (2)	4 (2)	0 (0)	0.015	<0.0001	0.0271
GSE13547 CTRL VS ANTI IGM STIM ZFX KO BCELL 2H UP	162	4 (2.5)	4 (2.5)	0 (0)	0.018	0.0002	0.0443
GSE29618 BCELL VS MONOCYTE UP	180	4 (2.2)	4 (2.2)	0 (0)	0.017	0.0002	0.0443
GSE3982 BCELL VS NKCELL UP	198	4 (2)	4 (2)	0 (0)	0.016	0.0002	0.0443
GSE41867 DAY6 VS DAY8 LCMV ARMSTRONG EFFECTOR CD8 ...	201	4 (2)	4 (2)	0 (0)	0.015	0.0002	0.0443
GSE22886 NAIVE VS IGG IGA MEMORY BCELL DN	193	4 (2.1)	4 (2.1)	0 (0)	0.016	0.0003	0.0635
GSE17721 CTRL VS PAM3CSK4 6H BMDC UP	201	4 (2)	4 (2)	0 (0)	0.015	0.0004	0.0812
GSE32901 NAIVE VS TH17 NEG CD4 TCELL DN	123	3 (2.4)	3 (2.4)	0 (0)	0.017	0.0005	0.0974

Table 120: Enriched MSigDB Immunological Signatures (PBMC, Trivalent Influenza Vaccine, Day 7). Results are sorted by FDR adjusted p-value and Jaccard similarity index.

Gene Set Name	Gene Set Genes #	DE Genes N (%)	Up-reg. DE Genes N (%)	Down-reg. DE Genes N (%)	Jaccard Index	P-Value	FDR Adjusted P-Value
enriched in neutrophils (I) (M37.1)	47	10 (21.3)	10 (21.3)	0 (0)	0.103	<0.0001	0.0069
plasma cells, immunoglobulins (M156.1)	22	7 (31.8)	7 (31.8)	0 (0)	0.093	<0.0001	0.0069
plasma cells and B cells, immunoglobulins (M156.0)	30	6 (20)	6 (20)	0 (0)	0.071	<0.0001	0.0069
TLR and inflammatory signaling (M16)	43	6 (14)	6 (14)	0 (0)	0.062	<0.0001	0.0069

Gene Set Name	Gene Set Genes #	DE Genes N (%)	Up-reg. DE Genes N (%)	Down-reg. DE Genes N (%)	Jaccard Index	P-Value	FDR Adjusted P-Value
recruitment of neutrophils (M132)	10	3 (30)	3 (30)	0 (0)	0.045	<0.0001	0.0069
antiviral IFN signature (M75)	22	4 (18.2)	4 (18.2)	0 (0)	0.051	0.0003	0.0173
enriched in activated dendritic cells (II) (M165)	35	4 (11.4)	4 (11.4)	0 (0)	0.044	0.0008	0.0395

Table 121: Enriched Blood Transcription Modules (PBMC, Trivalent Influenza Vaccine, Day 8). Results are sorted by FDR adjusted p-value and Jaccard similarity index.

Gene Set Name	Gene Set Genes #	DE Genes N (%)	Up-reg. DE Genes N (%)	Down-reg. DE Genes N (%)	Jaccard Index	P-Value	FDR Adjusted P-Value
GO RESPONSE TO INTERFERON ALPHA	20	3 (15)	3 (15)	0 (0)	0.039	<0.0001	0.0233
GO CELLULAR RESPONSE TO FATTY ACID	51	4 (7.8)	4 (7.8)	0 (0)	0.037	<0.0001	0.0233
GO RESPONSE TO TYPE I INTERFERON	67	4 (6)	4 (6)	0 (0)	0.033	<0.0001	0.0233
GO LEUKOCYTE CHEMOTAXIS	116	5 (4.3)	4 (3.4)	1 (0.9)	0.029	<0.0001	0.0233
GO RESPONSE TO FATTY ACID	83	4 (4.8)	4 (4.8)	0 (0)	0.029	<0.0001	0.0233
GO CELL CHEMOTAXIS	161	5 (3.1)	4 (2.5)	1 (0.6)	0.023	<0.0001	0.0233
GO CELLULAR RESPONSE TO ACID CHEMICAL	175	5 (2.9)	4 (2.3)	1 (0.6)	0.022	<0.0001	0.0233
GO POSITIVE REGULATION OF VASCULATURE DEVELOPMENT	133	4 (3)	3 (2.3)	1 (0.8)	0.021	<0.0001	0.0233
GO CYTOKINE MEDIATED SIGNALING PATHWAY	448	10 (2.2)	9 (2)	1 (0.2)	0.020	<0.0001	0.0233
GO INFLAMMATORY RESPONSE	451	10 (2.2)	9 (2)	1 (0.2)	0.020	<0.0001	0.0233
GO CELLULAR RESPONSE TO CYTOKINE STIMULUS	602	12 (2)	11 (1.8)	1 (0.2)	0.018	<0.0001	0.0233
GO RESPONSE TO CYTOKINE	710	12 (1.7)	11 (1.5)	1 (0.1)	0.016	<0.0001	0.0233
GO DEFENSE RESPONSE	1197	19 (1.6)	18 (1.5)	1 (0.1)	0.015	<0.0001	0.0233
GO DEFENSE RESPONSE TO OTHER ORGANISM	483	8 (1.7)	8 (1.7)	0 (0)	0.015	<0.0001	0.0233
GO REGULATION OF IMMUNE EFFECTOR PROCESS	426	7 (1.6)	7 (1.6)	0 (0)	0.015	<0.0001	0.0233
GO IMMUNE RESPONSE	1052	14 (1.3)	13 (1.2)	1 (0.1)	0.013	<0.0001	0.0233
GO RESPONSE TO BIOTIC STIMULUS	863	11 (1.3)	10 (1.2)	1 (0.1)	0.012	<0.0001	0.0233
GO RESPONSE TO EXTERNAL STIMULUS	1798	17 (0.9)	16 (0.9)	1 (0.1)	0.009	<0.0001	0.0233
GO CELLULAR RESPONSE TO ORGANIC SUBSTANCE	1848	17 (0.9)	15 (0.8)	2 (0.1)	0.009	<0.0001	0.0233
GO IMMUNE SYSTEM PROCESS	1932	17 (0.9)	16 (0.8)	1 (0.1)	0.009	<0.0001	0.0233
GO LIPID STORAGE	27	3 (11.1)	3 (11.1)	0 (0)	0.036	0.0002	0.0443
GO GRANULOCYTE MIGRATION	74	3 (4.1)	2 (2.7)	1 (1.4)	0.023	0.0003	0.0582
GO RESPONSE TO VIRUS	248	5 (2)	4 (1.6)	1 (0.4)	0.017	0.0003	0.0582
GO INNATE IMMUNE RESPONSE	589	7 (1.2)	6 (1)	1 (0.2)	0.011	0.0003	0.0582
GO RESPONSE TO ACID CHEMICAL	320	5 (1.6)	4 (1.2)	1 (0.3)	0.013	0.0004	0.0716
GO IMMUNE EFFECTOR PROCESS	456	6 (1.3)	6 (1.3)	0 (0)	0.012	0.0004	0.0716
GO DEFENSE RESPONSE TO VIRUS	165	4 (2.4)	4 (2.4)	0 (0)	0.018	0.0005	0.0862
GO CHEMOKINE MEDIATED SIGNALING PATHWAY	71	3 (4.2)	2 (2.8)	1 (1.4)	0.023	0.0006	0.0931
GO POSITIVE REGULATION OF ERK1 AND ERK2 CASCADE	169	4 (2.4)	2 (1.2)	2 (1.2)	0.018	0.0006	0.0931
GO HOMEOSTATIC PROCESS	1333	10 (0.8)	10 (0.8)	0 (0)	0.007	0.0006	0.0931
GO DENDRITIC CELL CHEMOTAXIS	16	2 (12.5)	2 (12.5)	0 (0)	0.027	0.0007	0.0979
GO NEGATIVE REGULATION OF GLUCOSE TRANSPORT	17	2 (11.8)	2 (11.8)	0 (0)	0.027	0.0007	0.0979
GO DENDRITIC CELL MIGRATION	21	2 (9.5)	2 (9.5)	0 (0)	0.025	0.0008	0.0979
GO REGULATION OF ACUTE INFLAMMATORY RESPONSE	74	3 (4.1)	3 (4.1)	0 (0)	0.023	0.0008	0.0979
GO NEGATIVE REGULATION OF LIPID METABOLIC PROCESS	80	3 (3.8)	3 (3.8)	0 (0)	0.022	0.0008	0.0979
GO LEUKOCYTE MIGRATION	258	5 (1.9)	4 (1.6)	1 (0.4)	0.016	0.0007	0.0979
GO TAXIS	463	6 (1.3)	5 (1.1)	1 (0.2)	0.012	0.0008	0.0979

Gene Set Name	Gene Set Genes #	DE Genes N (%)	Up-reg. DE Genes N (%)	Down-reg. DE Genes N (%)	Jaccard Index	P-Value	FDR Adjusted P-Value
GO REGULATION OF IMMUNE SYSTEM PROCESS	1369	10 (0.7)	8 (0.6)	2 (0.1)	0.007	0.0008	0.0979

Table 122: Enriched MSigDB Biological Processes (PBMC, Trivalent Influenza Vaccine, Day 8). Results are sorted by FDR adjusted p-value and Jaccard similarity index.

Gene Set Name	Gene Set Genes #	DE Genes N (%)	Up-reg. DE Genes N (%)	Down-reg. DE Genes N (%)	Jaccard Index	P-Value	FDR Adjusted P-Value
GSE22886 NAIVE BCELL VS NEUTROPHIL DN	197	14 (7.1)	14 (7.1)	0 (0)	0.058	<0.0001	0.0092
GSE9006 HEALTHY VS TYPE 1 DIABETES PBMC AT DX DN	191	12 (6.3)	12 (6.3)	0 (0)	0.050	<0.0001	0.0092
GSE22886 NAIVE TCELL VS NEUTROPHIL DN	197	12 (6.1)	12 (6.1)	0 (0)	0.049	<0.0001	0.0092
GSE29615 CTRL VS LAIV FLU VACCINE PBMC UP	189	8 (4.2)	8 (4.2)	0 (0)	0.033	<0.0001	0.0092
GSE22886 NAIVE CD8 TCELL VS NEUTROPHIL DN	194	8 (4.1)	8 (4.1)	0 (0)	0.033	<0.0001	0.0092
GSE22886 NEUTROPHIL VS MONOCYTE UP	195	8 (4.1)	8 (4.1)	0 (0)	0.032	<0.0001	0.0092
GSE3982 DC VS NEUTROPHIL DN	198	8 (4)	6 (3)	2 (1)	0.032	<0.0001	0.0092
GSE13485 DAY3 VS DAY7 YF17D VACCINE PBMC DN	199	8 (4)	7 (3.5)	1 (0.5)	0.032	<0.0001	0.0092
GSE13485 PRE VS POST YF17D VACCINATION PBMC UP	184	7 (3.8)	7 (3.8)	0 (0)	0.029	<0.0001	0.0092
GSE29615 CTRL VS DAY7 LAIV FLU VACCINE PBMC UP	184	7 (3.8)	7 (3.8)	0 (0)	0.029	<0.0001	0.0092
GSE22886 NAIVE CD4 TCELL VS NEUTROPHIL DN	196	7 (3.6)	7 (3.6)	0 (0)	0.028	<0.0001	0.0092
GSE360 L DONOVANI VS M TUBERCULOSIS DC DN	199	7 (3.5)	6 (3)	1 (0.5)	0.028	<0.0001	0.0092
GSE13484 UNSTIM VS YF17D VACCINE STIM PBMC DN	200	7 (3.5)	6 (3)	1 (0.5)	0.028	<0.0001	0.0092
GSE34392 ST2 KO VS WT DAY8 LCMV EFFECTOR CD8 TCELL...	200	7 (3.5)	7 (3.5)	0 (0)	0.028	<0.0001	0.0092
GSE19401 PLN VS PEYERS PATCH FOLLICULAR DC DN	201	7 (3.5)	6 (3)	1 (0.5)	0.028	<0.0001	0.0092
GSE14000 UNSTIM VS 4H LPS DC DN	197	6 (3)	5 (2.5)	1 (0.5)	0.024	<0.0001	0.0092
GSE40685 TREG VS FOXP3 KO TREG PRECURSOR DN	197	6 (3)	6 (3)	0 (0)	0.024	<0.0001	0.0092
GSE13485 CTRL VS DAY3 YF17D VACCINE PBMC DN	198	6 (3)	6 (3)	0 (0)	0.024	<0.0001	0.0092
GSE14000 UNSTIM VS 4H LPS DC TRANSLATED RNA DN	198	6 (3)	5 (2.5)	1 (0.5)	0.024	<0.0001	0.0092
GSE10325 MYELOID VS LUPUS MYELOID DN	199	6 (3)	6 (3)	0 (0)	0.024	<0.0001	0.0092
GSE13485 PRE VS POST YF17D VACCINATION PBMC DN	199	6 (3)	6 (3)	0 (0)	0.024	<0.0001	0.0092
GSE19888 ADENOSINE A3R INH VS ACT WITH INHIBITOR P...	200	6 (3)	5 (2.5)	1 (0.5)	0.024	<0.0001	0.0092
GSE7218 IGM VS IGG SIGNAL THGOUGH ANTIGEN BCELL DN	169	5 (3)	4 (2.4)	1 (0.6)	0.022	<0.0001	0.0092
GSE13485 CTRL VS DAY3 YF17D VACCINE PBMC UP	175	5 (2.9)	5 (2.9)	0 (0)	0.022	<0.0001	0.0092
GSE17974 IL4 AND ANTI IL12 VS UNTREATED 24H ACT CD...	181	5 (2.8)	4 (2.2)	1 (0.6)	0.021	<0.0001	0.0092
GSE2706 R848 VS R848 AND LPS 2H STIM DC DN	188	5 (2.7)	4 (2.1)	1 (0.5)	0.021	<0.0001	0.0092
GSE29164 CD8 TCELL VS CD8 TCELL AND IL12 TREATED M...	188	5 (2.7)	5 (2.7)	0 (0)	0.021	<0.0001	0.0092
GSE29615 CTRL VS DAY3 LAIV IFLU VACCINE PBMC UP	189	5 (2.6)	5 (2.6)	0 (0)	0.021	<0.0001	0.0092
GSE2706 UNSTIM VS 2H R848 DC DN	190	5 (2.6)	4 (2.1)	1 (0.5)	0.020	<0.0001	0.0092
GSE4748 CYANOBACTERIUM LPSLIKE VS LPS AND CYANOBAC...	190	5 (2.6)	5 (2.6)	0 (0)	0.020	<0.0001	0.0092
GSE18791 CTRL VS NEWCASTLE VIRUS DC 8H DN	193	5 (2.6)	5 (2.6)	0 (0)	0.020	<0.0001	0.0092
GSE41176 UNSTIM VS ANTI IGM STIM BCELL 1H UP	195	5 (2.6)	4 (2.1)	1 (0.5)	0.020	<0.0001	0.0092
GSE9006 1MONTH VS 4MONTH POST TYPE 1 DIABETES DX P...	195	5 (2.6)	5 (2.6)	0 (0)	0.020	<0.0001	0.0092
GSE36888 UNTREATED VS IL2 TREATED STAT5 AB KNOCKIN...	196	5 (2.6)	4 (2)	1 (0.5)	0.020	<0.0001	0.0092
GSE25123 WT VS PPARG KO MACROPHAGE UP	198	5 (2.5)	5 (2.5)	0 (0)	0.020	<0.0001	0.0092
GSE3982 CTRL VS LPS 48H DC DN	198	5 (2.5)	4 (2)	1 (0.5)	0.020	<0.0001	0.0092
GSE3982 NEUTROPHIL VS NKCELL UP	198	5 (2.5)	5 (2.5)	0 (0)	0.020	<0.0001	0.0092
GSE42021 CD24HI VS CD24LOW TREG THYMUS DN	198	5 (2.5)	5 (2.5)	0 (0)	0.020	<0.0001	0.0092
GSE9988 ANTI TREM1 VS LPS MONOCYTE DN	197	5 (2.5)	4 (2)	1 (0.5)	0.020	<0.0001	0.0092

Gene Set Name	Gene Set Genes #	DE Genes N (%)	Up-reg. DE Genes N (%)	Down-reg. DE Genes N (%)	Jaccard Index	P-Value	FDR Adjusted P-Value
GSE36392 TYPE 2 MYELOID VS EOSINOPHIL IL25 TREATED...	199	5 (2.5)	5 (2.5)	0 (0)	0.020	<0.0001	0.0092
GSE18281 SUBCAPSULAR VS CENTRAL CORTICAL REGION OF...	200	5 (2.5)	5 (2.5)	0 (0)	0.020	<0.0001	0.0092
GSE32986 CURDLAN LOWDOSE VS CURDLAN HIGHDOSE STIM ...	200	5 (2.5)	4 (2)	1 (0.5)	0.020	<0.0001	0.0092
GSE33424 CD161 INT VS NEG CD8 TCELL UP	200	5 (2.5)	4 (2)	1 (0.5)	0.020	<0.0001	0.0092
GSE42021 TREG VS TCONV PLN UP	200	5 (2.5)	5 (2.5)	0 (0)	0.020	<0.0001	0.0092
GSE7548 NAIVE VS DAY7 PCC IMMUNIZATION CD4 TCELL D...	200	5 (2.5)	4 (2)	1 (0.5)	0.020	<0.0001	0.0092
GSE8835 CD4 VS CD8 TCELL CLL PATIENT UP	200	5 (2.5)	4 (2)	1 (0.5)	0.020	<0.0001	0.0092
GSE13485 CTRL VS DAY7 YF17D VACCINE PBMC DN	202	5 (2.5)	5 (2.5)	0 (0)	0.019	<0.0001	0.0092
GSE19198 1H VS 6H IL21 TREATED TCELL DN	201	5 (2.5)	4 (2)	1 (0.5)	0.019	<0.0001	0.0092
GSE22140 GERMFREE VS SPF MOUSE CD4 TCELL DN	201	5 (2.5)	4 (2)	1 (0.5)	0.019	<0.0001	0.0092
GSE26030 TH1 VS TH17 DAY5 POST POLARIZATION UP	201	5 (2.5)	4 (2)	1 (0.5)	0.019	<0.0001	0.0092

Table 123: Enriched MSigDB Immunological Signatures (PBMC, Trivalent Influenza Vaccine, Day 8). Results are sorted by FDR adjusted p-value and Jaccard similarity index. Top 50 results are listed.

Gene Set Name	Gene Set Genes #	DE Genes N (%)	Up-reg. DE Genes N (%)	Down-reg. DE Genes N (%)	Jaccard Index	P-Value	FDR Adjusted P-Value
KEGG CYTOKINE CYTOKINE RECEPTOR INTERACTION	263	6 (2.3)	5 (1.9)	1 (0.4)	0.019	<0.0001	0.0186

Table 124: Enriched MSigDB KEGG Pathways (PBMC, Trivalent Influenza Vaccine, Day 8). Results are sorted by FDR adjusted p-value and Jaccard similarity index.

Gene Set Name	Gene Set Genes #	DE Genes N (%)	Up-reg. DE Genes N (%)	Down-reg. DE Genes N (%)	Jaccard Index	P-Value	FDR Adjusted P-Value
REACTOME INTERFERON ALPHA BETA SIGNALING	62	4 (6.5)	4 (6.5)	0 (0)	0.034	<0.0001	0.0674

Table 125: Enriched MSigDB Reactome Pathways (PBMC, Trivalent Influenza Vaccine, Day 8). Results are sorted by FDR adjusted p-value and Jaccard similarity index.

Gene Set Name	Gene Set Genes #	DE Genes N (%)	Up-reg. DE Genes N (%)	Down-reg. DE Genes N (%)	Jaccard Index	P-Value	FDR Adjusted P-Value
GO OXYGEN TRANSPORT	15	3 (20)	3 (20)	0 (0)	0.125	<0.0001	0.0465
GO GAS TRANSPORT	19	3 (15.8)	3 (15.8)	0 (0)	0.107	<0.0001	0.0465
GO HYDROGEN PEROXIDE CATABOLIC PROCESS	20	3 (15)	3 (15)	0 (0)	0.103	<0.0001	0.0465
GO HYDROGEN PEROXIDE METABOLIC PROCESS	30	3 (10)	3 (10)	0 (0)	0.077	<0.0001	0.0465
GO BICARBONATE TRANSPORT	44	3 (6.8)	3 (6.8)	0 (0)	0.057	<0.0001	0.0465
GO REACTIVE OXYGEN SPECIES METABOLIC PROCESS	96	4 (4.2)	4 (4.2)	0 (0)	0.038	<0.0001	0.0465
GO RESPONSE TO HYDROGEN PEROXIDE	109	3 (2.8)	3 (2.8)	0 (0)	0.025	<0.0001	0.0465
GO PROTEIN HETEROOLIGOMERIZATION	113	3 (2.7)	3 (2.7)	0 (0)	0.025	<0.0001	0.0465
GO RECEPTOR MEDIATED ENDOCYTOSIS	213	3 (1.4)	3 (1.4)	0 (0)	0.013	<0.0001	0.0465
GO RESPONSE TO INORGANIC SUBSTANCE	484	3 (0.6)	3 (0.6)	0 (0)	0.006	<0.0001	0.0465
GO DETOXIFICATION	76	3 (3.9)	3 (3.9)	0 (0)	0.035	0.0002	0.0665

Gene Set Name	Gene Set Genes #	DE Genes N (%)	Up-reg. DE Genes N (%)	Down-reg. DE Genes N (%)	Jaccard Index	P-Value	FDR Adjusted P-Value
GO RESPONSE TO REACTIVE OXYGEN SPECIES	191	3 (1.6)	3 (1.6)	0 (0)	0.015	0.0002	0.0665
GO RESPONSE TO TOXIC SUBSTANCE	241	3 (1.2)	3 (1.2)	0 (0)	0.012	0.0002	0.0665
GO RESPONSE TO OXIDATIVE STRESS	352	3 (0.9)	3 (0.9)	0 (0)	0.008	0.0002	0.0665
GO PROTEIN OLIGOMERIZATION	432	3 (0.7)	3 (0.7)	0 (0)	0.007	0.0003	0.0821
GO ENDOCYTOSIS	483	3 (0.6)	3 (0.6)	0 (0)	0.006	0.0003	0.0821
GO RESPONSE TO OXYGEN CONTAINING COMPOUND	1385	4 (0.3)	4 (0.3)	0 (0)	0.003	0.0003	0.0821

Table 126: Enriched MSigDB Biological Processes (PBMC, Trivalent Influenza Vaccine, Day 9). Results are sorted by FDR adjusted p-value and Jaccard similarity index.

Gene Set Name	Gene Set Genes #	DE Genes N (%)	Up-reg. DE Genes N (%)	Down-reg. DE Genes N (%)	Jaccard Index	P-Value	FDR Adjusted P-Value
GO HEMOGLOBIN COMPLEX	12	3 (25)	3 (25)	0 (0)	0.143	<0.0001	0.0117
GO ENDOCYTIC VESICLE LUMEN	17	3 (17.6)	3 (17.6)	0 (0)	0.115	<0.0001	0.0117
GO VESICLE LUMEN	106	3 (2.8)	3 (2.8)	0 (0)	0.026	<0.0001	0.0117
GO BLOOD MICROPARTICLE	118	3 (2.5)	3 (2.5)	0 (0)	0.024	<0.0001	0.0117
GO ENDOCYTIC VESICLE	255	3 (1.2)	3 (1.2)	0 (0)	0.011	<0.0001	0.0117
GO CYTOSOLIC SMALL RIBOSOMAL SUBUNIT	43	2 (4.7)	2 (4.7)	0 (0)	0.038	0.0002	0.0195
GO SMALL RIBOSOMAL SUBUNIT	67	2 (3)	2 (3)	0 (0)	0.026	0.0003	0.0219
GO CYTOSOLIC PART	220	3 (1.4)	3 (1.4)	0 (0)	0.013	0.0003	0.0219
GO CYTOPLASMIC VESICLE PART	600	3 (0.5)	3 (0.5)	0 (0)	0.005	0.001	0.0649
GO CYTOSOLIC RIBOSOME	110	2 (1.8)	2 (1.8)	0 (0)	0.017	0.0013	0.0759

Table 127: Enriched MSigDB Cellular Components (PBMC, Trivalent Influenza Vaccine, Day 9). Results are sorted by FDR adjusted p-value and Jaccard similarity index.

Gene Set Name	Gene Set Genes #	DE Genes N (%)	Up-reg. DE Genes N (%)	Down-reg. DE Genes N (%)	Jaccard Index	P-Value	FDR Adjusted P-Value
GO OXYGEN TRANSPORTER ACTIVITY	14	3 (21.4)	3 (21.4)	0 (0)	0.130	<0.0001	0.0186
GO OXIDOREDUCTASE ACTIVITY ACTING ON PEROXIDE AS A...	41	3 (7.3)	3 (7.3)	0 (0)	0.060	<0.0001	0.0186
GO OXYGEN BINDING	47	3 (6.4)	3 (6.4)	0 (0)	0.054	<0.0001	0.0186
GO ANTIOXIDANT ACTIVITY	69	3 (4.3)	3 (4.3)	0 (0)	0.038	<0.0001	0.0186
GO TETRAPYRROLE BINDING	133	3 (2.3)	3 (2.3)	0 (0)	0.021	<0.0001	0.0186
GO IRON ION BINDING	162	3 (1.9)	3 (1.9)	0 (0)	0.018	0.0002	0.031

Table 128: Enriched MSigDB Molecular Functions (PBMC, Trivalent Influenza Vaccine, Day 9). Results are sorted by FDR adjusted p-value and Jaccard similarity index.

Gene Set Name	Gene Set Genes #	DE Genes N (%)	Up-reg. DE Genes N (%)	Down-reg. DE Genes N (%)	Jaccard Index	P-Value	FDR Adjusted P-Value
enriched in neutrophils (I) (M37.1)	47	14 (29.8)	14 (29.8)	0 (0)	0.206	<0.0001	0.0346

Table 129: Enriched Blood Transcription Modules (PBMC, Trivalent Influenza Vaccine, Day 10). Results are sorted by FDR adjusted p-value and Jaccard similarity index.

Gene Set Name	Gene Set Genes #	DE Genes N (%)	Up-reg. DE Genes N (%)	Down-reg. DE Genes N (%)	Jaccard Index	P-Value	FDR Adjusted P-Value
GO OXYGEN TRANSPORT	15	4 (26.7)	4 (26.7)	0 (0)	0.087	<0.0001	0.0517
GO GAS TRANSPORT	19	4 (21.1)	4 (21.1)	0 (0)	0.080	<0.0001	0.0517
GO HYDROGEN PEROXIDE CATABOLIC PROCESS	20	3 (15)	3 (15)	0 (0)	0.058	<0.0001	0.0517
GO BICARBONATE TRANSPORT	44	4 (9.1)	4 (9.1)	0 (0)	0.053	<0.0001	0.0517
GO HYDROGEN PEROXIDE METABOLIC PROCESS	30	3 (10)	3 (10)	0 (0)	0.048	<0.0001	0.0517
GO RECEPTOR INTERNALIZATION	50	3 (6)	3 (6)	0 (0)	0.037	<0.0001	0.0517
GO REACTIVE OXYGEN SPECIES METABOLIC PROCESS	96	4 (4.2)	4 (4.2)	0 (0)	0.032	<0.0001	0.0517
GO RECEPTOR MEDIATED ENDOCYTOSIS	213	6 (2.8)	6 (2.8)	0 (0)	0.025	<0.0001	0.0517
GO DEFENSE RESPONSE	1197	10 (0.8)	10 (0.8)	0 (0)	0.008	<0.0001	0.0517

Table 130: Enriched MSigDB Biological Processes (PBMC, Trivalent Influenza Vaccine, Day 10). Results are sorted by FDR adjusted p-value and Jaccard similarity index.

Gene Set Name	Gene Set Genes #	DE Genes N (%)	Up-reg. DE Genes N (%)	Down-reg. DE Genes N (%)	Jaccard Index	P-Value	FDR Adjusted P-Value
GO HEMOGLOBIN COMPLEX	12	4 (33.3)	4 (33.3)	0 (0)	0.093	<0.0001	0.0195
GO ENDOCYTIC VESICLE LUMEN	17	3 (17.6)	3 (17.6)	0 (0)	0.061	<0.0001	0.0195
GO BLOOD MICROPARTICLE	118	5 (4.2)	5 (4.2)	0 (0)	0.034	<0.0001	0.0195
GO ANCHORED COMPONENT OF MEMBRANE	149	4 (2.7)	4 (2.7)	0 (0)	0.022	0.0003	0.035
GO EXTRACELLULAR SPACE	1339	10 (0.7)	10 (0.7)	0 (0)	0.007	0.0003	0.035
GO CYTOSOLIC PART	220	4 (1.8)	4 (1.8)	0 (0)	0.016	0.0009	0.0876

Table 131: Enriched MSigDB Cellular Components (PBMC, Trivalent Influenza Vaccine, Day 10). Results are sorted by FDR adjusted p-value and Jaccard similarity index.

Gene Set Name	Gene Set Genes #	DE Genes N (%)	Up-reg. DE Genes N (%)	Down-reg. DE Genes N (%)	Jaccard Index	P-Value	FDR Adjusted P-Value
GSE9006 HEALTHY VS TYPE 1 DIABETES PBMC AT DX DN	191	14 (7.3)	14 (7.3)	0 (0)	0.066	<0.0001	0.0304
GSE29615 CTRL VS LAIV FLU VACCINE PBMC UP	189	11 (5.8)	11 (5.8)	0 (0)	0.052	<0.0001	0.0304
GSE13485 PRE VS POST YF17D VACCINATION PBMC UP	184	9 (4.9)	9 (4.9)	0 (0)	0.043	<0.0001	0.0304
GSE22886 NEUTROPHIL VS MONOCYTE UP	195	9 (4.6)	9 (4.6)	0 (0)	0.041	<0.0001	0.0304
GSE13485 CTRL VS DAY7 YF17D VACCINE PBMC UP	179	8 (4.5)	8 (4.5)	0 (0)	0.039	<0.0001	0.0304
GSE29615 CTRL VS DAY7 LAIV FLU VACCINE PBMC UP	184	8 (4.3)	8 (4.3)	0 (0)	0.038	<0.0001	0.0304
GSE22886 NAIVE BCELL VS NEUTROPHIL DN	197	8 (4.1)	8 (4.1)	0 (0)	0.036	<0.0001	0.0304
GSE22886 NAIVE TCELL VS NEUTROPHIL DN	197	8 (4.1)	8 (4.1)	0 (0)	0.036	<0.0001	0.0304
GSE3982 DC VS NEUTROPHIL DN	198	6 (3)	6 (3)	0 (0)	0.026	<0.0001	0.0304
GSE13485 CTRL VS DAY3 YF17D VACCINE PBMC UP	175	5 (2.9)	5 (2.9)	0 (0)	0.024	<0.0001	0.0304
GSE29615 CTRL VS DAY3 LAIV IFLU VACCINE PBMC UP	189	5 (2.6)	5 (2.6)	0 (0)	0.023	<0.0001	0.0304
GSE22886 NEUTROPHIL VS DC UP	197	5 (2.5)	5 (2.5)	0 (0)	0.022	<0.0001	0.0304
GSE41176 UNSTIM VS ANTI IGM STIM TAK1 KO BCELL 24H...	199	5 (2.5)	5 (2.5)	0 (0)	0.022	<0.0001	0.0304
GSE13485 DAY7 VS DAY21 YF17D VACCINE PBMC DN	183	4 (2.2)	4 (2.2)	0 (0)	0.019	<0.0001	0.0304
GSE3982 EOSINOPHIL VS NEUTROPHIL DN	192	4 (2.1)	4 (2.1)	0 (0)	0.018	<0.0001	0.0304
GSE18281 SUBCAPSULAR VS CENTRAL CORTICAL REGION OF...	200	4 (2)	4 (2)	0 (0)	0.017	<0.0001	0.0304
GSE3039 CD4 TCELL VS B2 BCELL UP	201	5 (2.5)	5 (2.5)	0 (0)	0.022	0.0002	0.0541

Gene Set Name	Gene Set Genes #	DE Genes N (%)	Up-reg. DE Genes N (%)	Down-reg. DE Genes N (%)	Jaccard Index	P-Value	FDR Adjusted P-Value
GSE34205 HEALTHY VS RSV INF INFANT PBMC DN	199	4 (2)	4 (2)	0 (0)	0.017	0.0002	0.0541
GSE34205 RSV VS FLU INF INFANT PBMC UP	190	4 (2.1)	4 (2.1)	0 (0)	0.018	0.0003	0.0731
GSE15930 NAIVE VS 48H IN VITRO STIM CD8 TCELL UP	198	4 (2)	4 (2)	0 (0)	0.018	0.0003	0.0731
GSE2405 0H VS 3H A PHAGOCYTOPHILUM STIM NEUTROPHIL...	189	4 (2.1)	4 (2.1)	0 (0)	0.018	0.0004	0.0779
GSE22886 NAIVE CD8 TCELL VS NEUTROPHIL DN	194	4 (2.1)	4 (2.1)	0 (0)	0.018	0.0004	0.0779
GSE3982 NEUTROPHIL VS BCELL UP	196	4 (2)	4 (2)	0 (0)	0.018	0.0004	0.0779
GSE3039 NKT CELL VS ALPHAALPHA CD8 TCELL UP	199	4 (2)	4 (2)	0 (0)	0.017	0.0004	0.0779
GSE360 L DONOVANI VS M TUBERCULOSIS DC DN	199	4 (2)	4 (2)	0 (0)	0.017	0.0004	0.0779

Table 132: Enriched MSigDB Immunological Signatures (PBMC, Trivalent Influenza Vaccine, Day 10). Results are sorted by FDR adjusted p-value and Jaccard similarity index.

Gene Set Name	Gene Set Genes #	DE Genes N (%)	Up-reg. DE Genes N (%)	Down-reg. DE Genes N (%)	Jaccard Index	P-Value	FDR Adjusted P-Value
GO OXYGEN TRANSPORTER ACTIVITY	14	4 (28.6)	4 (28.6)	0 (0)	0.089	<0.0001	0.0186
GO OXYGEN BINDING	47	5 (10.6)	5 (10.6)	0 (0)	0.065	<0.0001	0.0186
GO OXIDOREDUCTASE ACTIVITY ACTING ON PEROXIDE AS A...	41	3 (7.3)	3 (7.3)	0 (0)	0.041	<0.0001	0.0186
GO TETRAPYRROLE BINDING	133	5 (3.8)	5 (3.8)	0 (0)	0.031	<0.0001	0.0186
GO IRON ION BINDING	162	5 (3.1)	5 (3.1)	0 (0)	0.026	<0.0001	0.0186
GO ANTIOXIDANT ACTIVITY	69	3 (4.3)	3 (4.3)	0 (0)	0.030	0.0002	0.031

Table 133: Enriched MSigDB Molecular Functions (PBMC, Trivalent Influenza Vaccine, Day 10). Results are sorted by FDR adjusted p-value and Jaccard similarity index.

Package Name	Version	Package Name	Version	Package Name	Version
nlme	3.1-131	BiocParallel	1.10.1	IRanges	2.10.3
bitops	1.0-6	RCurl	1.95-4.8	cluster	2.0.6
matrixStats	0.52.2	magrittr	1.5	Cairo	1.5-9
pbkrtest	0.4-7	GO.db	3.4.1	UpSetR	1.3.3
bit64	0.9-7	GenomeInfoDbData	0.99.0	gridExtra	2.3
GenomeInfoDb	1.12.2	Matrix	1.2-10	plyr	1.8.4
tools	3.4.1	Rcpp	0.12.12	stringr	1.2.0
KernSmooth	2.23-15	munsell	0.4.3	gtools	3.5.0
DBI	0.7	S4Vectors	0.14.4	R.utils	2.5.0
BiocGenerics	0.22.0	stringi	1.1.5	R.oo	1.21.0
lazyeval	0.2.0	SummarizedExperiment	1.6.4	R.methodsS3	1.7.1
mgcv	1.8-17	zlibbioc	1.22.0	vegan	2.4-4
colorspace	1.3-2	blob	1.1.0	lattice	0.20-35
nnet	7.3-12	parallel	3.4.1	permute	0.9-4
bit	1.1-12	gdata	2.18.0	car	2.1-5
compiler	3.4.1	Biostrings	2.44.2	pvclust	2.0-0
chron	2.3-50	splines	3.4.1	sqldf	0.4-11
quantreg	5.33	GenomicFeatures	1.28.5	RSQLite	2.0
Biobase	2.36.2	locfit	1.5-9.1	gsubfn	0.6-6
SparseM	1.77	GenomicRanges	1.28.5	proto	1.0.0
DelayedArray	0.2.7	codetools	0.2-15	gplots	3.0.1
rtracklayer	1.36.4	stats4	3.4.1	MASS	7.3-47
labeling	0.3	XML	3.98-1.9	xtable	1.8-2
caTools	1.17.1	evaluate	0.10.1	goseq	1.28.0
scales	0.5.0	nloptr	1.0.4	geneLenDataBase	1.12.0
digest	0.6.12	MatrixModels	0.4-1	BiasedUrn	1.07
Rsamtools	1.28.0	gtable	0.2.0	biomaRt	2.32.1
minqa	1.2.4	ggplot2	2.2.1	edgeR	3.18.1
XVector	0.16.0	tibble	1.3.4	limma	3.32.7
pkgconfig	2.0.1	GenomicAlignments	1.12.2	knitr	1.17
lme4	1.1-13	AnnotationDbi	1.38.2		
rlang	0.1.2	memoise	1.1.0		

Table 134: List of R packages and versions used for the analyses presented in this report. R version 3.4.1 (2017-06-30) 'Single Candle
