# Supplementary material for: RSEQREP: RNA-Seq Reports, an open-source cloud-enabled framework for reproducible RNA-Seq data processing, analysis, and result reporting
Source: F1000Res. 2018 Apr 13;6:2162. Originally published 2017 Dec 21. [Version 2] doi: 10.12688/f1000research.13049.2 (PMC6039931; doi:10.12688/f1000research.13049.2)
Supplement: Peer review history

# Peer review: Referee response for version 2

**DOI:** 10.5256/f1000research.15754.r33142

**Published:** 2018-07-10

**Authors:** Ben Busby

**Affiliations:** 1 National Center for Biotechnology Information, National Library of Medicine, National Institutes of Health (NIH), Bethesda, MD, USA

Its great to see that this is available on dockerhub and bioconductor!

 In my opinion, this is approvable for indexing!  

 That said, I have one additional comment (that should not stand in the way of indexing).  

 I like the way the perlscript for preprocessing is written, but in line 135, where it tries to fastq-dump out of SRA when it cant find the other files, I would highly encourage you to try streaming out of SRA using https://github.com/ncbi/ngs rather than fastq-dump.

I have read this submission. I believe that I have an appropriate level of expertise to confirm that it is of an acceptable scientific standard.

# Peer review: Referee response for version 2

**DOI:** 10.5256/f1000research.15754.r33143

**Published:** 2018-06-26

**Authors:** Anup Mahurkar

**Affiliations:** 1 ​ Institute for Genome Sciences, University of Maryland School of Medicine, Baltimore, MD, USA

The updates to documentation and explanation provided by the authors makes it a lot easier to use and clearer to understand. This is a useful tool that can benefit functional genomics research.

I have read this submission. I believe that I have an appropriate level of expertise to confirm that it is of an acceptable scientific standard.

# Peer review: Referee response for version 1

**DOI:** 10.5256/f1000research.14148.r29277

**Published:** 2018-01-31

**Authors:** Ben Busby

**Affiliations:** 1 National Center for Biotechnology Information, National Library of Medicine, National Institutes of Health (NIH), Bethesda, MD, USA

This work is an important example of building and advertising easy-to-implement yet powerful bioinformatics pipelines with established, popular algorithms.  

 That said, in my opinion, three minor additions would make this manuscript much more robust, and therefore, acceptable for publications.

 First, the authors state that these pipelines should work on a variety of cloud architectures, but all they explicitly provide is an AMI for AWS use.  Containerization through Docker (not an endorsement) or some other containerization protocol should simply bridge this gap.

 Second, there are many mentions of bioconductor, but it is not clear from the manuscript whether this particular pipeline is available in bioconductor.  Please clarify.

 Third, given that the components of such pipelines are continuously evolving, there should be some documentation, either in the manuscript or the github repo about switching in new modules for mapping, differential expression, or visualization.

I have read this submission. I believe that I have an appropriate level of expertise to confirm that it is of an acceptable scientific standard, however I have significant reservations, as outlined above.

# Response

**Published:** 2018-03-30

**Authors:** Johannes Goll

**Affiliations:** The Emmes Coporation, USA

We thank Ben Busby for his insightful comments and suggestions to increase the usefulness of this software. In the following, we address each of the reviewer's comments highlighted in bold:

**First, the authors state that these pipelines should work on a variety of cloud architectures, but all they explicitly provide is an AMI for AWS use.  Containerization through Docker (not an endorsement) or some other containerization protocol should simply bridge this gap.**

 To increase the software's portability, we created a public RSEQREP Docker container that contains all the required 3rd party software. The container image is available at https://hub.docker.com/r/emmesdock/rseqrep and can be accessed via the Docker utility using “docker pull emmesdock/rseqrep”.

**Second, there are many mentions of bioconductor, but it is not clear from the manuscript whether this particular pipeline is available in bioconductor.  Please clarify.**

 RSEQREP is not available as a Bioconductor R package and includes components not written in R. The source code is available at https://github.com/emmesgit/RSEQREP

1. **Third, given that the components of such pipelines are continuously evolving, there should be some documentation, either in the manuscript or the github repo about switching in new modules for mapping, differential expression, or visualization.**

 

 We agree that RNA-Seq data processing and analysis is a constantly evolving field and there is no consensus on how to best analyze the data. RSEQREP includes a collection of best practice analytical tools that we identified through extensive review of the peer-reviewed literature. Users have complete access to the RSEQREP source code to make custom updates to all workflow, analysis, and reporting components. In combination with scalable cloud resources this allows for rapid prototyping of analysis reports.

 Below are key RSEQREP programs to alter mapping, differential expression, visualizations, or tables:

 1) Read mapping is executed via “RSEQREP\source\perl\preprocess-rnaseq.pl”

 2) DE gene analysis is executed via “RSEQREP/source/r/02-sdeg-identification/init-edgeR-glm-model.r”

 3) Integration of visualizations (R graphics) as part of the report is handled via “/RSEQREP/source/knitr/*figures.Rnw” files.  

 4) Integration of tables (R xtable) as part of the report is handled via “/RSEQREP/source/knitr/*tables.Rnw” files.

# Peer review: Referee response for version 1

**DOI:** 10.5256/f1000research.14148.r29275

**Published:** 2018-01-19

**Authors:** Anup Mahurkar

**Affiliations:** 1 ​ Institute for Genome Sciences, University of Maryland School of Medicine, Baltimore, MD, USA

RSEQREP is a comprehensive RNAseq analysis pipeline for processing bulk human RNAseq data. The pipeline bundles a number of commonly used RNAseq analysis tools to create a single pipeline for end-to-end RNAseq data analysis. The pipeline includes tools such fastqc for data QC, STAR and Hisat for alignment, RSeQC for generating alignment stats, edgeR for differential gene expression, a number of R packages for clustering analysis, and GOSeq for gene set enrichment analysis. The output from all of these tools is then used to generate a comprehensive report that summarizes the data analysis. The pipeline can be downloaded locally and run in an Ubuntu VM or can be executed on Amazon using a prebuilt Amazon Machine Image (AMI).

 The article is well written with an example dataset used to illustrate the different outputs generated by the pipeline. This is a useful tool that will help small labs with limited bioinformatics expertise or computational resources run RNAseq analyses. The authors also provide documentation on the github repository. The configuration of the pipeline is made easy through an Excel template provided in the repository.

 While there are other similar tools and pipelines this is one of the few that bundles everything into a single pipeline and could be a great tool for biologist and bioinformaticians. Following are some suggestions that might improve the tool:

  

1. The authors have provided a number of useful visualizations including Heatmaps, UpSet plots, radar plots, and PCA plots, some commonly used plots in RNAseq analysis such as MA plots, or Volcano plots are not present. The authors could consider adding these plots to the package.
2. The generated report includes summary figures for the different analyses and tables with the list of genes and pathways detected through the analyses. For a large analysis like the time-series analysis used as an example the generated report includes over 300 pages. This makes it hard to navigate through the report and find things quickly. Also, it is hard to explore the DE results by changing log-fold cutoffs, or FDR cutoffs which could easily be done in a spreadsheet but not in a PDF. The authors might want to consider splitting the report in two parts, a PDF report with images and summary data and methods, and an accompanying workbook with the tables so users can explore the results.
3. The tool requires users to build the indexes for the database used as the reference genome. This typically requires a higher memory machine (16 GB for Hisat2, and 37 GB for STAR). The processing itself can be done with less memory. This could pose a problem for users trying to run this tool on a typical laptop or workstation. The authors could consider prebuilding some commonly used indices for human, mouse, and rat genomes so all users do not have to re-index. These references are relatively stable so the index may only need to be rebuilt once or twice a year. This will ease the burden significantly for end users.
4. The authors have benchmarked their tool on Amazon to identify the most optimal instance type (Figure 6) so users can minimize costs. The biggest performance gains seem to be when the machine instance is changed from 4 vCPUs to 8 vCPUs. However, if a user were to want to use STAR this instance type does not have sufficient RAM. To optimize, the user will need to either use a larger instance for the entire processing, or launch two instances, a larger instance for the indexing step, and a smaller instance for processing, and copy the indices. For this reason, having pre-built indices might alleviate this issue.
5. The documentation needs some improvement, particularly if the intended audience is users with limited computational experience. When I tried to launch the AMI on Amazon I was not sure what username to use to log into the running instance. Through trial and error, I figured out that the username was “ubuntu”. But it would be better if this were included in the documentation on the github repo.
6. Another related issue is that because the pipeline reads Excel config files the user needs to create the config file on the local machine and upload to the AMI. Most non-tech savvy users will not necessarily know how to do it easily. The documentation could point to some utilities that could be used to upload the edited file such as sftp.
7. It appears that the system is only setup for human genome analysis. While editing the config file it was not clear where to specify the reference genome information for other organisms. There is no reason the pipeline could not work for other model organisms which are commonly used for basic research studies. This will increase its adaption and userbase.
8. Once I had the AMI running I had a difficult time executing the test pipeline. I was getting errors about missing directories or data files. I would recommend that the authors test the AMI and have clearer instructions on how to download datasets and run the tools in the AMI. For instance after cloning the github repo I tried running the setup script with the command “sh RSEQREP/setup.sh” while I was in “/home/ubuntu” and got the following error:

“Fatal error: cannot open file '/home/ubuntu/source/r/parse-rnaseq-configuration.r': No such file or directory”.

 I then tried moving to the RSEQREP directory to run the same command and got the error:

 “File does not exist! /home/ubuntu/msigdb/c2.cp.kegg.v5.2.entrez.gmt.”

 Based on the error message it appears that the software expects the databases to be uploaded before the setup script can be run but the documentation does not specify that. As a result, I was not able to test the VM end-to-end, but with improved testing and documentation this should be easy to address.

I have read this submission. I believe that I have an appropriate level of expertise to confirm that it is of an acceptable scientific standard, however I have significant reservations, as outlined above.

# Response

**Published:** 2018-03-30

**Authors:** Johannes Goll

**Affiliations:** The Emmes Coporation, USA

We thank Anup Mahurkar for his insightful comments and suggestions to increase the usefulness of this software. In the following, we address each of the reviewer's comments highlighted in bold:

**The authors have provided a number of useful visualizations including Heatmaps, UpSet plots, radar plots, and PCA plots, some commonly used plots in RNAseq analysis such as MA plots, or Volcano plots are not present. The authors could consider adding these plots to the package.**

The Volcano and MA plots can be found on page Figures 20-23 in the case study report (Supplementary File S1).

**The generated report includes summary figures for the different analyses and tables with the list of genes and pathways detected through the analyses. For a large analysis like the time-series analysis used as an example the generated report includes over 300 pages. This makes it hard to navigate through the report and find things quickly. Also, it is hard to explore the DE results by changing log-fold cutoffs, or FDR cutoffs which could easily be done in a spreadsheet but not in a PDF. The authors might want to consider splitting the report in two parts, a PDF report with images and summary data and methods, and an accompanying workbook with the tables so users can explore the results.**

We recommend that users navigate to the figure and table listings at the beginning of the PDF report to find content of interest or use the PDF search option. RSEQREP provides user-friendly configuration options via a spreadsheet (see for example Supplementary File S2) including FDR and fold change cut offs that can be adjusted prior to generating reports. In addition to the PDF report, RSEQREP outputs all tables including DE gene lists in comma-separated values (CSV) format which then can be used within Excel or other spreadsheet software to dynamically filter DE gene lists.

**The tool requires users to build the indexes for the database used as the reference genome. This typically requires a higher memory machine (16 GB for Hisat2, and 37 GB for STAR). The processing itself can be done with less memory. This could pose a problem for users trying to run this tool on a typical laptop or workstation. The authors could consider prebuilding some commonly used indices for human, mouse, and rat genomes so all users do not have to re-index. These references are relatively stable so the index may only need to be rebuilt once or twice a year. This will ease the burden significantly for end users.**

We agree that a pre-built index would be more computational effective. However, we consider generating the index a part of the start-to-end analysis and, as a critical step to support reproducible research, indexing software, genome, and genome annotation versions are fully captured. This approach provides also the most flexibility to the users who can specify any Ensembl version including associated annotations and reference genome assembly during the initialization phase (see for example Supplementary File S2). A process to manually maintain genome indices would quickly become out of date.

**The authors have benchmarked their tool on Amazon to identify the most optimal instance type (Figure 6) so users can minimize costs. The biggest performance gains seem to be when the machine instance is changed from 4 vCPUs to 8 vCPUs. However, if a user were to want to use STAR this instance type does not have sufficient RAM. To optimize, the user will need to either use a larger instance for the entire processing, or launch two instances, a larger instance for the indexing step, and a smaller instance for processing, and copy the indices. For this reason, having pre-built indices might alleviate this issue.**

Since we completed our computational benchmarks, newer AWS EC2 instance types have become available. Most notably the r4.X and x1e.X instance types, which are more than capable of providing enough memory for indexing with 4 or 8 vCPUs.

**The documentation needs some improvement, particularly if the intended audience is users with limited computational experience. When I tried to launch the AMI on Amazon I was not sure what username to use to log into the running instance. Through trial and error, I figured out that the username was “ubuntu”. But it would be better if this were included in the documentation on the github repo.**

We have updated our README file on GitHub ( https://github.com/emmesgit/RSEQREP). We added detailed information about the AMI, installation, execution, and troubleshooting. This includes details on which user name and password to use to login into the AMI.

**Another related issue is that because the pipeline reads Excel config files the user needs to create the config file on the local machine and upload to the AMI. Most non-tech savvy users will not necessarily know how to do it easily. The documentation could point to some utilities that could be used to upload the edited file such as sftp.**

We provide the Amazon AMI preconfigured with the X2GO remote Desktop software. This allows users to connect to the instance using a user-friendly desktop environment. We have updated our README file on GitHub ( https://github.com/emmesgit/RSEQREP) to include information about this. Additionally, the instance comes pre-configured with the Libre Office software which contains an Excel editor.

**It appears that the system is only setup for human genome analysis. While editing the config file it was not clear where to specify the reference genome information for other organisms. There is no reason the pipeline could not work for other model organisms which are commonly used for basic research studies. This will increase its adaption and userbase.**

We purposefully designed the software to support human clinical research studies. While at this time, it supports only RNA-Seq analysis of human clinical samples, we may add support to expand on this in the future. We do encourage users to modify the existing source code for their own purposes including adaptations to support RNA-Seq analyses for other model organisms.

**Once I had the AMI running I had a difficult time executing the test pipeline. I was getting errors about missing directories or data files. I would recommend that the authors test the AMI and have clearer instructions on how to download datasets and run the tools in the AMI. For instance after cloning the github repo I tried running the setup script with the command “sh RSEQREP/setup.sh” while I was in “/home/ubuntu” and got the following error:** **“Fatal error: cannot open file '/home/ubuntu/source/r/parse-rnaseq-configuration.r': No such file or directory”.**

****

**I then tried moving to the RSEQREP directory to run the same command and got the error:**

****

**“File does not exist! /home/ubuntu/msigdb/c2.cp.kegg.v5.2.entrez.gmt.”**

****

**Based on the error message it appears that the software expects the databases to be uploaded before the setup script can be run but the documentation does not specify that. As a result, I was not able to test the VM end-to-end, but with improved testing and documentation this should be easy to address.**

We have updated our README file on GitHub ( https://github.com/emmesgit/RSEQREP). We added detailed information about using the AMI, installation, execution, and troubleshooting. We also updated the run-* scripts to be executed anywhere on the file system. Prior to running RSEQREP, most suitable gene set datasets (GTM files) for the pathway enrichment analysis (e.g. Reactome pathways, KEGG pathways, MSigDB pathways, etc.) need to be identified and downloaded by the user and added to the RSEQREP configuration file. To make it easier for users, we provide programs to download certain gene sets files (see our README file on GitHub page for further documentation).
